# Parallel point-multiplication architecture using combined group operations for high-speed cryptographic applications

**DOI:** 10.1371/journal.pone.0176214

**Published:** 2017-05-01

**Authors:** Md Selim Hossain, Ehsan Saeedi, Yinan Kong

**Affiliations:** Department of Engineering, Macquarie University, Sydney, NSW, Australia; University of Texas at San Antonio, UNITED STATES

## Abstract

In this paper, we propose a novel parallel architecture for fast hardware implementation of elliptic curve point multiplication (ECPM), which is the key operation of an elliptic curve cryptography processor. The point multiplication over binary fields is synthesized on both FPGA and ASIC technology by designing fast elliptic curve group operations in Jacobian projective coordinates. A novel combined point doubling and point addition (PDPA) architecture is proposed for group operations to achieve high speed and low hardware requirements for ECPM. It has been implemented over the binary field which is recommended by the National Institute of Standards and Technology (NIST). The proposed ECPM supports two Koblitz and random curves for the key sizes 233 and 163 bits. For group operations, a finite-field arithmetic operation, e.g. multiplication, is designed on a polynomial basis. The delay of a 233-bit point multiplication is only 3.05 and 3.56 *μ*s, in a Xilinx Virtex-7 FPGA, for Koblitz and random curves, respectively, and 0.81 *μ*s in an ASIC 65-nm technology, which are the fastest hardware implementation results reported in the literature to date. In addition, a 163-bit point multiplication is also implemented in FPGA and ASIC for fair comparison which takes around 0.33 and 0.46 *μ*s, respectively. The area-time product of the proposed point multiplication is very low compared to similar designs. The performance ($\frac{1}{Area\times Time}=\frac{1}{AT}$) and Area × Time × Energy (ATE) product of the proposed design are far better than the most significant studies found in the literature.

## 1 Introduction

With the swift growth of secure transactions over the network, the demand for cryptography to ensure security has increased rapidly in recent times. Public-key cryptography (PKC) and secret-key cryptography are the two main types of cryptography used for different data-security purposes. Various PKC techniques exist in the literature; among them elliptic curve cryptography (ECC) [1, 2] and the Rivest-Shamir-Adleman (RSA) cryptosystem [3, 4] are the most popular. However, ECC became popular for resource-constrained environments because it offers the same level of security as the traditional RSA cryptosystem with a significantly shorter key. For example, a 233-bit ECC over a binary field provides equivalent security to 2048-bit RSA [5–7]. The National Institute of Standards and Technology (NIST) [7] and IEEE [6], have standardized elliptic curve parameters for prime fields as well as binary fields. The proposed point multiplication hardware is implemented using the NIST standard on an FPGA, which provides higher flexibility of hardware design than an application-specific integrated circuit (ASIC), and means that the cryptographic algorithm can easily be updated if using FPGAs as hardware devices. Also, FPGAs are cheaper for prototype design or in small volumes since they do not incur any fabrication cost. However, bulk production (e.g. in high volumes) of ASICs, after the first run, is much cheaper than the corresponding production based on FPGA devices. Besides, ASIC-based implementation is needed for faster and low-power customized applications.

Elliptic curve point multiplication (ECPM), also called point multiplication, is defined as *Q* = *k*.*P*, where the multiplication of an elliptic curve point *P* by a scalar *k* provides the resultant point *Q* [5]. Numerous FPGA implementations of point multiplication over a binary field GF(2^*m*^) have been proposed in the literature, e.g. [8–25]. In the literature, most of the implementations of ECPM over GF(2^163^) are not secure based on today’s security level requirements. For this reason, a 233-bit point multiplication is implemented both in FPGA and ASIC. In addition, a 163-bit ECPM is implemented for a fair comparison purpose. In [8, 11, 12], a scalable elliptic curve cryptosystem processor in GF(2^*m*^) is proposed which reduces the latency of ECPM by improving finite-field arithmetic blocks. A Xilinx Virtex-5 FPGA is used in [8] and a Xilinx Virtex-4 FPGA is used in [11, 12] as a hardware platform. However, they have not focused on optimization of elliptic curve group operations in their design. An FPGA implementation of ECPM based on the Montgomery ladder method over binary fields is proposed in [9] and [21]. They designed the point multiplication using elliptic curve point addition (PA) and point doubling (PD). An efficient FPGA implementation of ECPM over binary finite fields is proposed in [10, 13, 15–17]. Among them [10] produces better results using digit-serial binary field operations. In [13], a point multiplication was designed in GF(2^163^) for Koblitz curves only. In [14, 18] and [22], a parallel architecture for scalar point multiplication was implemented on a Xilinx Virtex-4 FPGA using the Lopez-Dahab method and separate PA and PD. A practical hardware implementation of point multiplication over GF(2^163^) is proposed using polynomial residue arithmetic in [19]. Several ASIC-Based ECC processors have been proposed over the binary fields in the literature [10, 18, 26–33]. ECC can be used for modern practical applications like mobile services [34], authentication for identity protection for smart grid, wireless sensor and mesh networks [35–37], biometric-based authentication [38], identity-based cryptography [39], and session initiation protocol [40].

Various techniques are introduced, using either FPGA or ASIC implementation, to improve the performance of point multiplication, such as algorithm optimization and improved finite-field arithmetic architectures. Besides, most point multiplication architectures were implemented using separate group operations, which may increase the latency of group operations, hence reduce the speed of point multiplication. Although a few high-speed point-multiplication techniques for an ECC processor have been presented in the literature, most are only area-efficient. Our proposed architecture has a trade-off between speed and area which is suitable for modern faster cryptographic applications.

Contributions: This paper proposes a parallel hardware architecture for point multiplication using combined point doubling and point addition (PDPA) in Jacobian projective coordinates. The proposed point multiplication is synthesized both in FPGA and ASIC. A novel optimized data-flow architecture of the PDPA is introduced to develop high-performance point multiplication. The designed PDPA module is highly parallel, which means that it takes only one clock cycle to complete. In addition, a parallel hardware architecture using separate group operations (PD and PA) for the ECPM is designed and implemented, and compared with the performance of point multiplication using our combined PDPA. The point multiplication using the combined PDPA provides almost 13 times better performance than using separate group operations. To implement efficient group operations, hence point multiplication, a parallel architecture for field multiplication on a polynomial basis is introduced. The proposed point multiplication requires less time and a smaller area-time (AT) and area-time-energy (ATE) product, providing almost 50% better performance or efficiency than recent implementations.

This paper is organized as follows. Section 2 gives an introduction and the mathematical background of ECC over the binary field $F{}_{2}{}^{m}$. The proposed point multiplication architecture is described in Section 3. Section 4 describes elliptic curve group operations, namely PD, PA, and PDPA. Finite-field arithmetic, e.g. field multiplication, for $F{}_{2}{}^{m}$ is given in Section 5. Section 6 discusses the FPGA and ASIC implementation results and compares our work to the state of the art. Section 7 summarizes our work.

## 2 ECC background

ECC is a popular and powerful public-key encryption technique for cryptographic applications, and nowadays it is very popular due to the smaller field size, in either prime fields or binary fields. An elliptic curve over a binary field will be the emphasis of this work because it is very efficient for hardware implementation due to the use of modulo-2 arithmetic. An elliptic curve defined over a finite field provides a group structure that is used to implement the cryptographic system. The group operations are PD and PA. We have combined these two group operations into a compact hardware implementation and called it PDPA. Two well-known coordinate systems are often used for elliptic curve group operations: Affine coordinate systems and projective coordinate systems. A point on the elliptic curve E for affine coordinates can be represented by using two elements *x*, *y* ∈ $F{}_{2}{}^{m}$, i.e. P(*x*, *y*), whereas in projective coordinates, a point P on the EC needs three elements $X,Y,Z\in F{}_{2}{}^{m}$, i.e. P(*X*, *Y*, *Z*). In this work, we have implemented all elliptic curve operations in a Jacobian projective coordinate system, avoiding costly modular inversion.

An elliptic curve E over the binary field GF(2^*m*^) (or $F{}_{2}{}^{m}$) in affine coordinates is the set of solutions to the equation

$$\begin{array}{c} y^{2}+xy=x^{3}+ax^{2}+b \end{array}$$ (1)

where *x*, *y*, *a*, *b* ∈ *GF*(2^*m*^), *b* ≠ 0. The coefficients *a*, *b* ∈ $F{}_{2}{}^{m}$ are defined by the NIST standard, which is listed in [5, 7]. In our design, the value of *m* is 163 which means that we have implemented a 163-bit ECC system.

Let *P* = (*x*, *y*) be a point in an affine coordinate system; the Jacobian projective coordinates *P* = (*X*, *Y*, *Z*) are given by

$$\begin{array}{c} X=x;\;Y=y;\;Z=1. \end{array}$$ (2)

The Jacobian projective point *P* = (*X*, *Y*, *Z*), *Z* ≠ 0 corresponding to the affine point *P* = (*x*, *y*) is given by

$$\begin{array}{c} x=X/Z^{2};\;y=Y/Z^{3}. \end{array}$$ (3)

Using Eqs (1) and (3), the projective form of the Weierstrass equation of the elliptic curve becomes

$$\begin{array}{c} Y^{2}+XYZ=X^{3}+aX^{2}Z^{2}+bZ^{6} \end{array}$$ (4)

where the point at infinity is defined as (1, 1, 0). Let *P* = (*X*_1_, *Y*_1_, *Z*_1_) and *Q* = (*X*_2_, *Y*_2_, *Z*_2_) be two points on the elliptic curve, then the PD and PA formulae in Jacobian projective coordinates are given below, for doubling Eq (5) and adding Eq (6)

$$\begin{array}{ccc} R(X_{3},Y_{3},Z_{3}) & = & 2P\left(X_{1},Y_{1},Z_{1}\right)\in E\left(F{}_{2}{}^{m}\right),\\ Z_{3} & = & X_{1}Z_{1}^{2},\;\\ X_{3} & = & (X_{1}^{4}+bZ_{1}^{8}),\\ Y_{3} & = & X_{1}^{4}Z_{3}+\left(X_{1}^{2}+Y_{1}Z_{1}+Z_{3}\right)X_{3}; \end{array}$$ (5)

$$\begin{array}{ccc} R(X_{3},Y_{3},Z_{3}) & = & P\left(X_{1},Y_{1},Z_{1}\right)+Q\left(X_{2},Y_{2},Z_{2}\right)\in E\left(F{}_{2}{}^{m}\right),\\ Z_{3} & = & Z_{1}Z_{2}W,\\ X_{3} & = & aZ_{3}^{2}+R\left(R+Z_{3}\right)+W^{3},\\ Y_{3} & = & \left(R+Z_{3}\right)X_{3}+Z_{1}^{2}W^{2}\left(RX_{2}+Y_{2}Z_{1}W\right),\\ \text{where}\;\;W & = & \left(X_{1}Z_{2}^{2}+X_{2}Z_{1}^{2}\right)\;\;\text{and}\;\;R=\left(Y_{1}Z_{2}^{3}+Y_{2}Z_{1}^{3}\right). \end{array}$$ (6)

**Fig 1:**
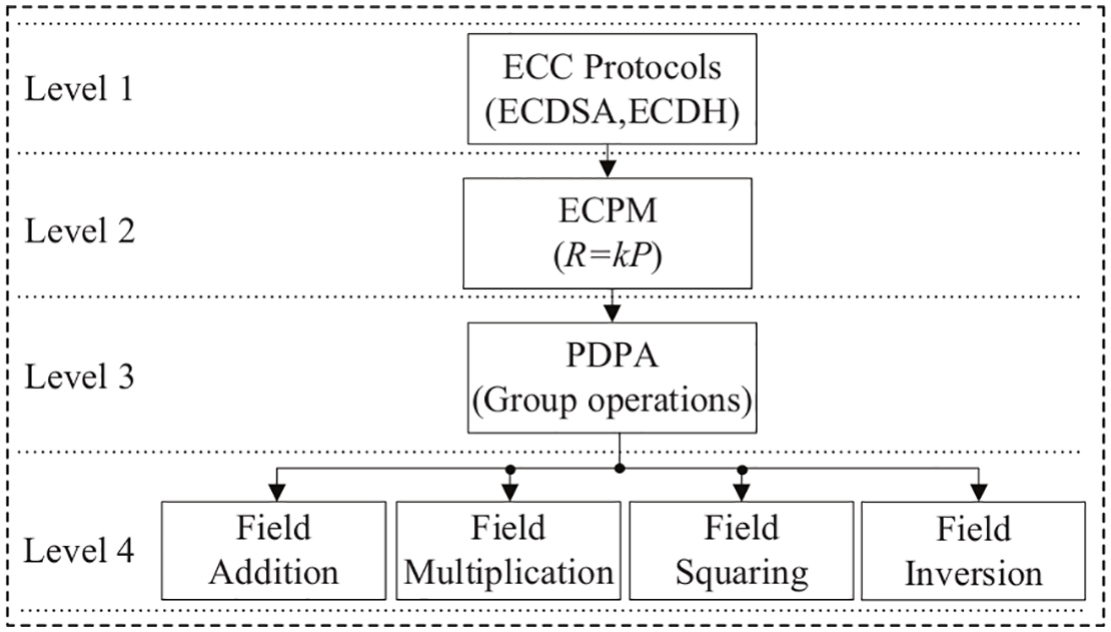
Implementation hierarchy of the ECC operations over $F_{2^{m}}$.

Hence when *P* = *Q*, then *R* = 2*P* is the PD operation corresponding to Eq (5) and when *P* ≠ *Q*, then *R* = *P* + *Q* is the PA operation corresponding to Eq (6) [41]. The implementation hierarchy of the ECC system over the binary field GF(2^*m*^) is presented in Fig 1. From this figure, elliptic curve cryptographic schemes such as ECDSA and ECDH are the building blocks of ECPM and elliptic curve group operations e.g., PDPA. This is the series of finite-field arithmetic operations such as field addition, multiplication, squaring, and inversion. The bottom level is finite-field arithmetic units, which are crucial for the overall performance of an ECC processor. Details of the algorithm, and a hardware architecture for ECPM, are discussed in Section 3.

## 3 Proposed point multiplication in projective coordinates

Point multiplication is the core operation of an ECC processor. It is computationally the most expensive operation throughout the entire processor. However, we have designed a novel parallel architecture for ECPM using our developed PDPA and finite-field arithmetic units. Details of group operations and finite-field arithmetic algorithms and the corresponding architectures which are essential for ECC are discussed in Section 4 and Section 5, respectively.

### 3.1 Point multiplication algorithm

The three most-used algorithms for implementing point multiplication are (1) double-and-add, (2) non-adjacent form (NAF) addition-subtraction chain, and (3) Montgomery ladder product. The easiest to implement is the double-and-add method, shown in Algorithm 1. In this approach, the scalar *k* (which is the private/secret key) is represented in binary, and iterates through each bit. Generally, a PD operation performs on every iteration, and a PA operation only performs when the particular bit of *k* is one. However, we have implemented a combined PDPA operation which produces PD and PA results simultaneously on each cycle. Then *m* iterations are required to compute the final result of ECPM, but each iteration needs only one clock cycle (CC) (CC for PDPA).

**Algorithm 1:** Double-and-add (Left to right) method for ECPM

**Input:** $k={\left(k_{m-1,\cdots ,k_{1},k_{0}}\right)}_{2},\;P\left(X,Y,Z\right)\in E\left(F{}_{2}{}^{m}\right)$

**Output:** *Q*(*X*, *Y*, *Z*) = *k*.*P*(*X*, *Y*, *Z*), where $Q\left(X,Y,Z\right)\in E\left(F{}_{2}{}^{m}\right)$

1.  *Q* = 0;

2.  **for** i = *m* − 1 to 0 **do** *Q* = 2*Q*;

2.1   **if** *k*(*i*) = ‘1’ **then** *Q* = *Q* + *P*; **end**

2.2  **end for**

3.  Return (*Q*(*X*, *Y*, *Z*))

### 3.2 Architecture for ECPM

**Fig 2:**
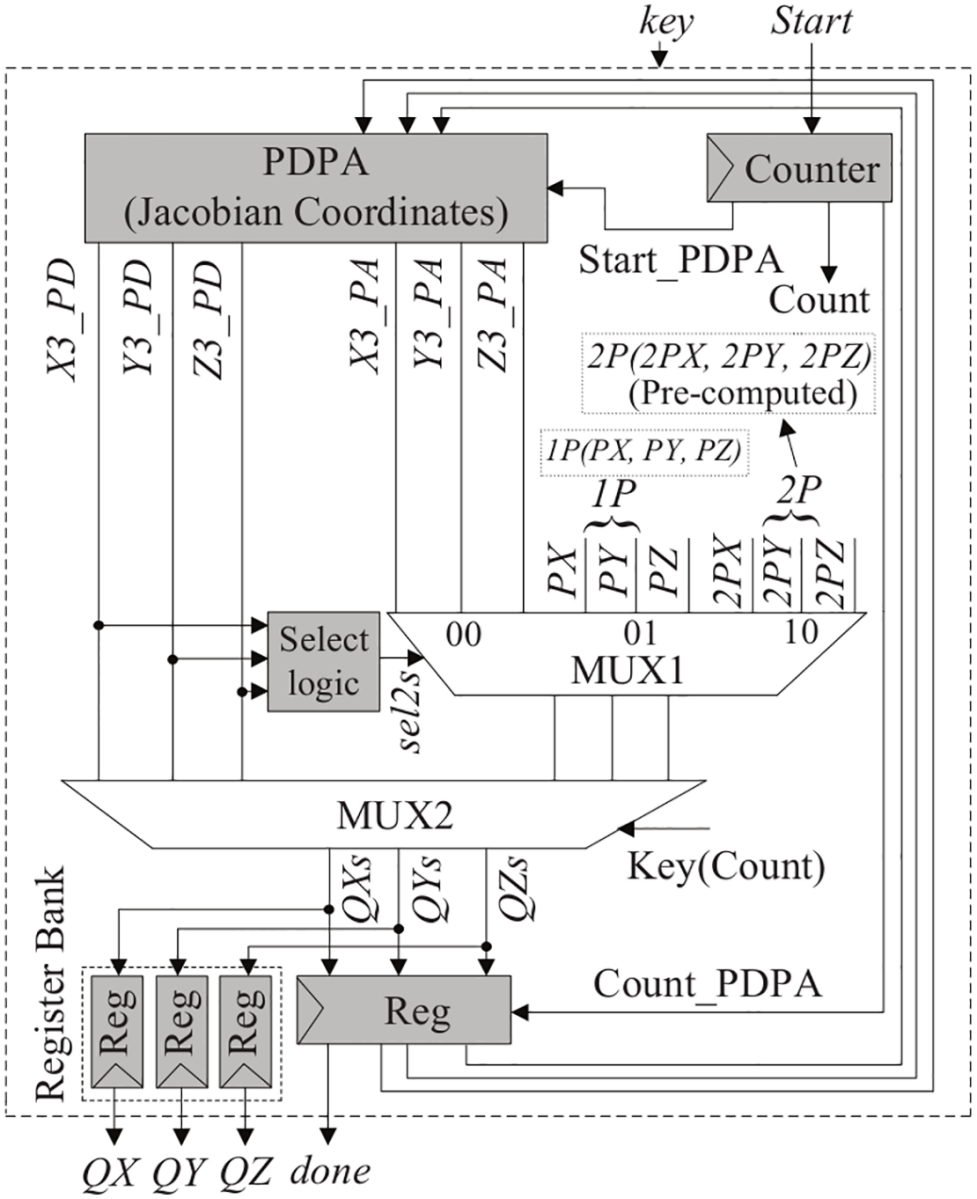
Hardware architecture of proposed ECPM in Jacobian coordinates.

A novel point multiplication architecture is proposed in Jacobian projective coordinates using our designed PDPA architecture, which is highly parallel. Note that most ECC implementations in the literature have used separate PD and PA modules, and require more computation time. The proposed ECPM architecture using PDPA is shown in Fig 2. Our proposed ECPM consists of PDPA, counter, select logic, multiplexer, and register modules. In Fig 2, the PDPA architecture generates the PD and PA results at the same time because it performs the group operations in parallel. For example, when 1*P*(*X*1, *Y*1, *Z*1) is an input, this architecture generates the 2*P*(2*PX*, 2*PY*, 2*PZ*) and 3*P*(3*PX*, 3*PY*, 3*PZ*) results concurrently. In this architecture, the outputs of PDPA are X3_PD, Y3_PD, Z3_PD, which stand for the outputs of PD, and X3_PA, Y3_PA, and Z3_PA, which stand for the outputs of PA. In this approach, the PDPA module is the main component to make a faster point multiplication. As can be seen from Fig 2, a two-bit ‘sel2s’ signal is generated from the select logic unit which is based on PD outputs. When PD results are zero, ‘sel2s = 01’, when PD results are equal to 1*P*(*X*1, *Y*1, *Z*1), then ‘sel2s = 10’, otherwise ‘sel2s = 00’ is produced from the select logic unit. Thus, ‘sel2s’ is a control signal for the MUX1 module that decides which output passes to the MUX2 module. As one can see from MUX1 in Fig 2, are of the 1*P*(*X*1, *Y*1, *Z*1), 2*P*(2*PX*, 2*PY*, 2*PZ*), and PA results, based on the ‘sel2s’ signal, goes to the MUX2 module, which means that when ‘sel2s = 00’, then PA results, when ‘sel2s = 01’, then 1*P*(*X*1, *Y*1, *Z*1), and when ‘sel2s = 10’, then 2*P*(2*PX*, 2*PY*, 2*PZ*) results are selected. The PA result from the PDPA module goes to the output when the particular bit of ‘*key*’ is one. Similarly, the PD result goes to the output when ‘*key*’ is zero. Hence, the PD and PA results are stored in the register bank to get the output. A counter module is used to decide when the results will be passed to the next input of the PDPA module. Note that, the combined PDPA module needs only one clock cycle to compute the PD and PA results concurrently, although it looks to need many logic stages. In this method, only 233 and 163 CCs are needed to compute a 233-bit and 163-bit point multiplication, respectively in projective coordinates due to the highly parallel PDPA architecture, which will be discussed in the next section.

### 3.3 Security analysis

**Fig 3:**
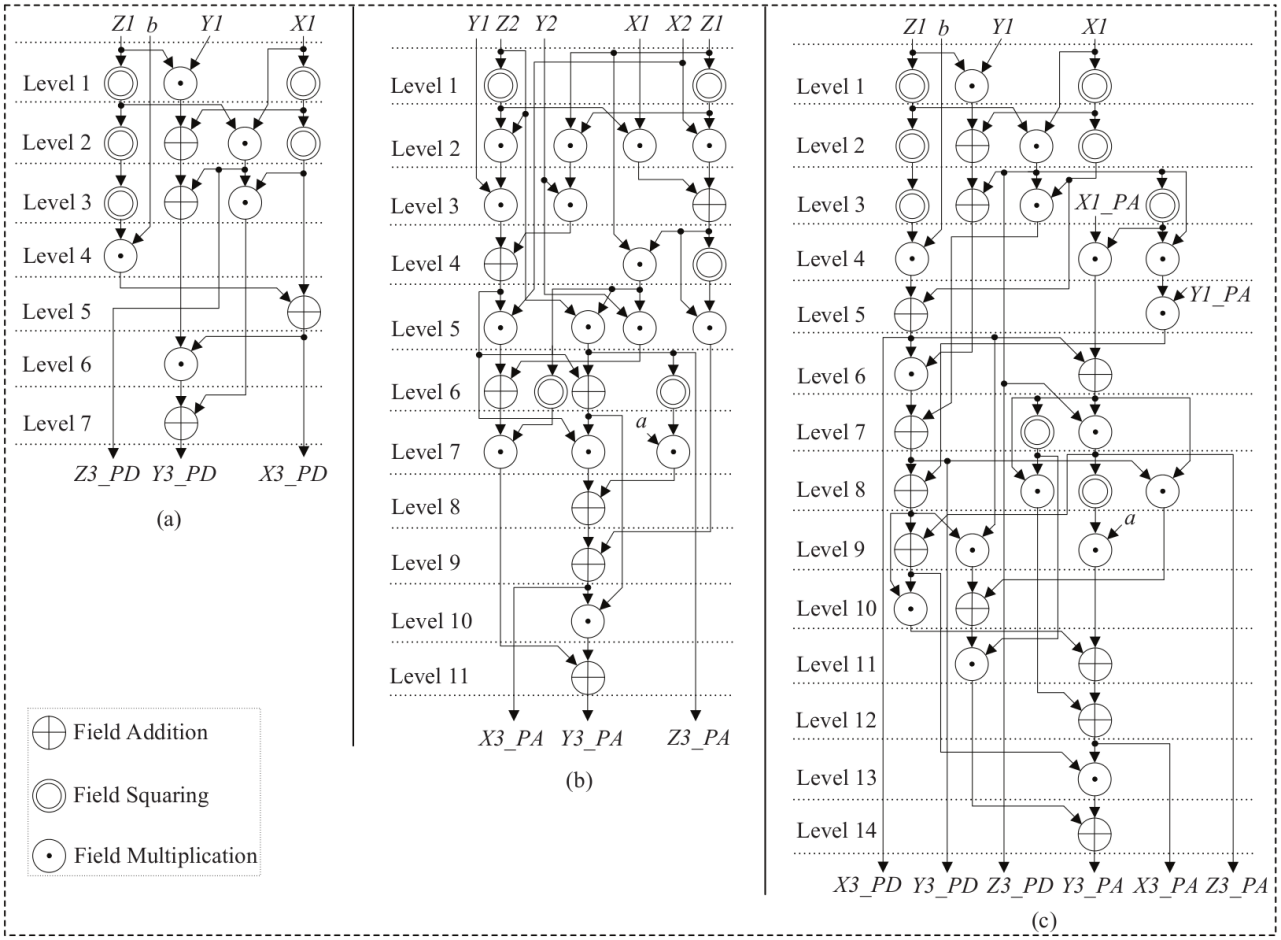
Proposed data-flow architecture for parallel computation of elliptic curve: (a) PD, (b) PA, and (c) PDPA.

A combined PDPA architecture is designed and implemented which performs the PD and PA operations concurrently, as demonstrated in Fig 3C. For this reason, the power consumption pattern for the PDPA hardware will be symmetric in nature. As shown in Fig 2, an ECPM hardware is developed using this combined PDPA architecture. A uniform power consumption profile may be measured throughout the point multiplication computation. From the analysis, we can say that any ‘key’ information is difficult to observe from this hardware. Besides, the double-and-add algorithm is secure against timing and simple power analysis (SPA) attacks [42].

## 4 Proposed group operations

A separate PD and PA architecture as well as a combined PDPA architecture have been designed in Jacobian projective coordinates for point multiplication. To decrease the latency of the group operations in Fig 3, different techniques have been used such as balancing the architecture, parallelization in operations, and pre-computations. In this work, we have utilized Koblitz curve K-163 for implementing group operations. Also, our proposed group operations are supports for a random curve. For doing this, the coefficients *a*, *b* ∈ $F{}_{2}{}^{m}$ defined by NIST [7] have been changed. Fig 3 depicts the proposed architecture of group operations in projective coordinates, corresponding to Eqs (5) and (6). From Fig 3A, the cost of PD is 4A + 5M + 5S, where A, M, and S are the costs of field addition, multiplication, and squaring, respectively. Field addition is the simplest operation in the binary field GF(2^*m*^), being simply a bit-wise exclusive-or (xor (⊕)). Field multiplication is one of the most complex operations in GF(2^*m*^). However, we have proposed an efficient architecture for field multiplication. A field squarer is similar to a field multiplier. As can be seen from Fig 3A, only 7 levels are required to implement the PD operation, and it is fully parallel. The hardware architecture for PA corresponding to Eq (6) is shown in Fig 3B. This architecture is also fully parallel, and the cost of this architecture is 7A + 15M + 5S. Fig 3B demonstrates that 11 levels are required for computing PA. Fig 3C illustrates the combined architecture for a group operation in Jacobian projective coordinates named PDPA. There are 18 levels (7 for PD and 11 for PA) required for group operations using separate architectures, whereas the combined architecture needs only 14 levels. Using this parallel combined architecture, the number of levels in the data path is reduced, which means that the number of logic stages can be minimized, and the overall performance is improved.

## 5 Proposed field multiplication for $F{}_{2}{}^{m}$

This section presents a field multiplication algorithm and a corresponding hardware architecture using a polynomial basis. It is the most crucial operation in implementing point multiplication, because the overall latency of ECPM in projective coordinates mostly depends on the field multiplication. The irreducible polynomials *f*(*x*) = *x*^163^+*x*^7^+*x*^6^+*x*^3^+1 and *f*(*x*) = *x*^233^+*x*^74^+1 have been used for the field GF(2^*m*^) (163-bit and 233-bit ECC). Field multiplication computes the product of two polynomials then applies modular reduction, as shown in Eq (7):

$$\begin{array}{c} Z(x)=U(x).V(x)\;\text{mod}\;f(x) \end{array}$$ (7)

**Fig 4:**
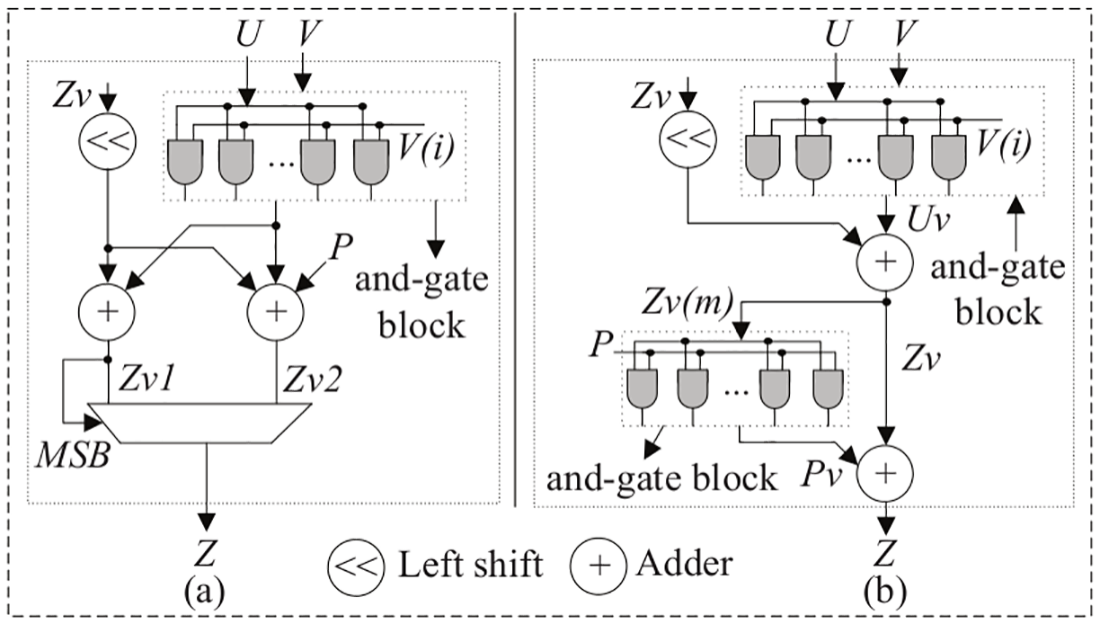
Proposed parallel field multiplication architecture in GF(2^*m*^).

Algorithm 2 presents field multiplication over binary field $F{}_{2}{}^{m}$. The proposed parallel architecture corresponding to Algorithm 2 is shown in Fig 4. As can be seen from Fig 4A, two field additions are performed at the same time. However, this method requires one multiplexer module, is a more expensive operation than the and-gate block (*P*_*v*_). On the other hand, Fig 4B (Algorithm 2) needs only two field additions, one left-shift operation, and two and-gate blocks. Multiplication by *x* can easily be computed by the binary left-shift operation. The and-gate operation is also straightforward as well as time efficient both on FPGA and ASIC. From Algorithm 2, we check whether the result is an element of GF(2^*m*^) with degree <*m*. Only when the multiplication result *Z*_*v*_ has degree *m* or higher is a modular reduction step necessary. This condition is checked by *Z*_*v*_(*m*). When the particular bit of *Z*_*v*_(*m*) is zero, then *P*_*v*_ from the and-gate block generates zero results. Otherwise, *P*_*v*_ generates some result which depends on the modulus *f*(*x*) (*P* = *f*(*x*)). The proposed polynomial-basis multiplication algorithm is better for ASIC-based implementation due to the efficient and-gate block. This architecture is performed fully in parallel. A parallel group operation has been designed using this efficient field multiplication.

**Algorithm 2:** Field multiplication in GF(2^*m*^)

**Input:** *U*(*x*), *V*(*x*) ∈ GF(2^*m*^), an irreducible polynomial *f*(*x*) of degree *m*

**Output:** *Z*(*x*) = *U*(*x*). *V*(*x*) mod *f*(*x*)

1. *Z*_*v*_ = 0; *P* = *f*(*x*);

2. **for** j = *m* − 1 to 0 **do**

2.1  *U*_*v*_ = ‘0’ & *U*(*x*); *Z*_*v*_ = *Z*_*v*_.*x* (left-shift operation);

2.2  **for** i = 0 to *m* − 1 **do** *U*_*v*_(*i*) = *U*_*v*_(*i*) and *V*(*j*); **end for**

2.3  *Z*_*v*_ = *Z*_*v*_ xor *U*_*v*_;

2.4  **for** l = 0 to *m* **do** *P*_*v*_(*l*) = *P*(*l*) and *Z*_*v*_(*m*); **end for**

2.5  *Z*_*v*_ = *Z*_*v*_ xor *P*_*v*_;

3.  **end for**

4. **Return** *Z*(*x*)

## 6 Comparisons and performance analysis

In this section, a performance comparison of various hardware implementations of point multiplication is discussed. The proposed point multiplication has been implemented using synthesizable VHDL code, and synthesized, placed and routed using Xilinx ISE 14.7 with an optimized goal of ‘speed’. It was simulated using both ModelSim PE and ISim. The target FPGA selected is the Xilinx Virtex-7 (XC7VX485T-2FFG1761). We have also implemented our design on a Xilinx Virtex-6 FPGA. In addition, we have synthesized our design using Synopsys Design Compiler with the 65-nm United Microelectronics (UMC) standard logic-cell library. The synthesis results provide better performance in terms of speed and energy than other similar designs in the literature.

**Fig 5:**
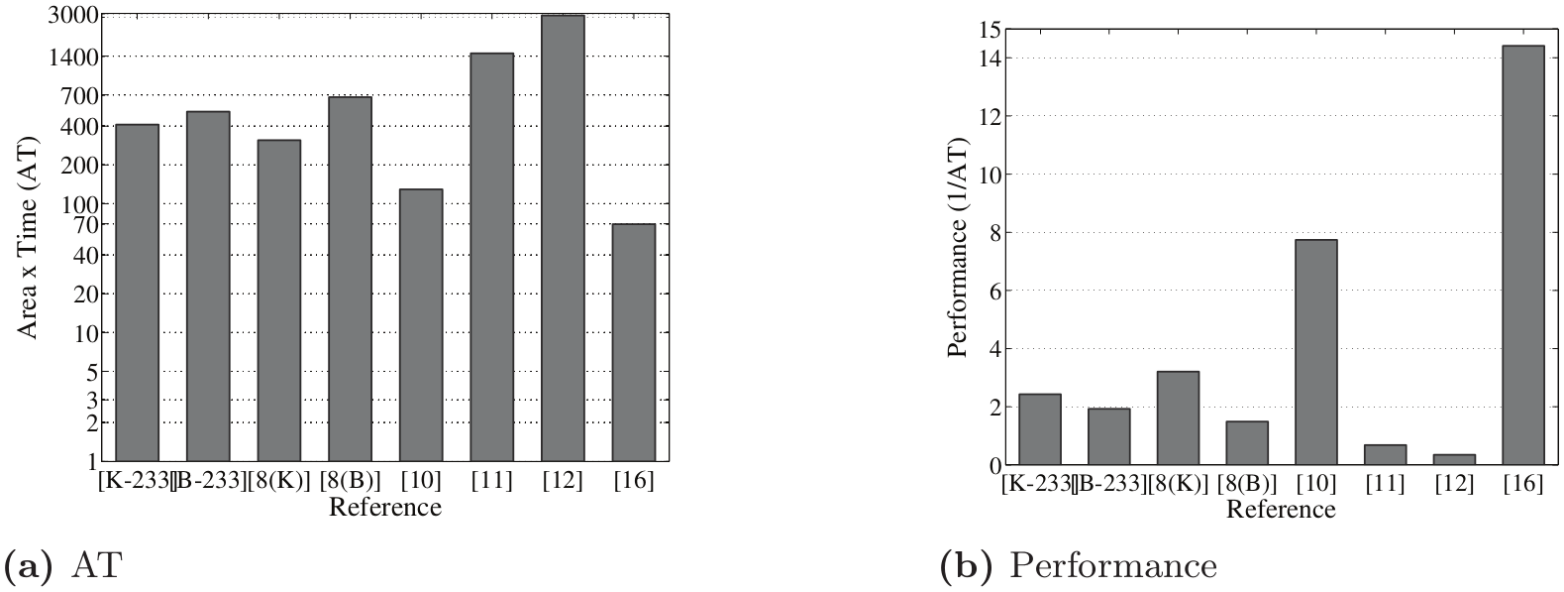
Comparison ((a) AT and (b) performance) of point multiplication over GF(2^233^).

In the literature, most of the point multiplications were implemented over GF(2^163^), but it is of no practical interest to test the algorithm for GF(2^163^), since this curve is no longer approved by NIST to generate digital signatures. For a fair comparison, we have implemented 233-bit as well as 163-bit ECPM for both random and Koblitz curves. Table 1 depicts the performance and a comparison of FPGA implementations of point multiplication over GF(2^233^). The AT value and performance of this design is comparable with other designs in the literature as shown in Fig 5. As can be seen from Table 1 and Fig 5, the point multiplication for a 233-bit random curves takes a little bit more delay and area than with the Koblitz curve. The combined group operation (PDPA) is used to implement ECPM instead of separate PD and PA operations, because the proposed combined PDPA provides better performance than separate group operations. In addition, 163-bit point multiplication is also implemented using both combined and separate group operations for fair comparison.

**Table 1:** Performance analysis of point multiplication on FPGA over GF(2^233^).

Work	Platform	Field Length	Reported Area (slices)	Cycles	Time (*μ*s/ECPM) @f (MHz)	Area × Time (AT)(Kilo-slices × *μ*s)	Efficiency (1/AT)	Throughput rate (Mbps)
**This work**^1^	Virtex-7	K-233	134.68K	233	3.05@76.50	410.78	2.43	76.39
		B-233	145.42K	233	3.56@65.48	517.70	1.93	65.45
[8] Loi (2014)	Virtex-5	K-233	7.43K	6792	41.91@162.07	311.39	3.21	5.56
		B-233	7.98K	12955	84.19@154.35	671.83	1.49	2.77
[10] Sutter (2013)	Virtex-5	233	6.49K	3825	19.89@192.30	129.10	7.74	11.71
[11] Loi (2013)	Virtex-4	233	2.43K	93825	604@155.38	1467.72	0.68	0.39
[12] Loi (2013)	Virtex-4	233	2.65K	155785	1093@142.53	2894.26	0.35	0.21
[16] Rebeiro (2012)	Virtex-5	233	5.64K	1919	12.3@156.00	69.42	14.41	18.94

^1^ Using Virtex-7 (XC7VX980T-2FFG1930)

The proposed point multiplication over GF(2^233^) is synthesized using a Xilinx Virtex-7 (XC7V980T-2FFG1930) FPGA; results are demonstrated in Table 1. As we can see in Table 1, the latency of 233-bit point multiplication is almost 3 *μ*s for a Koblitz curve and 3.56 *μ*s for a random curve with the maximum frequency of 76.50 and 65.48 MHz, respectively. Moreover, the proposed design takes very few clock cycles to implement, which is much better than other comparable work in the literature, but it takes more than 100K slices without using any DSP slices. From the results, we can say that the design provides high speed, but it takes a huge area to implement. However, we have a trade-off between speed and area. Note that the proposed parallel architecture is not suitable for lower versions of the FPGA due to resource (e.g. slices) limitations. On the other hand, our proposed point multiplication over GF(2^233^) provides a higher throughput rate than other related work. As one can see in Fig 5, the AT and performance of our design is similar to [8], but better than [11] and [12]. The point multiplication proposed in [10] and [16] provides a little bit better performance than our proposed design. However, our proposed design is almost six times as fast as [10] and almost four times as fast as [16], making it suitable for cryptographic applications that a require high throughput rate.

Table 2 shows a performance comparison of point multiplication over the last few years in FPGA technology as compared with our proposed parallel design over GF(2^163^). In the available literature, most point multiplication architectures were implemented using separate PD and PA (group operations) modules. We have proposed a novel ECPM hardware in Jacobian coordinates using PDPA (combined group operations). Our design takes *m* clock cycles for *m*-bit point multiplication, which is much less than other designs. As can be seen from Table 2, the point multiplication for a 163-bit random curve takes the same time as with the Koblitz curve, but it takes a little bit more area than the random curve. The proposed ECPM using PDPA architecture takes less time than all other similar designs on FPGA. We have achieved a point multiplication in 0.31 *μ*s and 0.33 *μ*s in a Virtex-7 and Virtex-6 FPGA, respectively. In addition, an ECPM is designed and implemented using separate group operations which take 3.51 *μ*s for a Virtex-7 FPGA and 3.82 *μ*s for a Virtex-6 FPGA. As can be seen from Table 2, ECPM using a combined PDPA architecture performs 13 times as fast as separate modules in either a Virtex-7 or a Virtex-6 FPGA device.

**Table 2:** Performance comparison of point multiplication on FPGA over GF(2^163^).

Work		Platform	Reported Area (slices)	Cycles	Time (*μ*s/ECPM) @f(MHz)	Area × Time (AT)(Kilo-slices × *μ*s)	Performance (1/AT)	Throughput rate (Mbps)
**[a]**	**This work**^1^	Virtex-7*	57.39K	163	0.31@528.58	17.70	56.50	528.58
**[b]**			72.43K	163	0.31@528.58	22.45	44.54	528.58
**[c]**		Virtex-6*	71.96K	163	0.33@500.16	23.45	42.64	500.16
**[d]**			73.47K	163	0.33@500.16	24.25	41.24	500.16
**[e]**	**This work**^2^	Virtex-7*	64.80K	163	3.51@46.41	227.59	4.39	46.41
**[f]**		Virtex-6*	77.54K	163	3.82@42.71	296.19	3.40	42.71
[8]	Loi (2014)	Virtex-5	7.43K^8*a*^	4745	29.28@162.07	217.46	4.60	5.57
			7.98K^8*b*^	9130	59.15@154.35	471.90	2.12	2.76
[9]	Liu (2014)	Virtex-4	10.42K	1091	9.00@121.00	93.75	10.70	18.11
[10]	Sutter (2013)	Virtex-5	6.15K	1371	5.50@249.27	33.83	29.56	29.64
[11]	Loi (2013)	Virtex-4	2.43K	42419	273@155.38	663.39	1.51	0.60
[12]	Loi (2013)	Virtex-4	2.65K	68842	483@142.53	1279.95	0.78	0.34
[13]	Reza (2013)	Stratix II	23.08K ALMs	1721	9.15@188.71	-	-	17.81
[14]	Mahdizadeh (2013)	Virtex-4	17.93K	2751	9.60@250.00	172.12	5.81	16.97
[15]	Reza (2012)	Virtex-5	5.79K	3880	14.50@267.10	83.93	11.92	11.24
[16]	Rebeiro (2012)	Virtex-5	3.5K	1436	8.60@167.00	29.64	33.74	18.95
[17]	Roy (2012)	Virtex-5	12.43K	552	12.10@45.62	150.40	6.65	13.47
[18]	Zhang (2010)	Virtex-4	20.81K	1428	7.70@185.00	160.21	6.24	21.17
[19]	Dimitrios (2009)	Virtex-2	12245 LUTs	9107	39.00@233.50	-	-	4.18
[20]	Chelton (2008)	Virtex-4	16.21K	3010	19.55@153.90	316.89	3.16	8.33
[21]	Ansari (2008)	Virtex-2	3.42K	4075	41.00@100.00	140.06	7.14	3.98
[22]	Kim (2008)	Virtex-4	24.36K	1446	10.00@143.00	243.63	4.11	16.30
[23]	Antao (2008)	Virtex-4	10.49K	14256	144.00@99.00	1510.27	0.66	1.13

[a], [c], [e] for Koblitz curve and [b], [d], [f] for random curve.

* using Virtex-7 (XC7VX485T-2FFG1761) and Virtex-6 (XC6VLX760-2ff1760).

^1^ ECPM using PDPA.

^2^ ECPM using separate PD and PA.

In Table 2, the results of [8, 10, 13, 15–17, 19, 21] show FPGA implementations of point multiplication in GF(2^163^). They used trivial group operations (PD and PA) for implementing ECPM. Their proposed designs require fewer slices than our design, but they need more clock cycles, hence more computation time, to complete. Point multiplication schemes over the binary field GF(2^163^) are presented in [9, 11, 12, 14, 18, 20, 22, 23]. Their proposed point multiplication schemes were implemented in a Virtex-4 FPGA device. Of them, the result provided in [9] shows the best result in terms of performance as shown in Table 2. On the other hand, our proposed point multiplication using the PDPA architecture delivers 5 times the performance (1/AT) of those in [9]. Besides, the throughput rate of our design is far better than the others.

**Fig 6:**
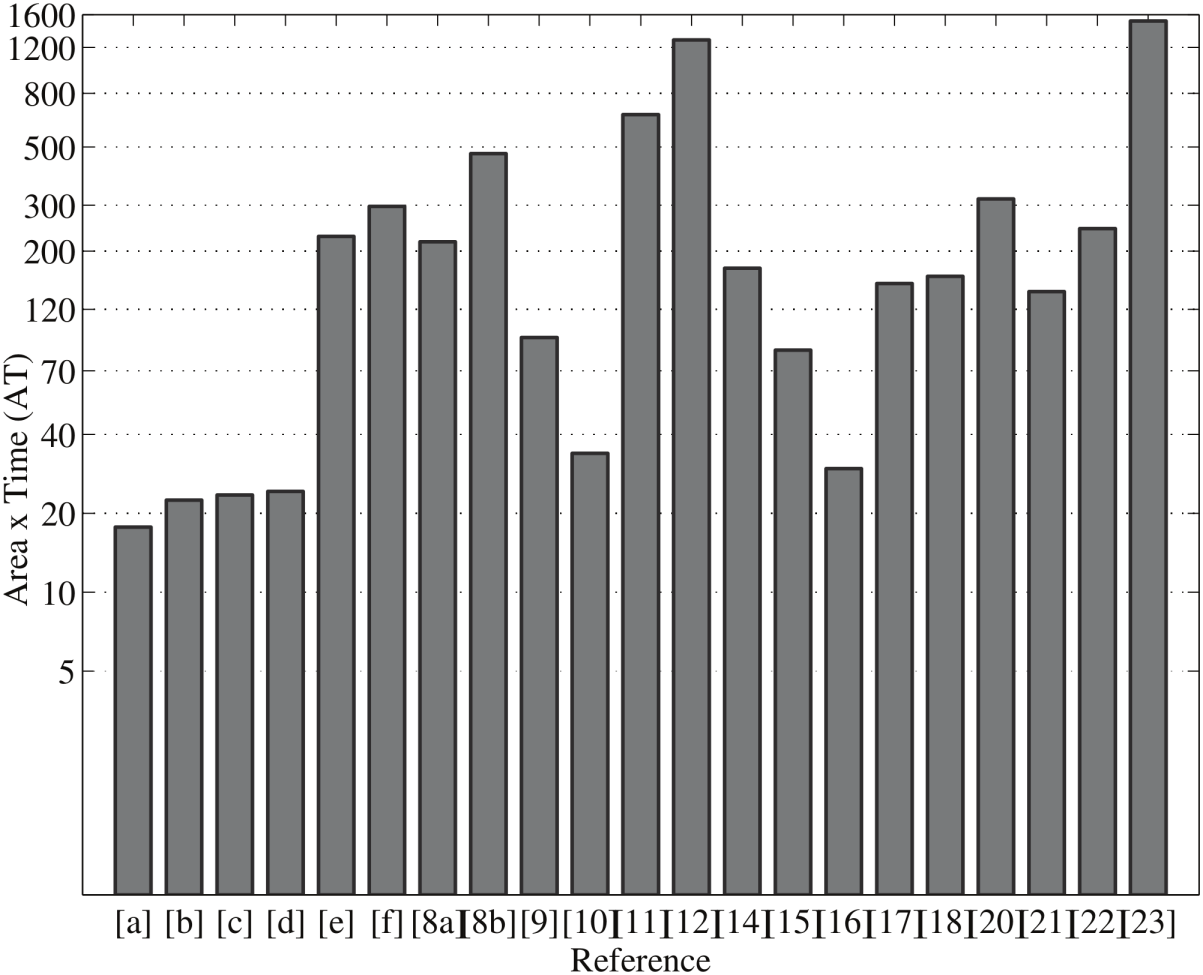
AT comparison of point multiplication ([a], [b], [c], [d], [e], and [f] represent our work) over GF(2^163^).

**Fig 7:**
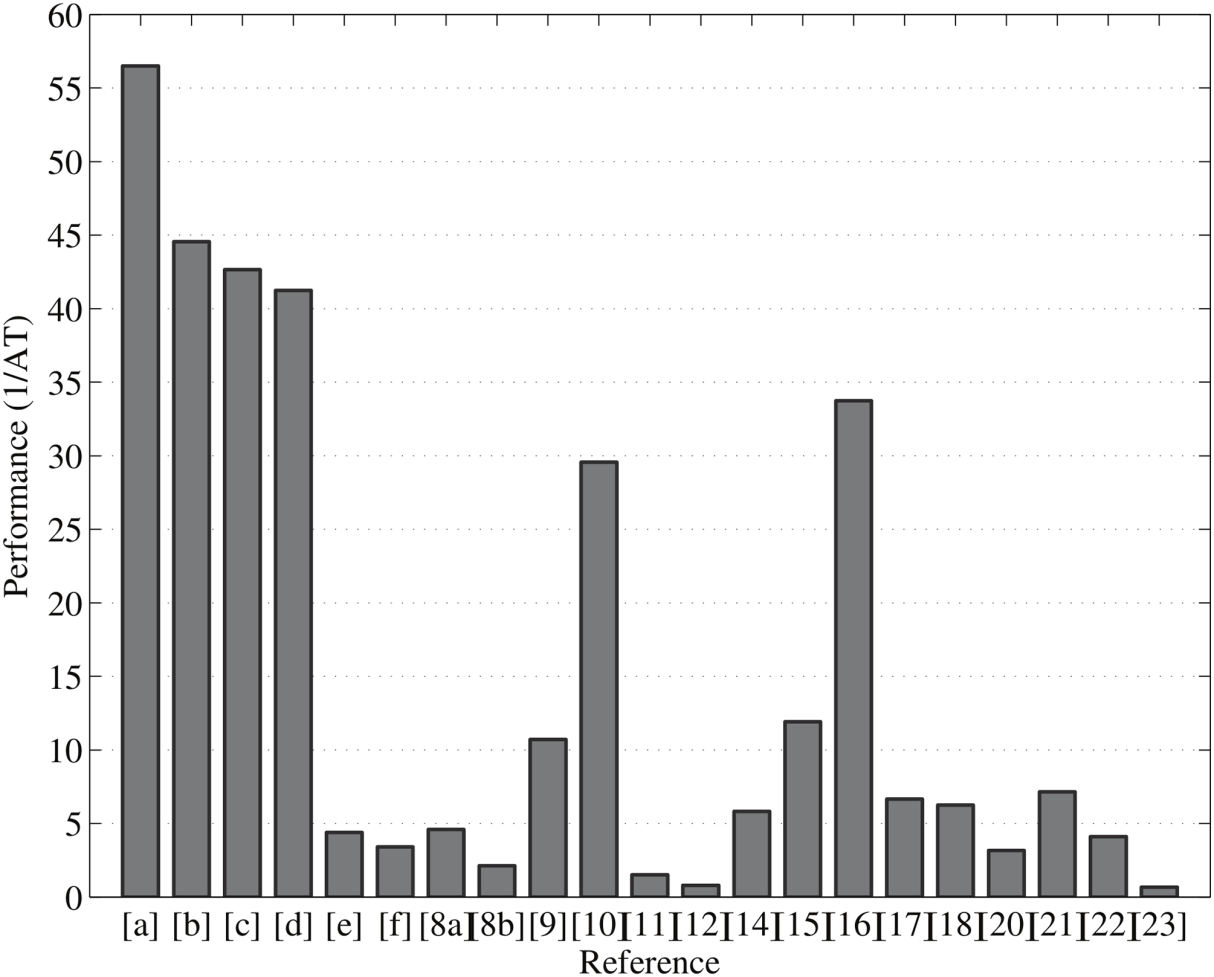
Performance comparison of point multiplication ([a], [b], [c], [d], [e], and [f] represent our work) over GF(2^163^).

The AT and performance or efficiency metric are the best indicators to say which design is better. The performance or efficiency of point multiplication is defined in Eq (8), in ECPM operations per sec per slice. The area-time (AT) comparison of point multiplication over GF(2^163^) with similar designs is shown in Fig 6. As can be seen from the graph, our design provides a lower AT value than all other designs. Fig 7 compares the performance of point multiplication with similar work in Table 2. Note that [a], [b], [c], [d], [e], and [f] of Figs 6 and 7 represent our implementation results and [8–12, 14–18, 20–23] illustrate reference FPGA implementations over GF(2^163^). The AT and performance metric demonstrates that we have achieved a higher efficiency than most of the similar designs in the available literature. Note that, of all the available designs, in terms of AT value the designs proposed in [10] and [16] perform better. However, we have achieved a 50% better performance than their designs. The point multiplication techniques proposed in the literature need fewer slices but require more computation time than our design. From the comparison of various ECPMs over the binary field GF(2^163^) in Table 2, our novel parallel point multiplication using combined PDPA in Jacobian coordinates is the fastest hardware implementation result reported in the literature to date.

$$\begin{array}{c} \text{Performance}=\text{Efficiency}=\frac{1}{\text{Area}\times \text{Time}}=\frac{1}{\text{AT}} \end{array}$$ (8)

In the state of the art, few implementations are targeted on ASIC, being mostly FPGA implementations. Both technologies (FPGA and ASIC) have been utilized for this paper. Table 3 depicts the ASIC-based performance analysis and comparison of elliptic curve point multiplication over GF(2^233^) and GF(2^163^). The proposed high-speed parallel point-multiplication architecture is synthesized using 65-nm CMOS technology, a more advanced version of ASIC technology than 0.13 *μ*m, 0.18 *μ*m, and 0.35 *μ*m CMOS technology. Besides, we have optimized our design for Koblitz (K-233 and K-163) curves as well as random (B-233 and B-163) curves to compare with those of other similar studies. We find that the NIST random curve takes more area than the NIST Koblitz curve for ASIC-based point multiplication design. The proposed design needs only 0.81 *μ*s for 233-bit ECPM and 0.46 *μ*s for 163-bit ECPM, either Koblitz or random curve, to complete. The point multiplication over GF(2^233^) takes 7.56 mm^2^ (for K-233) with 3635K gate count and 8.42 mm^2^ (for B-233) area with 4048K gate count in UMC 65-nm technology. Similarly, the results for 163-bit ECC (both in Koblitz and random curves) are depicted in Table 3, which takes 3.43 mm^2^ (for K-163) with 1649K gate count and 3.47 mm^2^ (for B-163) area with 1668K gate count. The implemented design is also energy-efficient. The energy is computed from the power consumption and point multiplication time. The energy consumption per point multiplication over GF(2^163^) and GF(2^233^) is between 0.22 and 0.98 *μ*J which is far less than most recent designs. For example, the power consumption of B-163 point multiplication is 487 mW, of which 178 mW is for cell internal power, 307 mW is for net switching power, and the rest is leakage power. Similarly, the power consumption for 233-bit ECC is simulated from the Synopsys design compiler.

**Table 3:** Performance analysis of ASIC-based point multiplication over binary fields.

Work		Platform	Field	Reported Area^1^	KCycles	Time (*μ* s)@f(MHz)	AT^2^	Energy (*μ*J)	ATE^3^	TR^4^ (Mbps)
**[g]**	**This work**	65-nm	K-233	7.56/3635	0.233	0.81@289	6.12/2.94	0.88	0.0054/2.59	288
**[h]**			B-233	8.42/4048	0.233	0.81@289	6.82/3.28	0.98	0.0067/3.21	288
**[i]**			K-163	3.43/1649	0.163	0.46@353	1.58/0.76	0.22	0.00035/0.17	354
**[j]**			B-163	3.47/1668	0.163	0.46@353	1.60/0.77	0.24	0.00038/0.18	354
[33]	Liu (2016)	55-nm	233	0.35/189	372.9	1180@316	413/223.0	40.4	16.7/9009	0.20
			163	0.35/189	189.6	600@316	210/113.4	20.6	4.3/2336	0.27
[26]	Lee (2014)	90-nm	B-233^26*a*^	1.12/313	124.3	520@238	582/162.8	34.0	19.8/5534	0.45
			B-163^26*b*^	0.24/65	62.5	220@277	53/14.3	8.0	0.43/117	0.74
[10]	Sutter (2013)	180-nm	B-163	-/138	1.70	9.5@179	-/1.3	-	-/-	17
[27]	Chen (2010)	130-nm	163	2.34/332	182.6	440@415	1030/146.1	61.0	63/8911	0.37
[18]	Zhang (2010)	180-nm	163	-/218	1.43	5.4@263	-/1.2	-	-/-	30
[28]	Hong (2009)	180-nm	B-163	2.10/69	228.1	1890@181	3969/130.4	257.0	1020/33515	0.09
[29]	Lee (2008)	130-nm	163	-/12.5	275.8	244000 @0.001	-/3050	9.0	-/27450	$\frac{0.65}{1000}$
[30]	Sakiyama (2007)	130-nm	163	-/393	22.0	70@292	-/27.5	-	-/-	2.33
[31]	Kumar (2006)	350-nm	163	-/16	376.8	27900@14	-/446.4	-	-/-	$\frac{5.87}{1000}$
[32]	Park (2005)	350-nm	163	-/46	134.0	3050@44	-/140.3	-	-/-	$\frac{53.46}{1000}$

^1^ Area (mm^2^/Kilo-Gates).

^2^ Area × Time = AT (mm^2^×*μ*s)/(KGs×ms).

^3^ ATE = Area × Time × Energy (mm^2^×*μ*s×mJ)/(KGs×ms×*μ*J).

^4^ TR = Throughput rate.

**Fig 8:**
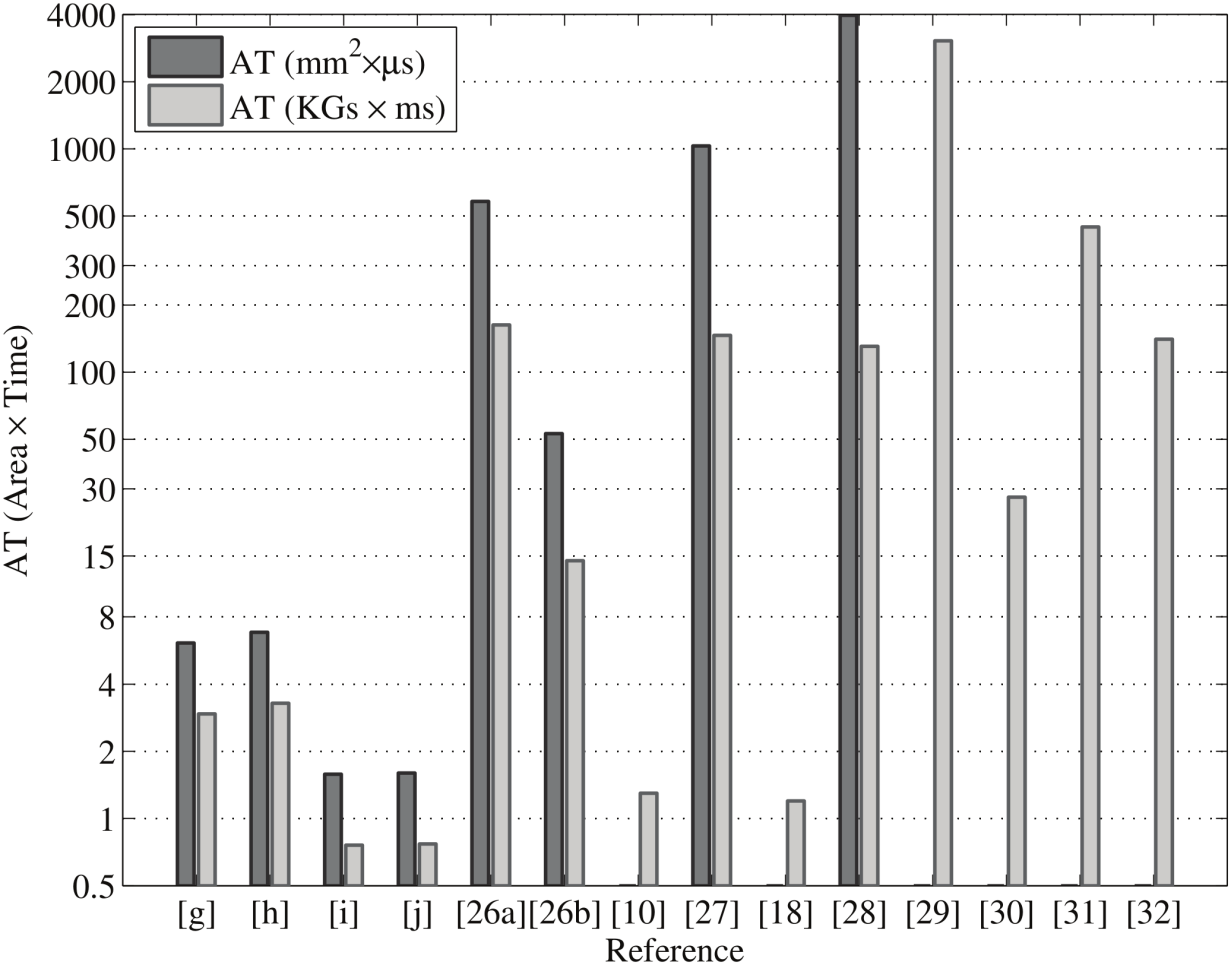
AT comparison of point multiplication ([g], [h], [i], and [j] represent our work) with references.

**Fig 9:**
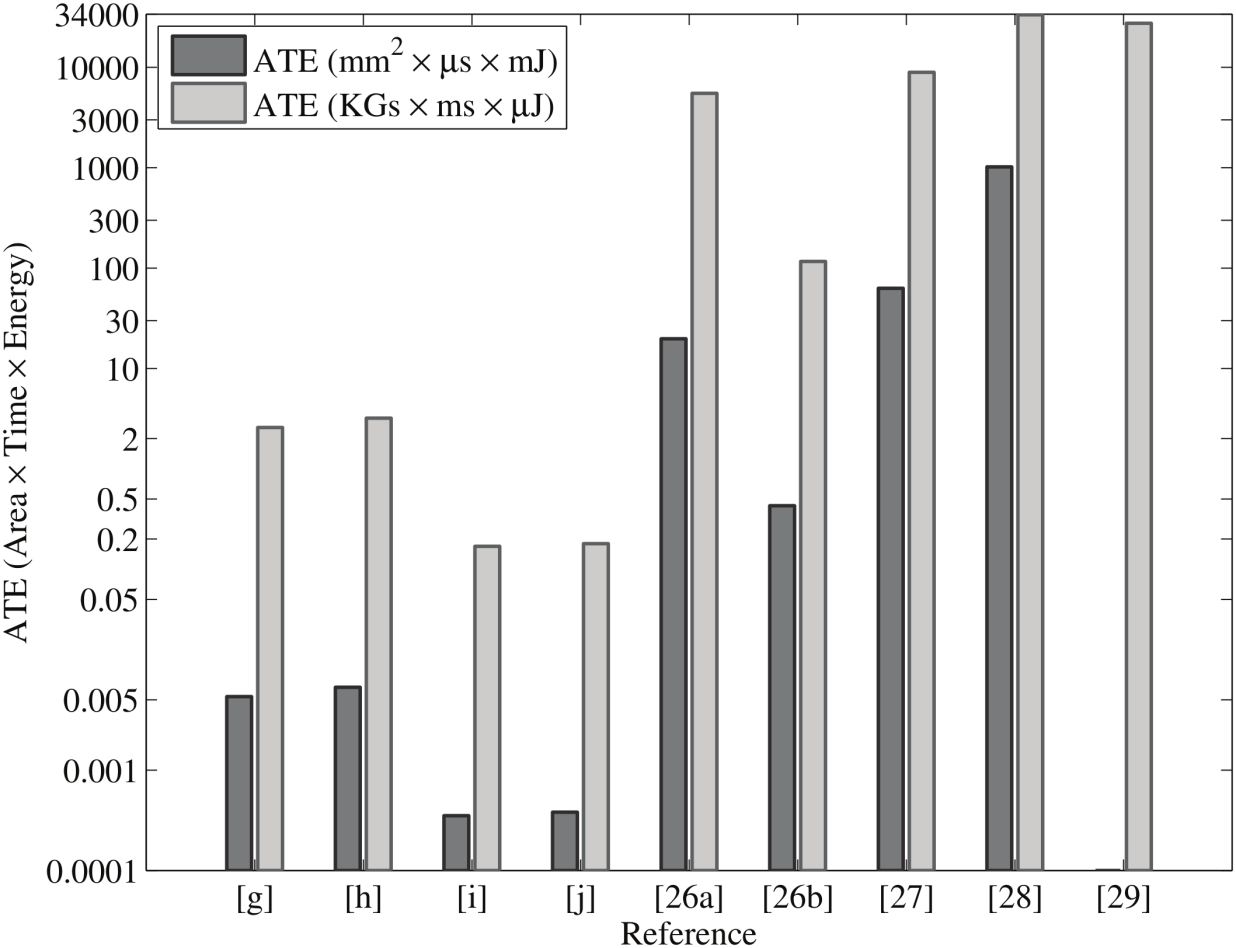
ATE comparison of point multiplication ([g], [h], [i], and [j] represent our work) with related designs.

Table 3 shows our synthesis results and the most recent work using ASIC implementation. As can be seen from Table 3, our design is faster as well as more energy-efficient than all other significant designs found in the available literature. However, our design is not area-efficient due to the parallel architecture. This is a kind of design trade-off between area, time, and energy. For a fair comparison, we have calculated area × time (AT) and area × time × energy (ATE) products. Figs 8 and 9 show the area-delay and area-delay-energy products for our proposed design and related circuits presented in Table 3. It is crystal clear that we present more outstanding results than other designs in terms of AT and ATE. [40]

## 7 Conclusion

A novel parallel architecture for point multiplication, the core operation of an ECC processor, has been proposed and implemented over GF(2^233^) and GF(2^163^). It is implemented by the double-and-add method using Jacobian projective coordinates. To provide efficient point multiplication, a novel combined group operation (PDPA) is designed which performs the PD and PA operations in parallel, aimed at reducing the number of levels and logic stages needed with separate PD and PA operations. A parallel field multiplication using a polynomial basis is developed for group operations, hence point multiplication.

Using parallel architecture, the proposed 233-bit ECPM takes only 3.05 *μ*s (for K-233) and 3.56 *μ*s (for B-233) in a Xilinx Virtex-7 FPGA. In addition, we have achieved a point multiplication over GF(2^163^) in 0.31 *μ*s and 0.33 *μ*s in a Virtex-7 and Virtex-6 FPGA, respectively. Regarding ASIC synthesis results, the proposed design takes a similar delay to FPGA implementation. The core area of the proposed design is a little bit higher than similar designs, namely 7.56 mm^2^ (for K-233) and is 3.43 mm^2^ (for K-163). The energy consumption per point multiplication is only 0.88 and 0.22 *μ*J for K-233 and K-163, respectively.

We can say that the proposed parallel architecture for point multiplication is energy-efficient. However, in both technologies (FPGA and ASIC), we require more area for implementation. According to our best knowledge, the proposed parallel point multiplication architecture is the fastest hardware implementation result to date. Based on the overall performance and comparisons, a 50% improvement is achieved over recent FPGA implementations and significant improvement is gained over the most recent ASIC-based designs. We conclude that our proposed design provides better performance which can be used for modern high-speed cryptographic applications.

## Supporting information

S1 Supporting Information S1_Supporting_Information.zip. (ZIP)
