# Supplementary material for: Parallel point-multiplication architecture using combined group operations for high-speed cryptographic applications
Source: PLoS One. 2017 May 1;12(5):e0176214. doi: 10.1371/journal.pone.0176214 (PMC5411040; doi:10.1371/journal.pone.0176214)
Supplement: S1 Supporting Information — (ZIP) [file pone.0176214.s001.zip › S1 Supporting Information/S1 File10 Table2_[c].pdf]

```
Release 14.7 Map P.20131013 (nt64)
Xilinx Mapping Report File for Design 'ECC_TOP_K_163'

Design Information
------------------
Command Line   : map -intstyle ise -p xc6vlx760-ff1760-2 -w -logic_opt off -ol
high -t 1 -xt 0 -register_duplication off -r 4 -global_opt off -mt off -ir off
-pr off -lc off -power off -o ECC_TOP_K_163_map.ncd ECC_TOP_K_163.ngd
ECC_TOP_K_163.pcf
Target Device  : xc6vlx760
Target Package : ff1760
Target Speed   : -2
Mapper Version : virtex6 -- $Revision: 1.55 $
Mapped Date    : Tue May 24 21:52:31 2016

Design Summary
--------------
Number of errors:      0
Number of warnings:    0
Slice Logic Utilization:
  Number of Slice Registers:                 1,476 out of 948,480    1%
    Number used as Flip Flops:                 987
    Number used as Latches:                    489
    Number used as Latch-thrus:                  0
    Number used as AND/OR logics:                0
  Number of Slice LUTs:                    210,390 out of 474,240   44%
    Number used as logic:                  210,387 out of 474,240   44%
      Number using O6 output only:         208,039
      Number using O5 output only:               0
      Number using O5 and O6:                2,348
      Number used as ROM:                        0
    Number used as Memory:                       0 out of 132,480    0%
    Number used exclusively as route-thrus:      3
      Number with same-slice register load:      3
      Number with same-slice carry load:         0
      Number with other load:                    0

Slice Logic Distribution:
  Number of occupied Slices:                71,964 out of 118,560   60%
  Number of LUT Flip Flop pairs used:      210,650
    Number with an unused Flip Flop:       209,178 out of 210,650   99%
    Number with an unused LUT:                 260 out of 210,650    1%
    Number of fully used LUT-FF pairs:       1,212 out of 210,650    1%
    Number of unique control sets:               3
    Number of slice register sites lost
      to control set restrictions:              20 out of 948,480    1%

  A LUT Flip Flop pair for this architecture represents one LUT paired with
  one Flip Flop within a slice.  A control set is a unique combination of
  clock, reset, set, and enable signals for a registered element.
  The Slice Logic Distribution report is not meaningful if the design is
  over-mapped for a non-slice resource or if Placement fails.
  OVERMAPPING of BRAM resources should be ignored if the design is
  over-mapped for a non-BRAM resource or if placement fails.

IO Utilization:
  Number of bonded IOBs:                       492 out of   1,200   41%

Specific Feature Utilization:
  Number of RAMB36E1/FIFO36E1s:                  0 out of     720    0%
  Number of RAMB18E1/FIFO18E1s:                  0 out of   1,440    0%
  Number of BUFG/BUFGCTRLs:                      2 out of      32    6%
    Number used as BUFGs:                        2
    Number used as BUFGCTRLs:                    0
  Number of ILOGICE1/ISERDESE1s:                 0 out of   1,440    0%
  Number of OLOGICE1/OSERDESE1s:                 0 out of   1,440    0%
  Number of BSCANs:                              0 out of       4    0%
  Number of BUFHCEs:                             0 out of     216    0%
  Number of BUFIODQSs:                           0 out of     144    0%
  Number of BUFRs:                               0 out of      72    0%
  Number of CAPTUREs:                            0 out of       1    0%
```

```
  Number of DSP48E1s:                             0 out of     864    0%
  Number of EFUSE_USRs:                           0 out of       1    0%
  Number of FRAME_ECCs:                           0 out of       1    0%
  Number of ICAPs:                                0 out of       2    0%
  Number of IDELAYCTRLs:                          0 out of      36    0%
  Number of IODELAYE1s:                           0 out of   1,440    0%
  Number of MMCM_ADVs:                            0 out of      18    0%
  Number of STARTUPs:                             0 out of       1    0%
  Number of SYSMONs:                              0 out of       1    0%

Average Fanout of Non-Clock Nets:                 5.56

Peak Memory Usage:  3375 MB
Total REAL time to MAP completion:  32 mins 10 secs
Total CPU time to MAP completion:   32 mins 3 secs

Table of Contents
-----------------
Section 1 - Errors
Section 2 - Warnings
Section 3 - Informational
Section 4 - Removed Logic Summary
Section 5 - Removed Logic
Section 6 - IOB Properties
Section 7 - RPMs
Section 8 - Guide Report
Section 9 - Area Group and Partition Summary
Section 10 - Timing Report
Section 11 - Configuration String Information
Section 12 - Control Set Information
Section 13 - Utilization by Hierarchy

Section 1 - Errors
------------------

Section 2 - Warnings
--------------------

Section 3 - Informational
-------------------------
INFO:LIT:243 - Logical network start has no load.
INFO:MapLib:562 - No environment variables are currently set.
INFO:LIT:244 - All of the single ended outputs in this design are using slew
   rate limited output drivers. The delay on speed critical single ended outputs
   can be dramatically reduced by designating them as fast outputs.
INFO:Pack:1716 - Initializing temperature to 85.000 Celsius. (default - Range:
   0.000 to 85.000 Celsius)
INFO:Pack:1720 - Initializing voltage to 0.950 Volts. (default - Range: 0.950 to
   1.050 Volts)
INFO:Map:215 - The Interim Design Summary has been generated in the MAP Report
   (.mrp).
INFO:Pack:1650 - Map created a placed design.

Section 4 - Removed Logic Summary
---------------------------------
  69 block(s) removed
  47 block(s) optimized away
  69 signal(s) removed

Section 5 - Removed Logic
-------------------------

The trimmed logic report below shows the logic removed from your design due to
sourceless or loadless signals, and VCC or ground connections.  If the removal
of a signal or symbol results in the subsequent removal of an additional signal
or symbol, the message explaining that second removal will be indented.  This
indentation will be repeated as a chain of related logic is removed.

To quickly locate the original cause for the removal of a chain of logic, look
above the place where that logic is listed in the trimming report, then locate
the lines that are least indented (begin at the leftmost edge).
```

The signal "uut_PD_PA_Jac_163/SQ_SQ2_PA/Mxor_SQ_BF.Cv_29_xo<0>" is sourceless and has been removed.
The signal "uut_PD_PA_Jac_163/SQ_SQ2_PA/Mxor_SQ_BF.Cv_29_xo<0>1" is sourceless and has been removed.
The signal "uut_PD_PA_Jac_163/SQ_SQ2_PA/Mxor_SQ_BF.Cv_29_xo<0>2" is sourceless and has been removed.
The signal "uut_PD_PA_Jac_163/SQ_SQ2_PA/Mxor_SQ_BF.Cv_29_xo<0>3" is sourceless and has been removed.
The signal "uut_PD_PA_Jac_163/SQ_SQ5_PD/Mxor_GND_10_o_GND_10_o_xor_64_OUT_163_xo<0>" is sourceless and has been removed.
The signal "uut_PD_PA_Jac_163/SQ_SQ5_PD/Mxor_GND_10_o_GND_10_o_xor_64_OUT_163_xo<0>1" is sourceless and has been removed.
The signal "uut_PD_PA_Jac_163/SQ_SQ5_PD/Mxor_GND_10_o_GND_10_o_xor_64_OUT_163_xo<0>2" is sourceless and has been removed.
The signal "uut_PD_PA_Jac_163/SQ_SQ5_PD/Mxor_GND_10_o_GND_10_o_xor_64_OUT_163_xo<0>3" is sourceless and has been removed.
The signal "uut_PD_PA_Jac_163/SQ_SQ5_PD/Mxor_SQ_BF.Cv_29_xo<0>" is sourceless and has been removed.
The signal "uut_PD_PA_Jac_163/SQ_SQ5_PD/Mxor_SQ_BF.Cv_29_xo<0>1" is sourceless and has been removed.
The signal "uut_PD_PA_Jac_163/SQ_SQ5_PD/Mxor_SQ_BF.Cv_29_xo<0>2" is sourceless and has been removed.
The signal "uut_PD_PA_Jac_163/SQ_SQ5_PD/Mxor_SQ_BF.Cv_29_xo<0>3" is sourceless and has been removed.
The signal "uut_PD_PA_Jac_163/SQ_SQ5_PD/Mxor_SQ_BF.Cv_46_xo<0>13" is sourceless and has been removed.
The signal "uut_PD_PA_Jac_163/SQ_SQ5_PD/Mxor_GND_10_o_GND_10_o_xor_192_OUT_163_xo<0>10" is sourceless and has been removed.
The signal "uut_PD_PA_Jac_163/SQ_SQ3_PA/Mxor_SQ_BF.Cv_29_xo<0>" is sourceless and has been removed.
The signal "uut_PD_PA_Jac_163/SQ_SQ3_PA/Mxor_SQ_BF.Cv_29_xo<0>1" is sourceless and has been removed.
The signal "uut_PD_PA_Jac_163/SQ_SQ3_PA/Mxor_SQ_BF.Cv_29_xo<0>2" is sourceless and has been removed.
The signal "uut_PD_PA_Jac_163/SQ_SQ3_PA/Mxor_SQ_BF.Cv_29_xo<0>3" is sourceless and has been removed.
The signal "uut_PD_PA_Jac_163/SQ_SQ3_PA/Mxor_SQ_BF.Cv_46_xo<0>13" is sourceless and has been removed.
The signal "uut_PD_PA_Jac_163/SQ_SQ3_PD/Mxor_GND_10_o_GND_10_o_xor_64_OUT_163_xo<0>" is sourceless and has been removed.
The signal "uut_PD_PA_Jac_163/SQ_SQ3_PD/Mxor_GND_10_o_GND_10_o_xor_64_OUT_163_xo<0>1" is sourceless and has been removed.
The signal "uut_PD_PA_Jac_163/SQ_SQ3_PD/Mxor_GND_10_o_GND_10_o_xor_64_OUT_163_xo<0>2" is sourceless and has been removed.
The signal "uut_PD_PA_Jac_163/SQ_SQ3_PD/Mxor_GND_10_o_GND_10_o_xor_64_OUT_163_xo<0>3" is sourceless and has been removed.
The signal "uut_PD_PA_Jac_163/SQ_SQ3_PD/Mxor_SQ_BF.Cv_29_xo<0>" is sourceless and has been removed.
The signal "uut_PD_PA_Jac_163/SQ_SQ3_PD/Mxor_SQ_BF.Cv_29_xo<0>1" is sourceless and has been removed.
The signal "uut_PD_PA_Jac_163/SQ_SQ3_PD/Mxor_SQ_BF.Cv_29_xo<0>2" is sourceless and has been removed.
The signal "uut_PD_PA_Jac_163/SQ_SQ3_PD/Mxor_SQ_BF.Cv_29_xo<0>3" is sourceless and has been removed.
The signal "uut_PD_PA_Jac_163/SQ_SQ3_PD/Mxor_SQ_BF.Cv_46_xo<0>13" is sourceless and has been removed.
The signal "uut_PD_PA_Jac_163/SQ_SQ3_PD/Mxor_GND_10_o_GND_10_o_xor_192_OUT_163_xo<0>10" is sourceless and has been removed.
The signal "uut_PD_PA_Jac_163/SQ_SQ1_PA/Mxor_GND_10_o_GND_10_o_xor_64_OUT_163_xo<0>" is

sourceless and has been removed.
The signal
"uut_PD_PA_Jac_163/SQ_SQ1_PA/Mxor_GND_10_o_GND_10_o_xor_64_OUT_163_xo<0>1" is
sourceless and has been removed.
The signal
"uut_PD_PA_Jac_163/SQ_SQ1_PA/Mxor_GND_10_o_GND_10_o_xor_64_OUT_163_xo<0>2" is
sourceless and has been removed.
The signal
"uut_PD_PA_Jac_163/SQ_SQ1_PA/Mxor_GND_10_o_GND_10_o_xor_64_OUT_163_xo<0>3" is
sourceless and has been removed.
The signal "uut_PD_PA_Jac_163/SQ_SQ1_PA/Mxor_SQ_BF.Cv_29_xo<0>" is sourceless
and has been removed.
The signal "uut_PD_PA_Jac_163/SQ_SQ1_PA/Mxor_SQ_BF.Cv_29_xo<0>1" is sourceless
and has been removed.
The signal "uut_PD_PA_Jac_163/SQ_SQ1_PA/Mxor_SQ_BF.Cv_29_xo<0>2" is sourceless
and has been removed.
The signal "uut_PD_PA_Jac_163/SQ_SQ1_PA/Mxor_SQ_BF.Cv_29_xo<0>3" is sourceless
and has been removed.
The signal "uut_PD_PA_Jac_163/SQ_SQ1_PA/Mxor_SQ_BF.Cv_46_xo<0>13" is sourceless
and has been removed.
The signal
"uut_PD_PA_Jac_163/SQ_SQ1_PA/Mxor_GND_10_o_GND_10_o_xor_192_OUT_163_xo<0>10" is
sourceless and has been removed.
The signal
"uut_PD_PA_Jac_163/SQ_SQ4_PD/Mxor_GND_10_o_GND_10_o_xor_64_OUT_163_xo<0>" is
sourceless and has been removed.
The signal
"uut_PD_PA_Jac_163/SQ_SQ4_PD/Mxor_GND_10_o_GND_10_o_xor_64_OUT_163_xo<0>1" is
sourceless and has been removed.
The signal
"uut_PD_PA_Jac_163/SQ_SQ4_PD/Mxor_GND_10_o_GND_10_o_xor_64_OUT_163_xo<0>2" is
sourceless and has been removed.
The signal
"uut_PD_PA_Jac_163/SQ_SQ4_PD/Mxor_GND_10_o_GND_10_o_xor_64_OUT_163_xo<0>3" is
sourceless and has been removed.
The signal "uut_PD_PA_Jac_163/SQ_SQ4_PD/Mxor_SQ_BF.Cv_29_xo<0>" is sourceless
and has been removed.
The signal "uut_PD_PA_Jac_163/SQ_SQ4_PD/Mxor_SQ_BF.Cv_29_xo<0>1" is sourceless
and has been removed.
The signal "uut_PD_PA_Jac_163/SQ_SQ4_PD/Mxor_SQ_BF.Cv_29_xo<0>2" is sourceless
and has been removed.
The signal "uut_PD_PA_Jac_163/SQ_SQ4_PD/Mxor_SQ_BF.Cv_29_xo<0>3" is sourceless
and has been removed.
The signal "uut_PD_PA_Jac_163/SQ_SQ4_PD/Mxor_SQ_BF.Cv_46_xo<0>13" is sourceless
and has been removed.
The signal
"uut_PD_PA_Jac_163/SQ_SQ4_PD/Mxor_GND_10_o_GND_10_o_xor_192_OUT_163_xo<0>10" is
sourceless and has been removed.
The signal
"uut_PD_PA_Jac_163/SQ_SQ1_PD/Mxor_GND_10_o_GND_10_o_xor_64_OUT_163_xo<0>" is
sourceless and has been removed.
The signal
"uut_PD_PA_Jac_163/SQ_SQ1_PD/Mxor_GND_10_o_GND_10_o_xor_64_OUT_163_xo<0>1" is
sourceless and has been removed.
The signal
"uut_PD_PA_Jac_163/SQ_SQ1_PD/Mxor_GND_10_o_GND_10_o_xor_64_OUT_163_xo<0>2" is
sourceless and has been removed.
The signal
"uut_PD_PA_Jac_163/SQ_SQ1_PD/Mxor_GND_10_o_GND_10_o_xor_64_OUT_163_xo<0>3" is
sourceless and has been removed.
The signal "uut_PD_PA_Jac_163/SQ_SQ1_PD/Mxor_SQ_BF.Cv_29_xo<0>" is sourceless
and has been removed.
The signal "uut_PD_PA_Jac_163/SQ_SQ1_PD/Mxor_SQ_BF.Cv_29_xo<0>1" is sourceless
and has been removed.
The signal "uut_PD_PA_Jac_163/SQ_SQ1_PD/Mxor_SQ_BF.Cv_29_xo<0>2" is sourceless
and has been removed.
The signal "uut_PD_PA_Jac_163/SQ_SQ1_PD/Mxor_SQ_BF.Cv_29_xo<0>3" is sourceless
and has been removed.
The signal "uut_PD_PA_Jac_163/SQ_SQ1_PD/Mxor_SQ_BF.Cv_46_xo<0>13" is sourceless
and has been removed.
The signal

"uut_PD_PA_Jac_163/SQ_SQ1_PD/Mxor_GND_10_o_GND_10_o_xor_192_OUT_163_xo<0>10" is
sourceless and has been removed.
The signal
"uut_PD_PA_Jac_163/SQ_SQ2_PD/Mxor_GND_10_o_GND_10_o_xor_64_OUT_163_xo<0>" is
sourceless and has been removed.
The signal
"uut_PD_PA_Jac_163/SQ_SQ2_PD/Mxor_GND_10_o_GND_10_o_xor_64_OUT_163_xo<0>1" is
sourceless and has been removed.
The signal
"uut_PD_PA_Jac_163/SQ_SQ2_PD/Mxor_GND_10_o_GND_10_o_xor_64_OUT_163_xo<0>2" is
sourceless and has been removed.
The signal
"uut_PD_PA_Jac_163/SQ_SQ2_PD/Mxor_GND_10_o_GND_10_o_xor_64_OUT_163_xo<0>3" is
sourceless and has been removed.
The signal "uut_PD_PA_Jac_163/SQ_SQ2_PD/Mxor_SQ_BF.Cv_29_xo<0>" is sourceless
and has been removed.
The signal "uut_PD_PA_Jac_163/SQ_SQ2_PD/Mxor_SQ_BF.Cv_29_xo<0>1" is sourceless
and has been removed.
The signal "uut_PD_PA_Jac_163/SQ_SQ2_PD/Mxor_SQ_BF.Cv_29_xo<0>2" is sourceless
and has been removed.
The signal "uut_PD_PA_Jac_163/SQ_SQ2_PD/Mxor_SQ_BF.Cv_29_xo<0>3" is sourceless
and has been removed.
The signal "uut_PD_PA_Jac_163/SQ_SQ2_PD/Mxor_SQ_BF.Cv_46_xo<0>13" is sourceless
and has been removed.
The signal
"uut_PD_PA_Jac_163/SQ_SQ2_PD/Mxor_GND_10_o_GND_10_o_xor_192_OUT_163_xo<0>10" is
sourceless and has been removed.
Unused block
"uut_PD_PA_Jac_163/SQ_SQ1_PA/Mxor_GND_10_o_GND_10_o_xor_192_OUT_163_xo<0>11"
(ROM) removed.
Unused block
"uut_PD_PA_Jac_163/SQ_SQ1_PA/Mxor_GND_10_o_GND_10_o_xor_64_OUT_163_xo<0>1" (ROM)
removed.
Unused block
"uut_PD_PA_Jac_163/SQ_SQ1_PA/Mxor_GND_10_o_GND_10_o_xor_64_OUT_163_xo<0>2" (ROM)
removed.
Unused block
"uut_PD_PA_Jac_163/SQ_SQ1_PA/Mxor_GND_10_o_GND_10_o_xor_64_OUT_163_xo<0>3" (ROM)
removed.
Unused block
"uut_PD_PA_Jac_163/SQ_SQ1_PA/Mxor_GND_10_o_GND_10_o_xor_64_OUT_163_xo<0>4" (ROM)
removed.
Unused block "uut_PD_PA_Jac_163/SQ_SQ1_PA/Mxor_SQ_BF.Cv_29_xo<0>1" (ROM)
removed.
Unused block "uut_PD_PA_Jac_163/SQ_SQ1_PA/Mxor_SQ_BF.Cv_29_xo<0>2" (ROM)
removed.
Unused block "uut_PD_PA_Jac_163/SQ_SQ1_PA/Mxor_SQ_BF.Cv_29_xo<0>3" (ROM)
removed.
Unused block "uut_PD_PA_Jac_163/SQ_SQ1_PA/Mxor_SQ_BF.Cv_29_xo<0>4" (ROM)
removed.
Unused block "uut_PD_PA_Jac_163/SQ_SQ1_PA/Mxor_SQ_BF.Cv_46_xo<0>8" (ROM)
removed.
Unused block
"uut_PD_PA_Jac_163/SQ_SQ1_PD/Mxor_GND_10_o_GND_10_o_xor_192_OUT_163_xo<0>11"
(ROM) removed.
Unused block
"uut_PD_PA_Jac_163/SQ_SQ1_PD/Mxor_GND_10_o_GND_10_o_xor_64_OUT_163_xo<0>1" (ROM)
removed.
Unused block
"uut_PD_PA_Jac_163/SQ_SQ1_PD/Mxor_GND_10_o_GND_10_o_xor_64_OUT_163_xo<0>2" (ROM)
removed.
Unused block
"uut_PD_PA_Jac_163/SQ_SQ1_PD/Mxor_GND_10_o_GND_10_o_xor_64_OUT_163_xo<0>3" (ROM)
removed.
Unused block
"uut_PD_PA_Jac_163/SQ_SQ1_PD/Mxor_GND_10_o_GND_10_o_xor_64_OUT_163_xo<0>4" (ROM)
removed.
Unused block "uut_PD_PA_Jac_163/SQ_SQ1_PD/Mxor_SQ_BF.Cv_29_xo<0>1" (ROM)
removed.
Unused block "uut_PD_PA_Jac_163/SQ_SQ1_PD/Mxor_SQ_BF.Cv_29_xo<0>2" (ROM)
removed.

```
Unused block "uut_PD_PA_Jac_163/SQ_SQ1_PD/Mxor_SQ_BF.Cv_29_xo<0>3" (ROM)
removed.
Unused block "uut_PD_PA_Jac_163/SQ_SQ1_PD/Mxor_SQ_BF.Cv_29_xo<0>4" (ROM)
removed.
Unused block "uut_PD_PA_Jac_163/SQ_SQ1_PD/Mxor_SQ_BF.Cv_46_xo<0>8" (ROM)
removed.
Unused block "uut_PD_PA_Jac_163/SQ_SQ2_PA/Mxor_SQ_BF.Cv_29_xo<0>1" (ROM)
removed.
Unused block "uut_PD_PA_Jac_163/SQ_SQ2_PA/Mxor_SQ_BF.Cv_29_xo<0>2" (ROM)
removed.
Unused block "uut_PD_PA_Jac_163/SQ_SQ2_PA/Mxor_SQ_BF.Cv_29_xo<0>3" (ROM)
removed.
Unused block "uut_PD_PA_Jac_163/SQ_SQ2_PA/Mxor_SQ_BF.Cv_29_xo<0>4" (ROM)
removed.
Unused block
"uut_PD_PA_Jac_163/SQ_SQ2_PD/Mxor_GND_10_o_GND_10_o_xor_192_OUT_163_xo<0>11"
(ROM) removed.
Unused block
"uut_PD_PA_Jac_163/SQ_SQ2_PD/Mxor_GND_10_o_GND_10_o_xor_64_OUT_163_xo<0>1" (ROM)
removed.
Unused block
"uut_PD_PA_Jac_163/SQ_SQ2_PD/Mxor_GND_10_o_GND_10_o_xor_64_OUT_163_xo<0>2" (ROM)
removed.
Unused block
"uut_PD_PA_Jac_163/SQ_SQ2_PD/Mxor_GND_10_o_GND_10_o_xor_64_OUT_163_xo<0>3" (ROM)
removed.
Unused block
"uut_PD_PA_Jac_163/SQ_SQ2_PD/Mxor_GND_10_o_GND_10_o_xor_64_OUT_163_xo<0>4" (ROM)
removed.
Unused block "uut_PD_PA_Jac_163/SQ_SQ2_PD/Mxor_SQ_BF.Cv_29_xo<0>1" (ROM)
removed.
Unused block "uut_PD_PA_Jac_163/SQ_SQ2_PD/Mxor_SQ_BF.Cv_29_xo<0>2" (ROM)
removed.
Unused block "uut_PD_PA_Jac_163/SQ_SQ2_PD/Mxor_SQ_BF.Cv_29_xo<0>3" (ROM)
removed.
Unused block "uut_PD_PA_Jac_163/SQ_SQ2_PD/Mxor_SQ_BF.Cv_29_xo<0>4" (ROM)
removed.
Unused block "uut_PD_PA_Jac_163/SQ_SQ2_PD/Mxor_SQ_BF.Cv_46_xo<0>8" (ROM)
removed.
Unused block "uut_PD_PA_Jac_163/SQ_SQ3_PA/Mxor_SQ_BF.Cv_29_xo<0>1" (ROM)
removed.
Unused block "uut_PD_PA_Jac_163/SQ_SQ3_PA/Mxor_SQ_BF.Cv_29_xo<0>2" (ROM)
removed.
Unused block "uut_PD_PA_Jac_163/SQ_SQ3_PA/Mxor_SQ_BF.Cv_29_xo<0>3" (ROM)
removed.
Unused block "uut_PD_PA_Jac_163/SQ_SQ3_PA/Mxor_SQ_BF.Cv_29_xo<0>4" (ROM)
removed.
Unused block "uut_PD_PA_Jac_163/SQ_SQ3_PA/Mxor_SQ_BF.Cv_46_xo<0>8" (ROM)
removed.
Unused block
"uut_PD_PA_Jac_163/SQ_SQ3_PD/Mxor_GND_10_o_GND_10_o_xor_192_OUT_163_xo<0>11"
(ROM) removed.
Unused block
"uut_PD_PA_Jac_163/SQ_SQ3_PD/Mxor_GND_10_o_GND_10_o_xor_64_OUT_163_xo<0>1" (ROM)
removed.
Unused block
"uut_PD_PA_Jac_163/SQ_SQ3_PD/Mxor_GND_10_o_GND_10_o_xor_64_OUT_163_xo<0>2" (ROM)
removed.
Unused block
"uut_PD_PA_Jac_163/SQ_SQ3_PD/Mxor_GND_10_o_GND_10_o_xor_64_OUT_163_xo<0>3" (ROM)
removed.
Unused block
"uut_PD_PA_Jac_163/SQ_SQ3_PD/Mxor_GND_10_o_GND_10_o_xor_64_OUT_163_xo<0>4" (ROM)
removed.
Unused block "uut_PD_PA_Jac_163/SQ_SQ3_PD/Mxor_SQ_BF.Cv_29_xo<0>1" (ROM)
removed.
Unused block "uut_PD_PA_Jac_163/SQ_SQ3_PD/Mxor_SQ_BF.Cv_29_xo<0>2" (ROM)
removed.
Unused block "uut_PD_PA_Jac_163/SQ_SQ3_PD/Mxor_SQ_BF.Cv_29_xo<0>3" (ROM)
removed.
Unused block "uut_PD_PA_Jac_163/SQ_SQ3_PD/Mxor_SQ_BF.Cv_29_xo<0>4" (ROM)
```

```
removed.
Unused block "uut_PD_PA_Jac_163/SQ_SQ3_PD/Mxor_SQ_BF.Cv_46_xo<0>8" (ROM)
removed.
Unused block
"uut_PD_PA_Jac_163/SQ_SQ4_PD/Mxor_GND_10_o_GND_10_o_xor_192_OUT_163_xo<0>11"
(ROM) removed.
Unused block
"uut_PD_PA_Jac_163/SQ_SQ4_PD/Mxor_GND_10_o_GND_10_o_xor_64_OUT_163_xo<0>1" (ROM)
removed.
Unused block
"uut_PD_PA_Jac_163/SQ_SQ4_PD/Mxor_GND_10_o_GND_10_o_xor_64_OUT_163_xo<0>2" (ROM)
removed.
Unused block
"uut_PD_PA_Jac_163/SQ_SQ4_PD/Mxor_GND_10_o_GND_10_o_xor_64_OUT_163_xo<0>3" (ROM)
removed.
Unused block
"uut_PD_PA_Jac_163/SQ_SQ4_PD/Mxor_GND_10_o_GND_10_o_xor_64_OUT_163_xo<0>4" (ROM)
removed.
Unused block "uut_PD_PA_Jac_163/SQ_SQ4_PD/Mxor_SQ_BF.Cv_29_xo<0>1" (ROM)
removed.
Unused block "uut_PD_PA_Jac_163/SQ_SQ4_PD/Mxor_SQ_BF.Cv_29_xo<0>2" (ROM)
removed.
Unused block "uut_PD_PA_Jac_163/SQ_SQ4_PD/Mxor_SQ_BF.Cv_29_xo<0>3" (ROM)
removed.
Unused block "uut_PD_PA_Jac_163/SQ_SQ4_PD/Mxor_SQ_BF.Cv_29_xo<0>4" (ROM)
removed.
Unused block "uut_PD_PA_Jac_163/SQ_SQ4_PD/Mxor_SQ_BF.Cv_46_xo<0>8" (ROM)
removed.
Unused block
"uut_PD_PA_Jac_163/SQ_SQ5_PD/Mxor_GND_10_o_GND_10_o_xor_192_OUT_163_xo<0>11"
(ROM) removed.
Unused block
"uut_PD_PA_Jac_163/SQ_SQ5_PD/Mxor_GND_10_o_GND_10_o_xor_64_OUT_163_xo<0>1" (ROM)
removed.
Unused block
"uut_PD_PA_Jac_163/SQ_SQ5_PD/Mxor_GND_10_o_GND_10_o_xor_64_OUT_163_xo<0>2" (ROM)
removed.
Unused block
"uut_PD_PA_Jac_163/SQ_SQ5_PD/Mxor_GND_10_o_GND_10_o_xor_64_OUT_163_xo<0>3" (ROM)
removed.
Unused block
"uut_PD_PA_Jac_163/SQ_SQ5_PD/Mxor_GND_10_o_GND_10_o_xor_64_OUT_163_xo<0>4" (ROM)
removed.
Unused block "uut_PD_PA_Jac_163/SQ_SQ5_PD/Mxor_SQ_BF.Cv_29_xo<0>1" (ROM)
removed.
Unused block "uut_PD_PA_Jac_163/SQ_SQ5_PD/Mxor_SQ_BF.Cv_29_xo<0>2" (ROM)
removed.
Unused block "uut_PD_PA_Jac_163/SQ_SQ5_PD/Mxor_SQ_BF.Cv_29_xo<0>3" (ROM)
removed.
Unused block "uut_PD_PA_Jac_163/SQ_SQ5_PD/Mxor_SQ_BF.Cv_29_xo<0>4" (ROM)
removed.
Unused block "uut_PD_PA_Jac_163/SQ_SQ5_PD/Mxor_SQ_BF.Cv_46_xo<0>8" (ROM)
removed.

Optimized Block(s):
TYPE        BLOCK
GND         XST_GND
VCC         XST_VCC
GND         uut_PD_PA_Jac_163/SQ_SQ1_PA/XST_GND
GND         uut_PD_PA_Jac_163/SQ_SQ1_PD/XST_GND
GND         uut_PD_PA_Jac_163/SQ_SQ2_PA/XST_GND
GND         uut_PD_PA_Jac_163/SQ_SQ2_PD/XST_GND
GND         uut_PD_PA_Jac_163/SQ_SQ3_PA/XST_GND
GND         uut_PD_PA_Jac_163/SQ_SQ3_PD/XST_GND
GND         uut_PD_PA_Jac_163/SQ_SQ4_PD/XST_GND
GND         uut_PD_PA_Jac_163/SQ_SQ5_PD/XST_GND
GND         uut_PD_PA_Jac_163/XST_GND
VCC         uut_PD_PA_Jac_163/XST_VCC
GND         uut_PD_PA_Jac_163/mult_M2_PA/XST_GND
VCC         uut_PD_PA_Jac_163/mult_M2_PA/XST_VCC
GND         uut_PD_PA_Jac_163/mult_M3_PA/XST_GND
```

```
GND          uut_PD_PA_Jac_163/mult_M4_PD/XST_GND
VCC          uut_PD_PA_Jac_163/mult_M4_PD/XST_VCC
GND          uut_PD_PA_Jac_163/mult_M9_PA/XST_GND
VCC          uut_PD_PA_Jac_163/mult_M9_PA/XST_VCC
LUT5         uut_PD_PA_Jac_163/SQ_SQ1_PA/Mxor_GND_10_o_GND_10_o_xor_64_OUT_163_xo<0>10
   optimized to 0
LUT4         uut_PD_PA_Jac_163/SQ_SQ1_PA/Mxor_GND_10_o_GND_10_o_xor_64_OUT_163_xo<0>5
    Property STUCK_AT NOT found
LUT5         uut_PD_PA_Jac_163/SQ_SQ1_PA/Mxor_SQ_BF.Cv_29_xo<0>10
   optimized to 0
LUT4         uut_PD_PA_Jac_163/SQ_SQ1_PA/Mxor_SQ_BF.Cv_29_xo<0>5
    Property STUCK_AT NOT found
LUT5         uut_PD_PA_Jac_163/SQ_SQ1_PD/Mxor_GND_10_o_GND_10_o_xor_64_OUT_163_xo<0>10
   optimized to 0
LUT4         uut_PD_PA_Jac_163/SQ_SQ1_PD/Mxor_GND_10_o_GND_10_o_xor_64_OUT_163_xo<0>5
    Property STUCK_AT NOT found
LUT5         uut_PD_PA_Jac_163/SQ_SQ1_PD/Mxor_SQ_BF.Cv_29_xo<0>10
   optimized to 0
LUT4         uut_PD_PA_Jac_163/SQ_SQ1_PD/Mxor_SQ_BF.Cv_29_xo<0>5
    Property STUCK_AT NOT found
LUT5         uut_PD_PA_Jac_163/SQ_SQ2_PA/Mxor_SQ_BF.Cv_29_xo<0>10
   optimized to 0
LUT4         uut_PD_PA_Jac_163/SQ_SQ2_PA/Mxor_SQ_BF.Cv_29_xo<0>5
    Property STUCK_AT NOT found
LUT5         uut_PD_PA_Jac_163/SQ_SQ2_PD/Mxor_GND_10_o_GND_10_o_xor_64_OUT_163_xo<0>10
   optimized to 0
LUT4         uut_PD_PA_Jac_163/SQ_SQ2_PD/Mxor_GND_10_o_GND_10_o_xor_64_OUT_163_xo<0>5
    Property STUCK_AT NOT found
LUT5         uut_PD_PA_Jac_163/SQ_SQ2_PD/Mxor_SQ_BF.Cv_29_xo<0>10
   optimized to 0
LUT4         uut_PD_PA_Jac_163/SQ_SQ2_PD/Mxor_SQ_BF.Cv_29_xo<0>5
    Property STUCK_AT NOT found
LUT5         uut_PD_PA_Jac_163/SQ_SQ3_PA/Mxor_SQ_BF.Cv_29_xo<0>10
   optimized to 0
LUT4         uut_PD_PA_Jac_163/SQ_SQ3_PA/Mxor_SQ_BF.Cv_29_xo<0>5
    Property STUCK_AT NOT found
LUT5         uut_PD_PA_Jac_163/SQ_SQ3_PD/Mxor_GND_10_o_GND_10_o_xor_64_OUT_163_xo<0>10
   optimized to 0
LUT4         uut_PD_PA_Jac_163/SQ_SQ3_PD/Mxor_GND_10_o_GND_10_o_xor_64_OUT_163_xo<0>5
    Property STUCK_AT NOT found
LUT5         uut_PD_PA_Jac_163/SQ_SQ3_PD/Mxor_SQ_BF.Cv_29_xo<0>10
   optimized to 0
LUT4         uut_PD_PA_Jac_163/SQ_SQ3_PD/Mxor_SQ_BF.Cv_29_xo<0>5
    Property STUCK_AT NOT found
LUT5         uut_PD_PA_Jac_163/SQ_SQ4_PD/Mxor_GND_10_o_GND_10_o_xor_64_OUT_163_xo<0>10
   optimized to 0
LUT4         uut_PD_PA_Jac_163/SQ_SQ4_PD/Mxor_GND_10_o_GND_10_o_xor_64_OUT_163_xo<0>5
    Property STUCK_AT NOT found
LUT5         uut_PD_PA_Jac_163/SQ_SQ4_PD/Mxor_SQ_BF.Cv_29_xo<0>10
   optimized to 0
LUT4         uut_PD_PA_Jac_163/SQ_SQ4_PD/Mxor_SQ_BF.Cv_29_xo<0>5
    Property STUCK_AT NOT found
LUT5         uut_PD_PA_Jac_163/SQ_SQ5_PD/Mxor_GND_10_o_GND_10_o_xor_64_OUT_163_xo<0>10
   optimized to 0
LUT4         uut_PD_PA_Jac_163/SQ_SQ5_PD/Mxor_GND_10_o_GND_10_o_xor_64_OUT_163_xo<0>5
    Property STUCK_AT NOT found
LUT5         uut_PD_PA_Jac_163/SQ_SQ5_PD/Mxor_SQ_BF.Cv_29_xo<0>10
   optimized to 0
LUT4         uut_PD_PA_Jac_163/SQ_SQ5_PD/Mxor_SQ_BF.Cv_29_xo<0>5
    Property STUCK_AT NOT found

To enable printing of redundant blocks removed and signals merged, set the
detailed map report option and rerun map.

Section 6 - IOB Properties
--------------------------

+---------------------------------------------------------------------------------------------------
----------------------------------------------------------------+
| IOB Name                              | Type             | Direction | IO Standard          |
Diff  | Drive     | Slew | Reg (s)      | Resistor | IOB      |
```

```
|                                      |                   |            |                       |
Term  | Strength | Rate |              |           | Delay    |
+-----------------------------------------------------------------------------------------------
-------------------------------------------------------------+
| QX<0>                                | IOB               | OUTPUT     | LVCMOS25
|       | 12       | SLOW |               |           |        |
| QX<1>                                | IOB               | OUTPUT     | LVCMOS25
|       | 12       | SLOW |               |           |        |
| QX<2>                                | IOB               | OUTPUT     | LVCMOS25
|       | 12       | SLOW |               |           |        |
| QX<3>                                | IOB               | OUTPUT     | LVCMOS25
|       | 12       | SLOW |               |           |        |
| QX<4>                                | IOB               | OUTPUT     | LVCMOS25
|       | 12       | SLOW |               |           |        |
| QX<5>                                | IOB               | OUTPUT     | LVCMOS25
|       | 12       | SLOW |               |           |        |
| QX<6>                                | IOB               | OUTPUT     | LVCMOS25
|       | 12       | SLOW |               |           |        |
| QX<7>                                | IOB               | OUTPUT     | LVCMOS25
|       | 12       | SLOW |               |           |        |
| QX<8>                                | IOB               | OUTPUT     | LVCMOS25
|       | 12       | SLOW |               |           |        |
| QX<9>                                | IOB               | OUTPUT     | LVCMOS25
|       | 12       | SLOW |               |           |        |
| QX<10>                               | IOB               | OUTPUT     | LVCMOS25
|       | 12       | SLOW |               |           |        |
| QX<11>                               | IOB               | OUTPUT     | LVCMOS25
|       | 12       | SLOW |               |           |        |
| QX<12>                               | IOB               | OUTPUT     | LVCMOS25
|       | 12       | SLOW |               |           |        |
| QX<13>                               | IOB               | OUTPUT     | LVCMOS25
|       | 12       | SLOW |               |           |        |
| QX<14>                               | IOB               | OUTPUT     | LVCMOS25
|       | 12       | SLOW |               |           |        |
| QX<15>                               | IOB               | OUTPUT     | LVCMOS25
|       | 12       | SLOW |               |           |        |
| QX<16>                               | IOB               | OUTPUT     | LVCMOS25
|       | 12       | SLOW |               |           |        |
| QX<17>                               | IOB               | OUTPUT     | LVCMOS25
|       | 12       | SLOW |               |           |        |
| QX<18>                               | IOB               | OUTPUT     | LVCMOS25
|       | 12       | SLOW |               |           |        |
| QX<19>                               | IOB               | OUTPUT     | LVCMOS25
|       | 12       | SLOW |               |           |        |
| QX<20>                               | IOB               | OUTPUT     | LVCMOS25
|       | 12       | SLOW |               |           |        |
| QX<21>                               | IOB               | OUTPUT     | LVCMOS25
|       | 12       | SLOW |               |           |        |
| QX<22>                               | IOB               | OUTPUT     | LVCMOS25
|       | 12       | SLOW |               |           |        |
| QX<23>                               | IOB               | OUTPUT     | LVCMOS25
|       | 12       | SLOW |               |           |        |
| QX<24>                               | IOB               | OUTPUT     | LVCMOS25
|       | 12       | SLOW |               |           |        |
| QX<25>                               | IOB               | OUTPUT     | LVCMOS25
|       | 12       | SLOW |               |           |        |
| QX<26>                               | IOB               | OUTPUT     | LVCMOS25
|       | 12       | SLOW |               |           |        |
| QX<27>                               | IOB               | OUTPUT     | LVCMOS25
|       | 12       | SLOW |               |           |        |
| QX<28>                               | IOB               | OUTPUT     | LVCMOS25
|       | 12       | SLOW |               |           |        |
| QX<29>                               | IOB               | OUTPUT     | LVCMOS25
|       | 12       | SLOW |               |           |        |
| QX<30>                               | IOB               | OUTPUT     | LVCMOS25
|       | 12       | SLOW |               |           |        |
| QX<31>                               | IOB               | OUTPUT     | LVCMOS25
|       | 12       | SLOW |               |           |        |
| QX<32>                               | IOB               | OUTPUT     | LVCMOS25
|       | 12       | SLOW |               |           |        |
| QX<33>                               | IOB               | OUTPUT     | LVCMOS25
```

```
|        | 12        | SLOW |              |          |          |
| QX<34>                          | IOB          | OUTPUT     | LVCMOS25
|        | 12        | SLOW |              |          |          |
| QX<35>                          | IOB          | OUTPUT     | LVCMOS25
|        | 12        | SLOW |              |          |          |
| QX<36>                          | IOB          | OUTPUT     | LVCMOS25
|        | 12        | SLOW |              |          |          |
| QX<37>                          | IOB          | OUTPUT     | LVCMOS25
|        | 12        | SLOW |              |          |          |
| QX<38>                          | IOB          | OUTPUT     | LVCMOS25
|        | 12        | SLOW |              |          |          |
| QX<39>                          | IOB          | OUTPUT     | LVCMOS25
|        | 12        | SLOW |              |          |          |
| QX<40>                          | IOB          | OUTPUT     | LVCMOS25
|        | 12        | SLOW |              |          |          |
| QX<41>                          | IOB          | OUTPUT     | LVCMOS25
|        | 12        | SLOW |              |          |          |
| QX<42>                          | IOB          | OUTPUT     | LVCMOS25
|        | 12        | SLOW |              |          |          |
| QX<43>                          | IOB          | OUTPUT     | LVCMOS25
|        | 12        | SLOW |              |          |          |
| QX<44>                          | IOB          | OUTPUT     | LVCMOS25
|        | 12        | SLOW |              |          |          |
| QX<45>                          | IOB          | OUTPUT     | LVCMOS25
|        | 12        | SLOW |              |          |          |
| QX<46>                          | IOB          | OUTPUT     | LVCMOS25
|        | 12        | SLOW |              |          |          |
| QX<47>                          | IOB          | OUTPUT     | LVCMOS25
|        | 12        | SLOW |              |          |          |
| QX<48>                          | IOB          | OUTPUT     | LVCMOS25
|        | 12        | SLOW |              |          |          |
| QX<49>                          | IOB          | OUTPUT     | LVCMOS25
|        | 12        | SLOW |              |          |          |
| QX<50>                          | IOB          | OUTPUT     | LVCMOS25
|        | 12        | SLOW |              |          |          |
| QX<51>                          | IOB          | OUTPUT     | LVCMOS25
|        | 12        | SLOW |              |          |          |
| QX<52>                          | IOB          | OUTPUT     | LVCMOS25
|        | 12        | SLOW |              |          |          |
| QX<53>                          | IOB          | OUTPUT     | LVCMOS25
|        | 12        | SLOW |              |          |          |
| QX<54>                          | IOB          | OUTPUT     | LVCMOS25
|        | 12        | SLOW |              |          |          |
| QX<55>                          | IOB          | OUTPUT     | LVCMOS25
|        | 12        | SLOW |              |          |          |
| QX<56>                          | IOB          | OUTPUT     | LVCMOS25
|        | 12        | SLOW |              |          |          |
| QX<57>                          | IOB          | OUTPUT     | LVCMOS25
|        | 12        | SLOW |              |          |          |
| QX<58>                          | IOB          | OUTPUT     | LVCMOS25
|        | 12        | SLOW |              |          |          |
| QX<59>                          | IOB          | OUTPUT     | LVCMOS25
|        | 12        | SLOW |              |          |          |
| QX<60>                          | IOB          | OUTPUT     | LVCMOS25
|        | 12        | SLOW |              |          |          |
| QX<61>                          | IOB          | OUTPUT     | LVCMOS25
|        | 12        | SLOW |              |          |          |
| QX<62>                          | IOB          | OUTPUT     | LVCMOS25
|        | 12        | SLOW |              |          |          |
| QX<63>                          | IOB          | OUTPUT     | LVCMOS25
|        | 12        | SLOW |              |          |          |
| QX<64>                          | IOB          | OUTPUT     | LVCMOS25
|        | 12        | SLOW |              |          |          |
| QX<65>                          | IOB          | OUTPUT     | LVCMOS25
|        | 12        | SLOW |              |          |          |
| QX<66>                          | IOB          | OUTPUT     | LVCMOS25
|        | 12        | SLOW |              |          |          |
| QX<67>                          | IOB          | OUTPUT     | LVCMOS25
|        | 12        | SLOW |              |          |          |
| QX<68>                          | IOB          | OUTPUT     | LVCMOS25
|        | 12        | SLOW |              |          |          |
```

```
| QX<69>                                 | IOB                | OUTPUT     | LVCMOS25
|        | 12        | SLOW |                  |           |           |
| QX<70>                                 | IOB                | OUTPUT     | LVCMOS25
|        | 12        | SLOW |                  |           |           |
| QX<71>                                 | IOB                | OUTPUT     | LVCMOS25
|        | 12        | SLOW |                  |           |           |
| QX<72>                                 | IOB                | OUTPUT     | LVCMOS25
|        | 12        | SLOW |                  |           |           |
| QX<73>                                 | IOB                | OUTPUT     | LVCMOS25
|        | 12        | SLOW |                  |           |           |
| QX<74>                                 | IOB                | OUTPUT     | LVCMOS25
|        | 12        | SLOW |                  |           |           |
| QX<75>                                 | IOB                | OUTPUT     | LVCMOS25
|        | 12        | SLOW |                  |           |           |
| QX<76>                                 | IOB                | OUTPUT     | LVCMOS25
|        | 12        | SLOW |                  |           |           |
| QX<77>                                 | IOB                | OUTPUT     | LVCMOS25
|        | 12        | SLOW |                  |           |           |
| QX<78>                                 | IOB                | OUTPUT     | LVCMOS25
|        | 12        | SLOW |                  |           |           |
| QX<79>                                 | IOB                | OUTPUT     | LVCMOS25
|        | 12        | SLOW |                  |           |           |
| QX<80>                                 | IOB                | OUTPUT     | LVCMOS25
|        | 12        | SLOW |                  |           |           |
| QX<81>                                 | IOB                | OUTPUT     | LVCMOS25
|        | 12        | SLOW |                  |           |           |
| QX<82>                                 | IOB                | OUTPUT     | LVCMOS25
|        | 12        | SLOW |                  |           |           |
| QX<83>                                 | IOB                | OUTPUT     | LVCMOS25
|        | 12        | SLOW |                  |           |           |
| QX<84>                                 | IOB                | OUTPUT     | LVCMOS25
|        | 12        | SLOW |                  |           |           |
| QX<85>                                 | IOB                | OUTPUT     | LVCMOS25
|        | 12        | SLOW |                  |           |           |
| QX<86>                                 | IOB                | OUTPUT     | LVCMOS25
|        | 12        | SLOW |                  |           |           |
| QX<87>                                 | IOB                | OUTPUT     | LVCMOS25
|        | 12        | SLOW |                  |           |           |
| QX<88>                                 | IOB                | OUTPUT     | LVCMOS25
|        | 12        | SLOW |                  |           |           |
| QX<89>                                 | IOB                | OUTPUT     | LVCMOS25
|        | 12        | SLOW |                  |           |           |
| QX<90>                                 | IOB                | OUTPUT     | LVCMOS25
|        | 12        | SLOW |                  |           |           |
| QX<91>                                 | IOB                | OUTPUT     | LVCMOS25
|        | 12        | SLOW |                  |           |           |
| QX<92>                                 | IOB                | OUTPUT     | LVCMOS25
|        | 12        | SLOW |                  |           |           |
| QX<93>                                 | IOB                | OUTPUT     | LVCMOS25
|        | 12        | SLOW |                  |           |           |
| QX<94>                                 | IOB                | OUTPUT     | LVCMOS25
|        | 12        | SLOW |                  |           |           |
| QX<95>                                 | IOB                | OUTPUT     | LVCMOS25
|        | 12        | SLOW |                  |           |           |
| QX<96>                                 | IOB                | OUTPUT     | LVCMOS25
|        | 12        | SLOW |                  |           |           |
| QX<97>                                 | IOB                | OUTPUT     | LVCMOS25
|        | 12        | SLOW |                  |           |           |
| QX<98>                                 | IOB                | OUTPUT     | LVCMOS25
|        | 12        | SLOW |                  |           |           |
| QX<99>                                 | IOB                | OUTPUT     | LVCMOS25
|        | 12        | SLOW |                  |           |           |
| QX<100>                                | IOB                | OUTPUT     | LVCMOS25
|        | 12        | SLOW |                  |           |           |
| QX<101>                                | IOB                | OUTPUT     | LVCMOS25
|        | 12        | SLOW |                  |           |           |
| QX<102>                                | IOB                | OUTPUT     | LVCMOS25
|        | 12        | SLOW |                  |           |           |
| QX<103>                                | IOB                | OUTPUT     | LVCMOS25
|        | 12        | SLOW |                  |           |           |
| QX<104>                                | IOB                | OUTPUT     | LVCMOS25
```

```
|        | 12        | SLOW |                |           |          |
| QX<105>                             | IOB              | OUTPUT     | LVCMOS25
|        | 12        | SLOW |                |           |          |
| QX<106>                             | IOB              | OUTPUT     | LVCMOS25
|        | 12        | SLOW |                |           |          |
| QX<107>                             | IOB              | OUTPUT     | LVCMOS25
|        | 12        | SLOW |                |           |          |
| QX<108>                             | IOB              | OUTPUT     | LVCMOS25
|        | 12        | SLOW |                |           |          |
| QX<109>                             | IOB              | OUTPUT     | LVCMOS25
|        | 12        | SLOW |                |           |          |
| QX<110>                             | IOB              | OUTPUT     | LVCMOS25
|        | 12        | SLOW |                |           |          |
| QX<111>                             | IOB              | OUTPUT     | LVCMOS25
|        | 12        | SLOW |                |           |          |
| QX<112>                             | IOB              | OUTPUT     | LVCMOS25
|        | 12        | SLOW |                |           |          |
| QX<113>                             | IOB              | OUTPUT     | LVCMOS25
|        | 12        | SLOW |                |           |          |
| QX<114>                             | IOB              | OUTPUT     | LVCMOS25
|        | 12        | SLOW |                |           |          |
| QX<115>                             | IOB              | OUTPUT     | LVCMOS25
|        | 12        | SLOW |                |           |          |
| QX<116>                             | IOB              | OUTPUT     | LVCMOS25
|        | 12        | SLOW |                |           |          |
| QX<117>                             | IOB              | OUTPUT     | LVCMOS25
|        | 12        | SLOW |                |           |          |
| QX<118>                             | IOB              | OUTPUT     | LVCMOS25
|        | 12        | SLOW |                |           |          |
| QX<119>                             | IOB              | OUTPUT     | LVCMOS25
|        | 12        | SLOW |                |           |          |
| QX<120>                             | IOB              | OUTPUT     | LVCMOS25
|        | 12        | SLOW |                |           |          |
| QX<121>                             | IOB              | OUTPUT     | LVCMOS25
|        | 12        | SLOW |                |           |          |
| QX<122>                             | IOB              | OUTPUT     | LVCMOS25
|        | 12        | SLOW |                |           |          |
| QX<123>                             | IOB              | OUTPUT     | LVCMOS25
|        | 12        | SLOW |                |           |          |
| QX<124>                             | IOB              | OUTPUT     | LVCMOS25
|        | 12        | SLOW |                |           |          |
| QX<125>                             | IOB              | OUTPUT     | LVCMOS25
|        | 12        | SLOW |                |           |          |
| QX<126>                             | IOB              | OUTPUT     | LVCMOS25
|        | 12        | SLOW |                |           |          |
| QX<127>                             | IOB              | OUTPUT     | LVCMOS25
|        | 12        | SLOW |                |           |          |
| QX<128>                             | IOB              | OUTPUT     | LVCMOS25
|        | 12        | SLOW |                |           |          |
| QX<129>                             | IOB              | OUTPUT     | LVCMOS25
|        | 12        | SLOW |                |           |          |
| QX<130>                             | IOB              | OUTPUT     | LVCMOS25
|        | 12        | SLOW |                |           |          |
| QX<131>                             | IOB              | OUTPUT     | LVCMOS25
|        | 12        | SLOW |                |           |          |
| QX<132>                             | IOB              | OUTPUT     | LVCMOS25
|        | 12        | SLOW |                |           |          |
| QX<133>                             | IOB              | OUTPUT     | LVCMOS25
|        | 12        | SLOW |                |           |          |
| QX<134>                             | IOB              | OUTPUT     | LVCMOS25
|        | 12        | SLOW |                |           |          |
| QX<135>                             | IOB              | OUTPUT     | LVCMOS25
|        | 12        | SLOW |                |           |          |
| QX<136>                             | IOB              | OUTPUT     | LVCMOS25
|        | 12        | SLOW |                |           |          |
| QX<137>                             | IOB              | OUTPUT     | LVCMOS25
|        | 12        | SLOW |                |           |          |
| QX<138>                             | IOB              | OUTPUT     | LVCMOS25
|        | 12        | SLOW |                |           |          |
| QX<139>                             | IOB              | OUTPUT     | LVCMOS25
|        | 12        | SLOW |                |           |          |
```

```
| QX<140>                            | IOB              | OUTPUT     | LVCMOS25
|        | 12        | SLOW |           |          |          |
| QX<141>                            | IOB              | OUTPUT     | LVCMOS25
|        | 12        | SLOW |           |          |          |
| QX<142>                            | IOB              | OUTPUT     | LVCMOS25
|        | 12        | SLOW |           |          |          |
| QX<143>                            | IOB              | OUTPUT     | LVCMOS25
|        | 12        | SLOW |           |          |          |
| QX<144>                            | IOB              | OUTPUT     | LVCMOS25
|        | 12        | SLOW |           |          |          |
| QX<145>                            | IOB              | OUTPUT     | LVCMOS25
|        | 12        | SLOW |           |          |          |
| QX<146>                            | IOB              | OUTPUT     | LVCMOS25
|        | 12        | SLOW |           |          |          |
| QX<147>                            | IOB              | OUTPUT     | LVCMOS25
|        | 12        | SLOW |           |          |          |
| QX<148>                            | IOB              | OUTPUT     | LVCMOS25
|        | 12        | SLOW |           |          |          |
| QX<149>                            | IOB              | OUTPUT     | LVCMOS25
|        | 12        | SLOW |           |          |          |
| QX<150>                            | IOB              | OUTPUT     | LVCMOS25
|        | 12        | SLOW |           |          |          |
| QX<151>                            | IOB              | OUTPUT     | LVCMOS25
|        | 12        | SLOW |           |          |          |
| QX<152>                            | IOB              | OUTPUT     | LVCMOS25
|        | 12        | SLOW |           |          |          |
| QX<153>                            | IOB              | OUTPUT     | LVCMOS25
|        | 12        | SLOW |           |          |          |
| QX<154>                            | IOB              | OUTPUT     | LVCMOS25
|        | 12        | SLOW |           |          |          |
| QX<155>                            | IOB              | OUTPUT     | LVCMOS25
|        | 12        | SLOW |           |          |          |
| QX<156>                            | IOB              | OUTPUT     | LVCMOS25
|        | 12        | SLOW |           |          |          |
| QX<157>                            | IOB              | OUTPUT     | LVCMOS25
|        | 12        | SLOW |           |          |          |
| QX<158>                            | IOB              | OUTPUT     | LVCMOS25
|        | 12        | SLOW |           |          |          |
| QX<159>                            | IOB              | OUTPUT     | LVCMOS25
|        | 12        | SLOW |           |          |          |
| QX<160>                            | IOB              | OUTPUT     | LVCMOS25
|        | 12        | SLOW |           |          |          |
| QX<161>                            | IOB              | OUTPUT     | LVCMOS25
|        | 12        | SLOW |           |          |          |
| QX<162>                            | IOB              | OUTPUT     | LVCMOS25
|        | 12        | SLOW |           |          |          |
| QY<0>                              | IOB              | OUTPUT     | LVCMOS25
|        | 12        | SLOW |           |          |          |
| QY<1>                              | IOB              | OUTPUT     | LVCMOS25
|        | 12        | SLOW |           |          |          |
| QY<2>                              | IOB              | OUTPUT     | LVCMOS25
|        | 12        | SLOW |           |          |          |
| QY<3>                              | IOB              | OUTPUT     | LVCMOS25
|        | 12        | SLOW |           |          |          |
| QY<4>                              | IOB              | OUTPUT     | LVCMOS25
|        | 12        | SLOW |           |          |          |
| QY<5>                              | IOB              | OUTPUT     | LVCMOS25
|        | 12        | SLOW |           |          |          |
| QY<6>                              | IOB              | OUTPUT     | LVCMOS25
|        | 12        | SLOW |           |          |          |
| QY<7>                              | IOB              | OUTPUT     | LVCMOS25
|        | 12        | SLOW |           |          |          |
| QY<8>                              | IOB              | OUTPUT     | LVCMOS25
|        | 12        | SLOW |           |          |          |
| QY<9>                              | IOB              | OUTPUT     | LVCMOS25
|        | 12        | SLOW |           |          |          |
| QY<10>                             | IOB              | OUTPUT     | LVCMOS25
|        | 12        | SLOW |           |          |          |
| QY<11>                             | IOB              | OUTPUT     | LVCMOS25
|        | 12        | SLOW |           |          |          |
| QY<12>                             | IOB              | OUTPUT     | LVCMOS25
```

```
|        | 12        | SLOW |               |          |        |
| QY<13>                              | IOB              | OUTPUT     | LVCMOS25
|        | 12        | SLOW |               |          |        |
| QY<14>                              | IOB              | OUTPUT     | LVCMOS25
|        | 12        | SLOW |               |          |        |
| QY<15>                              | IOB              | OUTPUT     | LVCMOS25
|        | 12        | SLOW |               |          |        |
| QY<16>                              | IOB              | OUTPUT     | LVCMOS25
|        | 12        | SLOW |               |          |        |
| QY<17>                              | IOB              | OUTPUT     | LVCMOS25
|        | 12        | SLOW |               |          |        |
| QY<18>                              | IOB              | OUTPUT     | LVCMOS25
|        | 12        | SLOW |               |          |        |
| QY<19>                              | IOB              | OUTPUT     | LVCMOS25
|        | 12        | SLOW |               |          |        |
| QY<20>                              | IOB              | OUTPUT     | LVCMOS25
|        | 12        | SLOW |               |          |        |
| QY<21>                              | IOB              | OUTPUT     | LVCMOS25
|        | 12        | SLOW |               |          |        |
| QY<22>                              | IOB              | OUTPUT     | LVCMOS25
|        | 12        | SLOW |               |          |        |
| QY<23>                              | IOB              | OUTPUT     | LVCMOS25
|        | 12        | SLOW |               |          |        |
| QY<24>                              | IOB              | OUTPUT     | LVCMOS25
|        | 12        | SLOW |               |          |        |
| QY<25>                              | IOB              | OUTPUT     | LVCMOS25
|        | 12        | SLOW |               |          |        |
| QY<26>                              | IOB              | OUTPUT     | LVCMOS25
|        | 12        | SLOW |               |          |        |
| QY<27>                              | IOB              | OUTPUT     | LVCMOS25
|        | 12        | SLOW |               |          |        |
| QY<28>                              | IOB              | OUTPUT     | LVCMOS25
|        | 12        | SLOW |               |          |        |
| QY<29>                              | IOB              | OUTPUT     | LVCMOS25
|        | 12        | SLOW |               |          |        |
| QY<30>                              | IOB              | OUTPUT     | LVCMOS25
|        | 12        | SLOW |               |          |        |
| QY<31>                              | IOB              | OUTPUT     | LVCMOS25
|        | 12        | SLOW |               |          |        |
| QY<32>                              | IOB              | OUTPUT     | LVCMOS25
|        | 12        | SLOW |               |          |        |
| QY<33>                              | IOB              | OUTPUT     | LVCMOS25
|        | 12        | SLOW |               |          |        |
| QY<34>                              | IOB              | OUTPUT     | LVCMOS25
|        | 12        | SLOW |               |          |        |
| QY<35>                              | IOB              | OUTPUT     | LVCMOS25
|        | 12        | SLOW |               |          |        |
| QY<36>                              | IOB              | OUTPUT     | LVCMOS25
|        | 12        | SLOW |               |          |        |
| QY<37>                              | IOB              | OUTPUT     | LVCMOS25
|        | 12        | SLOW |               |          |        |
| QY<38>                              | IOB              | OUTPUT     | LVCMOS25
|        | 12        | SLOW |               |          |        |
| QY<39>                              | IOB              | OUTPUT     | LVCMOS25
|        | 12        | SLOW |               |          |        |
| QY<40>                              | IOB              | OUTPUT     | LVCMOS25
|        | 12        | SLOW |               |          |        |
| QY<41>                              | IOB              | OUTPUT     | LVCMOS25
|        | 12        | SLOW |               |          |        |
| QY<42>                              | IOB              | OUTPUT     | LVCMOS25
|        | 12        | SLOW |               |          |        |
| QY<43>                              | IOB              | OUTPUT     | LVCMOS25
|        | 12        | SLOW |               |          |        |
| QY<44>                              | IOB              | OUTPUT     | LVCMOS25
|        | 12        | SLOW |               |          |        |
| QY<45>                              | IOB              | OUTPUT     | LVCMOS25
|        | 12        | SLOW |               |          |        |
| QY<46>                              | IOB              | OUTPUT     | LVCMOS25
|        | 12        | SLOW |               |          |        |
| QY<47>                              | IOB              | OUTPUT     | LVCMOS25
|        | 12        | SLOW |               |          |        |
```

```
| QY<48>                                  | IOB                 | OUTPUT      | LVCMOS25
|        | 12         | SLOW |                       |           |           |
| QY<49>                                  | IOB                 | OUTPUT      | LVCMOS25
|        | 12         | SLOW |                       |           |           |
| QY<50>                                  | IOB                 | OUTPUT      | LVCMOS25
|        | 12         | SLOW |                       |           |           |
| QY<51>                                  | IOB                 | OUTPUT      | LVCMOS25
|        | 12         | SLOW |                       |           |           |
| QY<52>                                  | IOB                 | OUTPUT      | LVCMOS25
|        | 12         | SLOW |                       |           |           |
| QY<53>                                  | IOB                 | OUTPUT      | LVCMOS25
|        | 12         | SLOW |                       |           |           |
| QY<54>                                  | IOB                 | OUTPUT      | LVCMOS25
|        | 12         | SLOW |                       |           |           |
| QY<55>                                  | IOB                 | OUTPUT      | LVCMOS25
|        | 12         | SLOW |                       |           |           |
| QY<56>                                  | IOB                 | OUTPUT      | LVCMOS25
|        | 12         | SLOW |                       |           |           |
| QY<57>                                  | IOB                 | OUTPUT      | LVCMOS25
|        | 12         | SLOW |                       |           |           |
| QY<58>                                  | IOB                 | OUTPUT      | LVCMOS25
|        | 12         | SLOW |                       |           |           |
| QY<59>                                  | IOB                 | OUTPUT      | LVCMOS25
|        | 12         | SLOW |                       |           |           |
| QY<60>                                  | IOB                 | OUTPUT      | LVCMOS25
|        | 12         | SLOW |                       |           |           |
| QY<61>                                  | IOB                 | OUTPUT      | LVCMOS25
|        | 12         | SLOW |                       |           |           |
| QY<62>                                  | IOB                 | OUTPUT      | LVCMOS25
|        | 12         | SLOW |                       |           |           |
| QY<63>                                  | IOB                 | OUTPUT      | LVCMOS25
|        | 12         | SLOW |                       |           |           |
| QY<64>                                  | IOB                 | OUTPUT      | LVCMOS25
|        | 12         | SLOW |                       |           |           |
| QY<65>                                  | IOB                 | OUTPUT      | LVCMOS25
|        | 12         | SLOW |                       |           |           |
| QY<66>                                  | IOB                 | OUTPUT      | LVCMOS25
|        | 12         | SLOW |                       |           |           |
| QY<67>                                  | IOB                 | OUTPUT      | LVCMOS25
|        | 12         | SLOW |                       |           |           |
| QY<68>                                  | IOB                 | OUTPUT      | LVCMOS25
|        | 12         | SLOW |                       |           |           |
| QY<69>                                  | IOB                 | OUTPUT      | LVCMOS25
|        | 12         | SLOW |                       |           |           |
| QY<70>                                  | IOB                 | OUTPUT      | LVCMOS25
|        | 12         | SLOW |                       |           |           |
| QY<71>                                  | IOB                 | OUTPUT      | LVCMOS25
|        | 12         | SLOW |                       |           |           |
| QY<72>                                  | IOB                 | OUTPUT      | LVCMOS25
|        | 12         | SLOW |                       |           |           |
| QY<73>                                  | IOB                 | OUTPUT      | LVCMOS25
|        | 12         | SLOW |                       |           |           |
| QY<74>                                  | IOB                 | OUTPUT      | LVCMOS25
|        | 12         | SLOW |                       |           |           |
| QY<75>                                  | IOB                 | OUTPUT      | LVCMOS25
|        | 12         | SLOW |                       |           |           |
| QY<76>                                  | IOB                 | OUTPUT      | LVCMOS25
|        | 12         | SLOW |                       |           |           |
| QY<77>                                  | IOB                 | OUTPUT      | LVCMOS25
|        | 12         | SLOW |                       |           |           |
| QY<78>                                  | IOB                 | OUTPUT      | LVCMOS25
|        | 12         | SLOW |                       |           |           |
| QY<79>                                  | IOB                 | OUTPUT      | LVCMOS25
|        | 12         | SLOW |                       |           |           |
| QY<80>                                  | IOB                 | OUTPUT      | LVCMOS25
|        | 12         | SLOW |                       |           |           |
| QY<81>                                  | IOB                 | OUTPUT      | LVCMOS25
|        | 12         | SLOW |                       |           |           |
| QY<82>                                  | IOB                 | OUTPUT      | LVCMOS25
|        | 12         | SLOW |                       |           |           |
| QY<83>                                  | IOB                 | OUTPUT      | LVCMOS25
```

```
|       | 12       | SLOW |              |          |          |
| QY<84>                             | IOB          | OUTPUT    | LVCMOS25
|       | 12       | SLOW |              |          |          |
| QY<85>                             | IOB          | OUTPUT    | LVCMOS25
|       | 12       | SLOW |              |          |          |
| QY<86>                             | IOB          | OUTPUT    | LVCMOS25
|       | 12       | SLOW |              |          |          |
| QY<87>                             | IOB          | OUTPUT    | LVCMOS25
|       | 12       | SLOW |              |          |          |
| QY<88>                             | IOB          | OUTPUT    | LVCMOS25
|       | 12       | SLOW |              |          |          |
| QY<89>                             | IOB          | OUTPUT    | LVCMOS25
|       | 12       | SLOW |              |          |          |
| QY<90>                             | IOB          | OUTPUT    | LVCMOS25
|       | 12       | SLOW |              |          |          |
| QY<91>                             | IOB          | OUTPUT    | LVCMOS25
|       | 12       | SLOW |              |          |          |
| QY<92>                             | IOB          | OUTPUT    | LVCMOS25
|       | 12       | SLOW |              |          |          |
| QY<93>                             | IOB          | OUTPUT    | LVCMOS25
|       | 12       | SLOW |              |          |          |
| QY<94>                             | IOB          | OUTPUT    | LVCMOS25
|       | 12       | SLOW |              |          |          |
| QY<95>                             | IOB          | OUTPUT    | LVCMOS25
|       | 12       | SLOW |              |          |          |
| QY<96>                             | IOB          | OUTPUT    | LVCMOS25
|       | 12       | SLOW |              |          |          |
| QY<97>                             | IOB          | OUTPUT    | LVCMOS25
|       | 12       | SLOW |              |          |          |
| QY<98>                             | IOB          | OUTPUT    | LVCMOS25
|       | 12       | SLOW |              |          |          |
| QY<99>                             | IOB          | OUTPUT    | LVCMOS25
|       | 12       | SLOW |              |          |          |
| QY<100>                            | IOB          | OUTPUT    | LVCMOS25
|       | 12       | SLOW |              |          |          |
| QY<101>                            | IOB          | OUTPUT    | LVCMOS25
|       | 12       | SLOW |              |          |          |
| QY<102>                            | IOB          | OUTPUT    | LVCMOS25
|       | 12       | SLOW |              |          |          |
| QY<103>                            | IOB          | OUTPUT    | LVCMOS25
|       | 12       | SLOW |              |          |          |
| QY<104>                            | IOB          | OUTPUT    | LVCMOS25
|       | 12       | SLOW |              |          |          |
| QY<105>                            | IOB          | OUTPUT    | LVCMOS25
|       | 12       | SLOW |              |          |          |
| QY<106>                            | IOB          | OUTPUT    | LVCMOS25
|       | 12       | SLOW |              |          |          |
| QY<107>                            | IOB          | OUTPUT    | LVCMOS25
|       | 12       | SLOW |              |          |          |
| QY<108>                            | IOB          | OUTPUT    | LVCMOS25
|       | 12       | SLOW |              |          |          |
| QY<109>                            | IOB          | OUTPUT    | LVCMOS25
|       | 12       | SLOW |              |          |          |
| QY<110>                            | IOB          | OUTPUT    | LVCMOS25
|       | 12       | SLOW |              |          |          |
| QY<111>                            | IOB          | OUTPUT    | LVCMOS25
|       | 12       | SLOW |              |          |          |
| QY<112>                            | IOB          | OUTPUT    | LVCMOS25
|       | 12       | SLOW |              |          |          |
| QY<113>                            | IOB          | OUTPUT    | LVCMOS25
|       | 12       | SLOW |              |          |          |
| QY<114>                            | IOB          | OUTPUT    | LVCMOS25
|       | 12       | SLOW |              |          |          |
| QY<115>                            | IOB          | OUTPUT    | LVCMOS25
|       | 12       | SLOW |              |          |          |
| QY<116>                            | IOB          | OUTPUT    | LVCMOS25
|       | 12       | SLOW |              |          |          |
| QY<117>                            | IOB          | OUTPUT    | LVCMOS25
|       | 12       | SLOW |              |          |          |
| QY<118>                            | IOB          | OUTPUT    | LVCMOS25
|       | 12       | SLOW |              |          |          |
```

```
| QY<119>                            | IOB                  | OUTPUT      | LVCMOS25
|        | 12          | SLOW |             |            |            |
| QY<120>                            | IOB                  | OUTPUT      | LVCMOS25
|        | 12          | SLOW |             |            |            |
| QY<121>                            | IOB                  | OUTPUT      | LVCMOS25
|        | 12          | SLOW |             |            |            |
| QY<122>                            | IOB                  | OUTPUT      | LVCMOS25
|        | 12          | SLOW |             |            |            |
| QY<123>                            | IOB                  | OUTPUT      | LVCMOS25
|        | 12          | SLOW |             |            |            |
| QY<124>                            | IOB                  | OUTPUT      | LVCMOS25
|        | 12          | SLOW |             |            |            |
| QY<125>                            | IOB                  | OUTPUT      | LVCMOS25
|        | 12          | SLOW |             |            |            |
| QY<126>                            | IOB                  | OUTPUT      | LVCMOS25
|        | 12          | SLOW |             |            |            |
| QY<127>                            | IOB                  | OUTPUT      | LVCMOS25
|        | 12          | SLOW |             |            |            |
| QY<128>                            | IOB                  | OUTPUT      | LVCMOS25
|        | 12          | SLOW |             |            |            |
| QY<129>                            | IOB                  | OUTPUT      | LVCMOS25
|        | 12          | SLOW |             |            |            |
| QY<130>                            | IOB                  | OUTPUT      | LVCMOS25
|        | 12          | SLOW |             |            |            |
| QY<131>                            | IOB                  | OUTPUT      | LVCMOS25
|        | 12          | SLOW |             |            |            |
| QY<132>                            | IOB                  | OUTPUT      | LVCMOS25
|        | 12          | SLOW |             |            |            |
| QY<133>                            | IOB                  | OUTPUT      | LVCMOS25
|        | 12          | SLOW |             |            |            |
| QY<134>                            | IOB                  | OUTPUT      | LVCMOS25
|        | 12          | SLOW |             |            |            |
| QY<135>                            | IOB                  | OUTPUT      | LVCMOS25
|        | 12          | SLOW |             |            |            |
| QY<136>                            | IOB                  | OUTPUT      | LVCMOS25
|        | 12          | SLOW |             |            |            |
| QY<137>                            | IOB                  | OUTPUT      | LVCMOS25
|        | 12          | SLOW |             |            |            |
| QY<138>                            | IOB                  | OUTPUT      | LVCMOS25
|        | 12          | SLOW |             |            |            |
| QY<139>                            | IOB                  | OUTPUT      | LVCMOS25
|        | 12          | SLOW |             |            |            |
| QY<140>                            | IOB                  | OUTPUT      | LVCMOS25
|        | 12          | SLOW |             |            |            |
| QY<141>                            | IOB                  | OUTPUT      | LVCMOS25
|        | 12          | SLOW |             |            |            |
| QY<142>                            | IOB                  | OUTPUT      | LVCMOS25
|        | 12          | SLOW |             |            |            |
| QY<143>                            | IOB                  | OUTPUT      | LVCMOS25
|        | 12          | SLOW |             |            |            |
| QY<144>                            | IOB                  | OUTPUT      | LVCMOS25
|        | 12          | SLOW |             |            |            |
| QY<145>                            | IOB                  | OUTPUT      | LVCMOS25
|        | 12          | SLOW |             |            |            |
| QY<146>                            | IOB                  | OUTPUT      | LVCMOS25
|        | 12          | SLOW |             |            |            |
| QY<147>                            | IOB                  | OUTPUT      | LVCMOS25
|        | 12          | SLOW |             |            |            |
| QY<148>                            | IOB                  | OUTPUT      | LVCMOS25
|        | 12          | SLOW |             |            |            |
| QY<149>                            | IOB                  | OUTPUT      | LVCMOS25
|        | 12          | SLOW |             |            |            |
| QY<150>                            | IOB                  | OUTPUT      | LVCMOS25
|        | 12          | SLOW |             |            |            |
| QY<151>                            | IOB                  | OUTPUT      | LVCMOS25
|        | 12          | SLOW |             |            |            |
| QY<152>                            | IOB                  | OUTPUT      | LVCMOS25
|        | 12          | SLOW |             |            |            |
| QY<153>                            | IOB                  | OUTPUT      | LVCMOS25
|        | 12          | SLOW |             |            |            |
| QY<154>                            | IOB                  | OUTPUT      | LVCMOS25
```

```
|        | 12         | SLOW |                       |          |          |
| QY<155>                                  | IOB                | OUTPUT     | LVCMOS25
|        | 12         | SLOW |                       |          |          |
| QY<156>                                  | IOB                | OUTPUT     | LVCMOS25
|        | 12         | SLOW |                       |          |          |
| QY<157>                                  | IOB                | OUTPUT     | LVCMOS25
|        | 12         | SLOW |                       |          |          |
| QY<158>                                  | IOB                | OUTPUT     | LVCMOS25
|        | 12         | SLOW |                       |          |          |
| QY<159>                                  | IOB                | OUTPUT     | LVCMOS25
|        | 12         | SLOW |                       |          |          |
| QY<160>                                  | IOB                | OUTPUT     | LVCMOS25
|        | 12         | SLOW |                       |          |          |
| QY<161>                                  | IOB                | OUTPUT     | LVCMOS25
|        | 12         | SLOW |                       |          |          |
| QY<162>                                  | IOB                | OUTPUT     | LVCMOS25
|        | 12         | SLOW |                       |          |          |
| QZ<0>                                    | IOB                | OUTPUT     | LVCMOS25
|        | 12         | SLOW |                       |          |          |
| QZ<1>                                    | IOB                | OUTPUT     | LVCMOS25
|        | 12         | SLOW |                       |          |          |
| QZ<2>                                    | IOB                | OUTPUT     | LVCMOS25
|        | 12         | SLOW |                       |          |          |
| QZ<3>                                    | IOB                | OUTPUT     | LVCMOS25
|        | 12         | SLOW |                       |          |          |
| QZ<4>                                    | IOB                | OUTPUT     | LVCMOS25
|        | 12         | SLOW |                       |          |          |
| QZ<5>                                    | IOB                | OUTPUT     | LVCMOS25
|        | 12         | SLOW |                       |          |          |
| QZ<6>                                    | IOB                | OUTPUT     | LVCMOS25
|        | 12         | SLOW |                       |          |          |
| QZ<7>                                    | IOB                | OUTPUT     | LVCMOS25
|        | 12         | SLOW |                       |          |          |
| QZ<8>                                    | IOB                | OUTPUT     | LVCMOS25
|        | 12         | SLOW |                       |          |          |
| QZ<9>                                    | IOB                | OUTPUT     | LVCMOS25
|        | 12         | SLOW |                       |          |          |
| QZ<10>                                   | IOB                | OUTPUT     | LVCMOS25
|        | 12         | SLOW |                       |          |          |
| QZ<11>                                   | IOB                | OUTPUT     | LVCMOS25
|        | 12         | SLOW |                       |          |          |
| QZ<12>                                   | IOB                | OUTPUT     | LVCMOS25
|        | 12         | SLOW |                       |          |          |
| QZ<13>                                   | IOB                | OUTPUT     | LVCMOS25
|        | 12         | SLOW |                       |          |          |
| QZ<14>                                   | IOB                | OUTPUT     | LVCMOS25
|        | 12         | SLOW |                       |          |          |
| QZ<15>                                   | IOB                | OUTPUT     | LVCMOS25
|        | 12         | SLOW |                       |          |          |
| QZ<16>                                   | IOB                | OUTPUT     | LVCMOS25
|        | 12         | SLOW |                       |          |          |
| QZ<17>                                   | IOB                | OUTPUT     | LVCMOS25
|        | 12         | SLOW |                       |          |          |
| QZ<18>                                   | IOB                | OUTPUT     | LVCMOS25
|        | 12         | SLOW |                       |          |          |
| QZ<19>                                   | IOB                | OUTPUT     | LVCMOS25
|        | 12         | SLOW |                       |          |          |
| QZ<20>                                   | IOB                | OUTPUT     | LVCMOS25
|        | 12         | SLOW |                       |          |          |
| QZ<21>                                   | IOB                | OUTPUT     | LVCMOS25
|        | 12         | SLOW |                       |          |          |
| QZ<22>                                   | IOB                | OUTPUT     | LVCMOS25
|        | 12         | SLOW |                       |          |          |
| QZ<23>                                   | IOB                | OUTPUT     | LVCMOS25
|        | 12         | SLOW |                       |          |          |
| QZ<24>                                   | IOB                | OUTPUT     | LVCMOS25
|        | 12         | SLOW |                       |          |          |
| QZ<25>                                   | IOB                | OUTPUT     | LVCMOS25
|        | 12         | SLOW |                       |          |          |
| QZ<26>                                   | IOB                | OUTPUT     | LVCMOS25
|        | 12         | SLOW |                       |          |          |
```

```
| QZ<27>                             | IOB              | OUTPUT    | LVCMOS25
|       | 12         | SLOW |                |           |          |
| QZ<28>                             | IOB              | OUTPUT    | LVCMOS25
|       | 12         | SLOW |                |           |          |
| QZ<29>                             | IOB              | OUTPUT    | LVCMOS25
|       | 12         | SLOW |                |           |          |
| QZ<30>                             | IOB              | OUTPUT    | LVCMOS25
|       | 12         | SLOW |                |           |          |
| QZ<31>                             | IOB              | OUTPUT    | LVCMOS25
|       | 12         | SLOW |                |           |          |
| QZ<32>                             | IOB              | OUTPUT    | LVCMOS25
|       | 12         | SLOW |                |           |          |
| QZ<33>                             | IOB              | OUTPUT    | LVCMOS25
|       | 12         | SLOW |                |           |          |
| QZ<34>                             | IOB              | OUTPUT    | LVCMOS25
|       | 12         | SLOW |                |           |          |
| QZ<35>                             | IOB              | OUTPUT    | LVCMOS25
|       | 12         | SLOW |                |           |          |
| QZ<36>                             | IOB              | OUTPUT    | LVCMOS25
|       | 12         | SLOW |                |           |          |
| QZ<37>                             | IOB              | OUTPUT    | LVCMOS25
|       | 12         | SLOW |                |           |          |
| QZ<38>                             | IOB              | OUTPUT    | LVCMOS25
|       | 12         | SLOW |                |           |          |
| QZ<39>                             | IOB              | OUTPUT    | LVCMOS25
|       | 12         | SLOW |                |           |          |
| QZ<40>                             | IOB              | OUTPUT    | LVCMOS25
|       | 12         | SLOW |                |           |          |
| QZ<41>                             | IOB              | OUTPUT    | LVCMOS25
|       | 12         | SLOW |                |           |          |
| QZ<42>                             | IOB              | OUTPUT    | LVCMOS25
|       | 12         | SLOW |                |           |          |
| QZ<43>                             | IOB              | OUTPUT    | LVCMOS25
|       | 12         | SLOW |                |           |          |
| QZ<44>                             | IOB              | OUTPUT    | LVCMOS25
|       | 12         | SLOW |                |           |          |
| QZ<45>                             | IOB              | OUTPUT    | LVCMOS25
|       | 12         | SLOW |                |           |          |
| QZ<46>                             | IOB              | OUTPUT    | LVCMOS25
|       | 12         | SLOW |                |           |          |
| QZ<47>                             | IOB              | OUTPUT    | LVCMOS25
|       | 12         | SLOW |                |           |          |
| QZ<48>                             | IOB              | OUTPUT    | LVCMOS25
|       | 12         | SLOW |                |           |          |
| QZ<49>                             | IOB              | OUTPUT    | LVCMOS25
|       | 12         | SLOW |                |           |          |
| QZ<50>                             | IOB              | OUTPUT    | LVCMOS25
|       | 12         | SLOW |                |           |          |
| QZ<51>                             | IOB              | OUTPUT    | LVCMOS25
|       | 12         | SLOW |                |           |          |
| QZ<52>                             | IOB              | OUTPUT    | LVCMOS25
|       | 12         | SLOW |                |           |          |
| QZ<53>                             | IOB              | OUTPUT    | LVCMOS25
|       | 12         | SLOW |                |           |          |
| QZ<54>                             | IOB              | OUTPUT    | LVCMOS25
|       | 12         | SLOW |                |           |          |
| QZ<55>                             | IOB              | OUTPUT    | LVCMOS25
|       | 12         | SLOW |                |           |          |
| QZ<56>                             | IOB              | OUTPUT    | LVCMOS25
|       | 12         | SLOW |                |           |          |
| QZ<57>                             | IOB              | OUTPUT    | LVCMOS25
|       | 12         | SLOW |                |           |          |
| QZ<58>                             | IOB              | OUTPUT    | LVCMOS25
|       | 12         | SLOW |                |           |          |
| QZ<59>                             | IOB              | OUTPUT    | LVCMOS25
|       | 12         | SLOW |                |           |          |
| QZ<60>                             | IOB              | OUTPUT    | LVCMOS25
|       | 12         | SLOW |                |           |          |
| QZ<61>                             | IOB              | OUTPUT    | LVCMOS25
|       | 12         | SLOW |                |           |          |
| QZ<62>                             | IOB              | OUTPUT    | LVCMOS25
```

```
|        | 12        | SLOW |              |           |
| QZ<63>                          | IOB           | OUTPUT    | LVCMOS25
|        | 12        | SLOW |              |           |
| QZ<64>                          | IOB           | OUTPUT    | LVCMOS25
|        | 12        | SLOW |              |           |
| QZ<65>                          | IOB           | OUTPUT    | LVCMOS25
|        | 12        | SLOW |              |           |
| QZ<66>                          | IOB           | OUTPUT    | LVCMOS25
|        | 12        | SLOW |              |           |
| QZ<67>                          | IOB           | OUTPUT    | LVCMOS25
|        | 12        | SLOW |              |           |
| QZ<68>                          | IOB           | OUTPUT    | LVCMOS25
|        | 12        | SLOW |              |           |
| QZ<69>                          | IOB           | OUTPUT    | LVCMOS25
|        | 12        | SLOW |              |           |
| QZ<70>                          | IOB           | OUTPUT    | LVCMOS25
|        | 12        | SLOW |              |           |
| QZ<71>                          | IOB           | OUTPUT    | LVCMOS25
|        | 12        | SLOW |              |           |
| QZ<72>                          | IOB           | OUTPUT    | LVCMOS25
|        | 12        | SLOW |              |           |
| QZ<73>                          | IOB           | OUTPUT    | LVCMOS25
|        | 12        | SLOW |              |           |
| QZ<74>                          | IOB           | OUTPUT    | LVCMOS25
|        | 12        | SLOW |              |           |
| QZ<75>                          | IOB           | OUTPUT    | LVCMOS25
|        | 12        | SLOW |              |           |
| QZ<76>                          | IOB           | OUTPUT    | LVCMOS25
|        | 12        | SLOW |              |           |
| QZ<77>                          | IOB           | OUTPUT    | LVCMOS25
|        | 12        | SLOW |              |           |
| QZ<78>                          | IOB           | OUTPUT    | LVCMOS25
|        | 12        | SLOW |              |           |
| QZ<79>                          | IOB           | OUTPUT    | LVCMOS25
|        | 12        | SLOW |              |           |
| QZ<80>                          | IOB           | OUTPUT    | LVCMOS25
|        | 12        | SLOW |              |           |
| QZ<81>                          | IOB           | OUTPUT    | LVCMOS25
|        | 12        | SLOW |              |           |
| QZ<82>                          | IOB           | OUTPUT    | LVCMOS25
|        | 12        | SLOW |              |           |
| QZ<83>                          | IOB           | OUTPUT    | LVCMOS25
|        | 12        | SLOW |              |           |
| QZ<84>                          | IOB           | OUTPUT    | LVCMOS25
|        | 12        | SLOW |              |           |
| QZ<85>                          | IOB           | OUTPUT    | LVCMOS25
|        | 12        | SLOW |              |           |
| QZ<86>                          | IOB           | OUTPUT    | LVCMOS25
|        | 12        | SLOW |              |           |
| QZ<87>                          | IOB           | OUTPUT    | LVCMOS25
|        | 12        | SLOW |              |           |
| QZ<88>                          | IOB           | OUTPUT    | LVCMOS25
|        | 12        | SLOW |              |           |
| QZ<89>                          | IOB           | OUTPUT    | LVCMOS25
|        | 12        | SLOW |              |           |
| QZ<90>                          | IOB           | OUTPUT    | LVCMOS25
|        | 12        | SLOW |              |           |
| QZ<91>                          | IOB           | OUTPUT    | LVCMOS25
|        | 12        | SLOW |              |           |
| QZ<92>                          | IOB           | OUTPUT    | LVCMOS25
|        | 12        | SLOW |              |           |
| QZ<93>                          | IOB           | OUTPUT    | LVCMOS25
|        | 12        | SLOW |              |           |
| QZ<94>                          | IOB           | OUTPUT    | LVCMOS25
|        | 12        | SLOW |              |           |
| QZ<95>                          | IOB           | OUTPUT    | LVCMOS25
|        | 12        | SLOW |              |           |
| QZ<96>                          | IOB           | OUTPUT    | LVCMOS25
|        | 12        | SLOW |              |           |
| QZ<97>                          | IOB           | OUTPUT    | LVCMOS25
|        | 12        | SLOW |              |           |
```

```
| QZ<98>                                    | IOB                  | OUTPUT     | LVCMOS25
|        | 12        | SLOW |                 |             |            |
| QZ<99>                                    | IOB                  | OUTPUT     | LVCMOS25
|        | 12        | SLOW |                 |             |            |
| QZ<100>                                   | IOB                  | OUTPUT     | LVCMOS25
|        | 12        | SLOW |                 |             |            |
| QZ<101>                                   | IOB                  | OUTPUT     | LVCMOS25
|        | 12        | SLOW |                 |             |            |
| QZ<102>                                   | IOB                  | OUTPUT     | LVCMOS25
|        | 12        | SLOW |                 |             |            |
| QZ<103>                                   | IOB                  | OUTPUT     | LVCMOS25
|        | 12        | SLOW |                 |             |            |
| QZ<104>                                   | IOB                  | OUTPUT     | LVCMOS25
|        | 12        | SLOW |                 |             |            |
| QZ<105>                                   | IOB                  | OUTPUT     | LVCMOS25
|        | 12        | SLOW |                 |             |            |
| QZ<106>                                   | IOB                  | OUTPUT     | LVCMOS25
|        | 12        | SLOW |                 |             |            |
| QZ<107>                                   | IOB                  | OUTPUT     | LVCMOS25
|        | 12        | SLOW |                 |             |            |
| QZ<108>                                   | IOB                  | OUTPUT     | LVCMOS25
|        | 12        | SLOW |                 |             |            |
| QZ<109>                                   | IOB                  | OUTPUT     | LVCMOS25
|        | 12        | SLOW |                 |             |            |
| QZ<110>                                   | IOB                  | OUTPUT     | LVCMOS25
|        | 12        | SLOW |                 |             |            |
| QZ<111>                                   | IOB                  | OUTPUT     | LVCMOS25
|        | 12        | SLOW |                 |             |            |
| QZ<112>                                   | IOB                  | OUTPUT     | LVCMOS25
|        | 12        | SLOW |                 |             |            |
| QZ<113>                                   | IOB                  | OUTPUT     | LVCMOS25
|        | 12        | SLOW |                 |             |            |
| QZ<114>                                   | IOB                  | OUTPUT     | LVCMOS25
|        | 12        | SLOW |                 |             |            |
| QZ<115>                                   | IOB                  | OUTPUT     | LVCMOS25
|        | 12        | SLOW |                 |             |            |
| QZ<116>                                   | IOB                  | OUTPUT     | LVCMOS25
|        | 12        | SLOW |                 |             |            |
| QZ<117>                                   | IOB                  | OUTPUT     | LVCMOS25
|        | 12        | SLOW |                 |             |            |
| QZ<118>                                   | IOB                  | OUTPUT     | LVCMOS25
|        | 12        | SLOW |                 |             |            |
| QZ<119>                                   | IOB                  | OUTPUT     | LVCMOS25
|        | 12        | SLOW |                 |             |            |
| QZ<120>                                   | IOB                  | OUTPUT     | LVCMOS25
|        | 12        | SLOW |                 |             |            |
| QZ<121>                                   | IOB                  | OUTPUT     | LVCMOS25
|        | 12        | SLOW |                 |             |            |
| QZ<122>                                   | IOB                  | OUTPUT     | LVCMOS25
|        | 12        | SLOW |                 |             |            |
| QZ<123>                                   | IOB                  | OUTPUT     | LVCMOS25
|        | 12        | SLOW |                 |             |            |
| QZ<124>                                   | IOB                  | OUTPUT     | LVCMOS25
|        | 12        | SLOW |                 |             |            |
| QZ<125>                                   | IOB                  | OUTPUT     | LVCMOS25
|        | 12        | SLOW |                 |             |            |
| QZ<126>                                   | IOB                  | OUTPUT     | LVCMOS25
|        | 12        | SLOW |                 |             |            |
| QZ<127>                                   | IOB                  | OUTPUT     | LVCMOS25
|        | 12        | SLOW |                 |             |            |
| QZ<128>                                   | IOB                  | OUTPUT     | LVCMOS25
|        | 12        | SLOW |                 |             |            |
| QZ<129>                                   | IOB                  | OUTPUT     | LVCMOS25
|        | 12        | SLOW |                 |             |            |
| QZ<130>                                   | IOB                  | OUTPUT     | LVCMOS25
|        | 12        | SLOW |                 |             |            |
| QZ<131>                                   | IOB                  | OUTPUT     | LVCMOS25
|        | 12        | SLOW |                 |             |            |
| QZ<132>                                   | IOB                  | OUTPUT     | LVCMOS25
|        | 12        | SLOW |                 |             |            |
| QZ<133>                                   | IOB                  | OUTPUT     | LVCMOS25
```

```
|        | 12        | SLOW |                    |           |
| QZ<134>                              | IOB              | OUTPUT      | LVCMOS25
|        | 12        | SLOW |                    |           |
| QZ<135>                              | IOB              | OUTPUT      | LVCMOS25
|        | 12        | SLOW |                    |           |
| QZ<136>                              | IOB              | OUTPUT      | LVCMOS25
|        | 12        | SLOW |                    |           |
| QZ<137>                              | IOB              | OUTPUT      | LVCMOS25
|        | 12        | SLOW |                    |           |
| QZ<138>                              | IOB              | OUTPUT      | LVCMOS25
|        | 12        | SLOW |                    |           |
| QZ<139>                              | IOB              | OUTPUT      | LVCMOS25
|        | 12        | SLOW |                    |           |
| QZ<140>                              | IOB              | OUTPUT      | LVCMOS25
|        | 12        | SLOW |                    |           |
| QZ<141>                              | IOB              | OUTPUT      | LVCMOS25
|        | 12        | SLOW |                    |           |
| QZ<142>                              | IOB              | OUTPUT      | LVCMOS25
|        | 12        | SLOW |                    |           |
| QZ<143>                              | IOB              | OUTPUT      | LVCMOS25
|        | 12        | SLOW |                    |           |
| QZ<144>                              | IOB              | OUTPUT      | LVCMOS25
|        | 12        | SLOW |                    |           |
| QZ<145>                              | IOB              | OUTPUT      | LVCMOS25
|        | 12        | SLOW |                    |           |
| QZ<146>                              | IOB              | OUTPUT      | LVCMOS25
|        | 12        | SLOW |                    |           |
| QZ<147>                              | IOB              | OUTPUT      | LVCMOS25
|        | 12        | SLOW |                    |           |
| QZ<148>                              | IOB              | OUTPUT      | LVCMOS25
|        | 12        | SLOW |                    |           |
| QZ<149>                              | IOB              | OUTPUT      | LVCMOS25
|        | 12        | SLOW |                    |           |
| QZ<150>                              | IOB              | OUTPUT      | LVCMOS25
|        | 12        | SLOW |                    |           |
| QZ<151>                              | IOB              | OUTPUT      | LVCMOS25
|        | 12        | SLOW |                    |           |
| QZ<152>                              | IOB              | OUTPUT      | LVCMOS25
|        | 12        | SLOW |                    |           |
| QZ<153>                              | IOB              | OUTPUT      | LVCMOS25
|        | 12        | SLOW |                    |           |
| QZ<154>                              | IOB              | OUTPUT      | LVCMOS25
|        | 12        | SLOW |                    |           |
| QZ<155>                              | IOB              | OUTPUT      | LVCMOS25
|        | 12        | SLOW |                    |           |
| QZ<156>                              | IOB              | OUTPUT      | LVCMOS25
|        | 12        | SLOW |                    |           |
| QZ<157>                              | IOB              | OUTPUT      | LVCMOS25
|        | 12        | SLOW |                    |           |
| QZ<158>                              | IOB              | OUTPUT      | LVCMOS25
|        | 12        | SLOW |                    |           |
| QZ<159>                              | IOB              | OUTPUT      | LVCMOS25
|        | 12        | SLOW |                    |           |
| QZ<160>                              | IOB              | OUTPUT      | LVCMOS25
|        | 12        | SLOW |                    |           |
| QZ<161>                              | IOB              | OUTPUT      | LVCMOS25
|        | 12        | SLOW |                    |           |
| QZ<162>                              | IOB              | OUTPUT      | LVCMOS25
|        | 12        | SLOW |                    |           |
| clk                                  | IOB              | INPUT       | LVCMOS25
|        |           |      |                    |           |
| done                                 | IOB              | OUTPUT      | LVCMOS25
|        | 12        | SLOW |                    |           |
| reset                                | IOB              | INPUT       | LVCMOS25
|        |           |      |                    |           |
+-----------------------------------------------------------------------------------------------
--------------------------------------------------------------+

Section 7 - RPMs
----------------
```

```
Section 8 - Guide Report
------------------------
Guide not run on this design.

Section 9 - Area Group and Partition Summary
--------------------------------------------

Partition Implementation Status
-------------------------------

  No Partitions were found in this design.

-------------------------------

Area Group Information
----------------------

  No area groups were found in this design.

----------------------

Section 10 - Timing Report
--------------------------
A logic-level (pre-route) timing report can be generated by using Xilinx static
timing analysis tools, Timing Analyzer (GUI) or TRCE (command line), with the
mapped NCD and PCF files. Please note that this timing report will be generated
using estimated delay information. For accurate numbers, please generate a
timing report with the post Place and Route NCD file.

For more information about the Timing Analyzer, consult the Xilinx Timing
Analyzer Reference Manual; for more information about TRCE, consult the Xilinx
Command Line Tools User Guide "TRACE" chapter.

Section 11 - Configuration String Details
-----------------------------------------
Use the "-detail" map option to print out Configuration Strings

Section 12 - Control Set Information
------------------------------------
Use the "-detail" map option to print out Control Set Information.

Section 13 - Utilization by Hierarchy
-------------------------------------
Use the "-detail" map option to print out the Utilization by Hierarchy section.
```
