# Supplementary material for: Parallel point-multiplication architecture using combined group operations for high-speed cryptographic applications
Source: PLoS One. 2017 May 1;12(5):e0176214. doi: 10.1371/journal.pone.0176214 (PMC5411040; doi:10.1371/journal.pone.0176214)
Supplement: S1 Supporting Information — (ZIP) [file pone.0176214.s001.zip › S1 Supporting Information/S1 File13 Table2_[f].pdf]

```
Release 14.7 - xst P.20131013 (nt64)
Copyright (c) 1995-2013 Xilinx, Inc.  All rights reserved.
--> Parameter TMPDIR set to xst/projnav.tmp


Total REAL time to Xst completion: 0.00 secs
Total CPU time to Xst completion: 0.09 secs

--> Parameter xsthdpdir set to xst


Total REAL time to Xst completion: 0.00 secs
Total CPU time to Xst completion: 0.09 secs

--> Reading design: ECC_K_163_Jac.prj

TABLE OF CONTENTS
  1) Synthesis Options Summary
  2) HDL Parsing
  3) HDL Elaboration
  4) HDL Synthesis
       4.1) HDL Synthesis Report
  5) Advanced HDL Synthesis
       5.1) Advanced HDL Synthesis Report
  6) Low Level Synthesis
  7) Partition Report
  8) Design Summary
       8.1) Primitive and Black Box Usage
       8.2) Device utilization summary
       8.3) Partition Resource Summary
       8.4) Timing Report
            8.4.1) Clock Information
            8.4.2) Asynchronous Control Signals Information
            8.4.3) Timing Summary
            8.4.4) Timing Details
            8.4.5) Cross Clock Domains Report


=========================================================================
*                      Synthesis Options Summary                        *
=========================================================================
---- Source Parameters
Input File Name                    : "ECC_K_163_Jac.prj"
Ignore Synthesis Constraint File   : NO

---- Target Parameters
Output File Name                   : "ECC_K_163_Jac"
Output Format                      : NGC
Target Device                      : xc6vlx760-2-ff1760

---- Source Options
Top Module Name                    : ECC_K_163_Jac
Automatic FSM Extraction           : YES
FSM Encoding Algorithm             : Auto
Safe Implementation                : No
FSM Style                          : LUT
RAM Extraction                     : Yes
RAM Style                          : Auto
ROM Extraction                     : Yes
Shift Register Extraction          : YES
ROM Style                          : Auto
Resource Sharing                   : YES
Asynchronous To Synchronous        : NO
Shift Register Minimum Size        : 2
Use DSP Block                      : Auto
Automatic Register Balancing       : No

---- Target Options
LUT Combining                      : Auto
Reduce Control Sets                : Auto
Add IO Buffers                     : YES
```

```
Global Maximum Fanout              : 100000
Add Generic Clock Buffer(BUFG)     : 32
Register Duplication               : YES
Optimize Instantiated Primitives   : NO
Use Clock Enable                   : Auto
Use Synchronous Set                : Auto
Use Synchronous Reset              : Auto
Pack IO Registers into IOBs        : Auto
Equivalent register Removal        : YES

---- General Options
Optimization Goal                  : Speed
Optimization Effort                : 1
Power Reduction                    : NO
Keep Hierarchy                     : No
Netlist Hierarchy                  : As_Optimized
RTL Output                         : Yes
Global Optimization                : AllClockNets
Read Cores                         : YES
Write Timing Constraints           : NO
Cross Clock Analysis               : NO
Hierarchy Separator                : /
Bus Delimiter                      : <>
Case Specifier                     : Maintain
Slice Utilization Ratio            : 100
BRAM Utilization Ratio             : 100
DSP48 Utilization Ratio            : 100
Auto BRAM Packing                  : NO
Slice Utilization Ratio Delta      : 5

=========================================================================


=========================================================================
*                           HDL Parsing                                 *
=========================================================================
Parsing VHDL file "E:\Research doc_After_March_2014_to_Current)\My Researcg Related
documents_2\VHDL Code_ALL\Parallel_163_283_ALL\ECC_K_163_Jac_Parallel\ECC_package_BF.vhd"
into library work
Parsing package <ECC_package_BF>.
Parsing VHDL file "E:\Research doc_After_March_2014_to_Current)\My Researcg Related
documents_2\VHDL Code_ALL\Parallel_163_283_ALL\ECC_K_163_Jac_Parallel\pol_SQ.vhd" into
library work
Parsing entity <pol_SQ>.
Parsing architecture <arch_pol_SQ> of entity <pol_sq>.
Parsing VHDL file "E:\Research doc_After_March_2014_to_Current)\My Researcg Related
documents_2\VHDL Code_ALL\Parallel_163_283_ALL\ECC_K_163_Jac_Parallel\mult_k_163.vhd" into
library work
Parsing entity <pol_mult>.
Parsing architecture <arch_pol_mult> of entity <pol_mult>.
Parsing VHDL file "E:\Research doc_After_March_2014_to_Current)\My Researcg Related
documents_2\VHDL Code_ALL\Parallel_163_283_ALL\ECC_K_163_Jac_Parallel\PD_k_163_Jac.vhd" into
library work
Parsing entity <PD_K_163_Jac>.
Parsing architecture <arch_PD_K_163_Jac> of entity <pd_k_163_jac>.
Parsing VHDL file "E:\Research doc_After_March_2014_to_Current)\My Researcg Related
documents_2\VHDL Code_ALL\Parallel_163_283_ALL\ECC_K_163_Jac_Parallel\PA_k_163_Jac.vhd" into
library work
Parsing entity <PA_K_163_Jac>.
Parsing architecture <arch_PA_K_163_Jac> of entity <pa_k_163_jac>.
Parsing VHDL file "E:\Research doc_After_March_2014_to_Current)\My Researcg Related
documents_2\VHDL Code_ALL\Parallel_163_283_ALL\ECC_K_163_Jac_Parallel\ECC_K_163_Jac.vhd"
into library work
Parsing entity <ECC_K_163_Jac>.
Parsing architecture <arch_ECC_K_163_Jac> of entity <ecc_k_163_jac>.

=========================================================================
*                            HDL Elaboration                            *
=========================================================================

Elaborating entity <ECC_K_163_Jac> (architecture <arch_ECC_K_163_Jac>) from library <work>.
```

```
Elaborating entity <PD_K_163_Jac> (architecture <arch_PD_K_163_Jac>) from library <work>.

Elaborating entity <pol_SQ> (architecture <arch_pol_SQ>) from library <work>.

Elaborating entity <pol_mult> (architecture <arch_pol_mult>) from library <work>.

Elaborating entity <PA_K_163_Jac> (architecture <arch_PA_K_163_Jac>) from library <work>.

=========================================================================
*                            HDL Synthesis                              *
=========================================================================

Synthesizing Unit <ECC_K_163_Jac>.
    Related source file is "E:\Research doc_After_March_2014_to_Current)\My Researcg Related
    documents_2\VHDL Code_ALL\Parallel_163_283_ALL\ECC_K_163_Jac_Parallel\ECC_K_163_Jac.vhd".
    Found 163-bit register for signal <QX>.
    Found 163-bit register for signal <QY>.
    Found 163-bit register for signal <QZ>.
    Found 8-bit register for signal <count>.
    Found 1-bit register for signal <done>.
    Found 8-bit subtractor for signal <GND_6_o_GND_6_o_sub_6_OUT<7:0>> created at line 83.
    Found 1-bit 163-to-1 multiplexer for signal <count[7]_X_6_o_Mux_0_o> created at line 70.
    Summary:
    inferred   1 Adder/Subtractor(s).
    inferred 498 D-type flip-flop(s).
    inferred   2 Multiplexer(s).
Unit <ECC_K_163_Jac> synthesized.

Synthesizing Unit <PD_K_163_Jac>.
    Related source file is "E:\Research doc_After_March_2014_to_Current)\My Researcg Related
    documents_2\VHDL Code_ALL\Parallel_163_283_ALL\ECC_K_163_Jac_Parallel\PD_k_163_Jac.vhd".
    Summary:
Unit <PD_K_163_Jac> synthesized.

Synthesizing Unit <pol_SQ>.
    Related source file is "E:\Research doc_After_March_2014_to_Current)\My Researcg Related
    documents_2\VHDL Code_ALL\Parallel_163_283_ALL\ECC_K_163_Jac_Parallel\pol_SQ.vhd".
    Summary:
Unit <pol_SQ> synthesized.

Synthesizing Unit <pol_mult>.
    Related source file is "E:\Research doc_After_March_2014_to_Current)\My Researcg Related
    documents_2\VHDL Code_ALL\Parallel_163_283_ALL\ECC_K_163_Jac_Parallel\mult_k_163.vhd".
    Summary:
Unit <pol_mult> synthesized.

Synthesizing Unit <PA_K_163_Jac>.
    Related source file is "E:\Research doc_After_March_2014_to_Current)\My Researcg Related
    documents_2\VHDL Code_ALL\Parallel_163_283_ALL\ECC_K_163_Jac_Parallel\PA_k_163_Jac.vhd".
    Summary:
Unit <PA_K_163_Jac> synthesized.

=========================================================================
HDL Synthesis Report

Macro Statistics
# Adders/Subtractors                                    : 1
 8-bit subtractor                                       : 1
# Registers                                             : 5
 1-bit register                                         : 1
 163-bit register                                       : 3
 8-bit register                                         : 1
# Multiplexers                                          : 2
 1-bit 163-to-1 multiplexer                             : 1
 8-bit 2-to-1 multiplexer                               : 1
# Xors                                                  : 9791
 163-bit xor2                                           : 11
 164-bit xor2                                           : 9780

=========================================================================
```

```
=========================================================================
*                      Advanced HDL Synthesis                           *
=========================================================================


Synthesizing (advanced) Unit <ECC_K_163_Jac>.
The following registers are absorbed into counter <count>: 1 register on signal <count>.
Unit <ECC_K_163_Jac> synthesized (advanced).

=========================================================================
Advanced HDL Synthesis Report

Macro Statistics
# Counters                                             : 1
 8-bit down counter                                    : 1
# Registers                                            : 490
 Flip-Flops                                            : 490
# Multiplexers                                         : 1
 1-bit 163-to-1 multiplexer                            : 1
# Xors                                                 : 9791
 163-bit xor2                                          : 11
 164-bit xor2                                          : 9780

=========================================================================

=========================================================================
*                         Low Level Synthesis                           *
=========================================================================

Optimizing unit <ECC_K_163_Jac> ...

Optimizing unit <PD_K_163_Jac> ...

Optimizing unit <pol_SQ> ...

Optimizing unit <pol_mult> ...

Optimizing unit <PA_K_163_Jac> ...

Mapping all equations...
Building and optimizing final netlist ...
Found area constraint ratio of 100 (+ 5) on block ECC_K_163_Jac, actual ratio is 16.

Final Macro Processing ...

=========================================================================
Final Register Report

Macro Statistics
# Registers                                            : 498
 Flip-Flops                                            : 498

=========================================================================

=========================================================================
*                           Partition Report                            *
=========================================================================

Partition Implementation Status
-------------------------------

  No Partitions were found in this design.

-------------------------------

=========================================================================
*                            Design Summary                             *
=========================================================================

Top Level Output File Name         : ECC_K_163_Jac.ngc
```

```
Primitive and Black Box Usage:
------------------------------
# BELS                             : 226863
#      GND                         : 21
#      INV                         : 4
#      LUT2                        : 1913
#      LUT3                        : 3937
#      LUT4                        : 22667
#      LUT5                        : 7690
#      LUT6                        : 190379
#      MUXCY                       : 199
#      MUXF7                       : 25
#      MUXF8                       : 11
#      VCC                         : 9
#      XORCY                       : 8
# FlipFlops/Latches                : 498
#      FDC                         : 494
#      FDCE                        : 1
#      FDP                         : 3
# Clock Buffers                    : 1
#      BUFGP                       : 1
# IO Buffers                       : 654
#      IBUF                        : 164
#      OBUF                        : 490

Device utilization summary:
---------------------------

Selected Device : 6vlx760ff1760-2


Slice Logic Utilization:
 Number of Slice Registers:              498  out of  948480     0%
 Number of Slice LUTs:                226590  out of  474240    47%
    Number used as Logic:             226590  out of  474240    47%

Slice Logic Distribution:
 Number of LUT Flip Flop pairs used:  226593
   Number with an unused Flip Flop:   226095  out of  226593    99%
   Number with an unused LUT:              3  out of  226593     0%
   Number of fully used LUT-FF pairs:    495  out of  226593     0%
   Number of unique control sets:          2

IO Utilization:
 Number of IOs:                          655
 Number of bonded IOBs:                  655  out of   1200    54%

Specific Feature Utilization:
 Number of BUFG/BUFGCTRLs:                 1  out of     32     3%

---------------------------
Partition Resource Summary:
---------------------------

  No Partitions were found in this design.

---------------------------



=========================================================================
Timing Report

NOTE: THESE TIMING NUMBERS ARE ONLY A SYNTHESIS ESTIMATE.
      FOR ACCURATE TIMING INFORMATION PLEASE REFER TO THE TRACE REPORT
      GENERATED AFTER PLACE-and-ROUTE.

Clock Information:
------------------
-----------------------------------+------------------------+-------+
Clock Signal                       | Clock buffer(FF name)  | Load  |
```

```
-----------------------------------+------------------------+-------+
clk                                | BUFGP                  | 498   |
-----------------------------------+------------------------+-------+

Asynchronous Control Signals Information:
----------------------------------------
No asynchronous control signals found in this design

Timing Summary:
---------------
Speed Grade: -2

   Minimum period: 23.417ns (Maximum Frequency: 42.705MHz)
   Minimum input arrival time before clock: 2.765ns
   Maximum output required time after clock: 0.864ns
   Maximum combinational path delay: No path found

Timing Details:
---------------
All values displayed in nanoseconds (ns)

=========================================================================
Timing constraint: Default period analysis for Clock 'clk'
  Clock period: 23.417ns (frequency: 42.705MHz)
  Total number of paths / destination ports: 441903094726814130000 / 498
-------------------------------------------------------------------------
Delay:               23.417ns (Levels of Logic = 49)
  Source:            sQZ_94 (FF)
  Destination:       sQY_6 (FF)
  Source Clock:      clk rising
  Destination Clock: clk rising

  Data Path: sQZ_94 to sQY_6
                                Gate     Net
    Cell:in->out      fanout   Delay   Delay  Logical Name (Net Name)
    ----------------------------------------  ------------
     FDC:C->Q            385   0.317   0.891  sQZ_94 (sQZ_94)
     begin scope: 'uut_PD_Jac_163:Z1<94>'
     begin scope: 'uut_PD_Jac_163/SQ_SQ1:A<94>'
     LUT6:I0->O            1   0.061   0.566  Mxor_GND_8_o_GND_8_o_xor_178_OUT_163_xo<0>16
     (Mxor_GND_8_o_GND_8_o_xor_178_OUT_163_xo<0>15)
     LUT4:I0->O            1   0.061   0.696  Mxor_GND_8_o_GND_8_o_xor_178_OUT_163_xo<0>20
     (Mxor_GND_8_o_GND_8_o_xor_178_OUT_163_xo<0>19)
     LUT6:I0->O            4   0.061   0.443  Mxor_GND_8_o_GND_8_o_xor_178_OUT_163_xo<0>30
     (GND_8_o_GND_8_o_xor_178_OUT<163>)
     LUT5:I3->O          378   0.061   0.706  Mxor_SQ_BF.Cv_79_xo<0>31 (C<79>)
     end scope: 'uut_PD_Jac_163/SQ_SQ1:C<79>'
     begin scope: 'uut_PD_Jac_163/mult_M2:A<79>'
     LUT6:I3->O            1   0.061   0.512  Mxor_GND_9_o_GND_9_o_xor_170_OUT_163_xo<0>11
     (Mxor_GND_9_o_GND_9_o_xor_170_OUT_163_xo<0>10)
     LUT4:I1->O            1   0.061   0.426  Mxor_GND_9_o_GND_9_o_xor_170_OUT_163_xo<0>15
     (Mxor_GND_9_o_GND_9_o_xor_170_OUT_163_xo<0>14)
     LUT6:I4->O            1   0.061   0.357  Mxor_GND_9_o_GND_9_o_xor_170_OUT_163_xo<0>19
     (Mxor_GND_9_o_GND_9_o_xor_170_OUT_163_xo<0>18)
     LUT2:I1->O            4   0.061   0.443  Mxor_GND_9_o_GND_9_o_xor_170_OUT_163_xo<0>37
     (GND_9_o_GND_9_o_xor_170_OUT<163>)
     LUT6:I4->O          165   0.061   0.512  Mxor_mult_BF.Cv_84_xo<0>37 (C<84>)
     end scope: 'uut_PD_Jac_163/mult_M2:C<84>'
     LUT6:I5->O          535   0.061   0.914  Z3<84>1 (Z3<84>)
     end scope: 'uut_PD_Jac_163:Z3<84>'
     begin scope: 'uut_PA_Jac_163:Z2<84>'
     begin scope: 'uut_PA_Jac_163/SQ_SQ1:A<84>'
     LUT6:I0->O            1   0.061   0.512  Mxor_SQ_BF.Cv_129_xo<0>23
     (Mxor_SQ_BF.Cv_129_xo<0>22)
     LUT5:I2->O            1   0.061   0.357
     Mxor_GND_8_o_GND_8_o_xor_66_OUT_163_xo<0>11_SW1 (N3283)
     LUT6:I5->O            1   0.061   0.512  Mxor_SQ_BF.Cv_129_xo<0>28_SW1 (N1816)
     LUT6:I3->O          306   0.061   0.693  Mxor_SQ_BF.Cv_129_xo<0>50 (C<129>)
     end scope: 'uut_PA_Jac_163/SQ_SQ1:C<129>'
     begin scope: 'uut_PA_Jac_163/mult_M3:B<129>'
     LUT6:I3->O            1   0.061   0.566  Mxor_GND_9_o_GND_9_o_xor_74_OUT_163_xo<0>13
```

```
     (Mxor_GND_9_o_GND_9_o_xor_74_OUT_163_xo<0>12)
     LUT4:I0->O             1   0.061   0.357
     Mxor_GND_9_o_GND_9_o_xor_74_OUT_163_xo<0>71_SW0_SW0 (N2760)
     LUT6:I5->O             4   0.061   0.711  Mxor_GND_9_o_GND_9_o_xor_74_OUT_163_xo<0>16
     (GND_9_o_GND_9_o_xor_74_OUT<163>)
     LUT5:I0->O             1   0.061   0.357  Mxor_mult_BF.Cv_128_xo<0>58_SW0 (N364)
     LUT6:I5->O             1   0.061   0.357  Mxor_mult_BF.Cv_128_xo<0>58 (C<128>)
     end scope: 'uut_PA_Jac_163/mult_M3:C<128>'
     LUT2:I1->O           370   0.061   0.550  Mxor_A1_128_xo<0>1 (A1<128>)
     begin scope: 'uut_PA_Jac_163/mult_M7:B<128>'
     LUT4:I3->O             1   0.061   0.357  Mxor_n27224_136_xo<0>13_SW0 (N8)
     LUT5:I4->O           514   0.061   0.911  Mxor_mult_BF.Cv_137_xo<0>1 (C<137>)
     end scope: 'uut_PA_Jac_163/mult_M7:C<137>'
     begin scope: 'uut_PA_Jac_163/mult_M10:A<137>'
     LUT6:I1->O             1   0.061   0.357  Mxor_n26970_161_xo<0>5 (Mxor_n26970_161_xo<0>4)
     LUT6:I5->O             4   0.061   0.443  Mxor_GND_9_o_GND_9_o_xor_72_OUT_163_xo<0>1
     (GND_9_o_GND_9_o_xor_72_OUT<163>)
     LUT6:I4->O             1   0.061   0.357  Mxor_mult_BF.Cv_129_xo<0>37_SW0 (N3444)
     LUT6:I5->O           205   0.061   0.519  Mxor_mult_BF.Cv_129_xo<0>58 (C<129>)
     end scope: 'uut_PA_Jac_163/mult_M10:C<129>'
     LUT3:I2->O           326   0.061   0.697  A3<129>1 (A3<129>)
     begin scope: 'uut_PA_Jac_163/mult_M14:A<129>'
     LUT6:I3->O             1   0.061   0.357  Mxor_GND_9_o_GND_9_o_xor_148_OUT_163_xo<0>15
     (Mxor_GND_9_o_GND_9_o_xor_148_OUT_163_xo<0>14)
     LUT6:I5->O             1   0.061   0.694  Mxor_GND_9_o_GND_9_o_xor_148_OUT_163_xo<0>17
     (Mxor_GND_9_o_GND_9_o_xor_148_OUT_163_xo<0>16)
     LUT6:I1->O             4   0.061   0.713  Mxor_GND_9_o_GND_9_o_xor_148_OUT_163_xo<0>32
     (GND_9_o_GND_9_o_xor_148_OUT<163>)
     LUT6:I0->O             2   0.061   0.431  Mxor_mult_BF.Cv_94_xo<0>43 (C<94>)
     end scope: 'uut_PA_Jac_163/mult_M14:C<94>'
     LUT3:I1->O           163   0.061   0.580  Mxor_A5_94_xo<0>1 (A5<94>)
     begin scope: 'uut_PA_Jac_163/mult_M16:B<94>'
     LUT6:I4->O             1   0.061   0.566  Mxor_n27208_160_xo<0>64
     (Mxor_n27208_160_xo<0>63)
     LUT4:I0->O             1   0.061   0.696  Mxor_n27208_160_xo<0>65
     (Mxor_n27208_160_xo<0>64)
     LUT6:I0->O             2   0.061   0.362  Mxor_n27208_160_xo<0>30_SW0_SW0_SW0_SW0 (N3858)
     LUT6:I5->O             3   0.061   0.524  Mxor_n27208_160_xo<0>66 (n27208<160>)
     LUT6:I3->O             1   0.061   0.357  Mxor_mult_BF.Cv_6_xo<0>4 (C<6>)
     end scope: 'uut_PA_Jac_163/mult_M16:C<6>'
     LUT4:I3->O             1   0.061   0.357  Y3<6>1 (Y3<6>)
     end scope: 'uut_PA_Jac_163:Y3<6>'
     LUT3:I2->O             1   0.061   0.000  Q2Y[162]_Q2pY[162]_mux_2_OUT<6>1
     (Q2Y[162]_Q2pY[162]_mux_2_OUT<6>)
     FDC:D                       -0.002          sQY_6
    ----------------------------------------
    Total                       23.417ns (2.696ns logic, 20.721ns route)
                                         (11.5% logic, 88.5% route)

=========================================================================
Timing constraint: Default OFFSET IN BEFORE for Clock 'clk'
  Total number of paths / destination ports: 80205 / 987
-------------------------------------------------------------------------
Offset:              2.765ns (Levels of Logic = 8)
  Source:            key<102> (PAD)
  Destination:       sQX_0 (FF)
  Destination Clock: clk rising

  Data Path: key<102> to sQX_0
                                Gate     Net
    Cell:in->out      fanout   Delay   Delay  Logical Name (Net Name)
    ----------------------------------------  ------------
     IBUF:I->O              1   0.003   0.566  key_102_IBUF (key_102_IBUF)
     LUT6:I2->O             1   0.061   0.000  Mmux_count[7]_X_6_o_Mux_0_o_135
     (Mmux_count[7]_X_6_o_Mux_0_o_135)
     MUXF7:I1->O            1   0.211   0.000  Mmux_count[7]_X_6_o_Mux_0_o_12_f7_0
     (Mmux_count[7]_X_6_o_Mux_0_o_12_f71)
     MUXF8:I0->O            1   0.149   0.566  Mmux_count[7]_X_6_o_Mux_0_o_10_f8_0
     (Mmux_count[7]_X_6_o_Mux_0_o_10_f81)
     LUT6:I2->O             1   0.061   0.000  Mmux_count[7]_X_6_o_Mux_0_o_51
     (Mmux_count[7]_X_6_o_Mux_0_o_51)
```

```
     MUXF7:I1->O           1   0.211   0.000  Mmux_count[7]_X_6_o_Mux_0_o_4_f7
     (Mmux_count[7]_X_6_o_Mux_0_o_4_f7)
     MUXF8:I0->O         489   0.149   0.727  Mmux_count[7]_X_6_o_Mux_0_o_2_f8
     (count[7]_X_6_o_Mux_0_o)
     LUT3:I0->O            1   0.061   0.000  Q2X[162]_Q2pX[162]_mux_1_OUT<162>1
     (Q2X[162]_Q2pX[162]_mux_1_OUT<162>)
     FDC:D                    -0.002          sQX_162
    ----------------------------------------
    Total                      2.765ns (0.906ns logic, 1.859ns route)
                                       (32.8% logic, 67.2% route)

=========================================================================
Timing constraint: Default OFFSET OUT AFTER for Clock 'clk'
  Total number of paths / destination ports: 490 / 490
-------------------------------------------------------------------------
Offset:              0.864ns (Levels of Logic = 1)
  Source:            sQX_9 (FF)
  Destination:       QX<9> (PAD)
  Source Clock:      clk rising

  Data Path: sQX_9 to QX<9>
                                Gate     Net
    Cell:in->out      fanout   Delay   Delay  Logical Name (Net Name)
    ----------------------------------------  ------------
     FDC:C->Q            434   0.317   0.544  sQX_9 (sQX_9)
     OBUF:I->O                 0.003          QX_9_OBUF (QX<9>)
    ----------------------------------------
    Total                      0.864ns (0.320ns logic, 0.544ns route)
                                       (37.1% logic, 62.9% route)

=========================================================================

Cross Clock Domains Report:
--------------------------

Clock to Setup on destination clock clk
---------------+---------+---------+---------+---------+
               | Src:Rise| Src:Fall| Src:Rise| Src:Fall|
Source Clock   |Dest:Rise|Dest:Rise|Dest:Fall|Dest:Fall|
---------------+---------+---------+---------+---------+
clk            |   23.417|         |         |         |
---------------+---------+---------+---------+---------+

=========================================================================


Total REAL time to Xst completion: 1161.00 secs
Total CPU time to Xst completion: 1160.56 secs

-->

Total memory usage is 2840280 kilobytes

Number of errors   :    0 (   0 filtered)
Number of warnings :    0 (   0 filtered)
Number of infos    :    0 (   0 filtered)
```
