# Supplementary material for: Parallel point-multiplication architecture using combined group operations for high-speed cryptographic applications
Source: PLoS One. 2017 May 1;12(5):e0176214. doi: 10.1371/journal.pone.0176214 (PMC5411040; doi:10.1371/journal.pone.0176214)
Supplement: S1 Supporting Information — (ZIP) [file pone.0176214.s001.zip › S1 Supporting Information/S1 File14 Table2_[f].pdf]

```
Release 14.7 Map P.20131013 (nt64)
Xilinx Mapping Report File for Design 'ECC_K_163_Jac'

Design Information
------------------
Command Line   : map -intstyle ise -p xc6vlx760-ff1760-2 -w -logic_opt off -ol
high -t 1 -xt 0 -register_duplication off -r 4 -global_opt off -mt off -ir off
-pr off -lc off -power off -o ECC_K_163_Jac_map.ncd ECC_K_163_Jac.ngd
ECC_K_163_Jac.pcf
Target Device  : xc6vlx760
Target Package : ff1760
Target Speed   : -2
Mapper Version : virtex6 -- $Revision: 1.55 $
Mapped Date    : Tue May 24 22:02:37 2016

Design Summary
--------------
Number of errors:      0
Number of warnings:    0
Slice Logic Utilization:
  Number of Slice Registers:                   498 out of 948,480    1%
    Number used as Flip Flops:                 498
    Number used as Latches:                      0
    Number used as Latch-thrus:                  0
    Number used as AND/OR logics:                0
  Number of Slice LUTs:                    215,926 out of 474,240   45%
    Number used as logic:                  215,926 out of 474,240   45%
      Number using O6 output only:         215,617
      Number using O5 output only:               0
      Number using O5 and O6:                  309
      Number used as ROM:                        0
    Number used as Memory:                       0 out of 132,480    0%
    Number used exclusively as route-thrus:      0

Slice Logic Distribution:
  Number of occupied Slices:                77,573 out of 118,560   65%
  Number of LUT Flip Flop pairs used:      215,926
    Number with an unused Flip Flop:       215,509 out of 215,926   99%
    Number with an unused LUT:                   0 out of 215,926    0%
    Number of fully used LUT-FF pairs:         417 out of 215,926    1%
    Number of unique control sets:               2
    Number of slice register sites lost
      to control set restrictions:              14 out of 948,480    1%

  A LUT Flip Flop pair for this architecture represents one LUT paired with
  one Flip Flop within a slice.  A control set is a unique combination of
  clock, reset, set, and enable signals for a registered element.
  The Slice Logic Distribution report is not meaningful if the design is
  over-mapped for a non-slice resource or if Placement fails.
  OVERMAPPING of BRAM resources should be ignored if the design is
  over-mapped for a non-BRAM resource or if placement fails.

IO Utilization:
  Number of bonded IOBs:                       655 out of   1,200   54%

Specific Feature Utilization:
  Number of RAMB36E1/FIFO36E1s:                  0 out of     720    0%
  Number of RAMB18E1/FIFO18E1s:                  0 out of   1,440    0%
  Number of BUFG/BUFGCTRLs:                      1 out of      32    3%
    Number used as BUFGs:                        1
    Number used as BUFGCTRLs:                    0
  Number of ILOGICE1/ISERDESE1s:                 0 out of   1,440    0%
  Number of OLOGICE1/OSERDESE1s:                 0 out of   1,440    0%
  Number of BSCANs:                              0 out of       4    0%
  Number of BUFHCEs:                             0 out of     216    0%
  Number of BUFIODQSs:                           0 out of     144    0%
  Number of BUFRs:                               0 out of      72    0%
  Number of CAPTUREs:                            0 out of       1    0%
  Number of DSP48E1s:                            0 out of     864    0%
  Number of EFUSE_USRs:                          0 out of       1    0%
  Number of FRAME_ECCs:                          0 out of       1    0%
```

```
  Number of ICAPs:                              0 out of       2    0%
  Number of IDELAYCTRLs:                        0 out of      36    0%
  Number of IODELAYE1s:                         0 out of   1,440    0%
  Number of MMCM_ADVs:                          0 out of      18    0%
  Number of STARTUPs:                           0 out of       1    0%
  Number of SYSMONs:                            0 out of       1    0%

Average Fanout of Non-Clock Nets:               5.59

Peak Memory Usage:  4074 MB
Total REAL time to MAP completion:  1 hrs 56 mins 36 secs
Total CPU time to MAP completion:   1 hrs 56 mins 8 secs

Table of Contents
-----------------
Section 1 - Errors
Section 2 - Warnings
Section 3 - Informational
Section 4 - Removed Logic Summary
Section 5 - Removed Logic
Section 6 - IOB Properties
Section 7 - RPMs
Section 8 - Guide Report
Section 9 - Area Group and Partition Summary
Section 10 - Timing Report
Section 11 - Configuration String Information
Section 12 - Control Set Information
Section 13 - Utilization by Hierarchy

Section 1 - Errors
------------------

Section 2 - Warnings
--------------------

Section 3 - Informational
-------------------------
INFO:MapLib:562 - No environment variables are currently set.
INFO:LIT:244 - All of the single ended outputs in this design are using slew
   rate limited output drivers. The delay on speed critical single ended outputs
   can be dramatically reduced by designating them as fast outputs.
INFO:Pack:1716 - Initializing temperature to 85.000 Celsius. (default - Range:
   0.000 to 85.000 Celsius)
INFO:Pack:1720 - Initializing voltage to 0.950 Volts. (default - Range: 0.950 to
   1.050 Volts)
INFO:Map:215 - The Interim Design Summary has been generated in the MAP Report
   (.mrp).
INFO:Pack:1650 - Map created a placed design.

Section 4 - Removed Logic Summary
---------------------------------
  24 block(s) removed
 160 block(s) optimized away
  24 signal(s) removed

Section 5 - Removed Logic
-------------------------

The trimmed logic report below shows the logic removed from your design due to
sourceless or loadless signals, and VCC or ground connections.  If the removal
of a signal or symbol results in the subsequent removal of an additional signal
or symbol, the message explaining that second removal will be indented.  This
indentation will be repeated as a chain of related logic is removed.

To quickly locate the original cause for the removal of a chain of logic, look
above the place where that logic is listed in the trimming report, then locate
the lines that are least indented (begin at the leftmost edge).

The signal "uut_PD_Jac_163/SQ_SQ3/Mxor_GND_8_o_GND_8_o_xor_228_OUT_163_xo<0>29"
is sourceless and has been removed.
The signal "uut_PD_Jac_163/SQ_SQ3/Mxor_GND_8_o_GND_8_o_xor_228_OUT_163_xo<0>30"
```

is sourceless and has been removed.
The signal "uut_PD_Jac_163/SQ_SQ3/Mxor_GND_8_o_GND_8_o_xor_228_OUT_163_xo<0>32" is sourceless and has been removed.
The signal "uut_PD_Jac_163/SQ_SQ3/Mxor_GND_8_o_GND_8_o_xor_224_OUT_163_xo<0>29" is sourceless and has been removed.
The signal "uut_PD_Jac_163/SQ_SQ3/Mxor_GND_8_o_GND_8_o_xor_224_OUT_163_xo<0>30" is sourceless and has been removed.
The signal "uut_PD_Jac_163/SQ_SQ3/Mxor_GND_8_o_GND_8_o_xor_224_OUT_163_xo<0>32" is sourceless and has been removed.
The signal "uut_PD_Jac_163/SQ_SQ3/Mxor_n13849_160_xo<0>29" is sourceless and has been removed.
The signal "uut_PD_Jac_163/SQ_SQ3/Mxor_n13849_160_xo<0>30" is sourceless and has been removed.
The signal "uut_PD_Jac_163/SQ_SQ1/Mxor_SQ_BF.Cv_46_xo<0>13" is sourceless and has been removed.
The signal "uut_PD_Jac_163/SQ_SQ1/Mxor_SQ_BF.Cv_64_xo<0>10" is sourceless and has been removed.
The signal "uut_PD_Jac_163/SQ_SQ1/Mxor_SQ_BF.Cv_64_xo<0>11" is sourceless and has been removed.
The signal "uut_PD_Jac_163/SQ_SQ1/Mxor_n13849_160_xo<0>29" is sourceless and has been removed.
The signal "uut_PD_Jac_163/SQ_SQ1/Mxor_n13849_160_xo<0>30" is sourceless and has been removed.
The signal "uut_PD_Jac_163/SQ_SQ2/Mxor_GND_8_o_GND_8_o_xor_58_OUT_163_xo<0>" is sourceless and has been removed.
The signal "uut_PD_Jac_163/SQ_SQ2/Mxor_GND_8_o_GND_8_o_xor_58_OUT_163_xo<0>2" is sourceless and has been removed.
The signal "uut_PD_Jac_163/SQ_SQ2/Mxor_SQ_BF.Cv_64_xo<0>10" is sourceless and has been removed.
The signal "uut_PD_Jac_163/SQ_SQ2/Mxor_SQ_BF.Cv_64_xo<0>11" is sourceless and has been removed.
The signal "uut_PD_Jac_163/SQ_SQ2/Mxor_GND_8_o_GND_8_o_xor_186_OUT_163_xo<0>12" is sourceless and has been removed.
The signal "uut_PD_Jac_163/SQ_SQ2/Mxor_n13849_160_xo<0>29" is sourceless and has been removed.
The signal "uut_PD_Jac_163/SQ_SQ2/Mxor_n13849_160_xo<0>30" is sourceless and has been removed.
The signal "uut_PA_Jac_163/SQ_SQ4/Mxor_SQ_BF.Cv_64_xo<0>10" is sourceless and has been removed.
The signal "uut_PA_Jac_163/SQ_SQ4/Mxor_SQ_BF.Cv_64_xo<0>11" is sourceless and has been removed.
The signal "uut_PA_Jac_163/SQ_SQ4/Mxor_GND_8_o_GND_8_o_xor_272_OUT_163_xo<0>16" is sourceless and has been removed.
The signal "uut_PA_Jac_163/SQ_SQ4/Mxor_GND_8_o_GND_8_o_xor_272_OUT_163_xo<0>17" is sourceless and has been removed.
Unused block "uut_PA_Jac_163/SQ_SQ4/Mxor_GND_8_o_GND_8_o_xor_272_OUT_163_xo<0>17" (ROM) removed.
Unused block "uut_PA_Jac_163/SQ_SQ4/Mxor_GND_8_o_GND_8_o_xor_272_OUT_163_xo<0>18" (ROM) removed.
Unused block "uut_PA_Jac_163/SQ_SQ4/Mxor_SQ_BF.Cv_64_xo<0>6" (ROM) removed.
Unused block "uut_PA_Jac_163/SQ_SQ4/Mxor_SQ_BF.Cv_64_xo<0>9" (ROM) removed.
Unused block "uut_PD_Jac_163/SQ_SQ1/Mxor_SQ_BF.Cv_46_xo<0>8" (ROM) removed.
Unused block "uut_PD_Jac_163/SQ_SQ1/Mxor_SQ_BF.Cv_64_xo<0>6" (ROM) removed.
Unused block "uut_PD_Jac_163/SQ_SQ1/Mxor_SQ_BF.Cv_64_xo<0>9" (ROM) removed.
Unused block "uut_PD_Jac_163/SQ_SQ1/Mxor_n13849_160_xo<0>30" (ROM) removed.
Unused block "uut_PD_Jac_163/SQ_SQ1/Mxor_n13849_160_xo<0>31" (ROM) removed.
Unused block "uut_PD_Jac_163/SQ_SQ2/Mxor_GND_8_o_GND_8_o_xor_186_OUT_163_xo<0>13" (ROM) removed.
Unused block "uut_PD_Jac_163/SQ_SQ2/Mxor_GND_8_o_GND_8_o_xor_58_OUT_163_xo<0>1" (ROM) removed.
Unused block "uut_PD_Jac_163/SQ_SQ2/Mxor_GND_8_o_GND_8_o_xor_58_OUT_163_xo<0>3" (ROM) removed.
Unused block "uut_PD_Jac_163/SQ_SQ2/Mxor_SQ_BF.Cv_64_xo<0>6" (ROM) removed.
Unused block "uut_PD_Jac_163/SQ_SQ2/Mxor_SQ_BF.Cv_64_xo<0>9" (ROM) removed.
Unused block "uut_PD_Jac_163/SQ_SQ2/Mxor_n13849_160_xo<0>30" (ROM) removed.
Unused block "uut_PD_Jac_163/SQ_SQ2/Mxor_n13849_160_xo<0>31" (ROM) removed.
Unused block "uut_PD_Jac_163/SQ_SQ3/Mxor_GND_8_o_GND_8_o_xor_224_OUT_163_xo<0>5" (ROM) removed.

```
Unused block "uut_PD_Jac_163/SQ_SQ3/Mxor_GND_8_o_GND_8_o_xor_224_OUT_163_xo<0>7"
(ROM) removed.
Unused block "uut_PD_Jac_163/SQ_SQ3/Mxor_GND_8_o_GND_8_o_xor_224_OUT_163_xo<0>8"
(ROM) removed.
Unused block "uut_PD_Jac_163/SQ_SQ3/Mxor_GND_8_o_GND_8_o_xor_228_OUT_163_xo<0>5"
(ROM) removed.
Unused block "uut_PD_Jac_163/SQ_SQ3/Mxor_GND_8_o_GND_8_o_xor_228_OUT_163_xo<0>7"
(ROM) removed.
Unused block "uut_PD_Jac_163/SQ_SQ3/Mxor_GND_8_o_GND_8_o_xor_228_OUT_163_xo<0>8"
(ROM) removed.
Unused block "uut_PD_Jac_163/SQ_SQ3/Mxor_n13849_160_xo<0>30" (ROM) removed.
Unused block "uut_PD_Jac_163/SQ_SQ3/Mxor_n13849_160_xo<0>31" (ROM) removed.

Optimized Block(s):
TYPE         BLOCK
GND          XST_GND
VCC          XST_VCC
GND          uut_PA_Jac_163/SQ_SQ1/XST_GND
GND          uut_PA_Jac_163/SQ_SQ2/XST_GND
VCC          uut_PA_Jac_163/SQ_SQ2/XST_VCC
GND          uut_PA_Jac_163/SQ_SQ3/XST_GND
GND          uut_PA_Jac_163/SQ_SQ4/XST_GND
GND          uut_PA_Jac_163/SQ_SQ5/XST_GND
GND          uut_PA_Jac_163/XST_GND
VCC          uut_PA_Jac_163/XST_VCC
LUT4         uut_PA_Jac_163/mult_M15/Mxor_n27224_136_xo<0>13_SW0
   optimized to 0
LUT4         uut_PA_Jac_163/mult_M15/Mxor_n27224_34_xo<0>3_SW0_SW0
   optimized to 0
LUT4         uut_PA_Jac_163/mult_M15/Mxor_n27224_51_xo<0>3_SW0_SW0
   optimized to 0
LUT4         uut_PA_Jac_163/mult_M15/Mxor_n27224_68_xo<0>16_SW0
   optimized to 0
LUT4         uut_PA_Jac_163/mult_M15/Mxor_n27224_85_xo<0>16_SW0_SW0
   optimized to 0
GND          uut_PA_Jac_163/mult_M15/XST_GND
VCC          uut_PA_Jac_163/mult_M15/XST_VCC
GND          uut_PA_Jac_163/mult_M2/XST_GND
VCC          uut_PA_Jac_163/mult_M2/XST_VCC
LUT4         uut_PA_Jac_163/mult_M3/Mxor_GND_9_o_GND_9_o_xor_104_OUT_163_xo<0>5_SW0
   optimized to 0
LUT4         uut_PA_Jac_163/mult_M3/Mxor_GND_9_o_GND_9_o_xor_124_OUT_163_xo<0>10_SW0
   optimized to 0
LUT4         uut_PA_Jac_163/mult_M3/Mxor_GND_9_o_GND_9_o_xor_130_OUT_163_xo<0>26_SW0
   optimized to 0
LUT4         uut_PA_Jac_163/mult_M3/Mxor_GND_9_o_GND_9_o_xor_136_OUT_163_xo<0>4_SW0
   optimized to 0
LUT2         uut_PA_Jac_163/mult_M3/Mxor_GND_9_o_GND_9_o_xor_152_OUT_163_xo<0>28_SW0
   optimized to 1
LUT4         uut_PA_Jac_163/mult_M3/Mxor_GND_9_o_GND_9_o_xor_154_OUT_163_xo<0>19_SW0
   optimized to 0
LUT4         uut_PA_Jac_163/mult_M3/Mxor_GND_9_o_GND_9_o_xor_162_OUT_163_xo<0>21_SW0
   optimized to 0
LUT6         uut_PA_Jac_163/mult_M3/Mxor_GND_9_o_GND_9_o_xor_178_OUT_163_xo<0>34_SW0
   optimized to 1
LUT4         uut_PA_Jac_163/mult_M3/Mxor_GND_9_o_GND_9_o_xor_190_OUT_163_xo<0>27_SW0
   optimized to 0
LUT4         uut_PA_Jac_163/mult_M3/Mxor_GND_9_o_GND_9_o_xor_190_OUT_163_xo<0>4_SW0
   optimized to 0
LUT4         uut_PA_Jac_163/mult_M3/Mxor_GND_9_o_GND_9_o_xor_200_OUT_163_xo<0>18_SW0
   optimized to 0
LUT2         uut_PA_Jac_163/mult_M3/Mxor_GND_9_o_GND_9_o_xor_218_OUT_163_xo<0>25_SW0
   optimized to 1
LUT4         uut_PA_Jac_163/mult_M3/Mxor_GND_9_o_GND_9_o_xor_218_OUT_163_xo<0>45_SW0
   optimized to 0
LUT4         uut_PA_Jac_163/mult_M3/Mxor_GND_9_o_GND_9_o_xor_218_OUT_163_xo<0>7_SW0
   optimized to 0
LUT4         uut_PA_Jac_163/mult_M3/Mxor_GND_9_o_GND_9_o_xor_222_OUT_163_xo<0>39_SW0
   optimized to 0
LUT4         uut_PA_Jac_163/mult_M3/Mxor_GND_9_o_GND_9_o_xor_230_OUT_163_xo<0>47_SW0
   optimized to 0
```

```
LUT4         uut_PA_Jac_163/mult_M3/Mxor_GND_9_o_GND_9_o_xor_240_OUT_163_xo<0>49_SW0
   optimized to 0
LUT2         uut_PA_Jac_163/mult_M3/Mxor_GND_9_o_GND_9_o_xor_242_OUT_163_xo<0>1_SW0
   optimized to 1
LUT4         uut_PA_Jac_163/mult_M3/Mxor_GND_9_o_GND_9_o_xor_248_OUT_163_xo<0>50_SW0
   optimized to 0
LUT4         uut_PA_Jac_163/mult_M3/Mxor_GND_9_o_GND_9_o_xor_268_OUT_163_xo<0>11_SW0
   optimized to 0
LUT4         uut_PA_Jac_163/mult_M3/Mxor_GND_9_o_GND_9_o_xor_268_OUT_163_xo<0>55_SW0
   optimized to 0
LUT6         uut_PA_Jac_163/mult_M3/Mxor_GND_9_o_GND_9_o_xor_26_OUT_163_xo<0>4_SW0
   optimized to 1
LUT4         uut_PA_Jac_163/mult_M3/Mxor_GND_9_o_GND_9_o_xor_270_OUT_163_xo<0>28_SW0
   optimized to 0
LUT2         uut_PA_Jac_163/mult_M3/Mxor_GND_9_o_GND_9_o_xor_276_OUT_163_xo<0>1_SW0
   optimized to 1
LUT4         uut_PA_Jac_163/mult_M3/Mxor_GND_9_o_GND_9_o_xor_280_OUT_163_xo<0>3_SW0
   optimized to 0
LUT4         uut_PA_Jac_163/mult_M3/Mxor_GND_9_o_GND_9_o_xor_282_OUT_163_xo<0>11_SW0
   optimized to 0
LUT4         uut_PA_Jac_163/mult_M3/Mxor_GND_9_o_GND_9_o_xor_298_OUT_163_xo<0>2_SW0
   optimized to 0
LUT4
        uut_PA_Jac_163/mult_M3/Mxor_GND_9_o_GND_9_o_xor_298_OUT_163_xo<0>53_SW0_SW0
   optimized to 0
LUT4         uut_PA_Jac_163/mult_M3/Mxor_GND_9_o_GND_9_o_xor_304_OUT_163_xo<0>13_SW0
   optimized to 0
LUT4
        uut_PA_Jac_163/mult_M3/Mxor_GND_9_o_GND_9_o_xor_304_OUT_163_xo<0>17_SW0_SW0
   optimized to 0
LUT4         uut_PA_Jac_163/mult_M3/Mxor_GND_9_o_GND_9_o_xor_304_OUT_163_xo<0>49_SW0
   optimized to 0
LUT4
        uut_PA_Jac_163/mult_M3/Mxor_GND_9_o_GND_9_o_xor_308_OUT_163_xo<0>16_SW0_SW0_SW
0
   optimized to 0
LUT4         uut_PA_Jac_163/mult_M3/Mxor_GND_9_o_GND_9_o_xor_322_OUT_163_xo<0>17_SW0
   optimized to 0
LUT4         uut_PA_Jac_163/mult_M3/Mxor_GND_9_o_GND_9_o_xor_322_OUT_163_xo<0>29_SW0
   optimized to 0
LUT4         uut_PA_Jac_163/mult_M3/Mxor_mult_BF.Cv_108_xo<0>16_SW0
   optimized to 0
LUT4         uut_PA_Jac_163/mult_M3/Mxor_mult_BF.Cv_118_xo<0>8_SW0
   optimized to 0
LUT4         uut_PA_Jac_163/mult_M3/Mxor_mult_BF.Cv_128_xo<0>53_SW0
   optimized to 0
LUT4         uut_PA_Jac_163/mult_M3/Mxor_mult_BF.Cv_130_xo<0>48_SW0
   optimized to 0
LUT4         uut_PA_Jac_163/mult_M3/Mxor_mult_BF.Cv_131_xo<0>28_SW0
   optimized to 0
LUT4         uut_PA_Jac_163/mult_M3/Mxor_mult_BF.Cv_132_xo<0>3_SW0
   optimized to 0
LUT4         uut_PA_Jac_163/mult_M3/Mxor_mult_BF.Cv_134_xo<0>16_SW0
   optimized to 0
LUT4         uut_PA_Jac_163/mult_M3/Mxor_mult_BF.Cv_136_xo<0>52_SW0
   optimized to 0
LUT4         uut_PA_Jac_163/mult_M3/Mxor_mult_BF.Cv_141_xo<0>43_SW0
   optimized to 0
LUT4         uut_PA_Jac_163/mult_M3/Mxor_mult_BF.Cv_146_xo<0>19_SW0
   optimized to 0
LUT4         uut_PA_Jac_163/mult_M3/Mxor_mult_BF.Cv_148_xo<0>12_SW0
   optimized to 0
LUT4         uut_PA_Jac_163/mult_M3/Mxor_mult_BF.Cv_148_xo<0>18_SW0
   optimized to 0
LUT4         uut_PA_Jac_163/mult_M3/Mxor_mult_BF.Cv_150_xo<0>2_SW0
   optimized to 0
LUT4         uut_PA_Jac_163/mult_M3/Mxor_mult_BF.Cv_156_xo<0>51_SW0
   optimized to 0
LUT4         uut_PA_Jac_163/mult_M3/Mxor_mult_BF.Cv_157_xo<0>41_SW0
   optimized to 0
LUT4         uut_PA_Jac_163/mult_M3/Mxor_mult_BF.Cv_162_xo<0>12_SW0
```

```
   optimized to 0
LUT4         uut_PA_Jac_163/mult_M3/Mxor_mult_BF.Cv_36_xo<0>8_SW0_SW0
   optimized to 0
LUT4         uut_PA_Jac_163/mult_M3/Mxor_mult_BF.Cv_38_xo<0>8_SW0
   optimized to 0
LUT4         uut_PA_Jac_163/mult_M3/Mxor_mult_BF.Cv_41_xo<0>15_SW0
   optimized to 0
LUT2         uut_PA_Jac_163/mult_M3/Mxor_mult_BF.Cv_67_xo<0>27_SW0
   optimized to 1
LUT2         uut_PA_Jac_163/mult_M3/Mxor_mult_BF.Cv_94_xo<0>39_SW0
   optimized to 1
LUT4         uut_PA_Jac_163/mult_M3/Mxor_mult_BF.Cv_97_xo<0>25_SW0
   optimized to 0
LUT2         uut_PA_Jac_163/mult_M3/Mxor_n26936_161_xo<0>7_SW0
   optimized to 1
LUT4         uut_PA_Jac_163/mult_M3/Mxor_n27038_161_xo<0>9_SW0
   optimized to 0
LUT4         uut_PA_Jac_163/mult_M3/Mxor_n27208_161_xo<0>48_SW0
   optimized to 0
LUT2         uut_PA_Jac_163/mult_M3/Mxor_n27208_161_xo<0>7_SW0
   optimized to 1
LUT2         uut_PA_Jac_163/mult_M3/Mxor_n27224_136_xo<0>60_SW0
   optimized to 1
GND          uut_PA_Jac_163/mult_M3/XST_GND
LUT4         uut_PA_Jac_163/mult_M4/Mxor_n27224_102_xo<0>31_SW0
   optimized to 0
LUT4         uut_PA_Jac_163/mult_M4/Mxor_n27224_119_xo<0>44_SW0
   optimized to 0
LUT4         uut_PA_Jac_163/mult_M4/Mxor_n27224_136_xo<0>13_SW0
   optimized to 0
LUT4         uut_PA_Jac_163/mult_M4/Mxor_n27224_153_xo<0>34_SW0
   optimized to 0
LUT4         uut_PA_Jac_163/mult_M4/Mxor_n27224_17_xo<0>3_SW0
   optimized to 0
LUT4         uut_PA_Jac_163/mult_M4/Mxor_n27224_34_xo<0>3_SW0
   optimized to 0
LUT4         uut_PA_Jac_163/mult_M4/Mxor_n27224_51_xo<0>3_SW0
   optimized to 0
LUT4         uut_PA_Jac_163/mult_M4/Mxor_n27224_68_xo<0>16_SW0_SW0
   optimized to 0
LUT4         uut_PA_Jac_163/mult_M4/Mxor_n27224_85_xo<0>16_SW0
   optimized to 0
GND          uut_PA_Jac_163/mult_M4/XST_GND
VCC          uut_PA_Jac_163/mult_M4/XST_VCC
LUT4         uut_PA_Jac_163/mult_M5/Mxor_GND_9_o_GND_9_o_xor_152_OUT_163_xo<0>30_SW0
   optimized to 0
LUT2         uut_PA_Jac_163/mult_M5/Mxor_GND_9_o_GND_9_o_xor_160_OUT_163_xo<0>1_SW0
   optimized to 1
LUT2         uut_PA_Jac_163/mult_M5/Mxor_GND_9_o_GND_9_o_xor_162_OUT_163_xo<0>1_SW0
   optimized to 1
LUT4         uut_PA_Jac_163/mult_M5/Mxor_GND_9_o_GND_9_o_xor_162_OUT_163_xo<0>21_SW0
   optimized to 0
LUT4         uut_PA_Jac_163/mult_M5/Mxor_GND_9_o_GND_9_o_xor_184_OUT_163_xo<0>12_SW0
   optimized to 0
LUT4         uut_PA_Jac_163/mult_M5/Mxor_GND_9_o_GND_9_o_xor_204_OUT_163_xo<0>8_SW0
   optimized to 0
LUT4         uut_PA_Jac_163/mult_M5/Mxor_GND_9_o_GND_9_o_xor_212_OUT_163_xo<0>18_SW0
   optimized to 0
LUT4         uut_PA_Jac_163/mult_M5/Mxor_GND_9_o_GND_9_o_xor_216_OUT_163_xo<0>17_SW0
   optimized to 0
LUT4         uut_PA_Jac_163/mult_M5/Mxor_GND_9_o_GND_9_o_xor_220_OUT_163_xo<0>22_SW0
   optimized to 0
LUT4         uut_PA_Jac_163/mult_M5/Mxor_GND_9_o_GND_9_o_xor_236_OUT_163_xo<0>26_SW0
   optimized to 0
LUT2         uut_PA_Jac_163/mult_M5/Mxor_GND_9_o_GND_9_o_xor_238_OUT_163_xo<0>27_SW0
   optimized to 1
LUT4         uut_PA_Jac_163/mult_M5/Mxor_GND_9_o_GND_9_o_xor_240_OUT_163_xo<0>34_SW0
   optimized to 0
LUT2         uut_PA_Jac_163/mult_M5/Mxor_GND_9_o_GND_9_o_xor_260_OUT_163_xo<0>21_SW0
   optimized to 1
LUT4         uut_PA_Jac_163/mult_M5/Mxor_GND_9_o_GND_9_o_xor_262_OUT_163_xo<0>47_SW0
```

```
   optimized to 0
LUT4         uut_PA_Jac_163/mult_M5/Mxor_GND_9_o_GND_9_o_xor_268_OUT_163_xo<0>39_SW0
   optimized to 0
LUT4         uut_PA_Jac_163/mult_M5/Mxor_GND_9_o_GND_9_o_xor_268_OUT_163_xo<0>54_SW0
   optimized to 0
LUT2         uut_PA_Jac_163/mult_M5/Mxor_GND_9_o_GND_9_o_xor_278_OUT_163_xo<0>53_SW0
   optimized to 1
LUT2
        uut_PA_Jac_163/mult_M5/Mxor_GND_9_o_GND_9_o_xor_282_OUT_163_xo<0>41_SW0_SW0
   optimized to 1
LUT2         uut_PA_Jac_163/mult_M5/Mxor_GND_9_o_GND_9_o_xor_284_OUT_163_xo<0>54_SW0
   optimized to 1
LUT4         uut_PA_Jac_163/mult_M5/Mxor_GND_9_o_GND_9_o_xor_306_OUT_163_xo<0>1_SW0
   optimized to 0
LUT4         uut_PA_Jac_163/mult_M5/Mxor_GND_9_o_GND_9_o_xor_308_OUT_163_xo<0>19_SW0
   optimized to 0
LUT6         uut_PA_Jac_163/mult_M5/Mxor_mult_BF.Cv_105_xo<0>27_SW0
   optimized to 1
LUT4         uut_PA_Jac_163/mult_M5/Mxor_mult_BF.Cv_106_xo<0>21_SW0
   optimized to 0
LUT4         uut_PA_Jac_163/mult_M5/Mxor_mult_BF.Cv_112_xo<0>40_SW0
   optimized to 0
LUT4         uut_PA_Jac_163/mult_M5/Mxor_mult_BF.Cv_115_xo<0>21_SW0
   optimized to 0
LUT4         uut_PA_Jac_163/mult_M5/Mxor_mult_BF.Cv_125_xo<0>20_SW0
   optimized to 0
LUT4         uut_PA_Jac_163/mult_M5/Mxor_mult_BF.Cv_146_xo<0>34_SW0
   optimized to 0
LUT2         uut_PA_Jac_163/mult_M5/Mxor_mult_BF.Cv_158_xo<0>26_SW0
   optimized to 1
LUT4         uut_PA_Jac_163/mult_M5/Mxor_mult_BF.Cv_33_xo<0>10_SW0
   optimized to 0
LUT2         uut_PA_Jac_163/mult_M5/Mxor_mult_BF.Cv_40_xo<0>9_SW0
   optimized to 1
LUT2         uut_PA_Jac_163/mult_M5/Mxor_mult_BF.Cv_48_xo<0>13_SW0
   optimized to 1
LUT4         uut_PA_Jac_163/mult_M5/Mxor_mult_BF.Cv_6_xo<0>2_SW0
   optimized to 0
LUT4         uut_PA_Jac_163/mult_M5/Mxor_mult_BF.Cv_71_xo<0>28_SW0
   optimized to 0
LUT2         uut_PA_Jac_163/mult_M5/Mxor_mult_BF.Cv_81_xo<0>5_SW0
   optimized to 1
LUT4         uut_PA_Jac_163/mult_M5/Mxor_mult_BF.Cv_8_xo<0>3_SW0_SW0
   optimized to 0
LUT4         uut_PA_Jac_163/mult_M5/Mxor_mult_BF.Cv_90_xo<0>28_SW0
   optimized to 0
LUT4         uut_PA_Jac_163/mult_M5/Mxor_mult_BF.Pv_163_xo<0>24_SW0
   optimized to 0
LUT4         uut_PA_Jac_163/mult_M5/Mxor_n27208_161_xo<0>24_SW0
   optimized to 0
LUT2         uut_PA_Jac_163/mult_M5/Mxor_n27224_17_xo<0>8_SW0
   optimized to 1
GND          uut_PA_Jac_163/mult_M5/XST_GND
LUT4         uut_PA_Jac_163/mult_M6/Mxor_mult_BF.Cv_126_xo<0>53_SW0
   optimized to 0
LUT4         uut_PA_Jac_163/mult_M6/Mxor_mult_BF.Cv_138_xo<0>59_SW0
   optimized to 0
LUT4         uut_PA_Jac_163/mult_M6/Mxor_mult_BF.Cv_141_xo<0>59_SW0
   optimized to 0
LUT4         uut_PA_Jac_163/mult_M6/Mxor_mult_BF.Cv_159_xo<0>61_SW0_SW0_SW0
   optimized to 0
LUT4         uut_PA_Jac_163/mult_M6/Mxor_mult_BF.Cv_3_xo<0>_SW0_SW0
   optimized to 0
LUT4         uut_PA_Jac_163/mult_M6/Mxor_mult_BF.Cv_6_xo<0>1_SW0_SW0
   optimized to 0
LUT4         uut_PA_Jac_163/mult_M6/Mxor_mult_BF.Cv_78_xo<0>16_SW0
   optimized to 0
LUT4         uut_PA_Jac_163/mult_M6/Mxor_mult_BF.Cv_9_xo<0>1_SW0
   optimized to 0
GND          uut_PA_Jac_163/mult_M6/XST_GND
LUT4         uut_PA_Jac_163/mult_M7/Mxor_n27224_102_xo<0>31_SW0_SW0_SW0
```

```
   optimized to 0
LUT4        uut_PA_Jac_163/mult_M7/Mxor_n27224_119_xo<0>44_SW0
   optimized to 0
LUT4        uut_PA_Jac_163/mult_M7/Mxor_n27224_136_xo<0>13_SW0
   optimized to 0
LUT4        uut_PA_Jac_163/mult_M7/Mxor_n27224_153_xo<0>34_SW0
   optimized to 0
LUT4        uut_PA_Jac_163/mult_M7/Mxor_n27224_17_xo<0>3_SW0
   optimized to 0
LUT4        uut_PA_Jac_163/mult_M7/Mxor_n27224_34_xo<0>3_SW0_SW0
   optimized to 0
GND         uut_PA_Jac_163/mult_M7/XST_GND
VCC         uut_PA_Jac_163/mult_M7/XST_VCC
GND         uut_PD_Jac_163/SQ_SQ1/XST_GND
GND         uut_PD_Jac_163/SQ_SQ2/XST_GND
GND         uut_PD_Jac_163/SQ_SQ3/XST_GND
GND         uut_PD_Jac_163/SQ_SQ4/XST_GND
GND         uut_PD_Jac_163/SQ_SQ5/XST_GND
GND         uut_PD_Jac_163/XST_GND
VCC         uut_PD_Jac_163/XST_VCC
LUT4        uut_PD_Jac_163/mult_M4/Mxor_n27224_102_xo<0>31_SW0
   optimized to 0
LUT4        uut_PD_Jac_163/mult_M4/Mxor_n27224_34_xo<0>3_SW0_SW0
   optimized to 0
GND         uut_PD_Jac_163/mult_M4/XST_GND
VCC         uut_PD_Jac_163/mult_M4/XST_VCC

To enable printing of redundant blocks removed and signals merged, set the
detailed map report option and rerun map.

Section 6 - IOB Properties
--------------------------

+-----------------------------------------------------------------------------------------------
-------------------------------------------------------------+
| IOB Name                           | Type             | Direction | IO Standard          |
Diff  | Drive    | Slew | Reg (s)      | Resistor | IOB      |
|                                    |                  |           |                      |
Term  | Strength | Rate |              |          | Delay    |
+-----------------------------------------------------------------------------------------------
-------------------------------------------------------------+
| QX<0>                              | IOB              | OUTPUT    | LVCMOS25
|       | 12       | SLOW |              |          |          |
| QX<1>                              | IOB              | OUTPUT    | LVCMOS25
|       | 12       | SLOW |              |          |          |
| QX<2>                              | IOB              | OUTPUT    | LVCMOS25
|       | 12       | SLOW |              |          |          |
| QX<3>                              | IOB              | OUTPUT    | LVCMOS25
|       | 12       | SLOW |              |          |          |
| QX<4>                              | IOB              | OUTPUT    | LVCMOS25
|       | 12       | SLOW |              |          |          |
| QX<5>                              | IOB              | OUTPUT    | LVCMOS25
|       | 12       | SLOW |              |          |          |
| QX<6>                              | IOB              | OUTPUT    | LVCMOS25
|       | 12       | SLOW |              |          |          |
| QX<7>                              | IOB              | OUTPUT    | LVCMOS25
|       | 12       | SLOW |              |          |          |
| QX<8>                              | IOB              | OUTPUT    | LVCMOS25
|       | 12       | SLOW |              |          |          |
| QX<9>                              | IOB              | OUTPUT    | LVCMOS25
|       | 12       | SLOW |              |          |          |
| QX<10>                             | IOB              | OUTPUT    | LVCMOS25
|       | 12       | SLOW |              |          |          |
| QX<11>                             | IOB              | OUTPUT    | LVCMOS25
|       | 12       | SLOW |              |          |          |
| QX<12>                             | IOB              | OUTPUT    | LVCMOS25
|       | 12       | SLOW |              |          |          |
| QX<13>                             | IOB              | OUTPUT    | LVCMOS25
|       | 12       | SLOW |              |          |          |
| QX<14>                             | IOB              | OUTPUT    | LVCMOS25
|       | 12       | SLOW |              |          |          |
```

```
| QX<15>                                   | IOB                 | OUTPUT    | LVCMOS25
|        | 12        | SLOW |                 |            |           |
| QX<16>                                   | IOB                 | OUTPUT    | LVCMOS25
|        | 12        | SLOW |                 |            |           |
| QX<17>                                   | IOB                 | OUTPUT    | LVCMOS25
|        | 12        | SLOW |                 |            |           |
| QX<18>                                   | IOB                 | OUTPUT    | LVCMOS25
|        | 12        | SLOW |                 |            |           |
| QX<19>                                   | IOB                 | OUTPUT    | LVCMOS25
|        | 12        | SLOW |                 |            |           |
| QX<20>                                   | IOB                 | OUTPUT    | LVCMOS25
|        | 12        | SLOW |                 |            |           |
| QX<21>                                   | IOB                 | OUTPUT    | LVCMOS25
|        | 12        | SLOW |                 |            |           |
| QX<22>                                   | IOB                 | OUTPUT    | LVCMOS25
|        | 12        | SLOW |                 |            |           |
| QX<23>                                   | IOB                 | OUTPUT    | LVCMOS25
|        | 12        | SLOW |                 |            |           |
| QX<24>                                   | IOB                 | OUTPUT    | LVCMOS25
|        | 12        | SLOW |                 |            |           |
| QX<25>                                   | IOB                 | OUTPUT    | LVCMOS25
|        | 12        | SLOW |                 |            |           |
| QX<26>                                   | IOB                 | OUTPUT    | LVCMOS25
|        | 12        | SLOW |                 |            |           |
| QX<27>                                   | IOB                 | OUTPUT    | LVCMOS25
|        | 12        | SLOW |                 |            |           |
| QX<28>                                   | IOB                 | OUTPUT    | LVCMOS25
|        | 12        | SLOW |                 |            |           |
| QX<29>                                   | IOB                 | OUTPUT    | LVCMOS25
|        | 12        | SLOW |                 |            |           |
| QX<30>                                   | IOB                 | OUTPUT    | LVCMOS25
|        | 12        | SLOW |                 |            |           |
| QX<31>                                   | IOB                 | OUTPUT    | LVCMOS25
|        | 12        | SLOW |                 |            |           |
| QX<32>                                   | IOB                 | OUTPUT    | LVCMOS25
|        | 12        | SLOW |                 |            |           |
| QX<33>                                   | IOB                 | OUTPUT    | LVCMOS25
|        | 12        | SLOW |                 |            |           |
| QX<34>                                   | IOB                 | OUTPUT    | LVCMOS25
|        | 12        | SLOW |                 |            |           |
| QX<35>                                   | IOB                 | OUTPUT    | LVCMOS25
|        | 12        | SLOW |                 |            |           |
| QX<36>                                   | IOB                 | OUTPUT    | LVCMOS25
|        | 12        | SLOW |                 |            |           |
| QX<37>                                   | IOB                 | OUTPUT    | LVCMOS25
|        | 12        | SLOW |                 |            |           |
| QX<38>                                   | IOB                 | OUTPUT    | LVCMOS25
|        | 12        | SLOW |                 |            |           |
| QX<39>                                   | IOB                 | OUTPUT    | LVCMOS25
|        | 12        | SLOW |                 |            |           |
| QX<40>                                   | IOB                 | OUTPUT    | LVCMOS25
|        | 12        | SLOW |                 |            |           |
| QX<41>                                   | IOB                 | OUTPUT    | LVCMOS25
|        | 12        | SLOW |                 |            |           |
| QX<42>                                   | IOB                 | OUTPUT    | LVCMOS25
|        | 12        | SLOW |                 |            |           |
| QX<43>                                   | IOB                 | OUTPUT    | LVCMOS25
|        | 12        | SLOW |                 |            |           |
| QX<44>                                   | IOB                 | OUTPUT    | LVCMOS25
|        | 12        | SLOW |                 |            |           |
| QX<45>                                   | IOB                 | OUTPUT    | LVCMOS25
|        | 12        | SLOW |                 |            |           |
| QX<46>                                   | IOB                 | OUTPUT    | LVCMOS25
|        | 12        | SLOW |                 |            |           |
| QX<47>                                   | IOB                 | OUTPUT    | LVCMOS25
|        | 12        | SLOW |                 |            |           |
| QX<48>                                   | IOB                 | OUTPUT    | LVCMOS25
|        | 12        | SLOW |                 |            |           |
| QX<49>                                   | IOB                 | OUTPUT    | LVCMOS25
|        | 12        | SLOW |                 |            |           |
| QX<50>                                   | IOB                 | OUTPUT    | LVCMOS25
```

```
|        | 12        | SLOW |                  |            |          |
| QX<51>                               | IOB              | OUTPUT     | LVCMOS25
|        | 12        | SLOW |                  |            |          |
| QX<52>                               | IOB              | OUTPUT     | LVCMOS25
|        | 12        | SLOW |                  |            |          |
| QX<53>                               | IOB              | OUTPUT     | LVCMOS25
|        | 12        | SLOW |                  |            |          |
| QX<54>                               | IOB              | OUTPUT     | LVCMOS25
|        | 12        | SLOW |                  |            |          |
| QX<55>                               | IOB              | OUTPUT     | LVCMOS25
|        | 12        | SLOW |                  |            |          |
| QX<56>                               | IOB              | OUTPUT     | LVCMOS25
|        | 12        | SLOW |                  |            |          |
| QX<57>                               | IOB              | OUTPUT     | LVCMOS25
|        | 12        | SLOW |                  |            |          |
| QX<58>                               | IOB              | OUTPUT     | LVCMOS25
|        | 12        | SLOW |                  |            |          |
| QX<59>                               | IOB              | OUTPUT     | LVCMOS25
|        | 12        | SLOW |                  |            |          |
| QX<60>                               | IOB              | OUTPUT     | LVCMOS25
|        | 12        | SLOW |                  |            |          |
| QX<61>                               | IOB              | OUTPUT     | LVCMOS25
|        | 12        | SLOW |                  |            |          |
| QX<62>                               | IOB              | OUTPUT     | LVCMOS25
|        | 12        | SLOW |                  |            |          |
| QX<63>                               | IOB              | OUTPUT     | LVCMOS25
|        | 12        | SLOW |                  |            |          |
| QX<64>                               | IOB              | OUTPUT     | LVCMOS25
|        | 12        | SLOW |                  |            |          |
| QX<65>                               | IOB              | OUTPUT     | LVCMOS25
|        | 12        | SLOW |                  |            |          |
| QX<66>                               | IOB              | OUTPUT     | LVCMOS25
|        | 12        | SLOW |                  |            |          |
| QX<67>                               | IOB              | OUTPUT     | LVCMOS25
|        | 12        | SLOW |                  |            |          |
| QX<68>                               | IOB              | OUTPUT     | LVCMOS25
|        | 12        | SLOW |                  |            |          |
| QX<69>                               | IOB              | OUTPUT     | LVCMOS25
|        | 12        | SLOW |                  |            |          |
| QX<70>                               | IOB              | OUTPUT     | LVCMOS25
|        | 12        | SLOW |                  |            |          |
| QX<71>                               | IOB              | OUTPUT     | LVCMOS25
|        | 12        | SLOW |                  |            |          |
| QX<72>                               | IOB              | OUTPUT     | LVCMOS25
|        | 12        | SLOW |                  |            |          |
| QX<73>                               | IOB              | OUTPUT     | LVCMOS25
|        | 12        | SLOW |                  |            |          |
| QX<74>                               | IOB              | OUTPUT     | LVCMOS25
|        | 12        | SLOW |                  |            |          |
| QX<75>                               | IOB              | OUTPUT     | LVCMOS25
|        | 12        | SLOW |                  |            |          |
| QX<76>                               | IOB              | OUTPUT     | LVCMOS25
|        | 12        | SLOW |                  |            |          |
| QX<77>                               | IOB              | OUTPUT     | LVCMOS25
|        | 12        | SLOW |                  |            |          |
| QX<78>                               | IOB              | OUTPUT     | LVCMOS25
|        | 12        | SLOW |                  |            |          |
| QX<79>                               | IOB              | OUTPUT     | LVCMOS25
|        | 12        | SLOW |                  |            |          |
| QX<80>                               | IOB              | OUTPUT     | LVCMOS25
|        | 12        | SLOW |                  |            |          |
| QX<81>                               | IOB              | OUTPUT     | LVCMOS25
|        | 12        | SLOW |                  |            |          |
| QX<82>                               | IOB              | OUTPUT     | LVCMOS25
|        | 12        | SLOW |                  |            |          |
| QX<83>                               | IOB              | OUTPUT     | LVCMOS25
|        | 12        | SLOW |                  |            |          |
| QX<84>                               | IOB              | OUTPUT     | LVCMOS25
|        | 12        | SLOW |                  |            |          |
| QX<85>                               | IOB              | OUTPUT     | LVCMOS25
|        | 12        | SLOW |                  |            |          |
```

```
| QX<86>                                  | IOB                  | OUTPUT      | LVCMOS25
|       | 12        | SLOW |              |           |          |
| QX<87>                                  | IOB                  | OUTPUT      | LVCMOS25
|       | 12        | SLOW |              |           |          |
| QX<88>                                  | IOB                  | OUTPUT      | LVCMOS25
|       | 12        | SLOW |              |           |          |
| QX<89>                                  | IOB                  | OUTPUT      | LVCMOS25
|       | 12        | SLOW |              |           |          |
| QX<90>                                  | IOB                  | OUTPUT      | LVCMOS25
|       | 12        | SLOW |              |           |          |
| QX<91>                                  | IOB                  | OUTPUT      | LVCMOS25
|       | 12        | SLOW |              |           |          |
| QX<92>                                  | IOB                  | OUTPUT      | LVCMOS25
|       | 12        | SLOW |              |           |          |
| QX<93>                                  | IOB                  | OUTPUT      | LVCMOS25
|       | 12        | SLOW |              |           |          |
| QX<94>                                  | IOB                  | OUTPUT      | LVCMOS25
|       | 12        | SLOW |              |           |          |
| QX<95>                                  | IOB                  | OUTPUT      | LVCMOS25
|       | 12        | SLOW |              |           |          |
| QX<96>                                  | IOB                  | OUTPUT      | LVCMOS25
|       | 12        | SLOW |              |           |          |
| QX<97>                                  | IOB                  | OUTPUT      | LVCMOS25
|       | 12        | SLOW |              |           |          |
| QX<98>                                  | IOB                  | OUTPUT      | LVCMOS25
|       | 12        | SLOW |              |           |          |
| QX<99>                                  | IOB                  | OUTPUT      | LVCMOS25
|       | 12        | SLOW |              |           |          |
| QX<100>                                 | IOB                  | OUTPUT      | LVCMOS25
|       | 12        | SLOW |              |           |          |
| QX<101>                                 | IOB                  | OUTPUT      | LVCMOS25
|       | 12        | SLOW |              |           |          |
| QX<102>                                 | IOB                  | OUTPUT      | LVCMOS25
|       | 12        | SLOW |              |           |          |
| QX<103>                                 | IOB                  | OUTPUT      | LVCMOS25
|       | 12        | SLOW |              |           |          |
| QX<104>                                 | IOB                  | OUTPUT      | LVCMOS25
|       | 12        | SLOW |              |           |          |
| QX<105>                                 | IOB                  | OUTPUT      | LVCMOS25
|       | 12        | SLOW |              |           |          |
| QX<106>                                 | IOB                  | OUTPUT      | LVCMOS25
|       | 12        | SLOW |              |           |          |
| QX<107>                                 | IOB                  | OUTPUT      | LVCMOS25
|       | 12        | SLOW |              |           |          |
| QX<108>                                 | IOB                  | OUTPUT      | LVCMOS25
|       | 12        | SLOW |              |           |          |
| QX<109>                                 | IOB                  | OUTPUT      | LVCMOS25
|       | 12        | SLOW |              |           |          |
| QX<110>                                 | IOB                  | OUTPUT      | LVCMOS25
|       | 12        | SLOW |              |           |          |
| QX<111>                                 | IOB                  | OUTPUT      | LVCMOS25
|       | 12        | SLOW |              |           |          |
| QX<112>                                 | IOB                  | OUTPUT      | LVCMOS25
|       | 12        | SLOW |              |           |          |
| QX<113>                                 | IOB                  | OUTPUT      | LVCMOS25
|       | 12        | SLOW |              |           |          |
| QX<114>                                 | IOB                  | OUTPUT      | LVCMOS25
|       | 12        | SLOW |              |           |          |
| QX<115>                                 | IOB                  | OUTPUT      | LVCMOS25
|       | 12        | SLOW |              |           |          |
| QX<116>                                 | IOB                  | OUTPUT      | LVCMOS25
|       | 12        | SLOW |              |           |          |
| QX<117>                                 | IOB                  | OUTPUT      | LVCMOS25
|       | 12        | SLOW |              |           |          |
| QX<118>                                 | IOB                  | OUTPUT      | LVCMOS25
|       | 12        | SLOW |              |           |          |
| QX<119>                                 | IOB                  | OUTPUT      | LVCMOS25
|       | 12        | SLOW |              |           |          |
| QX<120>                                 | IOB                  | OUTPUT      | LVCMOS25
|       | 12        | SLOW |              |           |          |
| QX<121>                                 | IOB                  | OUTPUT      | LVCMOS25
```

```
|        | 12        | SLOW |              |          |        |
| QX<122>                            | IOB              | OUTPUT     | LVCMOS25
|        | 12        | SLOW |              |          |        |
| QX<123>                            | IOB              | OUTPUT     | LVCMOS25
|        | 12        | SLOW |              |          |        |
| QX<124>                            | IOB              | OUTPUT     | LVCMOS25
|        | 12        | SLOW |              |          |        |
| QX<125>                            | IOB              | OUTPUT     | LVCMOS25
|        | 12        | SLOW |              |          |        |
| QX<126>                            | IOB              | OUTPUT     | LVCMOS25
|        | 12        | SLOW |              |          |        |
| QX<127>                            | IOB              | OUTPUT     | LVCMOS25
|        | 12        | SLOW |              |          |        |
| QX<128>                            | IOB              | OUTPUT     | LVCMOS25
|        | 12        | SLOW |              |          |        |
| QX<129>                            | IOB              | OUTPUT     | LVCMOS25
|        | 12        | SLOW |              |          |        |
| QX<130>                            | IOB              | OUTPUT     | LVCMOS25
|        | 12        | SLOW |              |          |        |
| QX<131>                            | IOB              | OUTPUT     | LVCMOS25
|        | 12        | SLOW |              |          |        |
| QX<132>                            | IOB              | OUTPUT     | LVCMOS25
|        | 12        | SLOW |              |          |        |
| QX<133>                            | IOB              | OUTPUT     | LVCMOS25
|        | 12        | SLOW |              |          |        |
| QX<134>                            | IOB              | OUTPUT     | LVCMOS25
|        | 12        | SLOW |              |          |        |
| QX<135>                            | IOB              | OUTPUT     | LVCMOS25
|        | 12        | SLOW |              |          |        |
| QX<136>                            | IOB              | OUTPUT     | LVCMOS25
|        | 12        | SLOW |              |          |        |
| QX<137>                            | IOB              | OUTPUT     | LVCMOS25
|        | 12        | SLOW |              |          |        |
| QX<138>                            | IOB              | OUTPUT     | LVCMOS25
|        | 12        | SLOW |              |          |        |
| QX<139>                            | IOB              | OUTPUT     | LVCMOS25
|        | 12        | SLOW |              |          |        |
| QX<140>                            | IOB              | OUTPUT     | LVCMOS25
|        | 12        | SLOW |              |          |        |
| QX<141>                            | IOB              | OUTPUT     | LVCMOS25
|        | 12        | SLOW |              |          |        |
| QX<142>                            | IOB              | OUTPUT     | LVCMOS25
|        | 12        | SLOW |              |          |        |
| QX<143>                            | IOB              | OUTPUT     | LVCMOS25
|        | 12        | SLOW |              |          |        |
| QX<144>                            | IOB              | OUTPUT     | LVCMOS25
|        | 12        | SLOW |              |          |        |
| QX<145>                            | IOB              | OUTPUT     | LVCMOS25
|        | 12        | SLOW |              |          |        |
| QX<146>                            | IOB              | OUTPUT     | LVCMOS25
|        | 12        | SLOW |              |          |        |
| QX<147>                            | IOB              | OUTPUT     | LVCMOS25
|        | 12        | SLOW |              |          |        |
| QX<148>                            | IOB              | OUTPUT     | LVCMOS25
|        | 12        | SLOW |              |          |        |
| QX<149>                            | IOB              | OUTPUT     | LVCMOS25
|        | 12        | SLOW |              |          |        |
| QX<150>                            | IOB              | OUTPUT     | LVCMOS25
|        | 12        | SLOW |              |          |        |
| QX<151>                            | IOB              | OUTPUT     | LVCMOS25
|        | 12        | SLOW |              |          |        |
| QX<152>                            | IOB              | OUTPUT     | LVCMOS25
|        | 12        | SLOW |              |          |        |
| QX<153>                            | IOB              | OUTPUT     | LVCMOS25
|        | 12        | SLOW |              |          |        |
| QX<154>                            | IOB              | OUTPUT     | LVCMOS25
|        | 12        | SLOW |              |          |        |
| QX<155>                            | IOB              | OUTPUT     | LVCMOS25
|        | 12        | SLOW |              |          |        |
| QX<156>                            | IOB              | OUTPUT     | LVCMOS25
|        | 12        | SLOW |              |          |        |
```

```
| QX<157>                                 | IOB                | OUTPUT    | LVCMOS25
|        | 12         | SLOW |                |            |
| QX<158>                                 | IOB                | OUTPUT    | LVCMOS25
|        | 12         | SLOW |                |            |
| QX<159>                                 | IOB                | OUTPUT    | LVCMOS25
|        | 12         | SLOW |                |            |
| QX<160>                                 | IOB                | OUTPUT    | LVCMOS25
|        | 12         | SLOW |                |            |
| QX<161>                                 | IOB                | OUTPUT    | LVCMOS25
|        | 12         | SLOW |                |            |
| QX<162>                                 | IOB                | OUTPUT    | LVCMOS25
|        | 12         | SLOW |                |            |
| QY<0>                                   | IOB                | OUTPUT    | LVCMOS25
|        | 12         | SLOW |                |            |
| QY<1>                                   | IOB                | OUTPUT    | LVCMOS25
|        | 12         | SLOW |                |            |
| QY<2>                                   | IOB                | OUTPUT    | LVCMOS25
|        | 12         | SLOW |                |            |
| QY<3>                                   | IOB                | OUTPUT    | LVCMOS25
|        | 12         | SLOW |                |            |
| QY<4>                                   | IOB                | OUTPUT    | LVCMOS25
|        | 12         | SLOW |                |            |
| QY<5>                                   | IOB                | OUTPUT    | LVCMOS25
|        | 12         | SLOW |                |            |
| QY<6>                                   | IOB                | OUTPUT    | LVCMOS25
|        | 12         | SLOW |                |            |
| QY<7>                                   | IOB                | OUTPUT    | LVCMOS25
|        | 12         | SLOW |                |            |
| QY<8>                                   | IOB                | OUTPUT    | LVCMOS25
|        | 12         | SLOW |                |            |
| QY<9>                                   | IOB                | OUTPUT    | LVCMOS25
|        | 12         | SLOW |                |            |
| QY<10>                                  | IOB                | OUTPUT    | LVCMOS25
|        | 12         | SLOW |                |            |
| QY<11>                                  | IOB                | OUTPUT    | LVCMOS25
|        | 12         | SLOW |                |            |
| QY<12>                                  | IOB                | OUTPUT    | LVCMOS25
|        | 12         | SLOW |                |            |
| QY<13>                                  | IOB                | OUTPUT    | LVCMOS25
|        | 12         | SLOW |                |            |
| QY<14>                                  | IOB                | OUTPUT    | LVCMOS25
|        | 12         | SLOW |                |            |
| QY<15>                                  | IOB                | OUTPUT    | LVCMOS25
|        | 12         | SLOW |                |            |
| QY<16>                                  | IOB                | OUTPUT    | LVCMOS25
|        | 12         | SLOW |                |            |
| QY<17>                                  | IOB                | OUTPUT    | LVCMOS25
|        | 12         | SLOW |                |            |
| QY<18>                                  | IOB                | OUTPUT    | LVCMOS25
|        | 12         | SLOW |                |            |
| QY<19>                                  | IOB                | OUTPUT    | LVCMOS25
|        | 12         | SLOW |                |            |
| QY<20>                                  | IOB                | OUTPUT    | LVCMOS25
|        | 12         | SLOW |                |            |
| QY<21>                                  | IOB                | OUTPUT    | LVCMOS25
|        | 12         | SLOW |                |            |
| QY<22>                                  | IOB                | OUTPUT    | LVCMOS25
|        | 12         | SLOW |                |            |
| QY<23>                                  | IOB                | OUTPUT    | LVCMOS25
|        | 12         | SLOW |                |            |
| QY<24>                                  | IOB                | OUTPUT    | LVCMOS25
|        | 12         | SLOW |                |            |
| QY<25>                                  | IOB                | OUTPUT    | LVCMOS25
|        | 12         | SLOW |                |            |
| QY<26>                                  | IOB                | OUTPUT    | LVCMOS25
|        | 12         | SLOW |                |            |
| QY<27>                                  | IOB                | OUTPUT    | LVCMOS25
|        | 12         | SLOW |                |            |
| QY<28>                                  | IOB                | OUTPUT    | LVCMOS25
|        | 12         | SLOW |                |            |
| QY<29>                                  | IOB                | OUTPUT    | LVCMOS25
```

```
|       | 12       | SLOW |             |          |
| QY<30>                           | IOB           | OUTPUT    | LVCMOS25
|       | 12       | SLOW |             |          |
| QY<31>                           | IOB           | OUTPUT    | LVCMOS25
|       | 12       | SLOW |             |          |
| QY<32>                           | IOB           | OUTPUT    | LVCMOS25
|       | 12       | SLOW |             |          |
| QY<33>                           | IOB           | OUTPUT    | LVCMOS25
|       | 12       | SLOW |             |          |
| QY<34>                           | IOB           | OUTPUT    | LVCMOS25
|       | 12       | SLOW |             |          |
| QY<35>                           | IOB           | OUTPUT    | LVCMOS25
|       | 12       | SLOW |             |          |
| QY<36>                           | IOB           | OUTPUT    | LVCMOS25
|       | 12       | SLOW |             |          |
| QY<37>                           | IOB           | OUTPUT    | LVCMOS25
|       | 12       | SLOW |             |          |
| QY<38>                           | IOB           | OUTPUT    | LVCMOS25
|       | 12       | SLOW |             |          |
| QY<39>                           | IOB           | OUTPUT    | LVCMOS25
|       | 12       | SLOW |             |          |
| QY<40>                           | IOB           | OUTPUT    | LVCMOS25
|       | 12       | SLOW |             |          |
| QY<41>                           | IOB           | OUTPUT    | LVCMOS25
|       | 12       | SLOW |             |          |
| QY<42>                           | IOB           | OUTPUT    | LVCMOS25
|       | 12       | SLOW |             |          |
| QY<43>                           | IOB           | OUTPUT    | LVCMOS25
|       | 12       | SLOW |             |          |
| QY<44>                           | IOB           | OUTPUT    | LVCMOS25
|       | 12       | SLOW |             |          |
| QY<45>                           | IOB           | OUTPUT    | LVCMOS25
|       | 12       | SLOW |             |          |
| QY<46>                           | IOB           | OUTPUT    | LVCMOS25
|       | 12       | SLOW |             |          |
| QY<47>                           | IOB           | OUTPUT    | LVCMOS25
|       | 12       | SLOW |             |          |
| QY<48>                           | IOB           | OUTPUT    | LVCMOS25
|       | 12       | SLOW |             |          |
| QY<49>                           | IOB           | OUTPUT    | LVCMOS25
|       | 12       | SLOW |             |          |
| QY<50>                           | IOB           | OUTPUT    | LVCMOS25
|       | 12       | SLOW |             |          |
| QY<51>                           | IOB           | OUTPUT    | LVCMOS25
|       | 12       | SLOW |             |          |
| QY<52>                           | IOB           | OUTPUT    | LVCMOS25
|       | 12       | SLOW |             |          |
| QY<53>                           | IOB           | OUTPUT    | LVCMOS25
|       | 12       | SLOW |             |          |
| QY<54>                           | IOB           | OUTPUT    | LVCMOS25
|       | 12       | SLOW |             |          |
| QY<55>                           | IOB           | OUTPUT    | LVCMOS25
|       | 12       | SLOW |             |          |
| QY<56>                           | IOB           | OUTPUT    | LVCMOS25
|       | 12       | SLOW |             |          |
| QY<57>                           | IOB           | OUTPUT    | LVCMOS25
|       | 12       | SLOW |             |          |
| QY<58>                           | IOB           | OUTPUT    | LVCMOS25
|       | 12       | SLOW |             |          |
| QY<59>                           | IOB           | OUTPUT    | LVCMOS25
|       | 12       | SLOW |             |          |
| QY<60>                           | IOB           | OUTPUT    | LVCMOS25
|       | 12       | SLOW |             |          |
| QY<61>                           | IOB           | OUTPUT    | LVCMOS25
|       | 12       | SLOW |             |          |
| QY<62>                           | IOB           | OUTPUT    | LVCMOS25
|       | 12       | SLOW |             |          |
| QY<63>                           | IOB           | OUTPUT    | LVCMOS25
|       | 12       | SLOW |             |          |
| QY<64>                           | IOB           | OUTPUT    | LVCMOS25
|       | 12       | SLOW |             |          |
```

```
| QY<65>                              | IOB                | OUTPUT     | LVCMOS25
|       | 12        | SLOW |          |          |          |
| QY<66>                              | IOB                | OUTPUT     | LVCMOS25
|       | 12        | SLOW |          |          |          |
| QY<67>                              | IOB                | OUTPUT     | LVCMOS25
|       | 12        | SLOW |          |          |          |
| QY<68>                              | IOB                | OUTPUT     | LVCMOS25
|       | 12        | SLOW |          |          |          |
| QY<69>                              | IOB                | OUTPUT     | LVCMOS25
|       | 12        | SLOW |          |          |          |
| QY<70>                              | IOB                | OUTPUT     | LVCMOS25
|       | 12        | SLOW |          |          |          |
| QY<71>                              | IOB                | OUTPUT     | LVCMOS25
|       | 12        | SLOW |          |          |          |
| QY<72>                              | IOB                | OUTPUT     | LVCMOS25
|       | 12        | SLOW |          |          |          |
| QY<73>                              | IOB                | OUTPUT     | LVCMOS25
|       | 12        | SLOW |          |          |          |
| QY<74>                              | IOB                | OUTPUT     | LVCMOS25
|       | 12        | SLOW |          |          |          |
| QY<75>                              | IOB                | OUTPUT     | LVCMOS25
|       | 12        | SLOW |          |          |          |
| QY<76>                              | IOB                | OUTPUT     | LVCMOS25
|       | 12        | SLOW |          |          |          |
| QY<77>                              | IOB                | OUTPUT     | LVCMOS25
|       | 12        | SLOW |          |          |          |
| QY<78>                              | IOB                | OUTPUT     | LVCMOS25
|       | 12        | SLOW |          |          |          |
| QY<79>                              | IOB                | OUTPUT     | LVCMOS25
|       | 12        | SLOW |          |          |          |
| QY<80>                              | IOB                | OUTPUT     | LVCMOS25
|       | 12        | SLOW |          |          |          |
| QY<81>                              | IOB                | OUTPUT     | LVCMOS25
|       | 12        | SLOW |          |          |          |
| QY<82>                              | IOB                | OUTPUT     | LVCMOS25
|       | 12        | SLOW |          |          |          |
| QY<83>                              | IOB                | OUTPUT     | LVCMOS25
|       | 12        | SLOW |          |          |          |
| QY<84>                              | IOB                | OUTPUT     | LVCMOS25
|       | 12        | SLOW |          |          |          |
| QY<85>                              | IOB                | OUTPUT     | LVCMOS25
|       | 12        | SLOW |          |          |          |
| QY<86>                              | IOB                | OUTPUT     | LVCMOS25
|       | 12        | SLOW |          |          |          |
| QY<87>                              | IOB                | OUTPUT     | LVCMOS25
|       | 12        | SLOW |          |          |          |
| QY<88>                              | IOB                | OUTPUT     | LVCMOS25
|       | 12        | SLOW |          |          |          |
| QY<89>                              | IOB                | OUTPUT     | LVCMOS25
|       | 12        | SLOW |          |          |          |
| QY<90>                              | IOB                | OUTPUT     | LVCMOS25
|       | 12        | SLOW |          |          |          |
| QY<91>                              | IOB                | OUTPUT     | LVCMOS25
|       | 12        | SLOW |          |          |          |
| QY<92>                              | IOB                | OUTPUT     | LVCMOS25
|       | 12        | SLOW |          |          |          |
| QY<93>                              | IOB                | OUTPUT     | LVCMOS25
|       | 12        | SLOW |          |          |          |
| QY<94>                              | IOB                | OUTPUT     | LVCMOS25
|       | 12        | SLOW |          |          |          |
| QY<95>                              | IOB                | OUTPUT     | LVCMOS25
|       | 12        | SLOW |          |          |          |
| QY<96>                              | IOB                | OUTPUT     | LVCMOS25
|       | 12        | SLOW |          |          |          |
| QY<97>                              | IOB                | OUTPUT     | LVCMOS25
|       | 12        | SLOW |          |          |          |
| QY<98>                              | IOB                | OUTPUT     | LVCMOS25
|       | 12        | SLOW |          |          |          |
| QY<99>                              | IOB                | OUTPUT     | LVCMOS25
|       | 12        | SLOW |          |          |          |
| QY<100>                             | IOB                | OUTPUT     | LVCMOS25
```

```
|        | 12        | SLOW |                  |         |          |
| QY<101>                                  | IOB              | OUTPUT    | LVCMOS25
|        | 12        | SLOW |                  |         |          |
| QY<102>                                  | IOB              | OUTPUT    | LVCMOS25
|        | 12        | SLOW |                  |         |          |
| QY<103>                                  | IOB              | OUTPUT    | LVCMOS25
|        | 12        | SLOW |                  |         |          |
| QY<104>                                  | IOB              | OUTPUT    | LVCMOS25
|        | 12        | SLOW |                  |         |          |
| QY<105>                                  | IOB              | OUTPUT    | LVCMOS25
|        | 12        | SLOW |                  |         |          |
| QY<106>                                  | IOB              | OUTPUT    | LVCMOS25
|        | 12        | SLOW |                  |         |          |
| QY<107>                                  | IOB              | OUTPUT    | LVCMOS25
|        | 12        | SLOW |                  |         |          |
| QY<108>                                  | IOB              | OUTPUT    | LVCMOS25
|        | 12        | SLOW |                  |         |          |
| QY<109>                                  | IOB              | OUTPUT    | LVCMOS25
|        | 12        | SLOW |                  |         |          |
| QY<110>                                  | IOB              | OUTPUT    | LVCMOS25
|        | 12        | SLOW |                  |         |          |
| QY<111>                                  | IOB              | OUTPUT    | LVCMOS25
|        | 12        | SLOW |                  |         |          |
| QY<112>                                  | IOB              | OUTPUT    | LVCMOS25
|        | 12        | SLOW |                  |         |          |
| QY<113>                                  | IOB              | OUTPUT    | LVCMOS25
|        | 12        | SLOW |                  |         |          |
| QY<114>                                  | IOB              | OUTPUT    | LVCMOS25
|        | 12        | SLOW |                  |         |          |
| QY<115>                                  | IOB              | OUTPUT    | LVCMOS25
|        | 12        | SLOW |                  |         |          |
| QY<116>                                  | IOB              | OUTPUT    | LVCMOS25
|        | 12        | SLOW |                  |         |          |
| QY<117>                                  | IOB              | OUTPUT    | LVCMOS25
|        | 12        | SLOW |                  |         |          |
| QY<118>                                  | IOB              | OUTPUT    | LVCMOS25
|        | 12        | SLOW |                  |         |          |
| QY<119>                                  | IOB              | OUTPUT    | LVCMOS25
|        | 12        | SLOW |                  |         |          |
| QY<120>                                  | IOB              | OUTPUT    | LVCMOS25
|        | 12        | SLOW |                  |         |          |
| QY<121>                                  | IOB              | OUTPUT    | LVCMOS25
|        | 12        | SLOW |                  |         |          |
| QY<122>                                  | IOB              | OUTPUT    | LVCMOS25
|        | 12        | SLOW |                  |         |          |
| QY<123>                                  | IOB              | OUTPUT    | LVCMOS25
|        | 12        | SLOW |                  |         |          |
| QY<124>                                  | IOB              | OUTPUT    | LVCMOS25
|        | 12        | SLOW |                  |         |          |
| QY<125>                                  | IOB              | OUTPUT    | LVCMOS25
|        | 12        | SLOW |                  |         |          |
| QY<126>                                  | IOB              | OUTPUT    | LVCMOS25
|        | 12        | SLOW |                  |         |          |
| QY<127>                                  | IOB              | OUTPUT    | LVCMOS25
|        | 12        | SLOW |                  |         |          |
| QY<128>                                  | IOB              | OUTPUT    | LVCMOS25
|        | 12        | SLOW |                  |         |          |
| QY<129>                                  | IOB              | OUTPUT    | LVCMOS25
|        | 12        | SLOW |                  |         |          |
| QY<130>                                  | IOB              | OUTPUT    | LVCMOS25
|        | 12        | SLOW |                  |         |          |
| QY<131>                                  | IOB              | OUTPUT    | LVCMOS25
|        | 12        | SLOW |                  |         |          |
| QY<132>                                  | IOB              | OUTPUT    | LVCMOS25
|        | 12        | SLOW |                  |         |          |
| QY<133>                                  | IOB              | OUTPUT    | LVCMOS25
|        | 12        | SLOW |                  |         |          |
| QY<134>                                  | IOB              | OUTPUT    | LVCMOS25
|        | 12        | SLOW |                  |         |          |
| QY<135>                                  | IOB              | OUTPUT    | LVCMOS25
|        | 12        | SLOW |                  |         |          |
```

```
| QY<136>                                | IOB              | OUTPUT    | LVCMOS25
|        | 12       | SLOW |                |            |          |
| QY<137>                                | IOB              | OUTPUT    | LVCMOS25
|        | 12       | SLOW |                |            |          |
| QY<138>                                | IOB              | OUTPUT    | LVCMOS25
|        | 12       | SLOW |                |            |          |
| QY<139>                                | IOB              | OUTPUT    | LVCMOS25
|        | 12       | SLOW |                |            |          |
| QY<140>                                | IOB              | OUTPUT    | LVCMOS25
|        | 12       | SLOW |                |            |          |
| QY<141>                                | IOB              | OUTPUT    | LVCMOS25
|        | 12       | SLOW |                |            |          |
| QY<142>                                | IOB              | OUTPUT    | LVCMOS25
|        | 12       | SLOW |                |            |          |
| QY<143>                                | IOB              | OUTPUT    | LVCMOS25
|        | 12       | SLOW |                |            |          |
| QY<144>                                | IOB              | OUTPUT    | LVCMOS25
|        | 12       | SLOW |                |            |          |
| QY<145>                                | IOB              | OUTPUT    | LVCMOS25
|        | 12       | SLOW |                |            |          |
| QY<146>                                | IOB              | OUTPUT    | LVCMOS25
|        | 12       | SLOW |                |            |          |
| QY<147>                                | IOB              | OUTPUT    | LVCMOS25
|        | 12       | SLOW |                |            |          |
| QY<148>                                | IOB              | OUTPUT    | LVCMOS25
|        | 12       | SLOW |                |            |          |
| QY<149>                                | IOB              | OUTPUT    | LVCMOS25
|        | 12       | SLOW |                |            |          |
| QY<150>                                | IOB              | OUTPUT    | LVCMOS25
|        | 12       | SLOW |                |            |          |
| QY<151>                                | IOB              | OUTPUT    | LVCMOS25
|        | 12       | SLOW |                |            |          |
| QY<152>                                | IOB              | OUTPUT    | LVCMOS25
|        | 12       | SLOW |                |            |          |
| QY<153>                                | IOB              | OUTPUT    | LVCMOS25
|        | 12       | SLOW |                |            |          |
| QY<154>                                | IOB              | OUTPUT    | LVCMOS25
|        | 12       | SLOW |                |            |          |
| QY<155>                                | IOB              | OUTPUT    | LVCMOS25
|        | 12       | SLOW |                |            |          |
| QY<156>                                | IOB              | OUTPUT    | LVCMOS25
|        | 12       | SLOW |                |            |          |
| QY<157>                                | IOB              | OUTPUT    | LVCMOS25
|        | 12       | SLOW |                |            |          |
| QY<158>                                | IOB              | OUTPUT    | LVCMOS25
|        | 12       | SLOW |                |            |          |
| QY<159>                                | IOB              | OUTPUT    | LVCMOS25
|        | 12       | SLOW |                |            |          |
| QY<160>                                | IOB              | OUTPUT    | LVCMOS25
|        | 12       | SLOW |                |            |          |
| QY<161>                                | IOB              | OUTPUT    | LVCMOS25
|        | 12       | SLOW |                |            |          |
| QY<162>                                | IOB              | OUTPUT    | LVCMOS25
|        | 12       | SLOW |                |            |          |
| QZ<0>                                  | IOB              | OUTPUT    | LVCMOS25
|        | 12       | SLOW |                |            |          |
| QZ<1>                                  | IOB              | OUTPUT    | LVCMOS25
|        | 12       | SLOW |                |            |          |
| QZ<2>                                  | IOB              | OUTPUT    | LVCMOS25
|        | 12       | SLOW |                |            |          |
| QZ<3>                                  | IOB              | OUTPUT    | LVCMOS25
|        | 12       | SLOW |                |            |          |
| QZ<4>                                  | IOB              | OUTPUT    | LVCMOS25
|        | 12       | SLOW |                |            |          |
| QZ<5>                                  | IOB              | OUTPUT    | LVCMOS25
|        | 12       | SLOW |                |            |          |
| QZ<6>                                  | IOB              | OUTPUT    | LVCMOS25
|        | 12       | SLOW |                |            |          |
| QZ<7>                                  | IOB              | OUTPUT    | LVCMOS25
|        | 12       | SLOW |                |            |          |
| QZ<8>                                  | IOB              | OUTPUT    | LVCMOS25
```

```
|        | 12         | SLOW |                 |           |           |
| QZ<9>                                 | IOB              | OUTPUT     | LVCMOS25
|        | 12         | SLOW |                 |           |           |
| QZ<10>                                | IOB              | OUTPUT     | LVCMOS25
|        | 12         | SLOW |                 |           |           |
| QZ<11>                                | IOB              | OUTPUT     | LVCMOS25
|        | 12         | SLOW |                 |           |           |
| QZ<12>                                | IOB              | OUTPUT     | LVCMOS25
|        | 12         | SLOW |                 |           |           |
| QZ<13>                                | IOB              | OUTPUT     | LVCMOS25
|        | 12         | SLOW |                 |           |           |
| QZ<14>                                | IOB              | OUTPUT     | LVCMOS25
|        | 12         | SLOW |                 |           |           |
| QZ<15>                                | IOB              | OUTPUT     | LVCMOS25
|        | 12         | SLOW |                 |           |           |
| QZ<16>                                | IOB              | OUTPUT     | LVCMOS25
|        | 12         | SLOW |                 |           |           |
| QZ<17>                                | IOB              | OUTPUT     | LVCMOS25
|        | 12         | SLOW |                 |           |           |
| QZ<18>                                | IOB              | OUTPUT     | LVCMOS25
|        | 12         | SLOW |                 |           |           |
| QZ<19>                                | IOB              | OUTPUT     | LVCMOS25
|        | 12         | SLOW |                 |           |           |
| QZ<20>                                | IOB              | OUTPUT     | LVCMOS25
|        | 12         | SLOW |                 |           |           |
| QZ<21>                                | IOB              | OUTPUT     | LVCMOS25
|        | 12         | SLOW |                 |           |           |
| QZ<22>                                | IOB              | OUTPUT     | LVCMOS25
|        | 12         | SLOW |                 |           |           |
| QZ<23>                                | IOB              | OUTPUT     | LVCMOS25
|        | 12         | SLOW |                 |           |           |
| QZ<24>                                | IOB              | OUTPUT     | LVCMOS25
|        | 12         | SLOW |                 |           |           |
| QZ<25>                                | IOB              | OUTPUT     | LVCMOS25
|        | 12         | SLOW |                 |           |           |
| QZ<26>                                | IOB              | OUTPUT     | LVCMOS25
|        | 12         | SLOW |                 |           |           |
| QZ<27>                                | IOB              | OUTPUT     | LVCMOS25
|        | 12         | SLOW |                 |           |           |
| QZ<28>                                | IOB              | OUTPUT     | LVCMOS25
|        | 12         | SLOW |                 |           |           |
| QZ<29>                                | IOB              | OUTPUT     | LVCMOS25
|        | 12         | SLOW |                 |           |           |
| QZ<30>                                | IOB              | OUTPUT     | LVCMOS25
|        | 12         | SLOW |                 |           |           |
| QZ<31>                                | IOB              | OUTPUT     | LVCMOS25
|        | 12         | SLOW |                 |           |           |
| QZ<32>                                | IOB              | OUTPUT     | LVCMOS25
|        | 12         | SLOW |                 |           |           |
| QZ<33>                                | IOB              | OUTPUT     | LVCMOS25
|        | 12         | SLOW |                 |           |           |
| QZ<34>                                | IOB              | OUTPUT     | LVCMOS25
|        | 12         | SLOW |                 |           |           |
| QZ<35>                                | IOB              | OUTPUT     | LVCMOS25
|        | 12         | SLOW |                 |           |           |
| QZ<36>                                | IOB              | OUTPUT     | LVCMOS25
|        | 12         | SLOW |                 |           |           |
| QZ<37>                                | IOB              | OUTPUT     | LVCMOS25
|        | 12         | SLOW |                 |           |           |
| QZ<38>                                | IOB              | OUTPUT     | LVCMOS25
|        | 12         | SLOW |                 |           |           |
| QZ<39>                                | IOB              | OUTPUT     | LVCMOS25
|        | 12         | SLOW |                 |           |           |
| QZ<40>                                | IOB              | OUTPUT     | LVCMOS25
|        | 12         | SLOW |                 |           |           |
| QZ<41>                                | IOB              | OUTPUT     | LVCMOS25
|        | 12         | SLOW |                 |           |           |
| QZ<42>                                | IOB              | OUTPUT     | LVCMOS25
|        | 12         | SLOW |                 |           |           |
| QZ<43>                                | IOB              | OUTPUT     | LVCMOS25
|        | 12         | SLOW |                 |           |           |
```

```
| QZ<44>                                  | IOB                  | OUTPUT       | LVCMOS25
|        | 12          | SLOW |                 |            |           |
| QZ<45>                                  | IOB                  | OUTPUT       | LVCMOS25
|        | 12          | SLOW |                 |            |           |
| QZ<46>                                  | IOB                  | OUTPUT       | LVCMOS25
|        | 12          | SLOW |                 |            |           |
| QZ<47>                                  | IOB                  | OUTPUT       | LVCMOS25
|        | 12          | SLOW |                 |            |           |
| QZ<48>                                  | IOB                  | OUTPUT       | LVCMOS25
|        | 12          | SLOW |                 |            |           |
| QZ<49>                                  | IOB                  | OUTPUT       | LVCMOS25
|        | 12          | SLOW |                 |            |           |
| QZ<50>                                  | IOB                  | OUTPUT       | LVCMOS25
|        | 12          | SLOW |                 |            |           |
| QZ<51>                                  | IOB                  | OUTPUT       | LVCMOS25
|        | 12          | SLOW |                 |            |           |
| QZ<52>                                  | IOB                  | OUTPUT       | LVCMOS25
|        | 12          | SLOW |                 |            |           |
| QZ<53>                                  | IOB                  | OUTPUT       | LVCMOS25
|        | 12          | SLOW |                 |            |           |
| QZ<54>                                  | IOB                  | OUTPUT       | LVCMOS25
|        | 12          | SLOW |                 |            |           |
| QZ<55>                                  | IOB                  | OUTPUT       | LVCMOS25
|        | 12          | SLOW |                 |            |           |
| QZ<56>                                  | IOB                  | OUTPUT       | LVCMOS25
|        | 12          | SLOW |                 |            |           |
| QZ<57>                                  | IOB                  | OUTPUT       | LVCMOS25
|        | 12          | SLOW |                 |            |           |
| QZ<58>                                  | IOB                  | OUTPUT       | LVCMOS25
|        | 12          | SLOW |                 |            |           |
| QZ<59>                                  | IOB                  | OUTPUT       | LVCMOS25
|        | 12          | SLOW |                 |            |           |
| QZ<60>                                  | IOB                  | OUTPUT       | LVCMOS25
|        | 12          | SLOW |                 |            |           |
| QZ<61>                                  | IOB                  | OUTPUT       | LVCMOS25
|        | 12          | SLOW |                 |            |           |
| QZ<62>                                  | IOB                  | OUTPUT       | LVCMOS25
|        | 12          | SLOW |                 |            |           |
| QZ<63>                                  | IOB                  | OUTPUT       | LVCMOS25
|        | 12          | SLOW |                 |            |           |
| QZ<64>                                  | IOB                  | OUTPUT       | LVCMOS25
|        | 12          | SLOW |                 |            |           |
| QZ<65>                                  | IOB                  | OUTPUT       | LVCMOS25
|        | 12          | SLOW |                 |            |           |
| QZ<66>                                  | IOB                  | OUTPUT       | LVCMOS25
|        | 12          | SLOW |                 |            |           |
| QZ<67>                                  | IOB                  | OUTPUT       | LVCMOS25
|        | 12          | SLOW |                 |            |           |
| QZ<68>                                  | IOB                  | OUTPUT       | LVCMOS25
|        | 12          | SLOW |                 |            |           |
| QZ<69>                                  | IOB                  | OUTPUT       | LVCMOS25
|        | 12          | SLOW |                 |            |           |
| QZ<70>                                  | IOB                  | OUTPUT       | LVCMOS25
|        | 12          | SLOW |                 |            |           |
| QZ<71>                                  | IOB                  | OUTPUT       | LVCMOS25
|        | 12          | SLOW |                 |            |           |
| QZ<72>                                  | IOB                  | OUTPUT       | LVCMOS25
|        | 12          | SLOW |                 |            |           |
| QZ<73>                                  | IOB                  | OUTPUT       | LVCMOS25
|        | 12          | SLOW |                 |            |           |
| QZ<74>                                  | IOB                  | OUTPUT       | LVCMOS25
|        | 12          | SLOW |                 |            |           |
| QZ<75>                                  | IOB                  | OUTPUT       | LVCMOS25
|        | 12          | SLOW |                 |            |           |
| QZ<76>                                  | IOB                  | OUTPUT       | LVCMOS25
|        | 12          | SLOW |                 |            |           |
| QZ<77>                                  | IOB                  | OUTPUT       | LVCMOS25
|        | 12          | SLOW |                 |            |           |
| QZ<78>                                  | IOB                  | OUTPUT       | LVCMOS25
|        | 12          | SLOW |                 |            |           |
| QZ<79>                                  | IOB                  | OUTPUT       | LVCMOS25
```

```
|        | 12        | SLOW |                    |          |          |
| QZ<80>                                | IOB              | OUTPUT     | LVCMOS25
|        | 12        | SLOW |                    |          |          |
| QZ<81>                                | IOB              | OUTPUT     | LVCMOS25
|        | 12        | SLOW |                    |          |          |
| QZ<82>                                | IOB              | OUTPUT     | LVCMOS25
|        | 12        | SLOW |                    |          |          |
| QZ<83>                                | IOB              | OUTPUT     | LVCMOS25
|        | 12        | SLOW |                    |          |          |
| QZ<84>                                | IOB              | OUTPUT     | LVCMOS25
|        | 12        | SLOW |                    |          |          |
| QZ<85>                                | IOB              | OUTPUT     | LVCMOS25
|        | 12        | SLOW |                    |          |          |
| QZ<86>                                | IOB              | OUTPUT     | LVCMOS25
|        | 12        | SLOW |                    |          |          |
| QZ<87>                                | IOB              | OUTPUT     | LVCMOS25
|        | 12        | SLOW |                    |          |          |
| QZ<88>                                | IOB              | OUTPUT     | LVCMOS25
|        | 12        | SLOW |                    |          |          |
| QZ<89>                                | IOB              | OUTPUT     | LVCMOS25
|        | 12        | SLOW |                    |          |          |
| QZ<90>                                | IOB              | OUTPUT     | LVCMOS25
|        | 12        | SLOW |                    |          |          |
| QZ<91>                                | IOB              | OUTPUT     | LVCMOS25
|        | 12        | SLOW |                    |          |          |
| QZ<92>                                | IOB              | OUTPUT     | LVCMOS25
|        | 12        | SLOW |                    |          |          |
| QZ<93>                                | IOB              | OUTPUT     | LVCMOS25
|        | 12        | SLOW |                    |          |          |
| QZ<94>                                | IOB              | OUTPUT     | LVCMOS25
|        | 12        | SLOW |                    |          |          |
| QZ<95>                                | IOB              | OUTPUT     | LVCMOS25
|        | 12        | SLOW |                    |          |          |
| QZ<96>                                | IOB              | OUTPUT     | LVCMOS25
|        | 12        | SLOW |                    |          |          |
| QZ<97>                                | IOB              | OUTPUT     | LVCMOS25
|        | 12        | SLOW |                    |          |          |
| QZ<98>                                | IOB              | OUTPUT     | LVCMOS25
|        | 12        | SLOW |                    |          |          |
| QZ<99>                                | IOB              | OUTPUT     | LVCMOS25
|        | 12        | SLOW |                    |          |          |
| QZ<100>                               | IOB              | OUTPUT     | LVCMOS25
|        | 12        | SLOW |                    |          |          |
| QZ<101>                               | IOB              | OUTPUT     | LVCMOS25
|        | 12        | SLOW |                    |          |          |
| QZ<102>                               | IOB              | OUTPUT     | LVCMOS25
|        | 12        | SLOW |                    |          |          |
| QZ<103>                               | IOB              | OUTPUT     | LVCMOS25
|        | 12        | SLOW |                    |          |          |
| QZ<104>                               | IOB              | OUTPUT     | LVCMOS25
|        | 12        | SLOW |                    |          |          |
| QZ<105>                               | IOB              | OUTPUT     | LVCMOS25
|        | 12        | SLOW |                    |          |          |
| QZ<106>                               | IOB              | OUTPUT     | LVCMOS25
|        | 12        | SLOW |                    |          |          |
| QZ<107>                               | IOB              | OUTPUT     | LVCMOS25
|        | 12        | SLOW |                    |          |          |
| QZ<108>                               | IOB              | OUTPUT     | LVCMOS25
|        | 12        | SLOW |                    |          |          |
| QZ<109>                               | IOB              | OUTPUT     | LVCMOS25
|        | 12        | SLOW |                    |          |          |
| QZ<110>                               | IOB              | OUTPUT     | LVCMOS25
|        | 12        | SLOW |                    |          |          |
| QZ<111>                               | IOB              | OUTPUT     | LVCMOS25
|        | 12        | SLOW |                    |          |          |
| QZ<112>                               | IOB              | OUTPUT     | LVCMOS25
|        | 12        | SLOW |                    |          |          |
| QZ<113>                               | IOB              | OUTPUT     | LVCMOS25
|        | 12        | SLOW |                    |          |          |
| QZ<114>                               | IOB              | OUTPUT     | LVCMOS25
|        | 12        | SLOW |                    |          |          |
```

```
| QZ<115>                              | IOB                  | OUTPUT       | LVCMOS25
|        | 12         | SLOW |            |           |            |
| QZ<116>                              | IOB                  | OUTPUT       | LVCMOS25
|        | 12         | SLOW |            |           |            |
| QZ<117>                              | IOB                  | OUTPUT       | LVCMOS25
|        | 12         | SLOW |            |           |            |
| QZ<118>                              | IOB                  | OUTPUT       | LVCMOS25
|        | 12         | SLOW |            |           |            |
| QZ<119>                              | IOB                  | OUTPUT       | LVCMOS25
|        | 12         | SLOW |            |           |            |
| QZ<120>                              | IOB                  | OUTPUT       | LVCMOS25
|        | 12         | SLOW |            |           |            |
| QZ<121>                              | IOB                  | OUTPUT       | LVCMOS25
|        | 12         | SLOW |            |           |            |
| QZ<122>                              | IOB                  | OUTPUT       | LVCMOS25
|        | 12         | SLOW |            |           |            |
| QZ<123>                              | IOB                  | OUTPUT       | LVCMOS25
|        | 12         | SLOW |            |           |            |
| QZ<124>                              | IOB                  | OUTPUT       | LVCMOS25
|        | 12         | SLOW |            |           |            |
| QZ<125>                              | IOB                  | OUTPUT       | LVCMOS25
|        | 12         | SLOW |            |           |            |
| QZ<126>                              | IOB                  | OUTPUT       | LVCMOS25
|        | 12         | SLOW |            |           |            |
| QZ<127>                              | IOB                  | OUTPUT       | LVCMOS25
|        | 12         | SLOW |            |           |            |
| QZ<128>                              | IOB                  | OUTPUT       | LVCMOS25
|        | 12         | SLOW |            |           |            |
| QZ<129>                              | IOB                  | OUTPUT       | LVCMOS25
|        | 12         | SLOW |            |           |            |
| QZ<130>                              | IOB                  | OUTPUT       | LVCMOS25
|        | 12         | SLOW |            |           |            |
| QZ<131>                              | IOB                  | OUTPUT       | LVCMOS25
|        | 12         | SLOW |            |           |            |
| QZ<132>                              | IOB                  | OUTPUT       | LVCMOS25
|        | 12         | SLOW |            |           |            |
| QZ<133>                              | IOB                  | OUTPUT       | LVCMOS25
|        | 12         | SLOW |            |           |            |
| QZ<134>                              | IOB                  | OUTPUT       | LVCMOS25
|        | 12         | SLOW |            |           |            |
| QZ<135>                              | IOB                  | OUTPUT       | LVCMOS25
|        | 12         | SLOW |            |           |            |
| QZ<136>                              | IOB                  | OUTPUT       | LVCMOS25
|        | 12         | SLOW |            |           |            |
| QZ<137>                              | IOB                  | OUTPUT       | LVCMOS25
|        | 12         | SLOW |            |           |            |
| QZ<138>                              | IOB                  | OUTPUT       | LVCMOS25
|        | 12         | SLOW |            |           |            |
| QZ<139>                              | IOB                  | OUTPUT       | LVCMOS25
|        | 12         | SLOW |            |           |            |
| QZ<140>                              | IOB                  | OUTPUT       | LVCMOS25
|        | 12         | SLOW |            |           |            |
| QZ<141>                              | IOB                  | OUTPUT       | LVCMOS25
|        | 12         | SLOW |            |           |            |
| QZ<142>                              | IOB                  | OUTPUT       | LVCMOS25
|        | 12         | SLOW |            |           |            |
| QZ<143>                              | IOB                  | OUTPUT       | LVCMOS25
|        | 12         | SLOW |            |           |            |
| QZ<144>                              | IOB                  | OUTPUT       | LVCMOS25
|        | 12         | SLOW |            |           |            |
| QZ<145>                              | IOB                  | OUTPUT       | LVCMOS25
|        | 12         | SLOW |            |           |            |
| QZ<146>                              | IOB                  | OUTPUT       | LVCMOS25
|        | 12         | SLOW |            |           |            |
| QZ<147>                              | IOB                  | OUTPUT       | LVCMOS25
|        | 12         | SLOW |            |           |            |
| QZ<148>                              | IOB                  | OUTPUT       | LVCMOS25
|        | 12         | SLOW |            |           |            |
| QZ<149>                              | IOB                  | OUTPUT       | LVCMOS25
|        | 12         | SLOW |            |           |            |
| QZ<150>                              | IOB                  | OUTPUT       | LVCMOS25
```

```
|       | 12        | SLOW |             |          |         |
| QZ<151>                            | IOB              | OUTPUT    | LVCMOS25
|       | 12        | SLOW |             |          |         |
| QZ<152>                            | IOB              | OUTPUT    | LVCMOS25
|       | 12        | SLOW |             |          |         |
| QZ<153>                            | IOB              | OUTPUT    | LVCMOS25
|       | 12        | SLOW |             |          |         |
| QZ<154>                            | IOB              | OUTPUT    | LVCMOS25
|       | 12        | SLOW |             |          |         |
| QZ<155>                            | IOB              | OUTPUT    | LVCMOS25
|       | 12        | SLOW |             |          |         |
| QZ<156>                            | IOB              | OUTPUT    | LVCMOS25
|       | 12        | SLOW |             |          |         |
| QZ<157>                            | IOB              | OUTPUT    | LVCMOS25
|       | 12        | SLOW |             |          |         |
| QZ<158>                            | IOB              | OUTPUT    | LVCMOS25
|       | 12        | SLOW |             |          |         |
| QZ<159>                            | IOB              | OUTPUT    | LVCMOS25
|       | 12        | SLOW |             |          |         |
| QZ<160>                            | IOB              | OUTPUT    | LVCMOS25
|       | 12        | SLOW |             |          |         |
| QZ<161>                            | IOB              | OUTPUT    | LVCMOS25
|       | 12        | SLOW |             |          |         |
| QZ<162>                            | IOB              | OUTPUT    | LVCMOS25
|       | 12        | SLOW |             |          |         |
| clk                                | IOB              | INPUT     | LVCMOS25
|       |           |      |             |          |         |
| done                               | IOB              | OUTPUT    | LVCMOS25
|       | 12        | SLOW |             |          |         |
| key<0>                             | IOB              | INPUT     | LVCMOS25
|       |           |      |             |          |         |
| key<1>                             | IOB              | INPUT     | LVCMOS25
|       |           |      |             |          |         |
| key<2>                             | IOB              | INPUT     | LVCMOS25
|       |           |      |             |          |         |
| key<3>                             | IOB              | INPUT     | LVCMOS25
|       |           |      |             |          |         |
| key<4>                             | IOB              | INPUT     | LVCMOS25
|       |           |      |             |          |         |
| key<5>                             | IOB              | INPUT     | LVCMOS25
|       |           |      |             |          |         |
| key<6>                             | IOB              | INPUT     | LVCMOS25
|       |           |      |             |          |         |
| key<7>                             | IOB              | INPUT     | LVCMOS25
|       |           |      |             |          |         |
| key<8>                             | IOB              | INPUT     | LVCMOS25
|       |           |      |             |          |         |
| key<9>                             | IOB              | INPUT     | LVCMOS25
|       |           |      |             |          |         |
| key<10>                            | IOB              | INPUT     | LVCMOS25
|       |           |      |             |          |         |
| key<11>                            | IOB              | INPUT     | LVCMOS25
|       |           |      |             |          |         |
| key<12>                            | IOB              | INPUT     | LVCMOS25
|       |           |      |             |          |         |
| key<13>                            | IOB              | INPUT     | LVCMOS25
|       |           |      |             |          |         |
| key<14>                            | IOB              | INPUT     | LVCMOS25
|       |           |      |             |          |         |
| key<15>                            | IOB              | INPUT     | LVCMOS25
|       |           |      |             |          |         |
| key<16>                            | IOB              | INPUT     | LVCMOS25
|       |           |      |             |          |         |
| key<17>                            | IOB              | INPUT     | LVCMOS25
|       |           |      |             |          |         |
| key<18>                            | IOB              | INPUT     | LVCMOS25
|       |           |      |             |          |         |
| key<19>                            | IOB              | INPUT     | LVCMOS25
|       |           |      |             |          |         |
| key<20>                            | IOB              | INPUT     | LVCMOS25
|       |           |      |             |          |         |
```

```
| key<21>                              | IOB               | INPUT      | LVCMOS25
|         |             |        |                 |             |            |
| key<22>                              | IOB               | INPUT      | LVCMOS25
|         |             |        |                 |             |            |
| key<23>                              | IOB               | INPUT      | LVCMOS25
|         |             |        |                 |             |            |
| key<24>                              | IOB               | INPUT      | LVCMOS25
|         |             |        |                 |             |            |
| key<25>                              | IOB               | INPUT      | LVCMOS25
|         |             |        |                 |             |            |
| key<26>                              | IOB               | INPUT      | LVCMOS25
|         |             |        |                 |             |            |
| key<27>                              | IOB               | INPUT      | LVCMOS25
|         |             |        |                 |             |            |
| key<28>                              | IOB               | INPUT      | LVCMOS25
|         |             |        |                 |             |            |
| key<29>                              | IOB               | INPUT      | LVCMOS25
|         |             |        |                 |             |            |
| key<30>                              | IOB               | INPUT      | LVCMOS25
|         |             |        |                 |             |            |
| key<31>                              | IOB               | INPUT      | LVCMOS25
|         |             |        |                 |             |            |
| key<32>                              | IOB               | INPUT      | LVCMOS25
|         |             |        |                 |             |            |
| key<33>                              | IOB               | INPUT      | LVCMOS25
|         |             |        |                 |             |            |
| key<34>                              | IOB               | INPUT      | LVCMOS25
|         |             |        |                 |             |            |
| key<35>                              | IOB               | INPUT      | LVCMOS25
|         |             |        |                 |             |            |
| key<36>                              | IOB               | INPUT      | LVCMOS25
|         |             |        |                 |             |            |
| key<37>                              | IOB               | INPUT      | LVCMOS25
|         |             |        |                 |             |            |
| key<38>                              | IOB               | INPUT      | LVCMOS25
|         |             |        |                 |             |            |
| key<39>                              | IOB               | INPUT      | LVCMOS25
|         |             |        |                 |             |            |
| key<40>                              | IOB               | INPUT      | LVCMOS25
|         |             |        |                 |             |            |
| key<41>                              | IOB               | INPUT      | LVCMOS25
|         |             |        |                 |             |            |
| key<42>                              | IOB               | INPUT      | LVCMOS25
|         |             |        |                 |             |            |
| key<43>                              | IOB               | INPUT      | LVCMOS25
|         |             |        |                 |             |            |
| key<44>                              | IOB               | INPUT      | LVCMOS25
|         |             |        |                 |             |            |
| key<45>                              | IOB               | INPUT      | LVCMOS25
|         |             |        |                 |             |            |
| key<46>                              | IOB               | INPUT      | LVCMOS25
|         |             |        |                 |             |            |
| key<47>                              | IOB               | INPUT      | LVCMOS25
|         |             |        |                 |             |            |
| key<48>                              | IOB               | INPUT      | LVCMOS25
|         |             |        |                 |             |            |
| key<49>                              | IOB               | INPUT      | LVCMOS25
|         |             |        |                 |             |            |
| key<50>                              | IOB               | INPUT      | LVCMOS25
|         |             |        |                 |             |            |
| key<51>                              | IOB               | INPUT      | LVCMOS25
|         |             |        |                 |             |            |
| key<52>                              | IOB               | INPUT      | LVCMOS25
|         |             |        |                 |             |            |
| key<53>                              | IOB               | INPUT      | LVCMOS25
|         |             |        |                 |             |            |
| key<54>                              | IOB               | INPUT      | LVCMOS25
|         |             |        |                 |             |            |
| key<55>                              | IOB               | INPUT      | LVCMOS25
|         |             |        |                 |             |            |
| key<56>                              | IOB               | INPUT      | LVCMOS25
```

```
|          |             |        |                 |            |             |
| key<57>                                  | IOB           | INPUT       | LVCMOS25
|          |             |        |                 |            |             |
| key<58>                                  | IOB           | INPUT       | LVCMOS25
|          |             |        |                 |            |             |
| key<59>                                  | IOB           | INPUT       | LVCMOS25
|          |             |        |                 |            |             |
| key<60>                                  | IOB           | INPUT       | LVCMOS25
|          |             |        |                 |            |             |
| key<61>                                  | IOB           | INPUT       | LVCMOS25
|          |             |        |                 |            |             |
| key<62>                                  | IOB           | INPUT       | LVCMOS25
|          |             |        |                 |            |             |
| key<63>                                  | IOB           | INPUT       | LVCMOS25
|          |             |        |                 |            |             |
| key<64>                                  | IOB           | INPUT       | LVCMOS25
|          |             |        |                 |            |             |
| key<65>                                  | IOB           | INPUT       | LVCMOS25
|          |             |        |                 |            |             |
| key<66>                                  | IOB           | INPUT       | LVCMOS25
|          |             |        |                 |            |             |
| key<67>                                  | IOB           | INPUT       | LVCMOS25
|          |             |        |                 |            |             |
| key<68>                                  | IOB           | INPUT       | LVCMOS25
|          |             |        |                 |            |             |
| key<69>                                  | IOB           | INPUT       | LVCMOS25
|          |             |        |                 |            |             |
| key<70>                                  | IOB           | INPUT       | LVCMOS25
|          |             |        |                 |            |             |
| key<71>                                  | IOB           | INPUT       | LVCMOS25
|          |             |        |                 |            |             |
| key<72>                                  | IOB           | INPUT       | LVCMOS25
|          |             |        |                 |            |             |
| key<73>                                  | IOB           | INPUT       | LVCMOS25
|          |             |        |                 |            |             |
| key<74>                                  | IOB           | INPUT       | LVCMOS25
|          |             |        |                 |            |             |
| key<75>                                  | IOB           | INPUT       | LVCMOS25
|          |             |        |                 |            |             |
| key<76>                                  | IOB           | INPUT       | LVCMOS25
|          |             |        |                 |            |             |
| key<77>                                  | IOB           | INPUT       | LVCMOS25
|          |             |        |                 |            |             |
| key<78>                                  | IOB           | INPUT       | LVCMOS25
|          |             |        |                 |            |             |
| key<79>                                  | IOB           | INPUT       | LVCMOS25
|          |             |        |                 |            |             |
| key<80>                                  | IOB           | INPUT       | LVCMOS25
|          |             |        |                 |            |             |
| key<81>                                  | IOB           | INPUT       | LVCMOS25
|          |             |        |                 |            |             |
| key<82>                                  | IOB           | INPUT       | LVCMOS25
|          |             |        |                 |            |             |
| key<83>                                  | IOB           | INPUT       | LVCMOS25
|          |             |        |                 |            |             |
| key<84>                                  | IOB           | INPUT       | LVCMOS25
|          |             |        |                 |            |             |
| key<85>                                  | IOB           | INPUT       | LVCMOS25
|          |             |        |                 |            |             |
| key<86>                                  | IOB           | INPUT       | LVCMOS25
|          |             |        |                 |            |             |
| key<87>                                  | IOB           | INPUT       | LVCMOS25
|          |             |        |                 |            |             |
| key<88>                                  | IOB           | INPUT       | LVCMOS25
|          |             |        |                 |            |             |
| key<89>                                  | IOB           | INPUT       | LVCMOS25
|          |             |        |                 |            |             |
| key<90>                                  | IOB           | INPUT       | LVCMOS25
|          |             |        |                 |            |             |
| key<91>                                  | IOB           | INPUT       | LVCMOS25
|          |             |        |                 |            |             |
```

```
| key<92>                          | IOB              | INPUT       | LVCMOS25
|       |           |       |                |            |          |
| key<93>                          | IOB              | INPUT       | LVCMOS25
|       |           |       |                |            |          |
| key<94>                          | IOB              | INPUT       | LVCMOS25
|       |           |       |                |            |          |
| key<95>                          | IOB              | INPUT       | LVCMOS25
|       |           |       |                |            |          |
| key<96>                          | IOB              | INPUT       | LVCMOS25
|       |           |       |                |            |          |
| key<97>                          | IOB              | INPUT       | LVCMOS25
|       |           |       |                |            |          |
| key<98>                          | IOB              | INPUT       | LVCMOS25
|       |           |       |                |            |          |
| key<99>                          | IOB              | INPUT       | LVCMOS25
|       |           |       |                |            |          |
| key<100>                         | IOB              | INPUT       | LVCMOS25
|       |           |       |                |            |          |
| key<101>                         | IOB              | INPUT       | LVCMOS25
|       |           |       |                |            |          |
| key<102>                         | IOB              | INPUT       | LVCMOS25
|       |           |       |                |            |          |
| key<103>                         | IOB              | INPUT       | LVCMOS25
|       |           |       |                |            |          |
| key<104>                         | IOB              | INPUT       | LVCMOS25
|       |           |       |                |            |          |
| key<105>                         | IOB              | INPUT       | LVCMOS25
|       |           |       |                |            |          |
| key<106>                         | IOB              | INPUT       | LVCMOS25
|       |           |       |                |            |          |
| key<107>                         | IOB              | INPUT       | LVCMOS25
|       |           |       |                |            |          |
| key<108>                         | IOB              | INPUT       | LVCMOS25
|       |           |       |                |            |          |
| key<109>                         | IOB              | INPUT       | LVCMOS25
|       |           |       |                |            |          |
| key<110>                         | IOB              | INPUT       | LVCMOS25
|       |           |       |                |            |          |
| key<111>                         | IOB              | INPUT       | LVCMOS25
|       |           |       |                |            |          |
| key<112>                         | IOB              | INPUT       | LVCMOS25
|       |           |       |                |            |          |
| key<113>                         | IOB              | INPUT       | LVCMOS25
|       |           |       |                |            |          |
| key<114>                         | IOB              | INPUT       | LVCMOS25
|       |           |       |                |            |          |
| key<115>                         | IOB              | INPUT       | LVCMOS25
|       |           |       |                |            |          |
| key<116>                         | IOB              | INPUT       | LVCMOS25
|       |           |       |                |            |          |
| key<117>                         | IOB              | INPUT       | LVCMOS25
|       |           |       |                |            |          |
| key<118>                         | IOB              | INPUT       | LVCMOS25
|       |           |       |                |            |          |
| key<119>                         | IOB              | INPUT       | LVCMOS25
|       |           |       |                |            |          |
| key<120>                         | IOB              | INPUT       | LVCMOS25
|       |           |       |                |            |          |
| key<121>                         | IOB              | INPUT       | LVCMOS25
|       |           |       |                |            |          |
| key<122>                         | IOB              | INPUT       | LVCMOS25
|       |           |       |                |            |          |
| key<123>                         | IOB              | INPUT       | LVCMOS25
|       |           |       |                |            |          |
| key<124>                         | IOB              | INPUT       | LVCMOS25
|       |           |       |                |            |          |
| key<125>                         | IOB              | INPUT       | LVCMOS25
|       |           |       |                |            |          |
| key<126>                         | IOB              | INPUT       | LVCMOS25
|       |           |       |                |            |          |
| key<127>                         | IOB              | INPUT       | LVCMOS25
```

```
|          |             |        |                 |            |            |
| key<128>                          | IOB                    | INPUT        | LVCMOS25
|          |             |        |                 |            |            |
| key<129>                          | IOB                    | INPUT        | LVCMOS25
|          |             |        |                 |            |            |
| key<130>                          | IOB                    | INPUT        | LVCMOS25
|          |             |        |                 |            |            |
| key<131>                          | IOB                    | INPUT        | LVCMOS25
|          |             |        |                 |            |            |
| key<132>                          | IOB                    | INPUT        | LVCMOS25
|          |             |        |                 |            |            |
| key<133>                          | IOB                    | INPUT        | LVCMOS25
|          |             |        |                 |            |            |
| key<134>                          | IOB                    | INPUT        | LVCMOS25
|          |             |        |                 |            |            |
| key<135>                          | IOB                    | INPUT        | LVCMOS25
|          |             |        |                 |            |            |
| key<136>                          | IOB                    | INPUT        | LVCMOS25
|          |             |        |                 |            |            |
| key<137>                          | IOB                    | INPUT        | LVCMOS25
|          |             |        |                 |            |            |
| key<138>                          | IOB                    | INPUT        | LVCMOS25
|          |             |        |                 |            |            |
| key<139>                          | IOB                    | INPUT        | LVCMOS25
|          |             |        |                 |            |            |
| key<140>                          | IOB                    | INPUT        | LVCMOS25
|          |             |        |                 |            |            |
| key<141>                          | IOB                    | INPUT        | LVCMOS25
|          |             |        |                 |            |            |
| key<142>                          | IOB                    | INPUT        | LVCMOS25
|          |             |        |                 |            |            |
| key<143>                          | IOB                    | INPUT        | LVCMOS25
|          |             |        |                 |            |            |
| key<144>                          | IOB                    | INPUT        | LVCMOS25
|          |             |        |                 |            |            |
| key<145>                          | IOB                    | INPUT        | LVCMOS25
|          |             |        |                 |            |            |
| key<146>                          | IOB                    | INPUT        | LVCMOS25
|          |             |        |                 |            |            |
| key<147>                          | IOB                    | INPUT        | LVCMOS25
|          |             |        |                 |            |            |
| key<148>                          | IOB                    | INPUT        | LVCMOS25
|          |             |        |                 |            |            |
| key<149>                          | IOB                    | INPUT        | LVCMOS25
|          |             |        |                 |            |            |
| key<150>                          | IOB                    | INPUT        | LVCMOS25
|          |             |        |                 |            |            |
| key<151>                          | IOB                    | INPUT        | LVCMOS25
|          |             |        |                 |            |            |
| key<152>                          | IOB                    | INPUT        | LVCMOS25
|          |             |        |                 |            |            |
| key<153>                          | IOB                    | INPUT        | LVCMOS25
|          |             |        |                 |            |            |
| key<154>                          | IOB                    | INPUT        | LVCMOS25
|          |             |        |                 |            |            |
| key<155>                          | IOB                    | INPUT        | LVCMOS25
|          |             |        |                 |            |            |
| key<156>                          | IOB                    | INPUT        | LVCMOS25
|          |             |        |                 |            |            |
| key<157>                          | IOB                    | INPUT        | LVCMOS25
|          |             |        |                 |            |            |
| key<158>                          | IOB                    | INPUT        | LVCMOS25
|          |             |        |                 |            |            |
| key<159>                          | IOB                    | INPUT        | LVCMOS25
|          |             |        |                 |            |            |
| key<160>                          | IOB                    | INPUT        | LVCMOS25
|          |             |        |                 |            |            |
| key<161>                          | IOB                    | INPUT        | LVCMOS25
|          |             |        |                 |            |            |
| key<162>                          | IOB                    | INPUT        | LVCMOS25
|          |             |        |                 |            |            |
```

```
| reset                                | IOB                | INPUT      | LVCMOS25
|       |          |      |              |          |          |
+-----------------------------------------------------------------------------------------------
-------------------------------------------------------------+

Section 7 - RPMs
----------------

Section 8 - Guide Report
------------------------
Guide not run on this design.

Section 9 - Area Group and Partition Summary
--------------------------------------------

Partition Implementation Status
-------------------------------

  No Partitions were found in this design.

-------------------------------

Area Group Information
----------------------

  No area groups were found in this design.

----------------------

Section 10 - Timing Report
--------------------------
A logic-level (pre-route) timing report can be generated by using Xilinx static
timing analysis tools, Timing Analyzer (GUI) or TRCE (command line), with the
mapped NCD and PCF files. Please note that this timing report will be generated
using estimated delay information. For accurate numbers, please generate a
timing report with the post Place and Route NCD file.

For more information about the Timing Analyzer, consult the Xilinx Timing
Analyzer Reference Manual; for more information about TRCE, consult the Xilinx
Command Line Tools User Guide "TRACE" chapter.

Section 11 - Configuration String Details
-----------------------------------------
Use the "-detail" map option to print out Configuration Strings

Section 12 - Control Set Information
------------------------------------
Use the "-detail" map option to print out Control Set Information.

Section 13 - Utilization by Hierarchy
-------------------------------------
Use the "-detail" map option to print out the Utilization by Hierarchy section.
```
