# Supplementary material for: Parallel point-multiplication architecture using combined group operations for high-speed cryptographic applications
Source: PLoS One. 2017 May 1;12(5):e0176214. doi: 10.1371/journal.pone.0176214 (PMC5411040; doi:10.1371/journal.pone.0176214)
Supplement: S1 Supporting Information — (ZIP) [file pone.0176214.s001.zip › S1 Supporting Information/S1 File15 Table3_[g].pdf]

```
****************************************
Report : area
Design : ECC_TOP_B_233
Version: F-2011.09-SP3
Date   : Wed Oct 12 07:51:50 2016
****************************************

Library(s) Used:

    CORE65LPLVT (File: /usr/local-
eit/cad2/cmpstm/stm065v536/CORE65LPLVT_
5.1/libs/CORE65LPLVT_nom_1.20V_25C.db)

Number of ports:                          936
Number of nets:                          7179
Number of cells:                         3452
Number of combinational cells:           2739
Number of sequential cells:               708
Number of macros:                           0
Number of buf/inv:                       1887
Number of references:                      73

Combinational area:       7578903.554620
Noncombinational area:    20081.359813
Net Interconnect area:      undefined  (Wire load has zero net
area)

Total cell area:          7598984.914433
Total area:                 undefined

Hierarchical area distribution
------------------------------

                                  Global cell area
Local cell area
                                  --------------------
--------------------------------
Hierarchical cell                 Absolute      Percent
Combi-        Noncombi-   Black
                                  Total         Total
national      national    boxes   Design
--------------------------------  ------------  -------
------------  ----------  ------  ------------
ECC_TOP_B_233                     7598984.9144    100.0
11947.5197   8050.6399  0.0000  ECC_TOP_B_233
uut_MUX1_new                         7229.0402      0.1
2503.8000   4725.2402  0.0000  MUX_1_new
uut_MUX2_new                         5938.3998      0.1
5938.3998      0.0000  0.0000  MUX_2_new
uut_MUX3                             7461.9997      0.1
156.5200   7305.4797  0.0000  Reg_MUX_3
uut_PD_PA_Jac_233                 7556456.7151     99.4
3339.9599      0.0000  0.0000  PD_PA_BF
uut_PD_PA_Jac_233/Add_A1_PA          1038.9600      0.0
1038.9600      0.0000  0.0000  pol_add_8
uut_PD_PA_Jac_233/Add_A1_PD          1217.8400      0.0
```

```
1217.8400      0.0000  0.0000  pol_add_0
uut_PD_PA_Jac_233/Add_A2_PA          1021.8000      0.0
1021.8000      0.0000  0.0000  pol_add_6
uut_PD_PA_Jac_233/Add_A2_PD          1191.8400      0.0
1191.8400      0.0000  0.0000  pol_add_10
uut_PD_PA_Jac_233/Add_A3_PA           970.3200      0.0
970.3200      0.0000  0.0000  pol_add_5
uut_PD_PA_Jac_233/Add_A3_PD          2610.9199      0.0
2610.9199      0.0000  0.0000  pol_add_9
uut_PD_PA_Jac_233/Add_A4_PA           969.8000      0.0
969.8000      0.0000  0.0000  pol_add_4
uut_PD_PA_Jac_233/Add_A4_PD          2281.2400      0.0
2281.2400      0.0000  0.0000  pol_add_7
uut_PD_PA_Jac_233/Add_A5_PA           969.2800      0.0
969.2800      0.0000  0.0000  pol_add_3
uut_PD_PA_Jac_233/Add_A6_PA           969.8000      0.0
969.8000      0.0000  0.0000  pol_add_2
uut_PD_PA_Jac_233/Add_A7_PA           969.2800      0.0
969.2800      0.0000  0.0000  pol_add_1
uut_PD_PA_Jac_233/SQ_SQ1_PA        380953.0261      5.0
380953.0261      0.0000  0.0000  pol_SQ_3
uut_PD_PA_Jac_233/SQ_SQ1_PD         13283.3997      0.2
13283.3997      0.0000  0.0000  pol_SQ_0
uut_PD_PA_Jac_233/SQ_SQ2_PA        380404.9461      5.0
380404.9461      0.0000  0.0000  pol_SQ_2
uut_PD_PA_Jac_233/SQ_SQ2_PD          5723.1198      0.1
5723.1198      0.0000  0.0000  pol_SQ_7
uut_PD_PA_Jac_233/SQ_SQ3_PA        384407.3860      5.1
384407.3860      0.0000  0.0000  pol_SQ_1
uut_PD_PA_Jac_233/SQ_SQ3_PD         62083.8384      0.8
62083.8384      0.0000  0.0000  pol_SQ_6
uut_PD_PA_Jac_233/SQ_SQ4_PD          5698.1598      0.1
5698.1598      0.0000  0.0000  pol_SQ_5
uut_PD_PA_Jac_233/SQ_SQ5_PD        123419.9168      1.6
123419.9168      0.0000  0.0000  pol_SQ_4
uut_PD_PA_Jac_233/mult_M10_PA      374691.1863      4.9
374691.1863      0.0000  0.0000  pol_mult_2
uut_PD_PA_Jac_233/mult_M11_PA      374642.8263      4.9
374642.8263      0.0000  0.0000  pol_mult_1
uut_PD_PA_Jac_233/mult_M1_PA       375476.3863      4.9
375476.3863      0.0000  0.0000  pol_mult_12
uut_PD_PA_Jac_233/mult_M1_PD       393501.6709      5.2
393501.6709      0.0000  0.0000  pol_mult_0
uut_PD_PA_Jac_233/mult_M2_PA       371846.2664      4.9
371846.2664      0.0000  0.0000  pol_mult_11
uut_PD_PA_Jac_233/mult_M2_PD       400869.0322      5.3
400869.0322      0.0000  0.0000  pol_mult_15
uut_PD_PA_Jac_233/mult_M3_PA       371583.6664      4.9
371583.6664      0.0000  0.0000  pol_mult_10
uut_PD_PA_Jac_233/mult_M3_PD       395516.1522      5.2
395516.1522      0.0000  0.0000  pol_mult_14
uut_PD_PA_Jac_233/mult_M4_PA       375176.8663      4.9
375176.8663      0.0000  0.0000  pol_mult_8
uut_PD_PA_Jac_233/mult_M4_PD       429571.9873      5.7
429571.9873      0.0000  0.0000  pol_mult_13
uut_PD_PA_Jac_233/mult_M5_PA       375044.2663      4.9
375044.2663      0.0000  0.0000  pol_mult_7
```

```
uut_PD_PA_Jac_233/mult_M5_PD        446632.1506       5.9
446632.1506      0.0000  0.0000  pol_mult_9
uut_PD_PA_Jac_233/mult_M6_PA        376323.9863       5.0
376323.9863      0.0000  0.0000  pol_mult_5
uut_PD_PA_Jac_233/mult_M7_PA        374968.3463       4.9
374968.3463      0.0000  0.0000  pol_mult_6
uut_PD_PA_Jac_233/mult_M8_PA        374687.5463       4.9
374687.5463      0.0000  0.0000  pol_mult_3
uut_PD_PA_Jac_233/mult_M9_PA        372399.5464       4.9
372399.5464      0.0000  0.0000  pol_mult_4
uut_select_signal                      1900.6000       0.0
1900.6000      0.0000  0.0000  select_logic
--------------------------------  ------------  -------
------------  ----------  ------  ------------
Total
7578903.5546  20081.3598  0.0000

1
```
