# Supplementary material for: Parallel point-multiplication architecture using combined group operations for high-speed cryptographic applications
Source: PLoS One. 2017 May 1;12(5):e0176214. doi: 10.1371/journal.pone.0176214 (PMC5411040; doi:10.1371/journal.pone.0176214)
Supplement: S1 Supporting Information — (ZIP) [file pone.0176214.s001.zip › S1 Supporting Information/S1 File16 Table3_[g].pdf]

```
Information: Updating design information... (UID-85)
Warning: Design 'ECC_TOP_B_233' contains 3 high-fanout nets. A
fanout number of 1000 will be used for delay calculations
involving these nets. (TIM-134)

****************************************
Report : timing
        -path full
        -delay max
        -max_paths 1
Design : ECC_TOP_B_233
Version: F-2011.09-SP3
Date   : Wed Oct 12 07:51:44 2016
****************************************

 # A fanout number of 1000 was used for high fanout net
computations.

Operating Conditions: nom_1.20V_25C   Library: CORE65LPLVT
Wire Load Model Mode: enclosed

  Startpoint: uut_MUX3/QZout_reg[231]
              (rising edge-triggered flip-flop clocked by clk)
  Endpoint: QY_reg[126]
            (rising edge-triggered flip-flop clocked by clk)
  Path Group: clk
  Path Type: max

  Des/Clust/Port     Wire Load Model       Library
  ------------------------------------------------
  ECC_TOP_B_233      area_780Kto1170K      CORE65LPLVT
  pol_SQ_0           area_12Kto18K         CORE65LPLVT
  PD_PA_BF           area_780Kto1170K      CORE65LPLVT
  pol_SQ_6           area_60Kto66K         CORE65LPLVT
  pol_SQ_4           area_78Kto156K        CORE65LPLVT
  pol_mult_13        area_390Kto780K       CORE65LPLVT
  pol_mult_9         area_390Kto780K       CORE65LPLVT
  MUX_2_new          area_5Kto6K           CORE65LPLVT

  Point                                                   Incr
Path
  ---------------------------------------------------------------
--------------
  clock clk (rise edge)                                   0.00
0.00
  clock network delay (ideal)                             0.00
0.00
  uut_MUX3/QZout_reg[231]/CP (HS65_LL_DFPRQX9)            0.00
#    0.00 r
  uut_MUX3/QZout_reg[231]/Q (HS65_LL_DFPRQX9)             0.10
0.10 f
  uut_MUX3/QZout[231] (Reg_MUX_3)                         0.00
0.10 f
  uut_PD_PA_Jac_233/Z1[231] (PD_PA_BF)                    0.00
0.10 f
  uut_PD_PA_Jac_233/SQ_SQ1_PD/A[231] (pol_SQ_0)           0.00
0.10 f
```

uut_PD_PA_Jac_233/SQ_SQ1_PD/U508/Z (HS65_LL_IVX18) 0.03
0.12 r
uut_PD_PA_Jac_233/SQ_SQ1_PD/U393/Z (HS65_LL_BFX53) 0.03
0.15 r
uut_PD_PA_Jac_233/SQ_SQ1_PD/U392/Z (HS65_LL_IVX27) 0.01
0.17 f
uut_PD_PA_Jac_233/SQ_SQ1_PD/U1443/Z (HS65_LL_AND2X18)
0.04
0.21 f
uut_PD_PA_Jac_233/SQ_SQ1_PD/U69/Z (HS65_LL_IVX44) 0.03
0.23 r
uut_PD_PA_Jac_233/SQ_SQ1_PD/U1127/Z (HS65_LL_MUX21I1X12)
0.04
0.27 r
uut_PD_PA_Jac_233/SQ_SQ1_PD/U1667/Z (HS65_LL_NAND2X5)
0.02
0.29 f
uut_PD_PA_Jac_233/SQ_SQ1_PD/U2109/Z (HS65_LL_XOR2X18)
0.06
0.35 f
uut_PD_PA_Jac_233/SQ_SQ1_PD/U345/Z (HS65_LL_IVX27) 0.02
0.37 r
uut_PD_PA_Jac_233/SQ_SQ1_PD/U976/Z (HS65_LL_XOR2X35)
0.06
0.43 r
uut_PD_PA_Jac_233/SQ_SQ1_PD/C[191] (pol_SQ_0) 0.00
0.43 r
uut_PD_PA_Jac_233/SQ_SQ3_PD/A[191] (pol_SQ_6) 0.00
0.43 r
uut_PD_PA_Jac_233/SQ_SQ3_PD/U1637/Z (HS65_LL_IVX53) 0.02
0.45 f
uut_PD_PA_Jac_233/SQ_SQ3_PD/U1636/Z (HS65_LL_IVX71) 0.02
0.47 r
uut_PD_PA_Jac_233/SQ_SQ3_PD/U8284/Z (HS65_LL_AND2X35)
0.04
0.51 r
uut_PD_PA_Jac_233/SQ_SQ3_PD/U6531/Z (HS65_LL_XOR3X18)
0.09
0.59 r
uut_PD_PA_Jac_233/SQ_SQ3_PD/U9157/Z (HS65_LL_XOR2X18)
0.04
0.63 f
uut_PD_PA_Jac_233/SQ_SQ3_PD/U9156/Z (HS65_LL_XOR3X18)
0.09
0.72 r
uut_PD_PA_Jac_233/SQ_SQ3_PD/U9890/Z (HS65_LL_XOR3X18)
0.08
0.80 r
uut_PD_PA_Jac_233/SQ_SQ3_PD/U9876/Z (HS65_LL_XOR3X18)
0.08
0.88 r
uut_PD_PA_Jac_233/SQ_SQ3_PD/U658/Z (HS65_LL_XOR3X18)
0.09
0.97 r
uut_PD_PA_Jac_233/SQ_SQ3_PD/U2235/Z (HS65_LL_XOR2X18)
0.06
1.03 r

```
  uut_PD_PA_Jac_233/SQ_SQ3_PD/C[94] (pol_SQ_6)             0.00
1.03 r
  uut_PD_PA_Jac_233/SQ_SQ5_PD/A[94] (pol_SQ_4)             0.00
1.03 r
  uut_PD_PA_Jac_233/SQ_SQ5_PD/U10863/Z (HS65_LL_IVX27)
                                                           0.01
1.04 f
  uut_PD_PA_Jac_233/SQ_SQ5_PD/U6058/Z (HS65_LL_BFX106)
                                                           0.04
1.08 f
  uut_PD_PA_Jac_233/SQ_SQ5_PD/U4127/Z (HS65_LL_IVX106)
                                                           0.02
1.10 r
  uut_PD_PA_Jac_233/SQ_SQ5_PD/U13845/Z (HS65_LL_AND2X4)
                                                           0.05
1.15 r
  uut_PD_PA_Jac_233/SQ_SQ5_PD/U19347/Z (HS65_LL_XOR3X9)
                                                           0.10
1.25 r
  uut_PD_PA_Jac_233/SQ_SQ5_PD/U7877/Z (HS65_LL_XOR2X18)
                                                           0.04
1.29 f
  uut_PD_PA_Jac_233/SQ_SQ5_PD/U19348/Z (HS65_LL_XOR3X9)
                                                           0.09
1.38 r
  uut_PD_PA_Jac_233/SQ_SQ5_PD/U19349/Z (HS65_LL_XOR2X9)
                                                           0.05
1.43 r
  uut_PD_PA_Jac_233/SQ_SQ5_PD/U4309/Z (HS65_LL_XOR3X4)
                                                           0.09
1.52 r
  uut_PD_PA_Jac_233/SQ_SQ5_PD/U18822/Z (HS65_LL_XOR2X18)
                                                           0.06
1.59 f
  uut_PD_PA_Jac_233/SQ_SQ5_PD/U21233/Z (HS65_LL_IVX18)
                                                           0.01
1.60 r
  uut_PD_PA_Jac_233/SQ_SQ5_PD/U15246/Z (HS65_LL_AO22X9)
                                                           0.04
1.64 r
  uut_PD_PA_Jac_233/SQ_SQ5_PD/U15245/Z (HS65_LL_AO22X27)
                                                           0.05
1.69 r
  uut_PD_PA_Jac_233/SQ_SQ5_PD/U5882/Z (HS65_LL_IVX9)       0.01
1.70 f
  uut_PD_PA_Jac_233/SQ_SQ5_PD/U9334/Z (HS65_LL_OAI22X6)
                                                           0.04
1.74 r
  uut_PD_PA_Jac_233/SQ_SQ5_PD/C[77] (pol_SQ_4)             0.00
1.74 r
  uut_PD_PA_Jac_233/U157/Z (HS65_LL_BFX31)                 0.04
1.78 r
  uut_PD_PA_Jac_233/mult_M4_PD/B[77] (pol_mult_13)         0.00
1.78 r
  uut_PD_PA_Jac_233/mult_M4_PD/U1343/Z (HS65_LL_IVX44)
                                                           0.02
1.80 f
```

```
  uut_PD_PA_Jac_233/mult_M4_PD/U13286/Z (HS65_LL_BFX106)
                                                              0.03
1.83 f
  uut_PD_PA_Jac_233/mult_M4_PD/U18098/Z (HS65_LL_IVX53)
                                                              0.02
1.85 r
  uut_PD_PA_Jac_233/mult_M4_PD/U10092/Z (HS65_LL_NAND2X4)
                                                              0.02
1.87 f
  uut_PD_PA_Jac_233/mult_M4_PD/U18096/Z (HS65_LLS_XOR3X4)
                                                              0.10
1.97 r
  uut_PD_PA_Jac_233/mult_M4_PD/U17397/Z (HS65_LL_XOR3X18)
                                                              0.11
2.08 r
  uut_PD_PA_Jac_233/mult_M4_PD/U17876/Z (HS65_LL_XOR3X18)
                                                              0.09
2.17 r
  uut_PD_PA_Jac_233/mult_M4_PD/U11462/Z (HS65_LL_XOR3X18)
                                                              0.08
2.25 r
  uut_PD_PA_Jac_233/mult_M4_PD/U72063/Z (HS65_LL_XOR3X18)
                                                              0.08
2.34 r
  uut_PD_PA_Jac_233/mult_M4_PD/U998/Z (HS65_LL_XOR3X18)
                                                              0.09
2.43 r
  uut_PD_PA_Jac_233/mult_M4_PD/U1/Z (HS65_LLS_XNOR2X18)
                                                              0.04
2.46 r
  uut_PD_PA_Jac_233/mult_M4_PD/C[152] (pol_mult_13)           0.00
2.46 r
  uut_PD_PA_Jac_233/Add_A3_PD/A[152] (pol_add_9)              0.00
2.46 r
  uut_PD_PA_Jac_233/Add_A3_PD/U79/Z (HS65_LL_XOR2X35)         0.05
2.51 f
  uut_PD_PA_Jac_233/Add_A3_PD/C[152] (pol_add_9)              0.00
2.51 f
  uut_PD_PA_Jac_233/mult_M5_PD/A[152] (pol_mult_9)            0.00
2.51 f
  uut_PD_PA_Jac_233/mult_M5_PD/U20025/Z (HS65_LL_BFX106)
                                                              0.04
2.55 f
  uut_PD_PA_Jac_233/mult_M5_PD/U2103/Z (HS65_LL_IVX31)
                                                              0.04
2.59 r
  uut_PD_PA_Jac_233/mult_M5_PD/U4116/Z (HS65_LL_NAND2X4)
                                                              0.04
2.63 f
  uut_PD_PA_Jac_233/mult_M5_PD/U44742/Z (HS65_LL_IVX18)
                                                              0.05
2.68 r
  uut_PD_PA_Jac_233/mult_M5_PD/U9550/Z (HS65_LL_MX41X4)
                                                              0.07
2.75 r
  uut_PD_PA_Jac_233/mult_M5_PD/U10968/Z (HS65_LL_IVX9)
                                                              0.02
```

```
2.77 f
  uut_PD_PA_Jac_233/mult_M5_PD/U10124/Z (HS65_LL_XOR3X18)
                                                          0.09
2.86 r
  uut_PD_PA_Jac_233/mult_M5_PD/U10123/Z (HS65_LLS_XOR3X4)
                                                          0.07
2.93 r
  uut_PD_PA_Jac_233/mult_M5_PD/U75586/Z (HS65_LL_XOR3X18)
                                                          0.10
3.04 r
  uut_PD_PA_Jac_233/mult_M5_PD/U29772/Z (HS65_LL_XOR2X35)
                                                          0.06
3.09 f
  uut_PD_PA_Jac_233/mult_M5_PD/U8703/Z (HS65_LL_XOR3X18)
                                                          0.09
3.18 r
  uut_PD_PA_Jac_233/mult_M5_PD/U31270/Z (HS65_LL_XOR2X18)
                                                          0.04
3.22 f
  uut_PD_PA_Jac_233/mult_M5_PD/U16496/Z (HS65_LL_XNOR2X18)
                                                          0.04
3.26 r
  uut_PD_PA_Jac_233/mult_M5_PD/U12881/Z (HS65_LL_XOR3X18)
                                                          0.08
3.34 r
  uut_PD_PA_Jac_233/mult_M5_PD/C[126] (pol_mult_9)        0.00
3.34 r
  uut_PD_PA_Jac_233/Add_A4_PD/A[126] (pol_add_7)          0.00
3.34 r
  uut_PD_PA_Jac_233/Add_A4_PD/U8/Z (HS65_LL_XOR2X35)      0.04
3.39 f
  uut_PD_PA_Jac_233/Add_A4_PD/C[126] (pol_add_7)          0.00
3.39 f
  uut_PD_PA_Jac_233/Y3_PD[126] (PD_PA_BF)                 0.00
3.39 f
  uut_MUX2_new/Y3_PD[126] (MUX_2_new)                     0.00
3.39 f
  uut_MUX2_new/U29/Z (HS65_LL_IVX27)                      0.01
3.40 r
  uut_MUX2_new/U468/Z (HS65_LL_MUX21I1X18)                0.01
3.42 f
  uut_MUX2_new/sQY[126] (MUX_2_new)                       0.00
3.42 f
  U878/Z (HS65_LL_MUX21X18)                               0.05
3.46 f
  QY_reg[126]/D (HS65_LLS_DFPRQX35)                       0.00
3.46 f
  data arrival time
3.46

  clock clk (rise edge)                                   3.00
3.00
  clock network delay (ideal)                             0.00
3.00
  clock uncertainty                                      -0.10
2.90
  QY_reg[126]/CP (HS65_LLS_DFPRQX35)                      0.00
```

```
2.90 r
  library setup time                                    -0.07
2.83
  data required time
2.83
  -------------------------------------------------------------
--------------
  data required time
2.83
  data arrival time
-3.46
  -------------------------------------------------------------
--------------
  slack (VIOLATED)
-0.63


1
```
