# Supplementary material for: Parallel point-multiplication architecture using combined group operations for high-speed cryptographic applications
Source: PLoS One. 2017 May 1;12(5):e0176214. doi: 10.1371/journal.pone.0176214 (PMC5411040; doi:10.1371/journal.pone.0176214)
Supplement: S1 Supporting Information — (ZIP) [file pone.0176214.s001.zip › S1 Supporting Information/S1 File17 Table3_[g].pdf]

```
Information: Propagating switching activity (low effort zero
delay simulation). (PWR-6)
Warning: Design has unannotated primary inputs. (PWR-414)
Warning: Design has unannotated sequential cell outputs. (PWR-
415)

****************************************
Report : power
        -analysis_effort low
Design : ECC_TOP_B_233
Version: F-2011.09-SP3
Date   : Wed Oct 12 07:53:55 2016
****************************************


Library(s) Used:

    CORE65LPLVT (File: /usr/local-
eit/cad2/cmpstm/stm065v536/CORE65LPLVT_
5.1/libs/CORE65LPLVT_nom_1.20V_25C.db)


Operating Conditions: nom_1.20V_25C   Library: CORE65LPLVT
Wire Load Model Mode: enclosed

Design        Wire Load Model            Library
------------------------------------------------
ECC_TOP_B_233          area_780Kto1170K  CORE65LPLVT
PD_PA_BF               area_780Kto1170K  CORE65LPLVT
select_logic           area_1Kto2K       CORE65LPLVT
MUX_1_new              area_7Kto8K       CORE65LPLVT
MUX_2_new              area_5Kto6K       CORE65LPLVT
Reg_MUX_3              area_7Kto8K       CORE65LPLVT
pol_SQ_0               area_12Kto18K     CORE65LPLVT
pol_mult_0             area_390Kto780K   CORE65LPLVT
pol_SQ_7               area_5Kto6K       CORE65LPLVT
pol_SQ_6               area_60Kto66K     CORE65LPLVT
pol_add_0              area_1Kto2K       CORE65LPLVT
pol_mult_15            area_390Kto780K   CORE65LPLVT
pol_SQ_5               area_5Kto6K       CORE65LPLVT
pol_SQ_4               area_78Kto156K    CORE65LPLVT
pol_add_10             area_1Kto2K       CORE65LPLVT
pol_mult_14            area_390Kto780K   CORE65LPLVT
pol_SQ_3               area_312Kto390K   CORE65LPLVT
pol_mult_13            area_390Kto780K   CORE65LPLVT
pol_mult_12            area_312Kto390K   CORE65LPLVT
pol_mult_11            area_312Kto390K   CORE65LPLVT
pol_add_9              area_2Kto3K       CORE65LPLVT
pol_mult_10            area_312Kto390K   CORE65LPLVT
pol_mult_9             area_390Kto780K   CORE65LPLVT
pol_add_8              area_1Kto2K       CORE65LPLVT
pol_add_7              area_2Kto3K       CORE65LPLVT
pol_mult_8             area_312Kto390K   CORE65LPLVT
pol_SQ_2               area_312Kto390K   CORE65LPLVT
pol_add_6              area_1Kto2K       CORE65LPLVT
pol_SQ_1               area_312Kto390K   CORE65LPLVT
pol_mult_7             area_312Kto390K   CORE65LPLVT
```

pol_mult_6	area_312Kto390K	CORE65LPLVT
pol_add_5	area_0Kto1K	CORE65LPLVT
pol_mult_5	area_312Kto390K	CORE65LPLVT
pol_mult_4	area_312Kto390K	CORE65LPLVT
pol_mult_3	area_312Kto390K	CORE65LPLVT
pol_add_4	area_0Kto1K	CORE65LPLVT
pol_mult_2	area_312Kto390K	CORE65LPLVT
pol_add_3	area_0Kto1K	CORE65LPLVT
pol_mult_1	area_312Kto390K	CORE65LPLVT
pol_add_1	area_0Kto1K	CORE65LPLVT
pol_add_2	area_0Kto1K	CORE65LPLVT

```
Global Operating Voltage = 1.2
Power-specific unit information :
    Voltage Units = 1V
    Capacitance Units = 1.000000pf
    Time Units = 1ns
    Dynamic Power Units = 1mW    (derived from V,C,T units)
    Leakage Power Units = 1mW


  Cell Internal Power  = 360.7072 mW   (33%)
  Net Switching Power  = 721.1508 mW   (67%)
                         ---------
Total Dynamic Power    =   1.0819  W  (100%)

Cell Leakage Power     =   3.1190 mW
```

Power Group	Internal Power	Switching Power	Leakage Power	Total Power (%)	Attrs
io_pad	0.0000	0.0000	0.0000	0.0000 (0.00%)	
memory	0.0000	0.0000	0.0000	0.0000 (0.00%)	
black_box	0.0000	0.0000	0.0000	0.0000 (0.00%)	
clock_network	0.0000	0.0000	0.0000	0.0000 (0.00%)	
register	4.9974	0.4726	1.0707e-02	5.4807 (0.51%)	
sequential	0.2851	0.1523	1.9226e-03	0.4393 (0.04%)	
combinational	355.0090	720.2741	3.1149	1.0784e+03 (99.45%)	
Total	360.2916 mW	720.8990 mW	3.1276 mW	1.0843e+03 mW	

1
