# Supplementary material for: Parallel point-multiplication architecture using combined group operations for high-speed cryptographic applications
Source: PLoS One. 2017 May 1;12(5):e0176214. doi: 10.1371/journal.pone.0176214 (PMC5411040; doi:10.1371/journal.pone.0176214)
Supplement: S1 Supporting Information — (ZIP) [file pone.0176214.s001.zip › S1 Supporting Information/S1 File18 Table3_[h].pdf]

```
****************************************
Report : area
Design : ECC_TOP_K_233
Version: F-2011.09-SP3
Date   : Wed Oct 12 21:58:42 2016
****************************************

Library(s) Used:

    CORE65LPLVT (File: /usr/local-
eit/cad2/cmpstm/stm065v536/CORE65LPLVT_
5.1/libs/CORE65LPLVT_nom_1.20V_25C.db)

Number of ports:                          936
Number of nets:                          7070
Number of cells:                         3343
Number of combinational cells:           2630
Number of sequential cells:               708
Number of macros:                           0
Number of buf/inv:                       1770
Number of references:                      69

Combinational area:       8184231.901326
Noncombinational area:    20505.679933
Net Interconnect area:      undefined  (Wire load has zero net
area)

Total cell area:          8204737.581259
Total area:                 undefined

Hierarchical area distribution
------------------------------


                                  Global cell area
Local cell area
                                  ---------------------
--------------------------------
Hierarchical cell                 Absolute      Percent
Combi-        Noncombi-   Black
                                  Total         Total
national      national    boxes   Design
--------------------------------  ------------  -------
------------  ----------  ------  ------------
ECC_TOP_K_233                     8204737.5813    100.0
9968.3997   8507.7200  0.0000  ECC_TOP_K_233
uut_MUX1_new                         7224.8802      0.1
2499.6400   4725.2402  0.0000  MUX_1_new
uut_MUX2_new                         4327.4399      0.1
4327.4399      0.0000  0.0000  MUX_2_new
uut_MUX3                             7406.3597      0.1
133.6400   7272.7197  0.0000  Reg_MUX_3
uut_PD_PA_Jac_233                 8165483.3017     99.5
2097.6799      0.0000  0.0000  PD_PA_BF
uut_PD_PA_Jac_233/Add_A1_PA          1067.5600      0.0
1067.5600      0.0000  0.0000  pol_add_8
uut_PD_PA_Jac_233/Add_A1_PD           970.3200      0.0
```

```
970.3200      0.0000  0.0000  pol_add_0
uut_PD_PA_Jac_233/Add_A2_PA          1037.9200      0.0
1037.9200      0.0000  0.0000  pol_add_6
uut_PD_PA_Jac_233/Add_A2_PD          1022.8400      0.0
1022.8400      0.0000  0.0000  pol_add_10
uut_PD_PA_Jac_233/Add_A3_PA           969.2800      0.0
969.2800      0.0000  0.0000  pol_add_5
uut_PD_PA_Jac_233/Add_A3_PD          2169.4400      0.0
2169.4400      0.0000  0.0000  pol_add_9
uut_PD_PA_Jac_233/Add_A4_PA           969.2800      0.0
969.2800      0.0000  0.0000  pol_add_4
uut_PD_PA_Jac_233/Add_A4_PD          2211.0400      0.0
2211.0400      0.0000  0.0000  pol_add_7
uut_PD_PA_Jac_233/Add_A5_PA           969.2800      0.0
969.2800      0.0000  0.0000  pol_add_3
uut_PD_PA_Jac_233/Add_A6_PA           969.2800      0.0
969.2800      0.0000  0.0000  pol_add_2
uut_PD_PA_Jac_233/Add_A7_PA           969.2800      0.0
969.2800      0.0000  0.0000  pol_add_1
uut_PD_PA_Jac_233/SQ_SQ1_PA        381413.2261      4.6
381413.2261      0.0000  0.0000  pol_SQ_3
uut_PD_PA_Jac_233/SQ_SQ1_PD        159861.5166      1.9
159861.5166      0.0000  0.0000  pol_SQ_0
uut_PD_PA_Jac_233/SQ_SQ2_PA        380615.5461      4.6
380615.5461      0.0000  0.0000  pol_SQ_2
uut_PD_PA_Jac_233/SQ_SQ2_PD          5396.5598      0.1
5396.5598      0.0000  0.0000  pol_SQ_7
uut_PD_PA_Jac_233/SQ_SQ3_PA        384311.7060      4.7
384311.7060      0.0000  0.0000  pol_SQ_1
uut_PD_PA_Jac_233/SQ_SQ3_PD        411943.4710      5.0
411943.4710      0.0000  0.0000  pol_SQ_6
uut_PD_PA_Jac_233/SQ_SQ4_PD          5277.4798      0.1
5277.4798      0.0000  0.0000  pol_SQ_5
uut_PD_PA_Jac_233/SQ_SQ5_PD        495791.3898      6.0
495791.3898      0.0000  0.0000  pol_SQ_4
uut_PD_PA_Jac_233/mult_M10_PA      373057.8664      4.5
373057.8664      0.0000  0.0000  pol_mult_2
uut_PD_PA_Jac_233/mult_M11_PA      373150.9464      4.5
373150.9464      0.0000  0.0000  pol_mult_1
uut_PD_PA_Jac_233/mult_M1_PA       373468.1464      4.6
373468.1464      0.0000  0.0000  pol_mult_12
uut_PD_PA_Jac_233/mult_M1_PD       355541.1515      4.3
355541.1515      0.0000  0.0000  pol_mult_0
uut_PD_PA_Jac_233/mult_M2_PA       368710.6665      4.5
368710.6665      0.0000  0.0000  pol_mult_11
uut_PD_PA_Jac_233/mult_M2_PD       354353.9908      4.3
354353.9908      0.0000  0.0000  pol_mult_15
uut_PD_PA_Jac_233/mult_M3_PA       368477.7065      4.5
368477.7065      0.0000  0.0000  pol_mult_10
uut_PD_PA_Jac_233/mult_M3_PD       354140.7909      4.3
354140.7909      0.0000  0.0000  pol_mult_14
uut_PD_PA_Jac_233/mult_M4_PA       373609.5864      4.6
373609.5864      0.0000  0.0000  pol_mult_8
uut_PD_PA_Jac_233/mult_M4_PD       359221.7104      4.4
359221.7104      0.0000  0.0000  pol_mult_13
uut_PD_PA_Jac_233/mult_M5_PA       373422.3864      4.6
373422.3864      0.0000  0.0000  pol_mult_7
```

```
uut_PD_PA_Jac_233/mult_M5_PD       407520.3530      5.0
407520.3530      0.0000  0.0000  pol_mult_9
uut_PD_PA_Jac_233/mult_M6_PA       374369.3063      4.6
374369.3063      0.0000  0.0000  pol_mult_5
uut_PD_PA_Jac_233/mult_M7_PA       373615.8264      4.6
373615.8264      0.0000  0.0000  pol_mult_6
uut_PD_PA_Jac_233/mult_M8_PA       373226.8664      4.5
373226.8664      0.0000  0.0000  pol_mult_3
uut_PD_PA_Jac_233/mult_M9_PA       369561.9065      4.5
369561.9065      0.0000  0.0000  pol_mult_4
uut_select_signal                    1819.4800      0.0
1819.4800      0.0000  0.0000  select_logic
--------------------------------  ------------  -------
------------  ----------  ------  ------------
Total
8184231.9013  20505.6799  0.0000

1
```
