# Supplementary material for: Parallel point-multiplication architecture using combined group operations for high-speed cryptographic applications
Source: PLoS One. 2017 May 1;12(5):e0176214. doi: 10.1371/journal.pone.0176214 (PMC5411040; doi:10.1371/journal.pone.0176214)
Supplement: S1 Supporting Information — (ZIP) [file pone.0176214.s001.zip › S1 Supporting Information/S1 File2 Table1.pdf]

```
Release 14.7 Map P.20131013 (nt64)
Xilinx Mapping Report File for Design 'ECC_TOP_K_233'

Design Information
------------------
Command Line   : map -intstyle ise -p xc7vx980t-ffg1930-2 -w -logic_opt off -ol
high -t 1 -xt 0 -register_duplication off -r 4 -mt off -ir off -pr off -lc off
-power off -o ECC_TOP_K_233_map.ncd ECC_TOP_K_233.ngd ECC_TOP_K_233.pcf
Target Device  : xc7vx980t
Target Package : ffg1930
Target Speed   : -2
Mapper Version : virtex7 -- $Revision: 1.55 $
Mapped Date    : Tue Sep 27 23:31:25 2016

Design Summary
--------------
Number of errors:      0
Number of warnings:  704
Slice Logic Utilization:
  Number of Slice Registers:                 2,106 out of 1,224,000    1%
    Number used as Flip Flops:               1,407
    Number used as Latches:                    699
    Number used as Latch-thrus:                  0
    Number used as AND/OR logics:                0
  Number of Slice LUTs:                    473,112 out of 612,000   77%
    Number used as logic:                  473,112 out of 612,000   77%
      Number using O6 output only:         430,344
      Number using O5 output only:               0
      Number using O5 and O6:               42,768
      Number used as ROM:                        0
    Number used as Memory:                       0 out of 221,400    0%
    Number used exclusively as route-thrus:      0

Slice Logic Distribution:
  Number of occupied Slices:               134,684 out of 153,000   88%
  Number of LUT Flip Flop pairs used:      473,323
    Number with an unused Flip Flop:       471,217 out of 473,323   99%
    Number with an unused LUT:                 211 out of 473,323    1%
    Number of fully used LUT-FF pairs:       1,895 out of 473,323    1%
    Number of unique control sets:               3
    Number of slice register sites lost
      to control set restrictions:              14 out of 1,224,000    1%

  A LUT Flip Flop pair for this architecture represents one LUT paired with
  one Flip Flop within a slice.  A control set is a unique combination of
  clock, reset, set, and enable signals for a registered element.
  The Slice Logic Distribution report is not meaningful if the design is
  over-mapped for a non-slice resource or if Placement fails.
  OVERMAPPING of BRAM resources should be ignored if the design is
  over-mapped for a non-BRAM resource or if placement fails.

IO Utilization:
  Number of bonded IOBs:                       702 out of     900   78%

Specific Feature Utilization:
  Number of RAMB36E1/FIFO36E1s:                  0 out of   1,500    0%
  Number of RAMB18E1/FIFO18E1s:                  0 out of   3,000    0%
  Number of BUFG/BUFGCTRLs:                      2 out of      32    6%
    Number used as BUFGs:                        2
    Number used as BUFGCTRLs:                    0
  Number of IDELAYE2/IDELAYE2_FINEDELAYs:        0 out of     900    0%
  Number of ILOGICE2/ILOGICE3/ISERDESE2s:        0 out of     900    0%
  Number of ODELAYE2/ODELAYE2_FINEDELAYs:        0 out of     900    0%
  Number of OLOGICE2/OLOGICE3/OSERDESE2s:        0 out of     900    0%
  Number of PHASER_IN/PHASER_IN_PHYs:            0 out of      72    0%
  Number of PHASER_OUT/PHASER_OUT_PHYs:          0 out of      72    0%
  Number of BSCANs:                              0 out of       4    0%
  Number of BUFHCEs:                             0 out of     216    0%
  Number of BUFRs:                               0 out of      72    0%
  Number of CAPTUREs:                            0 out of       1    0%
  Number of DNA_PORTs:                           0 out of       1    0%
```

```
  Number of DSP48E1s:                          0 out of   3,600    0%
  Number of EFUSE_USRs:                        0 out of       1    0%
  Number of FRAME_ECCs:                        0 out of       1    0%
  Number of GTHE2_CHANNELs:                    0 out of      72    0%
  Number of GTHE2_COMMONs:                     0 out of      18    0%
  Number of IBUFDS_GTE2s:                      0 out of      36    0%
  Number of ICAPs:                             0 out of       2    0%
  Number of IDELAYCTRLs:                       0 out of      18    0%
  Number of IN_FIFOs:                          0 out of      72    0%
  Number of MMCME2_ADVs:                       0 out of      18    0%
  Number of OUT_FIFOs:                         0 out of      72    0%
  Number of PCIE_3_0s:                         0 out of       3    0%
  Number of PHASER_REFs:                       0 out of      18    0%
  Number of PHY_CONTROLs:                      0 out of      18    0%
  Number of PLLE2_ADVs:                        0 out of      18    0%
  Number of STARTUPs:                          0 out of       1    0%
  Number of XADCs:                             0 out of       1    0%

Average Fanout of Non-Clock Nets:              4.50

Peak Memory Usage:  7680 MB
Total REAL time to MAP completion:  4 hrs 14 mins 52 secs
Total CPU time to MAP completion:   4 hrs 14 mins 16 secs

Table of Contents
-----------------
Section 1 - Errors
Section 2 - Warnings
Section 3 - Informational
Section 4 - Removed Logic Summary
Section 5 - Removed Logic
Section 6 - IOB Properties
Section 7 - RPMs
Section 8 - Guide Report
Section 9 - Area Group and Partition Summary
Section 10 - Timing Report
Section 11 - Configuration String Information
Section 12 - Control Set Information
Section 13 - Utilization by Hierarchy

Section 1 - Errors
------------------

Section 2 - Warnings
--------------------
WARNING:LIT:701 - PAD symbol "clk" has an undefined IOSTANDARD.
WARNING:LIT:702 - PAD symbol "clk" is not constrained (LOC) to a specific
   location.
WARNING:PhysDesignRules:2452 - The IOB reset is either not constrained (LOC) to
   a specific location and/or has an undefined I/O Standard (IOSTANDARD). This
   condition may seriously affect the device and will be an error in bitstream
   creation. It should be corrected by properly specifying the pin location and
   I/O Standard.
WARNING:PhysDesignRules:2452 - The IOB QY<10> is either not constrained (LOC) to
   a specific location and/or has an undefined I/O Standard (IOSTANDARD). This
   condition may seriously affect the device and will be an error in bitstream
   creation. It should be corrected by properly specifying the pin location and
   I/O Standard.
WARNING:PhysDesignRules:2452 - The IOB QY<11> is either not constrained (LOC) to
   a specific location and/or has an undefined I/O Standard (IOSTANDARD). This
   condition may seriously affect the device and will be an error in bitstream
   creation. It should be corrected by properly specifying the pin location and
   I/O Standard.
WARNING:PhysDesignRules:2452 - The IOB QY<12> is either not constrained (LOC) to
   a specific location and/or has an undefined I/O Standard (IOSTANDARD). This
   condition may seriously affect the device and will be an error in bitstream
   creation. It should be corrected by properly specifying the pin location and
   I/O Standard.
WARNING:PhysDesignRules:2452 - The IOB QY<13> is either not constrained (LOC) to
   a specific location and/or has an undefined I/O Standard (IOSTANDARD). This
   condition may seriously affect the device and will be an error in bitstream
```

```
   creation. It should be corrected by properly specifying the pin location and
   I/O Standard.
WARNING:PhysDesignRules:2452 - The IOB QY<14> is either not constrained (LOC) to
   a specific location and/or has an undefined I/O Standard (IOSTANDARD). This
   condition may seriously affect the device and will be an error in bitstream
   creation. It should be corrected by properly specifying the pin location and
   I/O Standard.
WARNING:PhysDesignRules:2452 - The IOB QY<15> is either not constrained (LOC) to
   a specific location and/or has an undefined I/O Standard (IOSTANDARD). This
   condition may seriously affect the device and will be an error in bitstream
   creation. It should be corrected by properly specifying the pin location and
   I/O Standard.
WARNING:PhysDesignRules:2452 - The IOB QY<16> is either not constrained (LOC) to
   a specific location and/or has an undefined I/O Standard (IOSTANDARD). This
   condition may seriously affect the device and will be an error in bitstream
   creation. It should be corrected by properly specifying the pin location and
   I/O Standard.
WARNING:PhysDesignRules:2452 - The IOB QY<17> is either not constrained (LOC) to
   a specific location and/or has an undefined I/O Standard (IOSTANDARD). This
   condition may seriously affect the device and will be an error in bitstream
   creation. It should be corrected by properly specifying the pin location and
   I/O Standard.
WARNING:PhysDesignRules:2452 - The IOB QY<18> is either not constrained (LOC) to
   a specific location and/or has an undefined I/O Standard (IOSTANDARD). This
   condition may seriously affect the device and will be an error in bitstream
   creation. It should be corrected by properly specifying the pin location and
   I/O Standard.
WARNING:PhysDesignRules:2452 - The IOB QY<19> is either not constrained (LOC) to
   a specific location and/or has an undefined I/O Standard (IOSTANDARD). This
   condition may seriously affect the device and will be an error in bitstream
   creation. It should be corrected by properly specifying the pin location and
   I/O Standard.
WARNING:PhysDesignRules:2452 - The IOB QY<20> is either not constrained (LOC) to
   a specific location and/or has an undefined I/O Standard (IOSTANDARD). This
   condition may seriously affect the device and will be an error in bitstream
   creation. It should be corrected by properly specifying the pin location and
   I/O Standard.
WARNING:PhysDesignRules:2452 - The IOB QY<21> is either not constrained (LOC) to
   a specific location and/or has an undefined I/O Standard (IOSTANDARD). This
   condition may seriously affect the device and will be an error in bitstream
   creation. It should be corrected by properly specifying the pin location and
   I/O Standard.
WARNING:PhysDesignRules:2452 - The IOB QY<22> is either not constrained (LOC) to
   a specific location and/or has an undefined I/O Standard (IOSTANDARD). This
   condition may seriously affect the device and will be an error in bitstream
   creation. It should be corrected by properly specifying the pin location and
   I/O Standard.
WARNING:PhysDesignRules:2452 - The IOB QY<23> is either not constrained (LOC) to
   a specific location and/or has an undefined I/O Standard (IOSTANDARD). This
   condition may seriously affect the device and will be an error in bitstream
   creation. It should be corrected by properly specifying the pin location and
   I/O Standard.
WARNING:PhysDesignRules:2452 - The IOB QY<24> is either not constrained (LOC) to
   a specific location and/or has an undefined I/O Standard (IOSTANDARD). This
   condition may seriously affect the device and will be an error in bitstream
   creation. It should be corrected by properly specifying the pin location and
   I/O Standard.
WARNING:PhysDesignRules:2452 - The IOB QY<25> is either not constrained (LOC) to
   a specific location and/or has an undefined I/O Standard (IOSTANDARD). This
   condition may seriously affect the device and will be an error in bitstream
   creation. It should be corrected by properly specifying the pin location and
   I/O Standard.
WARNING:PhysDesignRules:2452 - The IOB QY<26> is either not constrained (LOC) to
   a specific location and/or has an undefined I/O Standard (IOSTANDARD). This
   condition may seriously affect the device and will be an error in bitstream
   creation. It should be corrected by properly specifying the pin location and
   I/O Standard.
WARNING:PhysDesignRules:2452 - The IOB QY<27> is either not constrained (LOC) to
   a specific location and/or has an undefined I/O Standard (IOSTANDARD). This
   condition may seriously affect the device and will be an error in bitstream
   creation. It should be corrected by properly specifying the pin location and
```

```
   I/O Standard.
WARNING:PhysDesignRules:2452 - The IOB QY<28> is either not constrained (LOC) to
   a specific location and/or has an undefined I/O Standard (IOSTANDARD). This
   condition may seriously affect the device and will be an error in bitstream
   creation. It should be corrected by properly specifying the pin location and
   I/O Standard.
WARNING:PhysDesignRules:2452 - The IOB QY<29> is either not constrained (LOC) to
   a specific location and/or has an undefined I/O Standard (IOSTANDARD). This
   condition may seriously affect the device and will be an error in bitstream
   creation. It should be corrected by properly specifying the pin location and
   I/O Standard.
WARNING:PhysDesignRules:2452 - The IOB QY<30> is either not constrained (LOC) to
   a specific location and/or has an undefined I/O Standard (IOSTANDARD). This
   condition may seriously affect the device and will be an error in bitstream
   creation. It should be corrected by properly specifying the pin location and
   I/O Standard.
WARNING:PhysDesignRules:2452 - The IOB QY<31> is either not constrained (LOC) to
   a specific location and/or has an undefined I/O Standard (IOSTANDARD). This
   condition may seriously affect the device and will be an error in bitstream
   creation. It should be corrected by properly specifying the pin location and
   I/O Standard.
WARNING:PhysDesignRules:2452 - The IOB QY<32> is either not constrained (LOC) to
   a specific location and/or has an undefined I/O Standard (IOSTANDARD). This
   condition may seriously affect the device and will be an error in bitstream
   creation. It should be corrected by properly specifying the pin location and
   I/O Standard.
WARNING:PhysDesignRules:2452 - The IOB QY<33> is either not constrained (LOC) to
   a specific location and/or has an undefined I/O Standard (IOSTANDARD). This
   condition may seriously affect the device and will be an error in bitstream
   creation. It should be corrected by properly specifying the pin location and
   I/O Standard.
WARNING:PhysDesignRules:2452 - The IOB QY<34> is either not constrained (LOC) to
   a specific location and/or has an undefined I/O Standard (IOSTANDARD). This
   condition may seriously affect the device and will be an error in bitstream
   creation. It should be corrected by properly specifying the pin location and
   I/O Standard.
WARNING:PhysDesignRules:2452 - The IOB QY<35> is either not constrained (LOC) to
   a specific location and/or has an undefined I/O Standard (IOSTANDARD). This
   condition may seriously affect the device and will be an error in bitstream
   creation. It should be corrected by properly specifying the pin location and
   I/O Standard.
WARNING:PhysDesignRules:2452 - The IOB QY<36> is either not constrained (LOC) to
   a specific location and/or has an undefined I/O Standard (IOSTANDARD). This
   condition may seriously affect the device and will be an error in bitstream
   creation. It should be corrected by properly specifying the pin location and
   I/O Standard.
WARNING:PhysDesignRules:2452 - The IOB QY<37> is either not constrained (LOC) to
   a specific location and/or has an undefined I/O Standard (IOSTANDARD). This
   condition may seriously affect the device and will be an error in bitstream
   creation. It should be corrected by properly specifying the pin location and
   I/O Standard.
WARNING:PhysDesignRules:2452 - The IOB QY<38> is either not constrained (LOC) to
   a specific location and/or has an undefined I/O Standard (IOSTANDARD). This
   condition may seriously affect the device and will be an error in bitstream
   creation. It should be corrected by properly specifying the pin location and
   I/O Standard.
WARNING:PhysDesignRules:2452 - The IOB QY<39> is either not constrained (LOC) to
   a specific location and/or has an undefined I/O Standard (IOSTANDARD). This
   condition may seriously affect the device and will be an error in bitstream
   creation. It should be corrected by properly specifying the pin location and
   I/O Standard.
WARNING:PhysDesignRules:2452 - The IOB QY<40> is either not constrained (LOC) to
   a specific location and/or has an undefined I/O Standard (IOSTANDARD). This
   condition may seriously affect the device and will be an error in bitstream
   creation. It should be corrected by properly specifying the pin location and
   I/O Standard.
WARNING:PhysDesignRules:2452 - The IOB QY<41> is either not constrained (LOC) to
   a specific location and/or has an undefined I/O Standard (IOSTANDARD). This
   condition may seriously affect the device and will be an error in bitstream
   creation. It should be corrected by properly specifying the pin location and
   I/O Standard.
```

```
WARNING:PhysDesignRules:2452 - The IOB QY<42> is either not constrained (LOC) to
   a specific location and/or has an undefined I/O Standard (IOSTANDARD). This
   condition may seriously affect the device and will be an error in bitstream
   creation. It should be corrected by properly specifying the pin location and
   I/O Standard.
WARNING:PhysDesignRules:2452 - The IOB QY<43> is either not constrained (LOC) to
   a specific location and/or has an undefined I/O Standard (IOSTANDARD). This
   condition may seriously affect the device and will be an error in bitstream
   creation. It should be corrected by properly specifying the pin location and
   I/O Standard.
WARNING:PhysDesignRules:2452 - The IOB QY<44> is either not constrained (LOC) to
   a specific location and/or has an undefined I/O Standard (IOSTANDARD). This
   condition may seriously affect the device and will be an error in bitstream
   creation. It should be corrected by properly specifying the pin location and
   I/O Standard.
WARNING:PhysDesignRules:2452 - The IOB QY<45> is either not constrained (LOC) to
   a specific location and/or has an undefined I/O Standard (IOSTANDARD). This
   condition may seriously affect the device and will be an error in bitstream
   creation. It should be corrected by properly specifying the pin location and
   I/O Standard.
WARNING:PhysDesignRules:2452 - The IOB QY<46> is either not constrained (LOC) to
   a specific location and/or has an undefined I/O Standard (IOSTANDARD). This
   condition may seriously affect the device and will be an error in bitstream
   creation. It should be corrected by properly specifying the pin location and
   I/O Standard.
WARNING:PhysDesignRules:2452 - The IOB QY<47> is either not constrained (LOC) to
   a specific location and/or has an undefined I/O Standard (IOSTANDARD). This
   condition may seriously affect the device and will be an error in bitstream
   creation. It should be corrected by properly specifying the pin location and
   I/O Standard.
WARNING:PhysDesignRules:2452 - The IOB QY<48> is either not constrained (LOC) to
   a specific location and/or has an undefined I/O Standard (IOSTANDARD). This
   condition may seriously affect the device and will be an error in bitstream
   creation. It should be corrected by properly specifying the pin location and
   I/O Standard.
WARNING:PhysDesignRules:2452 - The IOB QY<49> is either not constrained (LOC) to
   a specific location and/or has an undefined I/O Standard (IOSTANDARD). This
   condition may seriously affect the device and will be an error in bitstream
   creation. It should be corrected by properly specifying the pin location and
   I/O Standard.
WARNING:PhysDesignRules:2452 - The IOB QY<50> is either not constrained (LOC) to
   a specific location and/or has an undefined I/O Standard (IOSTANDARD). This
   condition may seriously affect the device and will be an error in bitstream
   creation. It should be corrected by properly specifying the pin location and
   I/O Standard.
WARNING:PhysDesignRules:2452 - The IOB QY<51> is either not constrained (LOC) to
   a specific location and/or has an undefined I/O Standard (IOSTANDARD). This
   condition may seriously affect the device and will be an error in bitstream
   creation. It should be corrected by properly specifying the pin location and
   I/O Standard.
WARNING:PhysDesignRules:2452 - The IOB QY<52> is either not constrained (LOC) to
   a specific location and/or has an undefined I/O Standard (IOSTANDARD). This
   condition may seriously affect the device and will be an error in bitstream
   creation. It should be corrected by properly specifying the pin location and
   I/O Standard.
WARNING:PhysDesignRules:2452 - The IOB QY<53> is either not constrained (LOC) to
   a specific location and/or has an undefined I/O Standard (IOSTANDARD). This
   condition may seriously affect the device and will be an error in bitstream
   creation. It should be corrected by properly specifying the pin location and
   I/O Standard.
WARNING:PhysDesignRules:2452 - The IOB QY<54> is either not constrained (LOC) to
   a specific location and/or has an undefined I/O Standard (IOSTANDARD). This
   condition may seriously affect the device and will be an error in bitstream
   creation. It should be corrected by properly specifying the pin location and
   I/O Standard.
WARNING:PhysDesignRules:2452 - The IOB QY<55> is either not constrained (LOC) to
   a specific location and/or has an undefined I/O Standard (IOSTANDARD). This
   condition may seriously affect the device and will be an error in bitstream
   creation. It should be corrected by properly specifying the pin location and
   I/O Standard.
WARNING:PhysDesignRules:2452 - The IOB QY<56> is either not constrained (LOC) to
```

```
   a specific location and/or has an undefined I/O Standard (IOSTANDARD). This
   condition may seriously affect the device and will be an error in bitstream
   creation. It should be corrected by properly specifying the pin location and
   I/O Standard.
WARNING:PhysDesignRules:2452 - The IOB QY<57> is either not constrained (LOC) to
   a specific location and/or has an undefined I/O Standard (IOSTANDARD). This
   condition may seriously affect the device and will be an error in bitstream
   creation. It should be corrected by properly specifying the pin location and
   I/O Standard.
WARNING:PhysDesignRules:2452 - The IOB QY<58> is either not constrained (LOC) to
   a specific location and/or has an undefined I/O Standard (IOSTANDARD). This
   condition may seriously affect the device and will be an error in bitstream
   creation. It should be corrected by properly specifying the pin location and
   I/O Standard.
WARNING:PhysDesignRules:2452 - The IOB QY<59> is either not constrained (LOC) to
   a specific location and/or has an undefined I/O Standard (IOSTANDARD). This
   condition may seriously affect the device and will be an error in bitstream
   creation. It should be corrected by properly specifying the pin location and
   I/O Standard.
WARNING:PhysDesignRules:2452 - The IOB QY<60> is either not constrained (LOC) to
   a specific location and/or has an undefined I/O Standard (IOSTANDARD). This
   condition may seriously affect the device and will be an error in bitstream
   creation. It should be corrected by properly specifying the pin location and
   I/O Standard.
WARNING:PhysDesignRules:2452 - The IOB QY<61> is either not constrained (LOC) to
   a specific location and/or has an undefined I/O Standard (IOSTANDARD). This
   condition may seriously affect the device and will be an error in bitstream
   creation. It should be corrected by properly specifying the pin location and
   I/O Standard.
WARNING:PhysDesignRules:2452 - The IOB QY<62> is either not constrained (LOC) to
   a specific location and/or has an undefined I/O Standard (IOSTANDARD). This
   condition may seriously affect the device and will be an error in bitstream
   creation. It should be corrected by properly specifying the pin location and
   I/O Standard.
WARNING:PhysDesignRules:2452 - The IOB QY<63> is either not constrained (LOC) to
   a specific location and/or has an undefined I/O Standard (IOSTANDARD). This
   condition may seriously affect the device and will be an error in bitstream
   creation. It should be corrected by properly specifying the pin location and
   I/O Standard.
WARNING:PhysDesignRules:2452 - The IOB QY<64> is either not constrained (LOC) to
   a specific location and/or has an undefined I/O Standard (IOSTANDARD). This
   condition may seriously affect the device and will be an error in bitstream
   creation. It should be corrected by properly specifying the pin location and
   I/O Standard.
WARNING:PhysDesignRules:2452 - The IOB QY<65> is either not constrained (LOC) to
   a specific location and/or has an undefined I/O Standard (IOSTANDARD). This
   condition may seriously affect the device and will be an error in bitstream
   creation. It should be corrected by properly specifying the pin location and
   I/O Standard.
WARNING:PhysDesignRules:2452 - The IOB QY<66> is either not constrained (LOC) to
   a specific location and/or has an undefined I/O Standard (IOSTANDARD). This
   condition may seriously affect the device and will be an error in bitstream
   creation. It should be corrected by properly specifying the pin location and
   I/O Standard.
WARNING:PhysDesignRules:2452 - The IOB QY<67> is either not constrained (LOC) to
   a specific location and/or has an undefined I/O Standard (IOSTANDARD). This
   condition may seriously affect the device and will be an error in bitstream
   creation. It should be corrected by properly specifying the pin location and
   I/O Standard.
WARNING:PhysDesignRules:2452 - The IOB QY<68> is either not constrained (LOC) to
   a specific location and/or has an undefined I/O Standard (IOSTANDARD). This
   condition may seriously affect the device and will be an error in bitstream
   creation. It should be corrected by properly specifying the pin location and
   I/O Standard.
WARNING:PhysDesignRules:2452 - The IOB QY<69> is either not constrained (LOC) to
   a specific location and/or has an undefined I/O Standard (IOSTANDARD). This
   condition may seriously affect the device and will be an error in bitstream
   creation. It should be corrected by properly specifying the pin location and
   I/O Standard.
WARNING:PhysDesignRules:2452 - The IOB QY<70> is either not constrained (LOC) to
   a specific location and/or has an undefined I/O Standard (IOSTANDARD). This
```

```
   condition may seriously affect the device and will be an error in bitstream
   creation. It should be corrected by properly specifying the pin location and
   I/O Standard.
WARNING:PhysDesignRules:2452 - The IOB QY<71> is either not constrained (LOC) to
   a specific location and/or has an undefined I/O Standard (IOSTANDARD). This
   condition may seriously affect the device and will be an error in bitstream
   creation. It should be corrected by properly specifying the pin location and
   I/O Standard.
WARNING:PhysDesignRules:2452 - The IOB QY<72> is either not constrained (LOC) to
   a specific location and/or has an undefined I/O Standard (IOSTANDARD). This
   condition may seriously affect the device and will be an error in bitstream
   creation. It should be corrected by properly specifying the pin location and
   I/O Standard.
WARNING:PhysDesignRules:2452 - The IOB QY<73> is either not constrained (LOC) to
   a specific location and/or has an undefined I/O Standard (IOSTANDARD). This
   condition may seriously affect the device and will be an error in bitstream
   creation. It should be corrected by properly specifying the pin location and
   I/O Standard.
WARNING:PhysDesignRules:2452 - The IOB QY<74> is either not constrained (LOC) to
   a specific location and/or has an undefined I/O Standard (IOSTANDARD). This
   condition may seriously affect the device and will be an error in bitstream
   creation. It should be corrected by properly specifying the pin location and
   I/O Standard.
WARNING:PhysDesignRules:2452 - The IOB QY<75> is either not constrained (LOC) to
   a specific location and/or has an undefined I/O Standard (IOSTANDARD). This
   condition may seriously affect the device and will be an error in bitstream
   creation. It should be corrected by properly specifying the pin location and
   I/O Standard.
WARNING:PhysDesignRules:2452 - The IOB QY<76> is either not constrained (LOC) to
   a specific location and/or has an undefined I/O Standard (IOSTANDARD). This
   condition may seriously affect the device and will be an error in bitstream
   creation. It should be corrected by properly specifying the pin location and
   I/O Standard.
WARNING:PhysDesignRules:2452 - The IOB QY<77> is either not constrained (LOC) to
   a specific location and/or has an undefined I/O Standard (IOSTANDARD). This
   condition may seriously affect the device and will be an error in bitstream
   creation. It should be corrected by properly specifying the pin location and
   I/O Standard.
WARNING:PhysDesignRules:2452 - The IOB QY<78> is either not constrained (LOC) to
   a specific location and/or has an undefined I/O Standard (IOSTANDARD). This
   condition may seriously affect the device and will be an error in bitstream
   creation. It should be corrected by properly specifying the pin location and
   I/O Standard.
WARNING:PhysDesignRules:2452 - The IOB QY<79> is either not constrained (LOC) to
   a specific location and/or has an undefined I/O Standard (IOSTANDARD). This
   condition may seriously affect the device and will be an error in bitstream
   creation. It should be corrected by properly specifying the pin location and
   I/O Standard.
WARNING:PhysDesignRules:2452 - The IOB QY<80> is either not constrained (LOC) to
   a specific location and/or has an undefined I/O Standard (IOSTANDARD). This
   condition may seriously affect the device and will be an error in bitstream
   creation. It should be corrected by properly specifying the pin location and
   I/O Standard.
WARNING:PhysDesignRules:2452 - The IOB QY<81> is either not constrained (LOC) to
   a specific location and/or has an undefined I/O Standard (IOSTANDARD). This
   condition may seriously affect the device and will be an error in bitstream
   creation. It should be corrected by properly specifying the pin location and
   I/O Standard.
WARNING:PhysDesignRules:2452 - The IOB QY<82> is either not constrained (LOC) to
   a specific location and/or has an undefined I/O Standard (IOSTANDARD). This
   condition may seriously affect the device and will be an error in bitstream
   creation. It should be corrected by properly specifying the pin location and
   I/O Standard.
WARNING:PhysDesignRules:2452 - The IOB QY<83> is either not constrained (LOC) to
   a specific location and/or has an undefined I/O Standard (IOSTANDARD). This
   condition may seriously affect the device and will be an error in bitstream
   creation. It should be corrected by properly specifying the pin location and
   I/O Standard.
WARNING:PhysDesignRules:2452 - The IOB QY<84> is either not constrained (LOC) to
   a specific location and/or has an undefined I/O Standard (IOSTANDARD). This
   condition may seriously affect the device and will be an error in bitstream
```

```
   creation. It should be corrected by properly specifying the pin location and
   I/O Standard.
WARNING:PhysDesignRules:2452 - The IOB QY<85> is either not constrained (LOC) to
   a specific location and/or has an undefined I/O Standard (IOSTANDARD). This
   condition may seriously affect the device and will be an error in bitstream
   creation. It should be corrected by properly specifying the pin location and
   I/O Standard.
WARNING:PhysDesignRules:2452 - The IOB QY<86> is either not constrained (LOC) to
   a specific location and/or has an undefined I/O Standard (IOSTANDARD). This
   condition may seriously affect the device and will be an error in bitstream
   creation. It should be corrected by properly specifying the pin location and
   I/O Standard.
WARNING:PhysDesignRules:2452 - The IOB QY<87> is either not constrained (LOC) to
   a specific location and/or has an undefined I/O Standard (IOSTANDARD). This
   condition may seriously affect the device and will be an error in bitstream
   creation. It should be corrected by properly specifying the pin location and
   I/O Standard.
WARNING:PhysDesignRules:2452 - The IOB QY<88> is either not constrained (LOC) to
   a specific location and/or has an undefined I/O Standard (IOSTANDARD). This
   condition may seriously affect the device and will be an error in bitstream
   creation. It should be corrected by properly specifying the pin location and
   I/O Standard.
WARNING:PhysDesignRules:2452 - The IOB QY<89> is either not constrained (LOC) to
   a specific location and/or has an undefined I/O Standard (IOSTANDARD). This
   condition may seriously affect the device and will be an error in bitstream
   creation. It should be corrected by properly specifying the pin location and
   I/O Standard.
WARNING:PhysDesignRules:2452 - The IOB QY<90> is either not constrained (LOC) to
   a specific location and/or has an undefined I/O Standard (IOSTANDARD). This
   condition may seriously affect the device and will be an error in bitstream
   creation. It should be corrected by properly specifying the pin location and
   I/O Standard.
WARNING:PhysDesignRules:2452 - The IOB QY<91> is either not constrained (LOC) to
   a specific location and/or has an undefined I/O Standard (IOSTANDARD). This
   condition may seriously affect the device and will be an error in bitstream
   creation. It should be corrected by properly specifying the pin location and
   I/O Standard.
WARNING:PhysDesignRules:2452 - The IOB QY<92> is either not constrained (LOC) to
   a specific location and/or has an undefined I/O Standard (IOSTANDARD). This
   condition may seriously affect the device and will be an error in bitstream
   creation. It should be corrected by properly specifying the pin location and
   I/O Standard.
WARNING:PhysDesignRules:2452 - The IOB QY<93> is either not constrained (LOC) to
   a specific location and/or has an undefined I/O Standard (IOSTANDARD). This
   condition may seriously affect the device and will be an error in bitstream
   creation. It should be corrected by properly specifying the pin location and
   I/O Standard.
WARNING:PhysDesignRules:2452 - The IOB QY<94> is either not constrained (LOC) to
   a specific location and/or has an undefined I/O Standard (IOSTANDARD). This
   condition may seriously affect the device and will be an error in bitstream
   creation. It should be corrected by properly specifying the pin location and
   I/O Standard.
WARNING:PhysDesignRules:2452 - The IOB QY<95> is either not constrained (LOC) to
   a specific location and/or has an undefined I/O Standard (IOSTANDARD). This
   condition may seriously affect the device and will be an error in bitstream
   creation. It should be corrected by properly specifying the pin location and
   I/O Standard.
WARNING:PhysDesignRules:2452 - The IOB QY<96> is either not constrained (LOC) to
   a specific location and/or has an undefined I/O Standard (IOSTANDARD). This
   condition may seriously affect the device and will be an error in bitstream
   creation. It should be corrected by properly specifying the pin location and
   I/O Standard.
WARNING:PhysDesignRules:2452 - The IOB QY<97> is either not constrained (LOC) to
   a specific location and/or has an undefined I/O Standard (IOSTANDARD). This
   condition may seriously affect the device and will be an error in bitstream
   creation. It should be corrected by properly specifying the pin location and
   I/O Standard.
WARNING:PhysDesignRules:2452 - The IOB QY<98> is either not constrained (LOC) to
   a specific location and/or has an undefined I/O Standard (IOSTANDARD). This
   condition may seriously affect the device and will be an error in bitstream
   creation. It should be corrected by properly specifying the pin location and
```

I/O Standard.

WARNING:PhysDesignRules:2452 - The IOB QY<99> is either not constrained (LOC) to a specific location and/or has an undefined I/O Standard (IOSTANDARD). This condition may seriously affect the device and will be an error in bitstream creation. It should be corrected by properly specifying the pin location and I/O Standard.

WARNING:PhysDesignRules:2452 - The IOB clk is either not constrained (LOC) to a specific location and/or has an undefined I/O Standard (IOSTANDARD). This condition may seriously affect the device and will be an error in bitstream creation. It should be corrected by properly specifying the pin location and I/O Standard.

WARNING:PhysDesignRules:2452 - The IOB QY<102> is either not constrained (LOC) to a specific location and/or has an undefined I/O Standard (IOSTANDARD). This condition may seriously affect the device and will be an error in bitstream creation. It should be corrected by properly specifying the pin location and I/O Standard.

WARNING:PhysDesignRules:2452 - The IOB QY<101> is either not constrained (LOC) to a specific location and/or has an undefined I/O Standard (IOSTANDARD). This condition may seriously affect the device and will be an error in bitstream creation. It should be corrected by properly specifying the pin location and I/O Standard.

WARNING:PhysDesignRules:2452 - The IOB QY<104> is either not constrained (LOC) to a specific location and/or has an undefined I/O Standard (IOSTANDARD). This condition may seriously affect the device and will be an error in bitstream creation. It should be corrected by properly specifying the pin location and I/O Standard.

WARNING:PhysDesignRules:2452 - The IOB QY<103> is either not constrained (LOC) to a specific location and/or has an undefined I/O Standard (IOSTANDARD). This condition may seriously affect the device and will be an error in bitstream creation. It should be corrected by properly specifying the pin location and I/O Standard.

WARNING:PhysDesignRules:2452 - The IOB QY<100> is either not constrained (LOC) to a specific location and/or has an undefined I/O Standard (IOSTANDARD). This condition may seriously affect the device and will be an error in bitstream creation. It should be corrected by properly specifying the pin location and I/O Standard.

WARNING:PhysDesignRules:2452 - The IOB QY<109> is either not constrained (LOC) to a specific location and/or has an undefined I/O Standard (IOSTANDARD). This condition may seriously affect the device and will be an error in bitstream creation. It should be corrected by properly specifying the pin location and I/O Standard.

WARNING:PhysDesignRules:2452 - The IOB QY<106> is either not constrained (LOC) to a specific location and/or has an undefined I/O Standard (IOSTANDARD). This condition may seriously affect the device and will be an error in bitstream creation. It should be corrected by properly specifying the pin location and I/O Standard.

WARNING:PhysDesignRules:2452 - The IOB QY<105> is either not constrained (LOC) to a specific location and/or has an undefined I/O Standard (IOSTANDARD). This condition may seriously affect the device and will be an error in bitstream creation. It should be corrected by properly specifying the pin location and I/O Standard.

WARNING:PhysDesignRules:2452 - The IOB QY<108> is either not constrained (LOC) to a specific location and/or has an undefined I/O Standard (IOSTANDARD). This condition may seriously affect the device and will be an error in bitstream creation. It should be corrected by properly specifying the pin location and I/O Standard.

WARNING:PhysDesignRules:2452 - The IOB QY<107> is either not constrained (LOC) to a specific location and/or has an undefined I/O Standard (IOSTANDARD). This condition may seriously affect the device and will be an error in bitstream creation. It should be corrected by properly specifying the pin location and I/O Standard.

WARNING:PhysDesignRules:2452 - The IOB QY<112> is either not constrained (LOC) to a specific location and/or has an undefined I/O Standard (IOSTANDARD). This condition may seriously affect the device and will be an error in bitstream creation. It should be corrected by properly specifying the pin location and I/O Standard.

WARNING:PhysDesignRules:2452 - The IOB QY<111> is either not constrained (LOC) to a specific location and/or has an undefined I/O Standard (IOSTANDARD). This condition may seriously affect the device and will be an error in bitstream creation. It should be corrected by properly specifying the pin location and I/O Standard.

```
WARNING:PhysDesignRules:2452 - The IOB QY<114> is either not constrained (LOC)
   to a specific location and/or has an undefined I/O Standard (IOSTANDARD).
   This condition may seriously affect the device and will be an error in
   bitstream creation. It should be corrected by properly specifying the pin
   location and I/O Standard.
WARNING:PhysDesignRules:2452 - The IOB QY<113> is either not constrained (LOC)
   to a specific location and/or has an undefined I/O Standard (IOSTANDARD).
   This condition may seriously affect the device and will be an error in
   bitstream creation. It should be corrected by properly specifying the pin
   location and I/O Standard.
WARNING:PhysDesignRules:2452 - The IOB QY<110> is either not constrained (LOC)
   to a specific location and/or has an undefined I/O Standard (IOSTANDARD).
   This condition may seriously affect the device and will be an error in
   bitstream creation. It should be corrected by properly specifying the pin
   location and I/O Standard.
WARNING:PhysDesignRules:2452 - The IOB QY<119> is either not constrained (LOC)
   to a specific location and/or has an undefined I/O Standard (IOSTANDARD).
   This condition may seriously affect the device and will be an error in
   bitstream creation. It should be corrected by properly specifying the pin
   location and I/O Standard.
WARNING:PhysDesignRules:2452 - The IOB QY<116> is either not constrained (LOC)
   to a specific location and/or has an undefined I/O Standard (IOSTANDARD).
   This condition may seriously affect the device and will be an error in
   bitstream creation. It should be corrected by properly specifying the pin
   location and I/O Standard.
WARNING:PhysDesignRules:2452 - The IOB QY<115> is either not constrained (LOC)
   to a specific location and/or has an undefined I/O Standard (IOSTANDARD).
   This condition may seriously affect the device and will be an error in
   bitstream creation. It should be corrected by properly specifying the pin
   location and I/O Standard.
WARNING:PhysDesignRules:2452 - The IOB QY<118> is either not constrained (LOC)
   to a specific location and/or has an undefined I/O Standard (IOSTANDARD).
   This condition may seriously affect the device and will be an error in
   bitstream creation. It should be corrected by properly specifying the pin
   location and I/O Standard.
WARNING:PhysDesignRules:2452 - The IOB QY<117> is either not constrained (LOC)
   to a specific location and/or has an undefined I/O Standard (IOSTANDARD).
   This condition may seriously affect the device and will be an error in
   bitstream creation. It should be corrected by properly specifying the pin
   location and I/O Standard.
WARNING:PhysDesignRules:2452 - The IOB QY<122> is either not constrained (LOC)
   to a specific location and/or has an undefined I/O Standard (IOSTANDARD).
   This condition may seriously affect the device and will be an error in
   bitstream creation. It should be corrected by properly specifying the pin
   location and I/O Standard.
WARNING:PhysDesignRules:2452 - The IOB QY<121> is either not constrained (LOC)
   to a specific location and/or has an undefined I/O Standard (IOSTANDARD).
   This condition may seriously affect the device and will be an error in
   bitstream creation. It should be corrected by properly specifying the pin
   location and I/O Standard.
WARNING:PhysDesignRules:2452 - The IOB QY<124> is either not constrained (LOC)
   to a specific location and/or has an undefined I/O Standard (IOSTANDARD).
   This condition may seriously affect the device and will be an error in
   bitstream creation. It should be corrected by properly specifying the pin
   location and I/O Standard.
WARNING:PhysDesignRules:2452 - The IOB QY<123> is either not constrained (LOC)
   to a specific location and/or has an undefined I/O Standard (IOSTANDARD).
   This condition may seriously affect the device and will be an error in
   bitstream creation. It should be corrected by properly specifying the pin
   location and I/O Standard.
WARNING:PhysDesignRules:2452 - The IOB QY<120> is either not constrained (LOC)
   to a specific location and/or has an undefined I/O Standard (IOSTANDARD).
   This condition may seriously affect the device and will be an error in
   bitstream creation. It should be corrected by properly specifying the pin
   location and I/O Standard.
WARNING:PhysDesignRules:2452 - The IOB QY<129> is either not constrained (LOC)
   to a specific location and/or has an undefined I/O Standard (IOSTANDARD).
   This condition may seriously affect the device and will be an error in
   bitstream creation. It should be corrected by properly specifying the pin
   location and I/O Standard.
WARNING:PhysDesignRules:2452 - The IOB QY<126> is either not constrained (LOC)
```

```
   to a specific location and/or has an undefined I/O Standard (IOSTANDARD).
   This condition may seriously affect the device and will be an error in
   bitstream creation. It should be corrected by properly specifying the pin
   location and I/O Standard.
WARNING:PhysDesignRules:2452 - The IOB QY<125> is either not constrained (LOC)
   to a specific location and/or has an undefined I/O Standard (IOSTANDARD).
   This condition may seriously affect the device and will be an error in
   bitstream creation. It should be corrected by properly specifying the pin
   location and I/O Standard.
WARNING:PhysDesignRules:2452 - The IOB QY<128> is either not constrained (LOC)
   to a specific location and/or has an undefined I/O Standard (IOSTANDARD).
   This condition may seriously affect the device and will be an error in
   bitstream creation. It should be corrected by properly specifying the pin
   location and I/O Standard.
WARNING:PhysDesignRules:2452 - The IOB QY<127> is either not constrained (LOC)
   to a specific location and/or has an undefined I/O Standard (IOSTANDARD).
   This condition may seriously affect the device and will be an error in
   bitstream creation. It should be corrected by properly specifying the pin
   location and I/O Standard.
WARNING:PhysDesignRules:2452 - The IOB QY<132> is either not constrained (LOC)
   to a specific location and/or has an undefined I/O Standard (IOSTANDARD).
   This condition may seriously affect the device and will be an error in
   bitstream creation. It should be corrected by properly specifying the pin
   location and I/O Standard.
WARNING:PhysDesignRules:2452 - The IOB QY<131> is either not constrained (LOC)
   to a specific location and/or has an undefined I/O Standard (IOSTANDARD).
   This condition may seriously affect the device and will be an error in
   bitstream creation. It should be corrected by properly specifying the pin
   location and I/O Standard.
WARNING:PhysDesignRules:2452 - The IOB QY<134> is either not constrained (LOC)
   to a specific location and/or has an undefined I/O Standard (IOSTANDARD).
   This condition may seriously affect the device and will be an error in
   bitstream creation. It should be corrected by properly specifying the pin
   location and I/O Standard.
WARNING:PhysDesignRules:2452 - The IOB QY<133> is either not constrained (LOC)
   to a specific location and/or has an undefined I/O Standard (IOSTANDARD).
   This condition may seriously affect the device and will be an error in
   bitstream creation. It should be corrected by properly specifying the pin
   location and I/O Standard.
WARNING:PhysDesignRules:2452 - The IOB QY<130> is either not constrained (LOC)
   to a specific location and/or has an undefined I/O Standard (IOSTANDARD).
   This condition may seriously affect the device and will be an error in
   bitstream creation. It should be corrected by properly specifying the pin
   location and I/O Standard.
WARNING:PhysDesignRules:2452 - The IOB QY<139> is either not constrained (LOC)
   to a specific location and/or has an undefined I/O Standard (IOSTANDARD).
   This condition may seriously affect the device and will be an error in
   bitstream creation. It should be corrected by properly specifying the pin
   location and I/O Standard.
WARNING:PhysDesignRules:2452 - The IOB QY<136> is either not constrained (LOC)
   to a specific location and/or has an undefined I/O Standard (IOSTANDARD).
   This condition may seriously affect the device and will be an error in
   bitstream creation. It should be corrected by properly specifying the pin
   location and I/O Standard.
WARNING:PhysDesignRules:2452 - The IOB QY<135> is either not constrained (LOC)
   to a specific location and/or has an undefined I/O Standard (IOSTANDARD).
   This condition may seriously affect the device and will be an error in
   bitstream creation. It should be corrected by properly specifying the pin
   location and I/O Standard.
WARNING:PhysDesignRules:2452 - The IOB QY<138> is either not constrained (LOC)
   to a specific location and/or has an undefined I/O Standard (IOSTANDARD).
   This condition may seriously affect the device and will be an error in
   bitstream creation. It should be corrected by properly specifying the pin
   location and I/O Standard.
WARNING:PhysDesignRules:2452 - The IOB QY<137> is either not constrained (LOC)
   to a specific location and/or has an undefined I/O Standard (IOSTANDARD).
   This condition may seriously affect the device and will be an error in
   bitstream creation. It should be corrected by properly specifying the pin
   location and I/O Standard.
WARNING:PhysDesignRules:2452 - The IOB QY<142> is either not constrained (LOC)
   to a specific location and/or has an undefined I/O Standard (IOSTANDARD).
```

```
   This condition may seriously affect the device and will be an error in
   bitstream creation. It should be corrected by properly specifying the pin
   location and I/O Standard.
WARNING:PhysDesignRules:2452 - The IOB QY<141> is either not constrained (LOC)
   to a specific location and/or has an undefined I/O Standard (IOSTANDARD).
   This condition may seriously affect the device and will be an error in
   bitstream creation. It should be corrected by properly specifying the pin
   location and I/O Standard.
WARNING:PhysDesignRules:2452 - The IOB QY<144> is either not constrained (LOC)
   to a specific location and/or has an undefined I/O Standard (IOSTANDARD).
   This condition may seriously affect the device and will be an error in
   bitstream creation. It should be corrected by properly specifying the pin
   location and I/O Standard.
WARNING:PhysDesignRules:2452 - The IOB QY<143> is either not constrained (LOC)
   to a specific location and/or has an undefined I/O Standard (IOSTANDARD).
   This condition may seriously affect the device and will be an error in
   bitstream creation. It should be corrected by properly specifying the pin
   location and I/O Standard.
WARNING:PhysDesignRules:2452 - The IOB QY<140> is either not constrained (LOC)
   to a specific location and/or has an undefined I/O Standard (IOSTANDARD).
   This condition may seriously affect the device and will be an error in
   bitstream creation. It should be corrected by properly specifying the pin
   location and I/O Standard.
WARNING:PhysDesignRules:2452 - The IOB QY<149> is either not constrained (LOC)
   to a specific location and/or has an undefined I/O Standard (IOSTANDARD).
   This condition may seriously affect the device and will be an error in
   bitstream creation. It should be corrected by properly specifying the pin
   location and I/O Standard.
WARNING:PhysDesignRules:2452 - The IOB QY<146> is either not constrained (LOC)
   to a specific location and/or has an undefined I/O Standard (IOSTANDARD).
   This condition may seriously affect the device and will be an error in
   bitstream creation. It should be corrected by properly specifying the pin
   location and I/O Standard.
WARNING:PhysDesignRules:2452 - The IOB QY<145> is either not constrained (LOC)
   to a specific location and/or has an undefined I/O Standard (IOSTANDARD).
   This condition may seriously affect the device and will be an error in
   bitstream creation. It should be corrected by properly specifying the pin
   location and I/O Standard.
WARNING:PhysDesignRules:2452 - The IOB QY<148> is either not constrained (LOC)
   to a specific location and/or has an undefined I/O Standard (IOSTANDARD).
   This condition may seriously affect the device and will be an error in
   bitstream creation. It should be corrected by properly specifying the pin
   location and I/O Standard.
WARNING:PhysDesignRules:2452 - The IOB QY<147> is either not constrained (LOC)
   to a specific location and/or has an undefined I/O Standard (IOSTANDARD).
   This condition may seriously affect the device and will be an error in
   bitstream creation. It should be corrected by properly specifying the pin
   location and I/O Standard.
WARNING:PhysDesignRules:2452 - The IOB QY<152> is either not constrained (LOC)
   to a specific location and/or has an undefined I/O Standard (IOSTANDARD).
   This condition may seriously affect the device and will be an error in
   bitstream creation. It should be corrected by properly specifying the pin
   location and I/O Standard.
WARNING:PhysDesignRules:2452 - The IOB QY<151> is either not constrained (LOC)
   to a specific location and/or has an undefined I/O Standard (IOSTANDARD).
   This condition may seriously affect the device and will be an error in
   bitstream creation. It should be corrected by properly specifying the pin
   location and I/O Standard.
WARNING:PhysDesignRules:2452 - The IOB QY<154> is either not constrained (LOC)
   to a specific location and/or has an undefined I/O Standard (IOSTANDARD).
   This condition may seriously affect the device and will be an error in
   bitstream creation. It should be corrected by properly specifying the pin
   location and I/O Standard.
WARNING:PhysDesignRules:2452 - The IOB QY<153> is either not constrained (LOC)
   to a specific location and/or has an undefined I/O Standard (IOSTANDARD).
   This condition may seriously affect the device and will be an error in
   bitstream creation. It should be corrected by properly specifying the pin
   location and I/O Standard.
WARNING:PhysDesignRules:2452 - The IOB QY<150> is either not constrained (LOC)
   to a specific location and/or has an undefined I/O Standard (IOSTANDARD).
   This condition may seriously affect the device and will be an error in
```

```
   bitstream creation. It should be corrected by properly specifying the pin
   location and I/O Standard.
WARNING:PhysDesignRules:2452 - The IOB QY<159> is either not constrained (LOC)
   to a specific location and/or has an undefined I/O Standard (IOSTANDARD).
   This condition may seriously affect the device and will be an error in
   bitstream creation. It should be corrected by properly specifying the pin
   location and I/O Standard.
WARNING:PhysDesignRules:2452 - The IOB QY<156> is either not constrained (LOC)
   to a specific location and/or has an undefined I/O Standard (IOSTANDARD).
   This condition may seriously affect the device and will be an error in
   bitstream creation. It should be corrected by properly specifying the pin
   location and I/O Standard.
WARNING:PhysDesignRules:2452 - The IOB QY<155> is either not constrained (LOC)
   to a specific location and/or has an undefined I/O Standard (IOSTANDARD).
   This condition may seriously affect the device and will be an error in
   bitstream creation. It should be corrected by properly specifying the pin
   location and I/O Standard.
WARNING:PhysDesignRules:2452 - The IOB QY<158> is either not constrained (LOC)
   to a specific location and/or has an undefined I/O Standard (IOSTANDARD).
   This condition may seriously affect the device and will be an error in
   bitstream creation. It should be corrected by properly specifying the pin
   location and I/O Standard.
WARNING:PhysDesignRules:2452 - The IOB QY<157> is either not constrained (LOC)
   to a specific location and/or has an undefined I/O Standard (IOSTANDARD).
   This condition may seriously affect the device and will be an error in
   bitstream creation. It should be corrected by properly specifying the pin
   location and I/O Standard.
WARNING:PhysDesignRules:2452 - The IOB QY<162> is either not constrained (LOC)
   to a specific location and/or has an undefined I/O Standard (IOSTANDARD).
   This condition may seriously affect the device and will be an error in
   bitstream creation. It should be corrected by properly specifying the pin
   location and I/O Standard.
WARNING:PhysDesignRules:2452 - The IOB QY<161> is either not constrained (LOC)
   to a specific location and/or has an undefined I/O Standard (IOSTANDARD).
   This condition may seriously affect the device and will be an error in
   bitstream creation. It should be corrected by properly specifying the pin
   location and I/O Standard.
WARNING:PhysDesignRules:2452 - The IOB QY<164> is either not constrained (LOC)
   to a specific location and/or has an undefined I/O Standard (IOSTANDARD).
   This condition may seriously affect the device and will be an error in
   bitstream creation. It should be corrected by properly specifying the pin
   location and I/O Standard.
WARNING:PhysDesignRules:2452 - The IOB QY<163> is either not constrained (LOC)
   to a specific location and/or has an undefined I/O Standard (IOSTANDARD).
   This condition may seriously affect the device and will be an error in
   bitstream creation. It should be corrected by properly specifying the pin
   location and I/O Standard.
WARNING:PhysDesignRules:2452 - The IOB QY<160> is either not constrained (LOC)
   to a specific location and/or has an undefined I/O Standard (IOSTANDARD).
   This condition may seriously affect the device and will be an error in
   bitstream creation. It should be corrected by properly specifying the pin
   location and I/O Standard.
WARNING:PhysDesignRules:2452 - The IOB QY<169> is either not constrained (LOC)
   to a specific location and/or has an undefined I/O Standard (IOSTANDARD).
   This condition may seriously affect the device and will be an error in
   bitstream creation. It should be corrected by properly specifying the pin
   location and I/O Standard.
WARNING:PhysDesignRules:2452 - The IOB QY<166> is either not constrained (LOC)
   to a specific location and/or has an undefined I/O Standard (IOSTANDARD).
   This condition may seriously affect the device and will be an error in
   bitstream creation. It should be corrected by properly specifying the pin
   location and I/O Standard.
WARNING:PhysDesignRules:2452 - The IOB QY<165> is either not constrained (LOC)
   to a specific location and/or has an undefined I/O Standard (IOSTANDARD).
   This condition may seriously affect the device and will be an error in
   bitstream creation. It should be corrected by properly specifying the pin
   location and I/O Standard.
WARNING:PhysDesignRules:2452 - The IOB QY<168> is either not constrained (LOC)
   to a specific location and/or has an undefined I/O Standard (IOSTANDARD).
   This condition may seriously affect the device and will be an error in
   bitstream creation. It should be corrected by properly specifying the pin
```

```
   location and I/O Standard.
WARNING:PhysDesignRules:2452 - The IOB QY<167> is either not constrained (LOC)
   to a specific location and/or has an undefined I/O Standard (IOSTANDARD).
   This condition may seriously affect the device and will be an error in
   bitstream creation. It should be corrected by properly specifying the pin
   location and I/O Standard.
WARNING:PhysDesignRules:2452 - The IOB QY<172> is either not constrained (LOC)
   to a specific location and/or has an undefined I/O Standard (IOSTANDARD).
   This condition may seriously affect the device and will be an error in
   bitstream creation. It should be corrected by properly specifying the pin
   location and I/O Standard.
WARNING:PhysDesignRules:2452 - The IOB QY<171> is either not constrained (LOC)
   to a specific location and/or has an undefined I/O Standard (IOSTANDARD).
   This condition may seriously affect the device and will be an error in
   bitstream creation. It should be corrected by properly specifying the pin
   location and I/O Standard.
WARNING:PhysDesignRules:2452 - The IOB QY<174> is either not constrained (LOC)
   to a specific location and/or has an undefined I/O Standard (IOSTANDARD).
   This condition may seriously affect the device and will be an error in
   bitstream creation. It should be corrected by properly specifying the pin
   location and I/O Standard.
WARNING:PhysDesignRules:2452 - The IOB QY<173> is either not constrained (LOC)
   to a specific location and/or has an undefined I/O Standard (IOSTANDARD).
   This condition may seriously affect the device and will be an error in
   bitstream creation. It should be corrected by properly specifying the pin
   location and I/O Standard.
WARNING:PhysDesignRules:2452 - The IOB QY<170> is either not constrained (LOC)
   to a specific location and/or has an undefined I/O Standard (IOSTANDARD).
   This condition may seriously affect the device and will be an error in
   bitstream creation. It should be corrected by properly specifying the pin
   location and I/O Standard.
WARNING:PhysDesignRules:2452 - The IOB QY<179> is either not constrained (LOC)
   to a specific location and/or has an undefined I/O Standard (IOSTANDARD).
   This condition may seriously affect the device and will be an error in
   bitstream creation. It should be corrected by properly specifying the pin
   location and I/O Standard.
WARNING:PhysDesignRules:2452 - The IOB QY<176> is either not constrained (LOC)
   to a specific location and/or has an undefined I/O Standard (IOSTANDARD).
   This condition may seriously affect the device and will be an error in
   bitstream creation. It should be corrected by properly specifying the pin
   location and I/O Standard.
WARNING:PhysDesignRules:2452 - The IOB QY<175> is either not constrained (LOC)
   to a specific location and/or has an undefined I/O Standard (IOSTANDARD).
   This condition may seriously affect the device and will be an error in
   bitstream creation. It should be corrected by properly specifying the pin
   location and I/O Standard.
WARNING:PhysDesignRules:2452 - The IOB QY<178> is either not constrained (LOC)
   to a specific location and/or has an undefined I/O Standard (IOSTANDARD).
   This condition may seriously affect the device and will be an error in
   bitstream creation. It should be corrected by properly specifying the pin
   location and I/O Standard.
WARNING:PhysDesignRules:2452 - The IOB QY<177> is either not constrained (LOC)
   to a specific location and/or has an undefined I/O Standard (IOSTANDARD).
   This condition may seriously affect the device and will be an error in
   bitstream creation. It should be corrected by properly specifying the pin
   location and I/O Standard.
WARNING:PhysDesignRules:2452 - The IOB QY<182> is either not constrained (LOC)
   to a specific location and/or has an undefined I/O Standard (IOSTANDARD).
   This condition may seriously affect the device and will be an error in
   bitstream creation. It should be corrected by properly specifying the pin
   location and I/O Standard.
WARNING:PhysDesignRules:2452 - The IOB QY<181> is either not constrained (LOC)
   to a specific location and/or has an undefined I/O Standard (IOSTANDARD).
   This condition may seriously affect the device and will be an error in
   bitstream creation. It should be corrected by properly specifying the pin
   location and I/O Standard.
WARNING:PhysDesignRules:2452 - The IOB QY<184> is either not constrained (LOC)
   to a specific location and/or has an undefined I/O Standard (IOSTANDARD).
   This condition may seriously affect the device and will be an error in
   bitstream creation. It should be corrected by properly specifying the pin
   location and I/O Standard.
```

```
WARNING:PhysDesignRules:2452 - The IOB QY<183> is either not constrained (LOC)
   to a specific location and/or has an undefined I/O Standard (IOSTANDARD).
   This condition may seriously affect the device and will be an error in
   bitstream creation. It should be corrected by properly specifying the pin
   location and I/O Standard.
WARNING:PhysDesignRules:2452 - The IOB QY<180> is either not constrained (LOC)
   to a specific location and/or has an undefined I/O Standard (IOSTANDARD).
   This condition may seriously affect the device and will be an error in
   bitstream creation. It should be corrected by properly specifying the pin
   location and I/O Standard.
WARNING:PhysDesignRules:2452 - The IOB QY<189> is either not constrained (LOC)
   to a specific location and/or has an undefined I/O Standard (IOSTANDARD).
   This condition may seriously affect the device and will be an error in
   bitstream creation. It should be corrected by properly specifying the pin
   location and I/O Standard.
WARNING:PhysDesignRules:2452 - The IOB QY<186> is either not constrained (LOC)
   to a specific location and/or has an undefined I/O Standard (IOSTANDARD).
   This condition may seriously affect the device and will be an error in
   bitstream creation. It should be corrected by properly specifying the pin
   location and I/O Standard.
WARNING:PhysDesignRules:2452 - The IOB QY<185> is either not constrained (LOC)
   to a specific location and/or has an undefined I/O Standard (IOSTANDARD).
   This condition may seriously affect the device and will be an error in
   bitstream creation. It should be corrected by properly specifying the pin
   location and I/O Standard.
WARNING:PhysDesignRules:2452 - The IOB QY<188> is either not constrained (LOC)
   to a specific location and/or has an undefined I/O Standard (IOSTANDARD).
   This condition may seriously affect the device and will be an error in
   bitstream creation. It should be corrected by properly specifying the pin
   location and I/O Standard.
WARNING:PhysDesignRules:2452 - The IOB QY<187> is either not constrained (LOC)
   to a specific location and/or has an undefined I/O Standard (IOSTANDARD).
   This condition may seriously affect the device and will be an error in
   bitstream creation. It should be corrected by properly specifying the pin
   location and I/O Standard.
WARNING:PhysDesignRules:2452 - The IOB QY<192> is either not constrained (LOC)
   to a specific location and/or has an undefined I/O Standard (IOSTANDARD).
   This condition may seriously affect the device and will be an error in
   bitstream creation. It should be corrected by properly specifying the pin
   location and I/O Standard.
WARNING:PhysDesignRules:2452 - The IOB QY<191> is either not constrained (LOC)
   to a specific location and/or has an undefined I/O Standard (IOSTANDARD).
   This condition may seriously affect the device and will be an error in
   bitstream creation. It should be corrected by properly specifying the pin
   location and I/O Standard.
WARNING:PhysDesignRules:2452 - The IOB QY<194> is either not constrained (LOC)
   to a specific location and/or has an undefined I/O Standard (IOSTANDARD).
   This condition may seriously affect the device and will be an error in
   bitstream creation. It should be corrected by properly specifying the pin
   location and I/O Standard.
WARNING:PhysDesignRules:2452 - The IOB QY<193> is either not constrained (LOC)
   to a specific location and/or has an undefined I/O Standard (IOSTANDARD).
   This condition may seriously affect the device and will be an error in
   bitstream creation. It should be corrected by properly specifying the pin
   location and I/O Standard.
WARNING:PhysDesignRules:2452 - The IOB QY<190> is either not constrained (LOC)
   to a specific location and/or has an undefined I/O Standard (IOSTANDARD).
   This condition may seriously affect the device and will be an error in
   bitstream creation. It should be corrected by properly specifying the pin
   location and I/O Standard.
WARNING:PhysDesignRules:2452 - The IOB QY<199> is either not constrained (LOC)
   to a specific location and/or has an undefined I/O Standard (IOSTANDARD).
   This condition may seriously affect the device and will be an error in
   bitstream creation. It should be corrected by properly specifying the pin
   location and I/O Standard.
WARNING:PhysDesignRules:2452 - The IOB QY<196> is either not constrained (LOC)
   to a specific location and/or has an undefined I/O Standard (IOSTANDARD).
   This condition may seriously affect the device and will be an error in
   bitstream creation. It should be corrected by properly specifying the pin
   location and I/O Standard.
WARNING:PhysDesignRules:2452 - The IOB QY<195> is either not constrained (LOC)
```

```
   to a specific location and/or has an undefined I/O Standard (IOSTANDARD).
   This condition may seriously affect the device and will be an error in
   bitstream creation. It should be corrected by properly specifying the pin
   location and I/O Standard.
WARNING:PhysDesignRules:2452 - The IOB QY<198> is either not constrained (LOC)
   to a specific location and/or has an undefined I/O Standard (IOSTANDARD).
   This condition may seriously affect the device and will be an error in
   bitstream creation. It should be corrected by properly specifying the pin
   location and I/O Standard.
WARNING:PhysDesignRules:2452 - The IOB QY<197> is either not constrained (LOC)
   to a specific location and/or has an undefined I/O Standard (IOSTANDARD).
   This condition may seriously affect the device and will be an error in
   bitstream creation. It should be corrected by properly specifying the pin
   location and I/O Standard.
WARNING:PhysDesignRules:2452 - The IOB QY<202> is either not constrained (LOC)
   to a specific location and/or has an undefined I/O Standard (IOSTANDARD).
   This condition may seriously affect the device and will be an error in
   bitstream creation. It should be corrected by properly specifying the pin
   location and I/O Standard.
WARNING:PhysDesignRules:2452 - The IOB QY<201> is either not constrained (LOC)
   to a specific location and/or has an undefined I/O Standard (IOSTANDARD).
   This condition may seriously affect the device and will be an error in
   bitstream creation. It should be corrected by properly specifying the pin
   location and I/O Standard.
WARNING:PhysDesignRules:2452 - The IOB QY<204> is either not constrained (LOC)
   to a specific location and/or has an undefined I/O Standard (IOSTANDARD).
   This condition may seriously affect the device and will be an error in
   bitstream creation. It should be corrected by properly specifying the pin
   location and I/O Standard.
WARNING:PhysDesignRules:2452 - The IOB QY<203> is either not constrained (LOC)
   to a specific location and/or has an undefined I/O Standard (IOSTANDARD).
   This condition may seriously affect the device and will be an error in
   bitstream creation. It should be corrected by properly specifying the pin
   location and I/O Standard.
WARNING:PhysDesignRules:2452 - The IOB QY<200> is either not constrained (LOC)
   to a specific location and/or has an undefined I/O Standard (IOSTANDARD).
   This condition may seriously affect the device and will be an error in
   bitstream creation. It should be corrected by properly specifying the pin
   location and I/O Standard.
WARNING:PhysDesignRules:2452 - The IOB QY<209> is either not constrained (LOC)
   to a specific location and/or has an undefined I/O Standard (IOSTANDARD).
   This condition may seriously affect the device and will be an error in
   bitstream creation. It should be corrected by properly specifying the pin
   location and I/O Standard.
WARNING:PhysDesignRules:2452 - The IOB QY<206> is either not constrained (LOC)
   to a specific location and/or has an undefined I/O Standard (IOSTANDARD).
   This condition may seriously affect the device and will be an error in
   bitstream creation. It should be corrected by properly specifying the pin
   location and I/O Standard.
WARNING:PhysDesignRules:2452 - The IOB QY<205> is either not constrained (LOC)
   to a specific location and/or has an undefined I/O Standard (IOSTANDARD).
   This condition may seriously affect the device and will be an error in
   bitstream creation. It should be corrected by properly specifying the pin
   location and I/O Standard.
WARNING:PhysDesignRules:2452 - The IOB QY<208> is either not constrained (LOC)
   to a specific location and/or has an undefined I/O Standard (IOSTANDARD).
   This condition may seriously affect the device and will be an error in
   bitstream creation. It should be corrected by properly specifying the pin
   location and I/O Standard.
WARNING:PhysDesignRules:2452 - The IOB QY<207> is either not constrained (LOC)
   to a specific location and/or has an undefined I/O Standard (IOSTANDARD).
   This condition may seriously affect the device and will be an error in
   bitstream creation. It should be corrected by properly specifying the pin
   location and I/O Standard.
WARNING:PhysDesignRules:2452 - The IOB QY<212> is either not constrained (LOC)
   to a specific location and/or has an undefined I/O Standard (IOSTANDARD).
   This condition may seriously affect the device and will be an error in
   bitstream creation. It should be corrected by properly specifying the pin
   location and I/O Standard.
WARNING:PhysDesignRules:2452 - The IOB QY<211> is either not constrained (LOC)
   to a specific location and/or has an undefined I/O Standard (IOSTANDARD).
```

This condition may seriously affect the device and will be an error in bitstream creation. It should be corrected by properly specifying the pin location and I/O Standard.

WARNING:PhysDesignRules:2452 - The IOB QY<214> is either not constrained (LOC) to a specific location and/or has an undefined I/O Standard (IOSTANDARD). This condition may seriously affect the device and will be an error in bitstream creation. It should be corrected by properly specifying the pin location and I/O Standard.

WARNING:PhysDesignRules:2452 - The IOB QY<213> is either not constrained (LOC) to a specific location and/or has an undefined I/O Standard (IOSTANDARD). This condition may seriously affect the device and will be an error in bitstream creation. It should be corrected by properly specifying the pin location and I/O Standard.

WARNING:PhysDesignRules:2452 - The IOB QY<210> is either not constrained (LOC) to a specific location and/or has an undefined I/O Standard (IOSTANDARD). This condition may seriously affect the device and will be an error in bitstream creation. It should be corrected by properly specifying the pin location and I/O Standard.

WARNING:PhysDesignRules:2452 - The IOB QY<219> is either not constrained (LOC) to a specific location and/or has an undefined I/O Standard (IOSTANDARD). This condition may seriously affect the device and will be an error in bitstream creation. It should be corrected by properly specifying the pin location and I/O Standard.

WARNING:PhysDesignRules:2452 - The IOB QY<216> is either not constrained (LOC) to a specific location and/or has an undefined I/O Standard (IOSTANDARD). This condition may seriously affect the device and will be an error in bitstream creation. It should be corrected by properly specifying the pin location and I/O Standard.

WARNING:PhysDesignRules:2452 - The IOB QY<215> is either not constrained (LOC) to a specific location and/or has an undefined I/O Standard (IOSTANDARD). This condition may seriously affect the device and will be an error in bitstream creation. It should be corrected by properly specifying the pin location and I/O Standard.

WARNING:PhysDesignRules:2452 - The IOB QY<218> is either not constrained (LOC) to a specific location and/or has an undefined I/O Standard (IOSTANDARD). This condition may seriously affect the device and will be an error in bitstream creation. It should be corrected by properly specifying the pin location and I/O Standard.

WARNING:PhysDesignRules:2452 - The IOB QY<217> is either not constrained (LOC) to a specific location and/or has an undefined I/O Standard (IOSTANDARD). This condition may seriously affect the device and will be an error in bitstream creation. It should be corrected by properly specifying the pin location and I/O Standard.

WARNING:PhysDesignRules:2452 - The IOB QY<222> is either not constrained (LOC) to a specific location and/or has an undefined I/O Standard (IOSTANDARD). This condition may seriously affect the device and will be an error in bitstream creation. It should be corrected by properly specifying the pin location and I/O Standard.

WARNING:PhysDesignRules:2452 - The IOB QY<221> is either not constrained (LOC) to a specific location and/or has an undefined I/O Standard (IOSTANDARD). This condition may seriously affect the device and will be an error in bitstream creation. It should be corrected by properly specifying the pin location and I/O Standard.

WARNING:PhysDesignRules:2452 - The IOB QY<224> is either not constrained (LOC) to a specific location and/or has an undefined I/O Standard (IOSTANDARD). This condition may seriously affect the device and will be an error in bitstream creation. It should be corrected by properly specifying the pin location and I/O Standard.

WARNING:PhysDesignRules:2452 - The IOB QY<223> is either not constrained (LOC) to a specific location and/or has an undefined I/O Standard (IOSTANDARD). This condition may seriously affect the device and will be an error in bitstream creation. It should be corrected by properly specifying the pin location and I/O Standard.

WARNING:PhysDesignRules:2452 - The IOB QY<220> is either not constrained (LOC) to a specific location and/or has an undefined I/O Standard (IOSTANDARD). This condition may seriously affect the device and will be an error in bitstream creation. It should be corrected by properly specifying the pin location and I/O Standard.

WARNING:PhysDesignRules:2452 - The IOB QY<229> is either not constrained (LOC) to a specific location and/or has an undefined I/O Standard (IOSTANDARD). This condition may seriously affect the device and will be an error in

```
   bitstream creation. It should be corrected by properly specifying the pin
   location and I/O Standard.
WARNING:PhysDesignRules:2452 - The IOB QY<226> is either not constrained (LOC)
   to a specific location and/or has an undefined I/O Standard (IOSTANDARD).
   This condition may seriously affect the device and will be an error in
   bitstream creation. It should be corrected by properly specifying the pin
   location and I/O Standard.
WARNING:PhysDesignRules:2452 - The IOB QY<225> is either not constrained (LOC)
   to a specific location and/or has an undefined I/O Standard (IOSTANDARD).
   This condition may seriously affect the device and will be an error in
   bitstream creation. It should be corrected by properly specifying the pin
   location and I/O Standard.
WARNING:PhysDesignRules:2452 - The IOB QY<228> is either not constrained (LOC)
   to a specific location and/or has an undefined I/O Standard (IOSTANDARD).
   This condition may seriously affect the device and will be an error in
   bitstream creation. It should be corrected by properly specifying the pin
   location and I/O Standard.
WARNING:PhysDesignRules:2452 - The IOB QY<227> is either not constrained (LOC)
   to a specific location and/or has an undefined I/O Standard (IOSTANDARD).
   This condition may seriously affect the device and will be an error in
   bitstream creation. It should be corrected by properly specifying the pin
   location and I/O Standard.
WARNING:PhysDesignRules:2452 - The IOB QY<232> is either not constrained (LOC)
   to a specific location and/or has an undefined I/O Standard (IOSTANDARD).
   This condition may seriously affect the device and will be an error in
   bitstream creation. It should be corrected by properly specifying the pin
   location and I/O Standard.
WARNING:PhysDesignRules:2452 - The IOB QY<231> is either not constrained (LOC)
   to a specific location and/or has an undefined I/O Standard (IOSTANDARD).
   This condition may seriously affect the device and will be an error in
   bitstream creation. It should be corrected by properly specifying the pin
   location and I/O Standard.
WARNING:PhysDesignRules:2452 - The IOB QY<230> is either not constrained (LOC)
   to a specific location and/or has an undefined I/O Standard (IOSTANDARD).
   This condition may seriously affect the device and will be an error in
   bitstream creation. It should be corrected by properly specifying the pin
   location and I/O Standard.
WARNING:PhysDesignRules:2452 - The IOB QX<10> is either not constrained (LOC) to
   a specific location and/or has an undefined I/O Standard (IOSTANDARD). This
   condition may seriously affect the device and will be an error in bitstream
   creation. It should be corrected by properly specifying the pin location and
   I/O Standard.
WARNING:PhysDesignRules:2452 - The IOB QX<11> is either not constrained (LOC) to
   a specific location and/or has an undefined I/O Standard (IOSTANDARD). This
   condition may seriously affect the device and will be an error in bitstream
   creation. It should be corrected by properly specifying the pin location and
   I/O Standard.
WARNING:PhysDesignRules:2452 - The IOB QX<12> is either not constrained (LOC) to
   a specific location and/or has an undefined I/O Standard (IOSTANDARD). This
   condition may seriously affect the device and will be an error in bitstream
   creation. It should be corrected by properly specifying the pin location and
   I/O Standard.
WARNING:PhysDesignRules:2452 - The IOB QX<13> is either not constrained (LOC) to
   a specific location and/or has an undefined I/O Standard (IOSTANDARD). This
   condition may seriously affect the device and will be an error in bitstream
   creation. It should be corrected by properly specifying the pin location and
   I/O Standard.
WARNING:PhysDesignRules:2452 - The IOB QX<14> is either not constrained (LOC) to
   a specific location and/or has an undefined I/O Standard (IOSTANDARD). This
   condition may seriously affect the device and will be an error in bitstream
   creation. It should be corrected by properly specifying the pin location and
   I/O Standard.
WARNING:PhysDesignRules:2452 - The IOB QX<15> is either not constrained (LOC) to
   a specific location and/or has an undefined I/O Standard (IOSTANDARD). This
   condition may seriously affect the device and will be an error in bitstream
   creation. It should be corrected by properly specifying the pin location and
   I/O Standard.
WARNING:PhysDesignRules:2452 - The IOB QX<16> is either not constrained (LOC) to
   a specific location and/or has an undefined I/O Standard (IOSTANDARD). This
   condition may seriously affect the device and will be an error in bitstream
   creation. It should be corrected by properly specifying the pin location and
```

```
   I/O Standard.
WARNING:PhysDesignRules:2452 - The IOB QX<17> is either not constrained (LOC) to
   a specific location and/or has an undefined I/O Standard (IOSTANDARD). This
   condition may seriously affect the device and will be an error in bitstream
   creation. It should be corrected by properly specifying the pin location and
   I/O Standard.
WARNING:PhysDesignRules:2452 - The IOB QX<18> is either not constrained (LOC) to
   a specific location and/or has an undefined I/O Standard (IOSTANDARD). This
   condition may seriously affect the device and will be an error in bitstream
   creation. It should be corrected by properly specifying the pin location and
   I/O Standard.
WARNING:PhysDesignRules:2452 - The IOB QX<19> is either not constrained (LOC) to
   a specific location and/or has an undefined I/O Standard (IOSTANDARD). This
   condition may seriously affect the device and will be an error in bitstream
   creation. It should be corrected by properly specifying the pin location and
   I/O Standard.
WARNING:PhysDesignRules:2452 - The IOB QX<20> is either not constrained (LOC) to
   a specific location and/or has an undefined I/O Standard (IOSTANDARD). This
   condition may seriously affect the device and will be an error in bitstream
   creation. It should be corrected by properly specifying the pin location and
   I/O Standard.
WARNING:PhysDesignRules:2452 - The IOB QX<21> is either not constrained (LOC) to
   a specific location and/or has an undefined I/O Standard (IOSTANDARD). This
   condition may seriously affect the device and will be an error in bitstream
   creation. It should be corrected by properly specifying the pin location and
   I/O Standard.
WARNING:PhysDesignRules:2452 - The IOB QX<22> is either not constrained (LOC) to
   a specific location and/or has an undefined I/O Standard (IOSTANDARD). This
   condition may seriously affect the device and will be an error in bitstream
   creation. It should be corrected by properly specifying the pin location and
   I/O Standard.
WARNING:PhysDesignRules:2452 - The IOB QX<23> is either not constrained (LOC) to
   a specific location and/or has an undefined I/O Standard (IOSTANDARD). This
   condition may seriously affect the device and will be an error in bitstream
   creation. It should be corrected by properly specifying the pin location and
   I/O Standard.
WARNING:PhysDesignRules:2452 - The IOB QX<24> is either not constrained (LOC) to
   a specific location and/or has an undefined I/O Standard (IOSTANDARD). This
   condition may seriously affect the device and will be an error in bitstream
   creation. It should be corrected by properly specifying the pin location and
   I/O Standard.
WARNING:PhysDesignRules:2452 - The IOB QX<25> is either not constrained (LOC) to
   a specific location and/or has an undefined I/O Standard (IOSTANDARD). This
   condition may seriously affect the device and will be an error in bitstream
   creation. It should be corrected by properly specifying the pin location and
   I/O Standard.
WARNING:PhysDesignRules:2452 - The IOB QX<26> is either not constrained (LOC) to
   a specific location and/or has an undefined I/O Standard (IOSTANDARD). This
   condition may seriously affect the device and will be an error in bitstream
   creation. It should be corrected by properly specifying the pin location and
   I/O Standard.
WARNING:PhysDesignRules:2452 - The IOB QX<27> is either not constrained (LOC) to
   a specific location and/or has an undefined I/O Standard (IOSTANDARD). This
   condition may seriously affect the device and will be an error in bitstream
   creation. It should be corrected by properly specifying the pin location and
   I/O Standard.
WARNING:PhysDesignRules:2452 - The IOB QX<28> is either not constrained (LOC) to
   a specific location and/or has an undefined I/O Standard (IOSTANDARD). This
   condition may seriously affect the device and will be an error in bitstream
   creation. It should be corrected by properly specifying the pin location and
   I/O Standard.
WARNING:PhysDesignRules:2452 - The IOB QX<29> is either not constrained (LOC) to
   a specific location and/or has an undefined I/O Standard (IOSTANDARD). This
   condition may seriously affect the device and will be an error in bitstream
   creation. It should be corrected by properly specifying the pin location and
   I/O Standard.
WARNING:PhysDesignRules:2452 - The IOB QX<30> is either not constrained (LOC) to
   a specific location and/or has an undefined I/O Standard (IOSTANDARD). This
   condition may seriously affect the device and will be an error in bitstream
   creation. It should be corrected by properly specifying the pin location and
   I/O Standard.
```

```
WARNING:PhysDesignRules:2452 - The IOB QX<31> is either not constrained (LOC) to
   a specific location and/or has an undefined I/O Standard (IOSTANDARD). This
   condition may seriously affect the device and will be an error in bitstream
   creation. It should be corrected by properly specifying the pin location and
   I/O Standard.
WARNING:PhysDesignRules:2452 - The IOB QX<32> is either not constrained (LOC) to
   a specific location and/or has an undefined I/O Standard (IOSTANDARD). This
   condition may seriously affect the device and will be an error in bitstream
   creation. It should be corrected by properly specifying the pin location and
   I/O Standard.
WARNING:PhysDesignRules:2452 - The IOB QX<33> is either not constrained (LOC) to
   a specific location and/or has an undefined I/O Standard (IOSTANDARD). This
   condition may seriously affect the device and will be an error in bitstream
   creation. It should be corrected by properly specifying the pin location and
   I/O Standard.
WARNING:PhysDesignRules:2452 - The IOB QX<34> is either not constrained (LOC) to
   a specific location and/or has an undefined I/O Standard (IOSTANDARD). This
   condition may seriously affect the device and will be an error in bitstream
   creation. It should be corrected by properly specifying the pin location and
   I/O Standard.
WARNING:PhysDesignRules:2452 - The IOB QX<35> is either not constrained (LOC) to
   a specific location and/or has an undefined I/O Standard (IOSTANDARD). This
   condition may seriously affect the device and will be an error in bitstream
   creation. It should be corrected by properly specifying the pin location and
   I/O Standard.
WARNING:PhysDesignRules:2452 - The IOB QX<36> is either not constrained (LOC) to
   a specific location and/or has an undefined I/O Standard (IOSTANDARD). This
   condition may seriously affect the device and will be an error in bitstream
   creation. It should be corrected by properly specifying the pin location and
   I/O Standard.
WARNING:PhysDesignRules:2452 - The IOB QX<37> is either not constrained (LOC) to
   a specific location and/or has an undefined I/O Standard (IOSTANDARD). This
   condition may seriously affect the device and will be an error in bitstream
   creation. It should be corrected by properly specifying the pin location and
   I/O Standard.
WARNING:PhysDesignRules:2452 - The IOB QX<38> is either not constrained (LOC) to
   a specific location and/or has an undefined I/O Standard (IOSTANDARD). This
   condition may seriously affect the device and will be an error in bitstream
   creation. It should be corrected by properly specifying the pin location and
   I/O Standard.
WARNING:PhysDesignRules:2452 - The IOB QX<39> is either not constrained (LOC) to
   a specific location and/or has an undefined I/O Standard (IOSTANDARD). This
   condition may seriously affect the device and will be an error in bitstream
   creation. It should be corrected by properly specifying the pin location and
   I/O Standard.
WARNING:PhysDesignRules:2452 - The IOB QX<40> is either not constrained (LOC) to
   a specific location and/or has an undefined I/O Standard (IOSTANDARD). This
   condition may seriously affect the device and will be an error in bitstream
   creation. It should be corrected by properly specifying the pin location and
   I/O Standard.
WARNING:PhysDesignRules:2452 - The IOB QX<41> is either not constrained (LOC) to
   a specific location and/or has an undefined I/O Standard (IOSTANDARD). This
   condition may seriously affect the device and will be an error in bitstream
   creation. It should be corrected by properly specifying the pin location and
   I/O Standard.
WARNING:PhysDesignRules:2452 - The IOB QX<42> is either not constrained (LOC) to
   a specific location and/or has an undefined I/O Standard (IOSTANDARD). This
   condition may seriously affect the device and will be an error in bitstream
   creation. It should be corrected by properly specifying the pin location and
   I/O Standard.
WARNING:PhysDesignRules:2452 - The IOB QX<43> is either not constrained (LOC) to
   a specific location and/or has an undefined I/O Standard (IOSTANDARD). This
   condition may seriously affect the device and will be an error in bitstream
   creation. It should be corrected by properly specifying the pin location and
   I/O Standard.
WARNING:PhysDesignRules:2452 - The IOB QX<44> is either not constrained (LOC) to
   a specific location and/or has an undefined I/O Standard (IOSTANDARD). This
   condition may seriously affect the device and will be an error in bitstream
   creation. It should be corrected by properly specifying the pin location and
   I/O Standard.
WARNING:PhysDesignRules:2452 - The IOB QX<45> is either not constrained (LOC) to
```

```
   a specific location and/or has an undefined I/O Standard (IOSTANDARD). This
   condition may seriously affect the device and will be an error in bitstream
   creation. It should be corrected by properly specifying the pin location and
   I/O Standard.
WARNING:PhysDesignRules:2452 - The IOB QX<46> is either not constrained (LOC) to
   a specific location and/or has an undefined I/O Standard (IOSTANDARD). This
   condition may seriously affect the device and will be an error in bitstream
   creation. It should be corrected by properly specifying the pin location and
   I/O Standard.
WARNING:PhysDesignRules:2452 - The IOB QX<47> is either not constrained (LOC) to
   a specific location and/or has an undefined I/O Standard (IOSTANDARD). This
   condition may seriously affect the device and will be an error in bitstream
   creation. It should be corrected by properly specifying the pin location and
   I/O Standard.
WARNING:PhysDesignRules:2452 - The IOB QX<48> is either not constrained (LOC) to
   a specific location and/or has an undefined I/O Standard (IOSTANDARD). This
   condition may seriously affect the device and will be an error in bitstream
   creation. It should be corrected by properly specifying the pin location and
   I/O Standard.
WARNING:PhysDesignRules:2452 - The IOB QX<49> is either not constrained (LOC) to
   a specific location and/or has an undefined I/O Standard (IOSTANDARD). This
   condition may seriously affect the device and will be an error in bitstream
   creation. It should be corrected by properly specifying the pin location and
   I/O Standard.
WARNING:PhysDesignRules:2452 - The IOB QX<50> is either not constrained (LOC) to
   a specific location and/or has an undefined I/O Standard (IOSTANDARD). This
   condition may seriously affect the device and will be an error in bitstream
   creation. It should be corrected by properly specifying the pin location and
   I/O Standard.
WARNING:PhysDesignRules:2452 - The IOB QX<51> is either not constrained (LOC) to
   a specific location and/or has an undefined I/O Standard (IOSTANDARD). This
   condition may seriously affect the device and will be an error in bitstream
   creation. It should be corrected by properly specifying the pin location and
   I/O Standard.
WARNING:PhysDesignRules:2452 - The IOB QX<52> is either not constrained (LOC) to
   a specific location and/or has an undefined I/O Standard (IOSTANDARD). This
   condition may seriously affect the device and will be an error in bitstream
   creation. It should be corrected by properly specifying the pin location and
   I/O Standard.
WARNING:PhysDesignRules:2452 - The IOB QX<53> is either not constrained (LOC) to
   a specific location and/or has an undefined I/O Standard (IOSTANDARD). This
   condition may seriously affect the device and will be an error in bitstream
   creation. It should be corrected by properly specifying the pin location and
   I/O Standard.
WARNING:PhysDesignRules:2452 - The IOB QX<54> is either not constrained (LOC) to
   a specific location and/or has an undefined I/O Standard (IOSTANDARD). This
   condition may seriously affect the device and will be an error in bitstream
   creation. It should be corrected by properly specifying the pin location and
   I/O Standard.
WARNING:PhysDesignRules:2452 - The IOB QX<55> is either not constrained (LOC) to
   a specific location and/or has an undefined I/O Standard (IOSTANDARD). This
   condition may seriously affect the device and will be an error in bitstream
   creation. It should be corrected by properly specifying the pin location and
   I/O Standard.
WARNING:PhysDesignRules:2452 - The IOB QX<56> is either not constrained (LOC) to
   a specific location and/or has an undefined I/O Standard (IOSTANDARD). This
   condition may seriously affect the device and will be an error in bitstream
   creation. It should be corrected by properly specifying the pin location and
   I/O Standard.
WARNING:PhysDesignRules:2452 - The IOB QX<57> is either not constrained (LOC) to
   a specific location and/or has an undefined I/O Standard (IOSTANDARD). This
   condition may seriously affect the device and will be an error in bitstream
   creation. It should be corrected by properly specifying the pin location and
   I/O Standard.
WARNING:PhysDesignRules:2452 - The IOB QX<58> is either not constrained (LOC) to
   a specific location and/or has an undefined I/O Standard (IOSTANDARD). This
   condition may seriously affect the device and will be an error in bitstream
   creation. It should be corrected by properly specifying the pin location and
   I/O Standard.
WARNING:PhysDesignRules:2452 - The IOB QX<59> is either not constrained (LOC) to
   a specific location and/or has an undefined I/O Standard (IOSTANDARD). This
```

```
   condition may seriously affect the device and will be an error in bitstream
   creation. It should be corrected by properly specifying the pin location and
   I/O Standard.
WARNING:PhysDesignRules:2452 - The IOB QX<60> is either not constrained (LOC) to
   a specific location and/or has an undefined I/O Standard (IOSTANDARD). This
   condition may seriously affect the device and will be an error in bitstream
   creation. It should be corrected by properly specifying the pin location and
   I/O Standard.
WARNING:PhysDesignRules:2452 - The IOB QX<61> is either not constrained (LOC) to
   a specific location and/or has an undefined I/O Standard (IOSTANDARD). This
   condition may seriously affect the device and will be an error in bitstream
   creation. It should be corrected by properly specifying the pin location and
   I/O Standard.
WARNING:PhysDesignRules:2452 - The IOB QX<62> is either not constrained (LOC) to
   a specific location and/or has an undefined I/O Standard (IOSTANDARD). This
   condition may seriously affect the device and will be an error in bitstream
   creation. It should be corrected by properly specifying the pin location and
   I/O Standard.
WARNING:PhysDesignRules:2452 - The IOB QX<63> is either not constrained (LOC) to
   a specific location and/or has an undefined I/O Standard (IOSTANDARD). This
   condition may seriously affect the device and will be an error in bitstream
   creation. It should be corrected by properly specifying the pin location and
   I/O Standard.
WARNING:PhysDesignRules:2452 - The IOB QX<64> is either not constrained (LOC) to
   a specific location and/or has an undefined I/O Standard (IOSTANDARD). This
   condition may seriously affect the device and will be an error in bitstream
   creation. It should be corrected by properly specifying the pin location and
   I/O Standard.
WARNING:PhysDesignRules:2452 - The IOB QX<65> is either not constrained (LOC) to
   a specific location and/or has an undefined I/O Standard (IOSTANDARD). This
   condition may seriously affect the device and will be an error in bitstream
   creation. It should be corrected by properly specifying the pin location and
   I/O Standard.
WARNING:PhysDesignRules:2452 - The IOB QX<66> is either not constrained (LOC) to
   a specific location and/or has an undefined I/O Standard (IOSTANDARD). This
   condition may seriously affect the device and will be an error in bitstream
   creation. It should be corrected by properly specifying the pin location and
   I/O Standard.
WARNING:PhysDesignRules:2452 - The IOB QX<67> is either not constrained (LOC) to
   a specific location and/or has an undefined I/O Standard (IOSTANDARD). This
   condition may seriously affect the device and will be an error in bitstream
   creation. It should be corrected by properly specifying the pin location and
   I/O Standard.
WARNING:PhysDesignRules:2452 - The IOB QX<68> is either not constrained (LOC) to
   a specific location and/or has an undefined I/O Standard (IOSTANDARD). This
   condition may seriously affect the device and will be an error in bitstream
   creation. It should be corrected by properly specifying the pin location and
   I/O Standard.
WARNING:PhysDesignRules:2452 - The IOB QX<69> is either not constrained (LOC) to
   a specific location and/or has an undefined I/O Standard (IOSTANDARD). This
   condition may seriously affect the device and will be an error in bitstream
   creation. It should be corrected by properly specifying the pin location and
   I/O Standard.
WARNING:PhysDesignRules:2452 - The IOB QX<70> is either not constrained (LOC) to
   a specific location and/or has an undefined I/O Standard (IOSTANDARD). This
   condition may seriously affect the device and will be an error in bitstream
   creation. It should be corrected by properly specifying the pin location and
   I/O Standard.
WARNING:PhysDesignRules:2452 - The IOB QX<71> is either not constrained (LOC) to
   a specific location and/or has an undefined I/O Standard (IOSTANDARD). This
   condition may seriously affect the device and will be an error in bitstream
   creation. It should be corrected by properly specifying the pin location and
   I/O Standard.
WARNING:PhysDesignRules:2452 - The IOB QX<72> is either not constrained (LOC) to
   a specific location and/or has an undefined I/O Standard (IOSTANDARD). This
   condition may seriously affect the device and will be an error in bitstream
   creation. It should be corrected by properly specifying the pin location and
   I/O Standard.
WARNING:PhysDesignRules:2452 - The IOB QX<73> is either not constrained (LOC) to
   a specific location and/or has an undefined I/O Standard (IOSTANDARD). This
   condition may seriously affect the device and will be an error in bitstream
```

```
   creation. It should be corrected by properly specifying the pin location and
   I/O Standard.
WARNING:PhysDesignRules:2452 - The IOB QX<74> is either not constrained (LOC) to
   a specific location and/or has an undefined I/O Standard (IOSTANDARD). This
   condition may seriously affect the device and will be an error in bitstream
   creation. It should be corrected by properly specifying the pin location and
   I/O Standard.
WARNING:PhysDesignRules:2452 - The IOB QX<75> is either not constrained (LOC) to
   a specific location and/or has an undefined I/O Standard (IOSTANDARD). This
   condition may seriously affect the device and will be an error in bitstream
   creation. It should be corrected by properly specifying the pin location and
   I/O Standard.
WARNING:PhysDesignRules:2452 - The IOB QX<76> is either not constrained (LOC) to
   a specific location and/or has an undefined I/O Standard (IOSTANDARD). This
   condition may seriously affect the device and will be an error in bitstream
   creation. It should be corrected by properly specifying the pin location and
   I/O Standard.
WARNING:PhysDesignRules:2452 - The IOB QX<77> is either not constrained (LOC) to
   a specific location and/or has an undefined I/O Standard (IOSTANDARD). This
   condition may seriously affect the device and will be an error in bitstream
   creation. It should be corrected by properly specifying the pin location and
   I/O Standard.
WARNING:PhysDesignRules:2452 - The IOB QX<78> is either not constrained (LOC) to
   a specific location and/or has an undefined I/O Standard (IOSTANDARD). This
   condition may seriously affect the device and will be an error in bitstream
   creation. It should be corrected by properly specifying the pin location and
   I/O Standard.
WARNING:PhysDesignRules:2452 - The IOB QX<79> is either not constrained (LOC) to
   a specific location and/or has an undefined I/O Standard (IOSTANDARD). This
   condition may seriously affect the device and will be an error in bitstream
   creation. It should be corrected by properly specifying the pin location and
   I/O Standard.
WARNING:PhysDesignRules:2452 - The IOB QX<80> is either not constrained (LOC) to
   a specific location and/or has an undefined I/O Standard (IOSTANDARD). This
   condition may seriously affect the device and will be an error in bitstream
   creation. It should be corrected by properly specifying the pin location and
   I/O Standard.
WARNING:PhysDesignRules:2452 - The IOB QX<81> is either not constrained (LOC) to
   a specific location and/or has an undefined I/O Standard (IOSTANDARD). This
   condition may seriously affect the device and will be an error in bitstream
   creation. It should be corrected by properly specifying the pin location and
   I/O Standard.
WARNING:PhysDesignRules:2452 - The IOB QX<82> is either not constrained (LOC) to
   a specific location and/or has an undefined I/O Standard (IOSTANDARD). This
   condition may seriously affect the device and will be an error in bitstream
   creation. It should be corrected by properly specifying the pin location and
   I/O Standard.
WARNING:PhysDesignRules:2452 - The IOB QX<83> is either not constrained (LOC) to
   a specific location and/or has an undefined I/O Standard (IOSTANDARD). This
   condition may seriously affect the device and will be an error in bitstream
   creation. It should be corrected by properly specifying the pin location and
   I/O Standard.
WARNING:PhysDesignRules:2452 - The IOB QX<84> is either not constrained (LOC) to
   a specific location and/or has an undefined I/O Standard (IOSTANDARD). This
   condition may seriously affect the device and will be an error in bitstream
   creation. It should be corrected by properly specifying the pin location and
   I/O Standard.
WARNING:PhysDesignRules:2452 - The IOB QX<85> is either not constrained (LOC) to
   a specific location and/or has an undefined I/O Standard (IOSTANDARD). This
   condition may seriously affect the device and will be an error in bitstream
   creation. It should be corrected by properly specifying the pin location and
   I/O Standard.
WARNING:PhysDesignRules:2452 - The IOB QX<86> is either not constrained (LOC) to
   a specific location and/or has an undefined I/O Standard (IOSTANDARD). This
   condition may seriously affect the device and will be an error in bitstream
   creation. It should be corrected by properly specifying the pin location and
   I/O Standard.
WARNING:PhysDesignRules:2452 - The IOB QX<87> is either not constrained (LOC) to
   a specific location and/or has an undefined I/O Standard (IOSTANDARD). This
   condition may seriously affect the device and will be an error in bitstream
   creation. It should be corrected by properly specifying the pin location and
```

```
   I/O Standard.
WARNING:PhysDesignRules:2452 - The IOB QX<88> is either not constrained (LOC) to
   a specific location and/or has an undefined I/O Standard (IOSTANDARD). This
   condition may seriously affect the device and will be an error in bitstream
   creation. It should be corrected by properly specifying the pin location and
   I/O Standard.
WARNING:PhysDesignRules:2452 - The IOB QX<89> is either not constrained (LOC) to
   a specific location and/or has an undefined I/O Standard (IOSTANDARD). This
   condition may seriously affect the device and will be an error in bitstream
   creation. It should be corrected by properly specifying the pin location and
   I/O Standard.
WARNING:PhysDesignRules:2452 - The IOB QX<90> is either not constrained (LOC) to
   a specific location and/or has an undefined I/O Standard (IOSTANDARD). This
   condition may seriously affect the device and will be an error in bitstream
   creation. It should be corrected by properly specifying the pin location and
   I/O Standard.
WARNING:PhysDesignRules:2452 - The IOB QX<91> is either not constrained (LOC) to
   a specific location and/or has an undefined I/O Standard (IOSTANDARD). This
   condition may seriously affect the device and will be an error in bitstream
   creation. It should be corrected by properly specifying the pin location and
   I/O Standard.
WARNING:PhysDesignRules:2452 - The IOB QX<92> is either not constrained (LOC) to
   a specific location and/or has an undefined I/O Standard (IOSTANDARD). This
   condition may seriously affect the device and will be an error in bitstream
   creation. It should be corrected by properly specifying the pin location and
   I/O Standard.
WARNING:PhysDesignRules:2452 - The IOB QX<93> is either not constrained (LOC) to
   a specific location and/or has an undefined I/O Standard (IOSTANDARD). This
   condition may seriously affect the device and will be an error in bitstream
   creation. It should be corrected by properly specifying the pin location and
   I/O Standard.
WARNING:PhysDesignRules:2452 - The IOB QX<94> is either not constrained (LOC) to
   a specific location and/or has an undefined I/O Standard (IOSTANDARD). This
   condition may seriously affect the device and will be an error in bitstream
   creation. It should be corrected by properly specifying the pin location and
   I/O Standard.
WARNING:PhysDesignRules:2452 - The IOB QX<95> is either not constrained (LOC) to
   a specific location and/or has an undefined I/O Standard (IOSTANDARD). This
   condition may seriously affect the device and will be an error in bitstream
   creation. It should be corrected by properly specifying the pin location and
   I/O Standard.
WARNING:PhysDesignRules:2452 - The IOB QX<96> is either not constrained (LOC) to
   a specific location and/or has an undefined I/O Standard (IOSTANDARD). This
   condition may seriously affect the device and will be an error in bitstream
   creation. It should be corrected by properly specifying the pin location and
   I/O Standard.
WARNING:PhysDesignRules:2452 - The IOB QX<97> is either not constrained (LOC) to
   a specific location and/or has an undefined I/O Standard (IOSTANDARD). This
   condition may seriously affect the device and will be an error in bitstream
   creation. It should be corrected by properly specifying the pin location and
   I/O Standard.
WARNING:PhysDesignRules:2452 - The IOB QX<98> is either not constrained (LOC) to
   a specific location and/or has an undefined I/O Standard (IOSTANDARD). This
   condition may seriously affect the device and will be an error in bitstream
   creation. It should be corrected by properly specifying the pin location and
   I/O Standard.
WARNING:PhysDesignRules:2452 - The IOB QX<99> is either not constrained (LOC) to
   a specific location and/or has an undefined I/O Standard (IOSTANDARD). This
   condition may seriously affect the device and will be an error in bitstream
   creation. It should be corrected by properly specifying the pin location and
   I/O Standard.
WARNING:PhysDesignRules:2452 - The IOB QX<0> is either not constrained (LOC) to
   a specific location and/or has an undefined I/O Standard (IOSTANDARD). This
   condition may seriously affect the device and will be an error in bitstream
   creation. It should be corrected by properly specifying the pin location and
   I/O Standard.
WARNING:PhysDesignRules:2452 - The IOB QX<1> is either not constrained (LOC) to
   a specific location and/or has an undefined I/O Standard (IOSTANDARD). This
   condition may seriously affect the device and will be an error in bitstream
   creation. It should be corrected by properly specifying the pin location and
   I/O Standard.
```

```
WARNING:PhysDesignRules:2452 - The IOB QX<2> is either not constrained (LOC) to
   a specific location and/or has an undefined I/O Standard (IOSTANDARD). This
   condition may seriously affect the device and will be an error in bitstream
   creation. It should be corrected by properly specifying the pin location and
   I/O Standard.
WARNING:PhysDesignRules:2452 - The IOB QX<3> is either not constrained (LOC) to
   a specific location and/or has an undefined I/O Standard (IOSTANDARD). This
   condition may seriously affect the device and will be an error in bitstream
   creation. It should be corrected by properly specifying the pin location and
   I/O Standard.
WARNING:PhysDesignRules:2452 - The IOB QX<4> is either not constrained (LOC) to
   a specific location and/or has an undefined I/O Standard (IOSTANDARD). This
   condition may seriously affect the device and will be an error in bitstream
   creation. It should be corrected by properly specifying the pin location and
   I/O Standard.
WARNING:PhysDesignRules:2452 - The IOB QX<5> is either not constrained (LOC) to
   a specific location and/or has an undefined I/O Standard (IOSTANDARD). This
   condition may seriously affect the device and will be an error in bitstream
   creation. It should be corrected by properly specifying the pin location and
   I/O Standard.
WARNING:PhysDesignRules:2452 - The IOB QX<6> is either not constrained (LOC) to
   a specific location and/or has an undefined I/O Standard (IOSTANDARD). This
   condition may seriously affect the device and will be an error in bitstream
   creation. It should be corrected by properly specifying the pin location and
   I/O Standard.
WARNING:PhysDesignRules:2452 - The IOB QX<7> is either not constrained (LOC) to
   a specific location and/or has an undefined I/O Standard (IOSTANDARD). This
   condition may seriously affect the device and will be an error in bitstream
   creation. It should be corrected by properly specifying the pin location and
   I/O Standard.
WARNING:PhysDesignRules:2452 - The IOB QX<8> is either not constrained (LOC) to
   a specific location and/or has an undefined I/O Standard (IOSTANDARD). This
   condition may seriously affect the device and will be an error in bitstream
   creation. It should be corrected by properly specifying the pin location and
   I/O Standard.
WARNING:PhysDesignRules:2452 - The IOB QX<9> is either not constrained (LOC) to
   a specific location and/or has an undefined I/O Standard (IOSTANDARD). This
   condition may seriously affect the device and will be an error in bitstream
   creation. It should be corrected by properly specifying the pin location and
   I/O Standard.
WARNING:PhysDesignRules:2452 - The IOB QY<0> is either not constrained (LOC) to
   a specific location and/or has an undefined I/O Standard (IOSTANDARD). This
   condition may seriously affect the device and will be an error in bitstream
   creation. It should be corrected by properly specifying the pin location and
   I/O Standard.
WARNING:PhysDesignRules:2452 - The IOB QY<1> is either not constrained (LOC) to
   a specific location and/or has an undefined I/O Standard (IOSTANDARD). This
   condition may seriously affect the device and will be an error in bitstream
   creation. It should be corrected by properly specifying the pin location and
   I/O Standard.
WARNING:PhysDesignRules:2452 - The IOB QY<2> is either not constrained (LOC) to
   a specific location and/or has an undefined I/O Standard (IOSTANDARD). This
   condition may seriously affect the device and will be an error in bitstream
   creation. It should be corrected by properly specifying the pin location and
   I/O Standard.
WARNING:PhysDesignRules:2452 - The IOB QY<3> is either not constrained (LOC) to
   a specific location and/or has an undefined I/O Standard (IOSTANDARD). This
   condition may seriously affect the device and will be an error in bitstream
   creation. It should be corrected by properly specifying the pin location and
   I/O Standard.
WARNING:PhysDesignRules:2452 - The IOB QY<4> is either not constrained (LOC) to
   a specific location and/or has an undefined I/O Standard (IOSTANDARD). This
   condition may seriously affect the device and will be an error in bitstream
   creation. It should be corrected by properly specifying the pin location and
   I/O Standard.
WARNING:PhysDesignRules:2452 - The IOB QY<5> is either not constrained (LOC) to
   a specific location and/or has an undefined I/O Standard (IOSTANDARD). This
   condition may seriously affect the device and will be an error in bitstream
   creation. It should be corrected by properly specifying the pin location and
   I/O Standard.
WARNING:PhysDesignRules:2452 - The IOB QY<6> is either not constrained (LOC) to
```

```
   a specific location and/or has an undefined I/O Standard (IOSTANDARD). This
   condition may seriously affect the device and will be an error in bitstream
   creation. It should be corrected by properly specifying the pin location and
   I/O Standard.
WARNING:PhysDesignRules:2452 - The IOB QY<7> is either not constrained (LOC) to
   a specific location and/or has an undefined I/O Standard (IOSTANDARD). This
   condition may seriously affect the device and will be an error in bitstream
   creation. It should be corrected by properly specifying the pin location and
   I/O Standard.
WARNING:PhysDesignRules:2452 - The IOB QY<8> is either not constrained (LOC) to
   a specific location and/or has an undefined I/O Standard (IOSTANDARD). This
   condition may seriously affect the device and will be an error in bitstream
   creation. It should be corrected by properly specifying the pin location and
   I/O Standard.
WARNING:PhysDesignRules:2452 - The IOB QY<9> is either not constrained (LOC) to
   a specific location and/or has an undefined I/O Standard (IOSTANDARD). This
   condition may seriously affect the device and will be an error in bitstream
   creation. It should be corrected by properly specifying the pin location and
   I/O Standard.
WARNING:PhysDesignRules:2452 - The IOB QZ<10> is either not constrained (LOC) to
   a specific location and/or has an undefined I/O Standard (IOSTANDARD). This
   condition may seriously affect the device and will be an error in bitstream
   creation. It should be corrected by properly specifying the pin location and
   I/O Standard.
WARNING:PhysDesignRules:2452 - The IOB QZ<11> is either not constrained (LOC) to
   a specific location and/or has an undefined I/O Standard (IOSTANDARD). This
   condition may seriously affect the device and will be an error in bitstream
   creation. It should be corrected by properly specifying the pin location and
   I/O Standard.
WARNING:PhysDesignRules:2452 - The IOB QZ<12> is either not constrained (LOC) to
   a specific location and/or has an undefined I/O Standard (IOSTANDARD). This
   condition may seriously affect the device and will be an error in bitstream
   creation. It should be corrected by properly specifying the pin location and
   I/O Standard.
WARNING:PhysDesignRules:2452 - The IOB QZ<13> is either not constrained (LOC) to
   a specific location and/or has an undefined I/O Standard (IOSTANDARD). This
   condition may seriously affect the device and will be an error in bitstream
   creation. It should be corrected by properly specifying the pin location and
   I/O Standard.
WARNING:PhysDesignRules:2452 - The IOB QZ<14> is either not constrained (LOC) to
   a specific location and/or has an undefined I/O Standard (IOSTANDARD). This
   condition may seriously affect the device and will be an error in bitstream
   creation. It should be corrected by properly specifying the pin location and
   I/O Standard.
WARNING:PhysDesignRules:2452 - The IOB QZ<15> is either not constrained (LOC) to
   a specific location and/or has an undefined I/O Standard (IOSTANDARD). This
   condition may seriously affect the device and will be an error in bitstream
   creation. It should be corrected by properly specifying the pin location and
   I/O Standard.
WARNING:PhysDesignRules:2452 - The IOB QZ<16> is either not constrained (LOC) to
   a specific location and/or has an undefined I/O Standard (IOSTANDARD). This
   condition may seriously affect the device and will be an error in bitstream
   creation. It should be corrected by properly specifying the pin location and
   I/O Standard.
WARNING:PhysDesignRules:2452 - The IOB QZ<17> is either not constrained (LOC) to
   a specific location and/or has an undefined I/O Standard (IOSTANDARD). This
   condition may seriously affect the device and will be an error in bitstream
   creation. It should be corrected by properly specifying the pin location and
   I/O Standard.
WARNING:PhysDesignRules:2452 - The IOB QZ<18> is either not constrained (LOC) to
   a specific location and/or has an undefined I/O Standard (IOSTANDARD). This
   condition may seriously affect the device and will be an error in bitstream
   creation. It should be corrected by properly specifying the pin location and
   I/O Standard.
WARNING:PhysDesignRules:2452 - The IOB QZ<19> is either not constrained (LOC) to
   a specific location and/or has an undefined I/O Standard (IOSTANDARD). This
   condition may seriously affect the device and will be an error in bitstream
   creation. It should be corrected by properly specifying the pin location and
   I/O Standard.
WARNING:PhysDesignRules:2452 - The IOB QZ<20> is either not constrained (LOC) to
   a specific location and/or has an undefined I/O Standard (IOSTANDARD). This
```

```
   condition may seriously affect the device and will be an error in bitstream
   creation. It should be corrected by properly specifying the pin location and
   I/O Standard.
WARNING:PhysDesignRules:2452 - The IOB QZ<21> is either not constrained (LOC) to
   a specific location and/or has an undefined I/O Standard (IOSTANDARD). This
   condition may seriously affect the device and will be an error in bitstream
   creation. It should be corrected by properly specifying the pin location and
   I/O Standard.
WARNING:PhysDesignRules:2452 - The IOB QZ<22> is either not constrained (LOC) to
   a specific location and/or has an undefined I/O Standard (IOSTANDARD). This
   condition may seriously affect the device and will be an error in bitstream
   creation. It should be corrected by properly specifying the pin location and
   I/O Standard.
WARNING:PhysDesignRules:2452 - The IOB QZ<23> is either not constrained (LOC) to
   a specific location and/or has an undefined I/O Standard (IOSTANDARD). This
   condition may seriously affect the device and will be an error in bitstream
   creation. It should be corrected by properly specifying the pin location and
   I/O Standard.
WARNING:PhysDesignRules:2452 - The IOB QZ<24> is either not constrained (LOC) to
   a specific location and/or has an undefined I/O Standard (IOSTANDARD). This
   condition may seriously affect the device and will be an error in bitstream
   creation. It should be corrected by properly specifying the pin location and
   I/O Standard.
WARNING:PhysDesignRules:2452 - The IOB QZ<25> is either not constrained (LOC) to
   a specific location and/or has an undefined I/O Standard (IOSTANDARD). This
   condition may seriously affect the device and will be an error in bitstream
   creation. It should be corrected by properly specifying the pin location and
   I/O Standard.
WARNING:PhysDesignRules:2452 - The IOB QZ<26> is either not constrained (LOC) to
   a specific location and/or has an undefined I/O Standard (IOSTANDARD). This
   condition may seriously affect the device and will be an error in bitstream
   creation. It should be corrected by properly specifying the pin location and
   I/O Standard.
WARNING:PhysDesignRules:2452 - The IOB QZ<27> is either not constrained (LOC) to
   a specific location and/or has an undefined I/O Standard (IOSTANDARD). This
   condition may seriously affect the device and will be an error in bitstream
   creation. It should be corrected by properly specifying the pin location and
   I/O Standard.
WARNING:PhysDesignRules:2452 - The IOB QZ<28> is either not constrained (LOC) to
   a specific location and/or has an undefined I/O Standard (IOSTANDARD). This
   condition may seriously affect the device and will be an error in bitstream
   creation. It should be corrected by properly specifying the pin location and
   I/O Standard.
WARNING:PhysDesignRules:2452 - The IOB QZ<29> is either not constrained (LOC) to
   a specific location and/or has an undefined I/O Standard (IOSTANDARD). This
   condition may seriously affect the device and will be an error in bitstream
   creation. It should be corrected by properly specifying the pin location and
   I/O Standard.
WARNING:PhysDesignRules:2452 - The IOB QZ<30> is either not constrained (LOC) to
   a specific location and/or has an undefined I/O Standard (IOSTANDARD). This
   condition may seriously affect the device and will be an error in bitstream
   creation. It should be corrected by properly specifying the pin location and
   I/O Standard.
WARNING:PhysDesignRules:2452 - The IOB QZ<31> is either not constrained (LOC) to
   a specific location and/or has an undefined I/O Standard (IOSTANDARD). This
   condition may seriously affect the device and will be an error in bitstream
   creation. It should be corrected by properly specifying the pin location and
   I/O Standard.
WARNING:PhysDesignRules:2452 - The IOB QZ<32> is either not constrained (LOC) to
   a specific location and/or has an undefined I/O Standard (IOSTANDARD). This
   condition may seriously affect the device and will be an error in bitstream
   creation. It should be corrected by properly specifying the pin location and
   I/O Standard.
WARNING:PhysDesignRules:2452 - The IOB QZ<33> is either not constrained (LOC) to
   a specific location and/or has an undefined I/O Standard (IOSTANDARD). This
   condition may seriously affect the device and will be an error in bitstream
   creation. It should be corrected by properly specifying the pin location and
   I/O Standard.
WARNING:PhysDesignRules:2452 - The IOB QZ<34> is either not constrained (LOC) to
   a specific location and/or has an undefined I/O Standard (IOSTANDARD). This
   condition may seriously affect the device and will be an error in bitstream
```

```
   creation. It should be corrected by properly specifying the pin location and
   I/O Standard.
WARNING:PhysDesignRules:2452 - The IOB QZ<35> is either not constrained (LOC) to
   a specific location and/or has an undefined I/O Standard (IOSTANDARD). This
   condition may seriously affect the device and will be an error in bitstream
   creation. It should be corrected by properly specifying the pin location and
   I/O Standard.
WARNING:PhysDesignRules:2452 - The IOB QZ<36> is either not constrained (LOC) to
   a specific location and/or has an undefined I/O Standard (IOSTANDARD). This
   condition may seriously affect the device and will be an error in bitstream
   creation. It should be corrected by properly specifying the pin location and
   I/O Standard.
WARNING:PhysDesignRules:2452 - The IOB QZ<37> is either not constrained (LOC) to
   a specific location and/or has an undefined I/O Standard (IOSTANDARD). This
   condition may seriously affect the device and will be an error in bitstream
   creation. It should be corrected by properly specifying the pin location and
   I/O Standard.
WARNING:PhysDesignRules:2452 - The IOB QZ<38> is either not constrained (LOC) to
   a specific location and/or has an undefined I/O Standard (IOSTANDARD). This
   condition may seriously affect the device and will be an error in bitstream
   creation. It should be corrected by properly specifying the pin location and
   I/O Standard.
WARNING:PhysDesignRules:2452 - The IOB QZ<39> is either not constrained (LOC) to
   a specific location and/or has an undefined I/O Standard (IOSTANDARD). This
   condition may seriously affect the device and will be an error in bitstream
   creation. It should be corrected by properly specifying the pin location and
   I/O Standard.
WARNING:PhysDesignRules:2452 - The IOB QZ<40> is either not constrained (LOC) to
   a specific location and/or has an undefined I/O Standard (IOSTANDARD). This
   condition may seriously affect the device and will be an error in bitstream
   creation. It should be corrected by properly specifying the pin location and
   I/O Standard.
WARNING:PhysDesignRules:2452 - The IOB QZ<41> is either not constrained (LOC) to
   a specific location and/or has an undefined I/O Standard (IOSTANDARD). This
   condition may seriously affect the device and will be an error in bitstream
   creation. It should be corrected by properly specifying the pin location and
   I/O Standard.
WARNING:PhysDesignRules:2452 - The IOB QZ<42> is either not constrained (LOC) to
   a specific location and/or has an undefined I/O Standard (IOSTANDARD). This
   condition may seriously affect the device and will be an error in bitstream
   creation. It should be corrected by properly specifying the pin location and
   I/O Standard.
WARNING:PhysDesignRules:2452 - The IOB QZ<43> is either not constrained (LOC) to
   a specific location and/or has an undefined I/O Standard (IOSTANDARD). This
   condition may seriously affect the device and will be an error in bitstream
   creation. It should be corrected by properly specifying the pin location and
   I/O Standard.
WARNING:PhysDesignRules:2452 - The IOB QZ<44> is either not constrained (LOC) to
   a specific location and/or has an undefined I/O Standard (IOSTANDARD). This
   condition may seriously affect the device and will be an error in bitstream
   creation. It should be corrected by properly specifying the pin location and
   I/O Standard.
WARNING:PhysDesignRules:2452 - The IOB QZ<45> is either not constrained (LOC) to
   a specific location and/or has an undefined I/O Standard (IOSTANDARD). This
   condition may seriously affect the device and will be an error in bitstream
   creation. It should be corrected by properly specifying the pin location and
   I/O Standard.
WARNING:PhysDesignRules:2452 - The IOB QZ<46> is either not constrained (LOC) to
   a specific location and/or has an undefined I/O Standard (IOSTANDARD). This
   condition may seriously affect the device and will be an error in bitstream
   creation. It should be corrected by properly specifying the pin location and
   I/O Standard.
WARNING:PhysDesignRules:2452 - The IOB QZ<47> is either not constrained (LOC) to
   a specific location and/or has an undefined I/O Standard (IOSTANDARD). This
   condition may seriously affect the device and will be an error in bitstream
   creation. It should be corrected by properly specifying the pin location and
   I/O Standard.
WARNING:PhysDesignRules:2452 - The IOB QZ<48> is either not constrained (LOC) to
   a specific location and/or has an undefined I/O Standard (IOSTANDARD). This
   condition may seriously affect the device and will be an error in bitstream
   creation. It should be corrected by properly specifying the pin location and
```

I/O Standard.

WARNING:PhysDesignRules:2452 - The IOB QZ<49> is either not constrained (LOC) to a specific location and/or has an undefined I/O Standard (IOSTANDARD). This condition may seriously affect the device and will be an error in bitstream creation. It should be corrected by properly specifying the pin location and I/O Standard.

WARNING:PhysDesignRules:2452 - The IOB QZ<50> is either not constrained (LOC) to a specific location and/or has an undefined I/O Standard (IOSTANDARD). This condition may seriously affect the device and will be an error in bitstream creation. It should be corrected by properly specifying the pin location and I/O Standard.

WARNING:PhysDesignRules:2452 - The IOB QZ<51> is either not constrained (LOC) to a specific location and/or has an undefined I/O Standard (IOSTANDARD). This condition may seriously affect the device and will be an error in bitstream creation. It should be corrected by properly specifying the pin location and I/O Standard.

WARNING:PhysDesignRules:2452 - The IOB QZ<52> is either not constrained (LOC) to a specific location and/or has an undefined I/O Standard (IOSTANDARD). This condition may seriously affect the device and will be an error in bitstream creation. It should be corrected by properly specifying the pin location and I/O Standard.

WARNING:PhysDesignRules:2452 - The IOB QZ<53> is either not constrained (LOC) to a specific location and/or has an undefined I/O Standard (IOSTANDARD). This condition may seriously affect the device and will be an error in bitstream creation. It should be corrected by properly specifying the pin location and I/O Standard.

WARNING:PhysDesignRules:2452 - The IOB QZ<54> is either not constrained (LOC) to a specific location and/or has an undefined I/O Standard (IOSTANDARD). This condition may seriously affect the device and will be an error in bitstream creation. It should be corrected by properly specifying the pin location and I/O Standard.

WARNING:PhysDesignRules:2452 - The IOB QZ<55> is either not constrained (LOC) to a specific location and/or has an undefined I/O Standard (IOSTANDARD). This condition may seriously affect the device and will be an error in bitstream creation. It should be corrected by properly specifying the pin location and I/O Standard.

WARNING:PhysDesignRules:2452 - The IOB QZ<56> is either not constrained (LOC) to a specific location and/or has an undefined I/O Standard (IOSTANDARD). This condition may seriously affect the device and will be an error in bitstream creation. It should be corrected by properly specifying the pin location and I/O Standard.

WARNING:PhysDesignRules:2452 - The IOB QZ<57> is either not constrained (LOC) to a specific location and/or has an undefined I/O Standard (IOSTANDARD). This condition may seriously affect the device and will be an error in bitstream creation. It should be corrected by properly specifying the pin location and I/O Standard.

WARNING:PhysDesignRules:2452 - The IOB QZ<58> is either not constrained (LOC) to a specific location and/or has an undefined I/O Standard (IOSTANDARD). This condition may seriously affect the device and will be an error in bitstream creation. It should be corrected by properly specifying the pin location and I/O Standard.

WARNING:PhysDesignRules:2452 - The IOB QZ<59> is either not constrained (LOC) to a specific location and/or has an undefined I/O Standard (IOSTANDARD). This condition may seriously affect the device and will be an error in bitstream creation. It should be corrected by properly specifying the pin location and I/O Standard.

WARNING:PhysDesignRules:2452 - The IOB QZ<60> is either not constrained (LOC) to a specific location and/or has an undefined I/O Standard (IOSTANDARD). This condition may seriously affect the device and will be an error in bitstream creation. It should be corrected by properly specifying the pin location and I/O Standard.

WARNING:PhysDesignRules:2452 - The IOB QZ<61> is either not constrained (LOC) to a specific location and/or has an undefined I/O Standard (IOSTANDARD). This condition may seriously affect the device and will be an error in bitstream creation. It should be corrected by properly specifying the pin location and I/O Standard.

WARNING:PhysDesignRules:2452 - The IOB QZ<62> is either not constrained (LOC) to a specific location and/or has an undefined I/O Standard (IOSTANDARD). This condition may seriously affect the device and will be an error in bitstream creation. It should be corrected by properly specifying the pin location and I/O Standard.

```
WARNING:PhysDesignRules:2452 - The IOB QZ<63> is either not constrained (LOC) to
   a specific location and/or has an undefined I/O Standard (IOSTANDARD). This
   condition may seriously affect the device and will be an error in bitstream
   creation. It should be corrected by properly specifying the pin location and
   I/O Standard.
WARNING:PhysDesignRules:2452 - The IOB QZ<64> is either not constrained (LOC) to
   a specific location and/or has an undefined I/O Standard (IOSTANDARD). This
   condition may seriously affect the device and will be an error in bitstream
   creation. It should be corrected by properly specifying the pin location and
   I/O Standard.
WARNING:PhysDesignRules:2452 - The IOB QZ<65> is either not constrained (LOC) to
   a specific location and/or has an undefined I/O Standard (IOSTANDARD). This
   condition may seriously affect the device and will be an error in bitstream
   creation. It should be corrected by properly specifying the pin location and
   I/O Standard.
WARNING:PhysDesignRules:2452 - The IOB QZ<66> is either not constrained (LOC) to
   a specific location and/or has an undefined I/O Standard (IOSTANDARD). This
   condition may seriously affect the device and will be an error in bitstream
   creation. It should be corrected by properly specifying the pin location and
   I/O Standard.
WARNING:PhysDesignRules:2452 - The IOB QZ<67> is either not constrained (LOC) to
   a specific location and/or has an undefined I/O Standard (IOSTANDARD). This
   condition may seriously affect the device and will be an error in bitstream
   creation. It should be corrected by properly specifying the pin location and
   I/O Standard.
WARNING:PhysDesignRules:2452 - The IOB QZ<68> is either not constrained (LOC) to
   a specific location and/or has an undefined I/O Standard (IOSTANDARD). This
   condition may seriously affect the device and will be an error in bitstream
   creation. It should be corrected by properly specifying the pin location and
   I/O Standard.
WARNING:PhysDesignRules:2452 - The IOB QZ<69> is either not constrained (LOC) to
   a specific location and/or has an undefined I/O Standard (IOSTANDARD). This
   condition may seriously affect the device and will be an error in bitstream
   creation. It should be corrected by properly specifying the pin location and
   I/O Standard.
WARNING:PhysDesignRules:2452 - The IOB QZ<70> is either not constrained (LOC) to
   a specific location and/or has an undefined I/O Standard (IOSTANDARD). This
   condition may seriously affect the device and will be an error in bitstream
   creation. It should be corrected by properly specifying the pin location and
   I/O Standard.
WARNING:PhysDesignRules:2452 - The IOB QZ<71> is either not constrained (LOC) to
   a specific location and/or has an undefined I/O Standard (IOSTANDARD). This
   condition may seriously affect the device and will be an error in bitstream
   creation. It should be corrected by properly specifying the pin location and
   I/O Standard.
WARNING:PhysDesignRules:2452 - The IOB QZ<72> is either not constrained (LOC) to
   a specific location and/or has an undefined I/O Standard (IOSTANDARD). This
   condition may seriously affect the device and will be an error in bitstream
   creation. It should be corrected by properly specifying the pin location and
   I/O Standard.
WARNING:PhysDesignRules:2452 - The IOB QZ<73> is either not constrained (LOC) to
   a specific location and/or has an undefined I/O Standard (IOSTANDARD). This
   condition may seriously affect the device and will be an error in bitstream
   creation. It should be corrected by properly specifying the pin location and
   I/O Standard.
WARNING:PhysDesignRules:2452 - The IOB QZ<74> is either not constrained (LOC) to
   a specific location and/or has an undefined I/O Standard (IOSTANDARD). This
   condition may seriously affect the device and will be an error in bitstream
   creation. It should be corrected by properly specifying the pin location and
   I/O Standard.
WARNING:PhysDesignRules:2452 - The IOB QZ<75> is either not constrained (LOC) to
   a specific location and/or has an undefined I/O Standard (IOSTANDARD). This
   condition may seriously affect the device and will be an error in bitstream
   creation. It should be corrected by properly specifying the pin location and
   I/O Standard.
WARNING:PhysDesignRules:2452 - The IOB QZ<76> is either not constrained (LOC) to
   a specific location and/or has an undefined I/O Standard (IOSTANDARD). This
   condition may seriously affect the device and will be an error in bitstream
   creation. It should be corrected by properly specifying the pin location and
   I/O Standard.
WARNING:PhysDesignRules:2452 - The IOB QZ<77> is either not constrained (LOC) to
```

a specific location and/or has an undefined I/O Standard (IOSTANDARD). This condition may seriously affect the device and will be an error in bitstream creation. It should be corrected by properly specifying the pin location and I/O Standard.

WARNING:PhysDesignRules:2452 - The IOB QZ<78> is either not constrained (LOC) to a specific location and/or has an undefined I/O Standard (IOSTANDARD). This condition may seriously affect the device and will be an error in bitstream creation. It should be corrected by properly specifying the pin location and I/O Standard.

WARNING:PhysDesignRules:2452 - The IOB QZ<79> is either not constrained (LOC) to a specific location and/or has an undefined I/O Standard (IOSTANDARD). This condition may seriously affect the device and will be an error in bitstream creation. It should be corrected by properly specifying the pin location and I/O Standard.

WARNING:PhysDesignRules:2452 - The IOB QZ<80> is either not constrained (LOC) to a specific location and/or has an undefined I/O Standard (IOSTANDARD). This condition may seriously affect the device and will be an error in bitstream creation. It should be corrected by properly specifying the pin location and I/O Standard.

WARNING:PhysDesignRules:2452 - The IOB QZ<81> is either not constrained (LOC) to a specific location and/or has an undefined I/O Standard (IOSTANDARD). This condition may seriously affect the device and will be an error in bitstream creation. It should be corrected by properly specifying the pin location and I/O Standard.

WARNING:PhysDesignRules:2452 - The IOB QZ<82> is either not constrained (LOC) to a specific location and/or has an undefined I/O Standard (IOSTANDARD). This condition may seriously affect the device and will be an error in bitstream creation. It should be corrected by properly specifying the pin location and I/O Standard.

WARNING:PhysDesignRules:2452 - The IOB QZ<83> is either not constrained (LOC) to a specific location and/or has an undefined I/O Standard (IOSTANDARD). This condition may seriously affect the device and will be an error in bitstream creation. It should be corrected by properly specifying the pin location and I/O Standard.

WARNING:PhysDesignRules:2452 - The IOB QZ<84> is either not constrained (LOC) to a specific location and/or has an undefined I/O Standard (IOSTANDARD). This condition may seriously affect the device and will be an error in bitstream creation. It should be corrected by properly specifying the pin location and I/O Standard.

WARNING:PhysDesignRules:2452 - The IOB QZ<85> is either not constrained (LOC) to a specific location and/or has an undefined I/O Standard (IOSTANDARD). This condition may seriously affect the device and will be an error in bitstream creation. It should be corrected by properly specifying the pin location and I/O Standard.

WARNING:PhysDesignRules:2452 - The IOB QZ<86> is either not constrained (LOC) to a specific location and/or has an undefined I/O Standard (IOSTANDARD). This condition may seriously affect the device and will be an error in bitstream creation. It should be corrected by properly specifying the pin location and I/O Standard.

WARNING:PhysDesignRules:2452 - The IOB QZ<87> is either not constrained (LOC) to a specific location and/or has an undefined I/O Standard (IOSTANDARD). This condition may seriously affect the device and will be an error in bitstream creation. It should be corrected by properly specifying the pin location and I/O Standard.

WARNING:PhysDesignRules:2452 - The IOB QZ<88> is either not constrained (LOC) to a specific location and/or has an undefined I/O Standard (IOSTANDARD). This condition may seriously affect the device and will be an error in bitstream creation. It should be corrected by properly specifying the pin location and I/O Standard.

WARNING:PhysDesignRules:2452 - The IOB QZ<89> is either not constrained (LOC) to a specific location and/or has an undefined I/O Standard (IOSTANDARD). This condition may seriously affect the device and will be an error in bitstream creation. It should be corrected by properly specifying the pin location and I/O Standard.

WARNING:PhysDesignRules:2452 - The IOB QZ<90> is either not constrained (LOC) to a specific location and/or has an undefined I/O Standard (IOSTANDARD). This condition may seriously affect the device and will be an error in bitstream creation. It should be corrected by properly specifying the pin location and I/O Standard.

WARNING:PhysDesignRules:2452 - The IOB QZ<91> is either not constrained (LOC) to a specific location and/or has an undefined I/O Standard (IOSTANDARD). This

```
   condition may seriously affect the device and will be an error in bitstream
   creation. It should be corrected by properly specifying the pin location and
   I/O Standard.
WARNING:PhysDesignRules:2452 - The IOB QZ<92> is either not constrained (LOC) to
   a specific location and/or has an undefined I/O Standard (IOSTANDARD). This
   condition may seriously affect the device and will be an error in bitstream
   creation. It should be corrected by properly specifying the pin location and
   I/O Standard.
WARNING:PhysDesignRules:2452 - The IOB QZ<93> is either not constrained (LOC) to
   a specific location and/or has an undefined I/O Standard (IOSTANDARD). This
   condition may seriously affect the device and will be an error in bitstream
   creation. It should be corrected by properly specifying the pin location and
   I/O Standard.
WARNING:PhysDesignRules:2452 - The IOB QZ<94> is either not constrained (LOC) to
   a specific location and/or has an undefined I/O Standard (IOSTANDARD). This
   condition may seriously affect the device and will be an error in bitstream
   creation. It should be corrected by properly specifying the pin location and
   I/O Standard.
WARNING:PhysDesignRules:2452 - The IOB QZ<95> is either not constrained (LOC) to
   a specific location and/or has an undefined I/O Standard (IOSTANDARD). This
   condition may seriously affect the device and will be an error in bitstream
   creation. It should be corrected by properly specifying the pin location and
   I/O Standard.
WARNING:PhysDesignRules:2452 - The IOB QZ<96> is either not constrained (LOC) to
   a specific location and/or has an undefined I/O Standard (IOSTANDARD). This
   condition may seriously affect the device and will be an error in bitstream
   creation. It should be corrected by properly specifying the pin location and
   I/O Standard.
WARNING:PhysDesignRules:2452 - The IOB QZ<97> is either not constrained (LOC) to
   a specific location and/or has an undefined I/O Standard (IOSTANDARD). This
   condition may seriously affect the device and will be an error in bitstream
   creation. It should be corrected by properly specifying the pin location and
   I/O Standard.
WARNING:PhysDesignRules:2452 - The IOB QZ<98> is either not constrained (LOC) to
   a specific location and/or has an undefined I/O Standard (IOSTANDARD). This
   condition may seriously affect the device and will be an error in bitstream
   creation. It should be corrected by properly specifying the pin location and
   I/O Standard.
WARNING:PhysDesignRules:2452 - The IOB QZ<99> is either not constrained (LOC) to
   a specific location and/or has an undefined I/O Standard (IOSTANDARD). This
   condition may seriously affect the device and will be an error in bitstream
   creation. It should be corrected by properly specifying the pin location and
   I/O Standard.
WARNING:PhysDesignRules:2452 - The IOB QZ<102> is either not constrained (LOC)
   to a specific location and/or has an undefined I/O Standard (IOSTANDARD).
   This condition may seriously affect the device and will be an error in
   bitstream creation. It should be corrected by properly specifying the pin
   location and I/O Standard.
WARNING:PhysDesignRules:2452 - The IOB QZ<101> is either not constrained (LOC)
   to a specific location and/or has an undefined I/O Standard (IOSTANDARD).
   This condition may seriously affect the device and will be an error in
   bitstream creation. It should be corrected by properly specifying the pin
   location and I/O Standard.
WARNING:PhysDesignRules:2452 - The IOB QZ<104> is either not constrained (LOC)
   to a specific location and/or has an undefined I/O Standard (IOSTANDARD).
   This condition may seriously affect the device and will be an error in
   bitstream creation. It should be corrected by properly specifying the pin
   location and I/O Standard.
WARNING:PhysDesignRules:2452 - The IOB QZ<103> is either not constrained (LOC)
   to a specific location and/or has an undefined I/O Standard (IOSTANDARD).
   This condition may seriously affect the device and will be an error in
   bitstream creation. It should be corrected by properly specifying the pin
   location and I/O Standard.
WARNING:PhysDesignRules:2452 - The IOB QZ<100> is either not constrained (LOC)
   to a specific location and/or has an undefined I/O Standard (IOSTANDARD).
   This condition may seriously affect the device and will be an error in
   bitstream creation. It should be corrected by properly specifying the pin
   location and I/O Standard.
WARNING:PhysDesignRules:2452 - The IOB QZ<109> is either not constrained (LOC)
   to a specific location and/or has an undefined I/O Standard (IOSTANDARD).
   This condition may seriously affect the device and will be an error in
```

```
   bitstream creation. It should be corrected by properly specifying the pin
   location and I/O Standard.
WARNING:PhysDesignRules:2452 - The IOB QZ<106> is either not constrained (LOC)
   to a specific location and/or has an undefined I/O Standard (IOSTANDARD).
   This condition may seriously affect the device and will be an error in
   bitstream creation. It should be corrected by properly specifying the pin
   location and I/O Standard.
WARNING:PhysDesignRules:2452 - The IOB QZ<105> is either not constrained (LOC)
   to a specific location and/or has an undefined I/O Standard (IOSTANDARD).
   This condition may seriously affect the device and will be an error in
   bitstream creation. It should be corrected by properly specifying the pin
   location and I/O Standard.
WARNING:PhysDesignRules:2452 - The IOB done is either not constrained (LOC) to a
   specific location and/or has an undefined I/O Standard (IOSTANDARD). This
   condition may seriously affect the device and will be an error in bitstream
   creation. It should be corrected by properly specifying the pin location and
   I/O Standard.
WARNING:PhysDesignRules:2452 - The IOB QZ<108> is either not constrained (LOC)
   to a specific location and/or has an undefined I/O Standard (IOSTANDARD).
   This condition may seriously affect the device and will be an error in
   bitstream creation. It should be corrected by properly specifying the pin
   location and I/O Standard.
WARNING:PhysDesignRules:2452 - The IOB QZ<107> is either not constrained (LOC)
   to a specific location and/or has an undefined I/O Standard (IOSTANDARD).
   This condition may seriously affect the device and will be an error in
   bitstream creation. It should be corrected by properly specifying the pin
   location and I/O Standard.
WARNING:PhysDesignRules:2452 - The IOB QZ<112> is either not constrained (LOC)
   to a specific location and/or has an undefined I/O Standard (IOSTANDARD).
   This condition may seriously affect the device and will be an error in
   bitstream creation. It should be corrected by properly specifying the pin
   location and I/O Standard.
WARNING:PhysDesignRules:2452 - The IOB QZ<111> is either not constrained (LOC)
   to a specific location and/or has an undefined I/O Standard (IOSTANDARD).
   This condition may seriously affect the device and will be an error in
   bitstream creation. It should be corrected by properly specifying the pin
   location and I/O Standard.
WARNING:PhysDesignRules:2452 - The IOB QZ<114> is either not constrained (LOC)
   to a specific location and/or has an undefined I/O Standard (IOSTANDARD).
   This condition may seriously affect the device and will be an error in
   bitstream creation. It should be corrected by properly specifying the pin
   location and I/O Standard.
WARNING:PhysDesignRules:2452 - The IOB QZ<113> is either not constrained (LOC)
   to a specific location and/or has an undefined I/O Standard (IOSTANDARD).
   This condition may seriously affect the device and will be an error in
   bitstream creation. It should be corrected by properly specifying the pin
   location and I/O Standard.
WARNING:PhysDesignRules:2452 - The IOB QZ<110> is either not constrained (LOC)
   to a specific location and/or has an undefined I/O Standard (IOSTANDARD).
   This condition may seriously affect the device and will be an error in
   bitstream creation. It should be corrected by properly specifying the pin
   location and I/O Standard.
WARNING:PhysDesignRules:2452 - The IOB QZ<119> is either not constrained (LOC)
   to a specific location and/or has an undefined I/O Standard (IOSTANDARD).
   This condition may seriously affect the device and will be an error in
   bitstream creation. It should be corrected by properly specifying the pin
   location and I/O Standard.
WARNING:PhysDesignRules:2452 - The IOB QZ<116> is either not constrained (LOC)
   to a specific location and/or has an undefined I/O Standard (IOSTANDARD).
   This condition may seriously affect the device and will be an error in
   bitstream creation. It should be corrected by properly specifying the pin
   location and I/O Standard.
WARNING:PhysDesignRules:2452 - The IOB QZ<115> is either not constrained (LOC)
   to a specific location and/or has an undefined I/O Standard (IOSTANDARD).
   This condition may seriously affect the device and will be an error in
   bitstream creation. It should be corrected by properly specifying the pin
   location and I/O Standard.
WARNING:PhysDesignRules:2452 - The IOB QZ<118> is either not constrained (LOC)
   to a specific location and/or has an undefined I/O Standard (IOSTANDARD).
   This condition may seriously affect the device and will be an error in
   bitstream creation. It should be corrected by properly specifying the pin
```

```
   location and I/O Standard.
WARNING:PhysDesignRules:2452 - The IOB QZ<117> is either not constrained (LOC)
   to a specific location and/or has an undefined I/O Standard (IOSTANDARD).
   This condition may seriously affect the device and will be an error in
   bitstream creation. It should be corrected by properly specifying the pin
   location and I/O Standard.
WARNING:PhysDesignRules:2452 - The IOB QZ<122> is either not constrained (LOC)
   to a specific location and/or has an undefined I/O Standard (IOSTANDARD).
   This condition may seriously affect the device and will be an error in
   bitstream creation. It should be corrected by properly specifying the pin
   location and I/O Standard.
WARNING:PhysDesignRules:2452 - The IOB QZ<121> is either not constrained (LOC)
   to a specific location and/or has an undefined I/O Standard (IOSTANDARD).
   This condition may seriously affect the device and will be an error in
   bitstream creation. It should be corrected by properly specifying the pin
   location and I/O Standard.
WARNING:PhysDesignRules:2452 - The IOB QZ<124> is either not constrained (LOC)
   to a specific location and/or has an undefined I/O Standard (IOSTANDARD).
   This condition may seriously affect the device and will be an error in
   bitstream creation. It should be corrected by properly specifying the pin
   location and I/O Standard.
WARNING:PhysDesignRules:2452 - The IOB QZ<123> is either not constrained (LOC)
   to a specific location and/or has an undefined I/O Standard (IOSTANDARD).
   This condition may seriously affect the device and will be an error in
   bitstream creation. It should be corrected by properly specifying the pin
   location and I/O Standard.
WARNING:PhysDesignRules:2452 - The IOB QZ<120> is either not constrained (LOC)
   to a specific location and/or has an undefined I/O Standard (IOSTANDARD).
   This condition may seriously affect the device and will be an error in
   bitstream creation. It should be corrected by properly specifying the pin
   location and I/O Standard.
WARNING:PhysDesignRules:2452 - The IOB QZ<129> is either not constrained (LOC)
   to a specific location and/or has an undefined I/O Standard (IOSTANDARD).
   This condition may seriously affect the device and will be an error in
   bitstream creation. It should be corrected by properly specifying the pin
   location and I/O Standard.
WARNING:PhysDesignRules:2452 - The IOB QZ<126> is either not constrained (LOC)
   to a specific location and/or has an undefined I/O Standard (IOSTANDARD).
   This condition may seriously affect the device and will be an error in
   bitstream creation. It should be corrected by properly specifying the pin
   location and I/O Standard.
WARNING:PhysDesignRules:2452 - The IOB QZ<125> is either not constrained (LOC)
   to a specific location and/or has an undefined I/O Standard (IOSTANDARD).
   This condition may seriously affect the device and will be an error in
   bitstream creation. It should be corrected by properly specifying the pin
   location and I/O Standard.
WARNING:PhysDesignRules:2452 - The IOB QZ<128> is either not constrained (LOC)
   to a specific location and/or has an undefined I/O Standard (IOSTANDARD).
   This condition may seriously affect the device and will be an error in
   bitstream creation. It should be corrected by properly specifying the pin
   location and I/O Standard.
WARNING:PhysDesignRules:2452 - The IOB QZ<127> is either not constrained (LOC)
   to a specific location and/or has an undefined I/O Standard (IOSTANDARD).
   This condition may seriously affect the device and will be an error in
   bitstream creation. It should be corrected by properly specifying the pin
   location and I/O Standard.
WARNING:PhysDesignRules:2452 - The IOB QZ<132> is either not constrained (LOC)
   to a specific location and/or has an undefined I/O Standard (IOSTANDARD).
   This condition may seriously affect the device and will be an error in
   bitstream creation. It should be corrected by properly specifying the pin
   location and I/O Standard.
WARNING:PhysDesignRules:2452 - The IOB QZ<131> is either not constrained (LOC)
   to a specific location and/or has an undefined I/O Standard (IOSTANDARD).
   This condition may seriously affect the device and will be an error in
   bitstream creation. It should be corrected by properly specifying the pin
   location and I/O Standard.
WARNING:PhysDesignRules:2452 - The IOB QZ<134> is either not constrained (LOC)
   to a specific location and/or has an undefined I/O Standard (IOSTANDARD).
   This condition may seriously affect the device and will be an error in
   bitstream creation. It should be corrected by properly specifying the pin
   location and I/O Standard.
```

```
WARNING:PhysDesignRules:2452 - The IOB QZ<133> is either not constrained (LOC)
   to a specific location and/or has an undefined I/O Standard (IOSTANDARD).
   This condition may seriously affect the device and will be an error in
   bitstream creation. It should be corrected by properly specifying the pin
   location and I/O Standard.
WARNING:PhysDesignRules:2452 - The IOB QZ<130> is either not constrained (LOC)
   to a specific location and/or has an undefined I/O Standard (IOSTANDARD).
   This condition may seriously affect the device and will be an error in
   bitstream creation. It should be corrected by properly specifying the pin
   location and I/O Standard.
WARNING:PhysDesignRules:2452 - The IOB QZ<139> is either not constrained (LOC)
   to a specific location and/or has an undefined I/O Standard (IOSTANDARD).
   This condition may seriously affect the device and will be an error in
   bitstream creation. It should be corrected by properly specifying the pin
   location and I/O Standard.
WARNING:PhysDesignRules:2452 - The IOB QZ<136> is either not constrained (LOC)
   to a specific location and/or has an undefined I/O Standard (IOSTANDARD).
   This condition may seriously affect the device and will be an error in
   bitstream creation. It should be corrected by properly specifying the pin
   location and I/O Standard.
WARNING:PhysDesignRules:2452 - The IOB QZ<135> is either not constrained (LOC)
   to a specific location and/or has an undefined I/O Standard (IOSTANDARD).
   This condition may seriously affect the device and will be an error in
   bitstream creation. It should be corrected by properly specifying the pin
   location and I/O Standard.
WARNING:PhysDesignRules:2452 - The IOB QZ<138> is either not constrained (LOC)
   to a specific location and/or has an undefined I/O Standard (IOSTANDARD).
   This condition may seriously affect the device and will be an error in
   bitstream creation. It should be corrected by properly specifying the pin
   location and I/O Standard.
WARNING:PhysDesignRules:2452 - The IOB QZ<137> is either not constrained (LOC)
   to a specific location and/or has an undefined I/O Standard (IOSTANDARD).
   This condition may seriously affect the device and will be an error in
   bitstream creation. It should be corrected by properly specifying the pin
   location and I/O Standard.
WARNING:PhysDesignRules:2452 - The IOB QZ<142> is either not constrained (LOC)
   to a specific location and/or has an undefined I/O Standard (IOSTANDARD).
   This condition may seriously affect the device and will be an error in
   bitstream creation. It should be corrected by properly specifying the pin
   location and I/O Standard.
WARNING:PhysDesignRules:2452 - The IOB QZ<141> is either not constrained (LOC)
   to a specific location and/or has an undefined I/O Standard (IOSTANDARD).
   This condition may seriously affect the device and will be an error in
   bitstream creation. It should be corrected by properly specifying the pin
   location and I/O Standard.
WARNING:PhysDesignRules:2452 - The IOB QZ<144> is either not constrained (LOC)
   to a specific location and/or has an undefined I/O Standard (IOSTANDARD).
   This condition may seriously affect the device and will be an error in
   bitstream creation. It should be corrected by properly specifying the pin
   location and I/O Standard.
WARNING:PhysDesignRules:2452 - The IOB QZ<143> is either not constrained (LOC)
   to a specific location and/or has an undefined I/O Standard (IOSTANDARD).
   This condition may seriously affect the device and will be an error in
   bitstream creation. It should be corrected by properly specifying the pin
   location and I/O Standard.
WARNING:PhysDesignRules:2452 - The IOB QZ<140> is either not constrained (LOC)
   to a specific location and/or has an undefined I/O Standard (IOSTANDARD).
   This condition may seriously affect the device and will be an error in
   bitstream creation. It should be corrected by properly specifying the pin
   location and I/O Standard.
WARNING:PhysDesignRules:2452 - The IOB QZ<149> is either not constrained (LOC)
   to a specific location and/or has an undefined I/O Standard (IOSTANDARD).
   This condition may seriously affect the device and will be an error in
   bitstream creation. It should be corrected by properly specifying the pin
   location and I/O Standard.
WARNING:PhysDesignRules:2452 - The IOB QZ<146> is either not constrained (LOC)
   to a specific location and/or has an undefined I/O Standard (IOSTANDARD).
   This condition may seriously affect the device and will be an error in
   bitstream creation. It should be corrected by properly specifying the pin
   location and I/O Standard.
WARNING:PhysDesignRules:2452 - The IOB QZ<145> is either not constrained (LOC)
```

```
   to a specific location and/or has an undefined I/O Standard (IOSTANDARD).
   This condition may seriously affect the device and will be an error in
   bitstream creation. It should be corrected by properly specifying the pin
   location and I/O Standard.
WARNING:PhysDesignRules:2452 - The IOB QZ<148> is either not constrained (LOC)
   to a specific location and/or has an undefined I/O Standard (IOSTANDARD).
   This condition may seriously affect the device and will be an error in
   bitstream creation. It should be corrected by properly specifying the pin
   location and I/O Standard.
WARNING:PhysDesignRules:2452 - The IOB QZ<147> is either not constrained (LOC)
   to a specific location and/or has an undefined I/O Standard (IOSTANDARD).
   This condition may seriously affect the device and will be an error in
   bitstream creation. It should be corrected by properly specifying the pin
   location and I/O Standard.
WARNING:PhysDesignRules:2452 - The IOB QZ<152> is either not constrained (LOC)
   to a specific location and/or has an undefined I/O Standard (IOSTANDARD).
   This condition may seriously affect the device and will be an error in
   bitstream creation. It should be corrected by properly specifying the pin
   location and I/O Standard.
WARNING:PhysDesignRules:2452 - The IOB QZ<151> is either not constrained (LOC)
   to a specific location and/or has an undefined I/O Standard (IOSTANDARD).
   This condition may seriously affect the device and will be an error in
   bitstream creation. It should be corrected by properly specifying the pin
   location and I/O Standard.
WARNING:PhysDesignRules:2452 - The IOB QZ<154> is either not constrained (LOC)
   to a specific location and/or has an undefined I/O Standard (IOSTANDARD).
   This condition may seriously affect the device and will be an error in
   bitstream creation. It should be corrected by properly specifying the pin
   location and I/O Standard.
WARNING:PhysDesignRules:2452 - The IOB QZ<153> is either not constrained (LOC)
   to a specific location and/or has an undefined I/O Standard (IOSTANDARD).
   This condition may seriously affect the device and will be an error in
   bitstream creation. It should be corrected by properly specifying the pin
   location and I/O Standard.
WARNING:PhysDesignRules:2452 - The IOB QZ<150> is either not constrained (LOC)
   to a specific location and/or has an undefined I/O Standard (IOSTANDARD).
   This condition may seriously affect the device and will be an error in
   bitstream creation. It should be corrected by properly specifying the pin
   location and I/O Standard.
WARNING:PhysDesignRules:2452 - The IOB QZ<159> is either not constrained (LOC)
   to a specific location and/or has an undefined I/O Standard (IOSTANDARD).
   This condition may seriously affect the device and will be an error in
   bitstream creation. It should be corrected by properly specifying the pin
   location and I/O Standard.
WARNING:PhysDesignRules:2452 - The IOB QZ<156> is either not constrained (LOC)
   to a specific location and/or has an undefined I/O Standard (IOSTANDARD).
   This condition may seriously affect the device and will be an error in
   bitstream creation. It should be corrected by properly specifying the pin
   location and I/O Standard.
WARNING:PhysDesignRules:2452 - The IOB QZ<155> is either not constrained (LOC)
   to a specific location and/or has an undefined I/O Standard (IOSTANDARD).
   This condition may seriously affect the device and will be an error in
   bitstream creation. It should be corrected by properly specifying the pin
   location and I/O Standard.
WARNING:PhysDesignRules:2452 - The IOB QZ<158> is either not constrained (LOC)
   to a specific location and/or has an undefined I/O Standard (IOSTANDARD).
   This condition may seriously affect the device and will be an error in
   bitstream creation. It should be corrected by properly specifying the pin
   location and I/O Standard.
WARNING:PhysDesignRules:2452 - The IOB QZ<157> is either not constrained (LOC)
   to a specific location and/or has an undefined I/O Standard (IOSTANDARD).
   This condition may seriously affect the device and will be an error in
   bitstream creation. It should be corrected by properly specifying the pin
   location and I/O Standard.
WARNING:PhysDesignRules:2452 - The IOB QZ<162> is either not constrained (LOC)
   to a specific location and/or has an undefined I/O Standard (IOSTANDARD).
   This condition may seriously affect the device and will be an error in
   bitstream creation. It should be corrected by properly specifying the pin
   location and I/O Standard.
WARNING:PhysDesignRules:2452 - The IOB QZ<0> is either not constrained (LOC) to
   a specific location and/or has an undefined I/O Standard (IOSTANDARD). This
```

```
   condition may seriously affect the device and will be an error in bitstream
   creation. It should be corrected by properly specifying the pin location and
   I/O Standard.
WARNING:PhysDesignRules:2452 - The IOB QZ<161> is either not constrained (LOC)
   to a specific location and/or has an undefined I/O Standard (IOSTANDARD).
   This condition may seriously affect the device and will be an error in
   bitstream creation. It should be corrected by properly specifying the pin
   location and I/O Standard.
WARNING:PhysDesignRules:2452 - The IOB QZ<1> is either not constrained (LOC) to
   a specific location and/or has an undefined I/O Standard (IOSTANDARD). This
   condition may seriously affect the device and will be an error in bitstream
   creation. It should be corrected by properly specifying the pin location and
   I/O Standard.
WARNING:PhysDesignRules:2452 - The IOB QZ<164> is either not constrained (LOC)
   to a specific location and/or has an undefined I/O Standard (IOSTANDARD).
   This condition may seriously affect the device and will be an error in
   bitstream creation. It should be corrected by properly specifying the pin
   location and I/O Standard.
WARNING:PhysDesignRules:2452 - The IOB QZ<2> is either not constrained (LOC) to
   a specific location and/or has an undefined I/O Standard (IOSTANDARD). This
   condition may seriously affect the device and will be an error in bitstream
   creation. It should be corrected by properly specifying the pin location and
   I/O Standard.
WARNING:PhysDesignRules:2452 - The IOB QZ<163> is either not constrained (LOC)
   to a specific location and/or has an undefined I/O Standard (IOSTANDARD).
   This condition may seriously affect the device and will be an error in
   bitstream creation. It should be corrected by properly specifying the pin
   location and I/O Standard.
WARNING:PhysDesignRules:2452 - The IOB QZ<3> is either not constrained (LOC) to
   a specific location and/or has an undefined I/O Standard (IOSTANDARD). This
   condition may seriously affect the device and will be an error in bitstream
   creation. It should be corrected by properly specifying the pin location and
   I/O Standard.
WARNING:PhysDesignRules:2452 - The IOB QZ<4> is either not constrained (LOC) to
   a specific location and/or has an undefined I/O Standard (IOSTANDARD). This
   condition may seriously affect the device and will be an error in bitstream
   creation. It should be corrected by properly specifying the pin location and
   I/O Standard.
WARNING:PhysDesignRules:2452 - The IOB QZ<5> is either not constrained (LOC) to
   a specific location and/or has an undefined I/O Standard (IOSTANDARD). This
   condition may seriously affect the device and will be an error in bitstream
   creation. It should be corrected by properly specifying the pin location and
   I/O Standard.
WARNING:PhysDesignRules:2452 - The IOB QZ<160> is either not constrained (LOC)
   to a specific location and/or has an undefined I/O Standard (IOSTANDARD).
   This condition may seriously affect the device and will be an error in
   bitstream creation. It should be corrected by properly specifying the pin
   location and I/O Standard.
WARNING:PhysDesignRules:2452 - The IOB QZ<6> is either not constrained (LOC) to
   a specific location and/or has an undefined I/O Standard (IOSTANDARD). This
   condition may seriously affect the device and will be an error in bitstream
   creation. It should be corrected by properly specifying the pin location and
   I/O Standard.
WARNING:PhysDesignRules:2452 - The IOB QZ<7> is either not constrained (LOC) to
   a specific location and/or has an undefined I/O Standard (IOSTANDARD). This
   condition may seriously affect the device and will be an error in bitstream
   creation. It should be corrected by properly specifying the pin location and
   I/O Standard.
WARNING:PhysDesignRules:2452 - The IOB QZ<8> is either not constrained (LOC) to
   a specific location and/or has an undefined I/O Standard (IOSTANDARD). This
   condition may seriously affect the device and will be an error in bitstream
   creation. It should be corrected by properly specifying the pin location and
   I/O Standard.
WARNING:PhysDesignRules:2452 - The IOB QZ<169> is either not constrained (LOC)
   to a specific location and/or has an undefined I/O Standard (IOSTANDARD).
   This condition may seriously affect the device and will be an error in
   bitstream creation. It should be corrected by properly specifying the pin
   location and I/O Standard.
WARNING:PhysDesignRules:2452 - The IOB QZ<9> is either not constrained (LOC) to
   a specific location and/or has an undefined I/O Standard (IOSTANDARD). This
   condition may seriously affect the device and will be an error in bitstream
```

```
   creation. It should be corrected by properly specifying the pin location and
   I/O Standard.
WARNING:PhysDesignRules:2452 - The IOB QZ<166> is either not constrained (LOC)
   to a specific location and/or has an undefined I/O Standard (IOSTANDARD).
   This condition may seriously affect the device and will be an error in
   bitstream creation. It should be corrected by properly specifying the pin
   location and I/O Standard.
WARNING:PhysDesignRules:2452 - The IOB QZ<165> is either not constrained (LOC)
   to a specific location and/or has an undefined I/O Standard (IOSTANDARD).
   This condition may seriously affect the device and will be an error in
   bitstream creation. It should be corrected by properly specifying the pin
   location and I/O Standard.
WARNING:PhysDesignRules:2452 - The IOB QZ<168> is either not constrained (LOC)
   to a specific location and/or has an undefined I/O Standard (IOSTANDARD).
   This condition may seriously affect the device and will be an error in
   bitstream creation. It should be corrected by properly specifying the pin
   location and I/O Standard.
WARNING:PhysDesignRules:2452 - The IOB QZ<167> is either not constrained (LOC)
   to a specific location and/or has an undefined I/O Standard (IOSTANDARD).
   This condition may seriously affect the device and will be an error in
   bitstream creation. It should be corrected by properly specifying the pin
   location and I/O Standard.
WARNING:PhysDesignRules:2452 - The IOB QZ<172> is either not constrained (LOC)
   to a specific location and/or has an undefined I/O Standard (IOSTANDARD).
   This condition may seriously affect the device and will be an error in
   bitstream creation. It should be corrected by properly specifying the pin
   location and I/O Standard.
WARNING:PhysDesignRules:2452 - The IOB QZ<171> is either not constrained (LOC)
   to a specific location and/or has an undefined I/O Standard (IOSTANDARD).
   This condition may seriously affect the device and will be an error in
   bitstream creation. It should be corrected by properly specifying the pin
   location and I/O Standard.
WARNING:PhysDesignRules:2452 - The IOB QZ<174> is either not constrained (LOC)
   to a specific location and/or has an undefined I/O Standard (IOSTANDARD).
   This condition may seriously affect the device and will be an error in
   bitstream creation. It should be corrected by properly specifying the pin
   location and I/O Standard.
WARNING:PhysDesignRules:2452 - The IOB QZ<173> is either not constrained (LOC)
   to a specific location and/or has an undefined I/O Standard (IOSTANDARD).
   This condition may seriously affect the device and will be an error in
   bitstream creation. It should be corrected by properly specifying the pin
   location and I/O Standard.
WARNING:PhysDesignRules:2452 - The IOB QZ<170> is either not constrained (LOC)
   to a specific location and/or has an undefined I/O Standard (IOSTANDARD).
   This condition may seriously affect the device and will be an error in
   bitstream creation. It should be corrected by properly specifying the pin
   location and I/O Standard.
WARNING:PhysDesignRules:2452 - The IOB QZ<179> is either not constrained (LOC)
   to a specific location and/or has an undefined I/O Standard (IOSTANDARD).
   This condition may seriously affect the device and will be an error in
   bitstream creation. It should be corrected by properly specifying the pin
   location and I/O Standard.
WARNING:PhysDesignRules:2452 - The IOB QZ<176> is either not constrained (LOC)
   to a specific location and/or has an undefined I/O Standard (IOSTANDARD).
   This condition may seriously affect the device and will be an error in
   bitstream creation. It should be corrected by properly specifying the pin
   location and I/O Standard.
WARNING:PhysDesignRules:2452 - The IOB QZ<175> is either not constrained (LOC)
   to a specific location and/or has an undefined I/O Standard (IOSTANDARD).
   This condition may seriously affect the device and will be an error in
   bitstream creation. It should be corrected by properly specifying the pin
   location and I/O Standard.
WARNING:PhysDesignRules:2452 - The IOB QZ<178> is either not constrained (LOC)
   to a specific location and/or has an undefined I/O Standard (IOSTANDARD).
   This condition may seriously affect the device and will be an error in
   bitstream creation. It should be corrected by properly specifying the pin
   location and I/O Standard.
WARNING:PhysDesignRules:2452 - The IOB QZ<177> is either not constrained (LOC)
   to a specific location and/or has an undefined I/O Standard (IOSTANDARD).
   This condition may seriously affect the device and will be an error in
   bitstream creation. It should be corrected by properly specifying the pin
```

```
   location and I/O Standard.
WARNING:PhysDesignRules:2452 - The IOB QZ<182> is either not constrained (LOC)
   to a specific location and/or has an undefined I/O Standard (IOSTANDARD).
   This condition may seriously affect the device and will be an error in
   bitstream creation. It should be corrected by properly specifying the pin
   location and I/O Standard.
WARNING:PhysDesignRules:2452 - The IOB QZ<181> is either not constrained (LOC)
   to a specific location and/or has an undefined I/O Standard (IOSTANDARD).
   This condition may seriously affect the device and will be an error in
   bitstream creation. It should be corrected by properly specifying the pin
   location and I/O Standard.
WARNING:PhysDesignRules:2452 - The IOB QZ<184> is either not constrained (LOC)
   to a specific location and/or has an undefined I/O Standard (IOSTANDARD).
   This condition may seriously affect the device and will be an error in
   bitstream creation. It should be corrected by properly specifying the pin
   location and I/O Standard.
WARNING:PhysDesignRules:2452 - The IOB QZ<183> is either not constrained (LOC)
   to a specific location and/or has an undefined I/O Standard (IOSTANDARD).
   This condition may seriously affect the device and will be an error in
   bitstream creation. It should be corrected by properly specifying the pin
   location and I/O Standard.
WARNING:PhysDesignRules:2452 - The IOB QZ<180> is either not constrained (LOC)
   to a specific location and/or has an undefined I/O Standard (IOSTANDARD).
   This condition may seriously affect the device and will be an error in
   bitstream creation. It should be corrected by properly specifying the pin
   location and I/O Standard.
WARNING:PhysDesignRules:2452 - The IOB QZ<189> is either not constrained (LOC)
   to a specific location and/or has an undefined I/O Standard (IOSTANDARD).
   This condition may seriously affect the device and will be an error in
   bitstream creation. It should be corrected by properly specifying the pin
   location and I/O Standard.
WARNING:PhysDesignRules:2452 - The IOB QZ<186> is either not constrained (LOC)
   to a specific location and/or has an undefined I/O Standard (IOSTANDARD).
   This condition may seriously affect the device and will be an error in
   bitstream creation. It should be corrected by properly specifying the pin
   location and I/O Standard.
WARNING:PhysDesignRules:2452 - The IOB QZ<185> is either not constrained (LOC)
   to a specific location and/or has an undefined I/O Standard (IOSTANDARD).
   This condition may seriously affect the device and will be an error in
   bitstream creation. It should be corrected by properly specifying the pin
   location and I/O Standard.
WARNING:PhysDesignRules:2452 - The IOB QZ<188> is either not constrained (LOC)
   to a specific location and/or has an undefined I/O Standard (IOSTANDARD).
   This condition may seriously affect the device and will be an error in
   bitstream creation. It should be corrected by properly specifying the pin
   location and I/O Standard.
WARNING:PhysDesignRules:2452 - The IOB QZ<187> is either not constrained (LOC)
   to a specific location and/or has an undefined I/O Standard (IOSTANDARD).
   This condition may seriously affect the device and will be an error in
   bitstream creation. It should be corrected by properly specifying the pin
   location and I/O Standard.
WARNING:PhysDesignRules:2452 - The IOB QZ<192> is either not constrained (LOC)
   to a specific location and/or has an undefined I/O Standard (IOSTANDARD).
   This condition may seriously affect the device and will be an error in
   bitstream creation. It should be corrected by properly specifying the pin
   location and I/O Standard.
WARNING:PhysDesignRules:2452 - The IOB QZ<191> is either not constrained (LOC)
   to a specific location and/or has an undefined I/O Standard (IOSTANDARD).
   This condition may seriously affect the device and will be an error in
   bitstream creation. It should be corrected by properly specifying the pin
   location and I/O Standard.
WARNING:PhysDesignRules:2452 - The IOB QZ<194> is either not constrained (LOC)
   to a specific location and/or has an undefined I/O Standard (IOSTANDARD).
   This condition may seriously affect the device and will be an error in
   bitstream creation. It should be corrected by properly specifying the pin
   location and I/O Standard.
WARNING:PhysDesignRules:2452 - The IOB QZ<193> is either not constrained (LOC)
   to a specific location and/or has an undefined I/O Standard (IOSTANDARD).
   This condition may seriously affect the device and will be an error in
   bitstream creation. It should be corrected by properly specifying the pin
   location and I/O Standard.
```

```
WARNING:PhysDesignRules:2452 - The IOB QZ<190> is either not constrained (LOC)
   to a specific location and/or has an undefined I/O Standard (IOSTANDARD).
   This condition may seriously affect the device and will be an error in
   bitstream creation. It should be corrected by properly specifying the pin
   location and I/O Standard.
WARNING:PhysDesignRules:2452 - The IOB QZ<199> is either not constrained (LOC)
   to a specific location and/or has an undefined I/O Standard (IOSTANDARD).
   This condition may seriously affect the device and will be an error in
   bitstream creation. It should be corrected by properly specifying the pin
   location and I/O Standard.
WARNING:PhysDesignRules:2452 - The IOB QZ<196> is either not constrained (LOC)
   to a specific location and/or has an undefined I/O Standard (IOSTANDARD).
   This condition may seriously affect the device and will be an error in
   bitstream creation. It should be corrected by properly specifying the pin
   location and I/O Standard.
WARNING:PhysDesignRules:2452 - The IOB QZ<195> is either not constrained (LOC)
   to a specific location and/or has an undefined I/O Standard (IOSTANDARD).
   This condition may seriously affect the device and will be an error in
   bitstream creation. It should be corrected by properly specifying the pin
   location and I/O Standard.
WARNING:PhysDesignRules:2452 - The IOB QZ<198> is either not constrained (LOC)
   to a specific location and/or has an undefined I/O Standard (IOSTANDARD).
   This condition may seriously affect the device and will be an error in
   bitstream creation. It should be corrected by properly specifying the pin
   location and I/O Standard.
WARNING:PhysDesignRules:2452 - The IOB QZ<197> is either not constrained (LOC)
   to a specific location and/or has an undefined I/O Standard (IOSTANDARD).
   This condition may seriously affect the device and will be an error in
   bitstream creation. It should be corrected by properly specifying the pin
   location and I/O Standard.
WARNING:PhysDesignRules:2452 - The IOB QX<102> is either not constrained (LOC)
   to a specific location and/or has an undefined I/O Standard (IOSTANDARD).
   This condition may seriously affect the device and will be an error in
   bitstream creation. It should be corrected by properly specifying the pin
   location and I/O Standard.
WARNING:PhysDesignRules:2452 - The IOB QX<101> is either not constrained (LOC)
   to a specific location and/or has an undefined I/O Standard (IOSTANDARD).
   This condition may seriously affect the device and will be an error in
   bitstream creation. It should be corrected by properly specifying the pin
   location and I/O Standard.
WARNING:PhysDesignRules:2452 - The IOB QX<104> is either not constrained (LOC)
   to a specific location and/or has an undefined I/O Standard (IOSTANDARD).
   This condition may seriously affect the device and will be an error in
   bitstream creation. It should be corrected by properly specifying the pin
   location and I/O Standard.
WARNING:PhysDesignRules:2452 - The IOB QX<103> is either not constrained (LOC)
   to a specific location and/or has an undefined I/O Standard (IOSTANDARD).
   This condition may seriously affect the device and will be an error in
   bitstream creation. It should be corrected by properly specifying the pin
   location and I/O Standard.
WARNING:PhysDesignRules:2452 - The IOB QX<100> is either not constrained (LOC)
   to a specific location and/or has an undefined I/O Standard (IOSTANDARD).
   This condition may seriously affect the device and will be an error in
   bitstream creation. It should be corrected by properly specifying the pin
   location and I/O Standard.
WARNING:PhysDesignRules:2452 - The IOB QX<109> is either not constrained (LOC)
   to a specific location and/or has an undefined I/O Standard (IOSTANDARD).
   This condition may seriously affect the device and will be an error in
   bitstream creation. It should be corrected by properly specifying the pin
   location and I/O Standard.
WARNING:PhysDesignRules:2452 - The IOB QX<106> is either not constrained (LOC)
   to a specific location and/or has an undefined I/O Standard (IOSTANDARD).
   This condition may seriously affect the device and will be an error in
   bitstream creation. It should be corrected by properly specifying the pin
   location and I/O Standard.
WARNING:PhysDesignRules:2452 - The IOB QX<105> is either not constrained (LOC)
   to a specific location and/or has an undefined I/O Standard (IOSTANDARD).
   This condition may seriously affect the device and will be an error in
   bitstream creation. It should be corrected by properly specifying the pin
   location and I/O Standard.
WARNING:PhysDesignRules:2452 - The IOB QX<108> is either not constrained (LOC)
```

```
   to a specific location and/or has an undefined I/O Standard (IOSTANDARD).
   This condition may seriously affect the device and will be an error in
   bitstream creation. It should be corrected by properly specifying the pin
   location and I/O Standard.
WARNING:PhysDesignRules:2452 - The IOB QX<107> is either not constrained (LOC)
   to a specific location and/or has an undefined I/O Standard (IOSTANDARD).
   This condition may seriously affect the device and will be an error in
   bitstream creation. It should be corrected by properly specifying the pin
   location and I/O Standard.
WARNING:PhysDesignRules:2452 - The IOB QX<112> is either not constrained (LOC)
   to a specific location and/or has an undefined I/O Standard (IOSTANDARD).
   This condition may seriously affect the device and will be an error in
   bitstream creation. It should be corrected by properly specifying the pin
   location and I/O Standard.
WARNING:PhysDesignRules:2452 - The IOB QX<111> is either not constrained (LOC)
   to a specific location and/or has an undefined I/O Standard (IOSTANDARD).
   This condition may seriously affect the device and will be an error in
   bitstream creation. It should be corrected by properly specifying the pin
   location and I/O Standard.
WARNING:PhysDesignRules:2452 - The IOB QX<114> is either not constrained (LOC)
   to a specific location and/or has an undefined I/O Standard (IOSTANDARD).
   This condition may seriously affect the device and will be an error in
   bitstream creation. It should be corrected by properly specifying the pin
   location and I/O Standard.
WARNING:PhysDesignRules:2452 - The IOB QX<113> is either not constrained (LOC)
   to a specific location and/or has an undefined I/O Standard (IOSTANDARD).
   This condition may seriously affect the device and will be an error in
   bitstream creation. It should be corrected by properly specifying the pin
   location and I/O Standard.
WARNING:PhysDesignRules:2452 - The IOB QX<110> is either not constrained (LOC)
   to a specific location and/or has an undefined I/O Standard (IOSTANDARD).
   This condition may seriously affect the device and will be an error in
   bitstream creation. It should be corrected by properly specifying the pin
   location and I/O Standard.
WARNING:PhysDesignRules:2452 - The IOB QX<119> is either not constrained (LOC)
   to a specific location and/or has an undefined I/O Standard (IOSTANDARD).
   This condition may seriously affect the device and will be an error in
   bitstream creation. It should be corrected by properly specifying the pin
   location and I/O Standard.
WARNING:PhysDesignRules:2452 - The IOB QX<116> is either not constrained (LOC)
   to a specific location and/or has an undefined I/O Standard (IOSTANDARD).
   This condition may seriously affect the device and will be an error in
   bitstream creation. It should be corrected by properly specifying the pin
   location and I/O Standard.
WARNING:PhysDesignRules:2452 - The IOB QX<115> is either not constrained (LOC)
   to a specific location and/or has an undefined I/O Standard (IOSTANDARD).
   This condition may seriously affect the device and will be an error in
   bitstream creation. It should be corrected by properly specifying the pin
   location and I/O Standard.
WARNING:PhysDesignRules:2452 - The IOB QX<118> is either not constrained (LOC)
   to a specific location and/or has an undefined I/O Standard (IOSTANDARD).
   This condition may seriously affect the device and will be an error in
   bitstream creation. It should be corrected by properly specifying the pin
   location and I/O Standard.
WARNING:PhysDesignRules:2452 - The IOB QX<117> is either not constrained (LOC)
   to a specific location and/or has an undefined I/O Standard (IOSTANDARD).
   This condition may seriously affect the device and will be an error in
   bitstream creation. It should be corrected by properly specifying the pin
   location and I/O Standard.
WARNING:PhysDesignRules:2452 - The IOB QX<122> is either not constrained (LOC)
   to a specific location and/or has an undefined I/O Standard (IOSTANDARD).
   This condition may seriously affect the device and will be an error in
   bitstream creation. It should be corrected by properly specifying the pin
   location and I/O Standard.
WARNING:PhysDesignRules:2452 - The IOB QX<121> is either not constrained (LOC)
   to a specific location and/or has an undefined I/O Standard (IOSTANDARD).
   This condition may seriously affect the device and will be an error in
   bitstream creation. It should be corrected by properly specifying the pin
   location and I/O Standard.
WARNING:PhysDesignRules:2452 - The IOB QX<124> is either not constrained (LOC)
   to a specific location and/or has an undefined I/O Standard (IOSTANDARD).
```

```
   This condition may seriously affect the device and will be an error in
   bitstream creation. It should be corrected by properly specifying the pin
   location and I/O Standard.
WARNING:PhysDesignRules:2452 - The IOB QX<123> is either not constrained (LOC)
   to a specific location and/or has an undefined I/O Standard (IOSTANDARD).
   This condition may seriously affect the device and will be an error in
   bitstream creation. It should be corrected by properly specifying the pin
   location and I/O Standard.
WARNING:PhysDesignRules:2452 - The IOB QX<120> is either not constrained (LOC)
   to a specific location and/or has an undefined I/O Standard (IOSTANDARD).
   This condition may seriously affect the device and will be an error in
   bitstream creation. It should be corrected by properly specifying the pin
   location and I/O Standard.
WARNING:PhysDesignRules:2452 - The IOB QX<129> is either not constrained (LOC)
   to a specific location and/or has an undefined I/O Standard (IOSTANDARD).
   This condition may seriously affect the device and will be an error in
   bitstream creation. It should be corrected by properly specifying the pin
   location and I/O Standard.
WARNING:PhysDesignRules:2452 - The IOB QX<126> is either not constrained (LOC)
   to a specific location and/or has an undefined I/O Standard (IOSTANDARD).
   This condition may seriously affect the device and will be an error in
   bitstream creation. It should be corrected by properly specifying the pin
   location and I/O Standard.
WARNING:PhysDesignRules:2452 - The IOB QX<125> is either not constrained (LOC)
   to a specific location and/or has an undefined I/O Standard (IOSTANDARD).
   This condition may seriously affect the device and will be an error in
   bitstream creation. It should be corrected by properly specifying the pin
   location and I/O Standard.
WARNING:PhysDesignRules:2452 - The IOB QX<128> is either not constrained (LOC)
   to a specific location and/or has an undefined I/O Standard (IOSTANDARD).
   This condition may seriously affect the device and will be an error in
   bitstream creation. It should be corrected by properly specifying the pin
   location and I/O Standard.
WARNING:PhysDesignRules:2452 - The IOB QX<127> is either not constrained (LOC)
   to a specific location and/or has an undefined I/O Standard (IOSTANDARD).
   This condition may seriously affect the device and will be an error in
   bitstream creation. It should be corrected by properly specifying the pin
   location and I/O Standard.
WARNING:PhysDesignRules:2452 - The IOB QX<132> is either not constrained (LOC)
   to a specific location and/or has an undefined I/O Standard (IOSTANDARD).
   This condition may seriously affect the device and will be an error in
   bitstream creation. It should be corrected by properly specifying the pin
   location and I/O Standard.
WARNING:PhysDesignRules:2452 - The IOB QX<131> is either not constrained (LOC)
   to a specific location and/or has an undefined I/O Standard (IOSTANDARD).
   This condition may seriously affect the device and will be an error in
   bitstream creation. It should be corrected by properly specifying the pin
   location and I/O Standard.
WARNING:PhysDesignRules:2452 - The IOB QX<134> is either not constrained (LOC)
   to a specific location and/or has an undefined I/O Standard (IOSTANDARD).
   This condition may seriously affect the device and will be an error in
   bitstream creation. It should be corrected by properly specifying the pin
   location and I/O Standard.
WARNING:PhysDesignRules:2452 - The IOB QX<133> is either not constrained (LOC)
   to a specific location and/or has an undefined I/O Standard (IOSTANDARD).
   This condition may seriously affect the device and will be an error in
   bitstream creation. It should be corrected by properly specifying the pin
   location and I/O Standard.
WARNING:PhysDesignRules:2452 - The IOB QX<130> is either not constrained (LOC)
   to a specific location and/or has an undefined I/O Standard (IOSTANDARD).
   This condition may seriously affect the device and will be an error in
   bitstream creation. It should be corrected by properly specifying the pin
   location and I/O Standard.
WARNING:PhysDesignRules:2452 - The IOB QX<139> is either not constrained (LOC)
   to a specific location and/or has an undefined I/O Standard (IOSTANDARD).
   This condition may seriously affect the device and will be an error in
   bitstream creation. It should be corrected by properly specifying the pin
   location and I/O Standard.
WARNING:PhysDesignRules:2452 - The IOB QX<136> is either not constrained (LOC)
   to a specific location and/or has an undefined I/O Standard (IOSTANDARD).
   This condition may seriously affect the device and will be an error in
```

```
   bitstream creation. It should be corrected by properly specifying the pin
   location and I/O Standard.
WARNING:PhysDesignRules:2452 - The IOB QX<135> is either not constrained (LOC)
   to a specific location and/or has an undefined I/O Standard (IOSTANDARD).
   This condition may seriously affect the device and will be an error in
   bitstream creation. It should be corrected by properly specifying the pin
   location and I/O Standard.
WARNING:PhysDesignRules:2452 - The IOB QX<138> is either not constrained (LOC)
   to a specific location and/or has an undefined I/O Standard (IOSTANDARD).
   This condition may seriously affect the device and will be an error in
   bitstream creation. It should be corrected by properly specifying the pin
   location and I/O Standard.
WARNING:PhysDesignRules:2452 - The IOB QX<137> is either not constrained (LOC)
   to a specific location and/or has an undefined I/O Standard (IOSTANDARD).
   This condition may seriously affect the device and will be an error in
   bitstream creation. It should be corrected by properly specifying the pin
   location and I/O Standard.
WARNING:PhysDesignRules:2452 - The IOB QX<142> is either not constrained (LOC)
   to a specific location and/or has an undefined I/O Standard (IOSTANDARD).
   This condition may seriously affect the device and will be an error in
   bitstream creation. It should be corrected by properly specifying the pin
   location and I/O Standard.
WARNING:PhysDesignRules:2452 - The IOB QX<141> is either not constrained (LOC)
   to a specific location and/or has an undefined I/O Standard (IOSTANDARD).
   This condition may seriously affect the device and will be an error in
   bitstream creation. It should be corrected by properly specifying the pin
   location and I/O Standard.
WARNING:PhysDesignRules:2452 - The IOB QX<144> is either not constrained (LOC)
   to a specific location and/or has an undefined I/O Standard (IOSTANDARD).
   This condition may seriously affect the device and will be an error in
   bitstream creation. It should be corrected by properly specifying the pin
   location and I/O Standard.
WARNING:PhysDesignRules:2452 - The IOB QX<143> is either not constrained (LOC)
   to a specific location and/or has an undefined I/O Standard (IOSTANDARD).
   This condition may seriously affect the device and will be an error in
   bitstream creation. It should be corrected by properly specifying the pin
   location and I/O Standard.
WARNING:PhysDesignRules:2452 - The IOB QX<140> is either not constrained (LOC)
   to a specific location and/or has an undefined I/O Standard (IOSTANDARD).
   This condition may seriously affect the device and will be an error in
   bitstream creation. It should be corrected by properly specifying the pin
   location and I/O Standard.
WARNING:PhysDesignRules:2452 - The IOB QX<149> is either not constrained (LOC)
   to a specific location and/or has an undefined I/O Standard (IOSTANDARD).
   This condition may seriously affect the device and will be an error in
   bitstream creation. It should be corrected by properly specifying the pin
   location and I/O Standard.
WARNING:PhysDesignRules:2452 - The IOB QZ<202> is either not constrained (LOC)
   to a specific location and/or has an undefined I/O Standard (IOSTANDARD).
   This condition may seriously affect the device and will be an error in
   bitstream creation. It should be corrected by properly specifying the pin
   location and I/O Standard.
WARNING:PhysDesignRules:2452 - The IOB QZ<201> is either not constrained (LOC)
   to a specific location and/or has an undefined I/O Standard (IOSTANDARD).
   This condition may seriously affect the device and will be an error in
   bitstream creation. It should be corrected by properly specifying the pin
   location and I/O Standard.
WARNING:PhysDesignRules:2452 - The IOB QX<146> is either not constrained (LOC)
   to a specific location and/or has an undefined I/O Standard (IOSTANDARD).
   This condition may seriously affect the device and will be an error in
   bitstream creation. It should be corrected by properly specifying the pin
   location and I/O Standard.
WARNING:PhysDesignRules:2452 - The IOB QZ<204> is either not constrained (LOC)
   to a specific location and/or has an undefined I/O Standard (IOSTANDARD).
   This condition may seriously affect the device and will be an error in
   bitstream creation. It should be corrected by properly specifying the pin
   location and I/O Standard.
WARNING:PhysDesignRules:2452 - The IOB QX<145> is either not constrained (LOC)
   to a specific location and/or has an undefined I/O Standard (IOSTANDARD).
   This condition may seriously affect the device and will be an error in
   bitstream creation. It should be corrected by properly specifying the pin
```

```
   location and I/O Standard.
WARNING:PhysDesignRules:2452 - The IOB QZ<203> is either not constrained (LOC)
   to a specific location and/or has an undefined I/O Standard (IOSTANDARD).
   This condition may seriously affect the device and will be an error in
   bitstream creation. It should be corrected by properly specifying the pin
   location and I/O Standard.
WARNING:PhysDesignRules:2452 - The IOB QX<148> is either not constrained (LOC)
   to a specific location and/or has an undefined I/O Standard (IOSTANDARD).
   This condition may seriously affect the device and will be an error in
   bitstream creation. It should be corrected by properly specifying the pin
   location and I/O Standard.
WARNING:PhysDesignRules:2452 - The IOB QX<147> is either not constrained (LOC)
   to a specific location and/or has an undefined I/O Standard (IOSTANDARD).
   This condition may seriously affect the device and will be an error in
   bitstream creation. It should be corrected by properly specifying the pin
   location and I/O Standard.
WARNING:PhysDesignRules:2452 - The IOB QX<152> is either not constrained (LOC)
   to a specific location and/or has an undefined I/O Standard (IOSTANDARD).
   This condition may seriously affect the device and will be an error in
   bitstream creation. It should be corrected by properly specifying the pin
   location and I/O Standard.
WARNING:PhysDesignRules:2452 - The IOB QZ<200> is either not constrained (LOC)
   to a specific location and/or has an undefined I/O Standard (IOSTANDARD).
   This condition may seriously affect the device and will be an error in
   bitstream creation. It should be corrected by properly specifying the pin
   location and I/O Standard.
WARNING:PhysDesignRules:2452 - The IOB QX<151> is either not constrained (LOC)
   to a specific location and/or has an undefined I/O Standard (IOSTANDARD).
   This condition may seriously affect the device and will be an error in
   bitstream creation. It should be corrected by properly specifying the pin
   location and I/O Standard.
WARNING:PhysDesignRules:2452 - The IOB QX<154> is either not constrained (LOC)
   to a specific location and/or has an undefined I/O Standard (IOSTANDARD).
   This condition may seriously affect the device and will be an error in
   bitstream creation. It should be corrected by properly specifying the pin
   location and I/O Standard.
WARNING:PhysDesignRules:2452 - The IOB QX<153> is either not constrained (LOC)
   to a specific location and/or has an undefined I/O Standard (IOSTANDARD).
   This condition may seriously affect the device and will be an error in
   bitstream creation. It should be corrected by properly specifying the pin
   location and I/O Standard.
WARNING:PhysDesignRules:2452 - The IOB QZ<209> is either not constrained (LOC)
   to a specific location and/or has an undefined I/O Standard (IOSTANDARD).
   This condition may seriously affect the device and will be an error in
   bitstream creation. It should be corrected by properly specifying the pin
   location and I/O Standard.
WARNING:PhysDesignRules:2452 - The IOB QX<150> is either not constrained (LOC)
   to a specific location and/or has an undefined I/O Standard (IOSTANDARD).
   This condition may seriously affect the device and will be an error in
   bitstream creation. It should be corrected by properly specifying the pin
   location and I/O Standard.
WARNING:PhysDesignRules:2452 - The IOB QZ<206> is either not constrained (LOC)
   to a specific location and/or has an undefined I/O Standard (IOSTANDARD).
   This condition may seriously affect the device and will be an error in
   bitstream creation. It should be corrected by properly specifying the pin
   location and I/O Standard.
WARNING:PhysDesignRules:2452 - The IOB QZ<205> is either not constrained (LOC)
   to a specific location and/or has an undefined I/O Standard (IOSTANDARD).
   This condition may seriously affect the device and will be an error in
   bitstream creation. It should be corrected by properly specifying the pin
   location and I/O Standard.
WARNING:PhysDesignRules:2452 - The IOB QZ<208> is either not constrained (LOC)
   to a specific location and/or has an undefined I/O Standard (IOSTANDARD).
   This condition may seriously affect the device and will be an error in
   bitstream creation. It should be corrected by properly specifying the pin
   location and I/O Standard.
WARNING:PhysDesignRules:2452 - The IOB QX<159> is either not constrained (LOC)
   to a specific location and/or has an undefined I/O Standard (IOSTANDARD).
   This condition may seriously affect the device and will be an error in
   bitstream creation. It should be corrected by properly specifying the pin
   location and I/O Standard.
```

```
WARNING:PhysDesignRules:2452 - The IOB QZ<207> is either not constrained (LOC)
   to a specific location and/or has an undefined I/O Standard (IOSTANDARD).
   This condition may seriously affect the device and will be an error in
   bitstream creation. It should be corrected by properly specifying the pin
   location and I/O Standard.
WARNING:PhysDesignRules:2452 - The IOB QZ<212> is either not constrained (LOC)
   to a specific location and/or has an undefined I/O Standard (IOSTANDARD).
   This condition may seriously affect the device and will be an error in
   bitstream creation. It should be corrected by properly specifying the pin
   location and I/O Standard.
WARNING:PhysDesignRules:2452 - The IOB QZ<211> is either not constrained (LOC)
   to a specific location and/or has an undefined I/O Standard (IOSTANDARD).
   This condition may seriously affect the device and will be an error in
   bitstream creation. It should be corrected by properly specifying the pin
   location and I/O Standard.
WARNING:PhysDesignRules:2452 - The IOB QX<156> is either not constrained (LOC)
   to a specific location and/or has an undefined I/O Standard (IOSTANDARD).
   This condition may seriously affect the device and will be an error in
   bitstream creation. It should be corrected by properly specifying the pin
   location and I/O Standard.
WARNING:PhysDesignRules:2452 - The IOB QZ<214> is either not constrained (LOC)
   to a specific location and/or has an undefined I/O Standard (IOSTANDARD).
   This condition may seriously affect the device and will be an error in
   bitstream creation. It should be corrected by properly specifying the pin
   location and I/O Standard.
WARNING:PhysDesignRules:2452 - The IOB QX<155> is either not constrained (LOC)
   to a specific location and/or has an undefined I/O Standard (IOSTANDARD).
   This condition may seriously affect the device and will be an error in
   bitstream creation. It should be corrected by properly specifying the pin
   location and I/O Standard.
WARNING:PhysDesignRules:2452 - The IOB QZ<213> is either not constrained (LOC)
   to a specific location and/or has an undefined I/O Standard (IOSTANDARD).
   This condition may seriously affect the device and will be an error in
   bitstream creation. It should be corrected by properly specifying the pin
   location and I/O Standard.
WARNING:PhysDesignRules:2452 - The IOB QX<158> is either not constrained (LOC)
   to a specific location and/or has an undefined I/O Standard (IOSTANDARD).
   This condition may seriously affect the device and will be an error in
   bitstream creation. It should be corrected by properly specifying the pin
   location and I/O Standard.
WARNING:PhysDesignRules:2452 - The IOB QX<157> is either not constrained (LOC)
   to a specific location and/or has an undefined I/O Standard (IOSTANDARD).
   This condition may seriously affect the device and will be an error in
   bitstream creation. It should be corrected by properly specifying the pin
   location and I/O Standard.
WARNING:PhysDesignRules:2452 - The IOB QX<162> is either not constrained (LOC)
   to a specific location and/or has an undefined I/O Standard (IOSTANDARD).
   This condition may seriously affect the device and will be an error in
   bitstream creation. It should be corrected by properly specifying the pin
   location and I/O Standard.
WARNING:PhysDesignRules:2452 - The IOB QZ<210> is either not constrained (LOC)
   to a specific location and/or has an undefined I/O Standard (IOSTANDARD).
   This condition may seriously affect the device and will be an error in
   bitstream creation. It should be corrected by properly specifying the pin
   location and I/O Standard.
WARNING:PhysDesignRules:2452 - The IOB QX<161> is either not constrained (LOC)
   to a specific location and/or has an undefined I/O Standard (IOSTANDARD).
   This condition may seriously affect the device and will be an error in
   bitstream creation. It should be corrected by properly specifying the pin
   location and I/O Standard.
WARNING:PhysDesignRules:2452 - The IOB QX<164> is either not constrained (LOC)
   to a specific location and/or has an undefined I/O Standard (IOSTANDARD).
   This condition may seriously affect the device and will be an error in
   bitstream creation. It should be corrected by properly specifying the pin
   location and I/O Standard.
WARNING:PhysDesignRules:2452 - The IOB QX<163> is either not constrained (LOC)
   to a specific location and/or has an undefined I/O Standard (IOSTANDARD).
   This condition may seriously affect the device and will be an error in
   bitstream creation. It should be corrected by properly specifying the pin
   location and I/O Standard.
WARNING:PhysDesignRules:2452 - The IOB QZ<219> is either not constrained (LOC)
```

```
   to a specific location and/or has an undefined I/O Standard (IOSTANDARD).
   This condition may seriously affect the device and will be an error in
   bitstream creation. It should be corrected by properly specifying the pin
   location and I/O Standard.
WARNING:PhysDesignRules:2452 - The IOB QX<160> is either not constrained (LOC)
   to a specific location and/or has an undefined I/O Standard (IOSTANDARD).
   This condition may seriously affect the device and will be an error in
   bitstream creation. It should be corrected by properly specifying the pin
   location and I/O Standard.
WARNING:PhysDesignRules:2452 - The IOB QZ<216> is either not constrained (LOC)
   to a specific location and/or has an undefined I/O Standard (IOSTANDARD).
   This condition may seriously affect the device and will be an error in
   bitstream creation. It should be corrected by properly specifying the pin
   location and I/O Standard.
WARNING:PhysDesignRules:2452 - The IOB QZ<215> is either not constrained (LOC)
   to a specific location and/or has an undefined I/O Standard (IOSTANDARD).
   This condition may seriously affect the device and will be an error in
   bitstream creation. It should be corrected by properly specifying the pin
   location and I/O Standard.
WARNING:PhysDesignRules:2452 - The IOB QZ<218> is either not constrained (LOC)
   to a specific location and/or has an undefined I/O Standard (IOSTANDARD).
   This condition may seriously affect the device and will be an error in
   bitstream creation. It should be corrected by properly specifying the pin
   location and I/O Standard.
WARNING:PhysDesignRules:2452 - The IOB QX<169> is either not constrained (LOC)
   to a specific location and/or has an undefined I/O Standard (IOSTANDARD).
   This condition may seriously affect the device and will be an error in
   bitstream creation. It should be corrected by properly specifying the pin
   location and I/O Standard.
WARNING:PhysDesignRules:2452 - The IOB QZ<217> is either not constrained (LOC)
   to a specific location and/or has an undefined I/O Standard (IOSTANDARD).
   This condition may seriously affect the device and will be an error in
   bitstream creation. It should be corrected by properly specifying the pin
   location and I/O Standard.
WARNING:PhysDesignRules:2452 - The IOB QZ<222> is either not constrained (LOC)
   to a specific location and/or has an undefined I/O Standard (IOSTANDARD).
   This condition may seriously affect the device and will be an error in
   bitstream creation. It should be corrected by properly specifying the pin
   location and I/O Standard.
WARNING:PhysDesignRules:2452 - The IOB QZ<221> is either not constrained (LOC)
   to a specific location and/or has an undefined I/O Standard (IOSTANDARD).
   This condition may seriously affect the device and will be an error in
   bitstream creation. It should be corrected by properly specifying the pin
   location and I/O Standard.
WARNING:PhysDesignRules:2452 - The IOB QX<166> is either not constrained (LOC)
   to a specific location and/or has an undefined I/O Standard (IOSTANDARD).
   This condition may seriously affect the device and will be an error in
   bitstream creation. It should be corrected by properly specifying the pin
   location and I/O Standard.
WARNING:PhysDesignRules:2452 - The IOB QZ<224> is either not constrained (LOC)
   to a specific location and/or has an undefined I/O Standard (IOSTANDARD).
   This condition may seriously affect the device and will be an error in
   bitstream creation. It should be corrected by properly specifying the pin
   location and I/O Standard.
WARNING:PhysDesignRules:2452 - The IOB QX<165> is either not constrained (LOC)
   to a specific location and/or has an undefined I/O Standard (IOSTANDARD).
   This condition may seriously affect the device and will be an error in
   bitstream creation. It should be corrected by properly specifying the pin
   location and I/O Standard.
WARNING:PhysDesignRules:2452 - The IOB QZ<223> is either not constrained (LOC)
   to a specific location and/or has an undefined I/O Standard (IOSTANDARD).
   This condition may seriously affect the device and will be an error in
   bitstream creation. It should be corrected by properly specifying the pin
   location and I/O Standard.
WARNING:PhysDesignRules:2452 - The IOB QX<168> is either not constrained (LOC)
   to a specific location and/or has an undefined I/O Standard (IOSTANDARD).
   This condition may seriously affect the device and will be an error in
   bitstream creation. It should be corrected by properly specifying the pin
   location and I/O Standard.
WARNING:PhysDesignRules:2452 - The IOB QX<167> is either not constrained (LOC)
   to a specific location and/or has an undefined I/O Standard (IOSTANDARD).
```

```
   This condition may seriously affect the device and will be an error in
   bitstream creation. It should be corrected by properly specifying the pin
   location and I/O Standard.
WARNING:PhysDesignRules:2452 - The IOB QX<172> is either not constrained (LOC)
   to a specific location and/or has an undefined I/O Standard (IOSTANDARD).
   This condition may seriously affect the device and will be an error in
   bitstream creation. It should be corrected by properly specifying the pin
   location and I/O Standard.
WARNING:PhysDesignRules:2452 - The IOB QZ<220> is either not constrained (LOC)
   to a specific location and/or has an undefined I/O Standard (IOSTANDARD).
   This condition may seriously affect the device and will be an error in
   bitstream creation. It should be corrected by properly specifying the pin
   location and I/O Standard.
WARNING:PhysDesignRules:2452 - The IOB QX<171> is either not constrained (LOC)
   to a specific location and/or has an undefined I/O Standard (IOSTANDARD).
   This condition may seriously affect the device and will be an error in
   bitstream creation. It should be corrected by properly specifying the pin
   location and I/O Standard.
WARNING:PhysDesignRules:2452 - The IOB QX<174> is either not constrained (LOC)
   to a specific location and/or has an undefined I/O Standard (IOSTANDARD).
   This condition may seriously affect the device and will be an error in
   bitstream creation. It should be corrected by properly specifying the pin
   location and I/O Standard.
WARNING:PhysDesignRules:2452 - The IOB QX<173> is either not constrained (LOC)
   to a specific location and/or has an undefined I/O Standard (IOSTANDARD).
   This condition may seriously affect the device and will be an error in
   bitstream creation. It should be corrected by properly specifying the pin
   location and I/O Standard.
WARNING:PhysDesignRules:2452 - The IOB QZ<229> is either not constrained (LOC)
   to a specific location and/or has an undefined I/O Standard (IOSTANDARD).
   This condition may seriously affect the device and will be an error in
   bitstream creation. It should be corrected by properly specifying the pin
   location and I/O Standard.
WARNING:PhysDesignRules:2452 - The IOB QX<170> is either not constrained (LOC)
   to a specific location and/or has an undefined I/O Standard (IOSTANDARD).
   This condition may seriously affect the device and will be an error in
   bitstream creation. It should be corrected by properly specifying the pin
   location and I/O Standard.
WARNING:PhysDesignRules:2452 - The IOB QZ<226> is either not constrained (LOC)
   to a specific location and/or has an undefined I/O Standard (IOSTANDARD).
   This condition may seriously affect the device and will be an error in
   bitstream creation. It should be corrected by properly specifying the pin
   location and I/O Standard.
WARNING:PhysDesignRules:2452 - The IOB QZ<225> is either not constrained (LOC)
   to a specific location and/or has an undefined I/O Standard (IOSTANDARD).
   This condition may seriously affect the device and will be an error in
   bitstream creation. It should be corrected by properly specifying the pin
   location and I/O Standard.
WARNING:PhysDesignRules:2452 - The IOB QZ<228> is either not constrained (LOC)
   to a specific location and/or has an undefined I/O Standard (IOSTANDARD).
   This condition may seriously affect the device and will be an error in
   bitstream creation. It should be corrected by properly specifying the pin
   location and I/O Standard.
WARNING:PhysDesignRules:2452 - The IOB QX<179> is either not constrained (LOC)
   to a specific location and/or has an undefined I/O Standard (IOSTANDARD).
   This condition may seriously affect the device and will be an error in
   bitstream creation. It should be corrected by properly specifying the pin
   location and I/O Standard.
WARNING:PhysDesignRules:2452 - The IOB QZ<227> is either not constrained (LOC)
   to a specific location and/or has an undefined I/O Standard (IOSTANDARD).
   This condition may seriously affect the device and will be an error in
   bitstream creation. It should be corrected by properly specifying the pin
   location and I/O Standard.
WARNING:PhysDesignRules:2452 - The IOB QZ<232> is either not constrained (LOC)
   to a specific location and/or has an undefined I/O Standard (IOSTANDARD).
   This condition may seriously affect the device and will be an error in
   bitstream creation. It should be corrected by properly specifying the pin
   location and I/O Standard.
WARNING:PhysDesignRules:2452 - The IOB QZ<231> is either not constrained (LOC)
   to a specific location and/or has an undefined I/O Standard (IOSTANDARD).
   This condition may seriously affect the device and will be an error in
```

```
   bitstream creation. It should be corrected by properly specifying the pin
   location and I/O Standard.
WARNING:PhysDesignRules:2452 - The IOB QX<176> is either not constrained (LOC)
   to a specific location and/or has an undefined I/O Standard (IOSTANDARD).
   This condition may seriously affect the device and will be an error in
   bitstream creation. It should be corrected by properly specifying the pin
   location and I/O Standard.
WARNING:PhysDesignRules:2452 - The IOB QX<175> is either not constrained (LOC)
   to a specific location and/or has an undefined I/O Standard (IOSTANDARD).
   This condition may seriously affect the device and will be an error in
   bitstream creation. It should be corrected by properly specifying the pin
   location and I/O Standard.
WARNING:PhysDesignRules:2452 - The IOB QX<178> is either not constrained (LOC)
   to a specific location and/or has an undefined I/O Standard (IOSTANDARD).
   This condition may seriously affect the device and will be an error in
   bitstream creation. It should be corrected by properly specifying the pin
   location and I/O Standard.
WARNING:PhysDesignRules:2452 - The IOB QX<177> is either not constrained (LOC)
   to a specific location and/or has an undefined I/O Standard (IOSTANDARD).
   This condition may seriously affect the device and will be an error in
   bitstream creation. It should be corrected by properly specifying the pin
   location and I/O Standard.
WARNING:PhysDesignRules:2452 - The IOB QX<182> is either not constrained (LOC)
   to a specific location and/or has an undefined I/O Standard (IOSTANDARD).
   This condition may seriously affect the device and will be an error in
   bitstream creation. It should be corrected by properly specifying the pin
   location and I/O Standard.
WARNING:PhysDesignRules:2452 - The IOB QZ<230> is either not constrained (LOC)
   to a specific location and/or has an undefined I/O Standard (IOSTANDARD).
   This condition may seriously affect the device and will be an error in
   bitstream creation. It should be corrected by properly specifying the pin
   location and I/O Standard.
WARNING:PhysDesignRules:2452 - The IOB QX<181> is either not constrained (LOC)
   to a specific location and/or has an undefined I/O Standard (IOSTANDARD).
   This condition may seriously affect the device and will be an error in
   bitstream creation. It should be corrected by properly specifying the pin
   location and I/O Standard.
WARNING:PhysDesignRules:2452 - The IOB QX<184> is either not constrained (LOC)
   to a specific location and/or has an undefined I/O Standard (IOSTANDARD).
   This condition may seriously affect the device and will be an error in
   bitstream creation. It should be corrected by properly specifying the pin
   location and I/O Standard.
WARNING:PhysDesignRules:2452 - The IOB QX<183> is either not constrained (LOC)
   to a specific location and/or has an undefined I/O Standard (IOSTANDARD).
   This condition may seriously affect the device and will be an error in
   bitstream creation. It should be corrected by properly specifying the pin
   location and I/O Standard.
WARNING:PhysDesignRules:2452 - The IOB QX<180> is either not constrained (LOC)
   to a specific location and/or has an undefined I/O Standard (IOSTANDARD).
   This condition may seriously affect the device and will be an error in
   bitstream creation. It should be corrected by properly specifying the pin
   location and I/O Standard.
WARNING:PhysDesignRules:2452 - The IOB QX<189> is either not constrained (LOC)
   to a specific location and/or has an undefined I/O Standard (IOSTANDARD).
   This condition may seriously affect the device and will be an error in
   bitstream creation. It should be corrected by properly specifying the pin
   location and I/O Standard.
WARNING:PhysDesignRules:2452 - The IOB QX<186> is either not constrained (LOC)
   to a specific location and/or has an undefined I/O Standard (IOSTANDARD).
   This condition may seriously affect the device and will be an error in
   bitstream creation. It should be corrected by properly specifying the pin
   location and I/O Standard.
WARNING:PhysDesignRules:2452 - The IOB QX<185> is either not constrained (LOC)
   to a specific location and/or has an undefined I/O Standard (IOSTANDARD).
   This condition may seriously affect the device and will be an error in
   bitstream creation. It should be corrected by properly specifying the pin
   location and I/O Standard.
WARNING:PhysDesignRules:2452 - The IOB QX<188> is either not constrained (LOC)
   to a specific location and/or has an undefined I/O Standard (IOSTANDARD).
   This condition may seriously affect the device and will be an error in
   bitstream creation. It should be corrected by properly specifying the pin
```

```
   location and I/O Standard.
WARNING:PhysDesignRules:2452 - The IOB QX<187> is either not constrained (LOC)
   to a specific location and/or has an undefined I/O Standard (IOSTANDARD).
   This condition may seriously affect the device and will be an error in
   bitstream creation. It should be corrected by properly specifying the pin
   location and I/O Standard.
WARNING:PhysDesignRules:2452 - The IOB QX<192> is either not constrained (LOC)
   to a specific location and/or has an undefined I/O Standard (IOSTANDARD).
   This condition may seriously affect the device and will be an error in
   bitstream creation. It should be corrected by properly specifying the pin
   location and I/O Standard.
WARNING:PhysDesignRules:2452 - The IOB QX<191> is either not constrained (LOC)
   to a specific location and/or has an undefined I/O Standard (IOSTANDARD).
   This condition may seriously affect the device and will be an error in
   bitstream creation. It should be corrected by properly specifying the pin
   location and I/O Standard.
WARNING:PhysDesignRules:2452 - The IOB QX<194> is either not constrained (LOC)
   to a specific location and/or has an undefined I/O Standard (IOSTANDARD).
   This condition may seriously affect the device and will be an error in
   bitstream creation. It should be corrected by properly specifying the pin
   location and I/O Standard.
WARNING:PhysDesignRules:2452 - The IOB QX<193> is either not constrained (LOC)
   to a specific location and/or has an undefined I/O Standard (IOSTANDARD).
   This condition may seriously affect the device and will be an error in
   bitstream creation. It should be corrected by properly specifying the pin
   location and I/O Standard.
WARNING:PhysDesignRules:2452 - The IOB QX<190> is either not constrained (LOC)
   to a specific location and/or has an undefined I/O Standard (IOSTANDARD).
   This condition may seriously affect the device and will be an error in
   bitstream creation. It should be corrected by properly specifying the pin
   location and I/O Standard.
WARNING:PhysDesignRules:2452 - The IOB QX<199> is either not constrained (LOC)
   to a specific location and/or has an undefined I/O Standard (IOSTANDARD).
   This condition may seriously affect the device and will be an error in
   bitstream creation. It should be corrected by properly specifying the pin
   location and I/O Standard.
WARNING:PhysDesignRules:2452 - The IOB QX<196> is either not constrained (LOC)
   to a specific location and/or has an undefined I/O Standard (IOSTANDARD).
   This condition may seriously affect the device and will be an error in
   bitstream creation. It should be corrected by properly specifying the pin
   location and I/O Standard.
WARNING:PhysDesignRules:2452 - The IOB QX<195> is either not constrained (LOC)
   to a specific location and/or has an undefined I/O Standard (IOSTANDARD).
   This condition may seriously affect the device and will be an error in
   bitstream creation. It should be corrected by properly specifying the pin
   location and I/O Standard.
WARNING:PhysDesignRules:2452 - The IOB QX<198> is either not constrained (LOC)
   to a specific location and/or has an undefined I/O Standard (IOSTANDARD).
   This condition may seriously affect the device and will be an error in
   bitstream creation. It should be corrected by properly specifying the pin
   location and I/O Standard.
WARNING:PhysDesignRules:2452 - The IOB QX<197> is either not constrained (LOC)
   to a specific location and/or has an undefined I/O Standard (IOSTANDARD).
   This condition may seriously affect the device and will be an error in
   bitstream creation. It should be corrected by properly specifying the pin
   location and I/O Standard.
WARNING:PhysDesignRules:2452 - The IOB QX<202> is either not constrained (LOC)
   to a specific location and/or has an undefined I/O Standard (IOSTANDARD).
   This condition may seriously affect the device and will be an error in
   bitstream creation. It should be corrected by properly specifying the pin
   location and I/O Standard.
WARNING:PhysDesignRules:2452 - The IOB QX<201> is either not constrained (LOC)
   to a specific location and/or has an undefined I/O Standard (IOSTANDARD).
   This condition may seriously affect the device and will be an error in
   bitstream creation. It should be corrected by properly specifying the pin
   location and I/O Standard.
WARNING:PhysDesignRules:2452 - The IOB QX<204> is either not constrained (LOC)
   to a specific location and/or has an undefined I/O Standard (IOSTANDARD).
   This condition may seriously affect the device and will be an error in
   bitstream creation. It should be corrected by properly specifying the pin
   location and I/O Standard.
```

```
WARNING:PhysDesignRules:2452 - The IOB QX<203> is either not constrained (LOC)
   to a specific location and/or has an undefined I/O Standard (IOSTANDARD).
   This condition may seriously affect the device and will be an error in
   bitstream creation. It should be corrected by properly specifying the pin
   location and I/O Standard.
WARNING:PhysDesignRules:2452 - The IOB QX<200> is either not constrained (LOC)
   to a specific location and/or has an undefined I/O Standard (IOSTANDARD).
   This condition may seriously affect the device and will be an error in
   bitstream creation. It should be corrected by properly specifying the pin
   location and I/O Standard.
WARNING:PhysDesignRules:2452 - The IOB QX<209> is either not constrained (LOC)
   to a specific location and/or has an undefined I/O Standard (IOSTANDARD).
   This condition may seriously affect the device and will be an error in
   bitstream creation. It should be corrected by properly specifying the pin
   location and I/O Standard.
WARNING:PhysDesignRules:2452 - The IOB QX<206> is either not constrained (LOC)
   to a specific location and/or has an undefined I/O Standard (IOSTANDARD).
   This condition may seriously affect the device and will be an error in
   bitstream creation. It should be corrected by properly specifying the pin
   location and I/O Standard.
WARNING:PhysDesignRules:2452 - The IOB QX<205> is either not constrained (LOC)
   to a specific location and/or has an undefined I/O Standard (IOSTANDARD).
   This condition may seriously affect the device and will be an error in
   bitstream creation. It should be corrected by properly specifying the pin
   location and I/O Standard.
WARNING:PhysDesignRules:2452 - The IOB QX<208> is either not constrained (LOC)
   to a specific location and/or has an undefined I/O Standard (IOSTANDARD).
   This condition may seriously affect the device and will be an error in
   bitstream creation. It should be corrected by properly specifying the pin
   location and I/O Standard.
WARNING:PhysDesignRules:2452 - The IOB QX<207> is either not constrained (LOC)
   to a specific location and/or has an undefined I/O Standard (IOSTANDARD).
   This condition may seriously affect the device and will be an error in
   bitstream creation. It should be corrected by properly specifying the pin
   location and I/O Standard.
WARNING:PhysDesignRules:2452 - The IOB QX<212> is either not constrained (LOC)
   to a specific location and/or has an undefined I/O Standard (IOSTANDARD).
   This condition may seriously affect the device and will be an error in
   bitstream creation. It should be corrected by properly specifying the pin
   location and I/O Standard.
WARNING:PhysDesignRules:2452 - The IOB QX<211> is either not constrained (LOC)
   to a specific location and/or has an undefined I/O Standard (IOSTANDARD).
   This condition may seriously affect the device and will be an error in
   bitstream creation. It should be corrected by properly specifying the pin
   location and I/O Standard.
WARNING:PhysDesignRules:2452 - The IOB QX<214> is either not constrained (LOC)
   to a specific location and/or has an undefined I/O Standard (IOSTANDARD).
   This condition may seriously affect the device and will be an error in
   bitstream creation. It should be corrected by properly specifying the pin
   location and I/O Standard.
WARNING:PhysDesignRules:2452 - The IOB QX<213> is either not constrained (LOC)
   to a specific location and/or has an undefined I/O Standard (IOSTANDARD).
   This condition may seriously affect the device and will be an error in
   bitstream creation. It should be corrected by properly specifying the pin
   location and I/O Standard.
WARNING:PhysDesignRules:2452 - The IOB QX<210> is either not constrained (LOC)
   to a specific location and/or has an undefined I/O Standard (IOSTANDARD).
   This condition may seriously affect the device and will be an error in
   bitstream creation. It should be corrected by properly specifying the pin
   location and I/O Standard.
WARNING:PhysDesignRules:2452 - The IOB QX<219> is either not constrained (LOC)
   to a specific location and/or has an undefined I/O Standard (IOSTANDARD).
   This condition may seriously affect the device and will be an error in
   bitstream creation. It should be corrected by properly specifying the pin
   location and I/O Standard.
WARNING:PhysDesignRules:2452 - The IOB QX<216> is either not constrained (LOC)
   to a specific location and/or has an undefined I/O Standard (IOSTANDARD).
   This condition may seriously affect the device and will be an error in
   bitstream creation. It should be corrected by properly specifying the pin
   location and I/O Standard.
WARNING:PhysDesignRules:2452 - The IOB QX<215> is either not constrained (LOC)
```

```
   to a specific location and/or has an undefined I/O Standard (IOSTANDARD).
   This condition may seriously affect the device and will be an error in
   bitstream creation. It should be corrected by properly specifying the pin
   location and I/O Standard.
WARNING:PhysDesignRules:2452 - The IOB QX<218> is either not constrained (LOC)
   to a specific location and/or has an undefined I/O Standard (IOSTANDARD).
   This condition may seriously affect the device and will be an error in
   bitstream creation. It should be corrected by properly specifying the pin
   location and I/O Standard.
WARNING:PhysDesignRules:2452 - The IOB QX<217> is either not constrained (LOC)
   to a specific location and/or has an undefined I/O Standard (IOSTANDARD).
   This condition may seriously affect the device and will be an error in
   bitstream creation. It should be corrected by properly specifying the pin
   location and I/O Standard.
WARNING:PhysDesignRules:2452 - The IOB QX<222> is either not constrained (LOC)
   to a specific location and/or has an undefined I/O Standard (IOSTANDARD).
   This condition may seriously affect the device and will be an error in
   bitstream creation. It should be corrected by properly specifying the pin
   location and I/O Standard.
WARNING:PhysDesignRules:2452 - The IOB QX<221> is either not constrained (LOC)
   to a specific location and/or has an undefined I/O Standard (IOSTANDARD).
   This condition may seriously affect the device and will be an error in
   bitstream creation. It should be corrected by properly specifying the pin
   location and I/O Standard.
WARNING:PhysDesignRules:2452 - The IOB QX<224> is either not constrained (LOC)
   to a specific location and/or has an undefined I/O Standard (IOSTANDARD).
   This condition may seriously affect the device and will be an error in
   bitstream creation. It should be corrected by properly specifying the pin
   location and I/O Standard.
WARNING:PhysDesignRules:2452 - The IOB QX<223> is either not constrained (LOC)
   to a specific location and/or has an undefined I/O Standard (IOSTANDARD).
   This condition may seriously affect the device and will be an error in
   bitstream creation. It should be corrected by properly specifying the pin
   location and I/O Standard.
WARNING:PhysDesignRules:2452 - The IOB QX<220> is either not constrained (LOC)
   to a specific location and/or has an undefined I/O Standard (IOSTANDARD).
   This condition may seriously affect the device and will be an error in
   bitstream creation. It should be corrected by properly specifying the pin
   location and I/O Standard.
WARNING:PhysDesignRules:2452 - The IOB QX<229> is either not constrained (LOC)
   to a specific location and/or has an undefined I/O Standard (IOSTANDARD).
   This condition may seriously affect the device and will be an error in
   bitstream creation. It should be corrected by properly specifying the pin
   location and I/O Standard.
WARNING:PhysDesignRules:2452 - The IOB QX<226> is either not constrained (LOC)
   to a specific location and/or has an undefined I/O Standard (IOSTANDARD).
   This condition may seriously affect the device and will be an error in
   bitstream creation. It should be corrected by properly specifying the pin
   location and I/O Standard.
WARNING:PhysDesignRules:2452 - The IOB QX<225> is either not constrained (LOC)
   to a specific location and/or has an undefined I/O Standard (IOSTANDARD).
   This condition may seriously affect the device and will be an error in
   bitstream creation. It should be corrected by properly specifying the pin
   location and I/O Standard.
WARNING:PhysDesignRules:2452 - The IOB QX<228> is either not constrained (LOC)
   to a specific location and/or has an undefined I/O Standard (IOSTANDARD).
   This condition may seriously affect the device and will be an error in
   bitstream creation. It should be corrected by properly specifying the pin
   location and I/O Standard.
WARNING:PhysDesignRules:2452 - The IOB QX<227> is either not constrained (LOC)
   to a specific location and/or has an undefined I/O Standard (IOSTANDARD).
   This condition may seriously affect the device and will be an error in
   bitstream creation. It should be corrected by properly specifying the pin
   location and I/O Standard.
WARNING:PhysDesignRules:2452 - The IOB QX<232> is either not constrained (LOC)
   to a specific location and/or has an undefined I/O Standard (IOSTANDARD).
   This condition may seriously affect the device and will be an error in
   bitstream creation. It should be corrected by properly specifying the pin
   location and I/O Standard.
WARNING:PhysDesignRules:2452 - The IOB QX<231> is either not constrained (LOC)
   to a specific location and/or has an undefined I/O Standard (IOSTANDARD).
```

```
   This condition may seriously affect the device and will be an error in
   bitstream creation. It should be corrected by properly specifying the pin
   location and I/O Standard.
WARNING:PhysDesignRules:2452 - The IOB QX<230> is either not constrained (LOC)
   to a specific location and/or has an undefined I/O Standard (IOSTANDARD).
   This condition may seriously affect the device and will be an error in
   bitstream creation. It should be corrected by properly specifying the pin
   location and I/O Standard.

Section 3 - Informational
-------------------------
INFO:LIT:243 - Logical network start has no load.
INFO:LIT:244 - All of the single ended outputs in this design are using slew
   rate limited output drivers. The delay on speed critical single ended outputs
   can be dramatically reduced by designating them as fast outputs.
INFO:Pack:1716 - Initializing temperature to 85.000 Celsius. (default - Range:
   0.000 to 85.000 Celsius)
INFO:Pack:1720 - Initializing voltage to 0.970 Volts. (default - Range: 0.970 to
   1.030 Volts)
INFO:Map:215 - The Interim Design Summary has been generated in the MAP Report
   (.mrp).
INFO:Pack:1650 - Map created a placed design.

Section 4 - Removed Logic Summary
---------------------------------
 185 block(s) removed
  40 block(s) optimized away
 185 signal(s) removed

Section 5 - Removed Logic
-------------------------

The trimmed logic report below shows the logic removed from your design due to
sourceless or loadless signals, and VCC or ground connections.  If the removal
of a signal or symbol results in the subsequent removal of an additional signal
or symbol, the message explaining that second removal will be indented.  This
indentation will be repeated as a chain of related logic is removed.

To quickly locate the original cause for the removal of a chain of logic, look
above the place where that logic is listed in the trimming report, then locate
the lines that are least indented (begin at the leftmost edge).

The signal "uut_PD_PA_Jac_233/SQ_SQ1_PA/A[139]_A[232]_AND_140_o" is sourceless
and has been removed.
 Sourceless block
"uut_PD_PA_Jac_233/SQ_SQ1_PA/Mxor_GND_9_o_GND_9_o_xor_188_OUT_233_xo<0>3" (ROM)
removed.
  The signal
"uut_PD_PA_Jac_233/SQ_SQ1_PA/Mxor_GND_9_o_GND_9_o_xor_188_OUT_233_xo<0>13" is
sourceless and has been removed.
The signal "uut_PD_PA_Jac_233/SQ_SQ1_PA/A[165]_A[232]_AND_166_o" is sourceless
and has been removed.
 Sourceless block
"uut_PD_PA_Jac_233/SQ_SQ1_PA/Mxor_GND_9_o_GND_9_o_xor_136_OUT_233_xo<0>2" (ROM)
removed.
  The signal
"uut_PD_PA_Jac_233/SQ_SQ1_PA/Mxor_GND_9_o_GND_9_o_xor_136_OUT_233_xo<0>1" is
sourceless and has been removed.
The signal "uut_PD_PA_Jac_233/SQ_SQ1_PA/A[140]_A[231]_AND_373_o" is sourceless
and has been removed.
The signal "uut_PD_PA_Jac_233/SQ_SQ1_PA/A[141]_A[230]_AND_606_o" is sourceless
and has been removed.
The signal "uut_PD_PA_Jac_233/SQ_SQ1_PA/A[142]_A[229]_AND_839_o" is sourceless
and has been removed.
The signal "uut_PD_PA_Jac_233/SQ_SQ1_PA/A[143]_A[228]_AND_1072_o" is sourceless
and has been removed.
The signal "uut_PD_PA_Jac_233/SQ_SQ1_PA/A[144]_A[227]_AND_1305_o" is sourceless
and has been removed.
The signal "uut_PD_PA_Jac_233/SQ_SQ1_PA/A[145]_A[226]_AND_1538_o" is sourceless
and has been removed.
 Sourceless block
```

"uut_PD_PA_Jac_233/SQ_SQ1_PA/Mxor_GND_9_o_GND_9_o_xor_188_OUT_233_xo<0>4" (ROM)
removed.
  The signal
"uut_PD_PA_Jac_233/SQ_SQ1_PA/Mxor_GND_9_o_GND_9_o_xor_188_OUT_233_xo<0>10" is
sourceless and has been removed.
The signal "uut_PD_PA_Jac_233/SQ_SQ1_PA/A[146]_A[225]_AND_1771_o" is sourceless
and has been removed.
The signal "uut_PD_PA_Jac_233/SQ_SQ1_PA/A[147]_A[224]_AND_2004_o" is sourceless
and has been removed.
The signal "uut_PD_PA_Jac_233/SQ_SQ1_PA/A[148]_A[223]_AND_2237_o" is sourceless
and has been removed.
The signal "uut_PD_PA_Jac_233/SQ_SQ1_PA/A[149]_A[222]_AND_2470_o" is sourceless
and has been removed.
The signal "uut_PD_PA_Jac_233/SQ_SQ1_PA/A[150]_A[221]_AND_2703_o" is sourceless
and has been removed.
The signal "uut_PD_PA_Jac_233/SQ_SQ1_PA/A[151]_A[220]_AND_2936_o" is sourceless
and has been removed.
 Sourceless block
"uut_PD_PA_Jac_233/SQ_SQ1_PA/Mxor_GND_9_o_GND_9_o_xor_188_OUT_233_xo<0>1" (ROM)
removed.
  The signal
"uut_PD_PA_Jac_233/SQ_SQ1_PA/Mxor_GND_9_o_GND_9_o_xor_188_OUT_233_xo<0>" is
sourceless and has been removed.
The signal "uut_PD_PA_Jac_233/SQ_SQ1_PA/A[152]_A[219]_AND_3169_o" is sourceless
and has been removed.
The signal "uut_PD_PA_Jac_233/SQ_SQ1_PA/A[153]_A[218]_AND_3402_o" is sourceless
and has been removed.
The signal "uut_PD_PA_Jac_233/SQ_SQ1_PA/A[154]_A[217]_AND_3635_o" is sourceless
and has been removed.
The signal "uut_PD_PA_Jac_233/SQ_SQ1_PA/A[155]_A[216]_AND_3868_o" is sourceless
and has been removed.
The signal "uut_PD_PA_Jac_233/SQ_SQ1_PA/A[156]_A[215]_AND_4101_o" is sourceless
and has been removed.
The signal "uut_PD_PA_Jac_233/SQ_SQ1_PA/A[166]_A[231]_AND_399_o" is sourceless
and has been removed.
The signal "uut_PD_PA_Jac_233/SQ_SQ1_PA/A[167]_A[230]_AND_632_o" is sourceless
and has been removed.
The signal "uut_PD_PA_Jac_233/SQ_SQ1_PA/A[168]_A[229]_AND_865_o" is sourceless
and has been removed.
The signal "uut_PD_PA_Jac_233/SQ_SQ1_PA/A[169]_A[228]_AND_1098_o" is sourceless
and has been removed.
The signal "uut_PD_PA_Jac_233/SQ_SQ1_PA/A[170]_A[227]_AND_1331_o" is sourceless
and has been removed.
The signal "uut_PD_PA_Jac_233/SQ_SQ1_PA/A[171]_A[226]_AND_1564_o" is sourceless
and has been removed.
 Sourceless block
"uut_PD_PA_Jac_233/SQ_SQ1_PA/Mxor_GND_9_o_GND_9_o_xor_136_OUT_233_xo<0>5" (ROM)
removed.
  The signal
"uut_PD_PA_Jac_233/SQ_SQ1_PA/Mxor_GND_9_o_GND_9_o_xor_136_OUT_233_xo<0>10" is
sourceless and has been removed.
The signal "uut_PD_PA_Jac_233/SQ_SQ1_PA/A[172]_A[225]_AND_1797_o" is sourceless
and has been removed.
The signal "uut_PD_PA_Jac_233/SQ_SQ1_PA/A[173]_A[224]_AND_2030_o" is sourceless
and has been removed.
The signal "uut_PD_PA_Jac_233/SQ_SQ1_PA/A[174]_A[223]_AND_2263_o" is sourceless
and has been removed.
The signal "uut_PD_PA_Jac_233/SQ_SQ1_PA/A[175]_A[222]_AND_2496_o" is sourceless
and has been removed.
The signal "uut_PD_PA_Jac_233/SQ_SQ1_PA/A[176]_A[221]_AND_2729_o" is sourceless
and has been removed.
The signal "uut_PD_PA_Jac_233/SQ_SQ1_PA/A[177]_A[220]_AND_2962_o" is sourceless
and has been removed.
 Sourceless block
"uut_PD_PA_Jac_233/SQ_SQ1_PA/Mxor_GND_9_o_GND_9_o_xor_136_OUT_233_xo<0>6" (ROM)
removed.
  The signal
"uut_PD_PA_Jac_233/SQ_SQ1_PA/Mxor_GND_9_o_GND_9_o_xor_136_OUT_233_xo<0>5" is
sourceless and has been removed.
The signal "uut_PD_PA_Jac_233/SQ_SQ1_PA/A[178]_A[219]_AND_3195_o" is sourceless
and has been removed.

```
The signal "uut_PD_PA_Jac_233/SQ_SQ1_PA/A[179]_A[218]_AND_3428_o" is sourceless
and has been removed.
The signal "uut_PD_PA_Jac_233/SQ_SQ1_PA/A[180]_A[217]_AND_3661_o" is sourceless
and has been removed.
The signal "uut_PD_PA_Jac_233/SQ_SQ1_PA/A[181]_A[216]_AND_3894_o" is sourceless
and has been removed.
The signal "uut_PD_PA_Jac_233/SQ_SQ1_PA/A[182]_A[215]_AND_4127_o" is sourceless
and has been removed.
The signal "uut_PD_PA_Jac_233/SQ_SQ1_PA/A[183]_A[214]_AND_4360_o" is sourceless
and has been removed.
 Sourceless block
"uut_PD_PA_Jac_233/SQ_SQ1_PA/Mxor_GND_9_o_GND_9_o_xor_136_OUT_233_xo<0>4" (ROM)
removed.
  The signal
"uut_PD_PA_Jac_233/SQ_SQ1_PA/Mxor_GND_9_o_GND_9_o_xor_136_OUT_233_xo<0>3" is
sourceless and has been removed.
The signal "uut_PD_PA_Jac_233/SQ_SQ1_PA/A[184]_A[213]_AND_4593_o" is sourceless
and has been removed.
The signal "uut_PD_PA_Jac_233/SQ_SQ1_PA/A[185]_A[212]_AND_4826_o" is sourceless
and has been removed.
The signal "uut_PD_PA_Jac_233/SQ_SQ1_PA/A[186]_A[211]_AND_5059_o" is sourceless
and has been removed.
The signal "uut_PD_PA_Jac_233/SQ_SQ1_PA/A[187]_A[210]_AND_5292_o" is sourceless
and has been removed.
The signal "uut_PD_PA_Jac_233/SQ_SQ1_PA/A[188]_A[209]_AND_5525_o" is sourceless
and has been removed.
The signal "uut_PD_PA_Jac_233/SQ_SQ1_PA/A[204]_A[231]_AND_437_o" is sourceless
and has been removed.
The signal "uut_PD_PA_Jac_233/SQ_SQ1_PA/A[208]_A[228]_AND_1137_o" is sourceless
and has been removed.
The signal "uut_PD_PA_Jac_233/SQ_SQ1_PA/A[209]_A[227]_AND_1370_o" is sourceless
and has been removed.
The signal "uut_PD_PA_Jac_233/SQ_SQ1_PA/A[209]_A[226]_AND_1602_o" is sourceless
and has been removed.
The signal "uut_PD_PA_Jac_233/SQ_SQ1_PA/A[210]_A[226]_AND_1603_o" is sourceless
and has been removed.
The signal "uut_PD_PA_Jac_233/SQ_SQ1_PA/A[210]_A[225]_AND_1835_o" is sourceless
and has been removed.
The signal "uut_PD_PA_Jac_233/SQ_SQ1_PA/A[211]_A[225]_AND_1836_o" is sourceless
and has been removed.
The signal "uut_PD_PA_Jac_233/SQ_SQ1_PA/A[211]_A[224]_AND_2068_o" is sourceless
and has been removed.
The signal "uut_PD_PA_Jac_233/SQ_SQ1_PA/A[212]_A[224]_AND_2069_o" is sourceless
and has been removed.
The signal "uut_PD_PA_Jac_233/SQ_SQ1_PA/A[212]_A[223]_AND_2301_o" is sourceless
and has been removed.
The signal "uut_PD_PA_Jac_233/SQ_SQ4_PD/A[165]_A[232]_AND_166_o" is sourceless
and has been removed.
 Sourceless block
"uut_PD_PA_Jac_233/SQ_SQ4_PD/Mxor_GND_9_o_GND_9_o_xor_136_OUT_233_xo<0>2" (ROM)
removed.
  The signal
"uut_PD_PA_Jac_233/SQ_SQ4_PD/Mxor_GND_9_o_GND_9_o_xor_136_OUT_233_xo<0>1" is
sourceless and has been removed.
The signal "uut_PD_PA_Jac_233/SQ_SQ4_PD/A[166]_A[231]_AND_399_o" is sourceless
and has been removed.
The signal "uut_PD_PA_Jac_233/SQ_SQ4_PD/A[167]_A[230]_AND_632_o" is sourceless
and has been removed.
The signal "uut_PD_PA_Jac_233/SQ_SQ4_PD/A[168]_A[229]_AND_865_o" is sourceless
and has been removed.
The signal "uut_PD_PA_Jac_233/SQ_SQ4_PD/A[169]_A[228]_AND_1098_o" is sourceless
and has been removed.
The signal "uut_PD_PA_Jac_233/SQ_SQ4_PD/A[170]_A[227]_AND_1331_o" is sourceless
and has been removed.
The signal "uut_PD_PA_Jac_233/SQ_SQ4_PD/A[171]_A[226]_AND_1564_o" is sourceless
and has been removed.
 Sourceless block
"uut_PD_PA_Jac_233/SQ_SQ4_PD/Mxor_GND_9_o_GND_9_o_xor_136_OUT_233_xo<0>5" (ROM)
removed.
  The signal
"uut_PD_PA_Jac_233/SQ_SQ4_PD/Mxor_GND_9_o_GND_9_o_xor_136_OUT_233_xo<0>10" is
```

sourceless and has been removed.
The signal "uut_PD_PA_Jac_233/SQ_SQ4_PD/A[172]_A[225]_AND_1797_o" is sourceless and has been removed.
The signal "uut_PD_PA_Jac_233/SQ_SQ4_PD/A[173]_A[224]_AND_2030_o" is sourceless and has been removed.
The signal "uut_PD_PA_Jac_233/SQ_SQ4_PD/A[174]_A[223]_AND_2263_o" is sourceless and has been removed.
The signal "uut_PD_PA_Jac_233/SQ_SQ4_PD/A[175]_A[222]_AND_2496_o" is sourceless and has been removed.
The signal "uut_PD_PA_Jac_233/SQ_SQ4_PD/A[176]_A[221]_AND_2729_o" is sourceless and has been removed.
The signal "uut_PD_PA_Jac_233/SQ_SQ4_PD/A[177]_A[220]_AND_2962_o" is sourceless and has been removed.
Sourceless block "uut_PD_PA_Jac_233/SQ_SQ4_PD/Mxor_GND_9_o_GND_9_o_xor_136_OUT_233_xo<0>6" (ROM) removed.
The signal "uut_PD_PA_Jac_233/SQ_SQ4_PD/Mxor_GND_9_o_GND_9_o_xor_136_OUT_233_xo<0>5" is sourceless and has been removed.
The signal "uut_PD_PA_Jac_233/SQ_SQ4_PD/A[178]_A[219]_AND_3195_o" is sourceless and has been removed.
The signal "uut_PD_PA_Jac_233/SQ_SQ4_PD/A[179]_A[218]_AND_3428_o" is sourceless and has been removed.
The signal "uut_PD_PA_Jac_233/SQ_SQ4_PD/A[180]_A[217]_AND_3661_o" is sourceless and has been removed.
The signal "uut_PD_PA_Jac_233/SQ_SQ4_PD/A[181]_A[216]_AND_3894_o" is sourceless and has been removed.
The signal "uut_PD_PA_Jac_233/SQ_SQ4_PD/A[182]_A[215]_AND_4127_o" is sourceless and has been removed.
The signal "uut_PD_PA_Jac_233/SQ_SQ4_PD/A[183]_A[214]_AND_4360_o" is sourceless and has been removed.
Sourceless block "uut_PD_PA_Jac_233/SQ_SQ4_PD/Mxor_GND_9_o_GND_9_o_xor_136_OUT_233_xo<0>4" (ROM) removed.
The signal "uut_PD_PA_Jac_233/SQ_SQ4_PD/Mxor_GND_9_o_GND_9_o_xor_136_OUT_233_xo<0>3" is sourceless and has been removed.
The signal "uut_PD_PA_Jac_233/SQ_SQ4_PD/A[184]_A[213]_AND_4593_o" is sourceless and has been removed.
The signal "uut_PD_PA_Jac_233/SQ_SQ4_PD/A[185]_A[212]_AND_4826_o" is sourceless and has been removed.
The signal "uut_PD_PA_Jac_233/SQ_SQ4_PD/A[186]_A[211]_AND_5059_o" is sourceless and has been removed.
The signal "uut_PD_PA_Jac_233/SQ_SQ4_PD/A[187]_A[210]_AND_5292_o" is sourceless and has been removed.
The signal "uut_PD_PA_Jac_233/SQ_SQ4_PD/A[188]_A[209]_AND_5525_o" is sourceless and has been removed.
The signal "uut_PD_PA_Jac_233/SQ_SQ1_PD/A[133]_A[232]_AND_134_o" is sourceless and has been removed.
Sourceless block "uut_PD_PA_Jac_233/SQ_SQ1_PD/Mxor_GND_9_o_GND_9_o_xor_200_OUT_233_xo<0>9" (ROM) removed.
The signal "uut_PD_PA_Jac_233/SQ_SQ1_PD/Mxor_GND_9_o_GND_9_o_xor_200_OUT_233_xo<0>15" is sourceless and has been removed.
The signal "uut_PD_PA_Jac_233/SQ_SQ1_PD/A[139]_A[232]_AND_140_o" is sourceless and has been removed.
Sourceless block "uut_PD_PA_Jac_233/SQ_SQ1_PD/Mxor_GND_9_o_GND_9_o_xor_188_OUT_233_xo<0>3" (ROM) removed.
The signal "uut_PD_PA_Jac_233/SQ_SQ1_PD/Mxor_GND_9_o_GND_9_o_xor_188_OUT_233_xo<0>13" is sourceless and has been removed.
The signal "uut_PD_PA_Jac_233/SQ_SQ1_PD/A[165]_A[232]_AND_166_o" is sourceless and has been removed.
Sourceless block "uut_PD_PA_Jac_233/SQ_SQ1_PD/Mxor_GND_9_o_GND_9_o_xor_136_OUT_233_xo<0>2" (ROM) removed.
The signal "uut_PD_PA_Jac_233/SQ_SQ1_PD/Mxor_GND_9_o_GND_9_o_xor_136_OUT_233_xo<0>1" is sourceless and has been removed.

The signal "uut_PD_PA_Jac_233/SQ_SQ1_PD/A[134]_A[231]_AND_367_o" is sourceless and has been removed.
The signal "uut_PD_PA_Jac_233/SQ_SQ1_PD/A[135]_A[230]_AND_600_o" is sourceless and has been removed.
The signal "uut_PD_PA_Jac_233/SQ_SQ1_PD/A[136]_A[229]_AND_833_o" is sourceless and has been removed.
The signal "uut_PD_PA_Jac_233/SQ_SQ1_PD/A[137]_A[228]_AND_1066_o" is sourceless and has been removed.
The signal "uut_PD_PA_Jac_233/SQ_SQ1_PD/A[138]_A[227]_AND_1299_o" is sourceless and has been removed.
The signal "uut_PD_PA_Jac_233/SQ_SQ1_PD/A[139]_A[226]_AND_1532_o" is sourceless and has been removed.
Sourceless block "uut_PD_PA_Jac_233/SQ_SQ1_PD/Mxor_GND_9_o_GND_9_o_xor_200_OUT_233_xo<0>6" (ROM) removed.
The signal "uut_PD_PA_Jac_233/SQ_SQ1_PD/Mxor_GND_9_o_GND_9_o_xor_200_OUT_233_xo<0>16" is sourceless and has been removed.
The signal "uut_PD_PA_Jac_233/SQ_SQ1_PD/A[140]_A[231]_AND_373_o" is sourceless and has been removed.
The signal "uut_PD_PA_Jac_233/SQ_SQ1_PD/A[140]_A[225]_AND_1765_o" is sourceless and has been removed.
The signal "uut_PD_PA_Jac_233/SQ_SQ1_PD/A[141]_A[230]_AND_606_o" is sourceless and has been removed.
The signal "uut_PD_PA_Jac_233/SQ_SQ1_PD/A[141]_A[224]_AND_1998_o" is sourceless and has been removed.
The signal "uut_PD_PA_Jac_233/SQ_SQ1_PD/A[142]_A[229]_AND_839_o" is sourceless and has been removed.
The signal "uut_PD_PA_Jac_233/SQ_SQ1_PD/A[142]_A[223]_AND_2231_o" is sourceless and has been removed.
The signal "uut_PD_PA_Jac_233/SQ_SQ1_PD/A[143]_A[228]_AND_1072_o" is sourceless and has been removed.
The signal "uut_PD_PA_Jac_233/SQ_SQ1_PD/A[143]_A[222]_AND_2464_o" is sourceless and has been removed.
The signal "uut_PD_PA_Jac_233/SQ_SQ1_PD/A[144]_A[227]_AND_1305_o" is sourceless and has been removed.
The signal "uut_PD_PA_Jac_233/SQ_SQ1_PD/A[144]_A[221]_AND_2697_o" is sourceless and has been removed.
The signal "uut_PD_PA_Jac_233/SQ_SQ1_PD/A[145]_A[226]_AND_1538_o" is sourceless and has been removed.
Sourceless block "uut_PD_PA_Jac_233/SQ_SQ1_PD/Mxor_GND_9_o_GND_9_o_xor_188_OUT_233_xo<0>4" (ROM) removed.
The signal "uut_PD_PA_Jac_233/SQ_SQ1_PD/Mxor_GND_9_o_GND_9_o_xor_188_OUT_233_xo<0>10" is sourceless and has been removed.
The signal "uut_PD_PA_Jac_233/SQ_SQ1_PD/A[145]_A[220]_AND_2930_o" is sourceless and has been removed.
Sourceless block "uut_PD_PA_Jac_233/SQ_SQ1_PD/Mxor_GND_9_o_GND_9_o_xor_200_OUT_233_xo<0>7" (ROM) removed.
The signal "uut_PD_PA_Jac_233/SQ_SQ1_PD/Mxor_GND_9_o_GND_9_o_xor_200_OUT_233_xo<0>17" is sourceless and has been removed.
The signal "uut_PD_PA_Jac_233/SQ_SQ1_PD/A[146]_A[225]_AND_1771_o" is sourceless and has been removed.
The signal "uut_PD_PA_Jac_233/SQ_SQ1_PD/A[146]_A[219]_AND_3163_o" is sourceless and has been removed.
The signal "uut_PD_PA_Jac_233/SQ_SQ1_PD/A[147]_A[224]_AND_2004_o" is sourceless and has been removed.
The signal "uut_PD_PA_Jac_233/SQ_SQ1_PD/A[147]_A[218]_AND_3396_o" is sourceless and has been removed.
The signal "uut_PD_PA_Jac_233/SQ_SQ1_PD/A[148]_A[223]_AND_2237_o" is sourceless and has been removed.
The signal "uut_PD_PA_Jac_233/SQ_SQ1_PD/A[148]_A[217]_AND_3629_o" is sourceless and has been removed.
The signal "uut_PD_PA_Jac_233/SQ_SQ1_PD/A[149]_A[222]_AND_2470_o" is sourceless and has been removed.
The signal "uut_PD_PA_Jac_233/SQ_SQ1_PD/A[149]_A[216]_AND_3862_o" is sourceless and has been removed.
The signal "uut_PD_PA_Jac_233/SQ_SQ1_PD/A[150]_A[221]_AND_2703_o" is sourceless

and has been removed.
The signal "uut_PD_PA_Jac_233/SQ_SQ1_PD/A[150]_A[215]_AND_4095_o" is sourceless and has been removed.
The signal "uut_PD_PA_Jac_233/SQ_SQ1_PD/A[151]_A[220]_AND_2936_o" is sourceless and has been removed.
Sourceless block "uut_PD_PA_Jac_233/SQ_SQ1_PD/Mxor_GND_9_o_GND_9_o_xor_188_OUT_233_xo<0>1" (ROM) removed.
The signal "uut_PD_PA_Jac_233/SQ_SQ1_PD/Mxor_GND_9_o_GND_9_o_xor_188_OUT_233_xo<0>" is sourceless and has been removed.
The signal "uut_PD_PA_Jac_233/SQ_SQ1_PD/A[152]_A[219]_AND_3169_o" is sourceless and has been removed.
The signal "uut_PD_PA_Jac_233/SQ_SQ1_PD/A[153]_A[218]_AND_3402_o" is sourceless and has been removed.
The signal "uut_PD_PA_Jac_233/SQ_SQ1_PD/A[154]_A[217]_AND_3635_o" is sourceless and has been removed.
The signal "uut_PD_PA_Jac_233/SQ_SQ1_PD/A[155]_A[216]_AND_3868_o" is sourceless and has been removed.
The signal "uut_PD_PA_Jac_233/SQ_SQ1_PD/A[156]_A[215]_AND_4101_o" is sourceless and has been removed.
The signal "uut_PD_PA_Jac_233/SQ_SQ1_PD/A[166]_A[231]_AND_399_o" is sourceless and has been removed.
The signal "uut_PD_PA_Jac_233/SQ_SQ1_PD/A[167]_A[230]_AND_632_o" is sourceless and has been removed.
The signal "uut_PD_PA_Jac_233/SQ_SQ1_PD/A[168]_A[229]_AND_865_o" is sourceless and has been removed.
The signal "uut_PD_PA_Jac_233/SQ_SQ1_PD/A[169]_A[228]_AND_1098_o" is sourceless and has been removed.
The signal "uut_PD_PA_Jac_233/SQ_SQ1_PD/A[170]_A[227]_AND_1331_o" is sourceless and has been removed.
The signal "uut_PD_PA_Jac_233/SQ_SQ1_PD/A[171]_A[226]_AND_1564_o" is sourceless and has been removed.
Sourceless block "uut_PD_PA_Jac_233/SQ_SQ1_PD/Mxor_GND_9_o_GND_9_o_xor_136_OUT_233_xo<0>5" (ROM) removed.
The signal "uut_PD_PA_Jac_233/SQ_SQ1_PD/Mxor_GND_9_o_GND_9_o_xor_136_OUT_233_xo<0>10" is sourceless and has been removed.
The signal "uut_PD_PA_Jac_233/SQ_SQ1_PD/A[172]_A[225]_AND_1797_o" is sourceless and has been removed.
The signal "uut_PD_PA_Jac_233/SQ_SQ1_PD/A[173]_A[224]_AND_2030_o" is sourceless and has been removed.
The signal "uut_PD_PA_Jac_233/SQ_SQ1_PD/A[174]_A[223]_AND_2263_o" is sourceless and has been removed.
The signal "uut_PD_PA_Jac_233/SQ_SQ1_PD/A[175]_A[222]_AND_2496_o" is sourceless and has been removed.
The signal "uut_PD_PA_Jac_233/SQ_SQ1_PD/A[176]_A[221]_AND_2729_o" is sourceless and has been removed.
The signal "uut_PD_PA_Jac_233/SQ_SQ1_PD/A[177]_A[220]_AND_2962_o" is sourceless and has been removed.
Sourceless block "uut_PD_PA_Jac_233/SQ_SQ1_PD/Mxor_GND_9_o_GND_9_o_xor_136_OUT_233_xo<0>6" (ROM) removed.
The signal "uut_PD_PA_Jac_233/SQ_SQ1_PD/Mxor_GND_9_o_GND_9_o_xor_136_OUT_233_xo<0>5" is sourceless and has been removed.
The signal "uut_PD_PA_Jac_233/SQ_SQ1_PD/A[178]_A[219]_AND_3195_o" is sourceless and has been removed.
The signal "uut_PD_PA_Jac_233/SQ_SQ1_PD/A[179]_A[218]_AND_3428_o" is sourceless and has been removed.
The signal "uut_PD_PA_Jac_233/SQ_SQ1_PD/A[180]_A[217]_AND_3661_o" is sourceless and has been removed.
The signal "uut_PD_PA_Jac_233/SQ_SQ1_PD/A[181]_A[216]_AND_3894_o" is sourceless and has been removed.
The signal "uut_PD_PA_Jac_233/SQ_SQ1_PD/A[182]_A[215]_AND_4127_o" is sourceless and has been removed.
The signal "uut_PD_PA_Jac_233/SQ_SQ1_PD/A[183]_A[214]_AND_4360_o" is sourceless and has been removed.
Sourceless block "uut_PD_PA_Jac_233/SQ_SQ1_PD/Mxor_GND_9_o_GND_9_o_xor_136_OUT_233_xo<0>4" (ROM)

removed.
  The signal
"uut_PD_PA_Jac_233/SQ_SQ1_PD/Mxor_GND_9_o_GND_9_o_xor_136_OUT_233_xo<0>3" is
sourceless and has been removed.
The signal "uut_PD_PA_Jac_233/SQ_SQ1_PD/A[184]_A[213]_AND_4593_o" is sourceless
and has been removed.
The signal "uut_PD_PA_Jac_233/SQ_SQ1_PD/A[185]_A[212]_AND_4826_o" is sourceless
and has been removed.
The signal "uut_PD_PA_Jac_233/SQ_SQ1_PD/A[186]_A[211]_AND_5059_o" is sourceless
and has been removed.
The signal "uut_PD_PA_Jac_233/SQ_SQ1_PD/A[187]_A[210]_AND_5292_o" is sourceless
and has been removed.
The signal "uut_PD_PA_Jac_233/SQ_SQ1_PD/A[188]_A[209]_AND_5525_o" is sourceless
and has been removed.
The signal "uut_PD_PA_Jac_233/SQ_SQ2_PD/A[165]_A[232]_AND_166_o" is sourceless
and has been removed.
 Sourceless block
"uut_PD_PA_Jac_233/SQ_SQ2_PD/Mxor_GND_9_o_GND_9_o_xor_136_OUT_233_xo<0>2" (ROM)
removed.
  The signal
"uut_PD_PA_Jac_233/SQ_SQ2_PD/Mxor_GND_9_o_GND_9_o_xor_136_OUT_233_xo<0>1" is
sourceless and has been removed.
The signal "uut_PD_PA_Jac_233/SQ_SQ2_PD/A[166]_A[231]_AND_399_o" is sourceless
and has been removed.
The signal "uut_PD_PA_Jac_233/SQ_SQ2_PD/A[167]_A[230]_AND_632_o" is sourceless
and has been removed.
The signal "uut_PD_PA_Jac_233/SQ_SQ2_PD/A[168]_A[229]_AND_865_o" is sourceless
and has been removed.
The signal "uut_PD_PA_Jac_233/SQ_SQ2_PD/A[169]_A[228]_AND_1098_o" is sourceless
and has been removed.
The signal "uut_PD_PA_Jac_233/SQ_SQ2_PD/A[170]_A[227]_AND_1331_o" is sourceless
and has been removed.
The signal "uut_PD_PA_Jac_233/SQ_SQ2_PD/A[171]_A[226]_AND_1564_o" is sourceless
and has been removed.
 Sourceless block
"uut_PD_PA_Jac_233/SQ_SQ2_PD/Mxor_GND_9_o_GND_9_o_xor_136_OUT_233_xo<0>5" (ROM)
removed.
  The signal
"uut_PD_PA_Jac_233/SQ_SQ2_PD/Mxor_GND_9_o_GND_9_o_xor_136_OUT_233_xo<0>10" is
sourceless and has been removed.
The signal "uut_PD_PA_Jac_233/SQ_SQ2_PD/A[172]_A[225]_AND_1797_o" is sourceless
and has been removed.
The signal "uut_PD_PA_Jac_233/SQ_SQ2_PD/A[173]_A[224]_AND_2030_o" is sourceless
and has been removed.
The signal "uut_PD_PA_Jac_233/SQ_SQ2_PD/A[174]_A[223]_AND_2263_o" is sourceless
and has been removed.
The signal "uut_PD_PA_Jac_233/SQ_SQ2_PD/A[175]_A[222]_AND_2496_o" is sourceless
and has been removed.
The signal "uut_PD_PA_Jac_233/SQ_SQ2_PD/A[176]_A[221]_AND_2729_o" is sourceless
and has been removed.
The signal "uut_PD_PA_Jac_233/SQ_SQ2_PD/A[177]_A[220]_AND_2962_o" is sourceless
and has been removed.
 Sourceless block
"uut_PD_PA_Jac_233/SQ_SQ2_PD/Mxor_GND_9_o_GND_9_o_xor_136_OUT_233_xo<0>6" (ROM)
removed.
  The signal
"uut_PD_PA_Jac_233/SQ_SQ2_PD/Mxor_GND_9_o_GND_9_o_xor_136_OUT_233_xo<0>5" is
sourceless and has been removed.
The signal "uut_PD_PA_Jac_233/SQ_SQ2_PD/A[178]_A[219]_AND_3195_o" is sourceless
and has been removed.
The signal "uut_PD_PA_Jac_233/SQ_SQ2_PD/A[179]_A[218]_AND_3428_o" is sourceless
and has been removed.
The signal "uut_PD_PA_Jac_233/SQ_SQ2_PD/A[180]_A[217]_AND_3661_o" is sourceless
and has been removed.
The signal "uut_PD_PA_Jac_233/SQ_SQ2_PD/A[181]_A[216]_AND_3894_o" is sourceless
and has been removed.
The signal "uut_PD_PA_Jac_233/SQ_SQ2_PD/A[182]_A[215]_AND_4127_o" is sourceless
and has been removed.
The signal "uut_PD_PA_Jac_233/SQ_SQ2_PD/A[183]_A[214]_AND_4360_o" is sourceless
and has been removed.
 Sourceless block

"uut_PD_PA_Jac_233/SQ_SQ2_PD/Mxor_GND_9_o_GND_9_o_xor_136_OUT_233_xo<0>4" (ROM) removed.
  The signal "uut_PD_PA_Jac_233/SQ_SQ2_PD/Mxor_GND_9_o_GND_9_o_xor_136_OUT_233_xo<0>3" is sourceless and has been removed.
The signal "uut_PD_PA_Jac_233/SQ_SQ2_PD/A[184]_A[213]_AND_4593_o" is sourceless and has been removed.
The signal "uut_PD_PA_Jac_233/SQ_SQ2_PD/A[185]_A[212]_AND_4826_o" is sourceless and has been removed.
The signal "uut_PD_PA_Jac_233/SQ_SQ2_PD/A[186]_A[211]_AND_5059_o" is sourceless and has been removed.
The signal "uut_PD_PA_Jac_233/SQ_SQ2_PD/A[187]_A[210]_AND_5292_o" is sourceless and has been removed.
The signal "uut_PD_PA_Jac_233/SQ_SQ2_PD/A[188]_A[209]_AND_5525_o" is sourceless and has been removed.
Unused block "uut_PD_PA_Jac_233/SQ_SQ1_PA/A[139]_A[232]_AND_140_o1" (ROM) removed.
Unused block "uut_PD_PA_Jac_233/SQ_SQ1_PA/A[140]_A[231]_AND_373_o1" (ROM) removed.
Unused block "uut_PD_PA_Jac_233/SQ_SQ1_PA/A[141]_A[230]_AND_606_o1" (ROM) removed.
Unused block "uut_PD_PA_Jac_233/SQ_SQ1_PA/A[142]_A[229]_AND_839_o1" (ROM) removed.
Unused block "uut_PD_PA_Jac_233/SQ_SQ1_PA/A[143]_A[228]_AND_1072_o1" (ROM) removed.
Unused block "uut_PD_PA_Jac_233/SQ_SQ1_PA/A[144]_A[227]_AND_1305_o1" (ROM) removed.
Unused block "uut_PD_PA_Jac_233/SQ_SQ1_PA/A[145]_A[226]_AND_1538_o1" (ROM) removed.
Unused block "uut_PD_PA_Jac_233/SQ_SQ1_PA/A[146]_A[225]_AND_1771_o1" (ROM) removed.
Unused block "uut_PD_PA_Jac_233/SQ_SQ1_PA/A[147]_A[224]_AND_2004_o1" (ROM) removed.
Unused block "uut_PD_PA_Jac_233/SQ_SQ1_PA/A[148]_A[223]_AND_2237_o1" (ROM) removed.
Unused block "uut_PD_PA_Jac_233/SQ_SQ1_PA/A[149]_A[222]_AND_2470_o1" (ROM) removed.
Unused block "uut_PD_PA_Jac_233/SQ_SQ1_PA/A[150]_A[221]_AND_2703_o1" (ROM) removed.
Unused block "uut_PD_PA_Jac_233/SQ_SQ1_PA/A[151]_A[220]_AND_2936_o1" (ROM) removed.
Unused block "uut_PD_PA_Jac_233/SQ_SQ1_PA/A[152]_A[219]_AND_3169_o1" (ROM) removed.
Unused block "uut_PD_PA_Jac_233/SQ_SQ1_PA/A[153]_A[218]_AND_3402_o1" (ROM) removed.
Unused block "uut_PD_PA_Jac_233/SQ_SQ1_PA/A[154]_A[217]_AND_3635_o1" (ROM) removed.
Unused block "uut_PD_PA_Jac_233/SQ_SQ1_PA/A[155]_A[216]_AND_3868_o1" (ROM) removed.
Unused block "uut_PD_PA_Jac_233/SQ_SQ1_PA/A[156]_A[215]_AND_4101_o1" (ROM) removed.
Unused block "uut_PD_PA_Jac_233/SQ_SQ1_PA/A[165]_A[232]_AND_166_o1" (ROM) removed.
Unused block "uut_PD_PA_Jac_233/SQ_SQ1_PA/A[166]_A[231]_AND_399_o1" (ROM) removed.
Unused block "uut_PD_PA_Jac_233/SQ_SQ1_PA/A[167]_A[230]_AND_632_o1" (ROM) removed.
Unused block "uut_PD_PA_Jac_233/SQ_SQ1_PA/A[168]_A[229]_AND_865_o1" (ROM) removed.
Unused block "uut_PD_PA_Jac_233/SQ_SQ1_PA/A[169]_A[228]_AND_1098_o1" (ROM) removed.
Unused block "uut_PD_PA_Jac_233/SQ_SQ1_PA/A[170]_A[227]_AND_1331_o1" (ROM) removed.
Unused block "uut_PD_PA_Jac_233/SQ_SQ1_PA/A[171]_A[226]_AND_1564_o1" (ROM) removed.
Unused block "uut_PD_PA_Jac_233/SQ_SQ1_PA/A[172]_A[225]_AND_1797_o1" (ROM) removed.
Unused block "uut_PD_PA_Jac_233/SQ_SQ1_PA/A[173]_A[224]_AND_2030_o1" (ROM) removed.
Unused block "uut_PD_PA_Jac_233/SQ_SQ1_PA/A[174]_A[223]_AND_2263_o1" (ROM) removed.

```
Unused block "uut_PD_PA_Jac_233/SQ_SQ1_PA/A[175]_A[222]_AND_2496_o1" (ROM)
removed.
Unused block "uut_PD_PA_Jac_233/SQ_SQ1_PA/A[176]_A[221]_AND_2729_o1" (ROM)
removed.
Unused block "uut_PD_PA_Jac_233/SQ_SQ1_PA/A[177]_A[220]_AND_2962_o1" (ROM)
removed.
Unused block "uut_PD_PA_Jac_233/SQ_SQ1_PA/A[178]_A[219]_AND_3195_o1" (ROM)
removed.
Unused block "uut_PD_PA_Jac_233/SQ_SQ1_PA/A[179]_A[218]_AND_3428_o1" (ROM)
removed.
Unused block "uut_PD_PA_Jac_233/SQ_SQ1_PA/A[180]_A[217]_AND_3661_o1" (ROM)
removed.
Unused block "uut_PD_PA_Jac_233/SQ_SQ1_PA/A[181]_A[216]_AND_3894_o1" (ROM)
removed.
Unused block "uut_PD_PA_Jac_233/SQ_SQ1_PA/A[182]_A[215]_AND_4127_o1" (ROM)
removed.
Unused block "uut_PD_PA_Jac_233/SQ_SQ1_PA/A[183]_A[214]_AND_4360_o1" (ROM)
removed.
Unused block "uut_PD_PA_Jac_233/SQ_SQ1_PA/A[184]_A[213]_AND_4593_o1" (ROM)
removed.
Unused block "uut_PD_PA_Jac_233/SQ_SQ1_PA/A[185]_A[212]_AND_4826_o1" (ROM)
removed.
Unused block "uut_PD_PA_Jac_233/SQ_SQ1_PA/A[186]_A[211]_AND_5059_o1" (ROM)
removed.
Unused block "uut_PD_PA_Jac_233/SQ_SQ1_PA/A[187]_A[210]_AND_5292_o1" (ROM)
removed.
Unused block "uut_PD_PA_Jac_233/SQ_SQ1_PA/A[188]_A[209]_AND_5525_o1" (ROM)
removed.
Unused block "uut_PD_PA_Jac_233/SQ_SQ1_PA/A[204]_A[231]_AND_437_o1" (ROM)
removed.
Unused block "uut_PD_PA_Jac_233/SQ_SQ1_PA/A[208]_A[228]_AND_1137_o1" (ROM)
removed.
Unused block "uut_PD_PA_Jac_233/SQ_SQ1_PA/A[209]_A[226]_AND_1602_o1" (ROM)
removed.
Unused block "uut_PD_PA_Jac_233/SQ_SQ1_PA/A[209]_A[227]_AND_1370_o1" (ROM)
removed.
Unused block "uut_PD_PA_Jac_233/SQ_SQ1_PA/A[210]_A[225]_AND_1835_o1" (ROM)
removed.
Unused block "uut_PD_PA_Jac_233/SQ_SQ1_PA/A[210]_A[226]_AND_1603_o1" (ROM)
removed.
Unused block "uut_PD_PA_Jac_233/SQ_SQ1_PA/A[211]_A[224]_AND_2068_o1" (ROM)
removed.
Unused block "uut_PD_PA_Jac_233/SQ_SQ1_PA/A[211]_A[225]_AND_1836_o1" (ROM)
removed.
Unused block "uut_PD_PA_Jac_233/SQ_SQ1_PA/A[212]_A[223]_AND_2301_o1" (ROM)
removed.
Unused block "uut_PD_PA_Jac_233/SQ_SQ1_PA/A[212]_A[224]_AND_2069_o1" (ROM)
removed.
Unused block "uut_PD_PA_Jac_233/SQ_SQ1_PD/A[133]_A[232]_AND_134_o1" (ROM)
removed.
Unused block "uut_PD_PA_Jac_233/SQ_SQ1_PD/A[134]_A[231]_AND_367_o1" (ROM)
removed.
Unused block "uut_PD_PA_Jac_233/SQ_SQ1_PD/A[135]_A[230]_AND_600_o1" (ROM)
removed.
Unused block "uut_PD_PA_Jac_233/SQ_SQ1_PD/A[136]_A[229]_AND_833_o1" (ROM)
removed.
Unused block "uut_PD_PA_Jac_233/SQ_SQ1_PD/A[137]_A[228]_AND_1066_o1" (ROM)
removed.
Unused block "uut_PD_PA_Jac_233/SQ_SQ1_PD/A[138]_A[227]_AND_1299_o1" (ROM)
removed.
Unused block "uut_PD_PA_Jac_233/SQ_SQ1_PD/A[139]_A[226]_AND_1532_o1" (ROM)
removed.
Unused block "uut_PD_PA_Jac_233/SQ_SQ1_PD/A[139]_A[232]_AND_140_o1" (ROM)
removed.
Unused block "uut_PD_PA_Jac_233/SQ_SQ1_PD/A[140]_A[225]_AND_1765_o1" (ROM)
removed.
Unused block "uut_PD_PA_Jac_233/SQ_SQ1_PD/A[140]_A[231]_AND_373_o1" (ROM)
removed.
Unused block "uut_PD_PA_Jac_233/SQ_SQ1_PD/A[141]_A[224]_AND_1998_o1" (ROM)
removed.
Unused block "uut_PD_PA_Jac_233/SQ_SQ1_PD/A[141]_A[230]_AND_606_o1" (ROM)
```

```
removed.
Unused block "uut_PD_PA_Jac_233/SQ_SQ1_PD/A[142]_A[223]_AND_2231_o1" (ROM)
removed.
Unused block "uut_PD_PA_Jac_233/SQ_SQ1_PD/A[142]_A[229]_AND_839_o1" (ROM)
removed.
Unused block "uut_PD_PA_Jac_233/SQ_SQ1_PD/A[143]_A[222]_AND_2464_o1" (ROM)
removed.
Unused block "uut_PD_PA_Jac_233/SQ_SQ1_PD/A[143]_A[228]_AND_1072_o1" (ROM)
removed.
Unused block "uut_PD_PA_Jac_233/SQ_SQ1_PD/A[144]_A[221]_AND_2697_o1" (ROM)
removed.
Unused block "uut_PD_PA_Jac_233/SQ_SQ1_PD/A[144]_A[227]_AND_1305_o1" (ROM)
removed.
Unused block "uut_PD_PA_Jac_233/SQ_SQ1_PD/A[145]_A[220]_AND_2930_o1" (ROM)
removed.
Unused block "uut_PD_PA_Jac_233/SQ_SQ1_PD/A[145]_A[226]_AND_1538_o1" (ROM)
removed.
Unused block "uut_PD_PA_Jac_233/SQ_SQ1_PD/A[146]_A[219]_AND_3163_o1" (ROM)
removed.
Unused block "uut_PD_PA_Jac_233/SQ_SQ1_PD/A[146]_A[225]_AND_1771_o1" (ROM)
removed.
Unused block "uut_PD_PA_Jac_233/SQ_SQ1_PD/A[147]_A[218]_AND_3396_o1" (ROM)
removed.
Unused block "uut_PD_PA_Jac_233/SQ_SQ1_PD/A[147]_A[224]_AND_2004_o1" (ROM)
removed.
Unused block "uut_PD_PA_Jac_233/SQ_SQ1_PD/A[148]_A[217]_AND_3629_o1" (ROM)
removed.
Unused block "uut_PD_PA_Jac_233/SQ_SQ1_PD/A[148]_A[223]_AND_2237_o1" (ROM)
removed.
Unused block "uut_PD_PA_Jac_233/SQ_SQ1_PD/A[149]_A[216]_AND_3862_o1" (ROM)
removed.
Unused block "uut_PD_PA_Jac_233/SQ_SQ1_PD/A[149]_A[222]_AND_2470_o1" (ROM)
removed.
Unused block "uut_PD_PA_Jac_233/SQ_SQ1_PD/A[150]_A[215]_AND_4095_o1" (ROM)
removed.
Unused block "uut_PD_PA_Jac_233/SQ_SQ1_PD/A[150]_A[221]_AND_2703_o1" (ROM)
removed.
Unused block "uut_PD_PA_Jac_233/SQ_SQ1_PD/A[151]_A[220]_AND_2936_o1" (ROM)
removed.
Unused block "uut_PD_PA_Jac_233/SQ_SQ1_PD/A[152]_A[219]_AND_3169_o1" (ROM)
removed.
Unused block "uut_PD_PA_Jac_233/SQ_SQ1_PD/A[153]_A[218]_AND_3402_o1" (ROM)
removed.
Unused block "uut_PD_PA_Jac_233/SQ_SQ1_PD/A[154]_A[217]_AND_3635_o1" (ROM)
removed.
Unused block "uut_PD_PA_Jac_233/SQ_SQ1_PD/A[155]_A[216]_AND_3868_o1" (ROM)
removed.
Unused block "uut_PD_PA_Jac_233/SQ_SQ1_PD/A[156]_A[215]_AND_4101_o1" (ROM)
removed.
Unused block "uut_PD_PA_Jac_233/SQ_SQ1_PD/A[165]_A[232]_AND_166_o1" (ROM)
removed.
Unused block "uut_PD_PA_Jac_233/SQ_SQ1_PD/A[166]_A[231]_AND_399_o1" (ROM)
removed.
Unused block "uut_PD_PA_Jac_233/SQ_SQ1_PD/A[167]_A[230]_AND_632_o1" (ROM)
removed.
Unused block "uut_PD_PA_Jac_233/SQ_SQ1_PD/A[168]_A[229]_AND_865_o1" (ROM)
removed.
Unused block "uut_PD_PA_Jac_233/SQ_SQ1_PD/A[169]_A[228]_AND_1098_o1" (ROM)
removed.
Unused block "uut_PD_PA_Jac_233/SQ_SQ1_PD/A[170]_A[227]_AND_1331_o1" (ROM)
removed.
Unused block "uut_PD_PA_Jac_233/SQ_SQ1_PD/A[171]_A[226]_AND_1564_o1" (ROM)
removed.
Unused block "uut_PD_PA_Jac_233/SQ_SQ1_PD/A[172]_A[225]_AND_1797_o1" (ROM)
removed.
Unused block "uut_PD_PA_Jac_233/SQ_SQ1_PD/A[173]_A[224]_AND_2030_o1" (ROM)
removed.
Unused block "uut_PD_PA_Jac_233/SQ_SQ1_PD/A[174]_A[223]_AND_2263_o1" (ROM)
removed.
Unused block "uut_PD_PA_Jac_233/SQ_SQ1_PD/A[175]_A[222]_AND_2496_o1" (ROM)
removed.
```

Unused block "uut_PD_PA_Jac_233/SQ_SQ1_PD/A[176]_A[221]_AND_2729_o1" (ROM) removed.
Unused block "uut_PD_PA_Jac_233/SQ_SQ1_PD/A[177]_A[220]_AND_2962_o1" (ROM) removed.
Unused block "uut_PD_PA_Jac_233/SQ_SQ1_PD/A[178]_A[219]_AND_3195_o1" (ROM) removed.
Unused block "uut_PD_PA_Jac_233/SQ_SQ1_PD/A[179]_A[218]_AND_3428_o1" (ROM) removed.
Unused block "uut_PD_PA_Jac_233/SQ_SQ1_PD/A[180]_A[217]_AND_3661_o1" (ROM) removed.
Unused block "uut_PD_PA_Jac_233/SQ_SQ1_PD/A[181]_A[216]_AND_3894_o1" (ROM) removed.
Unused block "uut_PD_PA_Jac_233/SQ_SQ1_PD/A[182]_A[215]_AND_4127_o1" (ROM) removed.
Unused block "uut_PD_PA_Jac_233/SQ_SQ1_PD/A[183]_A[214]_AND_4360_o1" (ROM) removed.
Unused block "uut_PD_PA_Jac_233/SQ_SQ1_PD/A[184]_A[213]_AND_4593_o1" (ROM) removed.
Unused block "uut_PD_PA_Jac_233/SQ_SQ1_PD/A[185]_A[212]_AND_4826_o1" (ROM) removed.
Unused block "uut_PD_PA_Jac_233/SQ_SQ1_PD/A[186]_A[211]_AND_5059_o1" (ROM) removed.
Unused block "uut_PD_PA_Jac_233/SQ_SQ1_PD/A[187]_A[210]_AND_5292_o1" (ROM) removed.
Unused block "uut_PD_PA_Jac_233/SQ_SQ1_PD/A[188]_A[209]_AND_5525_o1" (ROM) removed.
Unused block "uut_PD_PA_Jac_233/SQ_SQ2_PD/A[165]_A[232]_AND_166_o1" (ROM) removed.
Unused block "uut_PD_PA_Jac_233/SQ_SQ2_PD/A[166]_A[231]_AND_399_o1" (ROM) removed.
Unused block "uut_PD_PA_Jac_233/SQ_SQ2_PD/A[167]_A[230]_AND_632_o1" (ROM) removed.
Unused block "uut_PD_PA_Jac_233/SQ_SQ2_PD/A[168]_A[229]_AND_865_o1" (ROM) removed.
Unused block "uut_PD_PA_Jac_233/SQ_SQ2_PD/A[169]_A[228]_AND_1098_o1" (ROM) removed.
Unused block "uut_PD_PA_Jac_233/SQ_SQ2_PD/A[170]_A[227]_AND_1331_o1" (ROM) removed.
Unused block "uut_PD_PA_Jac_233/SQ_SQ2_PD/A[171]_A[226]_AND_1564_o1" (ROM) removed.
Unused block "uut_PD_PA_Jac_233/SQ_SQ2_PD/A[172]_A[225]_AND_1797_o1" (ROM) removed.
Unused block "uut_PD_PA_Jac_233/SQ_SQ2_PD/A[173]_A[224]_AND_2030_o1" (ROM) removed.
Unused block "uut_PD_PA_Jac_233/SQ_SQ2_PD/A[174]_A[223]_AND_2263_o1" (ROM) removed.
Unused block "uut_PD_PA_Jac_233/SQ_SQ2_PD/A[175]_A[222]_AND_2496_o1" (ROM) removed.
Unused block "uut_PD_PA_Jac_233/SQ_SQ2_PD/A[176]_A[221]_AND_2729_o1" (ROM) removed.
Unused block "uut_PD_PA_Jac_233/SQ_SQ2_PD/A[177]_A[220]_AND_2962_o1" (ROM) removed.
Unused block "uut_PD_PA_Jac_233/SQ_SQ2_PD/A[178]_A[219]_AND_3195_o1" (ROM) removed.
Unused block "uut_PD_PA_Jac_233/SQ_SQ2_PD/A[179]_A[218]_AND_3428_o1" (ROM) removed.
Unused block "uut_PD_PA_Jac_233/SQ_SQ2_PD/A[180]_A[217]_AND_3661_o1" (ROM) removed.
Unused block "uut_PD_PA_Jac_233/SQ_SQ2_PD/A[181]_A[216]_AND_3894_o1" (ROM) removed.
Unused block "uut_PD_PA_Jac_233/SQ_SQ2_PD/A[182]_A[215]_AND_4127_o1" (ROM) removed.
Unused block "uut_PD_PA_Jac_233/SQ_SQ2_PD/A[183]_A[214]_AND_4360_o1" (ROM) removed.
Unused block "uut_PD_PA_Jac_233/SQ_SQ2_PD/A[184]_A[213]_AND_4593_o1" (ROM) removed.
Unused block "uut_PD_PA_Jac_233/SQ_SQ2_PD/A[185]_A[212]_AND_4826_o1" (ROM) removed.
Unused block "uut_PD_PA_Jac_233/SQ_SQ2_PD/A[186]_A[211]_AND_5059_o1" (ROM) removed.
Unused block "uut_PD_PA_Jac_233/SQ_SQ2_PD/A[187]_A[210]_AND_5292_o1" (ROM)

```
removed.
Unused block "uut_PD_PA_Jac_233/SQ_SQ2_PD/A[188]_A[209]_AND_5525_o1" (ROM)
removed.
Unused block "uut_PD_PA_Jac_233/SQ_SQ4_PD/A[165]_A[232]_AND_166_o1" (ROM)
removed.
Unused block "uut_PD_PA_Jac_233/SQ_SQ4_PD/A[166]_A[231]_AND_399_o1" (ROM)
removed.
Unused block "uut_PD_PA_Jac_233/SQ_SQ4_PD/A[167]_A[230]_AND_632_o1" (ROM)
removed.
Unused block "uut_PD_PA_Jac_233/SQ_SQ4_PD/A[168]_A[229]_AND_865_o1" (ROM)
removed.
Unused block "uut_PD_PA_Jac_233/SQ_SQ4_PD/A[169]_A[228]_AND_1098_o1" (ROM)
removed.
Unused block "uut_PD_PA_Jac_233/SQ_SQ4_PD/A[170]_A[227]_AND_1331_o1" (ROM)
removed.
Unused block "uut_PD_PA_Jac_233/SQ_SQ4_PD/A[171]_A[226]_AND_1564_o1" (ROM)
removed.
Unused block "uut_PD_PA_Jac_233/SQ_SQ4_PD/A[172]_A[225]_AND_1797_o1" (ROM)
removed.
Unused block "uut_PD_PA_Jac_233/SQ_SQ4_PD/A[173]_A[224]_AND_2030_o1" (ROM)
removed.
Unused block "uut_PD_PA_Jac_233/SQ_SQ4_PD/A[174]_A[223]_AND_2263_o1" (ROM)
removed.
Unused block "uut_PD_PA_Jac_233/SQ_SQ4_PD/A[175]_A[222]_AND_2496_o1" (ROM)
removed.
Unused block "uut_PD_PA_Jac_233/SQ_SQ4_PD/A[176]_A[221]_AND_2729_o1" (ROM)
removed.
Unused block "uut_PD_PA_Jac_233/SQ_SQ4_PD/A[177]_A[220]_AND_2962_o1" (ROM)
removed.
Unused block "uut_PD_PA_Jac_233/SQ_SQ4_PD/A[178]_A[219]_AND_3195_o1" (ROM)
removed.
Unused block "uut_PD_PA_Jac_233/SQ_SQ4_PD/A[179]_A[218]_AND_3428_o1" (ROM)
removed.
Unused block "uut_PD_PA_Jac_233/SQ_SQ4_PD/A[180]_A[217]_AND_3661_o1" (ROM)
removed.
Unused block "uut_PD_PA_Jac_233/SQ_SQ4_PD/A[181]_A[216]_AND_3894_o1" (ROM)
removed.
Unused block "uut_PD_PA_Jac_233/SQ_SQ4_PD/A[182]_A[215]_AND_4127_o1" (ROM)
removed.
Unused block "uut_PD_PA_Jac_233/SQ_SQ4_PD/A[183]_A[214]_AND_4360_o1" (ROM)
removed.
Unused block "uut_PD_PA_Jac_233/SQ_SQ4_PD/A[184]_A[213]_AND_4593_o1" (ROM)
removed.
Unused block "uut_PD_PA_Jac_233/SQ_SQ4_PD/A[185]_A[212]_AND_4826_o1" (ROM)
removed.
Unused block "uut_PD_PA_Jac_233/SQ_SQ4_PD/A[186]_A[211]_AND_5059_o1" (ROM)
removed.
Unused block "uut_PD_PA_Jac_233/SQ_SQ4_PD/A[187]_A[210]_AND_5292_o1" (ROM)
removed.
Unused block "uut_PD_PA_Jac_233/SQ_SQ4_PD/A[188]_A[209]_AND_5525_o1" (ROM)
removed.

Optimized Block(s):
TYPE         BLOCK
GND          XST_GND
VCC          XST_VCC
GND          uut_PD_PA_Jac_233/SQ_SQ1_PA/XST_GND
GND          uut_PD_PA_Jac_233/SQ_SQ1_PD/XST_GND
VCC          uut_PD_PA_Jac_233/SQ_SQ1_PD/XST_VCC
GND          uut_PD_PA_Jac_233/SQ_SQ2_PA/XST_GND
GND          uut_PD_PA_Jac_233/SQ_SQ2_PD/XST_GND
GND          uut_PD_PA_Jac_233/SQ_SQ3_PD/XST_GND
GND          uut_PD_PA_Jac_233/SQ_SQ4_PD/XST_GND
GND          uut_PD_PA_Jac_233/SQ_SQ5_PD/XST_GND
GND          uut_PD_PA_Jac_233/XST_GND
VCC          uut_PD_PA_Jac_233/XST_VCC
GND          uut_PD_PA_Jac_233/mult_M2_PA/XST_GND
VCC          uut_PD_PA_Jac_233/mult_M2_PA/XST_VCC
GND          uut_PD_PA_Jac_233/mult_M3_PA/XST_GND
LUT4         uut_PD_PA_Jac_233/mult_M4_PD/Mxor_n55224_102_xo<0>1_SW0_SW0
   optimized to 0
```

```
LUT4         uut_PD_PA_Jac_233/mult_M4_PD/Mxor_n55224_119_xo<0>49_SW0_SW0
   optimized to 0
LUT4         uut_PD_PA_Jac_233/mult_M4_PD/Mxor_n55224_136_xo<0>56_SW0
   optimized to 0
LUT4         uut_PD_PA_Jac_233/mult_M4_PD/Mxor_n55224_170_xo<0>60_SW0_SW0_SW0
   optimized to 0
LUT4         uut_PD_PA_Jac_233/mult_M4_PD/Mxor_n55224_17_xo<0>3_SW0
   optimized to 0
LUT4         uut_PD_PA_Jac_233/mult_M4_PD/Mxor_n55224_34_xo<0>13_SW0
   optimized to 0
LUT4         uut_PD_PA_Jac_233/mult_M4_PD/Mxor_n55224_51_xo<0>10_SW0
   optimized to 0
LUT4         uut_PD_PA_Jac_233/mult_M4_PD/Mxor_n55224_85_xo<0>16_SW0
   optimized to 0
GND          uut_PD_PA_Jac_233/mult_M4_PD/XST_GND
VCC          uut_PD_PA_Jac_233/mult_M4_PD/XST_VCC
LUT5         uut_PD_PA_Jac_233/SQ_SQ1_PA/Mxor_GND_9_o_GND_9_o_xor_136_OUT_233_xo<0>12
   optimized to 0
LUT4         uut_PD_PA_Jac_233/SQ_SQ1_PA/Mxor_GND_9_o_GND_9_o_xor_136_OUT_233_xo<0>71
    Property STUCK_AT NOT found
LUT5         uut_PD_PA_Jac_233/SQ_SQ1_PD/Mxor_GND_9_o_GND_9_o_xor_136_OUT_233_xo<0>12
   optimized to 0
LUT4         uut_PD_PA_Jac_233/SQ_SQ1_PD/Mxor_GND_9_o_GND_9_o_xor_136_OUT_233_xo<0>71
    Property STUCK_AT NOT found
LUT5         uut_PD_PA_Jac_233/SQ_SQ2_PD/Mxor_GND_9_o_GND_9_o_xor_136_OUT_233_xo<0>12
   optimized to 0
LUT4         uut_PD_PA_Jac_233/SQ_SQ2_PD/Mxor_GND_9_o_GND_9_o_xor_136_OUT_233_xo<0>71
    Property STUCK_AT NOT found
LUT5
        uut_PD_PA_Jac_233/SQ_SQ2_PD/Mxor_GND_9_o_GND_9_o_xor_254_OUT_233_xo<0>23_SW0
   optimized to 0
LUT4
        uut_PD_PA_Jac_233/SQ_SQ2_PD/Mxor_GND_9_o_GND_9_o_xor_254_OUT_233_xo<0>23_SW0_S
W1
    Property STUCK_AT NOT found
LUT5         uut_PD_PA_Jac_233/SQ_SQ3_PD/Mxor_GND_9_o_GND_9_o_xor_188_OUT_233_xo<0>15
   optimized to 0
LUT4
        uut_PD_PA_Jac_233/SQ_SQ3_PD/Mxor_GND_9_o_GND_9_o_xor_188_OUT_233_xo<0>15_SW0
    Property STUCK_AT NOT found
LUT5         uut_PD_PA_Jac_233/SQ_SQ4_PD/Mxor_GND_9_o_GND_9_o_xor_136_OUT_233_xo<0>12
   optimized to 0
LUT4         uut_PD_PA_Jac_233/SQ_SQ4_PD/Mxor_GND_9_o_GND_9_o_xor_136_OUT_233_xo<0>71
    Property STUCK_AT NOT found
LUT6         uut_PD_PA_Jac_233/SQ_SQ4_PD/Mxor_GND_9_o_GND_9_o_xor_60_OUT_233_xo<0>4
   optimized to 0
LUT2         uut_PD_PA_Jac_233/SQ_SQ4_PD/A[204]_A[231]_AND_437_o1
    Property STUCK_AT NOT found
LUT2         uut_PD_PA_Jac_233/SQ_SQ4_PD/A[203]_A[232]_AND_204_o1
    Property STUCK_AT NOT found

To enable printing of redundant blocks removed and signals merged, set the
detailed map report option and rerun map.

Section 6 - IOB Properties
--------------------------

+-------------------------------------------------------------------------------------------------
-------------------------------------------------------------+
| IOB Name                          | Type             | Direction | IO Standard          |
Diff  | Drive    | Slew | Reg (s)      | Resistor | IOB      |
|                                   |                  |           |                      |
Term  | Strength | Rate |              |          | Delay    |
+-------------------------------------------------------------------------------------------------
-------------------------------------------------------------+
| QX<0>                             | IOB              | OUTPUT    | LVCMOS18
|       | 12       | SLOW |              |          |          |
| QX<1>                             | IOB              | OUTPUT    | LVCMOS18
|       | 12       | SLOW |              |          |          |
| QX<2>                             | IOB              | OUTPUT    | LVCMOS18
|       | 12       | SLOW |              |          |          |
```

```
| QX<3>                                  | IOB                | OUTPUT     | LVCMOS18
|       | 12        | SLOW |             |          |            |
| QX<4>                                  | IOB                | OUTPUT     | LVCMOS18
|       | 12        | SLOW |             |          |            |
| QX<5>                                  | IOB                | OUTPUT     | LVCMOS18
|       | 12        | SLOW |             |          |            |
| QX<6>                                  | IOB                | OUTPUT     | LVCMOS18
|       | 12        | SLOW |             |          |            |
| QX<7>                                  | IOB                | OUTPUT     | LVCMOS18
|       | 12        | SLOW |             |          |            |
| QX<8>                                  | IOB                | OUTPUT     | LVCMOS18
|       | 12        | SLOW |             |          |            |
| QX<9>                                  | IOB                | OUTPUT     | LVCMOS18
|       | 12        | SLOW |             |          |            |
| QX<10>                                 | IOB                | OUTPUT     | LVCMOS18
|       | 12        | SLOW |             |          |            |
| QX<11>                                 | IOB                | OUTPUT     | LVCMOS18
|       | 12        | SLOW |             |          |            |
| QX<12>                                 | IOB                | OUTPUT     | LVCMOS18
|       | 12        | SLOW |             |          |            |
| QX<13>                                 | IOB                | OUTPUT     | LVCMOS18
|       | 12        | SLOW |             |          |            |
| QX<14>                                 | IOB                | OUTPUT     | LVCMOS18
|       | 12        | SLOW |             |          |            |
| QX<15>                                 | IOB                | OUTPUT     | LVCMOS18
|       | 12        | SLOW |             |          |            |
| QX<16>                                 | IOB                | OUTPUT     | LVCMOS18
|       | 12        | SLOW |             |          |            |
| QX<17>                                 | IOB                | OUTPUT     | LVCMOS18
|       | 12        | SLOW |             |          |            |
| QX<18>                                 | IOB                | OUTPUT     | LVCMOS18
|       | 12        | SLOW |             |          |            |
| QX<19>                                 | IOB                | OUTPUT     | LVCMOS18
|       | 12        | SLOW |             |          |            |
| QX<20>                                 | IOB                | OUTPUT     | LVCMOS18
|       | 12        | SLOW |             |          |            |
| QX<21>                                 | IOB                | OUTPUT     | LVCMOS18
|       | 12        | SLOW |             |          |            |
| QX<22>                                 | IOB                | OUTPUT     | LVCMOS18
|       | 12        | SLOW |             |          |            |
| QX<23>                                 | IOB                | OUTPUT     | LVCMOS18
|       | 12        | SLOW |             |          |            |
| QX<24>                                 | IOB                | OUTPUT     | LVCMOS18
|       | 12        | SLOW |             |          |            |
| QX<25>                                 | IOB                | OUTPUT     | LVCMOS18
|       | 12        | SLOW |             |          |            |
| QX<26>                                 | IOB                | OUTPUT     | LVCMOS18
|       | 12        | SLOW |             |          |            |
| QX<27>                                 | IOB                | OUTPUT     | LVCMOS18
|       | 12        | SLOW |             |          |            |
| QX<28>                                 | IOB                | OUTPUT     | LVCMOS18
|       | 12        | SLOW |             |          |            |
| QX<29>                                 | IOB                | OUTPUT     | LVCMOS18
|       | 12        | SLOW |             |          |            |
| QX<30>                                 | IOB                | OUTPUT     | LVCMOS18
|       | 12        | SLOW |             |          |            |
| QX<31>                                 | IOB                | OUTPUT     | LVCMOS18
|       | 12        | SLOW |             |          |            |
| QX<32>                                 | IOB                | OUTPUT     | LVCMOS18
|       | 12        | SLOW |             |          |            |
| QX<33>                                 | IOB                | OUTPUT     | LVCMOS18
|       | 12        | SLOW |             |          |            |
| QX<34>                                 | IOB                | OUTPUT     | LVCMOS18
|       | 12        | SLOW |             |          |            |
| QX<35>                                 | IOB                | OUTPUT     | LVCMOS18
|       | 12        | SLOW |             |          |            |
| QX<36>                                 | IOB                | OUTPUT     | LVCMOS18
|       | 12        | SLOW |             |          |            |
| QX<37>                                 | IOB                | OUTPUT     | LVCMOS18
|       | 12        | SLOW |             |          |            |
| QX<38>                                 | IOB                | OUTPUT     | LVCMOS18
```

```
|        | 12          | SLOW |                          |               |            |
| QX<39>                               | IOB              | OUTPUT     | LVCMOS18
|        | 12          | SLOW |                          |               |            |
| QX<40>                               | IOB              | OUTPUT     | LVCMOS18
|        | 12          | SLOW |                          |               |            |
| QX<41>                               | IOB              | OUTPUT     | LVCMOS18
|        | 12          | SLOW |                          |               |            |
| QX<42>                               | IOB              | OUTPUT     | LVCMOS18
|        | 12          | SLOW |                          |               |            |
| QX<43>                               | IOB              | OUTPUT     | LVCMOS18
|        | 12          | SLOW |                          |               |            |
| QX<44>                               | IOB              | OUTPUT     | LVCMOS18
|        | 12          | SLOW |                          |               |            |
| QX<45>                               | IOB              | OUTPUT     | LVCMOS18
|        | 12          | SLOW |                          |               |            |
| QX<46>                               | IOB              | OUTPUT     | LVCMOS18
|        | 12          | SLOW |                          |               |            |
| QX<47>                               | IOB              | OUTPUT     | LVCMOS18
|        | 12          | SLOW |                          |               |            |
| QX<48>                               | IOB              | OUTPUT     | LVCMOS18
|        | 12          | SLOW |                          |               |            |
| QX<49>                               | IOB              | OUTPUT     | LVCMOS18
|        | 12          | SLOW |                          |               |            |
| QX<50>                               | IOB              | OUTPUT     | LVCMOS18
|        | 12          | SLOW |                          |               |            |
| QX<51>                               | IOB              | OUTPUT     | LVCMOS18
|        | 12          | SLOW |                          |               |            |
| QX<52>                               | IOB              | OUTPUT     | LVCMOS18
|        | 12          | SLOW |                          |               |            |
| QX<53>                               | IOB              | OUTPUT     | LVCMOS18
|        | 12          | SLOW |                          |               |            |
| QX<54>                               | IOB              | OUTPUT     | LVCMOS18
|        | 12          | SLOW |                          |               |            |
| QX<55>                               | IOB              | OUTPUT     | LVCMOS18
|        | 12          | SLOW |                          |               |            |
| QX<56>                               | IOB              | OUTPUT     | LVCMOS18
|        | 12          | SLOW |                          |               |            |
| QX<57>                               | IOB              | OUTPUT     | LVCMOS18
|        | 12          | SLOW |                          |               |            |
| QX<58>                               | IOB              | OUTPUT     | LVCMOS18
|        | 12          | SLOW |                          |               |            |
| QX<59>                               | IOB              | OUTPUT     | LVCMOS18
|        | 12          | SLOW |                          |               |            |
| QX<60>                               | IOB              | OUTPUT     | LVCMOS18
|        | 12          | SLOW |                          |               |            |
| QX<61>                               | IOB              | OUTPUT     | LVCMOS18
|        | 12          | SLOW |                          |               |            |
| QX<62>                               | IOB              | OUTPUT     | LVCMOS18
|        | 12          | SLOW |                          |               |            |
| QX<63>                               | IOB              | OUTPUT     | LVCMOS18
|        | 12          | SLOW |                          |               |            |
| QX<64>                               | IOB              | OUTPUT     | LVCMOS18
|        | 12          | SLOW |                          |               |            |
| QX<65>                               | IOB              | OUTPUT     | LVCMOS18
|        | 12          | SLOW |                          |               |            |
| QX<66>                               | IOB              | OUTPUT     | LVCMOS18
|        | 12          | SLOW |                          |               |            |
| QX<67>                               | IOB              | OUTPUT     | LVCMOS18
|        | 12          | SLOW |                          |               |            |
| QX<68>                               | IOB              | OUTPUT     | LVCMOS18
|        | 12          | SLOW |                          |               |            |
| QX<69>                               | IOB              | OUTPUT     | LVCMOS18
|        | 12          | SLOW |                          |               |            |
| QX<70>                               | IOB              | OUTPUT     | LVCMOS18
|        | 12          | SLOW |                          |               |            |
| QX<71>                               | IOB              | OUTPUT     | LVCMOS18
|        | 12          | SLOW |                          |               |            |
| QX<72>                               | IOB              | OUTPUT     | LVCMOS18
|        | 12          | SLOW |                          |               |            |
| QX<73>                               | IOB              | OUTPUT     | LVCMOS18
|        | 12          | SLOW |                          |               |            |
```

```
| QX<74>                                 | IOB                 | OUTPUT     | LVCMOS18
|        | 12         | SLOW |           |           |          |
| QX<75>                                 | IOB                 | OUTPUT     | LVCMOS18
|        | 12         | SLOW |           |           |          |
| QX<76>                                 | IOB                 | OUTPUT     | LVCMOS18
|        | 12         | SLOW |           |           |          |
| QX<77>                                 | IOB                 | OUTPUT     | LVCMOS18
|        | 12         | SLOW |           |           |          |
| QX<78>                                 | IOB                 | OUTPUT     | LVCMOS18
|        | 12         | SLOW |           |           |          |
| QX<79>                                 | IOB                 | OUTPUT     | LVCMOS18
|        | 12         | SLOW |           |           |          |
| QX<80>                                 | IOB                 | OUTPUT     | LVCMOS18
|        | 12         | SLOW |           |           |          |
| QX<81>                                 | IOB                 | OUTPUT     | LVCMOS18
|        | 12         | SLOW |           |           |          |
| QX<82>                                 | IOB                 | OUTPUT     | LVCMOS18
|        | 12         | SLOW |           |           |          |
| QX<83>                                 | IOB                 | OUTPUT     | LVCMOS18
|        | 12         | SLOW |           |           |          |
| QX<84>                                 | IOB                 | OUTPUT     | LVCMOS18
|        | 12         | SLOW |           |           |          |
| QX<85>                                 | IOB                 | OUTPUT     | LVCMOS18
|        | 12         | SLOW |           |           |          |
| QX<86>                                 | IOB                 | OUTPUT     | LVCMOS18
|        | 12         | SLOW |           |           |          |
| QX<87>                                 | IOB                 | OUTPUT     | LVCMOS18
|        | 12         | SLOW |           |           |          |
| QX<88>                                 | IOB                 | OUTPUT     | LVCMOS18
|        | 12         | SLOW |           |           |          |
| QX<89>                                 | IOB                 | OUTPUT     | LVCMOS18
|        | 12         | SLOW |           |           |          |
| QX<90>                                 | IOB                 | OUTPUT     | LVCMOS18
|        | 12         | SLOW |           |           |          |
| QX<91>                                 | IOB                 | OUTPUT     | LVCMOS18
|        | 12         | SLOW |           |           |          |
| QX<92>                                 | IOB                 | OUTPUT     | LVCMOS18
|        | 12         | SLOW |           |           |          |
| QX<93>                                 | IOB                 | OUTPUT     | LVCMOS18
|        | 12         | SLOW |           |           |          |
| QX<94>                                 | IOB                 | OUTPUT     | LVCMOS18
|        | 12         | SLOW |           |           |          |
| QX<95>                                 | IOB                 | OUTPUT     | LVCMOS18
|        | 12         | SLOW |           |           |          |
| QX<96>                                 | IOB                 | OUTPUT     | LVCMOS18
|        | 12         | SLOW |           |           |          |
| QX<97>                                 | IOB                 | OUTPUT     | LVCMOS18
|        | 12         | SLOW |           |           |          |
| QX<98>                                 | IOB                 | OUTPUT     | LVCMOS18
|        | 12         | SLOW |           |           |          |
| QX<99>                                 | IOB                 | OUTPUT     | LVCMOS18
|        | 12         | SLOW |           |           |          |
| QX<100>                                | IOB                 | OUTPUT     | LVCMOS18
|        | 12         | SLOW |           |           |          |
| QX<101>                                | IOB                 | OUTPUT     | LVCMOS18
|        | 12         | SLOW |           |           |          |
| QX<102>                                | IOB                 | OUTPUT     | LVCMOS18
|        | 12         | SLOW |           |           |          |
| QX<103>                                | IOB                 | OUTPUT     | LVCMOS18
|        | 12         | SLOW |           |           |          |
| QX<104>                                | IOB                 | OUTPUT     | LVCMOS18
|        | 12         | SLOW |           |           |          |
| QX<105>                                | IOB                 | OUTPUT     | LVCMOS18
|        | 12         | SLOW |           |           |          |
| QX<106>                                | IOB                 | OUTPUT     | LVCMOS18
|        | 12         | SLOW |           |           |          |
| QX<107>                                | IOB                 | OUTPUT     | LVCMOS18
|        | 12         | SLOW |           |           |          |
| QX<108>                                | IOB                 | OUTPUT     | LVCMOS18
|        | 12         | SLOW |           |           |          |
| QX<109>                                | IOB                 | OUTPUT     | LVCMOS18
```

```
|        | 12        | SLOW |                    |           |           |
| QX<110>                            | IOB              | OUTPUT     | LVCMOS18
|        | 12        | SLOW |                    |           |           |
| QX<111>                            | IOB              | OUTPUT     | LVCMOS18
|        | 12        | SLOW |                    |           |           |
| QX<112>                            | IOB              | OUTPUT     | LVCMOS18
|        | 12        | SLOW |                    |           |           |
| QX<113>                            | IOB              | OUTPUT     | LVCMOS18
|        | 12        | SLOW |                    |           |           |
| QX<114>                            | IOB              | OUTPUT     | LVCMOS18
|        | 12        | SLOW |                    |           |           |
| QX<115>                            | IOB              | OUTPUT     | LVCMOS18
|        | 12        | SLOW |                    |           |           |
| QX<116>                            | IOB              | OUTPUT     | LVCMOS18
|        | 12        | SLOW |                    |           |           |
| QX<117>                            | IOB              | OUTPUT     | LVCMOS18
|        | 12        | SLOW |                    |           |           |
| QX<118>                            | IOB              | OUTPUT     | LVCMOS18
|        | 12        | SLOW |                    |           |           |
| QX<119>                            | IOB              | OUTPUT     | LVCMOS18
|        | 12        | SLOW |                    |           |           |
| QX<120>                            | IOB              | OUTPUT     | LVCMOS18
|        | 12        | SLOW |                    |           |           |
| QX<121>                            | IOB              | OUTPUT     | LVCMOS18
|        | 12        | SLOW |                    |           |           |
| QX<122>                            | IOB              | OUTPUT     | LVCMOS18
|        | 12        | SLOW |                    |           |           |
| QX<123>                            | IOB              | OUTPUT     | LVCMOS18
|        | 12        | SLOW |                    |           |           |
| QX<124>                            | IOB              | OUTPUT     | LVCMOS18
|        | 12        | SLOW |                    |           |           |
| QX<125>                            | IOB              | OUTPUT     | LVCMOS18
|        | 12        | SLOW |                    |           |           |
| QX<126>                            | IOB              | OUTPUT     | LVCMOS18
|        | 12        | SLOW |                    |           |           |
| QX<127>                            | IOB              | OUTPUT     | LVCMOS18
|        | 12        | SLOW |                    |           |           |
| QX<128>                            | IOB              | OUTPUT     | LVCMOS18
|        | 12        | SLOW |                    |           |           |
| QX<129>                            | IOB              | OUTPUT     | LVCMOS18
|        | 12        | SLOW |                    |           |           |
| QX<130>                            | IOB              | OUTPUT     | LVCMOS18
|        | 12        | SLOW |                    |           |           |
| QX<131>                            | IOB              | OUTPUT     | LVCMOS18
|        | 12        | SLOW |                    |           |           |
| QX<132>                            | IOB              | OUTPUT     | LVCMOS18
|        | 12        | SLOW |                    |           |           |
| QX<133>                            | IOB              | OUTPUT     | LVCMOS18
|        | 12        | SLOW |                    |           |           |
| QX<134>                            | IOB              | OUTPUT     | LVCMOS18
|        | 12        | SLOW |                    |           |           |
| QX<135>                            | IOB              | OUTPUT     | LVCMOS18
|        | 12        | SLOW |                    |           |           |
| QX<136>                            | IOB              | OUTPUT     | LVCMOS18
|        | 12        | SLOW |                    |           |           |
| QX<137>                            | IOB              | OUTPUT     | LVCMOS18
|        | 12        | SLOW |                    |           |           |
| QX<138>                            | IOB              | OUTPUT     | LVCMOS18
|        | 12        | SLOW |                    |           |           |
| QX<139>                            | IOB              | OUTPUT     | LVCMOS18
|        | 12        | SLOW |                    |           |           |
| QX<140>                            | IOB              | OUTPUT     | LVCMOS18
|        | 12        | SLOW |                    |           |           |
| QX<141>                            | IOB              | OUTPUT     | LVCMOS18
|        | 12        | SLOW |                    |           |           |
| QX<142>                            | IOB              | OUTPUT     | LVCMOS18
|        | 12        | SLOW |                    |           |           |
| QX<143>                            | IOB              | OUTPUT     | LVCMOS18
|        | 12        | SLOW |                    |           |           |
| QX<144>                            | IOB              | OUTPUT     | LVCMOS18
|        | 12        | SLOW |                    |           |           |
```

```
| QX<145>                              | IOB              | OUTPUT    | LVCMOS18
|      | 12       | SLOW |              |            |           |
| QX<146>                              | IOB              | OUTPUT    | LVCMOS18
|      | 12       | SLOW |              |            |           |
| QX<147>                              | IOB              | OUTPUT    | LVCMOS18
|      | 12       | SLOW |              |            |           |
| QX<148>                              | IOB              | OUTPUT    | LVCMOS18
|      | 12       | SLOW |              |            |           |
| QX<149>                              | IOB              | OUTPUT    | LVCMOS18
|      | 12       | SLOW |              |            |           |
| QX<150>                              | IOB              | OUTPUT    | LVCMOS18
|      | 12       | SLOW |              |            |           |
| QX<151>                              | IOB              | OUTPUT    | LVCMOS18
|      | 12       | SLOW |              |            |           |
| QX<152>                              | IOB              | OUTPUT    | LVCMOS18
|      | 12       | SLOW |              |            |           |
| QX<153>                              | IOB              | OUTPUT    | LVCMOS18
|      | 12       | SLOW |              |            |           |
| QX<154>                              | IOB              | OUTPUT    | LVCMOS18
|      | 12       | SLOW |              |            |           |
| QX<155>                              | IOB              | OUTPUT    | LVCMOS18
|      | 12       | SLOW |              |            |           |
| QX<156>                              | IOB              | OUTPUT    | LVCMOS18
|      | 12       | SLOW |              |            |           |
| QX<157>                              | IOB              | OUTPUT    | LVCMOS18
|      | 12       | SLOW |              |            |           |
| QX<158>                              | IOB              | OUTPUT    | LVCMOS18
|      | 12       | SLOW |              |            |           |
| QX<159>                              | IOB              | OUTPUT    | LVCMOS18
|      | 12       | SLOW |              |            |           |
| QX<160>                              | IOB              | OUTPUT    | LVCMOS18
|      | 12       | SLOW |              |            |           |
| QX<161>                              | IOB              | OUTPUT    | LVCMOS18
|      | 12       | SLOW |              |            |           |
| QX<162>                              | IOB              | OUTPUT    | LVCMOS18
|      | 12       | SLOW |              |            |           |
| QX<163>                              | IOB              | OUTPUT    | LVCMOS18
|      | 12       | SLOW |              |            |           |
| QX<164>                              | IOB              | OUTPUT    | LVCMOS18
|      | 12       | SLOW |              |            |           |
| QX<165>                              | IOB              | OUTPUT    | LVCMOS18
|      | 12       | SLOW |              |            |           |
| QX<166>                              | IOB              | OUTPUT    | LVCMOS18
|      | 12       | SLOW |              |            |           |
| QX<167>                              | IOB              | OUTPUT    | LVCMOS18
|      | 12       | SLOW |              |            |           |
| QX<168>                              | IOB              | OUTPUT    | LVCMOS18
|      | 12       | SLOW |              |            |           |
| QX<169>                              | IOB              | OUTPUT    | LVCMOS18
|      | 12       | SLOW |              |            |           |
| QX<170>                              | IOB              | OUTPUT    | LVCMOS18
|      | 12       | SLOW |              |            |           |
| QX<171>                              | IOB              | OUTPUT    | LVCMOS18
|      | 12       | SLOW |              |            |           |
| QX<172>                              | IOB              | OUTPUT    | LVCMOS18
|      | 12       | SLOW |              |            |           |
| QX<173>                              | IOB              | OUTPUT    | LVCMOS18
|      | 12       | SLOW |              |            |           |
| QX<174>                              | IOB              | OUTPUT    | LVCMOS18
|      | 12       | SLOW |              |            |           |
| QX<175>                              | IOB              | OUTPUT    | LVCMOS18
|      | 12       | SLOW |              |            |           |
| QX<176>                              | IOB              | OUTPUT    | LVCMOS18
|      | 12       | SLOW |              |            |           |
| QX<177>                              | IOB              | OUTPUT    | LVCMOS18
|      | 12       | SLOW |              |            |           |
| QX<178>                              | IOB              | OUTPUT    | LVCMOS18
|      | 12       | SLOW |              |            |           |
| QX<179>                              | IOB              | OUTPUT    | LVCMOS18
|      | 12       | SLOW |              |            |           |
| QX<180>                              | IOB              | OUTPUT    | LVCMOS18
```

```
|        | 12        | SLOW |                   |          |          |
| QX<181>                           | IOB               | OUTPUT    | LVCMOS18
|        | 12        | SLOW |                   |          |          |
| QX<182>                           | IOB               | OUTPUT    | LVCMOS18
|        | 12        | SLOW |                   |          |          |
| QX<183>                           | IOB               | OUTPUT    | LVCMOS18
|        | 12        | SLOW |                   |          |          |
| QX<184>                           | IOB               | OUTPUT    | LVCMOS18
|        | 12        | SLOW |                   |          |          |
| QX<185>                           | IOB               | OUTPUT    | LVCMOS18
|        | 12        | SLOW |                   |          |          |
| QX<186>                           | IOB               | OUTPUT    | LVCMOS18
|        | 12        | SLOW |                   |          |          |
| QX<187>                           | IOB               | OUTPUT    | LVCMOS18
|        | 12        | SLOW |                   |          |          |
| QX<188>                           | IOB               | OUTPUT    | LVCMOS18
|        | 12        | SLOW |                   |          |          |
| QX<189>                           | IOB               | OUTPUT    | LVCMOS18
|        | 12        | SLOW |                   |          |          |
| QX<190>                           | IOB               | OUTPUT    | LVCMOS18
|        | 12        | SLOW |                   |          |          |
| QX<191>                           | IOB               | OUTPUT    | LVCMOS18
|        | 12        | SLOW |                   |          |          |
| QX<192>                           | IOB               | OUTPUT    | LVCMOS18
|        | 12        | SLOW |                   |          |          |
| QX<193>                           | IOB               | OUTPUT    | LVCMOS18
|        | 12        | SLOW |                   |          |          |
| QX<194>                           | IOB               | OUTPUT    | LVCMOS18
|        | 12        | SLOW |                   |          |          |
| QX<195>                           | IOB               | OUTPUT    | LVCMOS18
|        | 12        | SLOW |                   |          |          |
| QX<196>                           | IOB               | OUTPUT    | LVCMOS18
|        | 12        | SLOW |                   |          |          |
| QX<197>                           | IOB               | OUTPUT    | LVCMOS18
|        | 12        | SLOW |                   |          |          |
| QX<198>                           | IOB               | OUTPUT    | LVCMOS18
|        | 12        | SLOW |                   |          |          |
| QX<199>                           | IOB               | OUTPUT    | LVCMOS18
|        | 12        | SLOW |                   |          |          |
| QX<200>                           | IOB               | OUTPUT    | LVCMOS18
|        | 12        | SLOW |                   |          |          |
| QX<201>                           | IOB               | OUTPUT    | LVCMOS18
|        | 12        | SLOW |                   |          |          |
| QX<202>                           | IOB               | OUTPUT    | LVCMOS18
|        | 12        | SLOW |                   |          |          |
| QX<203>                           | IOB               | OUTPUT    | LVCMOS18
|        | 12        | SLOW |                   |          |          |
| QX<204>                           | IOB               | OUTPUT    | LVCMOS18
|        | 12        | SLOW |                   |          |          |
| QX<205>                           | IOB               | OUTPUT    | LVCMOS18
|        | 12        | SLOW |                   |          |          |
| QX<206>                           | IOB               | OUTPUT    | LVCMOS18
|        | 12        | SLOW |                   |          |          |
| QX<207>                           | IOB               | OUTPUT    | LVCMOS18
|        | 12        | SLOW |                   |          |          |
| QX<208>                           | IOB               | OUTPUT    | LVCMOS18
|        | 12        | SLOW |                   |          |          |
| QX<209>                           | IOB               | OUTPUT    | LVCMOS18
|        | 12        | SLOW |                   |          |          |
| QX<210>                           | IOB               | OUTPUT    | LVCMOS18
|        | 12        | SLOW |                   |          |          |
| QX<211>                           | IOB               | OUTPUT    | LVCMOS18
|        | 12        | SLOW |                   |          |          |
| QX<212>                           | IOB               | OUTPUT    | LVCMOS18
|        | 12        | SLOW |                   |          |          |
| QX<213>                           | IOB               | OUTPUT    | LVCMOS18
|        | 12        | SLOW |                   |          |          |
| QX<214>                           | IOB               | OUTPUT    | LVCMOS18
|        | 12        | SLOW |                   |          |          |
| QX<215>                           | IOB               | OUTPUT    | LVCMOS18
|        | 12        | SLOW |                   |          |          |
```

```
| QX<216>                              | IOB                | OUTPUT      | LVCMOS18
|       | 12        | SLOW |                    |             |            |
| QX<217>                              | IOB                | OUTPUT      | LVCMOS18
|       | 12        | SLOW |                    |             |            |
| QX<218>                              | IOB                | OUTPUT      | LVCMOS18
|       | 12        | SLOW |                    |             |            |
| QX<219>                              | IOB                | OUTPUT      | LVCMOS18
|       | 12        | SLOW |                    |             |            |
| QX<220>                              | IOB                | OUTPUT      | LVCMOS18
|       | 12        | SLOW |                    |             |            |
| QX<221>                              | IOB                | OUTPUT      | LVCMOS18
|       | 12        | SLOW |                    |             |            |
| QX<222>                              | IOB                | OUTPUT      | LVCMOS18
|       | 12        | SLOW |                    |             |            |
| QX<223>                              | IOB                | OUTPUT      | LVCMOS18
|       | 12        | SLOW |                    |             |            |
| QX<224>                              | IOB                | OUTPUT      | LVCMOS18
|       | 12        | SLOW |                    |             |            |
| QX<225>                              | IOB                | OUTPUT      | LVCMOS18
|       | 12        | SLOW |                    |             |            |
| QX<226>                              | IOB                | OUTPUT      | LVCMOS18
|       | 12        | SLOW |                    |             |            |
| QX<227>                              | IOB                | OUTPUT      | LVCMOS18
|       | 12        | SLOW |                    |             |            |
| QX<228>                              | IOB                | OUTPUT      | LVCMOS18
|       | 12        | SLOW |                    |             |            |
| QX<229>                              | IOB                | OUTPUT      | LVCMOS18
|       | 12        | SLOW |                    |             |            |
| QX<230>                              | IOB                | OUTPUT      | LVCMOS18
|       | 12        | SLOW |                    |             |            |
| QX<231>                              | IOB                | OUTPUT      | LVCMOS18
|       | 12        | SLOW |                    |             |            |
| QX<232>                              | IOB                | OUTPUT      | LVCMOS18
|       | 12        | SLOW |                    |             |            |
| QY<0>                                | IOB                | OUTPUT      | LVCMOS18
|       | 12        | SLOW |                    |             |            |
| QY<1>                                | IOB                | OUTPUT      | LVCMOS18
|       | 12        | SLOW |                    |             |            |
| QY<2>                                | IOB                | OUTPUT      | LVCMOS18
|       | 12        | SLOW |                    |             |            |
| QY<3>                                | IOB                | OUTPUT      | LVCMOS18
|       | 12        | SLOW |                    |             |            |
| QY<4>                                | IOB                | OUTPUT      | LVCMOS18
|       | 12        | SLOW |                    |             |            |
| QY<5>                                | IOB                | OUTPUT      | LVCMOS18
|       | 12        | SLOW |                    |             |            |
| QY<6>                                | IOB                | OUTPUT      | LVCMOS18
|       | 12        | SLOW |                    |             |            |
| QY<7>                                | IOB                | OUTPUT      | LVCMOS18
|       | 12        | SLOW |                    |             |            |
| QY<8>                                | IOB                | OUTPUT      | LVCMOS18
|       | 12        | SLOW |                    |             |            |
| QY<9>                                | IOB                | OUTPUT      | LVCMOS18
|       | 12        | SLOW |                    |             |            |
| QY<10>                               | IOB                | OUTPUT      | LVCMOS18
|       | 12        | SLOW |                    |             |            |
| QY<11>                               | IOB                | OUTPUT      | LVCMOS18
|       | 12        | SLOW |                    |             |            |
| QY<12>                               | IOB                | OUTPUT      | LVCMOS18
|       | 12        | SLOW |                    |             |            |
| QY<13>                               | IOB                | OUTPUT      | LVCMOS18
|       | 12        | SLOW |                    |             |            |
| QY<14>                               | IOB                | OUTPUT      | LVCMOS18
|       | 12        | SLOW |                    |             |            |
| QY<15>                               | IOB                | OUTPUT      | LVCMOS18
|       | 12        | SLOW |                    |             |            |
| QY<16>                               | IOB                | OUTPUT      | LVCMOS18
|       | 12        | SLOW |                    |             |            |
| QY<17>                               | IOB                | OUTPUT      | LVCMOS18
|       | 12        | SLOW |                    |             |            |
| QY<18>                               | IOB                | OUTPUT      | LVCMOS18
```

```
|        | 12        | SLOW |                    |           |          |
| QY<19>                                | IOB              | OUTPUT     | LVCMOS18
|        | 12        | SLOW |                    |           |          |
| QY<20>                                | IOB              | OUTPUT     | LVCMOS18
|        | 12        | SLOW |                    |           |          |
| QY<21>                                | IOB              | OUTPUT     | LVCMOS18
|        | 12        | SLOW |                    |           |          |
| QY<22>                                | IOB              | OUTPUT     | LVCMOS18
|        | 12        | SLOW |                    |           |          |
| QY<23>                                | IOB              | OUTPUT     | LVCMOS18
|        | 12        | SLOW |                    |           |          |
| QY<24>                                | IOB              | OUTPUT     | LVCMOS18
|        | 12        | SLOW |                    |           |          |
| QY<25>                                | IOB              | OUTPUT     | LVCMOS18
|        | 12        | SLOW |                    |           |          |
| QY<26>                                | IOB              | OUTPUT     | LVCMOS18
|        | 12        | SLOW |                    |           |          |
| QY<27>                                | IOB              | OUTPUT     | LVCMOS18
|        | 12        | SLOW |                    |           |          |
| QY<28>                                | IOB              | OUTPUT     | LVCMOS18
|        | 12        | SLOW |                    |           |          |
| QY<29>                                | IOB              | OUTPUT     | LVCMOS18
|        | 12        | SLOW |                    |           |          |
| QY<30>                                | IOB              | OUTPUT     | LVCMOS18
|        | 12        | SLOW |                    |           |          |
| QY<31>                                | IOB              | OUTPUT     | LVCMOS18
|        | 12        | SLOW |                    |           |          |
| QY<32>                                | IOB              | OUTPUT     | LVCMOS18
|        | 12        | SLOW |                    |           |          |
| QY<33>                                | IOB              | OUTPUT     | LVCMOS18
|        | 12        | SLOW |                    |           |          |
| QY<34>                                | IOB              | OUTPUT     | LVCMOS18
|        | 12        | SLOW |                    |           |          |
| QY<35>                                | IOB              | OUTPUT     | LVCMOS18
|        | 12        | SLOW |                    |           |          |
| QY<36>                                | IOB              | OUTPUT     | LVCMOS18
|        | 12        | SLOW |                    |           |          |
| QY<37>                                | IOB              | OUTPUT     | LVCMOS18
|        | 12        | SLOW |                    |           |          |
| QY<38>                                | IOB              | OUTPUT     | LVCMOS18
|        | 12        | SLOW |                    |           |          |
| QY<39>                                | IOB              | OUTPUT     | LVCMOS18
|        | 12        | SLOW |                    |           |          |
| QY<40>                                | IOB              | OUTPUT     | LVCMOS18
|        | 12        | SLOW |                    |           |          |
| QY<41>                                | IOB              | OUTPUT     | LVCMOS18
|        | 12        | SLOW |                    |           |          |
| QY<42>                                | IOB              | OUTPUT     | LVCMOS18
|        | 12        | SLOW |                    |           |          |
| QY<43>                                | IOB              | OUTPUT     | LVCMOS18
|        | 12        | SLOW |                    |           |          |
| QY<44>                                | IOB              | OUTPUT     | LVCMOS18
|        | 12        | SLOW |                    |           |          |
| QY<45>                                | IOB              | OUTPUT     | LVCMOS18
|        | 12        | SLOW |                    |           |          |
| QY<46>                                | IOB              | OUTPUT     | LVCMOS18
|        | 12        | SLOW |                    |           |          |
| QY<47>                                | IOB              | OUTPUT     | LVCMOS18
|        | 12        | SLOW |                    |           |          |
| QY<48>                                | IOB              | OUTPUT     | LVCMOS18
|        | 12        | SLOW |                    |           |          |
| QY<49>                                | IOB              | OUTPUT     | LVCMOS18
|        | 12        | SLOW |                    |           |          |
| QY<50>                                | IOB              | OUTPUT     | LVCMOS18
|        | 12        | SLOW |                    |           |          |
| QY<51>                                | IOB              | OUTPUT     | LVCMOS18
|        | 12        | SLOW |                    |           |          |
| QY<52>                                | IOB              | OUTPUT     | LVCMOS18
|        | 12        | SLOW |                    |           |          |
| QY<53>                                | IOB              | OUTPUT     | LVCMOS18
|        | 12        | SLOW |                    |           |          |
```

```
| QY<54>                                 | IOB              | OUTPUT    | LVCMOS18
|       | 12        | SLOW |                |          |           |
| QY<55>                                 | IOB              | OUTPUT    | LVCMOS18
|       | 12        | SLOW |                |          |           |
| QY<56>                                 | IOB              | OUTPUT    | LVCMOS18
|       | 12        | SLOW |                |          |           |
| QY<57>                                 | IOB              | OUTPUT    | LVCMOS18
|       | 12        | SLOW |                |          |           |
| QY<58>                                 | IOB              | OUTPUT    | LVCMOS18
|       | 12        | SLOW |                |          |           |
| QY<59>                                 | IOB              | OUTPUT    | LVCMOS18
|       | 12        | SLOW |                |          |           |
| QY<60>                                 | IOB              | OUTPUT    | LVCMOS18
|       | 12        | SLOW |                |          |           |
| QY<61>                                 | IOB              | OUTPUT    | LVCMOS18
|       | 12        | SLOW |                |          |           |
| QY<62>                                 | IOB              | OUTPUT    | LVCMOS18
|       | 12        | SLOW |                |          |           |
| QY<63>                                 | IOB              | OUTPUT    | LVCMOS18
|       | 12        | SLOW |                |          |           |
| QY<64>                                 | IOB              | OUTPUT    | LVCMOS18
|       | 12        | SLOW |                |          |           |
| QY<65>                                 | IOB              | OUTPUT    | LVCMOS18
|       | 12        | SLOW |                |          |           |
| QY<66>                                 | IOB              | OUTPUT    | LVCMOS18
|       | 12        | SLOW |                |          |           |
| QY<67>                                 | IOB              | OUTPUT    | LVCMOS18
|       | 12        | SLOW |                |          |           |
| QY<68>                                 | IOB              | OUTPUT    | LVCMOS18
|       | 12        | SLOW |                |          |           |
| QY<69>                                 | IOB              | OUTPUT    | LVCMOS18
|       | 12        | SLOW |                |          |           |
| QY<70>                                 | IOB              | OUTPUT    | LVCMOS18
|       | 12        | SLOW |                |          |           |
| QY<71>                                 | IOB              | OUTPUT    | LVCMOS18
|       | 12        | SLOW |                |          |           |
| QY<72>                                 | IOB              | OUTPUT    | LVCMOS18
|       | 12        | SLOW |                |          |           |
| QY<73>                                 | IOB              | OUTPUT    | LVCMOS18
|       | 12        | SLOW |                |          |           |
| QY<74>                                 | IOB              | OUTPUT    | LVCMOS18
|       | 12        | SLOW |                |          |           |
| QY<75>                                 | IOB              | OUTPUT    | LVCMOS18
|       | 12        | SLOW |                |          |           |
| QY<76>                                 | IOB              | OUTPUT    | LVCMOS18
|       | 12        | SLOW |                |          |           |
| QY<77>                                 | IOB              | OUTPUT    | LVCMOS18
|       | 12        | SLOW |                |          |           |
| QY<78>                                 | IOB              | OUTPUT    | LVCMOS18
|       | 12        | SLOW |                |          |           |
| QY<79>                                 | IOB              | OUTPUT    | LVCMOS18
|       | 12        | SLOW |                |          |           |
| QY<80>                                 | IOB              | OUTPUT    | LVCMOS18
|       | 12        | SLOW |                |          |           |
| QY<81>                                 | IOB              | OUTPUT    | LVCMOS18
|       | 12        | SLOW |                |          |           |
| QY<82>                                 | IOB              | OUTPUT    | LVCMOS18
|       | 12        | SLOW |                |          |           |
| QY<83>                                 | IOB              | OUTPUT    | LVCMOS18
|       | 12        | SLOW |                |          |           |
| QY<84>                                 | IOB              | OUTPUT    | LVCMOS18
|       | 12        | SLOW |                |          |           |
| QY<85>                                 | IOB              | OUTPUT    | LVCMOS18
|       | 12        | SLOW |                |          |           |
| QY<86>                                 | IOB              | OUTPUT    | LVCMOS18
|       | 12        | SLOW |                |          |           |
| QY<87>                                 | IOB              | OUTPUT    | LVCMOS18
|       | 12        | SLOW |                |          |           |
| QY<88>                                 | IOB              | OUTPUT    | LVCMOS18
|       | 12        | SLOW |                |          |           |
| QY<89>                                 | IOB              | OUTPUT    | LVCMOS18
```

```
|        | 12       | SLOW |                 |          |         |
| QY<90>                              | IOB              | OUTPUT    | LVCMOS18
|        | 12       | SLOW |                 |          |         |
| QY<91>                              | IOB              | OUTPUT    | LVCMOS18
|        | 12       | SLOW |                 |          |         |
| QY<92>                              | IOB              | OUTPUT    | LVCMOS18
|        | 12       | SLOW |                 |          |         |
| QY<93>                              | IOB              | OUTPUT    | LVCMOS18
|        | 12       | SLOW |                 |          |         |
| QY<94>                              | IOB              | OUTPUT    | LVCMOS18
|        | 12       | SLOW |                 |          |         |
| QY<95>                              | IOB              | OUTPUT    | LVCMOS18
|        | 12       | SLOW |                 |          |         |
| QY<96>                              | IOB              | OUTPUT    | LVCMOS18
|        | 12       | SLOW |                 |          |         |
| QY<97>                              | IOB              | OUTPUT    | LVCMOS18
|        | 12       | SLOW |                 |          |         |
| QY<98>                              | IOB              | OUTPUT    | LVCMOS18
|        | 12       | SLOW |                 |          |         |
| QY<99>                              | IOB              | OUTPUT    | LVCMOS18
|        | 12       | SLOW |                 |          |         |
| QY<100>                             | IOB              | OUTPUT    | LVCMOS18
|        | 12       | SLOW |                 |          |         |
| QY<101>                             | IOB              | OUTPUT    | LVCMOS18
|        | 12       | SLOW |                 |          |         |
| QY<102>                             | IOB              | OUTPUT    | LVCMOS18
|        | 12       | SLOW |                 |          |         |
| QY<103>                             | IOB              | OUTPUT    | LVCMOS18
|        | 12       | SLOW |                 |          |         |
| QY<104>                             | IOB              | OUTPUT    | LVCMOS18
|        | 12       | SLOW |                 |          |         |
| QY<105>                             | IOB              | OUTPUT    | LVCMOS18
|        | 12       | SLOW |                 |          |         |
| QY<106>                             | IOB              | OUTPUT    | LVCMOS18
|        | 12       | SLOW |                 |          |         |
| QY<107>                             | IOB              | OUTPUT    | LVCMOS18
|        | 12       | SLOW |                 |          |         |
| QY<108>                             | IOB              | OUTPUT    | LVCMOS18
|        | 12       | SLOW |                 |          |         |
| QY<109>                             | IOB              | OUTPUT    | LVCMOS18
|        | 12       | SLOW |                 |          |         |
| QY<110>                             | IOB              | OUTPUT    | LVCMOS18
|        | 12       | SLOW |                 |          |         |
| QY<111>                             | IOB              | OUTPUT    | LVCMOS18
|        | 12       | SLOW |                 |          |         |
| QY<112>                             | IOB              | OUTPUT    | LVCMOS18
|        | 12       | SLOW |                 |          |         |
| QY<113>                             | IOB              | OUTPUT    | LVCMOS18
|        | 12       | SLOW |                 |          |         |
| QY<114>                             | IOB              | OUTPUT    | LVCMOS18
|        | 12       | SLOW |                 |          |         |
| QY<115>                             | IOB              | OUTPUT    | LVCMOS18
|        | 12       | SLOW |                 |          |         |
| QY<116>                             | IOB              | OUTPUT    | LVCMOS18
|        | 12       | SLOW |                 |          |         |
| QY<117>                             | IOB              | OUTPUT    | LVCMOS18
|        | 12       | SLOW |                 |          |         |
| QY<118>                             | IOB              | OUTPUT    | LVCMOS18
|        | 12       | SLOW |                 |          |         |
| QY<119>                             | IOB              | OUTPUT    | LVCMOS18
|        | 12       | SLOW |                 |          |         |
| QY<120>                             | IOB              | OUTPUT    | LVCMOS18
|        | 12       | SLOW |                 |          |         |
| QY<121>                             | IOB              | OUTPUT    | LVCMOS18
|        | 12       | SLOW |                 |          |         |
| QY<122>                             | IOB              | OUTPUT    | LVCMOS18
|        | 12       | SLOW |                 |          |         |
| QY<123>                             | IOB              | OUTPUT    | LVCMOS18
|        | 12       | SLOW |                 |          |         |
| QY<124>                             | IOB              | OUTPUT    | LVCMOS18
|        | 12       | SLOW |                 |          |         |
```

```
| QY<125>                              | IOB                  | OUTPUT     | LVCMOS18
|        | 12        | SLOW |           |            |            |
| QY<126>                              | IOB                  | OUTPUT     | LVCMOS18
|        | 12        | SLOW |           |            |            |
| QY<127>                              | IOB                  | OUTPUT     | LVCMOS18
|        | 12        | SLOW |           |            |            |
| QY<128>                              | IOB                  | OUTPUT     | LVCMOS18
|        | 12        | SLOW |           |            |            |
| QY<129>                              | IOB                  | OUTPUT     | LVCMOS18
|        | 12        | SLOW |           |            |            |
| QY<130>                              | IOB                  | OUTPUT     | LVCMOS18
|        | 12        | SLOW |           |            |            |
| QY<131>                              | IOB                  | OUTPUT     | LVCMOS18
|        | 12        | SLOW |           |            |            |
| QY<132>                              | IOB                  | OUTPUT     | LVCMOS18
|        | 12        | SLOW |           |            |            |
| QY<133>                              | IOB                  | OUTPUT     | LVCMOS18
|        | 12        | SLOW |           |            |            |
| QY<134>                              | IOB                  | OUTPUT     | LVCMOS18
|        | 12        | SLOW |           |            |            |
| QY<135>                              | IOB                  | OUTPUT     | LVCMOS18
|        | 12        | SLOW |           |            |            |
| QY<136>                              | IOB                  | OUTPUT     | LVCMOS18
|        | 12        | SLOW |           |            |            |
| QY<137>                              | IOB                  | OUTPUT     | LVCMOS18
|        | 12        | SLOW |           |            |            |
| QY<138>                              | IOB                  | OUTPUT     | LVCMOS18
|        | 12        | SLOW |           |            |            |
| QY<139>                              | IOB                  | OUTPUT     | LVCMOS18
|        | 12        | SLOW |           |            |            |
| QY<140>                              | IOB                  | OUTPUT     | LVCMOS18
|        | 12        | SLOW |           |            |            |
| QY<141>                              | IOB                  | OUTPUT     | LVCMOS18
|        | 12        | SLOW |           |            |            |
| QY<142>                              | IOB                  | OUTPUT     | LVCMOS18
|        | 12        | SLOW |           |            |            |
| QY<143>                              | IOB                  | OUTPUT     | LVCMOS18
|        | 12        | SLOW |           |            |            |
| QY<144>                              | IOB                  | OUTPUT     | LVCMOS18
|        | 12        | SLOW |           |            |            |
| QY<145>                              | IOB                  | OUTPUT     | LVCMOS18
|        | 12        | SLOW |           |            |            |
| QY<146>                              | IOB                  | OUTPUT     | LVCMOS18
|        | 12        | SLOW |           |            |            |
| QY<147>                              | IOB                  | OUTPUT     | LVCMOS18
|        | 12        | SLOW |           |            |            |
| QY<148>                              | IOB                  | OUTPUT     | LVCMOS18
|        | 12        | SLOW |           |            |            |
| QY<149>                              | IOB                  | OUTPUT     | LVCMOS18
|        | 12        | SLOW |           |            |            |
| QY<150>                              | IOB                  | OUTPUT     | LVCMOS18
|        | 12        | SLOW |           |            |            |
| QY<151>                              | IOB                  | OUTPUT     | LVCMOS18
|        | 12        | SLOW |           |            |            |
| QY<152>                              | IOB                  | OUTPUT     | LVCMOS18
|        | 12        | SLOW |           |            |            |
| QY<153>                              | IOB                  | OUTPUT     | LVCMOS18
|        | 12        | SLOW |           |            |            |
| QY<154>                              | IOB                  | OUTPUT     | LVCMOS18
|        | 12        | SLOW |           |            |            |
| QY<155>                              | IOB                  | OUTPUT     | LVCMOS18
|        | 12        | SLOW |           |            |            |
| QY<156>                              | IOB                  | OUTPUT     | LVCMOS18
|        | 12        | SLOW |           |            |            |
| QY<157>                              | IOB                  | OUTPUT     | LVCMOS18
|        | 12        | SLOW |           |            |            |
| QY<158>                              | IOB                  | OUTPUT     | LVCMOS18
|        | 12        | SLOW |           |            |            |
| QY<159>                              | IOB                  | OUTPUT     | LVCMOS18
|        | 12        | SLOW |           |            |            |
| QY<160>                              | IOB                  | OUTPUT     | LVCMOS18
```

```
|        | 12        | SLOW |                |            |          |
| QY<161>                              | IOB            | OUTPUT     | LVCMOS18
|        | 12        | SLOW |                |            |          |
| QY<162>                              | IOB            | OUTPUT     | LVCMOS18
|        | 12        | SLOW |                |            |          |
| QY<163>                              | IOB            | OUTPUT     | LVCMOS18
|        | 12        | SLOW |                |            |          |
| QY<164>                              | IOB            | OUTPUT     | LVCMOS18
|        | 12        | SLOW |                |            |          |
| QY<165>                              | IOB            | OUTPUT     | LVCMOS18
|        | 12        | SLOW |                |            |          |
| QY<166>                              | IOB            | OUTPUT     | LVCMOS18
|        | 12        | SLOW |                |            |          |
| QY<167>                              | IOB            | OUTPUT     | LVCMOS18
|        | 12        | SLOW |                |            |          |
| QY<168>                              | IOB            | OUTPUT     | LVCMOS18
|        | 12        | SLOW |                |            |          |
| QY<169>                              | IOB            | OUTPUT     | LVCMOS18
|        | 12        | SLOW |                |            |          |
| QY<170>                              | IOB            | OUTPUT     | LVCMOS18
|        | 12        | SLOW |                |            |          |
| QY<171>                              | IOB            | OUTPUT     | LVCMOS18
|        | 12        | SLOW |                |            |          |
| QY<172>                              | IOB            | OUTPUT     | LVCMOS18
|        | 12        | SLOW |                |            |          |
| QY<173>                              | IOB            | OUTPUT     | LVCMOS18
|        | 12        | SLOW |                |            |          |
| QY<174>                              | IOB            | OUTPUT     | LVCMOS18
|        | 12        | SLOW |                |            |          |
| QY<175>                              | IOB            | OUTPUT     | LVCMOS18
|        | 12        | SLOW |                |            |          |
| QY<176>                              | IOB            | OUTPUT     | LVCMOS18
|        | 12        | SLOW |                |            |          |
| QY<177>                              | IOB            | OUTPUT     | LVCMOS18
|        | 12        | SLOW |                |            |          |
| QY<178>                              | IOB            | OUTPUT     | LVCMOS18
|        | 12        | SLOW |                |            |          |
| QY<179>                              | IOB            | OUTPUT     | LVCMOS18
|        | 12        | SLOW |                |            |          |
| QY<180>                              | IOB            | OUTPUT     | LVCMOS18
|        | 12        | SLOW |                |            |          |
| QY<181>                              | IOB            | OUTPUT     | LVCMOS18
|        | 12        | SLOW |                |            |          |
| QY<182>                              | IOB            | OUTPUT     | LVCMOS18
|        | 12        | SLOW |                |            |          |
| QY<183>                              | IOB            | OUTPUT     | LVCMOS18
|        | 12        | SLOW |                |            |          |
| QY<184>                              | IOB            | OUTPUT     | LVCMOS18
|        | 12        | SLOW |                |            |          |
| QY<185>                              | IOB            | OUTPUT     | LVCMOS18
|        | 12        | SLOW |                |            |          |
| QY<186>                              | IOB            | OUTPUT     | LVCMOS18
|        | 12        | SLOW |                |            |          |
| QY<187>                              | IOB            | OUTPUT     | LVCMOS18
|        | 12        | SLOW |                |            |          |
| QY<188>                              | IOB            | OUTPUT     | LVCMOS18
|        | 12        | SLOW |                |            |          |
| QY<189>                              | IOB            | OUTPUT     | LVCMOS18
|        | 12        | SLOW |                |            |          |
| QY<190>                              | IOB            | OUTPUT     | LVCMOS18
|        | 12        | SLOW |                |            |          |
| QY<191>                              | IOB            | OUTPUT     | LVCMOS18
|        | 12        | SLOW |                |            |          |
| QY<192>                              | IOB            | OUTPUT     | LVCMOS18
|        | 12        | SLOW |                |            |          |
| QY<193>                              | IOB            | OUTPUT     | LVCMOS18
|        | 12        | SLOW |                |            |          |
| QY<194>                              | IOB            | OUTPUT     | LVCMOS18
|        | 12        | SLOW |                |            |          |
| QY<195>                              | IOB            | OUTPUT     | LVCMOS18
|        | 12        | SLOW |                |            |          |
```

```
| QY<196>                            | IOB                  | OUTPUT     | LVCMOS18
|        | 12        | SLOW |             |            |            |
| QY<197>                            | IOB                  | OUTPUT     | LVCMOS18
|        | 12        | SLOW |             |            |            |
| QY<198>                            | IOB                  | OUTPUT     | LVCMOS18
|        | 12        | SLOW |             |            |            |
| QY<199>                            | IOB                  | OUTPUT     | LVCMOS18
|        | 12        | SLOW |             |            |            |
| QY<200>                            | IOB                  | OUTPUT     | LVCMOS18
|        | 12        | SLOW |             |            |            |
| QY<201>                            | IOB                  | OUTPUT     | LVCMOS18
|        | 12        | SLOW |             |            |            |
| QY<202>                            | IOB                  | OUTPUT     | LVCMOS18
|        | 12        | SLOW |             |            |            |
| QY<203>                            | IOB                  | OUTPUT     | LVCMOS18
|        | 12        | SLOW |             |            |            |
| QY<204>                            | IOB                  | OUTPUT     | LVCMOS18
|        | 12        | SLOW |             |            |            |
| QY<205>                            | IOB                  | OUTPUT     | LVCMOS18
|        | 12        | SLOW |             |            |            |
| QY<206>                            | IOB                  | OUTPUT     | LVCMOS18
|        | 12        | SLOW |             |            |            |
| QY<207>                            | IOB                  | OUTPUT     | LVCMOS18
|        | 12        | SLOW |             |            |            |
| QY<208>                            | IOB                  | OUTPUT     | LVCMOS18
|        | 12        | SLOW |             |            |            |
| QY<209>                            | IOB                  | OUTPUT     | LVCMOS18
|        | 12        | SLOW |             |            |            |
| QY<210>                            | IOB                  | OUTPUT     | LVCMOS18
|        | 12        | SLOW |             |            |            |
| QY<211>                            | IOB                  | OUTPUT     | LVCMOS18
|        | 12        | SLOW |             |            |            |
| QY<212>                            | IOB                  | OUTPUT     | LVCMOS18
|        | 12        | SLOW |             |            |            |
| QY<213>                            | IOB                  | OUTPUT     | LVCMOS18
|        | 12        | SLOW |             |            |            |
| QY<214>                            | IOB                  | OUTPUT     | LVCMOS18
|        | 12        | SLOW |             |            |            |
| QY<215>                            | IOB                  | OUTPUT     | LVCMOS18
|        | 12        | SLOW |             |            |            |
| QY<216>                            | IOB                  | OUTPUT     | LVCMOS18
|        | 12        | SLOW |             |            |            |
| QY<217>                            | IOB                  | OUTPUT     | LVCMOS18
|        | 12        | SLOW |             |            |            |
| QY<218>                            | IOB                  | OUTPUT     | LVCMOS18
|        | 12        | SLOW |             |            |            |
| QY<219>                            | IOB                  | OUTPUT     | LVCMOS18
|        | 12        | SLOW |             |            |            |
| QY<220>                            | IOB                  | OUTPUT     | LVCMOS18
|        | 12        | SLOW |             |            |            |
| QY<221>                            | IOB                  | OUTPUT     | LVCMOS18
|        | 12        | SLOW |             |            |            |
| QY<222>                            | IOB                  | OUTPUT     | LVCMOS18
|        | 12        | SLOW |             |            |            |
| QY<223>                            | IOB                  | OUTPUT     | LVCMOS18
|        | 12        | SLOW |             |            |            |
| QY<224>                            | IOB                  | OUTPUT     | LVCMOS18
|        | 12        | SLOW |             |            |            |
| QY<225>                            | IOB                  | OUTPUT     | LVCMOS18
|        | 12        | SLOW |             |            |            |
| QY<226>                            | IOB                  | OUTPUT     | LVCMOS18
|        | 12        | SLOW |             |            |            |
| QY<227>                            | IOB                  | OUTPUT     | LVCMOS18
|        | 12        | SLOW |             |            |            |
| QY<228>                            | IOB                  | OUTPUT     | LVCMOS18
|        | 12        | SLOW |             |            |            |
| QY<229>                            | IOB                  | OUTPUT     | LVCMOS18
|        | 12        | SLOW |             |            |            |
| QY<230>                            | IOB                  | OUTPUT     | LVCMOS18
|        | 12        | SLOW |             |            |            |
| QY<231>                            | IOB                  | OUTPUT     | LVCMOS18
```

```
|        | 12        | SLOW |              |           |          |
| QY<232>                            | IOB              | OUTPUT     | LVCMOS18
|        | 12        | SLOW |              |           |          |
| QZ<0>                              | IOB              | OUTPUT     | LVCMOS18
|        | 12        | SLOW |              |           |          |
| QZ<1>                              | IOB              | OUTPUT     | LVCMOS18
|        | 12        | SLOW |              |           |          |
| QZ<2>                              | IOB              | OUTPUT     | LVCMOS18
|        | 12        | SLOW |              |           |          |
| QZ<3>                              | IOB              | OUTPUT     | LVCMOS18
|        | 12        | SLOW |              |           |          |
| QZ<4>                              | IOB              | OUTPUT     | LVCMOS18
|        | 12        | SLOW |              |           |          |
| QZ<5>                              | IOB              | OUTPUT     | LVCMOS18
|        | 12        | SLOW |              |           |          |
| QZ<6>                              | IOB              | OUTPUT     | LVCMOS18
|        | 12        | SLOW |              |           |          |
| QZ<7>                              | IOB              | OUTPUT     | LVCMOS18
|        | 12        | SLOW |              |           |          |
| QZ<8>                              | IOB              | OUTPUT     | LVCMOS18
|        | 12        | SLOW |              |           |          |
| QZ<9>                              | IOB              | OUTPUT     | LVCMOS18
|        | 12        | SLOW |              |           |          |
| QZ<10>                             | IOB              | OUTPUT     | LVCMOS18
|        | 12        | SLOW |              |           |          |
| QZ<11>                             | IOB              | OUTPUT     | LVCMOS18
|        | 12        | SLOW |              |           |          |
| QZ<12>                             | IOB              | OUTPUT     | LVCMOS18
|        | 12        | SLOW |              |           |          |
| QZ<13>                             | IOB              | OUTPUT     | LVCMOS18
|        | 12        | SLOW |              |           |          |
| QZ<14>                             | IOB              | OUTPUT     | LVCMOS18
|        | 12        | SLOW |              |           |          |
| QZ<15>                             | IOB              | OUTPUT     | LVCMOS18
|        | 12        | SLOW |              |           |          |
| QZ<16>                             | IOB              | OUTPUT     | LVCMOS18
|        | 12        | SLOW |              |           |          |
| QZ<17>                             | IOB              | OUTPUT     | LVCMOS18
|        | 12        | SLOW |              |           |          |
| QZ<18>                             | IOB              | OUTPUT     | LVCMOS18
|        | 12        | SLOW |              |           |          |
| QZ<19>                             | IOB              | OUTPUT     | LVCMOS18
|        | 12        | SLOW |              |           |          |
| QZ<20>                             | IOB              | OUTPUT     | LVCMOS18
|        | 12        | SLOW |              |           |          |
| QZ<21>                             | IOB              | OUTPUT     | LVCMOS18
|        | 12        | SLOW |              |           |          |
| QZ<22>                             | IOB              | OUTPUT     | LVCMOS18
|        | 12        | SLOW |              |           |          |
| QZ<23>                             | IOB              | OUTPUT     | LVCMOS18
|        | 12        | SLOW |              |           |          |
| QZ<24>                             | IOB              | OUTPUT     | LVCMOS18
|        | 12        | SLOW |              |           |          |
| QZ<25>                             | IOB              | OUTPUT     | LVCMOS18
|        | 12        | SLOW |              |           |          |
| QZ<26>                             | IOB              | OUTPUT     | LVCMOS18
|        | 12        | SLOW |              |           |          |
| QZ<27>                             | IOB              | OUTPUT     | LVCMOS18
|        | 12        | SLOW |              |           |          |
| QZ<28>                             | IOB              | OUTPUT     | LVCMOS18
|        | 12        | SLOW |              |           |          |
| QZ<29>                             | IOB              | OUTPUT     | LVCMOS18
|        | 12        | SLOW |              |           |          |
| QZ<30>                             | IOB              | OUTPUT     | LVCMOS18
|        | 12        | SLOW |              |           |          |
| QZ<31>                             | IOB              | OUTPUT     | LVCMOS18
|        | 12        | SLOW |              |           |          |
| QZ<32>                             | IOB              | OUTPUT     | LVCMOS18
|        | 12        | SLOW |              |           |          |
| QZ<33>                             | IOB              | OUTPUT     | LVCMOS18
|        | 12        | SLOW |              |           |          |
```

```
| QZ<34>                                | IOB                 | OUTPUT     | LVCMOS18
|       | 12        | SLOW |                |           |          |
| QZ<35>                                | IOB                 | OUTPUT     | LVCMOS18
|       | 12        | SLOW |                |           |          |
| QZ<36>                                | IOB                 | OUTPUT     | LVCMOS18
|       | 12        | SLOW |                |           |          |
| QZ<37>                                | IOB                 | OUTPUT     | LVCMOS18
|       | 12        | SLOW |                |           |          |
| QZ<38>                                | IOB                 | OUTPUT     | LVCMOS18
|       | 12        | SLOW |                |           |          |
| QZ<39>                                | IOB                 | OUTPUT     | LVCMOS18
|       | 12        | SLOW |                |           |          |
| QZ<40>                                | IOB                 | OUTPUT     | LVCMOS18
|       | 12        | SLOW |                |           |          |
| QZ<41>                                | IOB                 | OUTPUT     | LVCMOS18
|       | 12        | SLOW |                |           |          |
| QZ<42>                                | IOB                 | OUTPUT     | LVCMOS18
|       | 12        | SLOW |                |           |          |
| QZ<43>                                | IOB                 | OUTPUT     | LVCMOS18
|       | 12        | SLOW |                |           |          |
| QZ<44>                                | IOB                 | OUTPUT     | LVCMOS18
|       | 12        | SLOW |                |           |          |
| QZ<45>                                | IOB                 | OUTPUT     | LVCMOS18
|       | 12        | SLOW |                |           |          |
| QZ<46>                                | IOB                 | OUTPUT     | LVCMOS18
|       | 12        | SLOW |                |           |          |
| QZ<47>                                | IOB                 | OUTPUT     | LVCMOS18
|       | 12        | SLOW |                |           |          |
| QZ<48>                                | IOB                 | OUTPUT     | LVCMOS18
|       | 12        | SLOW |                |           |          |
| QZ<49>                                | IOB                 | OUTPUT     | LVCMOS18
|       | 12        | SLOW |                |           |          |
| QZ<50>                                | IOB                 | OUTPUT     | LVCMOS18
|       | 12        | SLOW |                |           |          |
| QZ<51>                                | IOB                 | OUTPUT     | LVCMOS18
|       | 12        | SLOW |                |           |          |
| QZ<52>                                | IOB                 | OUTPUT     | LVCMOS18
|       | 12        | SLOW |                |           |          |
| QZ<53>                                | IOB                 | OUTPUT     | LVCMOS18
|       | 12        | SLOW |                |           |          |
| QZ<54>                                | IOB                 | OUTPUT     | LVCMOS18
|       | 12        | SLOW |                |           |          |
| QZ<55>                                | IOB                 | OUTPUT     | LVCMOS18
|       | 12        | SLOW |                |           |          |
| QZ<56>                                | IOB                 | OUTPUT     | LVCMOS18
|       | 12        | SLOW |                |           |          |
| QZ<57>                                | IOB                 | OUTPUT     | LVCMOS18
|       | 12        | SLOW |                |           |          |
| QZ<58>                                | IOB                 | OUTPUT     | LVCMOS18
|       | 12        | SLOW |                |           |          |
| QZ<59>                                | IOB                 | OUTPUT     | LVCMOS18
|       | 12        | SLOW |                |           |          |
| QZ<60>                                | IOB                 | OUTPUT     | LVCMOS18
|       | 12        | SLOW |                |           |          |
| QZ<61>                                | IOB                 | OUTPUT     | LVCMOS18
|       | 12        | SLOW |                |           |          |
| QZ<62>                                | IOB                 | OUTPUT     | LVCMOS18
|       | 12        | SLOW |                |           |          |
| QZ<63>                                | IOB                 | OUTPUT     | LVCMOS18
|       | 12        | SLOW |                |           |          |
| QZ<64>                                | IOB                 | OUTPUT     | LVCMOS18
|       | 12        | SLOW |                |           |          |
| QZ<65>                                | IOB                 | OUTPUT     | LVCMOS18
|       | 12        | SLOW |                |           |          |
| QZ<66>                                | IOB                 | OUTPUT     | LVCMOS18
|       | 12        | SLOW |                |           |          |
| QZ<67>                                | IOB                 | OUTPUT     | LVCMOS18
|       | 12        | SLOW |                |           |          |
| QZ<68>                                | IOB                 | OUTPUT     | LVCMOS18
|       | 12        | SLOW |                |           |          |
| QZ<69>                                | IOB                 | OUTPUT     | LVCMOS18
```

```
|       | 12        | SLOW |                   |         |          |
| QZ<70>                           | IOB               | OUTPUT     | LVCMOS18
|       | 12        | SLOW |                   |         |          |
| QZ<71>                           | IOB               | OUTPUT     | LVCMOS18
|       | 12        | SLOW |                   |         |          |
| QZ<72>                           | IOB               | OUTPUT     | LVCMOS18
|       | 12        | SLOW |                   |         |          |
| QZ<73>                           | IOB               | OUTPUT     | LVCMOS18
|       | 12        | SLOW |                   |         |          |
| QZ<74>                           | IOB               | OUTPUT     | LVCMOS18
|       | 12        | SLOW |                   |         |          |
| QZ<75>                           | IOB               | OUTPUT     | LVCMOS18
|       | 12        | SLOW |                   |         |          |
| QZ<76>                           | IOB               | OUTPUT     | LVCMOS18
|       | 12        | SLOW |                   |         |          |
| QZ<77>                           | IOB               | OUTPUT     | LVCMOS18
|       | 12        | SLOW |                   |         |          |
| QZ<78>                           | IOB               | OUTPUT     | LVCMOS18
|       | 12        | SLOW |                   |         |          |
| QZ<79>                           | IOB               | OUTPUT     | LVCMOS18
|       | 12        | SLOW |                   |         |          |
| QZ<80>                           | IOB               | OUTPUT     | LVCMOS18
|       | 12        | SLOW |                   |         |          |
| QZ<81>                           | IOB               | OUTPUT     | LVCMOS18
|       | 12        | SLOW |                   |         |          |
| QZ<82>                           | IOB               | OUTPUT     | LVCMOS18
|       | 12        | SLOW |                   |         |          |
| QZ<83>                           | IOB               | OUTPUT     | LVCMOS18
|       | 12        | SLOW |                   |         |          |
| QZ<84>                           | IOB               | OUTPUT     | LVCMOS18
|       | 12        | SLOW |                   |         |          |
| QZ<85>                           | IOB               | OUTPUT     | LVCMOS18
|       | 12        | SLOW |                   |         |          |
| QZ<86>                           | IOB               | OUTPUT     | LVCMOS18
|       | 12        | SLOW |                   |         |          |
| QZ<87>                           | IOB               | OUTPUT     | LVCMOS18
|       | 12        | SLOW |                   |         |          |
| QZ<88>                           | IOB               | OUTPUT     | LVCMOS18
|       | 12        | SLOW |                   |         |          |
| QZ<89>                           | IOB               | OUTPUT     | LVCMOS18
|       | 12        | SLOW |                   |         |          |
| QZ<90>                           | IOB               | OUTPUT     | LVCMOS18
|       | 12        | SLOW |                   |         |          |
| QZ<91>                           | IOB               | OUTPUT     | LVCMOS18
|       | 12        | SLOW |                   |         |          |
| QZ<92>                           | IOB               | OUTPUT     | LVCMOS18
|       | 12        | SLOW |                   |         |          |
| QZ<93>                           | IOB               | OUTPUT     | LVCMOS18
|       | 12        | SLOW |                   |         |          |
| QZ<94>                           | IOB               | OUTPUT     | LVCMOS18
|       | 12        | SLOW |                   |         |          |
| QZ<95>                           | IOB               | OUTPUT     | LVCMOS18
|       | 12        | SLOW |                   |         |          |
| QZ<96>                           | IOB               | OUTPUT     | LVCMOS18
|       | 12        | SLOW |                   |         |          |
| QZ<97>                           | IOB               | OUTPUT     | LVCMOS18
|       | 12        | SLOW |                   |         |          |
| QZ<98>                           | IOB               | OUTPUT     | LVCMOS18
|       | 12        | SLOW |                   |         |          |
| QZ<99>                           | IOB               | OUTPUT     | LVCMOS18
|       | 12        | SLOW |                   |         |          |
| QZ<100>                          | IOB               | OUTPUT     | LVCMOS18
|       | 12        | SLOW |                   |         |          |
| QZ<101>                          | IOB               | OUTPUT     | LVCMOS18
|       | 12        | SLOW |                   |         |          |
| QZ<102>                          | IOB               | OUTPUT     | LVCMOS18
|       | 12        | SLOW |                   |         |          |
| QZ<103>                          | IOB               | OUTPUT     | LVCMOS18
|       | 12        | SLOW |                   |         |          |
| QZ<104>                          | IOB               | OUTPUT     | LVCMOS18
|       | 12        | SLOW |                   |         |          |
```

```
| QZ<105>                                  | IOB                 | OUTPUT     | LVCMOS18
|        | 12       | SLOW |                  |              |            |
| QZ<106>                                  | IOB                 | OUTPUT     | LVCMOS18
|        | 12       | SLOW |                  |              |            |
| QZ<107>                                  | IOB                 | OUTPUT     | LVCMOS18
|        | 12       | SLOW |                  |              |            |
| QZ<108>                                  | IOB                 | OUTPUT     | LVCMOS18
|        | 12       | SLOW |                  |              |            |
| QZ<109>                                  | IOB                 | OUTPUT     | LVCMOS18
|        | 12       | SLOW |                  |              |            |
| QZ<110>                                  | IOB                 | OUTPUT     | LVCMOS18
|        | 12       | SLOW |                  |              |            |
| QZ<111>                                  | IOB                 | OUTPUT     | LVCMOS18
|        | 12       | SLOW |                  |              |            |
| QZ<112>                                  | IOB                 | OUTPUT     | LVCMOS18
|        | 12       | SLOW |                  |              |            |
| QZ<113>                                  | IOB                 | OUTPUT     | LVCMOS18
|        | 12       | SLOW |                  |              |            |
| QZ<114>                                  | IOB                 | OUTPUT     | LVCMOS18
|        | 12       | SLOW |                  |              |            |
| QZ<115>                                  | IOB                 | OUTPUT     | LVCMOS18
|        | 12       | SLOW |                  |              |            |
| QZ<116>                                  | IOB                 | OUTPUT     | LVCMOS18
|        | 12       | SLOW |                  |              |            |
| QZ<117>                                  | IOB                 | OUTPUT     | LVCMOS18
|        | 12       | SLOW |                  |              |            |
| QZ<118>                                  | IOB                 | OUTPUT     | LVCMOS18
|        | 12       | SLOW |                  |              |            |
| QZ<119>                                  | IOB                 | OUTPUT     | LVCMOS18
|        | 12       | SLOW |                  |              |            |
| QZ<120>                                  | IOB                 | OUTPUT     | LVCMOS18
|        | 12       | SLOW |                  |              |            |
| QZ<121>                                  | IOB                 | OUTPUT     | LVCMOS18
|        | 12       | SLOW |                  |              |            |
| QZ<122>                                  | IOB                 | OUTPUT     | LVCMOS18
|        | 12       | SLOW |                  |              |            |
| QZ<123>                                  | IOB                 | OUTPUT     | LVCMOS18
|        | 12       | SLOW |                  |              |            |
| QZ<124>                                  | IOB                 | OUTPUT     | LVCMOS18
|        | 12       | SLOW |                  |              |            |
| QZ<125>                                  | IOB                 | OUTPUT     | LVCMOS18
|        | 12       | SLOW |                  |              |            |
| QZ<126>                                  | IOB                 | OUTPUT     | LVCMOS18
|        | 12       | SLOW |                  |              |            |
| QZ<127>                                  | IOB                 | OUTPUT     | LVCMOS18
|        | 12       | SLOW |                  |              |            |
| QZ<128>                                  | IOB                 | OUTPUT     | LVCMOS18
|        | 12       | SLOW |                  |              |            |
| QZ<129>                                  | IOB                 | OUTPUT     | LVCMOS18
|        | 12       | SLOW |                  |              |            |
| QZ<130>                                  | IOB                 | OUTPUT     | LVCMOS18
|        | 12       | SLOW |                  |              |            |
| QZ<131>                                  | IOB                 | OUTPUT     | LVCMOS18
|        | 12       | SLOW |                  |              |            |
| QZ<132>                                  | IOB                 | OUTPUT     | LVCMOS18
|        | 12       | SLOW |                  |              |            |
| QZ<133>                                  | IOB                 | OUTPUT     | LVCMOS18
|        | 12       | SLOW |                  |              |            |
| QZ<134>                                  | IOB                 | OUTPUT     | LVCMOS18
|        | 12       | SLOW |                  |              |            |
| QZ<135>                                  | IOB                 | OUTPUT     | LVCMOS18
|        | 12       | SLOW |                  |              |            |
| QZ<136>                                  | IOB                 | OUTPUT     | LVCMOS18
|        | 12       | SLOW |                  |              |            |
| QZ<137>                                  | IOB                 | OUTPUT     | LVCMOS18
|        | 12       | SLOW |                  |              |            |
| QZ<138>                                  | IOB                 | OUTPUT     | LVCMOS18
|        | 12       | SLOW |                  |              |            |
| QZ<139>                                  | IOB                 | OUTPUT     | LVCMOS18
|        | 12       | SLOW |                  |              |            |
| QZ<140>                                  | IOB                 | OUTPUT     | LVCMOS18
```

```
|        | 12         | SLOW |                 |           |          |
| QZ<141>                              | IOB                | OUTPUT     | LVCMOS18
|        | 12         | SLOW |                 |           |          |
| QZ<142>                              | IOB                | OUTPUT     | LVCMOS18
|        | 12         | SLOW |                 |           |          |
| QZ<143>                              | IOB                | OUTPUT     | LVCMOS18
|        | 12         | SLOW |                 |           |          |
| QZ<144>                              | IOB                | OUTPUT     | LVCMOS18
|        | 12         | SLOW |                 |           |          |
| QZ<145>                              | IOB                | OUTPUT     | LVCMOS18
|        | 12         | SLOW |                 |           |          |
| QZ<146>                              | IOB                | OUTPUT     | LVCMOS18
|        | 12         | SLOW |                 |           |          |
| QZ<147>                              | IOB                | OUTPUT     | LVCMOS18
|        | 12         | SLOW |                 |           |          |
| QZ<148>                              | IOB                | OUTPUT     | LVCMOS18
|        | 12         | SLOW |                 |           |          |
| QZ<149>                              | IOB                | OUTPUT     | LVCMOS18
|        | 12         | SLOW |                 |           |          |
| QZ<150>                              | IOB                | OUTPUT     | LVCMOS18
|        | 12         | SLOW |                 |           |          |
| QZ<151>                              | IOB                | OUTPUT     | LVCMOS18
|        | 12         | SLOW |                 |           |          |
| QZ<152>                              | IOB                | OUTPUT     | LVCMOS18
|        | 12         | SLOW |                 |           |          |
| QZ<153>                              | IOB                | OUTPUT     | LVCMOS18
|        | 12         | SLOW |                 |           |          |
| QZ<154>                              | IOB                | OUTPUT     | LVCMOS18
|        | 12         | SLOW |                 |           |          |
| QZ<155>                              | IOB                | OUTPUT     | LVCMOS18
|        | 12         | SLOW |                 |           |          |
| QZ<156>                              | IOB                | OUTPUT     | LVCMOS18
|        | 12         | SLOW |                 |           |          |
| QZ<157>                              | IOB                | OUTPUT     | LVCMOS18
|        | 12         | SLOW |                 |           |          |
| QZ<158>                              | IOB                | OUTPUT     | LVCMOS18
|        | 12         | SLOW |                 |           |          |
| QZ<159>                              | IOB                | OUTPUT     | LVCMOS18
|        | 12         | SLOW |                 |           |          |
| QZ<160>                              | IOB                | OUTPUT     | LVCMOS18
|        | 12         | SLOW |                 |           |          |
| QZ<161>                              | IOB                | OUTPUT     | LVCMOS18
|        | 12         | SLOW |                 |           |          |
| QZ<162>                              | IOB                | OUTPUT     | LVCMOS18
|        | 12         | SLOW |                 |           |          |
| QZ<163>                              | IOB                | OUTPUT     | LVCMOS18
|        | 12         | SLOW |                 |           |          |
| QZ<164>                              | IOB                | OUTPUT     | LVCMOS18
|        | 12         | SLOW |                 |           |          |
| QZ<165>                              | IOB                | OUTPUT     | LVCMOS18
|        | 12         | SLOW |                 |           |          |
| QZ<166>                              | IOB                | OUTPUT     | LVCMOS18
|        | 12         | SLOW |                 |           |          |
| QZ<167>                              | IOB                | OUTPUT     | LVCMOS18
|        | 12         | SLOW |                 |           |          |
| QZ<168>                              | IOB                | OUTPUT     | LVCMOS18
|        | 12         | SLOW |                 |           |          |
| QZ<169>                              | IOB                | OUTPUT     | LVCMOS18
|        | 12         | SLOW |                 |           |          |
| QZ<170>                              | IOB                | OUTPUT     | LVCMOS18
|        | 12         | SLOW |                 |           |          |
| QZ<171>                              | IOB                | OUTPUT     | LVCMOS18
|        | 12         | SLOW |                 |           |          |
| QZ<172>                              | IOB                | OUTPUT     | LVCMOS18
|        | 12         | SLOW |                 |           |          |
| QZ<173>                              | IOB                | OUTPUT     | LVCMOS18
|        | 12         | SLOW |                 |           |          |
| QZ<174>                              | IOB                | OUTPUT     | LVCMOS18
|        | 12         | SLOW |                 |           |          |
| QZ<175>                              | IOB                | OUTPUT     | LVCMOS18
|        | 12         | SLOW |                 |           |          |
```

```
| QZ<176>                                | IOB                  | OUTPUT     | LVCMOS18
|        | 12         | SLOW |              |            |            |
| QZ<177>                                | IOB                  | OUTPUT     | LVCMOS18
|        | 12         | SLOW |              |            |            |
| QZ<178>                                | IOB                  | OUTPUT     | LVCMOS18
|        | 12         | SLOW |              |            |            |
| QZ<179>                                | IOB                  | OUTPUT     | LVCMOS18
|        | 12         | SLOW |              |            |            |
| QZ<180>                                | IOB                  | OUTPUT     | LVCMOS18
|        | 12         | SLOW |              |            |            |
| QZ<181>                                | IOB                  | OUTPUT     | LVCMOS18
|        | 12         | SLOW |              |            |            |
| QZ<182>                                | IOB                  | OUTPUT     | LVCMOS18
|        | 12         | SLOW |              |            |            |
| QZ<183>                                | IOB                  | OUTPUT     | LVCMOS18
|        | 12         | SLOW |              |            |            |
| QZ<184>                                | IOB                  | OUTPUT     | LVCMOS18
|        | 12         | SLOW |              |            |            |
| QZ<185>                                | IOB                  | OUTPUT     | LVCMOS18
|        | 12         | SLOW |              |            |            |
| QZ<186>                                | IOB                  | OUTPUT     | LVCMOS18
|        | 12         | SLOW |              |            |            |
| QZ<187>                                | IOB                  | OUTPUT     | LVCMOS18
|        | 12         | SLOW |              |            |            |
| QZ<188>                                | IOB                  | OUTPUT     | LVCMOS18
|        | 12         | SLOW |              |            |            |
| QZ<189>                                | IOB                  | OUTPUT     | LVCMOS18
|        | 12         | SLOW |              |            |            |
| QZ<190>                                | IOB                  | OUTPUT     | LVCMOS18
|        | 12         | SLOW |              |            |            |
| QZ<191>                                | IOB                  | OUTPUT     | LVCMOS18
|        | 12         | SLOW |              |            |            |
| QZ<192>                                | IOB                  | OUTPUT     | LVCMOS18
|        | 12         | SLOW |              |            |            |
| QZ<193>                                | IOB                  | OUTPUT     | LVCMOS18
|        | 12         | SLOW |              |            |            |
| QZ<194>                                | IOB                  | OUTPUT     | LVCMOS18
|        | 12         | SLOW |              |            |            |
| QZ<195>                                | IOB                  | OUTPUT     | LVCMOS18
|        | 12         | SLOW |              |            |            |
| QZ<196>                                | IOB                  | OUTPUT     | LVCMOS18
|        | 12         | SLOW |              |            |            |
| QZ<197>                                | IOB                  | OUTPUT     | LVCMOS18
|        | 12         | SLOW |              |            |            |
| QZ<198>                                | IOB                  | OUTPUT     | LVCMOS18
|        | 12         | SLOW |              |            |            |
| QZ<199>                                | IOB                  | OUTPUT     | LVCMOS18
|        | 12         | SLOW |              |            |            |
| QZ<200>                                | IOB                  | OUTPUT     | LVCMOS18
|        | 12         | SLOW |              |            |            |
| QZ<201>                                | IOB                  | OUTPUT     | LVCMOS18
|        | 12         | SLOW |              |            |            |
| QZ<202>                                | IOB                  | OUTPUT     | LVCMOS18
|        | 12         | SLOW |              |            |            |
| QZ<203>                                | IOB                  | OUTPUT     | LVCMOS18
|        | 12         | SLOW |              |            |            |
| QZ<204>                                | IOB                  | OUTPUT     | LVCMOS18
|        | 12         | SLOW |              |            |            |
| QZ<205>                                | IOB                  | OUTPUT     | LVCMOS18
|        | 12         | SLOW |              |            |            |
| QZ<206>                                | IOB                  | OUTPUT     | LVCMOS18
|        | 12         | SLOW |              |            |            |
| QZ<207>                                | IOB                  | OUTPUT     | LVCMOS18
|        | 12         | SLOW |              |            |            |
| QZ<208>                                | IOB                  | OUTPUT     | LVCMOS18
|        | 12         | SLOW |              |            |            |
| QZ<209>                                | IOB                  | OUTPUT     | LVCMOS18
|        | 12         | SLOW |              |            |            |
| QZ<210>                                | IOB                  | OUTPUT     | LVCMOS18
|        | 12         | SLOW |              |            |            |
| QZ<211>                                | IOB                  | OUTPUT     | LVCMOS18
```

```
|       | 12        | SLOW |              |           |
| QZ<212>                              | IOB          | OUTPUT    | LVCMOS18
|       | 12        | SLOW |              |           |
| QZ<213>                              | IOB          | OUTPUT    | LVCMOS18
|       | 12        | SLOW |              |           |
| QZ<214>                              | IOB          | OUTPUT    | LVCMOS18
|       | 12        | SLOW |              |           |
| QZ<215>                              | IOB          | OUTPUT    | LVCMOS18
|       | 12        | SLOW |              |           |
| QZ<216>                              | IOB          | OUTPUT    | LVCMOS18
|       | 12        | SLOW |              |           |
| QZ<217>                              | IOB          | OUTPUT    | LVCMOS18
|       | 12        | SLOW |              |           |
| QZ<218>                              | IOB          | OUTPUT    | LVCMOS18
|       | 12        | SLOW |              |           |
| QZ<219>                              | IOB          | OUTPUT    | LVCMOS18
|       | 12        | SLOW |              |           |
| QZ<220>                              | IOB          | OUTPUT    | LVCMOS18
|       | 12        | SLOW |              |           |
| QZ<221>                              | IOB          | OUTPUT    | LVCMOS18
|       | 12        | SLOW |              |           |
| QZ<222>                              | IOB          | OUTPUT    | LVCMOS18
|       | 12        | SLOW |              |           |
| QZ<223>                              | IOB          | OUTPUT    | LVCMOS18
|       | 12        | SLOW |              |           |
| QZ<224>                              | IOB          | OUTPUT    | LVCMOS18
|       | 12        | SLOW |              |           |
| QZ<225>                              | IOB          | OUTPUT    | LVCMOS18
|       | 12        | SLOW |              |           |
| QZ<226>                              | IOB          | OUTPUT    | LVCMOS18
|       | 12        | SLOW |              |           |
| QZ<227>                              | IOB          | OUTPUT    | LVCMOS18
|       | 12        | SLOW |              |           |
| QZ<228>                              | IOB          | OUTPUT    | LVCMOS18
|       | 12        | SLOW |              |           |
| QZ<229>                              | IOB          | OUTPUT    | LVCMOS18
|       | 12        | SLOW |              |           |
| QZ<230>                              | IOB          | OUTPUT    | LVCMOS18
|       | 12        | SLOW |              |           |
| QZ<231>                              | IOB          | OUTPUT    | LVCMOS18
|       | 12        | SLOW |              |           |
| QZ<232>                              | IOB          | OUTPUT    | LVCMOS18
|       | 12        | SLOW |              |           |
| clk                                  | IOB          | INPUT     | LVCMOS18
|       |           |      |              |           |
| done                                 | IOB          | OUTPUT    | LVCMOS18
|       | 12        | SLOW |              |           |
| reset                                | IOB          | INPUT     | LVCMOS18
|       |           |      |              |           |
+----------------------------------------------------------------------------------------------------
----------------------------------------------------------------+

Section 7 - RPMs
----------------

Section 8 - Guide Report
------------------------
Guide not run on this design.

Section 9 - Area Group and Partition Summary
--------------------------------------------

Partition Implementation Status
-------------------------------

  No Partitions were found in this design.

-------------------------------

Area Group Information
----------------------
```

```
  No area groups were found in this design.

----------------------

Section 10 - Timing Report
--------------------------
A logic-level (pre-route) timing report can be generated by using Xilinx static
timing analysis tools, Timing Analyzer (GUI) or TRCE (command line), with the
mapped NCD and PCF files. Please note that this timing report will be generated
using estimated delay information. For accurate numbers, please generate a
timing report with the post Place and Route NCD file.

For more information about the Timing Analyzer, consult the Xilinx Timing
Analyzer Reference Manual; for more information about TRCE, consult the Xilinx
Command Line Tools User Guide "TRACE" chapter.

Section 11 - Configuration String Details
-----------------------------------------
Use the "-detail" map option to print out Configuration Strings

Section 12 - Control Set Information
------------------------------------
Use the "-detail" map option to print out Control Set Information.

Section 13 - Utilization by Hierarchy
-------------------------------------
Use the "-detail" map option to print out the Utilization by Hierarchy section.
```
