# Supplementary material for: Parallel point-multiplication architecture using combined group operations for high-speed cryptographic applications
Source: PLoS One. 2017 May 1;12(5):e0176214. doi: 10.1371/journal.pone.0176214 (PMC5411040; doi:10.1371/journal.pone.0176214)
Supplement: S1 Supporting Information — (ZIP) [file pone.0176214.s001.zip › S1 Supporting Information/S1 File20 Table3_[h].pdf]

```
Information: Propagating switching activity (low effort zero
delay simulation). (PWR-6)
Warning: Design has unannotated primary inputs. (PWR-414)
Warning: Design has unannotated sequential cell outputs. (PWR-
415)

****************************************
Report : power
        -analysis_effort low
Design : ECC_TOP_K_233
Version: F-2011.09-SP3
Date   : Wed Oct 12 22:01:06 2016
****************************************


Library(s) Used:

    CORE65LPLVT (File: /usr/local-
eit/cad2/cmpstm/stm065v536/CORE65LPLVT_
5.1/libs/CORE65LPLVT_nom_1.20V_25C.db)


Operating Conditions: nom_1.20V_25C   Library: CORE65LPLVT
Wire Load Model Mode: enclosed

Design        Wire Load Model            Library
------------------------------------------------
ECC_TOP_K_233          area_780Kto1170K  CORE65LPLVT
PD_PA_BF               area_780Kto1170K  CORE65LPLVT
select_logic           area_1Kto2K       CORE65LPLVT
MUX_1_new              area_7Kto8K       CORE65LPLVT
MUX_2_new              area_4Kto5K       CORE65LPLVT
Reg_MUX_3              area_7Kto8K       CORE65LPLVT
pol_SQ_0               area_156Kto234K   CORE65LPLVT
pol_mult_0             area_312Kto390K   CORE65LPLVT
pol_add_0              area_0Kto1K       CORE65LPLVT
pol_SQ_1               area_312Kto390K   CORE65LPLVT
pol_SQ_2               area_312Kto390K   CORE65LPLVT
pol_SQ_3               area_312Kto390K   CORE65LPLVT
pol_SQ_4               area_390Kto780K   CORE65LPLVT
pol_SQ_5               area_5Kto6K       CORE65LPLVT
pol_SQ_6               area_390Kto780K   CORE65LPLVT
pol_SQ_7               area_5Kto6K       CORE65LPLVT
pol_mult_1             area_312Kto390K   CORE65LPLVT
pol_mult_2             area_312Kto390K   CORE65LPLVT
pol_mult_3             area_312Kto390K   CORE65LPLVT
pol_mult_4             area_312Kto390K   CORE65LPLVT
pol_mult_5             area_312Kto390K   CORE65LPLVT
pol_mult_6             area_312Kto390K   CORE65LPLVT
pol_mult_7             area_312Kto390K   CORE65LPLVT
pol_mult_8             area_312Kto390K   CORE65LPLVT
pol_mult_9             area_390Kto780K   CORE65LPLVT
pol_mult_10            area_312Kto390K   CORE65LPLVT
pol_mult_11            area_312Kto390K   CORE65LPLVT
pol_mult_12            area_312Kto390K   CORE65LPLVT
pol_mult_13            area_312Kto390K   CORE65LPLVT
pol_mult_14            area_312Kto390K   CORE65LPLVT
```

```
pol_mult_15           area_312Kto390K   CORE65LPLVT
pol_add_1             area_0Kto1K       CORE65LPLVT
pol_add_2             area_0Kto1K       CORE65LPLVT
pol_add_3             area_0Kto1K       CORE65LPLVT
pol_add_4             area_0Kto1K       CORE65LPLVT
pol_add_5             area_0Kto1K       CORE65LPLVT
pol_add_6             area_0Kto1K       CORE65LPLVT
pol_add_7             area_1Kto2K       CORE65LPLVT
pol_add_8             area_0Kto1K       CORE65LPLVT
pol_add_9             area_1Kto2K       CORE65LPLVT
pol_add_10            area_1Kto2K       CORE65LPLVT


Global Operating Voltage = 1.2
Power-specific unit information :
    Voltage Units = 1V
    Capacitance Units = 1.000000pf
    Time Units = 1ns
    Dynamic Power Units = 1mW    (derived from V,C,T units)
    Leakage Power Units = 1mW


  Cell Internal Power  = 412.5363 mW   (34%)
  Net Switching Power  = 800.3209 mW   (66%)
                         ---------
Total Dynamic Power    =   1.2129  W  (100%)

Cell Leakage Power     =   4.1437 mW


                 Internal         Switching          Leakage
Total
Power Group      Power            Power              Power
Power   (   %    )  Attrs
--------------------------------------------------------------
------------------------------------
io_pad             0.0000           0.0000            0.0000
0.0000  (   0.00%)
memory             0.0000           0.0000            0.0000
0.0000  (   0.00%)
black_box          0.0000           0.0000            0.0000
0.0000  (   0.00%)
clock_network      0.0000           0.0000            0.0000
0.0000  (   0.00%)
register           5.0058           0.5286        1.2794e-02
5.5472  (   0.46%)
sequential         0.2825           0.1564        1.8992e-03
0.4408  (   0.04%)
combinational    406.7051         799.4918            4.1513
1.2111e+03  (  99.51%)
--------------------------------------------------------------
------------------------------------
Total            411.9933 mW      800.1769 mW         4.1660
mW     1.2171e+03 mW
1
```
