# Supplementary material for: Parallel point-multiplication architecture using combined group operations for high-speed cryptographic applications
Source: PLoS One. 2017 May 1;12(5):e0176214. doi: 10.1371/journal.pone.0176214 (PMC5411040; doi:10.1371/journal.pone.0176214)
Supplement: S1 Supporting Information — (ZIP) [file pone.0176214.s001.zip › S1 Supporting Information/S1 File21 Table3_[i].pdf]

```
****************************************
Report : area
Design : ECC_TOP_K_163
Version: F-2011.09-SP3
Date   : Sun Oct  9 14:59:58 2016
****************************************

Library(s) Used:

    CORE65LPLVT (File: /usr/local-
eit/cad2/cmpstm/stm065v536/CORE65LPLVT_
5.1/libs/CORE65LPLVT_nom_1.20V_25C.db)

Number of ports:                          493
Number of nets:                          4433
Number of cells:                         1988
Number of combinational cells:           1485
Number of sequential cells:               498
Number of macros:                           0
Number of buf/inv:                        969
Number of references:                      43

Combinational area:       3420384.710002
Noncombinational area:    10986.559817
Net Interconnect area:      undefined  (Wire load has zero net
area)

Total cell area:          3431371.269819
Total area:                 undefined

Hierarchical area distribution
------------------------------


                                  Global cell area
Local cell area
                                  --------------------
--------------------------------
Hierarchical cell                 Absolute      Percent
Combi-        Noncombi-   Black
                                  Total         Total
national      national    boxes   Design
--------------------------------  ------------  -------
------------  ----------  ------  ------------
ECC_TOP_K_163                     3431371.2698    100.0
6417.8399   5892.1200  0.0000  ECC_TOP_K_163
uut_MUX1_new                          2216.7600      0.1
2216.7600      0.0000  0.0000  MUX_1_new
uut_MUX2_new                          3855.2799      0.1
3855.2799      0.0000  0.0000  MUX_2_new
uut_MUX3                              5179.7198      0.2
85.2800   5094.4398  0.0000  Reg_MUX_3
uut_PD_PA_Jac_163                 3406314.5503     99.3
3814.7199      0.0000  0.0000  PD_PA_BF
uut_PD_PA_Jac_163/Add_A1_PA           1769.0400      0.1
1769.0400      0.0000  0.0000  pol_add_8
uut_PD_PA_Jac_163/Add_A1_PD            836.6800      0.0
```

```
836.6800       0.0000  0.0000  pol_add_0
uut_PD_PA_Jac_163/Add_A2_PA            1719.6400       0.1
1719.6400       0.0000  0.0000  pol_add_6
uut_PD_PA_Jac_163/Add_A2_PD             790.4000       0.0
790.4000       0.0000  0.0000  pol_add_10
uut_PD_PA_Jac_163/Add_A3_PA            1728.4799       0.1
1728.4799       0.0000  0.0000  pol_add_5
uut_PD_PA_Jac_163/Add_A3_PD             965.1200       0.0
965.1200       0.0000  0.0000  pol_add_9
uut_PD_PA_Jac_163/Add_A4_PA            1444.5600       0.0
1444.5600       0.0000  0.0000  pol_add_4
uut_PD_PA_Jac_163/Add_A4_PD            1016.6000       0.0
1016.6000       0.0000  0.0000  pol_add_7
uut_PD_PA_Jac_163/Add_A5_PA            1129.9600       0.0
1129.9600       0.0000  0.0000  pol_add_3
uut_PD_PA_Jac_163/Add_A6_PA            1797.1199       0.1
1797.1199       0.0000  0.0000  pol_add_2
uut_PD_PA_Jac_163/Add_A7_PA            1152.3200       0.0
1152.3200       0.0000  0.0000  pol_add_1
uut_PD_PA_Jac_163/SQ_SQ1_PA           23449.3997       0.7
23449.3997       0.0000  0.0000  pol_SQ_3
uut_PD_PA_Jac_163/SQ_SQ1_PD           22153.0398       0.6
22153.0398       0.0000  0.0000  pol_SQ_0
uut_PD_PA_Jac_163/SQ_SQ2_PA           19009.1198       0.6
19009.1198       0.0000  0.0000  pol_SQ_2
uut_PD_PA_Jac_163/SQ_SQ2_PD           18153.7198       0.5
18153.7198       0.0000  0.0000  pol_SQ_7
uut_PD_PA_Jac_163/SQ_SQ3_PA           18783.4398       0.5
18783.4398       0.0000  0.0000  pol_SQ_1
uut_PD_PA_Jac_163/SQ_SQ3_PD           18734.0398       0.5
18734.0398       0.0000  0.0000  pol_SQ_6
uut_PD_PA_Jac_163/SQ_SQ4_PD           17758.5198       0.5
17758.5198       0.0000  0.0000  pol_SQ_5
uut_PD_PA_Jac_163/SQ_SQ5_PD           18426.7198       0.5
18426.7198       0.0000  0.0000  pol_SQ_4
uut_PD_PA_Jac_163/mult_M10_PA        197324.3984       5.8
197324.3984       0.0000  0.0000  pol_mult_2
uut_PD_PA_Jac_163/mult_M11_PA        205776.4781       6.0
205776.4781       0.0000  0.0000  pol_mult_1
uut_PD_PA_Jac_163/mult_M1_PA         203767.1980       5.9
203767.1980       0.0000  0.0000  pol_mult_12
uut_PD_PA_Jac_163/mult_M1_PD         193568.4384       5.6
193568.4384       0.0000  0.0000  pol_mult_0
uut_PD_PA_Jac_163/mult_M2_PA         224085.1534       6.5
224085.1534       0.0000  0.0000  pol_mult_11
uut_PD_PA_Jac_163/mult_M2_PD         203858.7183       5.9
203858.7183       0.0000  0.0000  pol_mult_15
uut_PD_PA_Jac_163/mult_M3_PA         224618.6731       6.5
224618.6731       0.0000  0.0000  pol_mult_10
uut_PD_PA_Jac_163/mult_M3_PD         190586.2386       5.6
190586.2386       0.0000  0.0000  pol_mult_14
uut_PD_PA_Jac_163/mult_M4_PA         208300.5580       6.1
208300.5580       0.0000  0.0000  pol_mult_8
uut_PD_PA_Jac_163/mult_M4_PD         196391.5130       5.7
196391.5130       0.0000  0.0000  pol_mult_13
uut_PD_PA_Jac_163/mult_M5_PA         195375.9585       5.7
195375.9585       0.0000  0.0000  pol_mult_7
```

```
uut_PD_PA_Jac_163/mult_M5_PD       189266.9987      5.5
189266.9987      0.0000  0.0000  pol_mult_9
uut_PD_PA_Jac_163/mult_M6_PA       203442.7182      5.9
203442.7182      0.0000  0.0000  pol_mult_5
uut_PD_PA_Jac_163/mult_M7_PA       189869.1585      5.5
189869.1585      0.0000  0.0000  pol_mult_6
uut_PD_PA_Jac_163/mult_M8_PA       209535.5580      6.1
209535.5580      0.0000  0.0000  pol_mult_3
uut_PD_PA_Jac_163/mult_M9_PA       195914.1530      5.7
195914.1530      0.0000  0.0000  pol_mult_4
uut_select_signal                    1495.0000      0.0
1495.0000      0.0000  0.0000  select_logic
--------------------------------  ------------  -------
------------  ----------  ------  ------------
Total
3420384.7100  10986.5598  0.0000

1
```
