# Supplementary material for: Parallel point-multiplication architecture using combined group operations for high-speed cryptographic applications
Source: PLoS One. 2017 May 1;12(5):e0176214. doi: 10.1371/journal.pone.0176214 (PMC5411040; doi:10.1371/journal.pone.0176214)
Supplement: S1 Supporting Information — (ZIP) [file pone.0176214.s001.zip › S1 Supporting Information/S1 File22 Table3_[i].pdf]

```
Information: Updating design information... (UID-85)
Warning: Design 'ECC_TOP_B_163' contains 2 high-fanout nets. A
fanout number of 1000 will be used for delay calculations
involving these nets. (TIM-134)

****************************************
Report : timing
        -path full
        -delay max
        -max_paths 1
Design : ECC_TOP_B_163
Version: F-2011.09-SP3
Date   : Tue Oct 11 06:04:08 2016
****************************************

 # A fanout number of 1000 was used for high fanout net
computations.

Operating Conditions: nom_1.20V_25C   Library: CORE65LPLVT
Wire Load Model Mode: enclosed

  Startpoint: uut_MUX3/QZout_reg[161]
              (rising edge-triggered flip-flop clocked by clk)
  Endpoint: QY_reg[152]
            (rising edge-triggered flip-flop clocked by clk)
  Path Group: clk
  Path Type: max

  Des/Clust/Port     Wire Load Model       Library
  ------------------------------------------------
  ECC_TOP_B_163      area_780Kto1170K      CORE65LPLVT
  PD_PA_BF           area_780Kto1170K      CORE65LPLVT
  pol_SQ_0           area_18Kto24K         CORE65LPLVT
  pol_SQ_6           area_18Kto24K         CORE65LPLVT
  pol_SQ_4           area_18Kto24K         CORE65LPLVT
  pol_mult_13        area_156Kto234K       CORE65LPLVT
  pol_mult_9         area_156Kto234K       CORE65LPLVT
  pol_add_7          area_1Kto2K           CORE65LPLVT

  Point                                                    Incr
Path
  ---------------------------------------------------------------
--------------
  clock clk (rise edge)                                    0.00
0.00
  clock network delay (ideal)                              0.00
0.00
  uut_MUX3/QZout_reg[161]/CP (HS65_LL_DFPRQX9)             0.00
0.00 r
  uut_MUX3/QZout_reg[161]/Q (HS65_LL_DFPRQX9)              0.08
0.08 r
  uut_MUX3/QZout[161] (Reg_MUX_3)                          0.00
0.08 r
  uut_PD_PA_Jac_163/Z1[161] (PD_PA_BF)                     0.00
0.08 r
  uut_PD_PA_Jac_163/U81/Z (HS65_LL_BFX53)                  0.04
0.11 r
```

```
  uut_PD_PA_Jac_163/SQ_SQ1_PD/A[161] (pol_SQ_0)           0.00
0.11 r
  uut_PD_PA_Jac_163/SQ_SQ1_PD/U219/Z (HS65_LL_IVX31)      0.01
0.13 f
  uut_PD_PA_Jac_163/SQ_SQ1_PD/U218/Z (HS65_LL_IVX22)      0.02
0.15 r
  uut_PD_PA_Jac_163/SQ_SQ1_PD/U975/Z (HS65_LL_NAND2X43)
                                                          0.02
0.17 f
  uut_PD_PA_Jac_163/SQ_SQ1_PD/U2015/Z (HS65_LL_IVX106)
                                                          0.03
0.19 r
  uut_PD_PA_Jac_163/SQ_SQ1_PD/U973/Z (HS65_LL_BFX71)      0.04
0.23 r
  uut_PD_PA_Jac_163/SQ_SQ1_PD/U301/Z (HS65_LL_IVX22)      0.01
0.24 f
  uut_PD_PA_Jac_163/SQ_SQ1_PD/U2185/Z (HS65_LL_OAI212X5)
                                                          0.03
0.28 r
  uut_PD_PA_Jac_163/SQ_SQ1_PD/U2190/Z (HS65_LL_XOR2X18)
                                                          0.06
0.33 f
  uut_PD_PA_Jac_163/SQ_SQ1_PD/U2192/Z (HS65_LL_XOR2X35)
                                                          0.06
0.39 f
  uut_PD_PA_Jac_163/SQ_SQ1_PD/U1103/Z (HS65_LL_XNOR2X18)
                                                          0.04
0.43 r
  uut_PD_PA_Jac_163/SQ_SQ1_PD/U1102/Z (HS65_LL_XNOR2X18)
                                                          0.06
0.49 r
  uut_PD_PA_Jac_163/SQ_SQ1_PD/U2804/Z (HS65_LL_NAND2X21)
                                                          0.02
0.50 f
  uut_PD_PA_Jac_163/SQ_SQ1_PD/U419/Z (HS65_LL_NAND2X29)
                                                          0.02
0.52 r
  uut_PD_PA_Jac_163/SQ_SQ1_PD/C[160] (pol_SQ_0)           0.00
0.52 r
  uut_PD_PA_Jac_163/SQ_SQ3_PD/A[160] (pol_SQ_6)           0.00
0.52 r
  uut_PD_PA_Jac_163/SQ_SQ3_PD/U1240/Z (HS65_LL_IVX31)     0.01
0.54 f
  uut_PD_PA_Jac_163/SQ_SQ3_PD/U531/Z (HS65_LL_IVX44)      0.02
0.56 r
  uut_PD_PA_Jac_163/SQ_SQ3_PD/U513/Z (HS65_LL_NAND2X11)
                                                          0.02
0.58 f
  uut_PD_PA_Jac_163/SQ_SQ3_PD/U400/Z (HS65_LL_XNOR2X18)
                                                          0.05
0.63 f
  uut_PD_PA_Jac_163/SQ_SQ3_PD/U1395/Z (HS65_LL_XNOR2X18)
                                                          0.05
0.68 f
  uut_PD_PA_Jac_163/SQ_SQ3_PD/U1230/Z (HS65_LL_XOR3X18)
                                                          0.09
0.77 r
```

```
  uut_PD_PA_Jac_163/SQ_SQ3_PD/U1390/Z (HS65_LLS_XNOR3X4)
                                                                0.07
0.84 f
  uut_PD_PA_Jac_163/SQ_SQ3_PD/U1382/Z (HS65_LL_XNOR2X18)
                                                                0.05
0.90 f
  uut_PD_PA_Jac_163/SQ_SQ3_PD/C[162] (pol_SQ_6)          0.00
0.90 f
  uut_PD_PA_Jac_163/SQ_SQ5_PD/A[162] (pol_SQ_4)          0.00
0.90 f
  uut_PD_PA_Jac_163/SQ_SQ5_PD/U1417/Z (HS65_LL_IVX31)     0.02
0.91 r
  uut_PD_PA_Jac_163/SQ_SQ5_PD/U1419/Z (HS65_LL_NAND2AX29)
                                                                0.04
0.95 r
  uut_PD_PA_Jac_163/SQ_SQ5_PD/U865/Z (HS65_LL_IVX31)      0.02
0.97 f
  uut_PD_PA_Jac_163/SQ_SQ5_PD/U122/Z (HS65_LL_BFX62)      0.03
1.00 f
  uut_PD_PA_Jac_163/SQ_SQ5_PD/U1268/Z (HS65_LL_BFX62)     0.03
1.03 f
  uut_PD_PA_Jac_163/SQ_SQ5_PD/U1160/Z (HS65_LL_AOI22X4)
                                                                0.04
1.06 r
  uut_PD_PA_Jac_163/SQ_SQ5_PD/U1649/Z (HS65_LL_OAI212X5)
                                                                0.05
1.11 f
  uut_PD_PA_Jac_163/SQ_SQ5_PD/U483/Z (HS65_LL_XOR2X35)
                                                                0.07
1.18 f
  uut_PD_PA_Jac_163/SQ_SQ5_PD/U482/Z (HS65_LLS_XNOR2X24)
                                                                0.04
1.22 r
  uut_PD_PA_Jac_163/SQ_SQ5_PD/U2348/Z (HS65_LL_XNOR2X18)
                                                                0.05
1.26 r
  uut_PD_PA_Jac_163/SQ_SQ5_PD/U680/Z (HS65_LL_XNOR2X35)
                                                                0.04
1.30 f
  uut_PD_PA_Jac_163/SQ_SQ5_PD/U2930/Z (HS65_LLS_XNOR2X24)
                                                                0.04
1.35 r
  uut_PD_PA_Jac_163/SQ_SQ5_PD/C[122] (pol_SQ_4)          0.00
1.35 r
  uut_PD_PA_Jac_163/mult_M4_PD/B[122] (pol_mult_13)       0.00
1.35 r
  uut_PD_PA_Jac_163/mult_M4_PD/U4594/Z (HS65_LL_IVX27)
                                                                0.02
1.37 f
  uut_PD_PA_Jac_163/mult_M4_PD/U11939/Z (HS65_LL_BFX71)
                                                                0.03
1.40 f
  uut_PD_PA_Jac_163/mult_M4_PD/U12359/Z (HS65_LL_IVX31)
                                                                0.02
1.42 r
  uut_PD_PA_Jac_163/mult_M4_PD/U11937/Z (HS65_LL_NAND2X7)
                                                                0.02
```

```
1.44 f
  uut_PD_PA_Jac_163/mult_M4_PD/U29287/Z (HS65_LL_XNOR2X18)
                                                          0.05
1.49 f
  uut_PD_PA_Jac_163/mult_M4_PD/U7286/Z (HS65_LL_XOR2X18)
                                                          0.05
1.55 f
  uut_PD_PA_Jac_163/mult_M4_PD/U7016/Z (HS65_LLS_XNOR2X12)
                                                          0.04
1.59 r
  uut_PD_PA_Jac_163/mult_M4_PD/U7015/Z (HS65_LLS_XNOR2X12)
                                                          0.05
1.63 f
  uut_PD_PA_Jac_163/mult_M4_PD/U22343/Z (HS65_LLS_XNOR2X12)
                                                          0.04
1.67 r
  uut_PD_PA_Jac_163/mult_M4_PD/U914/Z (HS65_LLS_XNOR2X12)
                                                          0.04
1.72 f
  uut_PD_PA_Jac_163/mult_M4_PD/U11962/Z (HS65_LL_XOR2X18)
                                                          0.05
1.76 r
  uut_PD_PA_Jac_163/mult_M4_PD/U2990/Z (HS65_LLS_XNOR2X18)
                                                          0.04
1.80 r
  uut_PD_PA_Jac_163/mult_M4_PD/U19549/Z (HS65_LL_XOR2X35)
                                                          0.07
1.87 f
  uut_PD_PA_Jac_163/mult_M4_PD/U19548/Z (HS65_LLS_XNOR2X24)
                                                          0.04
1.91 r
  uut_PD_PA_Jac_163/mult_M4_PD/U36459/Z (HS65_LLS_XNOR2X24)
                                                          0.04
1.95 r
  uut_PD_PA_Jac_163/mult_M4_PD/C[64] (pol_mult_13)        0.00
1.95 r
  uut_PD_PA_Jac_163/Add_A3_PD/A[64] (pol_add_9)           0.00
1.95 r
  uut_PD_PA_Jac_163/Add_A3_PD/U179/Z (HS65_LL_XOR2X35)
                                                          0.05
2.01 f
  uut_PD_PA_Jac_163/Add_A3_PD/C[64] (pol_add_9)           0.00
2.01 f
  uut_PD_PA_Jac_163/mult_M5_PD/A[64] (pol_mult_9)         0.00
2.01 f
  uut_PD_PA_Jac_163/mult_M5_PD/U24600/Z (HS65_LL_BFX106)
                                                          0.04
2.05 f
  uut_PD_PA_Jac_163/mult_M5_PD/U5612/Z (HS65_LL_AND2X35)
                                                          0.04
2.08 f
  uut_PD_PA_Jac_163/mult_M5_PD/U5416/Z (HS65_LL_BFX62)
                                                          0.03
2.12 f
  uut_PD_PA_Jac_163/mult_M5_PD/U5896/Z (HS65_LL_BFX106)
                                                          0.03
2.15 f
```

```
  uut_PD_PA_Jac_163/mult_M5_PD/U30370/Z (HS65_LL_AOI22X3)
                                                            0.05
2.20 r
  uut_PD_PA_Jac_163/mult_M5_PD/U5893/Z (HS65_LL_OAI212X5)
                                                            0.05
2.25 f
  uut_PD_PA_Jac_163/mult_M5_PD/U26800/Z (HS65_LL_XNOR2X18)
                                                            0.06
2.31 f
  uut_PD_PA_Jac_163/mult_M5_PD/U3489/Z (HS65_LLS_XNOR2X6)
                                                            0.04
2.35 r
  uut_PD_PA_Jac_163/mult_M5_PD/U14825/Z (HS65_LL_XOR2X18)
                                                            0.05
2.40 f
  uut_PD_PA_Jac_163/mult_M5_PD/U28497/Z (HS65_LL_XOR2X18)
                                                            0.04
2.44 f
  uut_PD_PA_Jac_163/mult_M5_PD/U8192/Z (HS65_LL_XOR2X35)
                                                            0.05
2.50 f
  uut_PD_PA_Jac_163/mult_M5_PD/U22842/Z (HS65_LL_IVX18)
                                                            0.02
2.51 r
  uut_PD_PA_Jac_163/mult_M5_PD/U5232/Z (HS65_LL_NAND2X21)
                                                            0.01
2.53 f
  uut_PD_PA_Jac_163/mult_M5_PD/U2970/Z (HS65_LL_NAND2X29)
                                                            0.01
2.54 r
  uut_PD_PA_Jac_163/mult_M5_PD/U14026/Z (HS65_LLS_XOR3X4)
                                                            0.07
2.61 f
  uut_PD_PA_Jac_163/mult_M5_PD/U29869/Z (HS65_LL_XOR2X18)
                                                            0.05
2.66 r
  uut_PD_PA_Jac_163/mult_M5_PD/U4252/Z (HS65_LL_XOR2X35)
                                                            0.04
2.70 r
  uut_PD_PA_Jac_163/mult_M5_PD/C[152] (pol_mult_9)          0.00
2.70 r
  uut_PD_PA_Jac_163/Add_A4_PD/A[152] (pol_add_7)            0.00
2.70 r
  uut_PD_PA_Jac_163/Add_A4_PD/U37/Z (HS65_LL_IVX18)         0.01
2.71 f
  uut_PD_PA_Jac_163/Add_A4_PD/U36/Z (HS65_LL_NAND2X14)
                                                            0.01
2.73 r
  uut_PD_PA_Jac_163/Add_A4_PD/U138/Z (HS65_LL_NAND2X21)
                                                            0.02
2.74 f
  uut_PD_PA_Jac_163/Add_A4_PD/C[152] (pol_add_7)            0.00
2.74 f
  uut_PD_PA_Jac_163/Y3_PD[152] (PD_PA_BF)                   0.00
2.74 f
  uut_MUX2_new/Y3_PD[152] (MUX_2_new)                       0.00
2.74 f
```

```
  uut_MUX2_new/U58/Z (HS65_LL_MUX21X18)                        0.04
2.79 f
  uut_MUX2_new/sQY[152] (MUX_2_new)                            0.00
2.79 f
  U669/Z (HS65_LL_MUX21X18)                                    0.05
2.83 f
  QY_reg[152]/D (HS65_LLS_DFPRQX35)                            0.00
2.83 f
  data arrival time
2.83

  clock clk (rise edge)                                        3.00
3.00
  clock network delay (ideal)                                  0.00
3.00
  clock uncertainty                                           -0.10
2.90
  QY_reg[152]/CP (HS65_LLS_DFPRQX35)                           0.00
2.90 r
  library setup time                                          -0.07
2.83
  data required time
2.83
  -------------------------------------------------------------
--------------
  data required time
2.83
  data arrival time
-2.83
  -------------------------------------------------------------
--------------
  slack (MET)
0.00


1
```
