# Supplementary material for: Parallel point-multiplication architecture using combined group operations for high-speed cryptographic applications
Source: PLoS One. 2017 May 1;12(5):e0176214. doi: 10.1371/journal.pone.0176214 (PMC5411040; doi:10.1371/journal.pone.0176214)
Supplement: S1 Supporting Information — (ZIP) [file pone.0176214.s001.zip › S1 Supporting Information/S1 File24 Table3_[j].pdf]

```
****************************************
Report : area
Design : ECC_TOP_B_163
Version: F-2011.09-SP3
Date   : Tue Oct 11 06:04:11 2016
****************************************

Library(s) Used:

    CORE65LPLVT (File: /usr/local-
eit/cad2/cmpstm/stm065v536/CORE65LPLVT_
5.1/libs/CORE65LPLVT_nom_1.20V_25C.db)

Number of ports:                          656
Number of nets:                          5152
Number of cells:                         2545
Number of combinational cells:           2042
Number of sequential cells:               498
Number of macros:                           0
Number of buf/inv:                       1394
Number of references:                      67

Combinational area:       3473722.656627
Noncombinational area:    13889.199791
Net Interconnect area:      undefined  (Wire load has zero net
area)

Total cell area:          3487611.856418
Total area:                 undefined

Hierarchical area distribution
------------------------------


                                  Global cell area
Local cell area
                                  ---------------------
--------------------------------
Hierarchical cell                 Absolute      Percent
Combi-        Noncombi-   Black
                                  Total         Total
national      national    boxes   Design
--------------------------------  ------------  -------
------------  ----------  ------  ------------
ECC_TOP_B_163                     3487611.8564    100.0
7684.0398   5428.2799  0.0000  ECC_TOP_B_163
uut_MUX1_new                         5047.1201      0.1
1741.4800   3305.6401  0.0000  MUX_1_new
uut_MUX2_new                         3702.3999      0.1
3702.3999      0.0000  0.0000  MUX_2_new
uut_MUX3                             5231.7198      0.2
76.4400   5155.2798  0.0000  Reg_MUX_3
uut_PD_PA_Jac_163                 3459295.7769     99.2
2060.2399      0.0000  0.0000  PD_PA_BF
uut_PD_PA_Jac_163/Add_A1_PA           707.2000      0.0
707.2000      0.0000  0.0000  pol_add_8
uut_PD_PA_Jac_163/Add_A1_PD           872.5600      0.0
```

```
872.5600      0.0000  0.0000  pol_add_0
uut_PD_PA_Jac_163/Add_A2_PA             705.1200      0.0
705.1200      0.0000  0.0000  pol_add_6
uut_PD_PA_Jac_163/Add_A2_PD             995.2800      0.0
995.2800      0.0000  0.0000  pol_add_10
uut_PD_PA_Jac_163/Add_A3_PA             679.1200      0.0
679.1200      0.0000  0.0000  pol_add_5
uut_PD_PA_Jac_163/Add_A3_PD            2109.6399      0.1
2109.6399      0.0000  0.0000  pol_add_9
uut_PD_PA_Jac_163/Add_A4_PA             678.0800      0.0
678.0800      0.0000  0.0000  pol_add_4
uut_PD_PA_Jac_163/Add_A4_PD            2153.8399      0.1
2153.8399      0.0000  0.0000  pol_add_7
uut_PD_PA_Jac_163/Add_A5_PA             678.0800      0.0
678.0800      0.0000  0.0000  pol_add_3
uut_PD_PA_Jac_163/Add_A6_PA             678.0800      0.0
678.0800      0.0000  0.0000  pol_add_2
uut_PD_PA_Jac_163/Add_A7_PA             678.0800      0.0
678.0800      0.0000  0.0000  pol_add_1
uut_PD_PA_Jac_163/SQ_SQ1_PA          116734.2772      3.3
116734.2772      0.0000  0.0000  pol_SQ_3
uut_PD_PA_Jac_163/SQ_SQ1_PD           24523.7198      0.7
24523.7198      0.0000  0.0000  pol_SQ_0
uut_PD_PA_Jac_163/SQ_SQ2_PA          116706.7172      3.3
116706.7172      0.0000  0.0000  pol_SQ_2
uut_PD_PA_Jac_163/SQ_SQ2_PD           17545.8397      0.5
17545.8397      0.0000  0.0000  pol_SQ_7
uut_PD_PA_Jac_163/SQ_SQ3_PA          116522.1172      3.3
116522.1172      0.0000  0.0000  pol_SQ_1
uut_PD_PA_Jac_163/SQ_SQ3_PD           24784.2398      0.7
24784.2398      0.0000  0.0000  pol_SQ_6
uut_PD_PA_Jac_163/SQ_SQ4_PD           17670.1197      0.5
17670.1197      0.0000  0.0000  pol_SQ_5
uut_PD_PA_Jac_163/SQ_SQ5_PD           24775.9198      0.7
24775.9198      0.0000  0.0000  pol_SQ_4
uut_PD_PA_Jac_163/mult_M10_PA        180980.7966      5.2
180980.7966      0.0000  0.0000  pol_mult_2
uut_PD_PA_Jac_163/mult_M11_PA        180980.2766      5.2
180980.2766      0.0000  0.0000  pol_mult_1
uut_PD_PA_Jac_163/mult_M1_PA         181355.1966      5.2
181355.1966      0.0000  0.0000  pol_mult_12
uut_PD_PA_Jac_163/mult_M1_PD         174112.1167      5.0
174112.1167      0.0000  0.0000  pol_mult_0
uut_PD_PA_Jac_163/mult_M2_PA         178998.5567      5.1
178998.5567      0.0000  0.0000  pol_mult_11
uut_PD_PA_Jac_163/mult_M2_PD         186899.4365      5.4
186899.4365      0.0000  0.0000  pol_mult_15
uut_PD_PA_Jac_163/mult_M3_PA         178913.2767      5.1
178913.2767      0.0000  0.0000  pol_mult_10
uut_PD_PA_Jac_163/mult_M3_PD         187492.7566      5.4
187492.7566      0.0000  0.0000  pol_mult_14
uut_PD_PA_Jac_163/mult_M4_PA         181426.4366      5.2
181426.4366      0.0000  0.0000  pol_mult_8
uut_PD_PA_Jac_163/mult_M4_PD         231357.3571      6.6
231357.3571      0.0000  0.0000  pol_mult_13
uut_PD_PA_Jac_163/mult_M5_PA         181303.7166      5.2
181303.7166      0.0000  0.0000  pol_mult_7
```

```
uut_PD_PA_Jac_163/mult_M5_PD       220996.3574      6.3
220996.3574      0.0000  0.0000  pol_mult_9
uut_PD_PA_Jac_163/mult_M6_PA       181227.7966      5.2
181227.7966      0.0000  0.0000  pol_mult_5
uut_PD_PA_Jac_163/mult_M7_PA       181149.2766      5.2
181149.2766      0.0000  0.0000  pol_mult_6
uut_PD_PA_Jac_163/mult_M8_PA       181126.3966      5.2
181126.3966      0.0000  0.0000  pol_mult_3
uut_PD_PA_Jac_163/mult_M9_PA       178717.7567      5.1
178717.7567      0.0000  0.0000  pol_mult_4
uut_select_signal                    1222.5200      0.0
1222.5200      0.0000  0.0000  select_logic
--------------------------------  ------------  -------
------------  ----------  ------  ------------
Total
3473722.6566  13889.1998  0.0000

1
```
