# Supplementary material for: Parallel point-multiplication architecture using combined group operations for high-speed cryptographic applications
Source: PLoS One. 2017 May 1;12(5):e0176214. doi: 10.1371/journal.pone.0176214 (PMC5411040; doi:10.1371/journal.pone.0176214)
Supplement: S1 Supporting Information — (ZIP) [file pone.0176214.s001.zip › S1 Supporting Information/S1 File26 Table3_[j].pdf]

```
Information: Propagating switching activity (low effort zero
delay simulation). (PWR-6)
Warning: Design has unannotated primary inputs. (PWR-414)
Warning: Design has unannotated sequential cell outputs. (PWR-
415)

****************************************
Report : power
        -analysis_effort low
Design : ECC_TOP_B_163
Version: F-2011.09-SP3
Date   : Tue Oct 11 06:05:06 2016
****************************************


Library(s) Used:

    CORE65LPLVT (File: /usr/local-
eit/cad2/cmpstm/stm065v536/CORE65LPLVT_
5.1/libs/CORE65LPLVT_nom_1.20V_25C.db)


Operating Conditions: nom_1.20V_25C   Library: CORE65LPLVT
Wire Load Model Mode: enclosed

Design        Wire Load Model            Library
------------------------------------------------
ECC_TOP_B_163          area_780Kto1170K  CORE65LPLVT
PD_PA_BF               area_780Kto1170K  CORE65LPLVT
select_logic           area_1Kto2K       CORE65LPLVT
MUX_1_new              area_5Kto6K       CORE65LPLVT
MUX_2_new              area_2Kto3K       CORE65LPLVT
Reg_MUX_3              area_5Kto6K       CORE65LPLVT
pol_SQ_0               area_18Kto24K     CORE65LPLVT
pol_mult_0             area_156Kto234K   CORE65LPLVT
pol_add_0              area_0Kto1K       CORE65LPLVT
pol_SQ_1               area_78Kto156K    CORE65LPLVT
pol_SQ_2               area_78Kto156K    CORE65LPLVT
pol_SQ_3               area_78Kto156K    CORE65LPLVT
pol_SQ_4               area_18Kto24K     CORE65LPLVT
pol_SQ_5               area_12Kto18K     CORE65LPLVT
pol_SQ_6               area_18Kto24K     CORE65LPLVT
pol_SQ_7               area_12Kto18K     CORE65LPLVT
pol_mult_1             area_156Kto234K   CORE65LPLVT
pol_mult_2             area_156Kto234K   CORE65LPLVT
pol_mult_3             area_156Kto234K   CORE65LPLVT
pol_mult_4             area_156Kto234K   CORE65LPLVT
pol_mult_5             area_156Kto234K   CORE65LPLVT
pol_mult_6             area_156Kto234K   CORE65LPLVT
pol_mult_7             area_156Kto234K   CORE65LPLVT
pol_mult_8             area_156Kto234K   CORE65LPLVT
pol_mult_9             area_156Kto234K   CORE65LPLVT
pol_mult_10            area_156Kto234K   CORE65LPLVT
pol_mult_11            area_156Kto234K   CORE65LPLVT
pol_mult_12            area_156Kto234K   CORE65LPLVT
pol_mult_13            area_156Kto234K   CORE65LPLVT
pol_mult_14            area_156Kto234K   CORE65LPLVT
```

```
pol_mult_15            area_156Kto234K   CORE65LPLVT
pol_add_1              area_0Kto1K       CORE65LPLVT
pol_add_2              area_0Kto1K       CORE65LPLVT
pol_add_3              area_0Kto1K       CORE65LPLVT
pol_add_4              area_0Kto1K       CORE65LPLVT
pol_add_5              area_0Kto1K       CORE65LPLVT
pol_add_6              area_0Kto1K       CORE65LPLVT
pol_add_7              area_1Kto2K       CORE65LPLVT
pol_add_8              area_0Kto1K       CORE65LPLVT
pol_add_9              area_2Kto3K       CORE65LPLVT
pol_add_10             area_0Kto1K       CORE65LPLVT


Global Operating Voltage = 1.2
Power-specific unit information :
    Voltage Units = 1V
    Capacitance Units = 1.000000pf
    Time Units = 1ns
    Dynamic Power Units = 1mW    (derived from V,C,T units)
    Leakage Power Units = 1mW


  Cell Internal Power  = 178.0932 mW   (37%)
  Net Switching Power  = 307.7535 mW   (63%)
                         ---------
Total Dynamic Power    = 485.8466 mW  (100%)

Cell Leakage Power     =   1.8318 mW


                 Internal         Switching           Leakage
Total
Power Group      Power            Power               Power
Power   (   %    )  Attrs
--------------------------------------------------------------
------------------------------------
io_pad             0.0000           0.0000              0.0000
0.0000  (   0.00%)
memory             0.0000           0.0000              0.0000
0.0000  (   0.00%)
black_box          0.0000           0.0000              0.0000
0.0000  (   0.00%)
clock_network      0.0000           0.0000              0.0000
0.0000  (   0.00%)
register           3.5067           0.5406          6.6003e-03
4.0539  (   0.83%)
sequential         0.1994           0.1231          1.3274e-03
0.3238  (   0.07%)
combinational    174.3383         307.0893              1.8246
483.1473  (  99.10%)
--------------------------------------------------------------
------------------------------------
Total            178.0443 mW      307.7530 mW           1.8325
mW       487.5250 mW
1
```
