# Supplementary material for: Parallel point-multiplication architecture using combined group operations for high-speed cryptographic applications
Source: PLoS One. 2017 May 1;12(5):e0176214. doi: 10.1371/journal.pone.0176214 (PMC5411040; doi:10.1371/journal.pone.0176214)
Supplement: S1 Supporting Information — (ZIP) [file pone.0176214.s001.zip › S1 Supporting Information/S1 File7 Table2_[b].pdf]

```
Release 14.7 - xst P.20131013 (nt64)
Copyright (c) 1995-2013 Xilinx, Inc.  All rights reserved.
--> Parameter TMPDIR set to xst/projnav.tmp


Total REAL time to Xst completion: 0.00 secs
Total CPU time to Xst completion: 0.09 secs

--> Parameter xsthdpdir set to xst


Total REAL time to Xst completion: 0.00 secs
Total CPU time to Xst completion: 0.09 secs

--> Reading design: ECC_TOP_B_163.prj

TABLE OF CONTENTS
  1) Synthesis Options Summary
  2) HDL Parsing
  3) HDL Elaboration
  4) HDL Synthesis
       4.1) HDL Synthesis Report
  5) Advanced HDL Synthesis
       5.1) Advanced HDL Synthesis Report
  6) Low Level Synthesis
  7) Partition Report
  8) Design Summary
       8.1) Primitive and Black Box Usage
       8.2) Device utilization summary
       8.3) Partition Resource Summary
       8.4) Timing Report
            8.4.1) Clock Information
            8.4.2) Asynchronous Control Signals Information
            8.4.3) Timing Summary
            8.4.4) Timing Details
            8.4.5) Cross Clock Domains Report


=========================================================================
*                      Synthesis Options Summary                        *
=========================================================================
---- Source Parameters
Input File Name                    : "ECC_TOP_B_163.prj"
Ignore Synthesis Constraint File   : NO

---- Target Parameters
Output File Name                   : "ECC_TOP_B_163"
Output Format                      : NGC
Target Device                      : xc7vx485t-2-ffg1761

---- Source Options
Top Module Name                    : ECC_TOP_B_163
Automatic FSM Extraction           : YES
FSM Encoding Algorithm             : Auto
Safe Implementation                : No
FSM Style                          : LUT
RAM Extraction                     : Yes
RAM Style                          : Auto
ROM Extraction                     : Yes
Shift Register Extraction          : YES
ROM Style                          : Auto
Resource Sharing                   : YES
Asynchronous To Synchronous        : NO
Shift Register Minimum Size        : 2
Use DSP Block                      : Auto
Automatic Register Balancing       : No

---- Target Options
LUT Combining                      : Auto
Reduce Control Sets                : Auto
Add IO Buffers                     : YES
```

```
Global Maximum Fanout                  : 100000
Add Generic Clock Buffer(BUFG)         : 32
Register Duplication                   : YES
Optimize Instantiated Primitives       : NO
Use Clock Enable                       : Auto
Use Synchronous Set                    : Auto
Use Synchronous Reset                  : Auto
Pack IO Registers into IOBs            : Auto
Equivalent register Removal            : YES

---- General Options
Optimization Goal                      : Speed
Optimization Effort                    : 1
Power Reduction                        : NO
Keep Hierarchy                         : No
Netlist Hierarchy                      : As_Optimized
RTL Output                             : Yes
Global Optimization                    : AllClockNets
Read Cores                             : YES
Write Timing Constraints               : NO
Cross Clock Analysis                   : NO
Hierarchy Separator                    : /
Bus Delimiter                          : <>
Case Specifier                         : Maintain
Slice Utilization Ratio                : 100
BRAM Utilization Ratio                 : 100
DSP48 Utilization Ratio                : 100
Auto BRAM Packing                      : NO
Slice Utilization Ratio Delta          : 5

=========================================================================



=========================================================================
*                           HDL Parsing                                 *
=========================================================================
Parsing VHDL file "G:\VHDL_July_2016\PM_PDPA_BF_P_All\PM_PDPA_BF_P_B_163\ECC_package_BF.vhd"
into library work
Parsing package <ECC_package_BF>.
Parsing VHDL file "G:\VHDL_July_2016\PM_PDPA_BF_P_All\PM_PDPA_BF_P_B_163\pol_SQ.vhd" into
library work
Parsing entity <pol_SQ>.
Parsing architecture <arch_pol_SQ> of entity <pol_sq>.
Parsing VHDL file "G:\VHDL_July_2016\PM_PDPA_BF_P_All\PM_PDPA_BF_P_B_163\pol_add.vhd" into
library work
Parsing entity <pol_add>.
Parsing architecture <arch_pol_add> of entity <pol_add>.
Parsing VHDL file "G:\VHDL_July_2016\PM_PDPA_BF_P_All\PM_PDPA_BF_P_B_163\mult_k_163.vhd"
into library work
Parsing entity <pol_mult>.
Parsing architecture <arch_pol_mult> of entity <pol_mult>.
Parsing VHDL file "G:\VHDL_July_2016\PM_PDPA_BF_P_All\PM_PDPA_BF_P_B_163\select_logic.vhd"
into library work
Parsing entity <select_logic>.
Parsing architecture <arch_select_logic> of entity <select_logic>.
Parsing VHDL file "G:\VHDL_July_2016\PM_PDPA_BF_P_All\PM_PDPA_BF_P_B_163\Reg_MUX_3.vhd" into
library work
Parsing entity <Reg_MUX_3>.
Parsing architecture <arch_Reg_MUX_3> of entity <reg_mux_3>.
Parsing VHDL file "G:\VHDL_July_2016\PM_PDPA_BF_P_All\PM_PDPA_BF_P_B_163\PD_PA_BF.vhd" into
library work
Parsing entity <PD_PA_BF>.
Parsing architecture <arch_PD_PA_BF> of entity <pd_pa_bf>.
Parsing VHDL file "G:\VHDL_July_2016\PM_PDPA_BF_P_All\PM_PDPA_BF_P_B_163\MUX_2_new.vhd" into
library work
Parsing entity <MUX_2_new>.
Parsing architecture <arch_MUX_2_new> of entity <mux_2_new>.
Parsing VHDL file "G:\VHDL_July_2016\PM_PDPA_BF_P_All\PM_PDPA_BF_P_B_163\MUX_1_new.vhd" into
library work
Parsing entity <MUX_1_new>.
Parsing architecture <arch_MUX_1_new> of entity <mux_1_new>.
```

```
Parsing VHDL file "G:\VHDL_July_2016\PM_PDPA_BF_P_All\PM_PDPA_BF_P_B_163\ECC_TOP_K_163.vhd"
into library work
Parsing entity <ECC_TOP_B_163>.
Parsing architecture <arch_ECC_TOP_k_163> of entity <ecc_top_b_163>.

=========================================================================
*                            HDL Elaboration                            *
=========================================================================

Elaborating entity <ECC_TOP_B_163> (architecture <arch_ECC_TOP_k_163>) from library <work>.

Elaborating entity <PD_PA_BF> (architecture <arch_PD_PA_BF>) from library <work>.

Elaborating entity <pol_SQ> (architecture <arch_pol_SQ>) from library <work>.

Elaborating entity <pol_mult> (architecture <arch_pol_mult>) from library <work>.

Elaborating entity <pol_add> (architecture <arch_pol_add>) from library <work>.

Elaborating entity <select_logic> (architecture <arch_select_logic>) from library <work>.

Elaborating entity <MUX_1_new> (architecture <arch_MUX_1_new>) from library <work>.

Elaborating entity <MUX_2_new> (architecture <arch_MUX_2_new>) from library <work>.

Elaborating entity <Reg_MUX_3> (architecture <arch_Reg_MUX_3>) from library <work>.

=========================================================================
*                             HDL Synthesis                             *
=========================================================================

Synthesizing Unit <ECC_TOP_B_163>.
    Related source file is
    "G:\VHDL_July_2016\PM_PDPA_BF_P_All\PM_PDPA_BF_P_B_163\ECC_TOP_K_163.vhd".
WARNING:Xst:647 - Input <start> is never used. This port will be preserved and left
unconnected if it belongs to a top-level block or it belongs to a sub-block and the
hierarchy of this sub-block is preserved.
    Found 163-bit register for signal <QX>.
    Found 163-bit register for signal <QY>.
    Found 163-bit register for signal <QZ>.
    Found 8-bit register for signal <count>.
    Found 1-bit register for signal <done>.
    Found 8-bit subtractor for signal <GND_7_o_GND_7_o_sub_2_OUT<7:0>> created at line 113.
    Summary:
    inferred   1 Adder/Subtractor(s).
    inferred 498 D-type flip-flop(s).
    inferred   1 Multiplexer(s).
Unit <ECC_TOP_B_163> synthesized.

Synthesizing Unit <PD_PA_BF>.
    Related source file is
    "G:\VHDL_July_2016\PM_PDPA_BF_P_All\PM_PDPA_BF_P_B_163\PD_PA_BF.vhd".
    Summary:
    no macro.
Unit <PD_PA_BF> synthesized.

Synthesizing Unit <pol_SQ>.
    Related source file is "G:\VHDL_July_2016\PM_PDPA_BF_P_All\PM_PDPA_BF_P_B_163\pol_SQ.vhd".
    Summary:
Unit <pol_SQ> synthesized.

Synthesizing Unit <pol_mult>.
    Related source file is
    "G:\VHDL_July_2016\PM_PDPA_BF_P_All\PM_PDPA_BF_P_B_163\mult_k_163.vhd".
    Summary:
Unit <pol_mult> synthesized.

Synthesizing Unit <pol_add>.
    Related source file is
    "G:\VHDL_July_2016\PM_PDPA_BF_P_All\PM_PDPA_BF_P_B_163\pol_add.vhd".
    Summary:
```

```
Unit <pol_add> synthesized.

Synthesizing Unit <select_logic>.
    Related source file is
    "G:\VHDL_July_2016\PM_PDPA_BF_P_All\PM_PDPA_BF_P_B_163\select_logic.vhd".
    Summary:
    inferred   1 Multiplexer(s).
Unit <select_logic> synthesized.

Synthesizing Unit <MUX_1_new>.
    Related source file is
    "G:\VHDL_July_2016\PM_PDPA_BF_P_All\PM_PDPA_BF_P_B_163\MUX_1_new.vhd".
WARNING:Xst:737 - Found 1-bit latch for signal <PA_X3<161>>. Latches may be generated from
incomplete case or if statements. We do not recommend the use of latches in FPGA/CPLD
designs, as they may lead to timing problems.
WARNING:Xst:737 - Found 1-bit latch for signal <PA_X3<160>>. Latches may be generated from
incomplete case or if statements. We do not recommend the use of latches in FPGA/CPLD
designs, as they may lead to timing problems.
WARNING:Xst:737 - Found 1-bit latch for signal <PA_X3<159>>. Latches may be generated from
incomplete case or if statements. We do not recommend the use of latches in FPGA/CPLD
designs, as they may lead to timing problems.
WARNING:Xst:737 - Found 1-bit latch for signal <PA_X3<158>>. Latches may be generated from
incomplete case or if statements. We do not recommend the use of latches in FPGA/CPLD
designs, as they may lead to timing problems.
WARNING:Xst:737 - Found 1-bit latch for signal <PA_X3<157>>. Latches may be generated from
incomplete case or if statements. We do not recommend the use of latches in FPGA/CPLD
designs, as they may lead to timing problems.
WARNING:Xst:737 - Found 1-bit latch for signal <PA_X3<156>>. Latches may be generated from
incomplete case or if statements. We do not recommend the use of latches in FPGA/CPLD
designs, as they may lead to timing problems.
WARNING:Xst:737 - Found 1-bit latch for signal <PA_X3<155>>. Latches may be generated from
incomplete case or if statements. We do not recommend the use of latches in FPGA/CPLD
designs, as they may lead to timing problems.
WARNING:Xst:737 - Found 1-bit latch for signal <PA_X3<154>>. Latches may be generated from
incomplete case or if statements. We do not recommend the use of latches in FPGA/CPLD
designs, as they may lead to timing problems.
WARNING:Xst:737 - Found 1-bit latch for signal <PA_X3<153>>. Latches may be generated from
incomplete case or if statements. We do not recommend the use of latches in FPGA/CPLD
designs, as they may lead to timing problems.
WARNING:Xst:737 - Found 1-bit latch for signal <PA_X3<152>>. Latches may be generated from
incomplete case or if statements. We do not recommend the use of latches in FPGA/CPLD
designs, as they may lead to timing problems.
WARNING:Xst:737 - Found 1-bit latch for signal <PA_X3<151>>. Latches may be generated from
incomplete case or if statements. We do not recommend the use of latches in FPGA/CPLD
designs, as they may lead to timing problems.
WARNING:Xst:737 - Found 1-bit latch for signal <PA_X3<150>>. Latches may be generated from
incomplete case or if statements. We do not recommend the use of latches in FPGA/CPLD
designs, as they may lead to timing problems.
WARNING:Xst:737 - Found 1-bit latch for signal <PA_X3<149>>. Latches may be generated from
incomplete case or if statements. We do not recommend the use of latches in FPGA/CPLD
designs, as they may lead to timing problems.
WARNING:Xst:737 - Found 1-bit latch for signal <PA_X3<148>>. Latches may be generated from
incomplete case or if statements. We do not recommend the use of latches in FPGA/CPLD
designs, as they may lead to timing problems.
WARNING:Xst:737 - Found 1-bit latch for signal <PA_X3<147>>. Latches may be generated from
incomplete case or if statements. We do not recommend the use of latches in FPGA/CPLD
designs, as they may lead to timing problems.
WARNING:Xst:737 - Found 1-bit latch for signal <PA_X3<146>>. Latches may be generated from
incomplete case or if statements. We do not recommend the use of latches in FPGA/CPLD
designs, as they may lead to timing problems.
WARNING:Xst:737 - Found 1-bit latch for signal <PA_X3<145>>. Latches may be generated from
incomplete case or if statements. We do not recommend the use of latches in FPGA/CPLD
designs, as they may lead to timing problems.
WARNING:Xst:737 - Found 1-bit latch for signal <PA_X3<144>>. Latches may be generated from
incomplete case or if statements. We do not recommend the use of latches in FPGA/CPLD
designs, as they may lead to timing problems.
WARNING:Xst:737 - Found 1-bit latch for signal <PA_X3<143>>. Latches may be generated from
incomplete case or if statements. We do not recommend the use of latches in FPGA/CPLD
designs, as they may lead to timing problems.
WARNING:Xst:737 - Found 1-bit latch for signal <PA_X3<142>>. Latches may be generated from
incomplete case or if statements. We do not recommend the use of latches in FPGA/CPLD
```

designs, as they may lead to timing problems.
WARNING:Xst:737 - Found 1-bit latch for signal <PA_X3<141>>. Latches may be generated from incomplete case or if statements. We do not recommend the use of latches in FPGA/CPLD designs, as they may lead to timing problems.
WARNING:Xst:737 - Found 1-bit latch for signal <PA_X3<140>>. Latches may be generated from incomplete case or if statements. We do not recommend the use of latches in FPGA/CPLD designs, as they may lead to timing problems.
WARNING:Xst:737 - Found 1-bit latch for signal <PA_X3<139>>. Latches may be generated from incomplete case or if statements. We do not recommend the use of latches in FPGA/CPLD designs, as they may lead to timing problems.
WARNING:Xst:737 - Found 1-bit latch for signal <PA_X3<138>>. Latches may be generated from incomplete case or if statements. We do not recommend the use of latches in FPGA/CPLD designs, as they may lead to timing problems.
WARNING:Xst:737 - Found 1-bit latch for signal <PA_X3<137>>. Latches may be generated from incomplete case or if statements. We do not recommend the use of latches in FPGA/CPLD designs, as they may lead to timing problems.
WARNING:Xst:737 - Found 1-bit latch for signal <PA_X3<136>>. Latches may be generated from incomplete case or if statements. We do not recommend the use of latches in FPGA/CPLD designs, as they may lead to timing problems.
WARNING:Xst:737 - Found 1-bit latch for signal <PA_X3<135>>. Latches may be generated from incomplete case or if statements. We do not recommend the use of latches in FPGA/CPLD designs, as they may lead to timing problems.
WARNING:Xst:737 - Found 1-bit latch for signal <PA_X3<134>>. Latches may be generated from incomplete case or if statements. We do not recommend the use of latches in FPGA/CPLD designs, as they may lead to timing problems.
WARNING:Xst:737 - Found 1-bit latch for signal <PA_X3<133>>. Latches may be generated from incomplete case or if statements. We do not recommend the use of latches in FPGA/CPLD designs, as they may lead to timing problems.
WARNING:Xst:737 - Found 1-bit latch for signal <PA_X3<132>>. Latches may be generated from incomplete case or if statements. We do not recommend the use of latches in FPGA/CPLD designs, as they may lead to timing problems.
WARNING:Xst:737 - Found 1-bit latch for signal <PA_X3<131>>. Latches may be generated from incomplete case or if statements. We do not recommend the use of latches in FPGA/CPLD designs, as they may lead to timing problems.
WARNING:Xst:737 - Found 1-bit latch for signal <PA_X3<130>>. Latches may be generated from incomplete case or if statements. We do not recommend the use of latches in FPGA/CPLD designs, as they may lead to timing problems.
WARNING:Xst:737 - Found 1-bit latch for signal <PA_X3<129>>. Latches may be generated from incomplete case or if statements. We do not recommend the use of latches in FPGA/CPLD designs, as they may lead to timing problems.
WARNING:Xst:737 - Found 1-bit latch for signal <PA_X3<128>>. Latches may be generated from incomplete case or if statements. We do not recommend the use of latches in FPGA/CPLD designs, as they may lead to timing problems.
WARNING:Xst:737 - Found 1-bit latch for signal <PA_X3<127>>. Latches may be generated from incomplete case or if statements. We do not recommend the use of latches in FPGA/CPLD designs, as they may lead to timing problems.
WARNING:Xst:737 - Found 1-bit latch for signal <PA_X3<126>>. Latches may be generated from incomplete case or if statements. We do not recommend the use of latches in FPGA/CPLD designs, as they may lead to timing problems.
WARNING:Xst:737 - Found 1-bit latch for signal <PA_X3<125>>. Latches may be generated from incomplete case or if statements. We do not recommend the use of latches in FPGA/CPLD designs, as they may lead to timing problems.
WARNING:Xst:737 - Found 1-bit latch for signal <PA_X3<124>>. Latches may be generated from incomplete case or if statements. We do not recommend the use of latches in FPGA/CPLD designs, as they may lead to timing problems.
WARNING:Xst:737 - Found 1-bit latch for signal <PA_X3<123>>. Latches may be generated from incomplete case or if statements. We do not recommend the use of latches in FPGA/CPLD designs, as they may lead to timing problems.
WARNING:Xst:737 - Found 1-bit latch for signal <PA_X3<122>>. Latches may be generated from incomplete case or if statements. We do not recommend the use of latches in FPGA/CPLD designs, as they may lead to timing problems.
WARNING:Xst:737 - Found 1-bit latch for signal <PA_X3<121>>. Latches may be generated from incomplete case or if statements. We do not recommend the use of latches in FPGA/CPLD designs, as they may lead to timing problems.
WARNING:Xst:737 - Found 1-bit latch for signal <PA_X3<120>>. Latches may be generated from incomplete case or if statements. We do not recommend the use of latches in FPGA/CPLD designs, as they may lead to timing problems.
WARNING:Xst:737 - Found 1-bit latch for signal <PA_X3<119>>. Latches may be generated from incomplete case or if statements. We do not recommend the use of latches in FPGA/CPLD designs, as they may lead to timing problems.
WARNING:Xst:737 - Found 1-bit latch for signal <PA_X3<118>>. Latches may be generated from

incomplete case or if statements. We do not recommend the use of latches in FPGA/CPLD designs, as they may lead to timing problems.
WARNING:Xst:737 - Found 1-bit latch for signal <PA_X3<117>>. Latches may be generated from incomplete case or if statements. We do not recommend the use of latches in FPGA/CPLD designs, as they may lead to timing problems.
WARNING:Xst:737 - Found 1-bit latch for signal <PA_X3<116>>. Latches may be generated from incomplete case or if statements. We do not recommend the use of latches in FPGA/CPLD designs, as they may lead to timing problems.
WARNING:Xst:737 - Found 1-bit latch for signal <PA_X3<115>>. Latches may be generated from incomplete case or if statements. We do not recommend the use of latches in FPGA/CPLD designs, as they may lead to timing problems.
WARNING:Xst:737 - Found 1-bit latch for signal <PA_X3<114>>. Latches may be generated from incomplete case or if statements. We do not recommend the use of latches in FPGA/CPLD designs, as they may lead to timing problems.
WARNING:Xst:737 - Found 1-bit latch for signal <PA_X3<113>>. Latches may be generated from incomplete case or if statements. We do not recommend the use of latches in FPGA/CPLD designs, as they may lead to timing problems.
WARNING:Xst:737 - Found 1-bit latch for signal <PA_X3<112>>. Latches may be generated from incomplete case or if statements. We do not recommend the use of latches in FPGA/CPLD designs, as they may lead to timing problems.
WARNING:Xst:737 - Found 1-bit latch for signal <PA_X3<111>>. Latches may be generated from incomplete case or if statements. We do not recommend the use of latches in FPGA/CPLD designs, as they may lead to timing problems.
WARNING:Xst:737 - Found 1-bit latch for signal <PA_X3<110>>. Latches may be generated from incomplete case or if statements. We do not recommend the use of latches in FPGA/CPLD designs, as they may lead to timing problems.
WARNING:Xst:737 - Found 1-bit latch for signal <PA_X3<109>>. Latches may be generated from incomplete case or if statements. We do not recommend the use of latches in FPGA/CPLD designs, as they may lead to timing problems.
WARNING:Xst:737 - Found 1-bit latch for signal <PA_X3<108>>. Latches may be generated from incomplete case or if statements. We do not recommend the use of latches in FPGA/CPLD designs, as they may lead to timing problems.
WARNING:Xst:737 - Found 1-bit latch for signal <PA_X3<107>>. Latches may be generated from incomplete case or if statements. We do not recommend the use of latches in FPGA/CPLD designs, as they may lead to timing problems.
WARNING:Xst:737 - Found 1-bit latch for signal <PA_X3<106>>. Latches may be generated from incomplete case or if statements. We do not recommend the use of latches in FPGA/CPLD designs, as they may lead to timing problems.
WARNING:Xst:737 - Found 1-bit latch for signal <PA_X3<105>>. Latches may be generated from incomplete case or if statements. We do not recommend the use of latches in FPGA/CPLD designs, as they may lead to timing problems.
WARNING:Xst:737 - Found 1-bit latch for signal <PA_X3<104>>. Latches may be generated from incomplete case or if statements. We do not recommend the use of latches in FPGA/CPLD designs, as they may lead to timing problems.
WARNING:Xst:737 - Found 1-bit latch for signal <PA_X3<103>>. Latches may be generated from incomplete case or if statements. We do not recommend the use of latches in FPGA/CPLD designs, as they may lead to timing problems.
WARNING:Xst:737 - Found 1-bit latch for signal <PA_X3<102>>. Latches may be generated from incomplete case or if statements. We do not recommend the use of latches in FPGA/CPLD designs, as they may lead to timing problems.
WARNING:Xst:737 - Found 1-bit latch for signal <PA_X3<101>>. Latches may be generated from incomplete case or if statements. We do not recommend the use of latches in FPGA/CPLD designs, as they may lead to timing problems.
WARNING:Xst:737 - Found 1-bit latch for signal <PA_X3<100>>. Latches may be generated from incomplete case or if statements. We do not recommend the use of latches in FPGA/CPLD designs, as they may lead to timing problems.
WARNING:Xst:737 - Found 1-bit latch for signal <PA_X3<99>>. Latches may be generated from incomplete case or if statements. We do not recommend the use of latches in FPGA/CPLD designs, as they may lead to timing problems.
WARNING:Xst:737 - Found 1-bit latch for signal <PA_X3<98>>. Latches may be generated from incomplete case or if statements. We do not recommend the use of latches in FPGA/CPLD designs, as they may lead to timing problems.
WARNING:Xst:737 - Found 1-bit latch for signal <PA_X3<97>>. Latches may be generated from incomplete case or if statements. We do not recommend the use of latches in FPGA/CPLD designs, as they may lead to timing problems.
WARNING:Xst:737 - Found 1-bit latch for signal <PA_X3<96>>. Latches may be generated from incomplete case or if statements. We do not recommend the use of latches in FPGA/CPLD designs, as they may lead to timing problems.
WARNING:Xst:737 - Found 1-bit latch for signal <PA_X3<95>>. Latches may be generated from incomplete case or if statements. We do not recommend the use of latches in FPGA/CPLD designs, as they may lead to timing problems.

```
WARNING:Xst:737 - Found 1-bit latch for signal <PA_X3<94>>. Latches may be generated from
incomplete case or if statements. We do not recommend the use of latches in FPGA/CPLD
designs, as they may lead to timing problems.
WARNING:Xst:737 - Found 1-bit latch for signal <PA_X3<93>>. Latches may be generated from
incomplete case or if statements. We do not recommend the use of latches in FPGA/CPLD
designs, as they may lead to timing problems.
WARNING:Xst:737 - Found 1-bit latch for signal <PA_X3<92>>. Latches may be generated from
incomplete case or if statements. We do not recommend the use of latches in FPGA/CPLD
designs, as they may lead to timing problems.
WARNING:Xst:737 - Found 1-bit latch for signal <PA_X3<91>>. Latches may be generated from
incomplete case or if statements. We do not recommend the use of latches in FPGA/CPLD
designs, as they may lead to timing problems.
WARNING:Xst:737 - Found 1-bit latch for signal <PA_X3<90>>. Latches may be generated from
incomplete case or if statements. We do not recommend the use of latches in FPGA/CPLD
designs, as they may lead to timing problems.
WARNING:Xst:737 - Found 1-bit latch for signal <PA_X3<89>>. Latches may be generated from
incomplete case or if statements. We do not recommend the use of latches in FPGA/CPLD
designs, as they may lead to timing problems.
WARNING:Xst:737 - Found 1-bit latch for signal <PA_X3<88>>. Latches may be generated from
incomplete case or if statements. We do not recommend the use of latches in FPGA/CPLD
designs, as they may lead to timing problems.
WARNING:Xst:737 - Found 1-bit latch for signal <PA_X3<87>>. Latches may be generated from
incomplete case or if statements. We do not recommend the use of latches in FPGA/CPLD
designs, as they may lead to timing problems.
WARNING:Xst:737 - Found 1-bit latch for signal <PA_X3<86>>. Latches may be generated from
incomplete case or if statements. We do not recommend the use of latches in FPGA/CPLD
designs, as they may lead to timing problems.
WARNING:Xst:737 - Found 1-bit latch for signal <PA_X3<85>>. Latches may be generated from
incomplete case or if statements. We do not recommend the use of latches in FPGA/CPLD
designs, as they may lead to timing problems.
WARNING:Xst:737 - Found 1-bit latch for signal <PA_X3<84>>. Latches may be generated from
incomplete case or if statements. We do not recommend the use of latches in FPGA/CPLD
designs, as they may lead to timing problems.
WARNING:Xst:737 - Found 1-bit latch for signal <PA_X3<83>>. Latches may be generated from
incomplete case or if statements. We do not recommend the use of latches in FPGA/CPLD
designs, as they may lead to timing problems.
WARNING:Xst:737 - Found 1-bit latch for signal <PA_X3<82>>. Latches may be generated from
incomplete case or if statements. We do not recommend the use of latches in FPGA/CPLD
designs, as they may lead to timing problems.
WARNING:Xst:737 - Found 1-bit latch for signal <PA_X3<81>>. Latches may be generated from
incomplete case or if statements. We do not recommend the use of latches in FPGA/CPLD
designs, as they may lead to timing problems.
WARNING:Xst:737 - Found 1-bit latch for signal <PA_X3<80>>. Latches may be generated from
incomplete case or if statements. We do not recommend the use of latches in FPGA/CPLD
designs, as they may lead to timing problems.
WARNING:Xst:737 - Found 1-bit latch for signal <PA_X3<79>>. Latches may be generated from
incomplete case or if statements. We do not recommend the use of latches in FPGA/CPLD
designs, as they may lead to timing problems.
WARNING:Xst:737 - Found 1-bit latch for signal <PA_X3<78>>. Latches may be generated from
incomplete case or if statements. We do not recommend the use of latches in FPGA/CPLD
designs, as they may lead to timing problems.
WARNING:Xst:737 - Found 1-bit latch for signal <PA_X3<77>>. Latches may be generated from
incomplete case or if statements. We do not recommend the use of latches in FPGA/CPLD
designs, as they may lead to timing problems.
WARNING:Xst:737 - Found 1-bit latch for signal <PA_X3<76>>. Latches may be generated from
incomplete case or if statements. We do not recommend the use of latches in FPGA/CPLD
designs, as they may lead to timing problems.
WARNING:Xst:737 - Found 1-bit latch for signal <PA_X3<75>>. Latches may be generated from
incomplete case or if statements. We do not recommend the use of latches in FPGA/CPLD
designs, as they may lead to timing problems.
WARNING:Xst:737 - Found 1-bit latch for signal <PA_X3<74>>. Latches may be generated from
incomplete case or if statements. We do not recommend the use of latches in FPGA/CPLD
designs, as they may lead to timing problems.
WARNING:Xst:737 - Found 1-bit latch for signal <PA_X3<73>>. Latches may be generated from
incomplete case or if statements. We do not recommend the use of latches in FPGA/CPLD
designs, as they may lead to timing problems.
WARNING:Xst:737 - Found 1-bit latch for signal <PA_X3<72>>. Latches may be generated from
incomplete case or if statements. We do not recommend the use of latches in FPGA/CPLD
designs, as they may lead to timing problems.
WARNING:Xst:737 - Found 1-bit latch for signal <PA_X3<71>>. Latches may be generated from
incomplete case or if statements. We do not recommend the use of latches in FPGA/CPLD
```

```
designs, as they may lead to timing problems.
WARNING:Xst:737 - Found 1-bit latch for signal <PA_X3<70>>. Latches may be generated from
incomplete case or if statements. We do not recommend the use of latches in FPGA/CPLD
designs, as they may lead to timing problems.
WARNING:Xst:737 - Found 1-bit latch for signal <PA_X3<69>>. Latches may be generated from
incomplete case or if statements. We do not recommend the use of latches in FPGA/CPLD
designs, as they may lead to timing problems.
WARNING:Xst:737 - Found 1-bit latch for signal <PA_X3<68>>. Latches may be generated from
incomplete case or if statements. We do not recommend the use of latches in FPGA/CPLD
designs, as they may lead to timing problems.
WARNING:Xst:737 - Found 1-bit latch for signal <PA_X3<67>>. Latches may be generated from
incomplete case or if statements. We do not recommend the use of latches in FPGA/CPLD
designs, as they may lead to timing problems.
WARNING:Xst:737 - Found 1-bit latch for signal <PA_X3<66>>. Latches may be generated from
incomplete case or if statements. We do not recommend the use of latches in FPGA/CPLD
designs, as they may lead to timing problems.
WARNING:Xst:737 - Found 1-bit latch for signal <PA_X3<65>>. Latches may be generated from
incomplete case or if statements. We do not recommend the use of latches in FPGA/CPLD
designs, as they may lead to timing problems.
WARNING:Xst:737 - Found 1-bit latch for signal <PA_X3<64>>. Latches may be generated from
incomplete case or if statements. We do not recommend the use of latches in FPGA/CPLD
designs, as they may lead to timing problems.
WARNING:Xst:737 - Found 1-bit latch for signal <PA_X3<63>>. Latches may be generated from
incomplete case or if statements. We do not recommend the use of latches in FPGA/CPLD
designs, as they may lead to timing problems.
WARNING:Xst:737 - Found 1-bit latch for signal <PA_X3<62>>. Latches may be generated from
incomplete case or if statements. We do not recommend the use of latches in FPGA/CPLD
designs, as they may lead to timing problems.
WARNING:Xst:737 - Found 1-bit latch for signal <PA_X3<61>>. Latches may be generated from
incomplete case or if statements. We do not recommend the use of latches in FPGA/CPLD
designs, as they may lead to timing problems.
WARNING:Xst:737 - Found 1-bit latch for signal <PA_X3<60>>. Latches may be generated from
incomplete case or if statements. We do not recommend the use of latches in FPGA/CPLD
designs, as they may lead to timing problems.
WARNING:Xst:737 - Found 1-bit latch for signal <PA_X3<59>>. Latches may be generated from
incomplete case or if statements. We do not recommend the use of latches in FPGA/CPLD
designs, as they may lead to timing problems.
WARNING:Xst:737 - Found 1-bit latch for signal <PA_X3<58>>. Latches may be generated from
incomplete case or if statements. We do not recommend the use of latches in FPGA/CPLD
designs, as they may lead to timing problems.
WARNING:Xst:737 - Found 1-bit latch for signal <PA_X3<57>>. Latches may be generated from
incomplete case or if statements. We do not recommend the use of latches in FPGA/CPLD
designs, as they may lead to timing problems.
WARNING:Xst:737 - Found 1-bit latch for signal <PA_X3<56>>. Latches may be generated from
incomplete case or if statements. We do not recommend the use of latches in FPGA/CPLD
designs, as they may lead to timing problems.
WARNING:Xst:737 - Found 1-bit latch for signal <PA_X3<55>>. Latches may be generated from
incomplete case or if statements. We do not recommend the use of latches in FPGA/CPLD
designs, as they may lead to timing problems.
WARNING:Xst:737 - Found 1-bit latch for signal <PA_X3<54>>. Latches may be generated from
incomplete case or if statements. We do not recommend the use of latches in FPGA/CPLD
designs, as they may lead to timing problems.
WARNING:Xst:737 - Found 1-bit latch for signal <PA_X3<53>>. Latches may be generated from
incomplete case or if statements. We do not recommend the use of latches in FPGA/CPLD
designs, as they may lead to timing problems.
WARNING:Xst:737 - Found 1-bit latch for signal <PA_X3<52>>. Latches may be generated from
incomplete case or if statements. We do not recommend the use of latches in FPGA/CPLD
designs, as they may lead to timing problems.
WARNING:Xst:737 - Found 1-bit latch for signal <PA_X3<51>>. Latches may be generated from
incomplete case or if statements. We do not recommend the use of latches in FPGA/CPLD
designs, as they may lead to timing problems.
WARNING:Xst:737 - Found 1-bit latch for signal <PA_X3<50>>. Latches may be generated from
incomplete case or if statements. We do not recommend the use of latches in FPGA/CPLD
designs, as they may lead to timing problems.
WARNING:Xst:737 - Found 1-bit latch for signal <PA_X3<49>>. Latches may be generated from
incomplete case or if statements. We do not recommend the use of latches in FPGA/CPLD
designs, as they may lead to timing problems.
WARNING:Xst:737 - Found 1-bit latch for signal <PA_X3<48>>. Latches may be generated from
incomplete case or if statements. We do not recommend the use of latches in FPGA/CPLD
designs, as they may lead to timing problems.
WARNING:Xst:737 - Found 1-bit latch for signal <PA_X3<47>>. Latches may be generated from
```

incomplete case or if statements. We do not recommend the use of latches in FPGA/CPLD
designs, as they may lead to timing problems.
WARNING:Xst:737 - Found 1-bit latch for signal <PA_X3<46>>. Latches may be generated from
incomplete case or if statements. We do not recommend the use of latches in FPGA/CPLD
designs, as they may lead to timing problems.
WARNING:Xst:737 - Found 1-bit latch for signal <PA_X3<45>>. Latches may be generated from
incomplete case or if statements. We do not recommend the use of latches in FPGA/CPLD
designs, as they may lead to timing problems.
WARNING:Xst:737 - Found 1-bit latch for signal <PA_X3<44>>. Latches may be generated from
incomplete case or if statements. We do not recommend the use of latches in FPGA/CPLD
designs, as they may lead to timing problems.
WARNING:Xst:737 - Found 1-bit latch for signal <PA_X3<43>>. Latches may be generated from
incomplete case or if statements. We do not recommend the use of latches in FPGA/CPLD
designs, as they may lead to timing problems.
WARNING:Xst:737 - Found 1-bit latch for signal <PA_X3<42>>. Latches may be generated from
incomplete case or if statements. We do not recommend the use of latches in FPGA/CPLD
designs, as they may lead to timing problems.
WARNING:Xst:737 - Found 1-bit latch for signal <PA_X3<41>>. Latches may be generated from
incomplete case or if statements. We do not recommend the use of latches in FPGA/CPLD
designs, as they may lead to timing problems.
WARNING:Xst:737 - Found 1-bit latch for signal <PA_X3<40>>. Latches may be generated from
incomplete case or if statements. We do not recommend the use of latches in FPGA/CPLD
designs, as they may lead to timing problems.
WARNING:Xst:737 - Found 1-bit latch for signal <PA_X3<39>>. Latches may be generated from
incomplete case or if statements. We do not recommend the use of latches in FPGA/CPLD
designs, as they may lead to timing problems.
WARNING:Xst:737 - Found 1-bit latch for signal <PA_X3<38>>. Latches may be generated from
incomplete case or if statements. We do not recommend the use of latches in FPGA/CPLD
designs, as they may lead to timing problems.
WARNING:Xst:737 - Found 1-bit latch for signal <PA_X3<37>>. Latches may be generated from
incomplete case or if statements. We do not recommend the use of latches in FPGA/CPLD
designs, as they may lead to timing problems.
WARNING:Xst:737 - Found 1-bit latch for signal <PA_X3<36>>. Latches may be generated from
incomplete case or if statements. We do not recommend the use of latches in FPGA/CPLD
designs, as they may lead to timing problems.
WARNING:Xst:737 - Found 1-bit latch for signal <PA_X3<35>>. Latches may be generated from
incomplete case or if statements. We do not recommend the use of latches in FPGA/CPLD
designs, as they may lead to timing problems.
WARNING:Xst:737 - Found 1-bit latch for signal <PA_X3<34>>. Latches may be generated from
incomplete case or if statements. We do not recommend the use of latches in FPGA/CPLD
designs, as they may lead to timing problems.
WARNING:Xst:737 - Found 1-bit latch for signal <PA_X3<33>>. Latches may be generated from
incomplete case or if statements. We do not recommend the use of latches in FPGA/CPLD
designs, as they may lead to timing problems.
WARNING:Xst:737 - Found 1-bit latch for signal <PA_X3<32>>. Latches may be generated from
incomplete case or if statements. We do not recommend the use of latches in FPGA/CPLD
designs, as they may lead to timing problems.
WARNING:Xst:737 - Found 1-bit latch for signal <PA_X3<31>>. Latches may be generated from
incomplete case or if statements. We do not recommend the use of latches in FPGA/CPLD
designs, as they may lead to timing problems.
WARNING:Xst:737 - Found 1-bit latch for signal <PA_X3<30>>. Latches may be generated from
incomplete case or if statements. We do not recommend the use of latches in FPGA/CPLD
designs, as they may lead to timing problems.
WARNING:Xst:737 - Found 1-bit latch for signal <PA_X3<29>>. Latches may be generated from
incomplete case or if statements. We do not recommend the use of latches in FPGA/CPLD
designs, as they may lead to timing problems.
WARNING:Xst:737 - Found 1-bit latch for signal <PA_X3<28>>. Latches may be generated from
incomplete case or if statements. We do not recommend the use of latches in FPGA/CPLD
designs, as they may lead to timing problems.
WARNING:Xst:737 - Found 1-bit latch for signal <PA_X3<27>>. Latches may be generated from
incomplete case or if statements. We do not recommend the use of latches in FPGA/CPLD
designs, as they may lead to timing problems.
WARNING:Xst:737 - Found 1-bit latch for signal <PA_X3<26>>. Latches may be generated from
incomplete case or if statements. We do not recommend the use of latches in FPGA/CPLD
designs, as they may lead to timing problems.
WARNING:Xst:737 - Found 1-bit latch for signal <PA_X3<25>>. Latches may be generated from
incomplete case or if statements. We do not recommend the use of latches in FPGA/CPLD
designs, as they may lead to timing problems.
WARNING:Xst:737 - Found 1-bit latch for signal <PA_X3<24>>. Latches may be generated from
incomplete case or if statements. We do not recommend the use of latches in FPGA/CPLD
designs, as they may lead to timing problems.

```
WARNING:Xst:737 - Found 1-bit latch for signal <PA_X3<23>>. Latches may be generated from
incomplete case or if statements. We do not recommend the use of latches in FPGA/CPLD
designs, as they may lead to timing problems.
WARNING:Xst:737 - Found 1-bit latch for signal <PA_X3<22>>. Latches may be generated from
incomplete case or if statements. We do not recommend the use of latches in FPGA/CPLD
designs, as they may lead to timing problems.
WARNING:Xst:737 - Found 1-bit latch for signal <PA_X3<21>>. Latches may be generated from
incomplete case or if statements. We do not recommend the use of latches in FPGA/CPLD
designs, as they may lead to timing problems.
WARNING:Xst:737 - Found 1-bit latch for signal <PA_X3<20>>. Latches may be generated from
incomplete case or if statements. We do not recommend the use of latches in FPGA/CPLD
designs, as they may lead to timing problems.
WARNING:Xst:737 - Found 1-bit latch for signal <PA_X3<19>>. Latches may be generated from
incomplete case or if statements. We do not recommend the use of latches in FPGA/CPLD
designs, as they may lead to timing problems.
WARNING:Xst:737 - Found 1-bit latch for signal <PA_X3<18>>. Latches may be generated from
incomplete case or if statements. We do not recommend the use of latches in FPGA/CPLD
designs, as they may lead to timing problems.
WARNING:Xst:737 - Found 1-bit latch for signal <PA_X3<17>>. Latches may be generated from
incomplete case or if statements. We do not recommend the use of latches in FPGA/CPLD
designs, as they may lead to timing problems.
WARNING:Xst:737 - Found 1-bit latch for signal <PA_X3<16>>. Latches may be generated from
incomplete case or if statements. We do not recommend the use of latches in FPGA/CPLD
designs, as they may lead to timing problems.
WARNING:Xst:737 - Found 1-bit latch for signal <PA_X3<15>>. Latches may be generated from
incomplete case or if statements. We do not recommend the use of latches in FPGA/CPLD
designs, as they may lead to timing problems.
WARNING:Xst:737 - Found 1-bit latch for signal <PA_X3<14>>. Latches may be generated from
incomplete case or if statements. We do not recommend the use of latches in FPGA/CPLD
designs, as they may lead to timing problems.
WARNING:Xst:737 - Found 1-bit latch for signal <PA_X3<13>>. Latches may be generated from
incomplete case or if statements. We do not recommend the use of latches in FPGA/CPLD
designs, as they may lead to timing problems.
WARNING:Xst:737 - Found 1-bit latch for signal <PA_X3<12>>. Latches may be generated from
incomplete case or if statements. We do not recommend the use of latches in FPGA/CPLD
designs, as they may lead to timing problems.
WARNING:Xst:737 - Found 1-bit latch for signal <PA_X3<11>>. Latches may be generated from
incomplete case or if statements. We do not recommend the use of latches in FPGA/CPLD
designs, as they may lead to timing problems.
WARNING:Xst:737 - Found 1-bit latch for signal <PA_X3<10>>. Latches may be generated from
incomplete case or if statements. We do not recommend the use of latches in FPGA/CPLD
designs, as they may lead to timing problems.
WARNING:Xst:737 - Found 1-bit latch for signal <PA_X3<9>>. Latches may be generated from
incomplete case or if statements. We do not recommend the use of latches in FPGA/CPLD
designs, as they may lead to timing problems.
WARNING:Xst:737 - Found 1-bit latch for signal <PA_X3<8>>. Latches may be generated from
incomplete case or if statements. We do not recommend the use of latches in FPGA/CPLD
designs, as they may lead to timing problems.
WARNING:Xst:737 - Found 1-bit latch for signal <PA_X3<7>>. Latches may be generated from
incomplete case or if statements. We do not recommend the use of latches in FPGA/CPLD
designs, as they may lead to timing problems.
WARNING:Xst:737 - Found 1-bit latch for signal <PA_X3<6>>. Latches may be generated from
incomplete case or if statements. We do not recommend the use of latches in FPGA/CPLD
designs, as they may lead to timing problems.
WARNING:Xst:737 - Found 1-bit latch for signal <PA_X3<5>>. Latches may be generated from
incomplete case or if statements. We do not recommend the use of latches in FPGA/CPLD
designs, as they may lead to timing problems.
WARNING:Xst:737 - Found 1-bit latch for signal <PA_X3<4>>. Latches may be generated from
incomplete case or if statements. We do not recommend the use of latches in FPGA/CPLD
designs, as they may lead to timing problems.
WARNING:Xst:737 - Found 1-bit latch for signal <PA_X3<3>>. Latches may be generated from
incomplete case or if statements. We do not recommend the use of latches in FPGA/CPLD
designs, as they may lead to timing problems.
WARNING:Xst:737 - Found 1-bit latch for signal <PA_X3<2>>. Latches may be generated from
incomplete case or if statements. We do not recommend the use of latches in FPGA/CPLD
designs, as they may lead to timing problems.
WARNING:Xst:737 - Found 1-bit latch for signal <PA_X3<1>>. Latches may be generated from
incomplete case or if statements. We do not recommend the use of latches in FPGA/CPLD
designs, as they may lead to timing problems.
WARNING:Xst:737 - Found 1-bit latch for signal <PA_X3<0>>. Latches may be generated from
incomplete case or if statements. We do not recommend the use of latches in FPGA/CPLD
```

```
designs, as they may lead to timing problems.
WARNING:Xst:737 - Found 1-bit latch for signal <PA_Y3<162>>. Latches may be generated from
incomplete case or if statements. We do not recommend the use of latches in FPGA/CPLD
designs, as they may lead to timing problems.
WARNING:Xst:737 - Found 1-bit latch for signal <PA_Y3<161>>. Latches may be generated from
incomplete case or if statements. We do not recommend the use of latches in FPGA/CPLD
designs, as they may lead to timing problems.
WARNING:Xst:737 - Found 1-bit latch for signal <PA_Y3<160>>. Latches may be generated from
incomplete case or if statements. We do not recommend the use of latches in FPGA/CPLD
designs, as they may lead to timing problems.
WARNING:Xst:737 - Found 1-bit latch for signal <PA_Y3<159>>. Latches may be generated from
incomplete case or if statements. We do not recommend the use of latches in FPGA/CPLD
designs, as they may lead to timing problems.
WARNING:Xst:737 - Found 1-bit latch for signal <PA_Y3<158>>. Latches may be generated from
incomplete case or if statements. We do not recommend the use of latches in FPGA/CPLD
designs, as they may lead to timing problems.
WARNING:Xst:737 - Found 1-bit latch for signal <PA_Y3<157>>. Latches may be generated from
incomplete case or if statements. We do not recommend the use of latches in FPGA/CPLD
designs, as they may lead to timing problems.
WARNING:Xst:737 - Found 1-bit latch for signal <PA_Y3<156>>. Latches may be generated from
incomplete case or if statements. We do not recommend the use of latches in FPGA/CPLD
designs, as they may lead to timing problems.
WARNING:Xst:737 - Found 1-bit latch for signal <PA_Y3<155>>. Latches may be generated from
incomplete case or if statements. We do not recommend the use of latches in FPGA/CPLD
designs, as they may lead to timing problems.
WARNING:Xst:737 - Found 1-bit latch for signal <PA_Y3<154>>. Latches may be generated from
incomplete case or if statements. We do not recommend the use of latches in FPGA/CPLD
designs, as they may lead to timing problems.
WARNING:Xst:737 - Found 1-bit latch for signal <PA_Y3<153>>. Latches may be generated from
incomplete case or if statements. We do not recommend the use of latches in FPGA/CPLD
designs, as they may lead to timing problems.
WARNING:Xst:737 - Found 1-bit latch for signal <PA_Y3<152>>. Latches may be generated from
incomplete case or if statements. We do not recommend the use of latches in FPGA/CPLD
designs, as they may lead to timing problems.
WARNING:Xst:737 - Found 1-bit latch for signal <PA_Y3<151>>. Latches may be generated from
incomplete case or if statements. We do not recommend the use of latches in FPGA/CPLD
designs, as they may lead to timing problems.
WARNING:Xst:737 - Found 1-bit latch for signal <PA_Y3<150>>. Latches may be generated from
incomplete case or if statements. We do not recommend the use of latches in FPGA/CPLD
designs, as they may lead to timing problems.
WARNING:Xst:737 - Found 1-bit latch for signal <PA_Y3<149>>. Latches may be generated from
incomplete case or if statements. We do not recommend the use of latches in FPGA/CPLD
designs, as they may lead to timing problems.
WARNING:Xst:737 - Found 1-bit latch for signal <PA_Y3<148>>. Latches may be generated from
incomplete case or if statements. We do not recommend the use of latches in FPGA/CPLD
designs, as they may lead to timing problems.
WARNING:Xst:737 - Found 1-bit latch for signal <PA_Y3<147>>. Latches may be generated from
incomplete case or if statements. We do not recommend the use of latches in FPGA/CPLD
designs, as they may lead to timing problems.
WARNING:Xst:737 - Found 1-bit latch for signal <PA_Y3<146>>. Latches may be generated from
incomplete case or if statements. We do not recommend the use of latches in FPGA/CPLD
designs, as they may lead to timing problems.
WARNING:Xst:737 - Found 1-bit latch for signal <PA_Y3<145>>. Latches may be generated from
incomplete case or if statements. We do not recommend the use of latches in FPGA/CPLD
designs, as they may lead to timing problems.
WARNING:Xst:737 - Found 1-bit latch for signal <PA_Y3<144>>. Latches may be generated from
incomplete case or if statements. We do not recommend the use of latches in FPGA/CPLD
designs, as they may lead to timing problems.
WARNING:Xst:737 - Found 1-bit latch for signal <PA_Y3<143>>. Latches may be generated from
incomplete case or if statements. We do not recommend the use of latches in FPGA/CPLD
designs, as they may lead to timing problems.
WARNING:Xst:737 - Found 1-bit latch for signal <PA_Y3<142>>. Latches may be generated from
incomplete case or if statements. We do not recommend the use of latches in FPGA/CPLD
designs, as they may lead to timing problems.
WARNING:Xst:737 - Found 1-bit latch for signal <PA_Y3<141>>. Latches may be generated from
incomplete case or if statements. We do not recommend the use of latches in FPGA/CPLD
designs, as they may lead to timing problems.
WARNING:Xst:737 - Found 1-bit latch for signal <PA_Y3<140>>. Latches may be generated from
incomplete case or if statements. We do not recommend the use of latches in FPGA/CPLD
designs, as they may lead to timing problems.
WARNING:Xst:737 - Found 1-bit latch for signal <PA_Y3<139>>. Latches may be generated from
```

```
incomplete case or if statements. We do not recommend the use of latches in FPGA/CPLD
designs, as they may lead to timing problems.
WARNING:Xst:737 - Found 1-bit latch for signal <PA_Y3<138>>. Latches may be generated from
incomplete case or if statements. We do not recommend the use of latches in FPGA/CPLD
designs, as they may lead to timing problems.
WARNING:Xst:737 - Found 1-bit latch for signal <PA_Y3<137>>. Latches may be generated from
incomplete case or if statements. We do not recommend the use of latches in FPGA/CPLD
designs, as they may lead to timing problems.
WARNING:Xst:737 - Found 1-bit latch for signal <PA_Y3<136>>. Latches may be generated from
incomplete case or if statements. We do not recommend the use of latches in FPGA/CPLD
designs, as they may lead to timing problems.
WARNING:Xst:737 - Found 1-bit latch for signal <PA_Y3<135>>. Latches may be generated from
incomplete case or if statements. We do not recommend the use of latches in FPGA/CPLD
designs, as they may lead to timing problems.
WARNING:Xst:737 - Found 1-bit latch for signal <PA_Y3<134>>. Latches may be generated from
incomplete case or if statements. We do not recommend the use of latches in FPGA/CPLD
designs, as they may lead to timing problems.
WARNING:Xst:737 - Found 1-bit latch for signal <PA_Y3<133>>. Latches may be generated from
incomplete case or if statements. We do not recommend the use of latches in FPGA/CPLD
designs, as they may lead to timing problems.
WARNING:Xst:737 - Found 1-bit latch for signal <PA_Y3<132>>. Latches may be generated from
incomplete case or if statements. We do not recommend the use of latches in FPGA/CPLD
designs, as they may lead to timing problems.
WARNING:Xst:737 - Found 1-bit latch for signal <PA_Y3<131>>. Latches may be generated from
incomplete case or if statements. We do not recommend the use of latches in FPGA/CPLD
designs, as they may lead to timing problems.
WARNING:Xst:737 - Found 1-bit latch for signal <PA_Y3<130>>. Latches may be generated from
incomplete case or if statements. We do not recommend the use of latches in FPGA/CPLD
designs, as they may lead to timing problems.
WARNING:Xst:737 - Found 1-bit latch for signal <PA_Y3<129>>. Latches may be generated from
incomplete case or if statements. We do not recommend the use of latches in FPGA/CPLD
designs, as they may lead to timing problems.
WARNING:Xst:737 - Found 1-bit latch for signal <PA_Y3<128>>. Latches may be generated from
incomplete case or if statements. We do not recommend the use of latches in FPGA/CPLD
designs, as they may lead to timing problems.
WARNING:Xst:737 - Found 1-bit latch for signal <PA_Y3<127>>. Latches may be generated from
incomplete case or if statements. We do not recommend the use of latches in FPGA/CPLD
designs, as they may lead to timing problems.
WARNING:Xst:737 - Found 1-bit latch for signal <PA_Y3<126>>. Latches may be generated from
incomplete case or if statements. We do not recommend the use of latches in FPGA/CPLD
designs, as they may lead to timing problems.
WARNING:Xst:737 - Found 1-bit latch for signal <PA_Y3<125>>. Latches may be generated from
incomplete case or if statements. We do not recommend the use of latches in FPGA/CPLD
designs, as they may lead to timing problems.
WARNING:Xst:737 - Found 1-bit latch for signal <PA_Y3<124>>. Latches may be generated from
incomplete case or if statements. We do not recommend the use of latches in FPGA/CPLD
designs, as they may lead to timing problems.
WARNING:Xst:737 - Found 1-bit latch for signal <PA_Y3<123>>. Latches may be generated from
incomplete case or if statements. We do not recommend the use of latches in FPGA/CPLD
designs, as they may lead to timing problems.
WARNING:Xst:737 - Found 1-bit latch for signal <PA_Y3<122>>. Latches may be generated from
incomplete case or if statements. We do not recommend the use of latches in FPGA/CPLD
designs, as they may lead to timing problems.
WARNING:Xst:737 - Found 1-bit latch for signal <PA_Y3<121>>. Latches may be generated from
incomplete case or if statements. We do not recommend the use of latches in FPGA/CPLD
designs, as they may lead to timing problems.
WARNING:Xst:737 - Found 1-bit latch for signal <PA_Y3<120>>. Latches may be generated from
incomplete case or if statements. We do not recommend the use of latches in FPGA/CPLD
designs, as they may lead to timing problems.
WARNING:Xst:737 - Found 1-bit latch for signal <PA_Y3<119>>. Latches may be generated from
incomplete case or if statements. We do not recommend the use of latches in FPGA/CPLD
designs, as they may lead to timing problems.
WARNING:Xst:737 - Found 1-bit latch for signal <PA_Y3<118>>. Latches may be generated from
incomplete case or if statements. We do not recommend the use of latches in FPGA/CPLD
designs, as they may lead to timing problems.
WARNING:Xst:737 - Found 1-bit latch for signal <PA_Y3<117>>. Latches may be generated from
incomplete case or if statements. We do not recommend the use of latches in FPGA/CPLD
designs, as they may lead to timing problems.
WARNING:Xst:737 - Found 1-bit latch for signal <PA_Y3<116>>. Latches may be generated from
incomplete case or if statements. We do not recommend the use of latches in FPGA/CPLD
designs, as they may lead to timing problems.
```

```
WARNING:Xst:737 - Found 1-bit latch for signal <PA_Y3<115>>. Latches may be generated from
incomplete case or if statements. We do not recommend the use of latches in FPGA/CPLD
designs, as they may lead to timing problems.
WARNING:Xst:737 - Found 1-bit latch for signal <PA_Y3<114>>. Latches may be generated from
incomplete case or if statements. We do not recommend the use of latches in FPGA/CPLD
designs, as they may lead to timing problems.
WARNING:Xst:737 - Found 1-bit latch for signal <PA_Y3<113>>. Latches may be generated from
incomplete case or if statements. We do not recommend the use of latches in FPGA/CPLD
designs, as they may lead to timing problems.
WARNING:Xst:737 - Found 1-bit latch for signal <PA_Y3<112>>. Latches may be generated from
incomplete case or if statements. We do not recommend the use of latches in FPGA/CPLD
designs, as they may lead to timing problems.
WARNING:Xst:737 - Found 1-bit latch for signal <PA_Y3<111>>. Latches may be generated from
incomplete case or if statements. We do not recommend the use of latches in FPGA/CPLD
designs, as they may lead to timing problems.
WARNING:Xst:737 - Found 1-bit latch for signal <PA_Y3<110>>. Latches may be generated from
incomplete case or if statements. We do not recommend the use of latches in FPGA/CPLD
designs, as they may lead to timing problems.
WARNING:Xst:737 - Found 1-bit latch for signal <PA_Y3<109>>. Latches may be generated from
incomplete case or if statements. We do not recommend the use of latches in FPGA/CPLD
designs, as they may lead to timing problems.
WARNING:Xst:737 - Found 1-bit latch for signal <PA_Y3<108>>. Latches may be generated from
incomplete case or if statements. We do not recommend the use of latches in FPGA/CPLD
designs, as they may lead to timing problems.
WARNING:Xst:737 - Found 1-bit latch for signal <PA_Y3<107>>. Latches may be generated from
incomplete case or if statements. We do not recommend the use of latches in FPGA/CPLD
designs, as they may lead to timing problems.
WARNING:Xst:737 - Found 1-bit latch for signal <PA_Y3<106>>. Latches may be generated from
incomplete case or if statements. We do not recommend the use of latches in FPGA/CPLD
designs, as they may lead to timing problems.
WARNING:Xst:737 - Found 1-bit latch for signal <PA_Y3<105>>. Latches may be generated from
incomplete case or if statements. We do not recommend the use of latches in FPGA/CPLD
designs, as they may lead to timing problems.
WARNING:Xst:737 - Found 1-bit latch for signal <PA_Y3<104>>. Latches may be generated from
incomplete case or if statements. We do not recommend the use of latches in FPGA/CPLD
designs, as they may lead to timing problems.
WARNING:Xst:737 - Found 1-bit latch for signal <PA_Y3<103>>. Latches may be generated from
incomplete case or if statements. We do not recommend the use of latches in FPGA/CPLD
designs, as they may lead to timing problems.
WARNING:Xst:737 - Found 1-bit latch for signal <PA_Y3<102>>. Latches may be generated from
incomplete case or if statements. We do not recommend the use of latches in FPGA/CPLD
designs, as they may lead to timing problems.
WARNING:Xst:737 - Found 1-bit latch for signal <PA_Y3<101>>. Latches may be generated from
incomplete case or if statements. We do not recommend the use of latches in FPGA/CPLD
designs, as they may lead to timing problems.
WARNING:Xst:737 - Found 1-bit latch for signal <PA_Y3<100>>. Latches may be generated from
incomplete case or if statements. We do not recommend the use of latches in FPGA/CPLD
designs, as they may lead to timing problems.
WARNING:Xst:737 - Found 1-bit latch for signal <PA_Y3<99>>. Latches may be generated from
incomplete case or if statements. We do not recommend the use of latches in FPGA/CPLD
designs, as they may lead to timing problems.
WARNING:Xst:737 - Found 1-bit latch for signal <PA_Y3<98>>. Latches may be generated from
incomplete case or if statements. We do not recommend the use of latches in FPGA/CPLD
designs, as they may lead to timing problems.
WARNING:Xst:737 - Found 1-bit latch for signal <PA_Y3<97>>. Latches may be generated from
incomplete case or if statements. We do not recommend the use of latches in FPGA/CPLD
designs, as they may lead to timing problems.
WARNING:Xst:737 - Found 1-bit latch for signal <PA_Y3<96>>. Latches may be generated from
incomplete case or if statements. We do not recommend the use of latches in FPGA/CPLD
designs, as they may lead to timing problems.
WARNING:Xst:737 - Found 1-bit latch for signal <PA_Y3<95>>. Latches may be generated from
incomplete case or if statements. We do not recommend the use of latches in FPGA/CPLD
designs, as they may lead to timing problems.
WARNING:Xst:737 - Found 1-bit latch for signal <PA_Y3<94>>. Latches may be generated from
incomplete case or if statements. We do not recommend the use of latches in FPGA/CPLD
designs, as they may lead to timing problems.
WARNING:Xst:737 - Found 1-bit latch for signal <PA_Y3<93>>. Latches may be generated from
incomplete case or if statements. We do not recommend the use of latches in FPGA/CPLD
designs, as they may lead to timing problems.
WARNING:Xst:737 - Found 1-bit latch for signal <PA_Y3<92>>. Latches may be generated from
incomplete case or if statements. We do not recommend the use of latches in FPGA/CPLD
```

designs, as they may lead to timing problems.
WARNING:Xst:737 - Found 1-bit latch for signal <PA_Y3<91>>. Latches may be generated from incomplete case or if statements. We do not recommend the use of latches in FPGA/CPLD designs, as they may lead to timing problems.
WARNING:Xst:737 - Found 1-bit latch for signal <PA_Y3<90>>. Latches may be generated from incomplete case or if statements. We do not recommend the use of latches in FPGA/CPLD designs, as they may lead to timing problems.
WARNING:Xst:737 - Found 1-bit latch for signal <PA_Y3<89>>. Latches may be generated from incomplete case or if statements. We do not recommend the use of latches in FPGA/CPLD designs, as they may lead to timing problems.
WARNING:Xst:737 - Found 1-bit latch for signal <PA_Y3<88>>. Latches may be generated from incomplete case or if statements. We do not recommend the use of latches in FPGA/CPLD designs, as they may lead to timing problems.
WARNING:Xst:737 - Found 1-bit latch for signal <PA_Y3<87>>. Latches may be generated from incomplete case or if statements. We do not recommend the use of latches in FPGA/CPLD designs, as they may lead to timing problems.
WARNING:Xst:737 - Found 1-bit latch for signal <PA_Y3<86>>. Latches may be generated from incomplete case or if statements. We do not recommend the use of latches in FPGA/CPLD designs, as they may lead to timing problems.
WARNING:Xst:737 - Found 1-bit latch for signal <PA_Y3<85>>. Latches may be generated from incomplete case or if statements. We do not recommend the use of latches in FPGA/CPLD designs, as they may lead to timing problems.
WARNING:Xst:737 - Found 1-bit latch for signal <PA_Y3<84>>. Latches may be generated from incomplete case or if statements. We do not recommend the use of latches in FPGA/CPLD designs, as they may lead to timing problems.
WARNING:Xst:737 - Found 1-bit latch for signal <PA_Y3<83>>. Latches may be generated from incomplete case or if statements. We do not recommend the use of latches in FPGA/CPLD designs, as they may lead to timing problems.
WARNING:Xst:737 - Found 1-bit latch for signal <PA_Y3<82>>. Latches may be generated from incomplete case or if statements. We do not recommend the use of latches in FPGA/CPLD designs, as they may lead to timing problems.
WARNING:Xst:737 - Found 1-bit latch for signal <PA_Y3<81>>. Latches may be generated from incomplete case or if statements. We do not recommend the use of latches in FPGA/CPLD designs, as they may lead to timing problems.
WARNING:Xst:737 - Found 1-bit latch for signal <PA_Y3<80>>. Latches may be generated from incomplete case or if statements. We do not recommend the use of latches in FPGA/CPLD designs, as they may lead to timing problems.
WARNING:Xst:737 - Found 1-bit latch for signal <PA_Y3<79>>. Latches may be generated from incomplete case or if statements. We do not recommend the use of latches in FPGA/CPLD designs, as they may lead to timing problems.
WARNING:Xst:737 - Found 1-bit latch for signal <PA_Y3<78>>. Latches may be generated from incomplete case or if statements. We do not recommend the use of latches in FPGA/CPLD designs, as they may lead to timing problems.
WARNING:Xst:737 - Found 1-bit latch for signal <PA_Y3<77>>. Latches may be generated from incomplete case or if statements. We do not recommend the use of latches in FPGA/CPLD designs, as they may lead to timing problems.
WARNING:Xst:737 - Found 1-bit latch for signal <PA_Y3<76>>. Latches may be generated from incomplete case or if statements. We do not recommend the use of latches in FPGA/CPLD designs, as they may lead to timing problems.
WARNING:Xst:737 - Found 1-bit latch for signal <PA_Y3<75>>. Latches may be generated from incomplete case or if statements. We do not recommend the use of latches in FPGA/CPLD designs, as they may lead to timing problems.
WARNING:Xst:737 - Found 1-bit latch for signal <PA_Y3<74>>. Latches may be generated from incomplete case or if statements. We do not recommend the use of latches in FPGA/CPLD designs, as they may lead to timing problems.
WARNING:Xst:737 - Found 1-bit latch for signal <PA_Y3<73>>. Latches may be generated from incomplete case or if statements. We do not recommend the use of latches in FPGA/CPLD designs, as they may lead to timing problems.
WARNING:Xst:737 - Found 1-bit latch for signal <PA_Y3<72>>. Latches may be generated from incomplete case or if statements. We do not recommend the use of latches in FPGA/CPLD designs, as they may lead to timing problems.
WARNING:Xst:737 - Found 1-bit latch for signal <PA_Y3<71>>. Latches may be generated from incomplete case or if statements. We do not recommend the use of latches in FPGA/CPLD designs, as they may lead to timing problems.
WARNING:Xst:737 - Found 1-bit latch for signal <PA_Y3<70>>. Latches may be generated from incomplete case or if statements. We do not recommend the use of latches in FPGA/CPLD designs, as they may lead to timing problems.
WARNING:Xst:737 - Found 1-bit latch for signal <PA_Y3<69>>. Latches may be generated from incomplete case or if statements. We do not recommend the use of latches in FPGA/CPLD designs, as they may lead to timing problems.
WARNING:Xst:737 - Found 1-bit latch for signal <PA_Y3<68>>. Latches may be generated from

```
incomplete case or if statements. We do not recommend the use of latches in FPGA/CPLD
designs, as they may lead to timing problems.
WARNING:Xst:737 - Found 1-bit latch for signal <PA_Y3<67>>. Latches may be generated from
incomplete case or if statements. We do not recommend the use of latches in FPGA/CPLD
designs, as they may lead to timing problems.
WARNING:Xst:737 - Found 1-bit latch for signal <PA_Y3<66>>. Latches may be generated from
incomplete case or if statements. We do not recommend the use of latches in FPGA/CPLD
designs, as they may lead to timing problems.
WARNING:Xst:737 - Found 1-bit latch for signal <PA_Y3<65>>. Latches may be generated from
incomplete case or if statements. We do not recommend the use of latches in FPGA/CPLD
designs, as they may lead to timing problems.
WARNING:Xst:737 - Found 1-bit latch for signal <PA_Y3<64>>. Latches may be generated from
incomplete case or if statements. We do not recommend the use of latches in FPGA/CPLD
designs, as they may lead to timing problems.
WARNING:Xst:737 - Found 1-bit latch for signal <PA_Y3<63>>. Latches may be generated from
incomplete case or if statements. We do not recommend the use of latches in FPGA/CPLD
designs, as they may lead to timing problems.
WARNING:Xst:737 - Found 1-bit latch for signal <PA_Y3<62>>. Latches may be generated from
incomplete case or if statements. We do not recommend the use of latches in FPGA/CPLD
designs, as they may lead to timing problems.
WARNING:Xst:737 - Found 1-bit latch for signal <PA_Y3<61>>. Latches may be generated from
incomplete case or if statements. We do not recommend the use of latches in FPGA/CPLD
designs, as they may lead to timing problems.
WARNING:Xst:737 - Found 1-bit latch for signal <PA_Y3<60>>. Latches may be generated from
incomplete case or if statements. We do not recommend the use of latches in FPGA/CPLD
designs, as they may lead to timing problems.
WARNING:Xst:737 - Found 1-bit latch for signal <PA_Y3<59>>. Latches may be generated from
incomplete case or if statements. We do not recommend the use of latches in FPGA/CPLD
designs, as they may lead to timing problems.
WARNING:Xst:737 - Found 1-bit latch for signal <PA_Y3<58>>. Latches may be generated from
incomplete case or if statements. We do not recommend the use of latches in FPGA/CPLD
designs, as they may lead to timing problems.
WARNING:Xst:737 - Found 1-bit latch for signal <PA_Y3<57>>. Latches may be generated from
incomplete case or if statements. We do not recommend the use of latches in FPGA/CPLD
designs, as they may lead to timing problems.
WARNING:Xst:737 - Found 1-bit latch for signal <PA_Y3<56>>. Latches may be generated from
incomplete case or if statements. We do not recommend the use of latches in FPGA/CPLD
designs, as they may lead to timing problems.
WARNING:Xst:737 - Found 1-bit latch for signal <PA_Y3<55>>. Latches may be generated from
incomplete case or if statements. We do not recommend the use of latches in FPGA/CPLD
designs, as they may lead to timing problems.
WARNING:Xst:737 - Found 1-bit latch for signal <PA_Y3<54>>. Latches may be generated from
incomplete case or if statements. We do not recommend the use of latches in FPGA/CPLD
designs, as they may lead to timing problems.
WARNING:Xst:737 - Found 1-bit latch for signal <PA_Y3<53>>. Latches may be generated from
incomplete case or if statements. We do not recommend the use of latches in FPGA/CPLD
designs, as they may lead to timing problems.
WARNING:Xst:737 - Found 1-bit latch for signal <PA_Y3<52>>. Latches may be generated from
incomplete case or if statements. We do not recommend the use of latches in FPGA/CPLD
designs, as they may lead to timing problems.
WARNING:Xst:737 - Found 1-bit latch for signal <PA_Y3<51>>. Latches may be generated from
incomplete case or if statements. We do not recommend the use of latches in FPGA/CPLD
designs, as they may lead to timing problems.
WARNING:Xst:737 - Found 1-bit latch for signal <PA_Y3<50>>. Latches may be generated from
incomplete case or if statements. We do not recommend the use of latches in FPGA/CPLD
designs, as they may lead to timing problems.
WARNING:Xst:737 - Found 1-bit latch for signal <PA_Y3<49>>. Latches may be generated from
incomplete case or if statements. We do not recommend the use of latches in FPGA/CPLD
designs, as they may lead to timing problems.
WARNING:Xst:737 - Found 1-bit latch for signal <PA_Y3<48>>. Latches may be generated from
incomplete case or if statements. We do not recommend the use of latches in FPGA/CPLD
designs, as they may lead to timing problems.
WARNING:Xst:737 - Found 1-bit latch for signal <PA_Y3<47>>. Latches may be generated from
incomplete case or if statements. We do not recommend the use of latches in FPGA/CPLD
designs, as they may lead to timing problems.
WARNING:Xst:737 - Found 1-bit latch for signal <PA_Y3<46>>. Latches may be generated from
incomplete case or if statements. We do not recommend the use of latches in FPGA/CPLD
designs, as they may lead to timing problems.
WARNING:Xst:737 - Found 1-bit latch for signal <PA_Y3<45>>. Latches may be generated from
incomplete case or if statements. We do not recommend the use of latches in FPGA/CPLD
designs, as they may lead to timing problems.
```

```
WARNING:Xst:737 - Found 1-bit latch for signal <PA_Y3<44>>. Latches may be generated from
incomplete case or if statements. We do not recommend the use of latches in FPGA/CPLD
designs, as they may lead to timing problems.
WARNING:Xst:737 - Found 1-bit latch for signal <PA_Y3<43>>. Latches may be generated from
incomplete case or if statements. We do not recommend the use of latches in FPGA/CPLD
designs, as they may lead to timing problems.
WARNING:Xst:737 - Found 1-bit latch for signal <PA_Y3<42>>. Latches may be generated from
incomplete case or if statements. We do not recommend the use of latches in FPGA/CPLD
designs, as they may lead to timing problems.
WARNING:Xst:737 - Found 1-bit latch for signal <PA_Y3<41>>. Latches may be generated from
incomplete case or if statements. We do not recommend the use of latches in FPGA/CPLD
designs, as they may lead to timing problems.
WARNING:Xst:737 - Found 1-bit latch for signal <PA_Y3<40>>. Latches may be generated from
incomplete case or if statements. We do not recommend the use of latches in FPGA/CPLD
designs, as they may lead to timing problems.
WARNING:Xst:737 - Found 1-bit latch for signal <PA_Y3<39>>. Latches may be generated from
incomplete case or if statements. We do not recommend the use of latches in FPGA/CPLD
designs, as they may lead to timing problems.
WARNING:Xst:737 - Found 1-bit latch for signal <PA_Y3<38>>. Latches may be generated from
incomplete case or if statements. We do not recommend the use of latches in FPGA/CPLD
designs, as they may lead to timing problems.
WARNING:Xst:737 - Found 1-bit latch for signal <PA_Y3<37>>. Latches may be generated from
incomplete case or if statements. We do not recommend the use of latches in FPGA/CPLD
designs, as they may lead to timing problems.
WARNING:Xst:737 - Found 1-bit latch for signal <PA_Y3<36>>. Latches may be generated from
incomplete case or if statements. We do not recommend the use of latches in FPGA/CPLD
designs, as they may lead to timing problems.
WARNING:Xst:737 - Found 1-bit latch for signal <PA_Y3<35>>. Latches may be generated from
incomplete case or if statements. We do not recommend the use of latches in FPGA/CPLD
designs, as they may lead to timing problems.
WARNING:Xst:737 - Found 1-bit latch for signal <PA_Y3<34>>. Latches may be generated from
incomplete case or if statements. We do not recommend the use of latches in FPGA/CPLD
designs, as they may lead to timing problems.
WARNING:Xst:737 - Found 1-bit latch for signal <PA_Y3<33>>. Latches may be generated from
incomplete case or if statements. We do not recommend the use of latches in FPGA/CPLD
designs, as they may lead to timing problems.
WARNING:Xst:737 - Found 1-bit latch for signal <PA_Y3<32>>. Latches may be generated from
incomplete case or if statements. We do not recommend the use of latches in FPGA/CPLD
designs, as they may lead to timing problems.
WARNING:Xst:737 - Found 1-bit latch for signal <PA_Y3<31>>. Latches may be generated from
incomplete case or if statements. We do not recommend the use of latches in FPGA/CPLD
designs, as they may lead to timing problems.
WARNING:Xst:737 - Found 1-bit latch for signal <PA_Y3<30>>. Latches may be generated from
incomplete case or if statements. We do not recommend the use of latches in FPGA/CPLD
designs, as they may lead to timing problems.
WARNING:Xst:737 - Found 1-bit latch for signal <PA_Y3<29>>. Latches may be generated from
incomplete case or if statements. We do not recommend the use of latches in FPGA/CPLD
designs, as they may lead to timing problems.
WARNING:Xst:737 - Found 1-bit latch for signal <PA_Y3<28>>. Latches may be generated from
incomplete case or if statements. We do not recommend the use of latches in FPGA/CPLD
designs, as they may lead to timing problems.
WARNING:Xst:737 - Found 1-bit latch for signal <PA_Y3<27>>. Latches may be generated from
incomplete case or if statements. We do not recommend the use of latches in FPGA/CPLD
designs, as they may lead to timing problems.
WARNING:Xst:737 - Found 1-bit latch for signal <PA_Y3<26>>. Latches may be generated from
incomplete case or if statements. We do not recommend the use of latches in FPGA/CPLD
designs, as they may lead to timing problems.
WARNING:Xst:737 - Found 1-bit latch for signal <PA_Y3<25>>. Latches may be generated from
incomplete case or if statements. We do not recommend the use of latches in FPGA/CPLD
designs, as they may lead to timing problems.
WARNING:Xst:737 - Found 1-bit latch for signal <PA_Y3<24>>. Latches may be generated from
incomplete case or if statements. We do not recommend the use of latches in FPGA/CPLD
designs, as they may lead to timing problems.
WARNING:Xst:737 - Found 1-bit latch for signal <PA_Y3<23>>. Latches may be generated from
incomplete case or if statements. We do not recommend the use of latches in FPGA/CPLD
designs, as they may lead to timing problems.
WARNING:Xst:737 - Found 1-bit latch for signal <PA_Y3<22>>. Latches may be generated from
incomplete case or if statements. We do not recommend the use of latches in FPGA/CPLD
designs, as they may lead to timing problems.
WARNING:Xst:737 - Found 1-bit latch for signal <PA_Y3<21>>. Latches may be generated from
incomplete case or if statements. We do not recommend the use of latches in FPGA/CPLD
```

```
designs, as they may lead to timing problems.
WARNING:Xst:737 - Found 1-bit latch for signal <PA_Y3<20>>. Latches may be generated from
incomplete case or if statements. We do not recommend the use of latches in FPGA/CPLD
designs, as they may lead to timing problems.
WARNING:Xst:737 - Found 1-bit latch for signal <PA_Y3<19>>. Latches may be generated from
incomplete case or if statements. We do not recommend the use of latches in FPGA/CPLD
designs, as they may lead to timing problems.
WARNING:Xst:737 - Found 1-bit latch for signal <PA_Y3<18>>. Latches may be generated from
incomplete case or if statements. We do not recommend the use of latches in FPGA/CPLD
designs, as they may lead to timing problems.
WARNING:Xst:737 - Found 1-bit latch for signal <PA_Y3<17>>. Latches may be generated from
incomplete case or if statements. We do not recommend the use of latches in FPGA/CPLD
designs, as they may lead to timing problems.
WARNING:Xst:737 - Found 1-bit latch for signal <PA_Y3<16>>. Latches may be generated from
incomplete case or if statements. We do not recommend the use of latches in FPGA/CPLD
designs, as they may lead to timing problems.
WARNING:Xst:737 - Found 1-bit latch for signal <PA_Y3<15>>. Latches may be generated from
incomplete case or if statements. We do not recommend the use of latches in FPGA/CPLD
designs, as they may lead to timing problems.
WARNING:Xst:737 - Found 1-bit latch for signal <PA_Y3<14>>. Latches may be generated from
incomplete case or if statements. We do not recommend the use of latches in FPGA/CPLD
designs, as they may lead to timing problems.
WARNING:Xst:737 - Found 1-bit latch for signal <PA_Y3<13>>. Latches may be generated from
incomplete case or if statements. We do not recommend the use of latches in FPGA/CPLD
designs, as they may lead to timing problems.
WARNING:Xst:737 - Found 1-bit latch for signal <PA_Y3<12>>. Latches may be generated from
incomplete case or if statements. We do not recommend the use of latches in FPGA/CPLD
designs, as they may lead to timing problems.
WARNING:Xst:737 - Found 1-bit latch for signal <PA_Y3<11>>. Latches may be generated from
incomplete case or if statements. We do not recommend the use of latches in FPGA/CPLD
designs, as they may lead to timing problems.
WARNING:Xst:737 - Found 1-bit latch for signal <PA_Y3<10>>. Latches may be generated from
incomplete case or if statements. We do not recommend the use of latches in FPGA/CPLD
designs, as they may lead to timing problems.
WARNING:Xst:737 - Found 1-bit latch for signal <PA_Y3<9>>. Latches may be generated from
incomplete case or if statements. We do not recommend the use of latches in FPGA/CPLD
designs, as they may lead to timing problems.
WARNING:Xst:737 - Found 1-bit latch for signal <PA_Y3<8>>. Latches may be generated from
incomplete case or if statements. We do not recommend the use of latches in FPGA/CPLD
designs, as they may lead to timing problems.
WARNING:Xst:737 - Found 1-bit latch for signal <PA_Y3<7>>. Latches may be generated from
incomplete case or if statements. We do not recommend the use of latches in FPGA/CPLD
designs, as they may lead to timing problems.
WARNING:Xst:737 - Found 1-bit latch for signal <PA_Y3<6>>. Latches may be generated from
incomplete case or if statements. We do not recommend the use of latches in FPGA/CPLD
designs, as they may lead to timing problems.
WARNING:Xst:737 - Found 1-bit latch for signal <PA_Y3<5>>. Latches may be generated from
incomplete case or if statements. We do not recommend the use of latches in FPGA/CPLD
designs, as they may lead to timing problems.
WARNING:Xst:737 - Found 1-bit latch for signal <PA_Y3<4>>. Latches may be generated from
incomplete case or if statements. We do not recommend the use of latches in FPGA/CPLD
designs, as they may lead to timing problems.
WARNING:Xst:737 - Found 1-bit latch for signal <PA_Y3<3>>. Latches may be generated from
incomplete case or if statements. We do not recommend the use of latches in FPGA/CPLD
designs, as they may lead to timing problems.
WARNING:Xst:737 - Found 1-bit latch for signal <PA_Y3<2>>. Latches may be generated from
incomplete case or if statements. We do not recommend the use of latches in FPGA/CPLD
designs, as they may lead to timing problems.
WARNING:Xst:737 - Found 1-bit latch for signal <PA_Y3<1>>. Latches may be generated from
incomplete case or if statements. We do not recommend the use of latches in FPGA/CPLD
designs, as they may lead to timing problems.
WARNING:Xst:737 - Found 1-bit latch for signal <PA_Y3<0>>. Latches may be generated from
incomplete case or if statements. We do not recommend the use of latches in FPGA/CPLD
designs, as they may lead to timing problems.
WARNING:Xst:737 - Found 1-bit latch for signal <PA_Z3<162>>. Latches may be generated from
incomplete case or if statements. We do not recommend the use of latches in FPGA/CPLD
designs, as they may lead to timing problems.
WARNING:Xst:737 - Found 1-bit latch for signal <PA_Z3<161>>. Latches may be generated from
incomplete case or if statements. We do not recommend the use of latches in FPGA/CPLD
designs, as they may lead to timing problems.
WARNING:Xst:737 - Found 1-bit latch for signal <PA_Z3<160>>. Latches may be generated from
```

```
incomplete case or if statements. We do not recommend the use of latches in FPGA/CPLD
designs, as they may lead to timing problems.
WARNING:Xst:737 - Found 1-bit latch for signal <PA_Z3<159>>. Latches may be generated from
incomplete case or if statements. We do not recommend the use of latches in FPGA/CPLD
designs, as they may lead to timing problems.
WARNING:Xst:737 - Found 1-bit latch for signal <PA_Z3<158>>. Latches may be generated from
incomplete case or if statements. We do not recommend the use of latches in FPGA/CPLD
designs, as they may lead to timing problems.
WARNING:Xst:737 - Found 1-bit latch for signal <PA_Z3<157>>. Latches may be generated from
incomplete case or if statements. We do not recommend the use of latches in FPGA/CPLD
designs, as they may lead to timing problems.
WARNING:Xst:737 - Found 1-bit latch for signal <PA_Z3<156>>. Latches may be generated from
incomplete case or if statements. We do not recommend the use of latches in FPGA/CPLD
designs, as they may lead to timing problems.
WARNING:Xst:737 - Found 1-bit latch for signal <PA_Z3<155>>. Latches may be generated from
incomplete case or if statements. We do not recommend the use of latches in FPGA/CPLD
designs, as they may lead to timing problems.
WARNING:Xst:737 - Found 1-bit latch for signal <PA_Z3<154>>. Latches may be generated from
incomplete case or if statements. We do not recommend the use of latches in FPGA/CPLD
designs, as they may lead to timing problems.
WARNING:Xst:737 - Found 1-bit latch for signal <PA_Z3<153>>. Latches may be generated from
incomplete case or if statements. We do not recommend the use of latches in FPGA/CPLD
designs, as they may lead to timing problems.
WARNING:Xst:737 - Found 1-bit latch for signal <PA_Z3<152>>. Latches may be generated from
incomplete case or if statements. We do not recommend the use of latches in FPGA/CPLD
designs, as they may lead to timing problems.
WARNING:Xst:737 - Found 1-bit latch for signal <PA_Z3<151>>. Latches may be generated from
incomplete case or if statements. We do not recommend the use of latches in FPGA/CPLD
designs, as they may lead to timing problems.
WARNING:Xst:737 - Found 1-bit latch for signal <PA_Z3<150>>. Latches may be generated from
incomplete case or if statements. We do not recommend the use of latches in FPGA/CPLD
designs, as they may lead to timing problems.
WARNING:Xst:737 - Found 1-bit latch for signal <PA_Z3<149>>. Latches may be generated from
incomplete case or if statements. We do not recommend the use of latches in FPGA/CPLD
designs, as they may lead to timing problems.
WARNING:Xst:737 - Found 1-bit latch for signal <PA_Z3<148>>. Latches may be generated from
incomplete case or if statements. We do not recommend the use of latches in FPGA/CPLD
designs, as they may lead to timing problems.
WARNING:Xst:737 - Found 1-bit latch for signal <PA_Z3<147>>. Latches may be generated from
incomplete case or if statements. We do not recommend the use of latches in FPGA/CPLD
designs, as they may lead to timing problems.
WARNING:Xst:737 - Found 1-bit latch for signal <PA_Z3<146>>. Latches may be generated from
incomplete case or if statements. We do not recommend the use of latches in FPGA/CPLD
designs, as they may lead to timing problems.
WARNING:Xst:737 - Found 1-bit latch for signal <PA_Z3<145>>. Latches may be generated from
incomplete case or if statements. We do not recommend the use of latches in FPGA/CPLD
designs, as they may lead to timing problems.
WARNING:Xst:737 - Found 1-bit latch for signal <PA_Z3<144>>. Latches may be generated from
incomplete case or if statements. We do not recommend the use of latches in FPGA/CPLD
designs, as they may lead to timing problems.
WARNING:Xst:737 - Found 1-bit latch for signal <PA_Z3<143>>. Latches may be generated from
incomplete case or if statements. We do not recommend the use of latches in FPGA/CPLD
designs, as they may lead to timing problems.
WARNING:Xst:737 - Found 1-bit latch for signal <PA_Z3<142>>. Latches may be generated from
incomplete case or if statements. We do not recommend the use of latches in FPGA/CPLD
designs, as they may lead to timing problems.
WARNING:Xst:737 - Found 1-bit latch for signal <PA_Z3<141>>. Latches may be generated from
incomplete case or if statements. We do not recommend the use of latches in FPGA/CPLD
designs, as they may lead to timing problems.
WARNING:Xst:737 - Found 1-bit latch for signal <PA_Z3<140>>. Latches may be generated from
incomplete case or if statements. We do not recommend the use of latches in FPGA/CPLD
designs, as they may lead to timing problems.
WARNING:Xst:737 - Found 1-bit latch for signal <PA_Z3<139>>. Latches may be generated from
incomplete case or if statements. We do not recommend the use of latches in FPGA/CPLD
designs, as they may lead to timing problems.
WARNING:Xst:737 - Found 1-bit latch for signal <PA_Z3<138>>. Latches may be generated from
incomplete case or if statements. We do not recommend the use of latches in FPGA/CPLD
designs, as they may lead to timing problems.
WARNING:Xst:737 - Found 1-bit latch for signal <PA_Z3<137>>. Latches may be generated from
incomplete case or if statements. We do not recommend the use of latches in FPGA/CPLD
designs, as they may lead to timing problems.
```

```
WARNING:Xst:737 - Found 1-bit latch for signal <PA_Z3<136>>. Latches may be generated from
incomplete case or if statements. We do not recommend the use of latches in FPGA/CPLD
designs, as they may lead to timing problems.
WARNING:Xst:737 - Found 1-bit latch for signal <PA_Z3<135>>. Latches may be generated from
incomplete case or if statements. We do not recommend the use of latches in FPGA/CPLD
designs, as they may lead to timing problems.
WARNING:Xst:737 - Found 1-bit latch for signal <PA_Z3<134>>. Latches may be generated from
incomplete case or if statements. We do not recommend the use of latches in FPGA/CPLD
designs, as they may lead to timing problems.
WARNING:Xst:737 - Found 1-bit latch for signal <PA_Z3<133>>. Latches may be generated from
incomplete case or if statements. We do not recommend the use of latches in FPGA/CPLD
designs, as they may lead to timing problems.
WARNING:Xst:737 - Found 1-bit latch for signal <PA_Z3<132>>. Latches may be generated from
incomplete case or if statements. We do not recommend the use of latches in FPGA/CPLD
designs, as they may lead to timing problems.
WARNING:Xst:737 - Found 1-bit latch for signal <PA_Z3<131>>. Latches may be generated from
incomplete case or if statements. We do not recommend the use of latches in FPGA/CPLD
designs, as they may lead to timing problems.
WARNING:Xst:737 - Found 1-bit latch for signal <PA_Z3<130>>. Latches may be generated from
incomplete case or if statements. We do not recommend the use of latches in FPGA/CPLD
designs, as they may lead to timing problems.
WARNING:Xst:737 - Found 1-bit latch for signal <PA_Z3<129>>. Latches may be generated from
incomplete case or if statements. We do not recommend the use of latches in FPGA/CPLD
designs, as they may lead to timing problems.
WARNING:Xst:737 - Found 1-bit latch for signal <PA_Z3<128>>. Latches may be generated from
incomplete case or if statements. We do not recommend the use of latches in FPGA/CPLD
designs, as they may lead to timing problems.
WARNING:Xst:737 - Found 1-bit latch for signal <PA_Z3<127>>. Latches may be generated from
incomplete case or if statements. We do not recommend the use of latches in FPGA/CPLD
designs, as they may lead to timing problems.
WARNING:Xst:737 - Found 1-bit latch for signal <PA_Z3<126>>. Latches may be generated from
incomplete case or if statements. We do not recommend the use of latches in FPGA/CPLD
designs, as they may lead to timing problems.
WARNING:Xst:737 - Found 1-bit latch for signal <PA_Z3<125>>. Latches may be generated from
incomplete case or if statements. We do not recommend the use of latches in FPGA/CPLD
designs, as they may lead to timing problems.
WARNING:Xst:737 - Found 1-bit latch for signal <PA_Z3<124>>. Latches may be generated from
incomplete case or if statements. We do not recommend the use of latches in FPGA/CPLD
designs, as they may lead to timing problems.
WARNING:Xst:737 - Found 1-bit latch for signal <PA_Z3<123>>. Latches may be generated from
incomplete case or if statements. We do not recommend the use of latches in FPGA/CPLD
designs, as they may lead to timing problems.
WARNING:Xst:737 - Found 1-bit latch for signal <PA_Z3<122>>. Latches may be generated from
incomplete case or if statements. We do not recommend the use of latches in FPGA/CPLD
designs, as they may lead to timing problems.
WARNING:Xst:737 - Found 1-bit latch for signal <PA_Z3<121>>. Latches may be generated from
incomplete case or if statements. We do not recommend the use of latches in FPGA/CPLD
designs, as they may lead to timing problems.
WARNING:Xst:737 - Found 1-bit latch for signal <PA_Z3<120>>. Latches may be generated from
incomplete case or if statements. We do not recommend the use of latches in FPGA/CPLD
designs, as they may lead to timing problems.
WARNING:Xst:737 - Found 1-bit latch for signal <PA_Z3<119>>. Latches may be generated from
incomplete case or if statements. We do not recommend the use of latches in FPGA/CPLD
designs, as they may lead to timing problems.
WARNING:Xst:737 - Found 1-bit latch for signal <PA_Z3<118>>. Latches may be generated from
incomplete case or if statements. We do not recommend the use of latches in FPGA/CPLD
designs, as they may lead to timing problems.
WARNING:Xst:737 - Found 1-bit latch for signal <PA_Z3<117>>. Latches may be generated from
incomplete case or if statements. We do not recommend the use of latches in FPGA/CPLD
designs, as they may lead to timing problems.
WARNING:Xst:737 - Found 1-bit latch for signal <PA_Z3<116>>. Latches may be generated from
incomplete case or if statements. We do not recommend the use of latches in FPGA/CPLD
designs, as they may lead to timing problems.
WARNING:Xst:737 - Found 1-bit latch for signal <PA_Z3<115>>. Latches may be generated from
incomplete case or if statements. We do not recommend the use of latches in FPGA/CPLD
designs, as they may lead to timing problems.
WARNING:Xst:737 - Found 1-bit latch for signal <PA_Z3<114>>. Latches may be generated from
incomplete case or if statements. We do not recommend the use of latches in FPGA/CPLD
designs, as they may lead to timing problems.
WARNING:Xst:737 - Found 1-bit latch for signal <PA_Z3<113>>. Latches may be generated from
incomplete case or if statements. We do not recommend the use of latches in FPGA/CPLD
```

```
designs, as they may lead to timing problems.
WARNING:Xst:737 - Found 1-bit latch for signal <PA_Z3<112>>. Latches may be generated from
incomplete case or if statements. We do not recommend the use of latches in FPGA/CPLD
designs, as they may lead to timing problems.
WARNING:Xst:737 - Found 1-bit latch for signal <PA_Z3<111>>. Latches may be generated from
incomplete case or if statements. We do not recommend the use of latches in FPGA/CPLD
designs, as they may lead to timing problems.
WARNING:Xst:737 - Found 1-bit latch for signal <PA_Z3<110>>. Latches may be generated from
incomplete case or if statements. We do not recommend the use of latches in FPGA/CPLD
designs, as they may lead to timing problems.
WARNING:Xst:737 - Found 1-bit latch for signal <PA_Z3<109>>. Latches may be generated from
incomplete case or if statements. We do not recommend the use of latches in FPGA/CPLD
designs, as they may lead to timing problems.
WARNING:Xst:737 - Found 1-bit latch for signal <PA_Z3<108>>. Latches may be generated from
incomplete case or if statements. We do not recommend the use of latches in FPGA/CPLD
designs, as they may lead to timing problems.
WARNING:Xst:737 - Found 1-bit latch for signal <PA_Z3<107>>. Latches may be generated from
incomplete case or if statements. We do not recommend the use of latches in FPGA/CPLD
designs, as they may lead to timing problems.
WARNING:Xst:737 - Found 1-bit latch for signal <PA_Z3<106>>. Latches may be generated from
incomplete case or if statements. We do not recommend the use of latches in FPGA/CPLD
designs, as they may lead to timing problems.
WARNING:Xst:737 - Found 1-bit latch for signal <PA_Z3<105>>. Latches may be generated from
incomplete case or if statements. We do not recommend the use of latches in FPGA/CPLD
designs, as they may lead to timing problems.
WARNING:Xst:737 - Found 1-bit latch for signal <PA_Z3<104>>. Latches may be generated from
incomplete case or if statements. We do not recommend the use of latches in FPGA/CPLD
designs, as they may lead to timing problems.
WARNING:Xst:737 - Found 1-bit latch for signal <PA_Z3<103>>. Latches may be generated from
incomplete case or if statements. We do not recommend the use of latches in FPGA/CPLD
designs, as they may lead to timing problems.
WARNING:Xst:737 - Found 1-bit latch for signal <PA_Z3<102>>. Latches may be generated from
incomplete case or if statements. We do not recommend the use of latches in FPGA/CPLD
designs, as they may lead to timing problems.
WARNING:Xst:737 - Found 1-bit latch for signal <PA_Z3<101>>. Latches may be generated from
incomplete case or if statements. We do not recommend the use of latches in FPGA/CPLD
designs, as they may lead to timing problems.
WARNING:Xst:737 - Found 1-bit latch for signal <PA_Z3<100>>. Latches may be generated from
incomplete case or if statements. We do not recommend the use of latches in FPGA/CPLD
designs, as they may lead to timing problems.
WARNING:Xst:737 - Found 1-bit latch for signal <PA_Z3<99>>. Latches may be generated from
incomplete case or if statements. We do not recommend the use of latches in FPGA/CPLD
designs, as they may lead to timing problems.
WARNING:Xst:737 - Found 1-bit latch for signal <PA_Z3<98>>. Latches may be generated from
incomplete case or if statements. We do not recommend the use of latches in FPGA/CPLD
designs, as they may lead to timing problems.
WARNING:Xst:737 - Found 1-bit latch for signal <PA_Z3<97>>. Latches may be generated from
incomplete case or if statements. We do not recommend the use of latches in FPGA/CPLD
designs, as they may lead to timing problems.
WARNING:Xst:737 - Found 1-bit latch for signal <PA_Z3<96>>. Latches may be generated from
incomplete case or if statements. We do not recommend the use of latches in FPGA/CPLD
designs, as they may lead to timing problems.
WARNING:Xst:737 - Found 1-bit latch for signal <PA_Z3<95>>. Latches may be generated from
incomplete case or if statements. We do not recommend the use of latches in FPGA/CPLD
designs, as they may lead to timing problems.
WARNING:Xst:737 - Found 1-bit latch for signal <PA_Z3<94>>. Latches may be generated from
incomplete case or if statements. We do not recommend the use of latches in FPGA/CPLD
designs, as they may lead to timing problems.
WARNING:Xst:737 - Found 1-bit latch for signal <PA_Z3<93>>. Latches may be generated from
incomplete case or if statements. We do not recommend the use of latches in FPGA/CPLD
designs, as they may lead to timing problems.
WARNING:Xst:737 - Found 1-bit latch for signal <PA_Z3<92>>. Latches may be generated from
incomplete case or if statements. We do not recommend the use of latches in FPGA/CPLD
designs, as they may lead to timing problems.
WARNING:Xst:737 - Found 1-bit latch for signal <PA_Z3<91>>. Latches may be generated from
incomplete case or if statements. We do not recommend the use of latches in FPGA/CPLD
designs, as they may lead to timing problems.
WARNING:Xst:737 - Found 1-bit latch for signal <PA_Z3<90>>. Latches may be generated from
incomplete case or if statements. We do not recommend the use of latches in FPGA/CPLD
designs, as they may lead to timing problems.
WARNING:Xst:737 - Found 1-bit latch for signal <PA_Z3<89>>. Latches may be generated from
```

```
incomplete case or if statements. We do not recommend the use of latches in FPGA/CPLD
designs, as they may lead to timing problems.
WARNING:Xst:737 - Found 1-bit latch for signal <PA_Z3<88>>. Latches may be generated from
incomplete case or if statements. We do not recommend the use of latches in FPGA/CPLD
designs, as they may lead to timing problems.
WARNING:Xst:737 - Found 1-bit latch for signal <PA_Z3<87>>. Latches may be generated from
incomplete case or if statements. We do not recommend the use of latches in FPGA/CPLD
designs, as they may lead to timing problems.
WARNING:Xst:737 - Found 1-bit latch for signal <PA_Z3<86>>. Latches may be generated from
incomplete case or if statements. We do not recommend the use of latches in FPGA/CPLD
designs, as they may lead to timing problems.
WARNING:Xst:737 - Found 1-bit latch for signal <PA_Z3<85>>. Latches may be generated from
incomplete case or if statements. We do not recommend the use of latches in FPGA/CPLD
designs, as they may lead to timing problems.
WARNING:Xst:737 - Found 1-bit latch for signal <PA_Z3<84>>. Latches may be generated from
incomplete case or if statements. We do not recommend the use of latches in FPGA/CPLD
designs, as they may lead to timing problems.
WARNING:Xst:737 - Found 1-bit latch for signal <PA_Z3<83>>. Latches may be generated from
incomplete case or if statements. We do not recommend the use of latches in FPGA/CPLD
designs, as they may lead to timing problems.
WARNING:Xst:737 - Found 1-bit latch for signal <PA_Z3<82>>. Latches may be generated from
incomplete case or if statements. We do not recommend the use of latches in FPGA/CPLD
designs, as they may lead to timing problems.
WARNING:Xst:737 - Found 1-bit latch for signal <PA_Z3<81>>. Latches may be generated from
incomplete case or if statements. We do not recommend the use of latches in FPGA/CPLD
designs, as they may lead to timing problems.
WARNING:Xst:737 - Found 1-bit latch for signal <PA_Z3<80>>. Latches may be generated from
incomplete case or if statements. We do not recommend the use of latches in FPGA/CPLD
designs, as they may lead to timing problems.
WARNING:Xst:737 - Found 1-bit latch for signal <PA_Z3<79>>. Latches may be generated from
incomplete case or if statements. We do not recommend the use of latches in FPGA/CPLD
designs, as they may lead to timing problems.
WARNING:Xst:737 - Found 1-bit latch for signal <PA_Z3<78>>. Latches may be generated from
incomplete case or if statements. We do not recommend the use of latches in FPGA/CPLD
designs, as they may lead to timing problems.
WARNING:Xst:737 - Found 1-bit latch for signal <PA_Z3<77>>. Latches may be generated from
incomplete case or if statements. We do not recommend the use of latches in FPGA/CPLD
designs, as they may lead to timing problems.
WARNING:Xst:737 - Found 1-bit latch for signal <PA_Z3<76>>. Latches may be generated from
incomplete case or if statements. We do not recommend the use of latches in FPGA/CPLD
designs, as they may lead to timing problems.
WARNING:Xst:737 - Found 1-bit latch for signal <PA_Z3<75>>. Latches may be generated from
incomplete case or if statements. We do not recommend the use of latches in FPGA/CPLD
designs, as they may lead to timing problems.
WARNING:Xst:737 - Found 1-bit latch for signal <PA_Z3<74>>. Latches may be generated from
incomplete case or if statements. We do not recommend the use of latches in FPGA/CPLD
designs, as they may lead to timing problems.
WARNING:Xst:737 - Found 1-bit latch for signal <PA_Z3<73>>. Latches may be generated from
incomplete case or if statements. We do not recommend the use of latches in FPGA/CPLD
designs, as they may lead to timing problems.
WARNING:Xst:737 - Found 1-bit latch for signal <PA_Z3<72>>. Latches may be generated from
incomplete case or if statements. We do not recommend the use of latches in FPGA/CPLD
designs, as they may lead to timing problems.
WARNING:Xst:737 - Found 1-bit latch for signal <PA_Z3<71>>. Latches may be generated from
incomplete case or if statements. We do not recommend the use of latches in FPGA/CPLD
designs, as they may lead to timing problems.
WARNING:Xst:737 - Found 1-bit latch for signal <PA_Z3<70>>. Latches may be generated from
incomplete case or if statements. We do not recommend the use of latches in FPGA/CPLD
designs, as they may lead to timing problems.
WARNING:Xst:737 - Found 1-bit latch for signal <PA_Z3<69>>. Latches may be generated from
incomplete case or if statements. We do not recommend the use of latches in FPGA/CPLD
designs, as they may lead to timing problems.
WARNING:Xst:737 - Found 1-bit latch for signal <PA_Z3<68>>. Latches may be generated from
incomplete case or if statements. We do not recommend the use of latches in FPGA/CPLD
designs, as they may lead to timing problems.
WARNING:Xst:737 - Found 1-bit latch for signal <PA_Z3<67>>. Latches may be generated from
incomplete case or if statements. We do not recommend the use of latches in FPGA/CPLD
designs, as they may lead to timing problems.
WARNING:Xst:737 - Found 1-bit latch for signal <PA_Z3<66>>. Latches may be generated from
incomplete case or if statements. We do not recommend the use of latches in FPGA/CPLD
designs, as they may lead to timing problems.
```

```
WARNING:Xst:737 - Found 1-bit latch for signal <PA_Z3<65>>. Latches may be generated from
incomplete case or if statements. We do not recommend the use of latches in FPGA/CPLD
designs, as they may lead to timing problems.
WARNING:Xst:737 - Found 1-bit latch for signal <PA_Z3<64>>. Latches may be generated from
incomplete case or if statements. We do not recommend the use of latches in FPGA/CPLD
designs, as they may lead to timing problems.
WARNING:Xst:737 - Found 1-bit latch for signal <PA_Z3<63>>. Latches may be generated from
incomplete case or if statements. We do not recommend the use of latches in FPGA/CPLD
designs, as they may lead to timing problems.
WARNING:Xst:737 - Found 1-bit latch for signal <PA_Z3<62>>. Latches may be generated from
incomplete case or if statements. We do not recommend the use of latches in FPGA/CPLD
designs, as they may lead to timing problems.
WARNING:Xst:737 - Found 1-bit latch for signal <PA_Z3<61>>. Latches may be generated from
incomplete case or if statements. We do not recommend the use of latches in FPGA/CPLD
designs, as they may lead to timing problems.
WARNING:Xst:737 - Found 1-bit latch for signal <PA_Z3<60>>. Latches may be generated from
incomplete case or if statements. We do not recommend the use of latches in FPGA/CPLD
designs, as they may lead to timing problems.
WARNING:Xst:737 - Found 1-bit latch for signal <PA_Z3<59>>. Latches may be generated from
incomplete case or if statements. We do not recommend the use of latches in FPGA/CPLD
designs, as they may lead to timing problems.
WARNING:Xst:737 - Found 1-bit latch for signal <PA_Z3<58>>. Latches may be generated from
incomplete case or if statements. We do not recommend the use of latches in FPGA/CPLD
designs, as they may lead to timing problems.
WARNING:Xst:737 - Found 1-bit latch for signal <PA_Z3<57>>. Latches may be generated from
incomplete case or if statements. We do not recommend the use of latches in FPGA/CPLD
designs, as they may lead to timing problems.
WARNING:Xst:737 - Found 1-bit latch for signal <PA_Z3<56>>. Latches may be generated from
incomplete case or if statements. We do not recommend the use of latches in FPGA/CPLD
designs, as they may lead to timing problems.
WARNING:Xst:737 - Found 1-bit latch for signal <PA_Z3<55>>. Latches may be generated from
incomplete case or if statements. We do not recommend the use of latches in FPGA/CPLD
designs, as they may lead to timing problems.
WARNING:Xst:737 - Found 1-bit latch for signal <PA_Z3<54>>. Latches may be generated from
incomplete case or if statements. We do not recommend the use of latches in FPGA/CPLD
designs, as they may lead to timing problems.
WARNING:Xst:737 - Found 1-bit latch for signal <PA_Z3<53>>. Latches may be generated from
incomplete case or if statements. We do not recommend the use of latches in FPGA/CPLD
designs, as they may lead to timing problems.
WARNING:Xst:737 - Found 1-bit latch for signal <PA_Z3<52>>. Latches may be generated from
incomplete case or if statements. We do not recommend the use of latches in FPGA/CPLD
designs, as they may lead to timing problems.
WARNING:Xst:737 - Found 1-bit latch for signal <PA_Z3<51>>. Latches may be generated from
incomplete case or if statements. We do not recommend the use of latches in FPGA/CPLD
designs, as they may lead to timing problems.
WARNING:Xst:737 - Found 1-bit latch for signal <PA_Z3<50>>. Latches may be generated from
incomplete case or if statements. We do not recommend the use of latches in FPGA/CPLD
designs, as they may lead to timing problems.
WARNING:Xst:737 - Found 1-bit latch for signal <PA_Z3<49>>. Latches may be generated from
incomplete case or if statements. We do not recommend the use of latches in FPGA/CPLD
designs, as they may lead to timing problems.
WARNING:Xst:737 - Found 1-bit latch for signal <PA_Z3<48>>. Latches may be generated from
incomplete case or if statements. We do not recommend the use of latches in FPGA/CPLD
designs, as they may lead to timing problems.
WARNING:Xst:737 - Found 1-bit latch for signal <PA_Z3<47>>. Latches may be generated from
incomplete case or if statements. We do not recommend the use of latches in FPGA/CPLD
designs, as they may lead to timing problems.
WARNING:Xst:737 - Found 1-bit latch for signal <PA_Z3<46>>. Latches may be generated from
incomplete case or if statements. We do not recommend the use of latches in FPGA/CPLD
designs, as they may lead to timing problems.
WARNING:Xst:737 - Found 1-bit latch for signal <PA_Z3<45>>. Latches may be generated from
incomplete case or if statements. We do not recommend the use of latches in FPGA/CPLD
designs, as they may lead to timing problems.
WARNING:Xst:737 - Found 1-bit latch for signal <PA_Z3<44>>. Latches may be generated from
incomplete case or if statements. We do not recommend the use of latches in FPGA/CPLD
designs, as they may lead to timing problems.
WARNING:Xst:737 - Found 1-bit latch for signal <PA_Z3<43>>. Latches may be generated from
incomplete case or if statements. We do not recommend the use of latches in FPGA/CPLD
designs, as they may lead to timing problems.
WARNING:Xst:737 - Found 1-bit latch for signal <PA_Z3<42>>. Latches may be generated from
incomplete case or if statements. We do not recommend the use of latches in FPGA/CPLD
```

designs, as they may lead to timing problems.
WARNING:Xst:737 - Found 1-bit latch for signal <PA_Z3<41>>. Latches may be generated from incomplete case or if statements. We do not recommend the use of latches in FPGA/CPLD designs, as they may lead to timing problems.
WARNING:Xst:737 - Found 1-bit latch for signal <PA_Z3<40>>. Latches may be generated from incomplete case or if statements. We do not recommend the use of latches in FPGA/CPLD designs, as they may lead to timing problems.
WARNING:Xst:737 - Found 1-bit latch for signal <PA_Z3<39>>. Latches may be generated from incomplete case or if statements. We do not recommend the use of latches in FPGA/CPLD designs, as they may lead to timing problems.
WARNING:Xst:737 - Found 1-bit latch for signal <PA_Z3<38>>. Latches may be generated from incomplete case or if statements. We do not recommend the use of latches in FPGA/CPLD designs, as they may lead to timing problems.
WARNING:Xst:737 - Found 1-bit latch for signal <PA_Z3<37>>. Latches may be generated from incomplete case or if statements. We do not recommend the use of latches in FPGA/CPLD designs, as they may lead to timing problems.
WARNING:Xst:737 - Found 1-bit latch for signal <PA_Z3<36>>. Latches may be generated from incomplete case or if statements. We do not recommend the use of latches in FPGA/CPLD designs, as they may lead to timing problems.
WARNING:Xst:737 - Found 1-bit latch for signal <PA_Z3<35>>. Latches may be generated from incomplete case or if statements. We do not recommend the use of latches in FPGA/CPLD designs, as they may lead to timing problems.
WARNING:Xst:737 - Found 1-bit latch for signal <PA_Z3<34>>. Latches may be generated from incomplete case or if statements. We do not recommend the use of latches in FPGA/CPLD designs, as they may lead to timing problems.
WARNING:Xst:737 - Found 1-bit latch for signal <PA_Z3<33>>. Latches may be generated from incomplete case or if statements. We do not recommend the use of latches in FPGA/CPLD designs, as they may lead to timing problems.
WARNING:Xst:737 - Found 1-bit latch for signal <PA_Z3<32>>. Latches may be generated from incomplete case or if statements. We do not recommend the use of latches in FPGA/CPLD designs, as they may lead to timing problems.
WARNING:Xst:737 - Found 1-bit latch for signal <PA_Z3<31>>. Latches may be generated from incomplete case or if statements. We do not recommend the use of latches in FPGA/CPLD designs, as they may lead to timing problems.
WARNING:Xst:737 - Found 1-bit latch for signal <PA_Z3<30>>. Latches may be generated from incomplete case or if statements. We do not recommend the use of latches in FPGA/CPLD designs, as they may lead to timing problems.
WARNING:Xst:737 - Found 1-bit latch for signal <PA_Z3<29>>. Latches may be generated from incomplete case or if statements. We do not recommend the use of latches in FPGA/CPLD designs, as they may lead to timing problems.
WARNING:Xst:737 - Found 1-bit latch for signal <PA_Z3<28>>. Latches may be generated from incomplete case or if statements. We do not recommend the use of latches in FPGA/CPLD designs, as they may lead to timing problems.
WARNING:Xst:737 - Found 1-bit latch for signal <PA_Z3<27>>. Latches may be generated from incomplete case or if statements. We do not recommend the use of latches in FPGA/CPLD designs, as they may lead to timing problems.
WARNING:Xst:737 - Found 1-bit latch for signal <PA_Z3<26>>. Latches may be generated from incomplete case or if statements. We do not recommend the use of latches in FPGA/CPLD designs, as they may lead to timing problems.
WARNING:Xst:737 - Found 1-bit latch for signal <PA_Z3<25>>. Latches may be generated from incomplete case or if statements. We do not recommend the use of latches in FPGA/CPLD designs, as they may lead to timing problems.
WARNING:Xst:737 - Found 1-bit latch for signal <PA_Z3<24>>. Latches may be generated from incomplete case or if statements. We do not recommend the use of latches in FPGA/CPLD designs, as they may lead to timing problems.
WARNING:Xst:737 - Found 1-bit latch for signal <PA_Z3<23>>. Latches may be generated from incomplete case or if statements. We do not recommend the use of latches in FPGA/CPLD designs, as they may lead to timing problems.
WARNING:Xst:737 - Found 1-bit latch for signal <PA_Z3<22>>. Latches may be generated from incomplete case or if statements. We do not recommend the use of latches in FPGA/CPLD designs, as they may lead to timing problems.
WARNING:Xst:737 - Found 1-bit latch for signal <PA_Z3<21>>. Latches may be generated from incomplete case or if statements. We do not recommend the use of latches in FPGA/CPLD designs, as they may lead to timing problems.
WARNING:Xst:737 - Found 1-bit latch for signal <PA_Z3<20>>. Latches may be generated from incomplete case or if statements. We do not recommend the use of latches in FPGA/CPLD designs, as they may lead to timing problems.
WARNING:Xst:737 - Found 1-bit latch for signal <PA_Z3<19>>. Latches may be generated from incomplete case or if statements. We do not recommend the use of latches in FPGA/CPLD designs, as they may lead to timing problems.
WARNING:Xst:737 - Found 1-bit latch for signal <PA_Z3<18>>. Latches may be generated from

```
incomplete case or if statements. We do not recommend the use of latches in FPGA/CPLD
designs, as they may lead to timing problems.
WARNING:Xst:737 - Found 1-bit latch for signal <PA_Z3<17>>. Latches may be generated from
incomplete case or if statements. We do not recommend the use of latches in FPGA/CPLD
designs, as they may lead to timing problems.
WARNING:Xst:737 - Found 1-bit latch for signal <PA_Z3<16>>. Latches may be generated from
incomplete case or if statements. We do not recommend the use of latches in FPGA/CPLD
designs, as they may lead to timing problems.
WARNING:Xst:737 - Found 1-bit latch for signal <PA_Z3<15>>. Latches may be generated from
incomplete case or if statements. We do not recommend the use of latches in FPGA/CPLD
designs, as they may lead to timing problems.
WARNING:Xst:737 - Found 1-bit latch for signal <PA_Z3<14>>. Latches may be generated from
incomplete case or if statements. We do not recommend the use of latches in FPGA/CPLD
designs, as they may lead to timing problems.
WARNING:Xst:737 - Found 1-bit latch for signal <PA_Z3<13>>. Latches may be generated from
incomplete case or if statements. We do not recommend the use of latches in FPGA/CPLD
designs, as they may lead to timing problems.
WARNING:Xst:737 - Found 1-bit latch for signal <PA_Z3<12>>. Latches may be generated from
incomplete case or if statements. We do not recommend the use of latches in FPGA/CPLD
designs, as they may lead to timing problems.
WARNING:Xst:737 - Found 1-bit latch for signal <PA_Z3<11>>. Latches may be generated from
incomplete case or if statements. We do not recommend the use of latches in FPGA/CPLD
designs, as they may lead to timing problems.
WARNING:Xst:737 - Found 1-bit latch for signal <PA_Z3<10>>. Latches may be generated from
incomplete case or if statements. We do not recommend the use of latches in FPGA/CPLD
designs, as they may lead to timing problems.
WARNING:Xst:737 - Found 1-bit latch for signal <PA_Z3<9>>. Latches may be generated from
incomplete case or if statements. We do not recommend the use of latches in FPGA/CPLD
designs, as they may lead to timing problems.
WARNING:Xst:737 - Found 1-bit latch for signal <PA_Z3<8>>. Latches may be generated from
incomplete case or if statements. We do not recommend the use of latches in FPGA/CPLD
designs, as they may lead to timing problems.
WARNING:Xst:737 - Found 1-bit latch for signal <PA_Z3<7>>. Latches may be generated from
incomplete case or if statements. We do not recommend the use of latches in FPGA/CPLD
designs, as they may lead to timing problems.
WARNING:Xst:737 - Found 1-bit latch for signal <PA_Z3<6>>. Latches may be generated from
incomplete case or if statements. We do not recommend the use of latches in FPGA/CPLD
designs, as they may lead to timing problems.
WARNING:Xst:737 - Found 1-bit latch for signal <PA_Z3<5>>. Latches may be generated from
incomplete case or if statements. We do not recommend the use of latches in FPGA/CPLD
designs, as they may lead to timing problems.
WARNING:Xst:737 - Found 1-bit latch for signal <PA_Z3<4>>. Latches may be generated from
incomplete case or if statements. We do not recommend the use of latches in FPGA/CPLD
designs, as they may lead to timing problems.
WARNING:Xst:737 - Found 1-bit latch for signal <PA_Z3<3>>. Latches may be generated from
incomplete case or if statements. We do not recommend the use of latches in FPGA/CPLD
designs, as they may lead to timing problems.
WARNING:Xst:737 - Found 1-bit latch for signal <PA_Z3<2>>. Latches may be generated from
incomplete case or if statements. We do not recommend the use of latches in FPGA/CPLD
designs, as they may lead to timing problems.
WARNING:Xst:737 - Found 1-bit latch for signal <PA_Z3<1>>. Latches may be generated from
incomplete case or if statements. We do not recommend the use of latches in FPGA/CPLD
designs, as they may lead to timing problems.
WARNING:Xst:737 - Found 1-bit latch for signal <PA_Z3<0>>. Latches may be generated from
incomplete case or if statements. We do not recommend the use of latches in FPGA/CPLD
designs, as they may lead to timing problems.
WARNING:Xst:737 - Found 1-bit latch for signal <PA_X3<162>>. Latches may be generated from
incomplete case or if statements. We do not recommend the use of latches in FPGA/CPLD
designs, as they may lead to timing problems.
    Summary:
    inferred 489 Latch(s).
    inferred 489 Multiplexer(s).
Unit <MUX_1_new> synthesized.

Synthesizing Unit <MUX_2_new>.
    Related source file is
    "G:\VHDL_July_2016\PM_PDPA_BF_P_All\PM_PDPA_BF_P_B_163\MUX_2_new.vhd".
    Summary:
    no macro.
Unit <MUX_2_new> synthesized.
```

```
Synthesizing Unit <Reg_MUX_3>.
    Related source file is
    "G:\VHDL_July_2016\PM_PDPA_BF_P_All\PM_PDPA_BF_P_B_163\Reg_MUX_3.vhd".
    Found 163-bit register for signal <QYout>.
    Found 163-bit register for signal <QZout>.
    Found 163-bit register for signal <QXout>.
    Summary:
    inferred 489 D-type flip-flop(s).
Unit <Reg_MUX_3> synthesized.

=========================================================================
HDL Synthesis Report

Macro Statistics
# Adders/Subtractors                                    : 1
 8-bit subtractor                                       : 1
# Registers                                             : 8
 1-bit register                                         : 1
 163-bit register                                       : 6
 8-bit register                                         : 1
# Latches                                               : 489
 1-bit latch                                            : 489
# Multiplexers                                          : 491
 1-bit 2-to-1 multiplexer                               : 489
 2-bit 2-to-1 multiplexer                               : 1
 8-bit 2-to-1 multiplexer                               : 1
# Xors                                                  : 7835
 163-bit xor2                                           : 11
 164-bit xor2                                           : 7824

=========================================================================

=========================================================================
*                       Advanced HDL Synthesis                          *
=========================================================================


Synthesizing (advanced) Unit <ECC_TOP_B_163>.
The following registers are absorbed into counter <count>: 1 register on signal <count>.
Unit <ECC_TOP_B_163> synthesized (advanced).

=========================================================================
Advanced HDL Synthesis Report

Macro Statistics
# Counters                                              : 1
 8-bit down counter                                     : 1
# Registers                                             : 979
 Flip-Flops                                             : 979
# Multiplexers                                          : 489
 1-bit 2-to-1 multiplexer                               : 489
# Xors                                                  : 7835
 163-bit xor2                                           : 11
 164-bit xor2                                           : 7824

=========================================================================

=========================================================================
*                         Low Level Synthesis                           *
=========================================================================

Optimizing unit <Reg_MUX_3> ...

Optimizing unit <ECC_TOP_B_163> ...

Optimizing unit <PD_PA_BF> ...

Optimizing unit <pol_SQ> ...

Optimizing unit <pol_mult> ...
```

```
Optimizing unit <MUX_1_new> ...

Mapping all equations...
Building and optimizing final netlist ...
Found area constraint ratio of 100 (+ 5) on block ECC_TOP_B_163, actual ratio is 29.

Final Macro Processing ...

=========================================================================
Final Register Report

Macro Statistics
# Registers                                            : 987
 Flip-Flops                                            : 987

=========================================================================

=========================================================================
*                            Partition Report                           *
=========================================================================

Partition Implementation Status
-------------------------------

  No Partitions were found in this design.

-------------------------------

=========================================================================
*                             Design Summary                            *
=========================================================================

Top Level Output File Name         : ECC_TOP_B_163.ngc

Primitive and Black Box Usage:
------------------------------
# BELS                             : 232144
#      GND                         : 14
#      INV                         : 5
#      LUT2                        : 1726
#      LUT3                        : 2914
#      LUT4                        : 24072
#      LUT5                        : 9860
#      LUT6                        : 193424
#      MUXCY                       : 117
#      VCC                         : 4
#      XORCY                       : 8
# FlipFlops/Latches                : 1476
#      FDC                         : 494
#      FDCE                        : 490
#      FDP                         : 3
#      LD                          : 489
# Clock Buffers                    : 2
#      BUFG                        : 1
#      BUFGP                       : 1
# IO Buffers                       : 491
#      IBUF                        : 1
#      OBUF                        : 490

Device utilization summary:
---------------------------

Selected Device : 7vx485tffg1761-2


Slice Logic Utilization:
 Number of Slice Registers:            1476  out of  607200     0%
 Number of Slice LUTs:               232001  out of  303600    76%
    Number used as Logic:            232001  out of  303600    76%

Slice Logic Distribution:
```

```
 Number of LUT Flip Flop pairs used:  232492
   Number with an unused Flip Flop:   231016  out of  232492    99%
   Number with an unused LUT:           491  out of  232492     0%
   Number of fully used LUT-FF pairs:   985  out of  232492     0%
   Number of unique control sets:         3

IO Utilization:
 Number of IOs:                         493
 Number of bonded IOBs:                 492  out of    700    70%

Specific Feature Utilization:
 Number of BUFG/BUFGCTRLs:                2  out of     32     6%

---------------------------
Partition Resource Summary:
---------------------------

  No Partitions were found in this design.

---------------------------



=========================================================================
Timing Report

NOTE: THESE TIMING NUMBERS ARE ONLY A SYNTHESIS ESTIMATE.
      FOR ACCURATE TIMING INFORMATION PLEASE REFER TO THE TRACE REPORT
      GENERATED AFTER PLACE-and-ROUTE.

Clock Information:
------------------
-----------------------------------------------------------------------------------------+------
------------------------+-------+
Clock Signal                                                                             |
Clock buffer(FF name)        | Load  |
-----------------------------------------------------------------------------------------+------
------------------------+-------+
clk                                                                                      |
BUFGP                        | 987   |
uut_MUX1_new/GND_439_o_GND_439_o_OR_165_o(uut_MUX1_new/GND_439_o_GND_439_o_OR_165_o1:O)|
BUFG(*)(uut_MUX1_new/PA_Z3_1)| 489   |
-----------------------------------------------------------------------------------------+------
------------------------+-------+
(*) This 1 clock signal(s) are generated by combinatorial logic,
and XST is not able to identify which are the primary clock signals.
Please use the CLOCK_SIGNAL constraint to specify the clock signal(s) generated by
combinatorial logic.

Asynchronous Control Signals Information:
----------------------------------------
No asynchronous control signals found in this design

Timing Summary:
---------------
Speed Grade: -2

   Minimum period: 1.892ns (Maximum Frequency: 528.583MHz)
   Minimum input arrival time before clock: 0.838ns
   Maximum output required time after clock: 0.580ns
   Maximum combinational path delay: No path found

Timing Details:
---------------
All values displayed in nanoseconds (ns)

=========================================================================
Timing constraint: Default period analysis for Clock 'clk'
  Clock period: 1.892ns (frequency: 528.583MHz)
  Total number of paths / destination ports: 4591 / 988
-------------------------------------------------------------------------
Delay:               1.892ns (Levels of Logic = 10)
```

```
  Source:            count_7 (FF)
  Destination:       count_7 (FF)
  Source Clock:      clk rising
  Destination Clock: clk rising

  Data Path: count_7 to count_7
                                Gate     Net
    Cell:in->out      fanout   Delay   Delay  Logical Name (Net Name)
    ----------------------------------------  ------------
     FDP:C->Q              3   0.236   0.507  count_7 (count_7)
     LUT3:I0->O            3   0.043   0.362  GND_7_o_count[7]_equal_1_o<7>_SW0 (N2)
     LUT6:I5->O            2   0.043   0.344  GND_7_o_count[7]_equal_1_o<7>
     (GND_7_o_count[7]_equal_1_o)
     MUXCY:CI->O           1   0.014   0.000  Mcount_count_cy<0> (Mcount_count_cy<0>)
     MUXCY:CI->O           1   0.014   0.000  Mcount_count_cy<1> (Mcount_count_cy<1>)
     MUXCY:CI->O           1   0.014   0.000  Mcount_count_cy<2> (Mcount_count_cy<2>)
     MUXCY:CI->O           1   0.014   0.000  Mcount_count_cy<3> (Mcount_count_cy<3>)
     MUXCY:CI->O           1   0.014   0.000  Mcount_count_cy<4> (Mcount_count_cy<4>)
     MUXCY:CI->O           1   0.014   0.000  Mcount_count_cy<5> (Mcount_count_cy<5>)
     MUXCY:CI->O           0   0.014   0.000  Mcount_count_cy<6> (Mcount_count_cy<6>)
     XORCY:CI->O           1   0.262   0.000  Mcount_count_xor<7> (Mcount_count7)
     FDP:D                    -0.000          count_7
    ----------------------------------------
    Total                      1.892ns (0.678ns logic, 1.213ns route)
                                       (35.9% logic, 64.1% route)

=========================================================================
Timing constraint: Default OFFSET IN BEFORE for Clock 'clk'
  Total number of paths / destination ports: 987 / 987
-------------------------------------------------------------------------
Offset:              0.838ns (Levels of Logic = 1)
  Source:            reset (PAD)
  Destination:       done (FF)
  Destination Clock: clk rising

  Data Path: reset to done
                                Gate     Net
    Cell:in->out      fanout   Delay   Delay  Logical Name (Net Name)
    ----------------------------------------  ------------
     IBUF:I->O           987   0.000   0.574  reset_IBUF (reset_IBUF)
     FDCE:CLR                  0.264          done
    ----------------------------------------
    Total                      0.838ns (0.264ns logic, 0.574ns route)
                                       (31.5% logic, 68.5% route)

=========================================================================
Timing constraint: Default OFFSET OUT AFTER for Clock 'clk'
  Total number of paths / destination ports: 490 / 490
-------------------------------------------------------------------------
Offset:              0.580ns (Levels of Logic = 1)
  Source:            QX_162 (FF)
  Destination:       QX<162> (PAD)
  Source Clock:      clk rising

  Data Path: QX_162 to QX<162>
                                Gate     Net
    Cell:in->out      fanout   Delay   Delay  Logical Name (Net Name)
    ----------------------------------------  ------------
     FDCE:C->Q             2   0.236   0.344  QX_162 (QX_162)
     OBUF:I->O                 0.000          QX_162_OBUF (QX<162>)
    ----------------------------------------
    Total                      0.580ns (0.236ns logic, 0.344ns route)
                                       (40.7% logic, 59.3% route)

=========================================================================

Cross Clock Domains Report:
--------------------------

Clock to Setup on destination clock clk
---------------------------------------+---------+---------+---------+---------+
```

```
                                         | Src:Rise| Src:Fall| Src:Rise| Src:Fall|
Source Clock                             |Dest:Rise|Dest:Rise|Dest:Fall|Dest:Fall|
-----------------------------------------+---------+---------+---------+---------+
clk                                      |    1.892|         |         |         |
uut_MUX1_new/GND_439_o_GND_439_o_OR_165_o|         |    0.873|         |         |
-----------------------------------------+---------+---------+---------+---------+

Clock to Setup on destination clock uut_MUX1_new/GND_439_o_GND_439_o_OR_165_o
---------------+---------+---------+---------+---------+
               | Src:Rise| Src:Fall| Src:Rise| Src:Fall|
Source Clock   |Dest:Rise|Dest:Rise|Dest:Fall|Dest:Fall|
---------------+---------+---------+---------+---------+
clk            |         |         |   28.858|         |
---------------+---------+---------+---------+---------+

=========================================================================


Total REAL time to Xst completion: 682.00 secs
Total CPU time to Xst completion: 682.00 secs

-->

Total memory usage is 2372520 kilobytes

Number of errors   :    0 (   0 filtered)
Number of warnings :  490 (   0 filtered)
Number of infos    :    0 (   0 filtered)
```
